# MicroExonator enables systematic discovery and quantification of microexons across mouse embryonic development

**DOI:** 10.1186/s13059-020-02246-2

**Published:** 2021-01-22

**Authors:** Guillermo E. Parada, Roberto Munita, Ilias Georgakopoulos-Soares, Hugo J. R. Fernandes, Veronika R. Kedlian, Emmanouil Metzakopian, Maria Estela Andres, Eric A. Miska, Martin Hemberg

**Affiliations:** 1 Wellcome Sanger Institute, Wellcome Genome Campus, Cambridge, CB10 1SA UK; 2 Wellcome Trust Cancer Research UK Gurdon Institute, University of Cambridge, Tennis Court Road, Cambridge, CB2 1QN UK; 3 Department of Cellular and Molecular Biology, Faculty of Biological Sciences, Pontificia Universidad Católica de Chile, Santiago, Chile; 4 Department of Bioengineering and Therapeutic Sciences, University of California San Francisco, San Francisco, CA 94158 USA; 5 UK Dementia Research Institute, Department of Clinical Neurosciences, University of Cambridge, Cambridge, CB2 0AH UK; 6 Department of Genetics, University of Cambridge, Downing Street, Cambridge, CB2 3EH UK

**Keywords:** Microexons, Splicing, Alternative splicing, Neuronal development, Single-cell, Reproducible software

## Abstract

**Background:**

Microexons, exons that are ≤ 30 nucleotides, are a highly conserved and dynamically regulated set of cassette exons. They have key roles in nervous system development and function, as evidenced by recent results demonstrating the impact of microexons on behaviour and cognition. However, microexons are often overlooked due to the difficulty of detecting them using standard RNA-seq aligners.

**Results:**

Here, we present MicroExonator, a novel pipeline for reproducible de novo discovery and quantification of microexons. We process 289 RNA-seq datasets from eighteen mouse tissues corresponding to nine embryonic and postnatal stages, providing the most comprehensive survey of microexons available for mice. We detect 2984 microexons, 332 of which are differentially spliced throughout mouse embryonic brain development, including 29 that are not present in mouse transcript annotation databases. Unsupervised clustering of microexons based on their inclusion patterns segregates brain tissues by developmental time, and further analysis suggests a key function for microexons in axon growth and synapse formation. Finally, we analyse single-cell RNA-seq data from the mouse visual cortex, and for the first time, we report differential inclusion between neuronal subpopulations, suggesting that some microexons could be cell type-specific.

**Conclusions:**

MicroExonator facilitates the investigation of microexons in transcriptome studies, particularly when analysing large volumes of data. As a proof of principle, we use MicroExonator to analyse a large collection of both mouse bulk and single-cell RNA-seq datasets. The analyses enabled the discovery of previously uncharacterized microexons, and our study provides a comprehensive microexon inclusion catalogue during mouse development.

**Supplementary Information:**

The online version contains supplementary material available at 10.1186/s13059-020-02246-2.

## Background

In eukaryotes, mRNA processing is a key regulatory step of gene expression [1]. Alternative splicing is arguably one of the most important processes affecting the vast majority of transcripts in higher eukaryotes [2]. Consequently, alternative splicing impinges directly onto numerous biological processes such as cell cycle, cell differentiation, development, sex, circadian rhythm, response to environmental change, pathogen exposure and disease [3–6]. High-throughput RNA sequencing (RNA-seq) coupled with efficient computational methods has facilitated annotation of low abundance and tissue-specific transcripts and thus revolutionized our understanding of alternative and non-canonical splicing events [7].

In vertebrates, dramatic changes in alternative splicing control neurogenesis, neuronal migration, synaptogenesis and synaptic function [8]. In particular, it was shown that short exons tend to be included more frequently in the central nervous system [9, 10]. Recently, it was also shown that extremely short exons, known as microexons, herein defined as exons ≤ 30 nucleotides, are the most highly conserved component of neuronal alternative splicing during development [11]. Importantly, microexon inclusion has been proposed to have a key regulatory role during brain development, having an influence over neurite outgrowth, cortical layering and axon guidance [12–17]. However, the quantification of microexons using RNA-seq remains challenging due to their incomplete annotation [18].

The first algorithms for genome-wide microexon discovery were based on EST/cDNA misalignment corrections and discovered 170 microexons [19, 20]. De novo discovery of microexon insertions by aligning short segments of mRNA using standard software is difficult because most algorithms require a perfectly matching seed sequence that often cannot fit within a single microexon. Detection can be improved by reducing the size of alignment seeds, as was done for Olego which enabled the identification of 630 novel microexons 9–27 nucleotides (nt) in mice [21]. Another strategy for increasing the sensitivity of microexon discovery is to directly map RNA-seq reads to libraries of annotated splice junctions [11, 22], but the bioinformatic pipelines used in these seminal studies have not been released to the public domain. Today, VAST-TOOLS is the most widely used tool for microexon quantification from RNA-seq data [23]. However, a significant restriction of VAST-TOOLS is that it can only identify microexons that are annotated in VastDB [23], which is only available for a limited number of species.

**Fig. 1:**
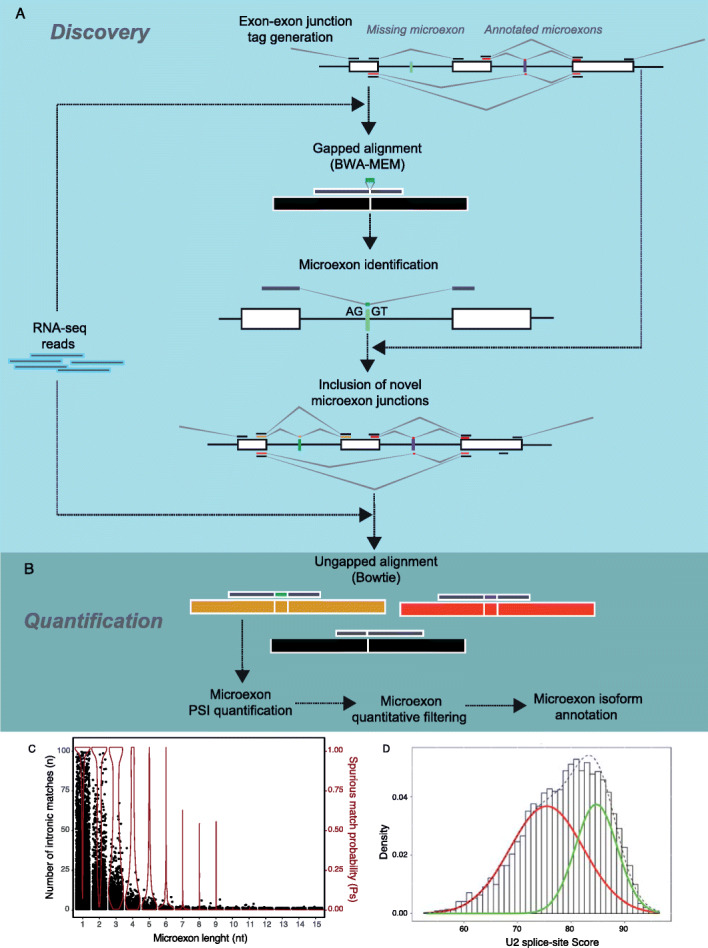
Overview of the MicroExonator workflow. **a** To discover unannotated microexons, RNA-seq reads are aligned with BWA-MEM to the annotated splice junctions. The resulting alignments are postprocessed to discover novel microexons flanked by canonical U2-type GT-AG splice sites. **b** Both putative novel and annotated microexons are quantified and filtered to produce a final list of microexon incorporation into transcript models which can be used for downstream analysis. **c** Number of intronic matches and distribution of spurious match probability across microexon lengths. **d** A two-component Gaussian mixture is used to fit the U2 consensus splicing score distribution. The red line shows the Gaussian with lower mean U2 splice score, which is assumed to consist mainly of false positives, while the green line denotes the Gaussian with higher mean U2 splice-site score

Here, we introduce MicroExonator, a computational workflow for discovery and quantification of microexons using RNA-seq data. MicroExonator employs a two-step procedure whereby it first carries out a de novo search for unannotated microexons and subsequently quantifies both new and previously annotated microexons (Fig. 1). Using simulations, we show that MicroExonator outperforms other available tools, both in terms of sensitivity and specificity. We then analyse mouse embryonic development RNA-seq datasets, and we identify a total of 2984 microexons, 37% of these are not previously annotated in GENCODE [24] mouse transcript models. We focus our analysis on 326 microexons that change during neuronal development, and 18% of which are not present in VastDB. Our analysis shows a pattern of orchestrated microexon inclusion during brain development as evidenced by the high degree of connectivity of the protein-protein interaction network encompassing the genes that contain microexons. We also directly demonstrate the high degree of conservation of microexons by analysing 23 zebrafish brain RNA-seq samples where we detect 348 zebrafish microexons that were conserved in mice, including 54 that were not annotated in the Ensembl gene annotations. Finally, we apply MicroExonator to single-cell RNA-seq data, and we demonstrate that some microexons are not only tissue-specific, but also cell type-specific.

## Results

### Reproducible detection and quantification of microexons using RNA-seq data

MicroExonator is a computational workflow that integrates several existing software packages with custom python and R scripts to perform discovery and quantification of microexons using RNA-seq data. MicroExonator can analyse RNA-seq data stored locally, but it can also fetch any RNA-seq datasets deposited in the NCBI Short Read Archive or other web-based repositories. As microexon annotations remain incomplete and sometimes inconsistent across different transcript annotations, MicroExonator can incorporate prior information from multiple databases such as RefSeq [25], GENCODE [24], ENSEMBL [26], UCSC [27] or VastDB [23]. To discover putative novel microexons, reads are first mapped using BWA-MEM [28] to a reference library of splice junction sequences. Misaligned reads are then searched for insertions located at exon-exon junctions. Detected insertions are retained if they can be successfully mapped to the corresponding intronic region with flanking canonical U2-type splicing dinucleotides [29] (Fig. 1a). To maximize the number of reads that can be assigned to each splice site, annotated and putative novel microexon sequences are integrated as part of the initial splice tags where they were detected. Reads are re-aligned with Bowtie, performing a fast but sensitive mapping of reads which is further processed to quantify percent spliced in (PSI) microexon values and perform quantitative filters (Fig. 1b, Additional file 1: Fig. S1).

MicroExonator employs several filters (Fig. 1b–d) to remove spurious matches to intronic sequences which may arise due to sequencing errors [20]. To illustrate these filters, we ran the initial mapping steps over the total RNA-seq from mice (289 RNA-seq samples from 18 different murine tissues and 1657 single cells from mice visual cortex [30–32]) used in this paper. As the first filtering step, only those insertions that can be detected in a minimum number of independent samples (i.e. technical or biological replicates, three samples is set as default) are considered. Additionally, MicroExonator scores the sequence context of the detected canonical splice sites to measure the strength of their upstream and downstream splice junctions as quantified by a splicing strength score [33], and a Gaussian mixture model is used to exclude matches that have weak splice site signals (Fig. 1d). Finally, MicroExonator integrates the splicing strength, probability of spurious intronic matching and conservation (optional) in an adaptive filtering function to remove low confidence candidates (Additional file 1: Fig. S2).

To ensure that analyses are fully reproducible, MicroExonator was implemented using the SnakeMake workflow manager [34]. As MicroExonator may require significant computational resources, SnakeMake also facilitates running computational analyses on high-performance computer clusters by automating the scheduling of interdependent jobs. SnakeMake itself can be installed from Bioconda [35], and it can initiate MicroExonator directly after downloading the code from our GitHub repository (https://github.com/hemberg-lab/MicroExonator). During runtime, MicroExonator creates custom conda virtual environments which contain specific combinations of software packages found in BioConda repositories to ensure that the same versions are consistently used.

### Benchmarking of computational methods for microexon discovery

To compare MicroExonator with other methods, we incorporated a set of synthetic microexons into the GENCODE gene annotation. The microexon sizes were drawn from the previously reported distributions [11, 22] with a greater abundance of in-frame microexons. We also modified the genomic sequence by replacing the intronic flanking regions of simulated microexons with sequences extracted from annotated splice sites. To simulate spurious microexons, we randomly incorporated insertions across splice junctions, as these inserted sequences have the potential to map to intronic spaces.

**Fig. 2:**
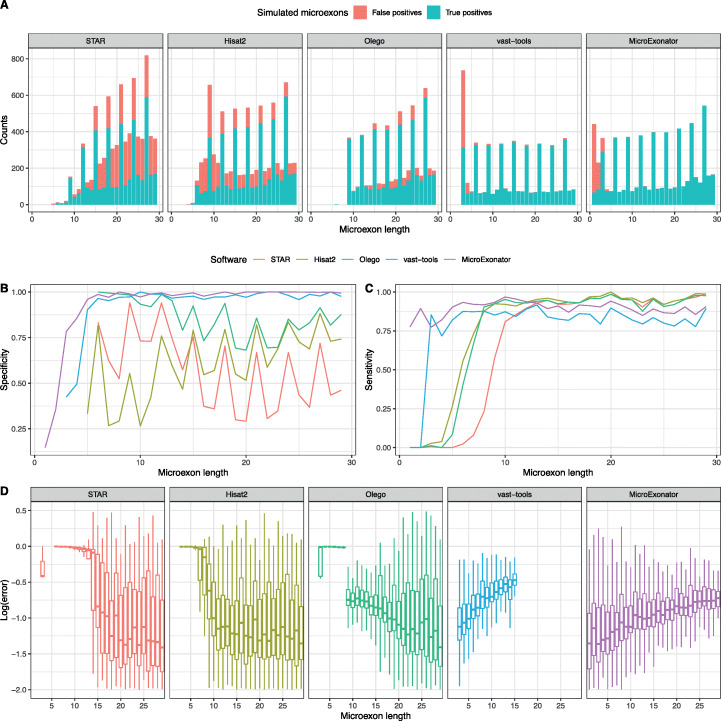
Evaluation of microexon discovery performance of RNA-seq aligners, VAST-TOOLS and MicroExonator using synthetic data. **a** Size distribution of simulated microexons that were detected by the different software. **b**, **c** Specificity and sensitivity of detected simulated microexons. **d** Log10 error PSI values show the accuracy of the microexon quantification

We used Polyester [36] to simulate reads with a standard Illumina sequencing error rate and processed them using either MicroExonator, VAST-TOOLS [11], Hisat2 [37], STAR [38] or Olego [21]. Our results show that both VAST-TOOLS and MicroExonator performed significantly better than stand-alone RNA-seq aligners (Fig. 2a–c), demonstrating the benefit of using a dedicated computational workflow for microexon discovery. Even though all three aligners could detect a significant fraction of the simulated microexons, they are all limited in their ability to discover very short microexons; STAR’s sensitivity drastically declines for microexons < 10 nt, while the sensitivity of Hisat2 and Olego drops for microexons < 8 nt (Fig. 2b). By contrast, VAST-TOOLS could detect microexons 3 nt or longer with an overall sensitivity of 84.6%, and MicroExonator could detect microexons of all sizes with a sensitivity of 88.8%. Moreover, our results indicate that the direct output of STAR and Hisat2 do not represent a reliable source of microexons, as they have low specificity. Using the default parameters results in false discovery rates (FDR) of 0.43 and 0.33, respectively. Olego had the highest specificity (FDR = 0.13) of the aligners, while VAST-TOOLS and MicroExonator achieved an FDR of 0.12 and 0.10, respectively. However, most of the MicroExonator’s false discovery events correspond to microexons 1–2 nt (which are not reported by VAST-TOOLS), and when only ≥ 3-nt microexons are considered, FDR drops to 0.02 (Additional file 1: Fig. S3).

The simulations also allow us to calculate the ground truth percent spliced in (PSI) values for the microexons, a quantity that represents how frequently a splice junction is incorporated in a transcript. Both MicroExonator and VAST-TOOLS exhibited significantly lower PSI estimation errors for microexons < 10 nt compared to stand-alone aligners (Fig. 2d). However, VAST-TOOLS was designed to discover and quantify microexons 3–15 nt (additional VAST-TOOLS modules are required to quantify longer microexons), while MicroExonator provides accurate PSI estimates for all microexon sizes. Even though MicroExonator’s error rates are slightly higher for microexons > 10 nt, they are still comparable to the results obtained by stand-alone aligners. Taken together, these results show that MicroExonator is more accurate for annotating and quantifying microexons from RNA-seq data compared to conventional RNA-seq aligners and previously developed pipelines for microexon discovery.

### Microexon inclusion changes dramatically throughout mouse embryonic development

**Fig. 3:**
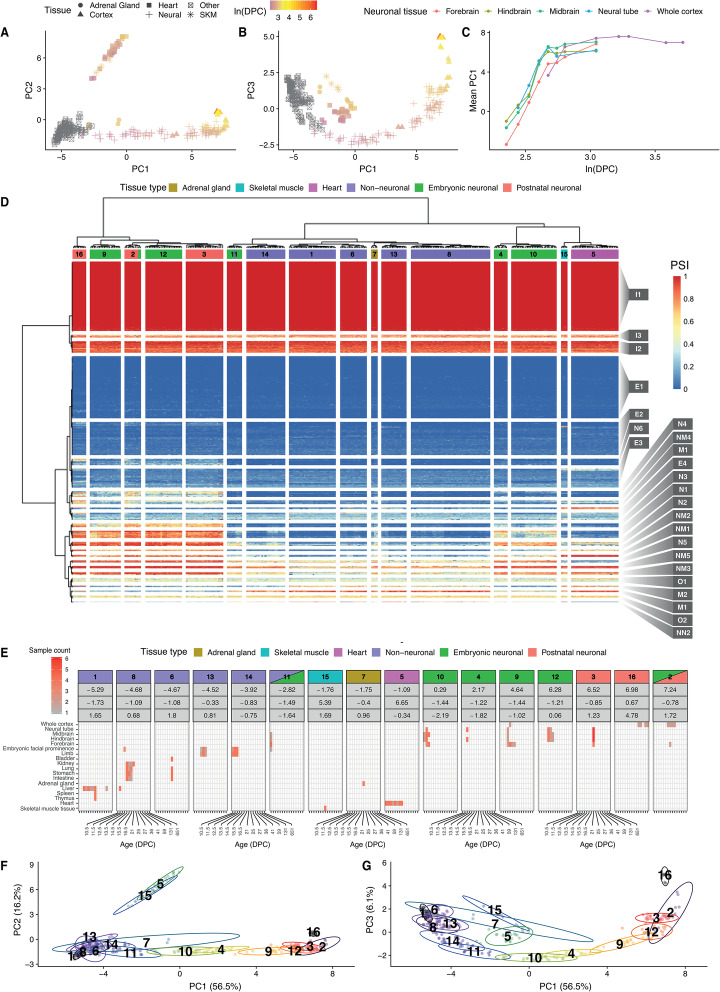
Microexon inclusion through mouse embryonic development. **a**, **b** Dimensionality reduction using probabilistic principal component analysis of microexon PSI values across mouse embryonic and postnatal samples reveals correlation with developmental time for PC1, while PC3 is correlated with the developmental time of the postnatal brain samples. Dot shapes denote different sample types, and their colour indicate their developmental stage, here expressed as log days postconception (DPC). **c** PC1 correspondence with embryonic developmental time. **d** Heatmap showing the microexon inclusion patterns across analysed RNA-seq samples, where rows correspond to microexons and columns to RNA-seq samples. Blue to red colour scale represents PSI values. RNA-seq samples were clustered in 16 different clusters. Microexons were clustered in 24 different clusters, and these were named according to their further classification as neuronal (N), muscular (M), neuromuscular (NM), weak-neuronal (WN) and non-neuronal (NN). **e** Tissue type and developmental stage composition from sample clusters containing neuronal samples or samples associated with high microexon inclusion. **f**, **g** Projection of the sample clusters across the estimated principal components

To investigate how microexon inclusion patterns change during mouse development, we analysed 271 RNA-seq datasets generated by the ENCODE Consortium [39, 40]. These RNA-seq data originate from 17 different tissues, (including the forebrain, hindbrain, midbrain, neural tube, adrenal gland, heart and skeletal muscle) across 7 different embryonic stages (ranging from E10.5 to E16.5), early postnatal (P0) and early adulthood (8 weeks). In addition, we analysed 18 RNA-seq experiments from mouse cortex across nine different time points: embryonic development (E.14.5 and E16.5), early postnatal (P4, P7, P17, P30) and older (4 months and 21 months) [32]. To generate the initial library of splice junctions, we provided MicroExonator with the GENCODE [24] and VastDB [11] transcript annotations. We detected and quantified 2984 microexons that are 3 nt or longer, and 928 of these were not annotated in neither GENCODE nor VastDB (Additional file 2: Table S1). As some microexons were detected in lowly expressed genes, we only retained microexons whose inclusion or exclusion was supported by > 5 reads in > 10% of the samples, and this resulted in 2599 microexons. To characterize the splicing patterns, we performed dimensionality reduction using probabilistic principal component analysis [41, 42], and we identified three components that together explain 78.9% of the total PSI variance across samples (Fig. 3a, b, Additional file 1: Fig. S4). The first principal component (PC1) accounts for 56.5% of the PSI variance and strongly correlates with the embryonic developmental stage of neuronal samples measured as days postconception (DPC) between E10.5 and E14.5, suggesting a strong coordination of microexon splicing during brain embryonic development (Fig. 3c, Additional file 1: Fig. S5). PC2 explains 16.2% of PSI variability and is mostly related to muscular-specific microexon inclusion patterns that were detected in the heart and skeletal muscle, suggesting muscle-specific microexon splicing patterns (Fig. 3a, Additional file 1: Fig. S4). Finally, PC3 explains 6.1% of PSI variability, and it is related to microexon alternative splicing changes in the whole cortex postnatal samples, suggesting that microexon neuronal splicing keeps changing after birth, but to a lesser extent than during embryonic development (Fig. 3b, Additional file 1: Fig. S4).

**Fig. 4:**
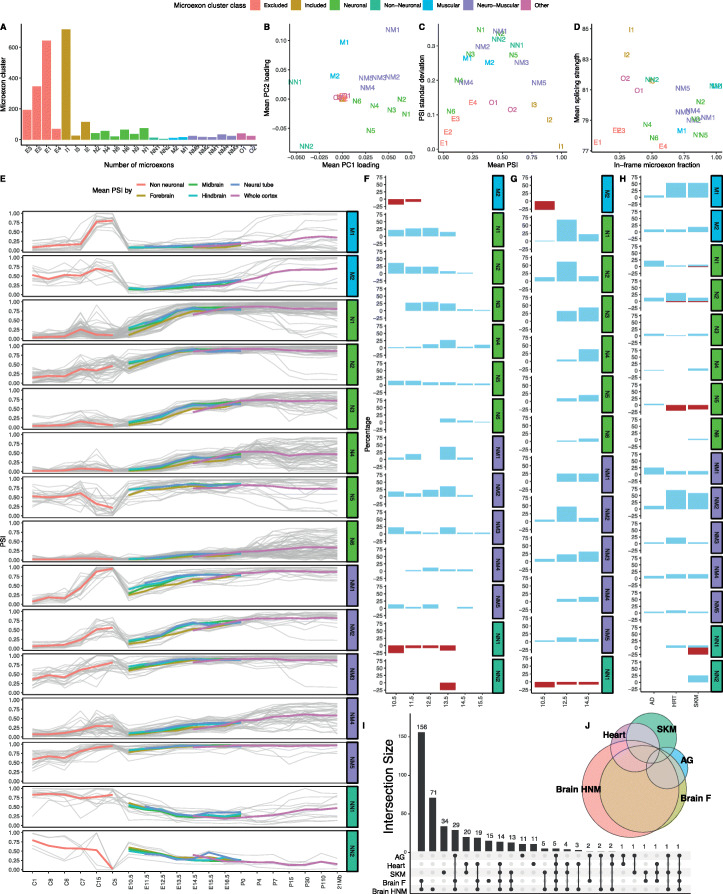
Inclusion properties of microexon clusters. **a** Number of microexons belonging to each cluster. **b** Mean loading factors across each cluster for PC1 and PC2. **c** Mean and standard deviation of PSI values across microexon clusters. **d** Mean U2 scores and in-frame fraction across microexon clusters. **e** Mean PSI values across neuronal and neuromuscular microexons. Each grey line represents the mean PSI values for a microexon across all samples from a tissue cluster or neuronal developmental stage (*x*-axis). Colour lines represent the mean PSI values across all clusters and stages. **f**–**h** Alternative microexons detected between non-neuronal tissue samples and midbrain, hindbrain and neural tube (**f**); forebrain (**g**); and adrenal gland (AG), heart (HRT) and skeletal muscle (SKM) (**h**). Microexon splicing changes are the percentage of microexons corresponding to each microexon cluster, where microexon inclusion fractions are represented with blue bars and exclusion events with red bars. **i** Intersection between microexon sets that were differentially included across sample groups. The vertical bars show the number of microexons corresponding to combinations indicated by the connected dots below. **j** Area-proportional Euler diagram representing the most abundant intersections between differentially included microexon sets

To further investigate tissue-specific microexon changes throughout the development, we performed bi-clustering of microexon PSI values from the different embryonic samples, and we obtained 24 microexon and 16 sample clusters (Fig. 3d). Each of the sample clusters represents a combination of well-defined subsets of tissues and embryonic states (Fig. 3e). For example, samples corresponding to the brain, heart, skeletal muscles (SKM) and adrenal gland (AG) form separate groups, with the only exception being E10.5 brain samples which clustered together with embryonic facial prominence limb from E10.5 to E12.0. Consistent with the dimensionality reduction analysis, samples from the brain cluster preferentially by developmental time rather than by neuronal tissue, suggesting that microexon alternative splicing changes are greater between developmental stages than between brain regions (Fig. 3e–g). As PC1 corresponds to changes of microexon inclusion during neuronal development and PC2 to muscle tissues, we used the mean loading factor values of each microexon cluster from PC1 and PC2 to classify 17 microexon clusters as neuronal (N), muscular (M), neuromuscular (NM), weak-neuronal (WN) and non-neuronal (NN) (Fig. 4a, b). Additionally, we found 10 microexon clusters that did not have strong tissue-specific patterns, but were instead either constitutively included (I) or excluded (E) (Figs. 3d and 4c).

Studies of standard alternative exons have shown that they typically have weaker splice signals than constitutive ones and that they are less likely to disrupt the reading frame [43]. Thus, we measured the splice site strengths as defined by the average U2 score of microexon flanking splice sites and the fraction of microexons that preserve the reading frame for each cluster (Fig. 4d). As expected, the included clusters exhibit the strongest splicing signals, while the excluded clusters have the weakest splice sites, suggesting that constitutive inclusion of microexons relies on strong splicing signals. Moreover, the excluded clusters have a lower fraction of in-frame events, implying that they are likely to be more disruptive to gene function. Interestingly, neuronal, muscular and some neuromuscular clusters have almost as weak splice sites as the excluded clusters, but the total in-frame fraction of these clusters is 0.74. This is considerably higher than the in-frame fractions for longer cassette exons (overall 0.43 and developmentally regulated 0.68) [32]. On the other hand, non-neuronal clusters have high U2 scores and also the highest in-frame microexon fraction. The in-frame fraction of each microexon cluster is strongly correlated with the conservation of the coding sequence (Pearson correlation = 0.88, *p* value < 1e−7, Additional file 1: Fig. S6, which implies that microexon clusters with higher conservation tend to preserve the protein frame.

We found a pattern of gradually increased microexon inclusion in the neuronal and neuromuscular categories during mouse brain development in neuronal tissues (Fig. 4e, Additional file 1: Fig. S7). By contrast, non-neuronal microexons exhibited the opposite trend. In addition, since neuronal and neuromuscular microexons have higher loading factors on PC1, they are likely to have the most variation across mouse embryonic development (Additional file 1: Fig. S8). To quantitatively assess alternative splicing across mouse brain development, we integrated Whippet [44] as part of an optional downstream MicroExonator module. We analysed 221 ENCODE RNA-seq experiments, using 85 non-neuronal samples from the three clusters (C1, C6 and C8) with the lowest PC1 loadings as negative controls. We systematically compared alternative splicing patterns detected in the brain, SKM, heart and AG against other non-neuronal tissues. To find microexon splicing changes associated with specific neuronal developmental stages, we pooled by embryonic stage RNA-seq samples from the midbrain, hindbrain and neural tube (MHN) between E10.5 and E16.5, and we used Whippet-delta to assess alternative splicing changes using MicroExonator and Whippet PSI values (Additional file 3: Table S2). We observed high correlations between the PSI values obtained from MicroExonator and Whippet, with the exception of microexons derived from 3′ or 5′ alternative splice sites (Additional file 1: Fig. S9). In total, we found 426 microexons that were consistently detected as differentially included (delta PSI > 0.1 and probability > 0.9 using MicroExonator and Whippet PSI values) in at least one of the comparisons between MHN and control groups. Interestingly, 326 of these microexon changes are maintained for all subsequent stages once they have been observed. The distribution of the developmental stages when these sustained microexon changes started to be detected differed. While some microexon clusters showed early changes (N1 and N2), other clusters started to be differentially included later on (N3, NM1 and NM2) (Fig. 4f). As the forebrain tends to show delayed microexon inclusion compared to the midbrain, hindbrain and neural tube (Figs. 3c and 4e), we pooled forebrain samples between E10.5 and postnatal (P0) and compared the samples grouped by developmental stage with the non-neuronal control sample group. We found 407 microexons that were differentially included during at least one forebrain developmental stage, with 257 sustained through all later developmental stages (Fig. 4g). In total, we found 332 differentially included microexon events that were sustained through all later developmental stages of MHN or forebrain. While all the observed microexon changes across neuronal and neuromuscular clusters correspond to inclusion events, microexons from the non-neuronal cluster (NN1) only correspond to exclusion (Fig. 4f, g).

In agreement with previous studies [11, 22] we also found strong inclusion patterns associated with heart and SKM. In addition, we found microexon inclusion patterns associated with AG samples (Fig. 3a, b, d, e). Compared with the set of non-neuronal control samples, we found 83, 106 and 58 microexons to be differentially included in the heart, SKM and AG, respectively (Fig. 4h). Most neuronal and neuromuscular microexon clusters show distinct microexon inclusion patterns for these tissues compared to controls, whereas amongst non-neuronal clusters, differentially included events were restricted to the heart and SKM (Fig. 4h).

The set of microexons that were differentially included across the different tissue groups (brain-MHN, forebrain, heart, SKM and AG) overlaps. Closer inspection reveals high concordance between the set of microexons associated with sustained changes in inclusion across MHN and forebrain samples. Surprisingly, we found a significant overlap of alternatively included microexons that have concordant patterns in AG and neuronal samples (hypergeometric test *p* value < 1e−30). Nearly all of the AG microexons are also found in neuronal samples, but in AG, we observed lower PSI values (Fig. 4i, j, Additional file 1: Fig. S10). We hypothesize that the mixture between neuronal and non-neuronal isoforms found in AG is due to the chromaffin cells in the adrenal medulla which are derived from the neural crest and share fundamental properties with neurons [45, 46].

### Microexon alternative splicing is coordinated throughout embryonic development

**Fig. 5:**
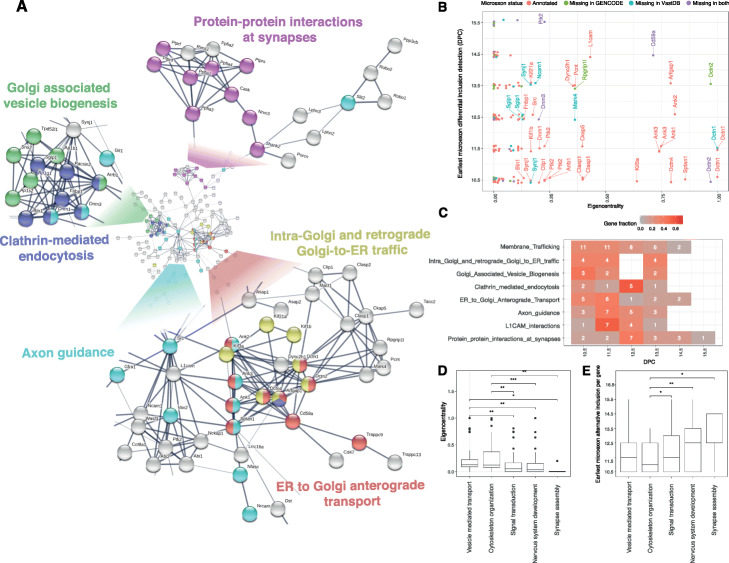
Microexon protein-protein interaction network. **a** PPI network using as input genes that have microexons that are differentially included across mouse embryonic brain development. Edges represent protein-protein interactions that are supported by either experimental evidence or databases. Edge width represents the confidence of the interaction based on a combined score calculated by STRING. Colours represent different Reactome pathways that were enriched on the network. **b** Eigencentrality calculated for each gene node in relation to the developmental stage at which each microexon was included. Labels are provided for the protein nodes belonging to the upper quartile eigencentrality, and their colours indicate their corresponding annotation status. **c** Effect of microexon alternative splicing for different Reactome pathways. Counts indicate the number of microexons that start to be differentially included at each developmental stage. **d**, **e** Eigencentrality and earliest developmental stage at which each gene is affected by differential microexon inclusion show differences across some of the GO categories that were significantly enriched after gene background correction. Statistical differences were assessed by one-sided Wilcoxon test while correcting for multiple comparisons. Significant *p* values are denoted by *> 0.05, **> 0.01 and ***> 0.001

Based on in vitro studies of neuronal differentiation, it has been proposed that microexons are an integral part of a highly conserved alternative splicing network [11]. Our analysis of mouse embryonic data (Fig. 4e) shows that most microexons remain included once their splicing status has changed. To explore the possible functional consequences of these splicing changes, we analysed the interactions between the proteins which contain microexons by constructing tissue-specific protein-protein interaction (PPI) networks for the brain, heart, SKM and AG using STRING [47]. For all four PPI networks, the degree of connectivity was significantly higher than expected by chance (*p* value < 1e−16) (Fig. 5a, Additional file 1: Fig. S11-S12). On average, there were 2.3-fold more connections than expected by chance, with the brain having the largest number of connections (Additional file 1: Table S3). Next, we considered the Gene Ontology (GO) terms and pathways associated with the PPI networks [48]. The Reactome pathways that showed a significant enrichment, include parts of molecular complexes that are involved in the membrane trafficking pathways, e.g. “ER to Golgi anterograde transport”, “clathrin-mediated endocytosis”, “Golgi-associated vesicle biogenesis”, “intra-Golgi and retrograde Golgi-to-ER traffic” and “lysosome vesicle biogenesis”. We also found a distinct cluster that is annotated as part of “protein-protein interactions at synapses”. This group includes presynaptic proteins, e.g. liprins (Ppfia1, Ppfia2 and Ppfia4), protein tyrosine phosphatase receptors (Ptprf, Ptprd and Ptprs) and neurexins (Nrxn3), which are involved in *trans*-synaptic interactions with multiple postsynaptic proteins, having a key role in synaptic adhesion and synapse organization. The interactions of these proteins have been shown to be highly regulated by alternative splicing [49], and our results reveal that many of these events occur towards the end of embryonic development.

In agreement with previous reports that have highlighted the importance of microexons for axonal and neurite outgrowth [14, 50], we detected 17 alternative neuronal microexons that affects 15 proteins in the PPI network that are annotated as part of the “axon guidance” Reactome pathway. These proteins are found in the centre of the network, and they are connected with the domains involved with membrane trafficking and *trans*-synaptic protein-protein interactions (Fig. 5a). For two of the proteins associated with this pathway, the non-receptor tyrosine kinase protein Src and L1 cell adhesion molecule (L1cam), microexon inclusion is known to play a key role in neuritogenesis [51, 52], but the importance of microexons in other proteins involved in this pathway remains poorly characterized.

To characterize the topology of the PPI network, we calculated the eigencentrality for each protein. Amongst the nodes with centrality scores from the upper quartile, we identified two microexons (in Dctn2 and Rpgrip1l) that were differentially included at E13.5, and they were not annotated in GENECODE, but only in VastDB (Fig. 5b). Conversely, we found seven alternative microexons (in Dctn1, Mark4, Ncam1, Synj1 and Sgip1) that were not annotated in VastDB, but only in GENECODE. Interestingly, within the upper quartile of eigencentrality values, we also detected four alternative microexons (in Dctn2, Cd59a, Ptk2 and Dnm3) that were not annotated neither in GENCODE nor in VastDB. This result demonstrates that it is important to perform microexon exon discovery and to integrate different sources of transcript annotation, as many of the central nodes in the PPI network would have been missed otherwise. At early developmental stages (E10.5–E11.5), we found several microexon alternative splicing events in genes associated with “membrane trafficking” pathways concentrated. A subset, “clathrin-mediated endocytosis”, is associated with microexon changes in the later stages, as most events became significant only after E12.5 (Fig. 5c). Similarly, “axon guidance” microexon changes mostly occur at E11.5, in particular, the microexon alternative splicing events for proteins that interact with L1cam. L1cam and 7 out of 10 of its interactors are amongst the 25% of nodes with the highest eigencentrality, and Src has the highest harmonic centrality and betweenness. These results suggest that microexon regulation across mouse embryonic development may impact Src/L1cam-associated pathways. The inclusion of Src microexon has been reported to enable L1-CAM-dependent neurite elongation [52]; however, the global effect of microexons on Src/L1cam-interacting proteins is currently unknown.

Finally, an investigation of genes corresponding to some of the most relevant GO terms revealed that groups of genes related with vesicle-mediated transport and cytoskeleton organization hold more central positions in the PPI network than genes associated with signal transduction, nervous system development and synapse assembly (Kruskal-Wallis rank sum test, *p* values < 0.05, Fig. 5d). Similar to the microexons found in genes associated with cytoskeleton organization, they are also included earlier in development than in microexon found in genes related with signal transduction, nervous system development and synapse assembly (Kruskal-Wallis rank sum, *p* values < 0.05, Fig. 5e).

### MicroExonator enables the identification of novel neuronal microexons

**Fig. 6:**
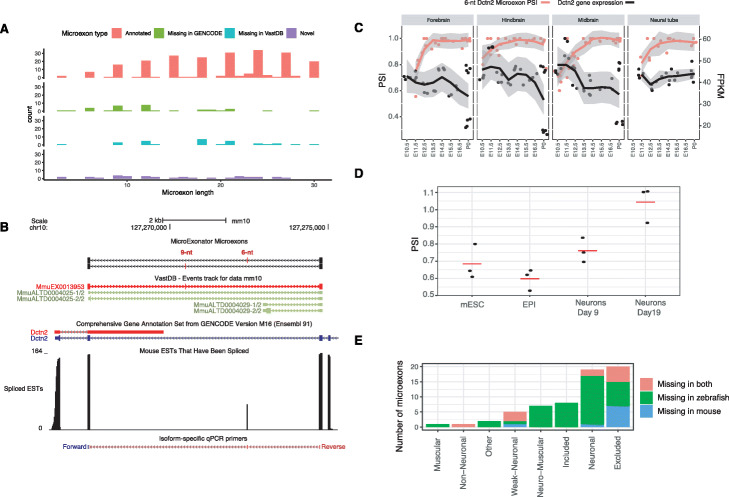
Discovery of novel microexons in mouse and zebrafish. **a** Histogram showing the size distribution of microexons that were found to be differentially included across mouse embryonic development. **b** Alternative Dctn2 microexons that are inconsistently annotated in mouse GENCODE and VastDB annotations. **c** Novel 6-nt Dctn2 microexon shows a progressive inclusion through mouse embryonic development. **d** PSI values calculated from normalized RT-PCR measurements show a gradual inclusion of the 6-nt Dctn2 microexon though in vitro neuronal mESC to neuron differentiation. **e** Number of conserved microexons between mouse and zebrafish that are missing from their transcript annotation

Of the 332 microexons that were differentially included across brain development, 98 were inconsistently annotated as compared to GENCODE and VastDB. Of these 98 neuronal microexons, we found 35 that are only annotated in GENCODE, and 30 neuronal microexons that are not annotated in GENCODE, but are present in VastDB. Despite the fact that the mouse genome is comprehensively annotated, we found 33 neuronal microexons that are not annotated neither in GENCODE nor in VastDB. The high sensitivity and specificity demonstrated in simulations imply that the false discovery rate is 0.0053 for microexons ≥ 6nt (Fig. 2, Additional file 1: Fig. S3). Thus, we expect that all 31 microexons ≥ 6 nt are true positives, and our conclusion is further supported by the fact that the lengths follow a similar periodicity pattern to annotated microexons (Fig. 6a).

To validate one of the novel microexons, we focused on the Dctn2 gene (eigencentrality of 0.76), where we detected two adjacent differentially included microexons of length 9 and 6 nt (Fig. 6b). Neither of these microexons are annotated in GENCODE, but the 9-nt microexon is annotated in VastDB (MmuEX0013953). Interestingly, the downstream 6-nt microexon that was discovered by MicroExonator is validated by spliced ESTs [53]. We detected differential inclusion of the 6-nt Dctn2 microexon from E10.5 in MHN samples, whereas in the forebrain, it is differentially included from E12.5 (Fig. 6c).

We performed qRT-PCR experiments to assess the inclusion of the Dctn2 6-nt microexon during mESC to neuron differentiation. We used one set of primers to amplify Dctn2 isoforms with 6-nt microexon inclusion and another set to amplify total Dctn2 isoforms. Next, we calculated the ratio of 6-nt inclusion across mESC, epiblast stem cells and differentiated neurons at two different stages (Fig. 6d). The inclusion ratios from the qRT-PCR measurements indicate that the Dctn2 6-nt microexon is included through in vitro differentiation of mESC to neuron, consistent with our findings during embryonic development for this microexon. These results show that the alternative splicing quantification provided by MicroExonator can identify novel microexons, even for model organisms that are well annotated.

### Identification of microexons in zebrafish brain

To demonstrate how MicroExonator can be applied to species with less complete annotation, we analysed 23 RNA-seq samples from zebrafish brain [54]. We found 1882 microexons (Additional file 4: Table S4), of which 23.8% are not found in the ENSEMBL gene annotation. We used the liftover tools [55] to assess whether some of these microexons are evolutionary conserved microexons in mice, and we successfully mapped 401 zebrafish microexons. Of these, 85% mapped directly to a previously identified mouse microexon, and most of the remaining 15% mapped to longer exons. Mapping the microexons in the other direction, 617 out of 2938 that were identified from the mouse development data mapped to the zebrafish genome and 49.7% of those in return mapped to a zebrafish microexon. By integrating these results, we obtained a total of 402 microexon pairs that are found in both zebrafish and mice (Additional file 5: Table S5). Since 90.3% of the pairs had an identical length in both species, they are highly likely to correspond to the evolutionary conserved microexons.

To compare the microexon annotation between mouse and zebrafish, we asked how many of the 402 conserved microexons that are missing in mouse or zebrafish gene transcript annotation. While only 6.9% of these exons are missing from the mouse transcript annotation provided by GENCODE, 16.1% are missing from the ENSEMBL zebrafish transcript annotation. Moreover, the largest fraction of conserved microexons that are missing in zebrafish transcript annotation corresponds to neuronal microexons (Fig. 6e).

### Cell type-specific microexon inclusion in mouse visual cortex

Our analysis of neuronal development suggested that the main difference in microexon inclusion is between time points rather than tissues. However, since these data do not reflect the diversity of cell types within neuronal tissues, we hypothesized that microexon inclusion patterns may vary amongst different neuronal subtypes. To study the cell type-specific patterns of microexon inclusion, we analysed the SMART-seq2 scRNA-seq data from the visual cortex of adult male mice [31]. The sample contains 1657 cells which were assigned into six cell type classes that were further subdivided into 49 distinct clusters.

We focused on the GABA-ergic and the glutamatergic clusters of neurons which contain 739 and 764 cells, respectively. We first ran the microexon discovery module with an expanded annotation, which included the microexons discovered from our previous analyses. This yielded 2344 microexons that were included in at least one cell. Next, we used Whippet to quantify the PSI of the microexons detected by MicroExonator for each cell. Due to the sparse coverage, the single-cell analysis is sensitive to errors. Thus, for each neuronal type, we also pooled 15 randomly selected neurons into pseudo-bulk groups that were quantified by Whippet. To avoid false positives due to the pooling, we ran the analysis 50 times and we integrated the results to ensure the robustness of the reported alternative splicing changes between the two neuronal subtypes. From a total of 195,441 splicing nodes tested, we detected 208 that were consistently differentially included between GABA-ergic and glutamatergic neurons (Additional file 6: Table S6). Amongst these nodes, 2265 correspond to microexon splicing events, and 29 were differentially included between these neuronal classes (28 core exon and 1 alternative acceptor node). These results show that alternative splicing events between GABA-ergic and glutamatergic neurons are strongly enriched for microexon splicing events (hypergeometric test *p* value < 10^−19^ when the total amount of nodes or just the core exon nodes are considered).

**Fig. 7:**
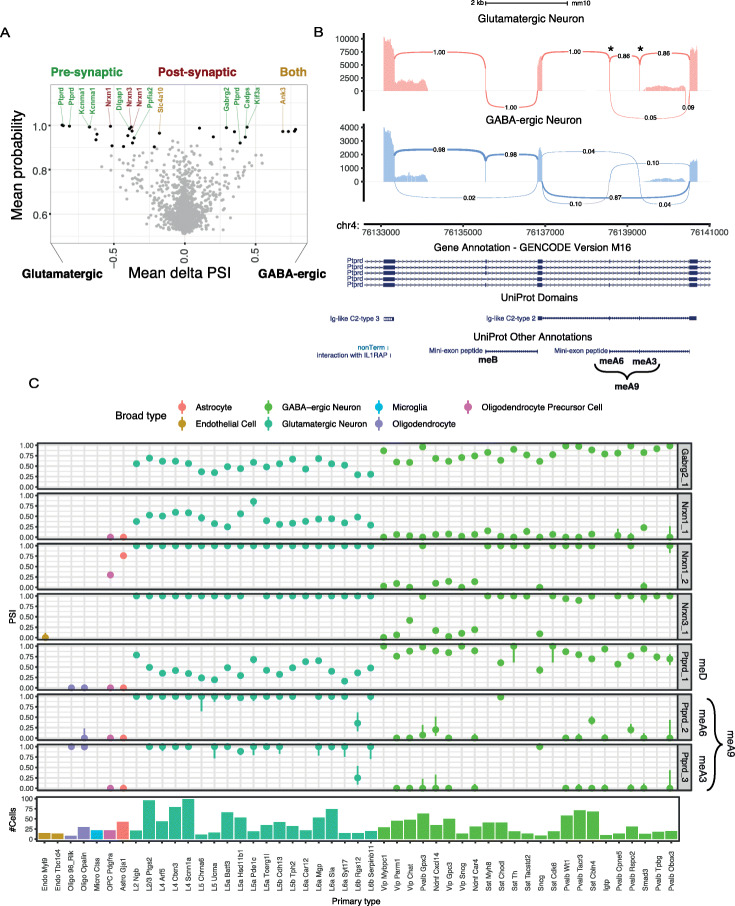
Differential alternative splicing analysis of microexons between glutamatergic and GABA-ergic neurons using scRNA-seq data. **a** Volcano plot showing alternatively included microexons between glutamatergic and GABA-ergic neurons. Differentially included microexons are highlighted in black. Synaptic proteins are labelled with different colours depending on their sub-synaptic localization. **b** Sashimi plot showing Ptprd microexons that determine the inclusion of meA/B mini-exon peptides. **c** Microexon inclusion patterns across all core clusters at proteins involved in *trans*-synaptic interactions. Ptprd microexons involved in the inclusion of mini-exon peptides are labelled on the side (meA and meD)

Amongst the genes that contain differentially included microexons between GABA-ergic and glutamatergic neurons is a group of eleven genes that encode for proteins that localize at synaptic compartments. We found seven presynaptic proteins, two postsynaptic proteins and two proteins that have been observed at both locations (Fig. 7a). For example, the type IIa RPTP subfamily of proteins undergo tissue-specific alternative splicing that determines the inclusion of four short peptide inserts, known as mini-exon peptides (meA-meD) [49, 56, 57]. While meB comprises four residues (ELRE) and is encoded by a single microexon, meA has three possible variants that can form as a result of the combinatorial inclusion of two microexons: meA3 (ESI), meA6 (GGTPIR) and meA9 (ESIGGTPIR) [58]. Ptprd (also known as PTPδ) is a member of the RPTP subfamily, and our analysis shows a consistent inclusion of Ptprd meB in both GABA-ergic and glutamatergic neurons. However, we detected cell type- specific rearrangement of meA microexons which promotes the inclusion of meA9 in glutamatergic neurons, while in GABA-ergic neurons, meA variants are mostly excluded (Fig. 7b). Alternative splicing of meA/B microexons is key to determining the selective *trans*-synaptic binding of Ptprd to postsynaptic proteins, which is a major determinant of the synaptic organization [49]. In addition, we found that the Ptprd microexon that determines the inclusion of meD is alternatively included, as well as microexons in genes that are involved in synaptic cell adhesion, e.g. Gabrg2, Nrxn1 and Nrxn3 [49, 59]. The microexon inclusion in these genes is variable across the core clusters, sometimes showing stark differences between GABA-ergic and glutamatergic neuron subtypes (Fig. 7c). These results suggest that microexon inclusion is not only coordinated at the tissue type level, but it is also finely tuned across neuronal cell types, and these differences may be of importance for determining neuronal identity and synapse assembly.

## Discussion

We have presented MicroExonator, a complete bioinformatic workflow for reproducible discovery and quantification of microexons. MicroExonator is designed to handle large volumes of data, and it will automatically download datasets and schedule jobs on a computer cluster. Currently, it is the only publicly available method that allows these types of investigations. MicroExonator’s discovery module is based on the detection of inserted sequences between annotated splice sites which enables the identification of very short microexons that cannot be reliably detected by spliced RNA-seq aligners (Fig. 2c). Thus, MicroExonator will greatly facilitate the study of microexons. MicroExonator is straightforward to incorporate into an existing RNA-seq analysis workflow. Importantly, MicroExonator can be used to directly study the microexon conservation across species, thereby making it possible to understand if inclusion patterns are as well conserved as the nucleotide sequences. Furthermore, MicroExonator makes it possible to study RNA-seq data from large cohorts to investigate if there are microexons that differ amongst individuals.

As proof of principle, we used MicroExonator to analyse RNA-seq data from 301 RNA samples from mice at embryonic and adult stages and 1679 single cells. We have expanded the catalogue of murine microexons by identifying 928 previously uncharacterized loci. In agreement with previous analyses, we identified microexons that were differentially included in the brain, heart and SKM [11, 22], but we also detected 58 microexons that are differentially included in the adrenal gland. Taken together, we have presented the most comprehensive catalogue of microexons available to date, and it allowed us to uncover several distinct inclusion patterns in both developing and adult mice.

### Microexon coordination across neuronal development

Our quantitative analysis revealed that the proteins containing microexons form a highly connected network during mouse neuronal development. Moreover, analysis of the topology of the network suggests that the microexons for the most central nodes are included early in development. It is not yet fully understood how this coordination is achieved, but it has been shown that microexon inclusion relies on upstream intronic splicing enhancers which promote neuron-specific microexon inclusion [60]. However, we also identified a large group of microexons that are constitutively included across murine tissues, suggesting that their inclusion cannot be dependent on tissue-specific factors alone. Instead, our analysis points to a more straightforward explanation as the constitutive microexons have stronger splicing signals than neuronal microexons. Further analysis of neuronal microexon *cis*-regulatory elements is required to understand how inclusion events are coordinated and why there is a small number of microexon that is progressively excluded through brain development.

The predominant mechanism for regulating alternative splicing events during neuronal development is through RNA-binding proteins [8]. In the case of microexons, SRRM4 and RBFOX1 have a critical role in coordinating microexon inclusion through brain development, and changes in the expression of these splicing factors have been linked to misregulation of alternative splicing events in individuals with autism spectrum disorder (ASD) [11, 22, 61]. In fact, alternative splicing changes associated with ASD are enriched for microexons, and they are recapitulated in mutant mice haploinsufficient for SRRM4, which also display multiple autistic features [14]. Moreover, a recent genome-wide CRISPR-Cas9 screen has identified two additional factors, SRSF11 and RNPS1, that contribute to SRRM4-dependent microexon regulation, and these genes have also been implicated in ASD and other neurological disorders [60]. Another example of a protein where imbalances of microexon inclusion have been associated with an elevated risk of ASD is cytoplasmic polyadenylation element-binding protein 4 (CPEB4) [62]. We found differential inclusion of CPEB4 microexon during mouse embryonic brain development, and we also found microexon changes in other protein factors that are involved in mRNA polyadenylation, such as CPEB2, CPEB3 and FIP1L1. However, the role of these microexons in neuronal function and neuropsychiatric diseases remains unexplored.

The high degree of conservation of microexons strongly suggests that they are functionally important, but for the most part, we lack a detailed, mechanistic understanding. A notable exception is Src where microexon inclusion leads to the production of n-Src, a well-characterized neuronal splice variant. The Src microexon encodes for a positively charged residue located at an SH3 domain that has been shown to regulate Src kinase activity and specificity [63]. From the STRING analysis, we found evidence for Src-dependent phosphorylation of Git1, Ctnnd1 and Ptk2 [64–66], though the impact of neuronal microexon alternative splicing for these phosphorylation events remains unknown. Moreover, recent studies show that n-Src microexon inclusion is required for normal primary neurogenesis and L1cam-dependent neurite elongation [52, 67], implying a strong phenotype. Another central node in the PPI network that is known to undergo microexon alternative splicing changes that are important for axon growth is L1cam, a founding member of the L1cam protein family. Across the L1cam protein family, a sorting signal is included due to 12-nucleotide alternative microexons. In the case of L1cam, the 12-nucleotide microexon mediates its clathrin-mediated endocytosis by interacting with adaptor protein complex 2 (AP-2) [68]. Our analysis shows that the AP-2 mu subunit (Ap2m1) is also affected by microexon inclusion through mouse brain development.

### Cell type-specific microexon alternative splicing across the mouse visual cortex

Single-cell RNA-seq data is providing an unprecedented opportunity to survey cell-specific expression profiles. However, with a few notable exceptions [69–72], most scRNA-seq analyses have focused on analysis at gene rather than the transcript level. Here, we applied MicroExonator to GABA-ergic and glutamatergic cells from the visual cortex, and to increase the power, we developed a downstream SnakeMake workflow, snakepool. As many splicing events are undetected in single-cell data due to poor coverage, a pooling strategy is necessary to increase the power to identify significant differential inclusion events.

We identified 29 microexons that were differentially included between GABA-ergic and glutamatergic neurons and 11 synaptic proteins that are affected by 15 of these cell type-specific microexons. Amongst these, we found three alternative microexons on Ptprd, which control the inclusion of meA and meD mini-exon peptides. While meA is known to have a key role in modulating *trans*-synaptic interactions and having a direct impact on synapse formation [58, 73], the functional repercussions of meD inclusion remain unexplored. In addition, we also show that microexons found in Ptprd and other proteins involved in *trans*-synaptic protein interactions can have distinctive alternative inclusion profiles across GABA-ergic and glutamatergic subtypes (Fig. 7c). Importantly, this result demonstrates that even though bulk RNA samples from different brain regions are largely similar, there are differences between both neuronal and non-neuronal populations. The differential inclusion of microexons could have profound effects on neuronal identity, synapse formation and disease. For example, the differential microexon inclusion event that we identified in GABAa receptor subunit γ (GABRG2) can have a direct impact on GABA-ergic neurons as this microexon introduces a phosphorylation site that regulates GABA-activated current. Misregulation of this alternative splicing event has been associated with schizophrenia in human patients [18]. However, additional analyses of alternative microexon patterns across neuronal cell types will be required to fully understand their contribution to neuronal heterogeneity and function.

## Methods

### Annotation-guided microexon discovery using RNA-seq data

MicroExonator was implemented over the SnakeMake workflow engine [34], to facilitate reproducible processing of large numbers of RNA-seq samples. In the initial discovery module, MicroExonator uses annotated splice junctions supplied by the user (a gene model annotation file can be provided in GTF or BED format) to find novel microexons. RNA-seq reads are first mapped to a library of reference splice junction tags using BWA-MEM [28] with a configuration that enhances deletion detection (bwa mem -O 6,2 -L 25). The library of splice junction tags consists of annotated splice junctions between exons ≥ 30 nt and spanning introns ≥ 80 nt. For each splice junction, a reference sequence tag is generated by taking 100 nt upstream and downstream from the corresponding transcript sequence. Splice junction alignments are processed to extract read insertions with anchors ≥ 8 nt that map to exon-exon junction coordinates. Inserted sequences are then re-aligned inside the corresponding intronic sequence, but only matches flanked by canonical splice site dinucleotides (GT-AG) are retained (Fig. 1a). The obtained reads are re-mapped to the reference genome using hisat2 [74]. A preliminary list of microexon candidates is generated based on reads whose insertions are aligned to the intronic spaces with no mismatches and that could not be fully mapped to the reference genome (soft clipping alignments are ignored).

### Quantification of microexon inclusion

In a subsequent quantification module, novel microexon candidates are integrated into the gene annotation to generate a second library of splice junctions tags, where putative novel loci from the discovery phase and annotated microexons are integrated at the middle of the tag sequences (Fig. 1b). Reads are aligned again to this expanded library of splice junction tags using Bowtie [75], which performs a fast ungapped alignment allowing for 2 mismatches (bowtie -v 2 -S). Reads that map to the splice junction tags are also mapped to the reference genome using Bowtie, also allowing two mismatches. Reads that could only fully map to a single splice junction tag but no other location count towards novel or annotated microexons.

### Filtering of spurious intronic matches

MicroExonator uses a series of filters to distinguish real splicing events that may result in a novel microexon of length *L* from spurious matches with intronic sequences. Since we only allow for intronic matches that are flanked by canonical dinucleotides (4 nt), we search for a matching sequence of length *L* + 4 in the intron.

For a random sequence of length *L*, where all four nucleotides have the same frequency, the probability of at least one spurious match inside an intron with flanking GT-AG dinucleotides, *P*_*s*_, can be calculated as *P*_*s*_ = 1 − (1 − 1/4^*L* + 4^)^*K*^, where *K* is the number of *k*-mers of length *L* + 4 that are found in an intron of length *N*, with *K* = *N* − *L* − 4. Since microexons shorter than 3 nt cannot be identified with high specificity, they are reported as a separate list without further filtering.

Microexons that are 3 nt or longer are filtered further by evaluating the splice site signal by measuring the match to the canonical splicing motif as defined by the U2-type intron position frequency matrices [29]. We normalize the score to range from 1 to 100, and we call this quantity U2 score. We then fit the distribution of U2 scores using a two-component Gaussian mixture model (Fig. 1d), and from this, we calculate a score, *M*_*s*_, for each putative microexon as *M*_*s*_ = 1 − (1 − *P*_*s*_*P*_U2_)/*n*, where *P*_U2_ is the probability that the observed U2 score came from the Gaussian with the higher mean and *n* is the number of matches for a given intron. Finally, MicroExonator calculates an adaptive threshold, M^*^, to determine the minimal *M*_*s*_ score required. Let *R*^*t*^ denote the number of detected microexons that have *M*_*s*_ > *t*. A linear model is used to fit *R*^*t*^ as a function of their length, with *t* ranging between 0 and 1. MicroExonator recommends M^*^ as the score corresponding to t^*^, the value which results in the minimal residual standard deviation sum. This threshold is used to generate a high confidence list of microexons, but all detected microexon are reported across different output files. By default, MicroExonator uses M^*^ to filter out microexons with low scores, but the threshold can be set manually by the user. If conservation data (e.g. Phylop/Phastcon) is provided, then microexons with *M*_*s*_ < M^*^ that exceed a user-defined conservation threshold (default value = 2) are also included in a high confidence list of microexons and flagged as “rescued”.

### RNA-seq simulation

We used Polyester [36] to simulate RNA-seq reads from modified mouse GENCODE gene models (V11). To generate true positive microexons, we inserted a set of 4930 randomly selected sequences with a length ranging from 1 to 29 nucleotides inside annotated introns longer than 80 nt. At the same time, we swapped the original intronic sequences of annotated microexons for splicing signals found at another randomly selected annotated exon. To simulate spurious microexon matches (false-positive microexons), we randomly included 5180 insertions corresponding to intronic sequences at exon-exon junctions that were not flanked by canonical splicing sequences. The insertion rates and lengths were simulated using parameters extracted from real RNA-seq experiments from postnatal forebrain samples. Our simulations provide a realistic set of false-positive microexons that emulates real RNA-seq experiment condition as closely as possible. The microexon discovery module from VAST-TOOLS was made available by the authors upon request. To discover novel microexons with VAST-TOOLS, reads were pre-processed using “VAST-TOOLS align” to split each simulated 100-nt reads into 50-nt reads with 25 nt of overlap (using the arguments -sp mm10 --noIR --keep -c 15). The obtained reads were further processed using the run_mic_extraction.sh script to obtain a list of novel microexons (using the arguments -c 15 -maxL 29).

### Using MicroExonator to analyse publicly available RNA-seq datasets

MicroExonator can be configured to download and process any number of RNA-seq samples that can be found locally or deposited on public archives, such as Short Read Archive, European Nucleotide Archive or ENCODE. During the initial configuration steps, MicroExonator extracts annotated microexons and splice sites from one or more gene annotation databases (e.g. GENCODE) and optionally complements them with multiple specialized alternative splicing databases such as VastDB [23]. After configuration, SnakeMake enables coordination with cluster schedulers used on high-performance computing platforms or direct process management on a single computer. Thus, given a shortlist of configuration files, MicroExonator can be set to fully reproduce any previous analyses through a single command. Moreover, we provide additional SnakeMake workflows to integrate MicroExonator with downstream quantification steps and to optimize analyses of single-cell RNA-seq, which are often much noisier than bulk RNA-seq data.

### Microexon analyses across mouse development using bulk RNA-seq data

As a proof of principle, we applied MicroExonator to 283 RNA-seq datasets obtained from the ENCODE Project (Sloan et al. [30]), corresponding to embryonic and postnatal tissue samples coming from 17 different tissues. We used mm10 mouse genome assembly obtained from the UCSC Genome Browser database (Karolchik et al.), and as a source of annotated splice junctions, we used the union of GENCODE Release M16 (Harrow et al. [24]) and VastDB [23]. We quantified novel and annotated microexons through percent of spliced-in (PSI) values by using MicroExonator’s built-in scripts or by using Whippet [44]. Bi-clustering of samples and microexons was performed by applying Ward’s minimum variance criterion in R [76, 77] over a MicroExonator Euclidean distance matrix where the similarity of the samples was calculated from the PSI values (Additional file 8).

PSI values were also used to perform PPCA using the ppca function from the pcaMethods R library [78]. The obtained PPCA loading factors were used to classify microexon clusters. Assuming that PC1 and PC2 are related with variance observed at the brain and muscle, respectively, loading factors can be used to evaluate the tissue specificity of microxon inclusion. Thus, microexons that have loading factors > 0.03 for PC1 and PC2 were considered as neuromuscular (NM1–3). The ones that only have high loading factors for either PC1 or PC2 were considered as neuronal (N1–4) and muscular (M1–3), respectively. We found one microexon cluster with a significant negative loading factor over PC1 (< − 0.03), which we considered to be non-neuronal (NN1). We also found microexon clusters that have a consistent inclusion (I1–7) or exclusion (E1–5) pattern across all samples.

We quantified splicing nodes using Whippet’s quantification module (whippet-quant.jl) and we supplied MicroExonator output as input to the Whippet differential inclusion module (whippet-delta.jl). We used both MicroExonator and Whippet quantification to assess changes in microexon inclusion between different sample groups. A baseline was defined by the non-neuronal tissue clusters that had the lowest PC1 values (clusters C1, C6 and C6). Different neuronal sample groups were defined by developmental stages (E10.5, E11.5, E12.5, E13.5, E14.5, E15.5) and brain tissue type; samples corresponding to the midbrain, hindbrain and neural tube were pooled together (MHN sample group) whereas forebrain samples were evaluated independently. Moreover, additional non-neuronal groups were formed according to their tissue of origin, which correspond to the heart, SKM or adrenal gland. Each sample group was compared with the baseline groups. Across the different comparisons, we only considered as significant those microexons which have > 0.9 probability of being differentially included and ≥ 0.1 delta PSI values. To further avoid quantification errors, we only selected those microexons that were detected as differentially included using both MicroExonator and Whippet quantification. The sets of genes that have at least one microexon differentially included in the brain, SKM, heart or adrenal gland were analysed by building a protein-protein interaction network using STRING [47]. PPI network analyses were performed using STRING v.11.0 [47], through the main webserver (https://string-db.org/) taking as input the ENSEMBL ID of the set of genes which contains one or more microexons.

### Neuronal mouse dopamine neuron preparation and RT-PCR validations

Mouse embryonic stem cells (mESC) were differentiated into dopamine neurons as previously described [79]. Briefly, mESCs were first differentiated into epiblast stem cells (EPI) using fibronectin-coated plates and N2B27 basal media (composed of Neurobasal media, DMEM/F12, B27 and N2 supplements, l-glutamine and 2-mercaptoethanol) supplemented with FGF2 (10 mg/ml) and activin A (25 mg/ml). After three passages, EPI were differentiated into dopaminergic neurons using plates coated with poly-l-lysine (0.01%) and laminin (10 ng/ml) and N2B7 media supplemented with PD0325901 (1 mM) for 48 h (day 0 to day 2). Three days later (day 5), N2B27 media were supplemented with Shh agonist SAG (100 nM) and Fgf8 (100 ng/ml) for 4 days. The media were then changed to N2B27 media supplemented with BDNF (10 ng/ml), GDNF (10 ng/ml) and ascorbic acid (200 nM) from day 9 onwards. During neuronal differentiation, cells were passaged at day 3 and day 9. Cells were collected for qRT-PCR analysis at several stages: mESC, EPI, day 9 neurons and day 19 neurons. RNA extraction was performed using the RNeasy Mini Kit (Qiagen), and samples were analysed with a QuantStudio 5 PCR system (Thermo Fisher Scientific).

### Microexon identification in Zebrafish brain

RNA-seq experiments for Zebrafish brain tissues were obtained from (Park et al. [54]) using GEO accession code GSM2971317. Microexon detection and quantification were performed with MicroExonator using default parameters based on Ensembl gene predictions 95 and the danRer11 genome assembly. To compare mouse and zebrafish microexons, we performed a batch coordinate conversion using the liftover script from UCSC utilities [80].

### Single-cell analyses

To identify differentially included microexons across cell populations profiled using scRNA-seq, we have developed snakepool, a SnakeMake framework that works as a downstream module of MicroExonator. The sparse nature of scRNA-seq data makes it difficult to estimate PSI, and to get around this problem, we pool cells into pseudo-bulks (default = 50 pools of equal size). snakepool runs Whippet splicing node quantification (whippet-quant.jl) on the groups of cells, and the resulting quantification of pseudo-bulks is used to provide a probability of differential inclusion for each splicing node (whippet-delta.jl). To avoid false positives due to the pooling of cells, the pseudo-bulk quantification of splicing nodes and differential inclusion assessment is repeated *r* times (default *r* = 50). We fit a beta distribution to the *r* probabilities of differential inclusion for each splicing node. The beta distribution models the probability of including a splicing node. Let the cumulative distribution function be *P*_*s*_ and let *y* = argmin *P*_*s*_(*x*) > *t*, where *t* is a user-defined threshold (default *t* = 0.8). If *y* < 0.05, and the mean probability of differential inclusion is > 0.9, then a node is flagged as differentially included.

We applied snakepool with default parameters to assess the differential inclusion of microexons between GABA-ergic and glutamatergic neurons. Sashimi plots were generated by adapting ggsashimi [81] to display the total number of reads that is supported by each splice site (Supplemental Material). The total read count for each cell type was subsequently used to calculate splice site usage rates.

## Supplementary Information

**Additional file 1: Figure S1.** Number of reads assigned to microexon splice sites during the first and second splice junction alignment performed during discovery and quantification modules respectively. **Figure S2.** Filtering of putative novel and annotated microexons. **Figure S3.** Microexon false discovery rate across evaluated software. **Figure S4.** PCA analysis of 289 bulk RNA-seq samples. **Figure S5.** A) PCA plot where only the ENCODE samples are shown. Neuronal samples are color coded on a blue to red scale based on developmental time. B) Relationship between PC1 and mouse developmental stage (age) of ENCODE samples. **Figure S6.** Relationship between mean conservation score (PhyloP) and fraction of in-frame microexons for different microexon clusters and developmental stages. **Figure S7.** Average Microexon PSI for each microexon cluster across the different sample clusters are shown in red. Grey lines show the average PSI of individual microexons. **Figure S8.** Boxplots showing PC1-3 loading factors for the different microexon clusters. **Figure S9.** Volcano plots showing the distribution of delta PSI values of microexon splicing nodes and their corresponding probability of being differentially included across MHN samples coming from different developmental stages (E10.5-E16.5). **Figure S10.** Differences in PSI values between adrenal gland, brain MHN and forebrain tissues. **Figure S11.** String PPI network of genes that were detected to have differentially included microexons between the control group and neuronal samples. **Figure S12.** PPI network corresponding to the group of genes that were detected to have differentially included microexons between the control groups and A) Heart B) Skeletal muscle C) Adrenal gland. **Table S3.** PPI network summary statistics reported by STRING.

**Additional file 2: Table S1.** High confidence list of detected microexons. Here we report all of the high confidence microexons detected by MicroExonator from mouse bulk and scRNA-seq. The table also includes information about the downstream analyses. Columns PC1-3 summarize the PPCA results; In.10_percent_of_bulk indicates if the microexon was supported by >5 reads in >10% of bulk RNA-seq samples; MHN/F.diff columns indicate if they were found to be alternatively included in these sample groups at any of the time points that were compared with the control group. For the microexons that remained differentially included during brain development MHN/F.change_dir and MHN/F.diff_age indicate the direction of inclusion and the embryonic stage since they started to be detected as differentially included.

**Additional file 3: Table S2.** Condition groups assigned to each analysed bulk RNA-seq sample.

**Additional file 4: Table S4.** Microexons detected in zebrafish. This table shows the output from MicroExonator when applied to the zebrafish RNA-seq samples.

**Additional file 5: Table S5.** Overlapping microexons between mouse and zebrafish.

**Additional file 6: Table S6.** Differential inclusion of splicing nodes between GABA-ergic and glutamatergic neurons. The total set of splicing nodes analysed by snakepool. Negative DeltaPsi.mean values indicate higher inclusion in glutamatergic neurons and positive values indicate higher inclusion in GABA-ergic neurons. The column is.diff indicates significant differences between splicing node inclusion levels and microexon_ID provides the corresponding coordinates of microexon quantified as splicing nodes.

**Additional file 7.** Text file used by MicroExonator to retrieve the analysed RNA-seq from ENCODE. It contains the ENCODE accession codes and URLs from all the experiments analysed for this article.

**Additional file 8.** R Notebook file.

**Additional file 9.** Review history.

## Data Availability

MicroExonator is freely available, and its current version can be obtained from https://github.com/hemberg-lab/MicroExonator under the MIT license [82]. MicroExonator v1.0.0 was used for the analyses presented in this article, and its source code is available as a Zenodo archive with DOI 10.5281/zenodo.4314702 [83]. The data analysed in this study is available via the ENCODE portal, SRA and GEO under the following accession numbers: SRP055008, GSM1839288 and GSM2971317. The individual accession numbers and URLs for data extracted from ENCODE portal are available as a supplementary material (Additional file 7).
