# Supplementary material for: MicroExonator enables systematic discovery and quantification of microexons across mouse embryonic development
Source: Genome Biol. 2021 Jan 22;22:43. doi: 10.1186/s13059-020-02246-2 (PMC7821500; doi:10.1186/s13059-020-02246-2)
Supplement: Supplementary file 1 — Additional file 1: Figure S1. Number of reads assigned to microexon splice sites during the first and second splice junction alignment performed during discovery and quantification modules respectively. Figure S2. Filtering of putative novel and annotated microexons. Figure S3. Microexon false discovery rate across evaluated software. Figure S4. PCA analysis of 289 bulk RNA-seq samples. Figure S5. A) PCA plot where only the ENCODE samples are shown. Neuronal samples are color coded on a blue to red scale based on developmental time. B) Relationship between PC1 and mouse developmental stage (age) of ENCODE samples. Figure S6. Relationship between mean conservation score (PhyloP) and fraction of in-frame microexons for different microexon clusters and developmental stages. Figure S7. Average Microexon PSI for each microexon cluster across the different sample clusters are shown in red. Grey lines show the average PSI of individual microexons. Figure S8. Boxplots showing PC1-3 loading factors for the different microexon clusters. Figure S9. Volcano plots showing the distribution of delta PSI values of microexon splicing nodes and their corresponding probability of being differentially included across MHN samples coming from different developmental stages (E10.5-E16.5). Figure S10. Differences in PSI values between adrenal gland, brain MHN and forebrain tissues. Figure S11. String PPI network of genes that were detected to have differentially included microexons between the control group and neuronal samples. Figure S12. PPI network corresponding to the group of genes that were detected to have differentially included microexons between the control groups and A) Heart B) Skeletal muscle C) Adrenal gland. Table S3. PPI network summary statistics reported by STRING. [file 13059_2020_2246_MOESM1_ESM.pdf]

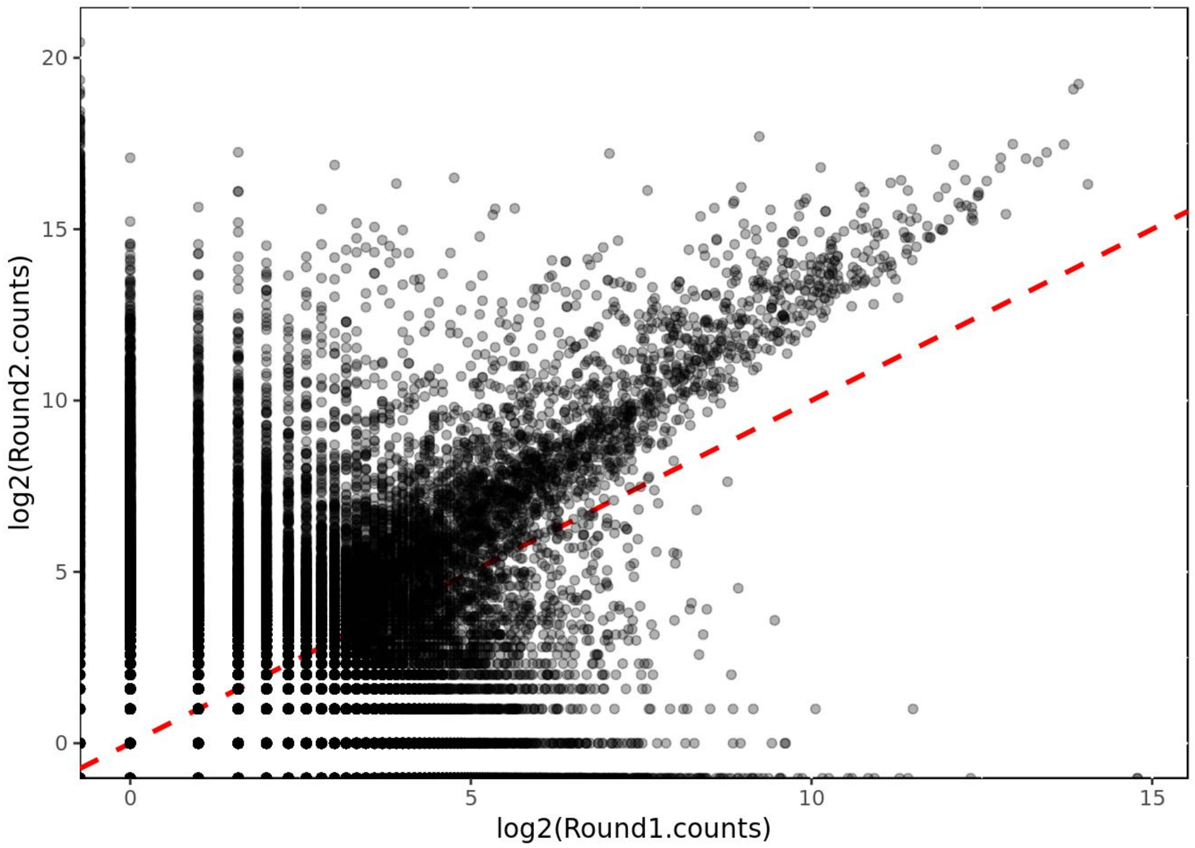

Figure S1: Number of reads assigned to microexon splice sites during the first and second splice junction alignment performed during discovery and quantification modules respectively.

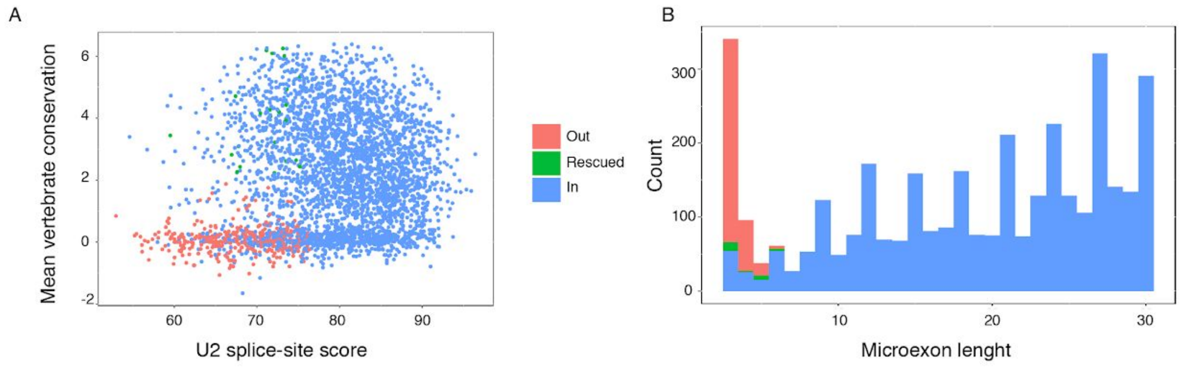

Figure S2. Filtering of putative novel and annotated microexons. A) Conservation and splicing strength of microexons that pass the final quantitative filtering steps. A few microexons were initially filtered out were rescued based on their conservation signal (Phylop score >= 2). B) Size distribution of filtered microexons.

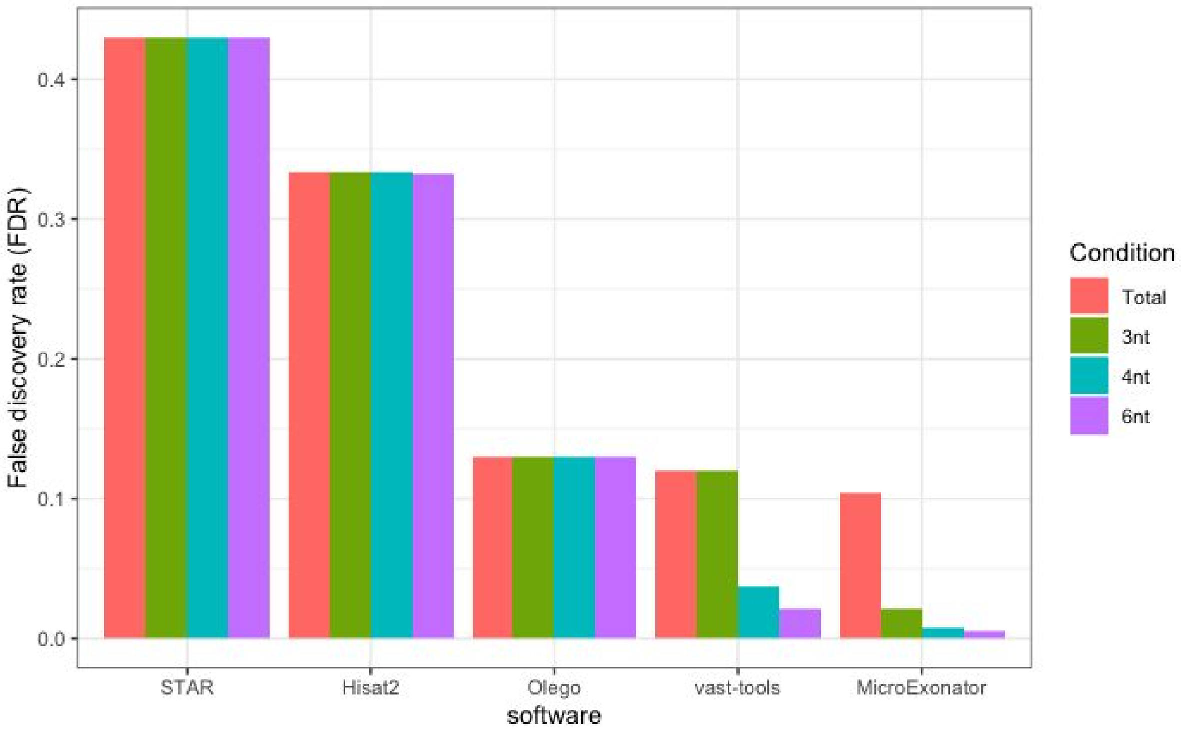

Figure S3. Microexon false discovery rate across evaluated software. Bar colour indicates the smallest microexon size that was considered. Both vast-tools and MicroExonator exhibit decreasing FDR when the microexon length threshold is higher.

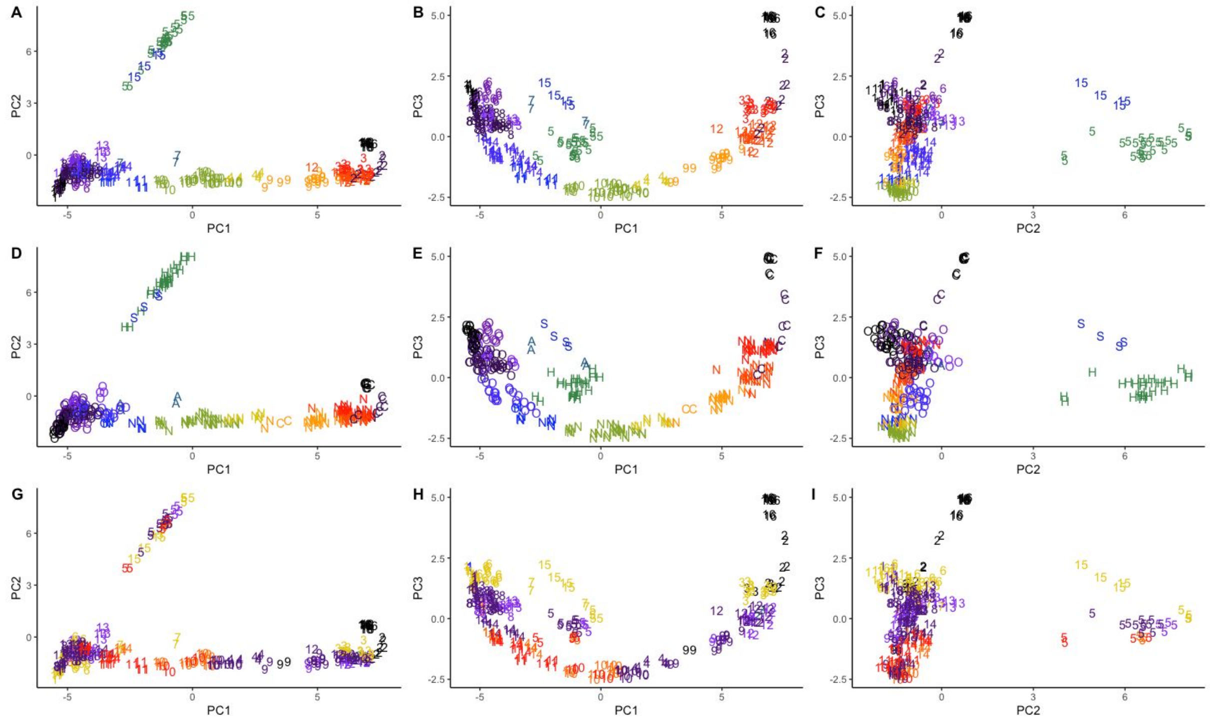

Figure S4. PCA analysis of 289 bulk RNA-seq samples. A-C)**.** Labels in the plot correspond to the tissue cluster number which each sample belongs to. D-F) Each sample was labeled according to the first letter of the tissue of origin, which are; **s**keletal muscle, **h**eart, **a**drenal gland, **n**euronal (forebrain, hindbrain, midbrain, neural tube), **c**ortex and **o**thers. G-I)**.** Each sample was labeled according to the a sample batch ID number, showing that the samples do not cluster by batch group.

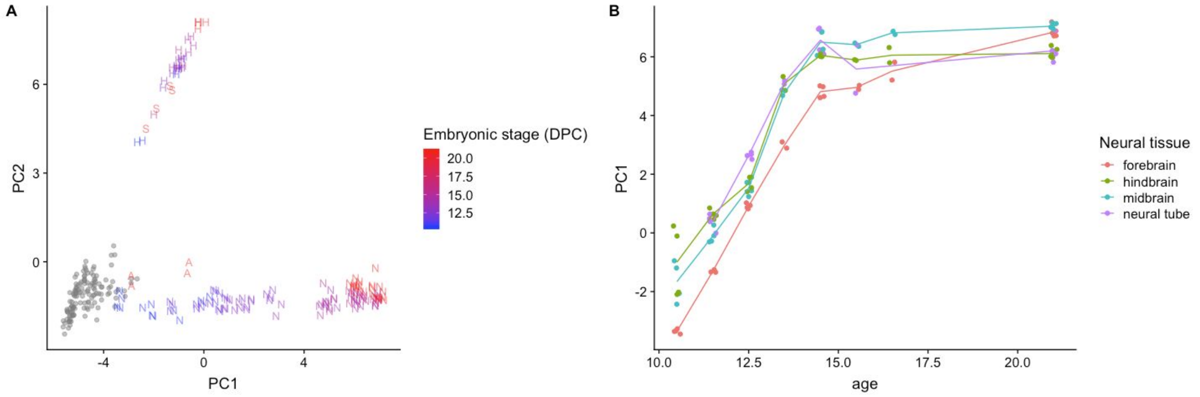

Figure S5. A) PCA plot where only the ENCODE samples are shown. Neuronal samples are color coded on a blue to red scale based on developmental time. B) Relationship between PC1 and mouse developmental stage (age) of ENCODE samples.

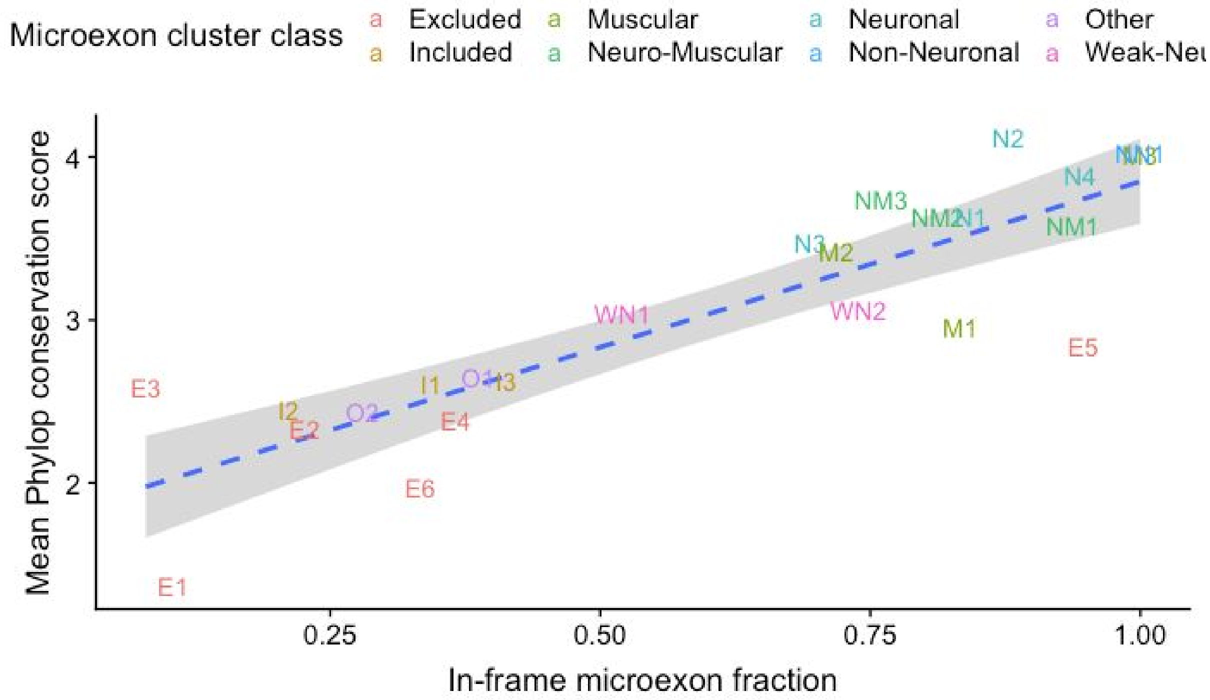

Figure S6. Relationship between mean conservation score (PhyloP) and fraction of in-frame microexons for different microexon clusters and developmental stages.

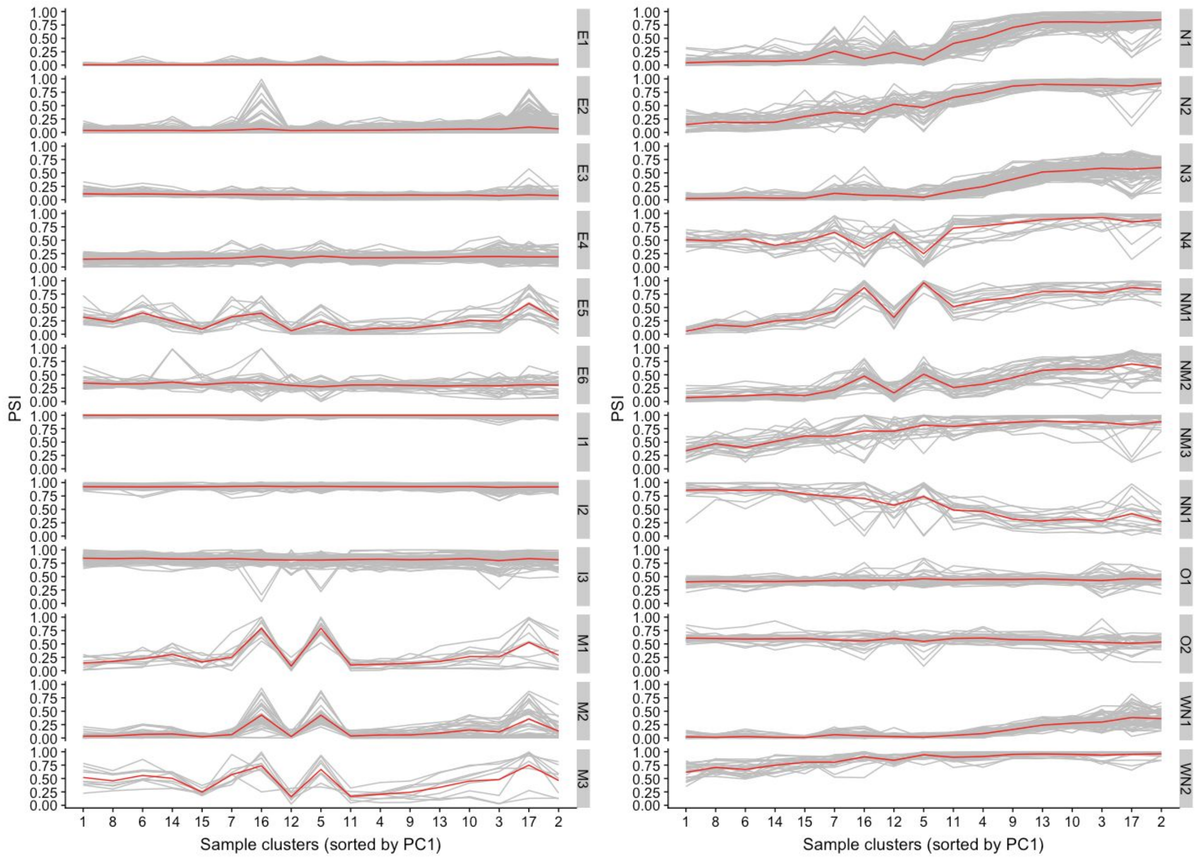

Figure S7. Average Microexon PSI for each microexon cluster across the different sample clusters are shown in red. Grey lines show the average PSI of individual microexons.

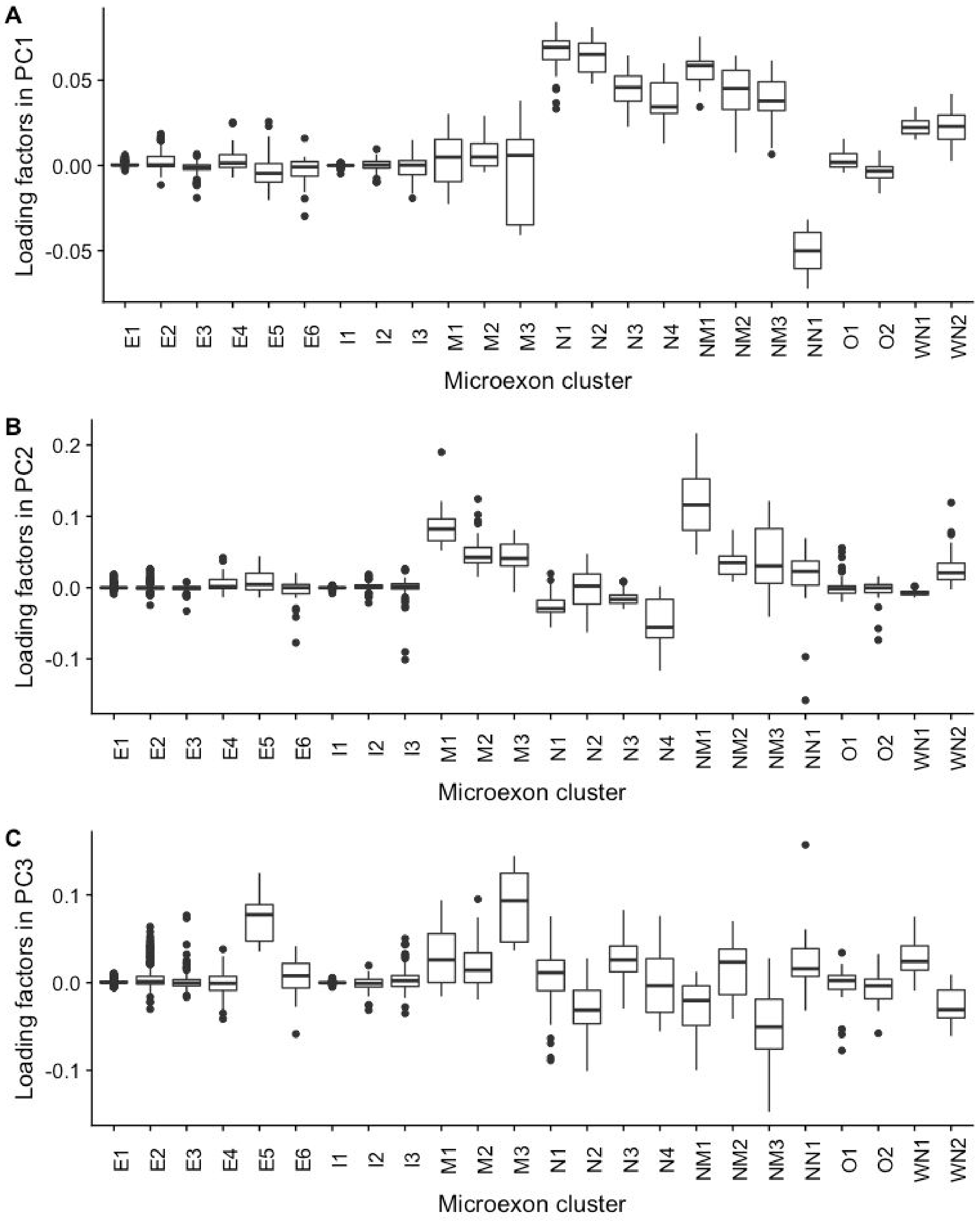

Figure S8. Boxplots showing PC1-3 loading factors for the different microexon clusters.

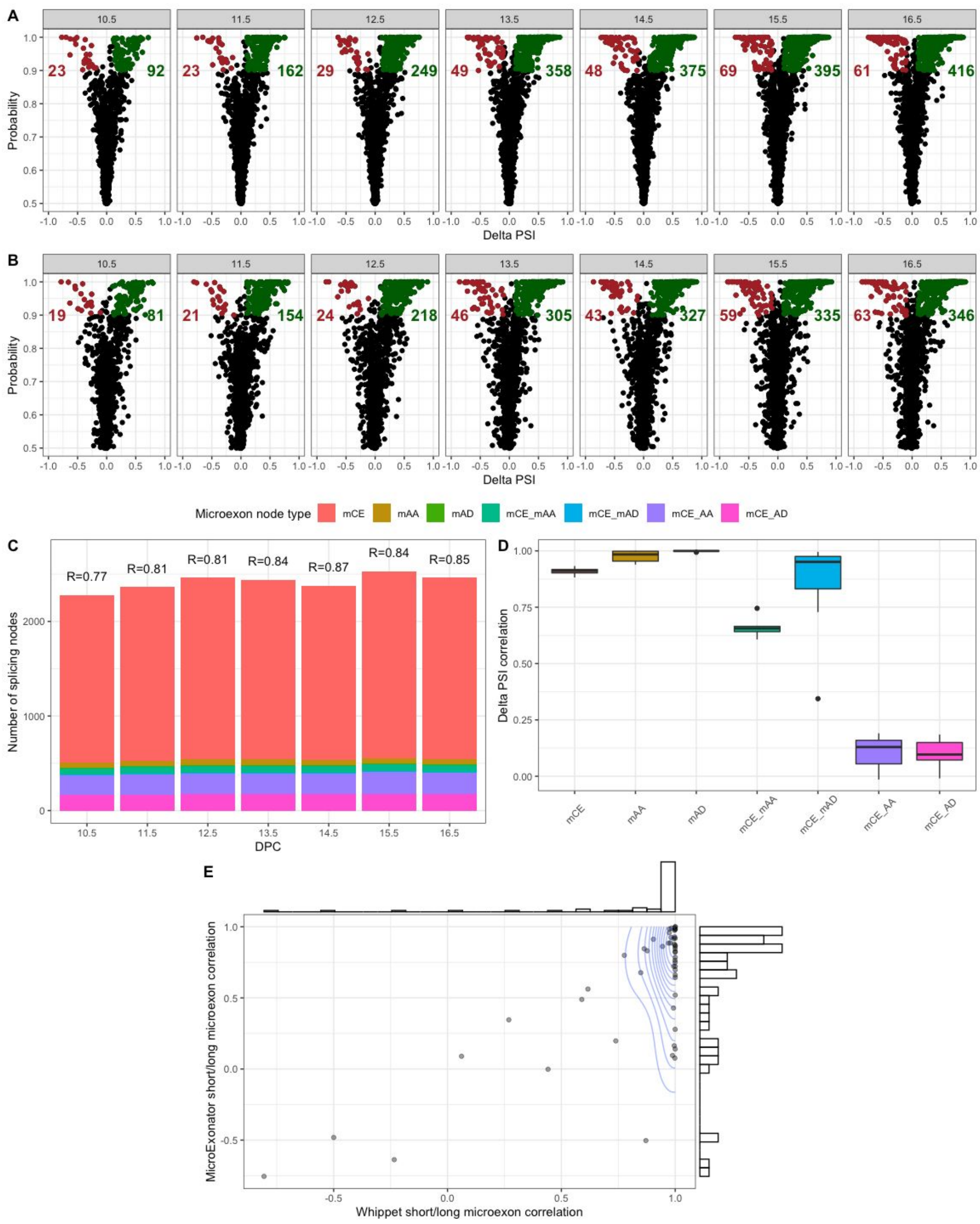

Figure S9: Volcano plots showing the distribution of delta PSI values of microexon splicing nodes and their corresponding probability of being differentially included across MHN samples coming from different developmental stages (E10.5-E16.5). Delta PSI measurements were calculated using Whippet (A) or MicroExonator (B) microexon inclusion quantification. Alternatively included splicing nodes are highlighted in red (excluded) and green (included). Coloured numbers indicate the corresponding quantity of each group of differentially included splicing nodes. C-D) Microexon quantification was derived from different types of Whippet's splicing nodes: core exon (CE), alternative acceptor (AA) and alternative donor (AD). Splicing nodes corresponding to microexon events are denoted with "m" as a prefix. If a microexon is flanked by alternative splicing sites that produce a longer microexon, the CE nodes will be next to a AA or AD node representing a exon or microexon node (mCE_mAA, mCE_mAD, mCE_AA and mCE_AD). The number of nodes in each category and the corresponding PSI correlation coefficient values are shown in (C). The

correlation for each splicing node is shown in (D), revealing a particularly low correlation for mCE_AA and mCE_AD splicing nodes, which correspond to microexons which are associated with alternative splice sites that lead to the inclusion of longer exons (> 30 nt). E) Correlation between PSI estimates from Whippet and MicroExonator for mCE_AA/mCE_AD nodes and their corresponding longer exon. MicroExonator's PSI measurement exhibited greater independence.

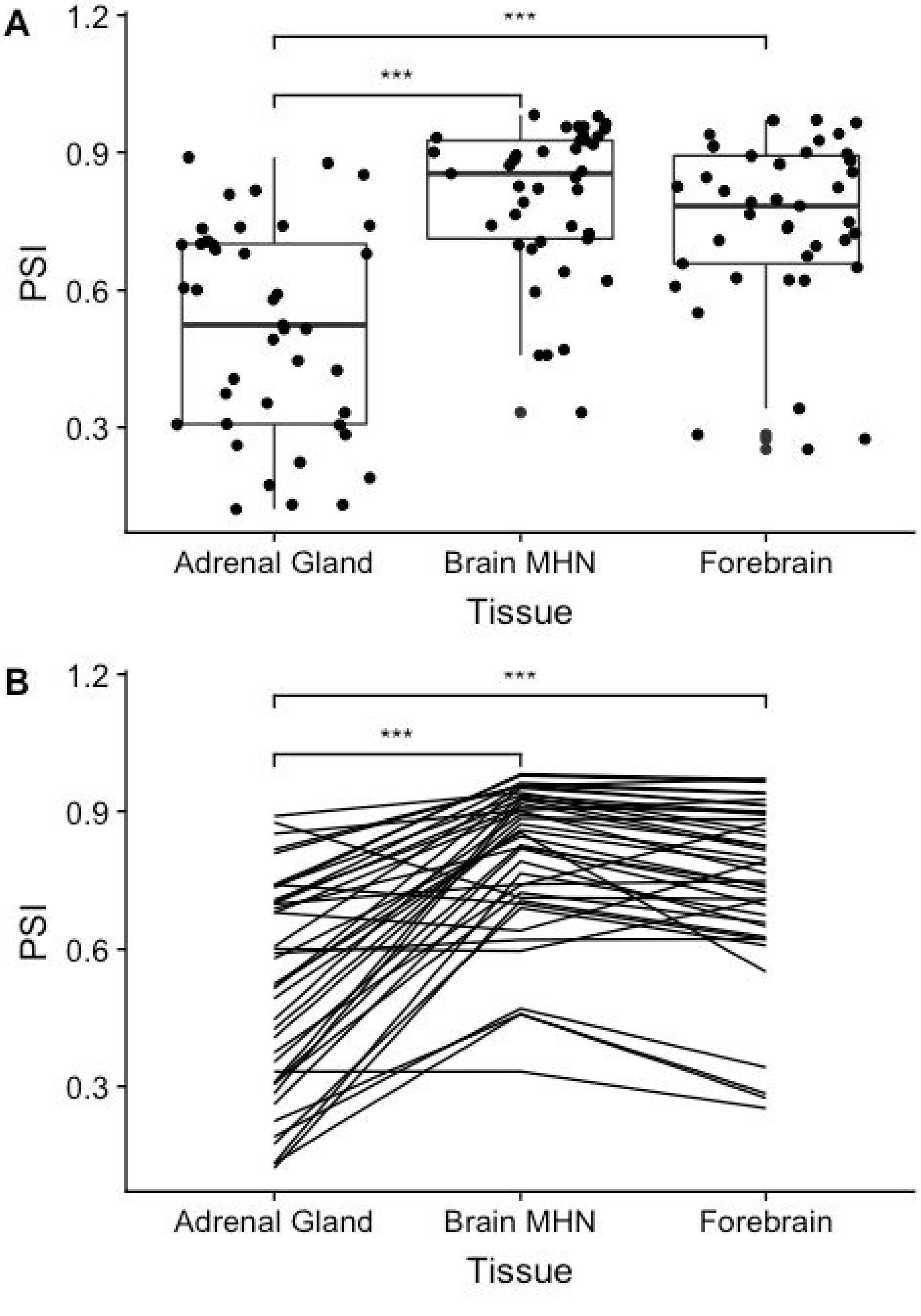

Figure S10: Differences in PSI values between adrenal gland, brain MHN and forebrain tissues. A) Boxplots showing PSI values of each group. Dots represent mean PSI values of each differentially included microexon across the different groups. B) Line plot showing the average PSI variation of each differentially included microexon across the different sample groups. Significant p-values are denoted by * (>0.05), ** (>0.01) and *** (>0.001).

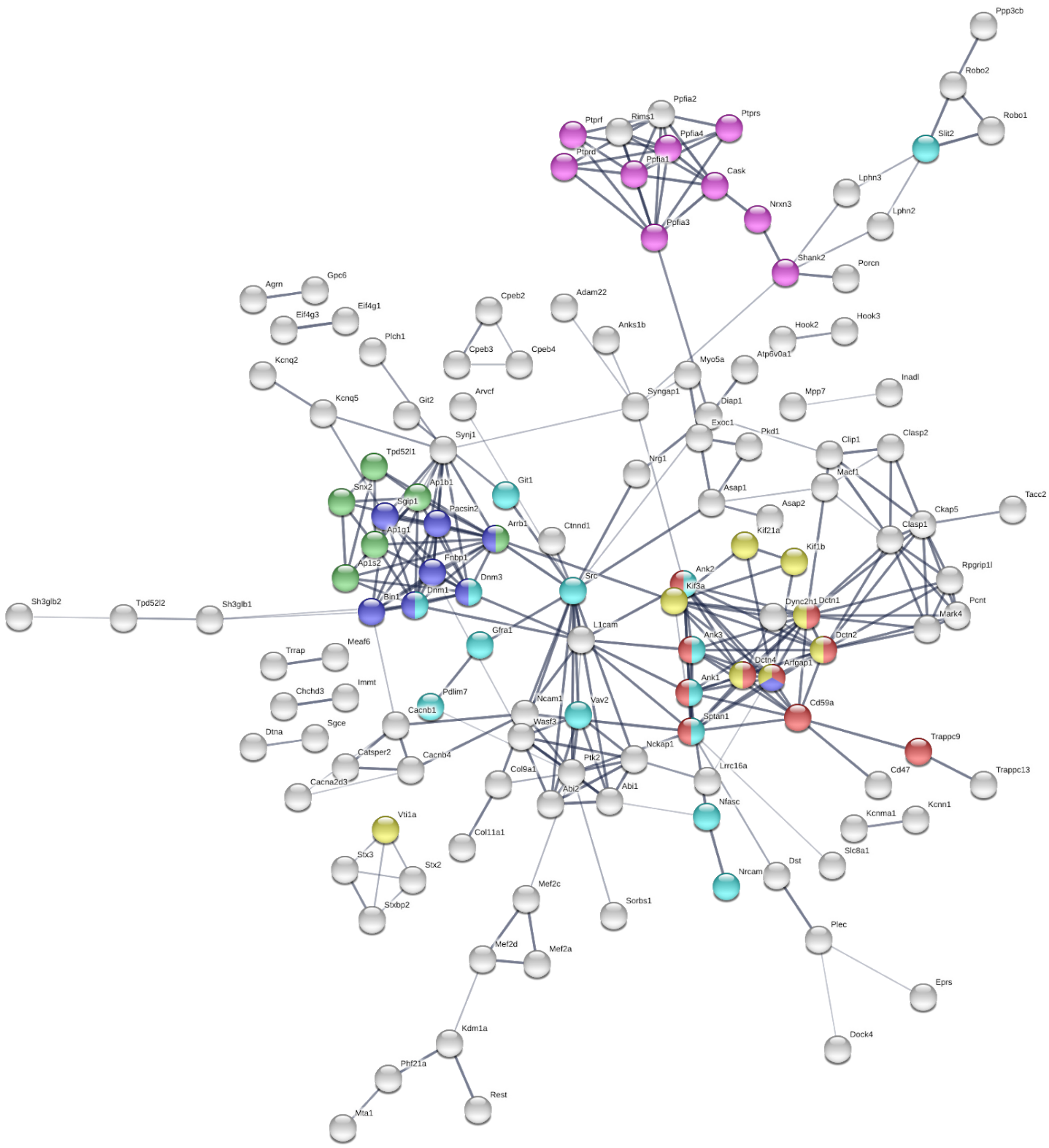

Figure S11. String PPI network of genes that were detected to have differentially included microexons between the control group and neuronal samples. Colors represent different Reactome pathways that were enriched on the network; Axon guidance (light blue), Protein-protein interactions at synapses (pink), ER to Golgi anterograde transport (red), Clathrin-mediated endocytosis (dark blue), Golgi associated vesicle biogenesis (green), Intra-Golgi and retrograde Golgi-to-ER traffic (yellow).

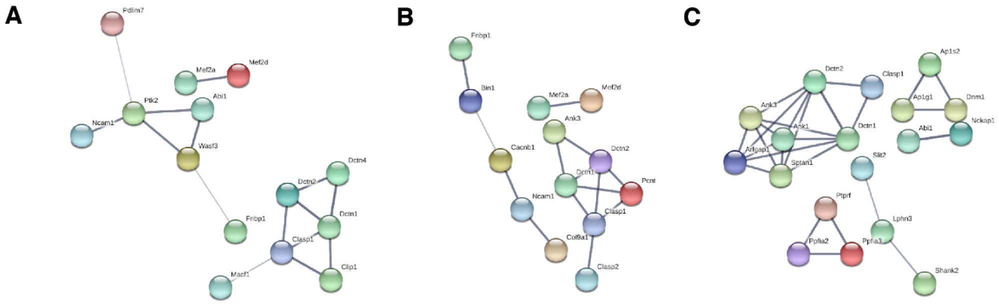

Figure S12. PPI network corresponding to the group of genes that were detected to have differentially included microexons between the control groups and A) Heart B) Skeletal muscle C) Adrenal gland.

Table S3. PPI network summary statistics reported by STRING.

PPI network	Number of nodes	Expected number of edges	Number of edges	Average node degree	Average local clustering coefficient	PPI enrichment p-value
**Brain**	268	133	290	2.16	0.343	< 1.0e-16
**Heart**	47	5	15	0.638	0.234	0.000252
**SKM**	47	5	14	0.596	0.188	0.000397
**AD**	43	6	26	1.21	0.383	3.01e-09
