# Supplementary material for: MicroExonator enables systematic discovery and quantification of microexons across mouse embryonic development
Source: Genome Biol. 2021 Jan 22;22:43. doi: 10.1186/s13059-020-02246-2 (PMC7821500; doi:10.1186/s13059-020-02246-2)
Supplement: Supplementary file 8 — Additional file 8. R Notebook file. [file 13059_2020_2246_MOESM8_ESM.html]

R Notebook


Code 

- Show All Code
- Hide All Code
- Download Rmd

# R Notebook


```
library(reshape2)
```


```
Attaching package: ‘reshape2’

The following objects are masked from ‘package:data.table’:

    dcast, melt
```


```
library(ggplot2)
library(gplots)
```


```
package ‘gplots’ was built under R version 3.5.2
Attaching package: ‘gplots’

The following object is masked from ‘package:IRanges’:

    space

The following object is masked from ‘package:S4Vectors’:

    space

The following object is masked from ‘package:stats’:

    lowess
```


```
library(pheatmap)
```


```
package ‘pheatmap’ was built under R version 3.5.2
```


```
library(plyr)
```


```
package ‘plyr’ was built under R version 3.5.2
Attaching package: ‘plyr’

The following object is masked from ‘package:IRanges’:

    desc

The following object is masked from ‘package:S4Vectors’:

    rename
```


```
set.seed(123)
library(data.table)
library(magrittr)
library(dplyr)
```


```
package ‘dplyr’ was built under R version 3.5.2
Attaching package: ‘dplyr’

The following objects are masked from ‘package:plyr’:

    arrange, count, desc, failwith, id, mutate, rename, summarise, summarize

The following objects are masked from ‘package:data.table’:

    between, first, last

The following object is masked from ‘package:AnnotationDbi’:

    select

The following objects are masked from ‘package:GenomicRanges’:

    intersect, setdiff, union

The following object is masked from ‘package:GenomeInfoDb’:

    intersect

The following objects are masked from ‘package:IRanges’:

    collapse, desc, intersect, setdiff, slice, union

The following objects are masked from ‘package:S4Vectors’:

    first, intersect, rename, setdiff, setequal, union

The following object is masked from ‘package:Biobase’:

    combine

The following objects are masked from ‘package:BiocGenerics’:

    combine, intersect, setdiff, union

The following object is masked from ‘package:biomaRt’:

    select

The following objects are masked from ‘package:stats’:

    filter, lag

The following objects are masked from ‘package:base’:

    intersect, setdiff, setequal, union
```


```
library(readr)
library(cowplot)
```


```
package ‘cowplot’ was built under R version 3.5.2
********************************************************
Note: As of version 1.0.0, cowplot does not change the
  default ggplot2 theme anymore. To recover the previous
  behavior, execute:
  theme_set(theme_cowplot())
********************************************************
```


```
library(igraph)
```


```
package ‘igraph’ was built under R version 3.5.2
Attaching package: ‘igraph’

The following objects are masked from ‘package:dplyr’:

    as_data_frame, groups, union

The following object is masked from ‘package:GenomicRanges’:

    union

The following object is masked from ‘package:IRanges’:

    union

The following object is masked from ‘package:S4Vectors’:

    union

The following objects are masked from ‘package:BiocGenerics’:

    normalize, path, union

The following objects are masked from ‘package:stats’:

    decompose, spectrum

The following object is masked from ‘package:base’:

    union
```


```
theme_set(theme_classic() )
```


```
get_colors <- function(groups, group.col = palette()){
  groups <- as.factor(groups)
  ngrps <- length(levels(groups))
  if(ngrps > length(group.col)) 
    group.col <- rep(group.col, ngrps)
  color <- group.col[as.numeric(groups)]
  names(color) <- as.vector(groups)
  return(color)
}


gg_color_hue <- function(n) {
  hues = seq(15, 375, length = n + 1)
  hcl(h = hues, l = 65, c = 100)[1:n]
}
```


Importing metadata


```
metadata
```


```
     File.accession Biosample.Age Experiment.date.released Biosample.term.name Paired.end Paired.with Audit.WARNING
  1:    ENCFF405VKS      13.5 day               2015-10-13         neural tube         NA                          
  2:    ENCFF241GLU      13.5 day               2015-10-13         neural tube         NA                          
  3:    ENCFF957SPL         0 day               2015-06-23         neural tube         NA                          
  4:    ENCFF046EJC         0 day               2015-06-23         neural tube         NA                          
  5:    ENCFF085MBO         0 day               2015-06-23         neural tube         NA                          
 ---                                                                                                               
293:     SRR1805824          P30a                    16484        whole cortex         NA                          
294:     SRR1805823         P110a                    16484        whole cortex         NA                          
295:     SRR1805822         P110b                    16484        whole cortex         NA                          
296:     SRR1805821          21Ma                    16484        whole cortex         NA                          
297:     SRR1805820          21Mb                    16484        whole cortex         NA
```


```
ME_final.rep1 <- fread("~/Google_Drive/Results/ME/Paper/Final_Report/Reps/Rep1/out.high_quality.txt")
ME_final.rep2 <- fread("~/Google_Drive/Results/ME/Paper/Final_Report/Reps/Rep2/out.high_quality.txt")

ME_final <- ME_final.rep1[ ME %in% ME_final.rep2$ME, ]


ME_cov <- fread("~/Google_Drive/Results/ME/Paper/Final_Report/Reps/Rep1/out_filtered_ME.PSI.txt")
ME_cov[, `:=`(FILE_NAME=File, ME=ME_coords)]

number_of_microexons <- length(ME_final[ , unique(ME)])


ME_cov_filtered <- ME_cov[ME %in% ME_final[, ME]  , ]
```


```
library(stringi)


#mat <- stri_split_fixed(ME_cov_filtered$ME_coverages, ',', simplify=T)
#mat <- `dim<-`(as.numeric(mat), dim(mat))  # convert to numeric and save dims
#rowsum(mat, na.rm=T)

#sum(mat, na.rm=T)


#sapply(strsplit(as.character(ME_cov_filtered$ME_coverages), ",", fixed=T), function(x) sum(as.numeric(x)))
```


```
#ME_cov_filtered[, strsplit( ME_coverages, ",") ]


#library(dplyr)
#library(tidyr)
#df %>%
#   separate_rows(y, z, convert = TRUE) %>%
#   group_by(x) %>% 
#   summarise_all(sum)
```


Obtainin PSI matrix


```
Tissues <- as.character(metadata$Biosample.term.name)
names(Tissues) <- as.character(metadata$File.accession)

Ages <- as.character(metadata$Biosample.Age)
names(Ages) <- as.character(metadata$File.accession)


ME_cov_filtered  <- unique(ME_cov_filtered[FILE_NAME %in% metadata[ , File.accession],])
#ME_cov_filtered_ENCODE <- unique(ME_cov_filtered[FILE_NAME %in% metadata[ , File.accession],])


#colnames(Tissue_PSI_matrix_dcast) <- as.character( paste(sep = " ", Tissues[colnames(Tissue_PSI_matrix_dcast)], Ages[colnames(Tissue_PSI_matrix_dcast)])) #To replase the file name by the biological sample name

#Tissue_PSI_matrix_dcast <- Tissue_PSI_matrix_dcast[, order(paste(sep = " ", Tissues[colnames(Tissue_PSI_matrix_dcast)], Ages[colnames(Tissue_PSI_matrix_dcast)]))]
```


Probabilistic PCA


```
library(pcaMethods)

Tissue_PSI_matrix_melt <- ME_cov_filtered[, c("ME", "FILE_NAME", "PSI") ]
Tissue_PSI_matrix_dcast <- reshape2::dcast(Tissue_PSI_matrix_melt, ME ~ FILE_NAME)
row.names(Tissue_PSI_matrix_dcast) <- Tissue_PSI_matrix_dcast$ME
Tissue_PSI_matrix_dcast <- data.matrix(Tissue_PSI_matrix_dcast)[,-1]


Tissue_PSI_matrix_dcast[Tissue_PSI_matrix_dcast=="NaN"] <- NA

dim(Tissue_PSI_matrix_dcast)

dim(Tissue_PSI_matrix_dcast[apply(Tissue_PSI_matrix_dcast, 1, function(x) length(which(is.na(x)))) < 271*0.9, ])

dim(Tissue_PSI_matrix_dcast[apply(Tissue_PSI_matrix_dcast, 1, function(x) length(which(is.na(x)))) < 289*0.9, ])

result <- pca(t(Tissue_PSI_matrix_dcast[apply(Tissue_PSI_matrix_dcast, 1, function(x) length(which(is.na(x)))) < 289*0.9, ]), method="ppca", nPcs=3, seed=123)
## Get the estimated complete observations
cObs <- completeObs(result)
## Plot the scores
plotPcs(result, type = "scores")


summary(result)
```


```
Tissue_PSI_matrix_dcast_ppca <- t(cObs)

Tissue_PSI_matrix_dcast_ppca[Tissue_PSI_matrix_dcast_ppca>1] <- 1
Tissue_PSI_matrix_dcast_ppca[Tissue_PSI_matrix_dcast_ppca<0] <- 0


##colnames(Tissue_PSI_matrix_dcast_ppca) <- as.character( paste(sep = " ", Tissues[colnames(Tissue_PSI_matrix_dcast_ppca)], Ages[colnames(Tissue_PSI_matrix_dcast_ppca)])) #To replase the file name by the biological sample name


#tissue_heatmap <- pheatmap::pheatmap(Tissue_PSI_matrix_dcast_ppca,  , fontsize = 4,  cutree_rows = 24, cutree_cols = 16, clustering_method = "ward.D2")
```


```
#Tissue_PSI_matrix_dcast_ppca <- t(cObs)

#Tissue_PSI_matrix_dcast_ppca[Tissue_PSI_matrix_dcast_ppca>1] <- 1
#Tissue_PSI_matrix_dcast_ppca[Tissue_PSI_matrix_dcast_ppca<0] <- 0


##colnames(Tissue_PSI_matrix_dcast_ppca) <- as.character( paste(sep = " ", Tissues[colnames(Tissue_PSI_matrix_dcast_ppca)], Ages[colnames(Tissue_PSI_matrix_dcast_ppca)])) #To replase the file name by the biological sample name


#tissue_heatmap <- pheatmap::pheatmap(Tissue_PSI_matrix_dcast_ppca,  , fontsize = 4,  cutree_rows = 24, cutree_cols = 17, clustering_method = "ward.D2")
```


```
hc_cols <- hclust(dist(t(Tissue_PSI_matrix_dcast_ppca)), method = "ward.D2")
hc_rows <- hclust(dist(Tissue_PSI_matrix_dcast_ppca), method = "ward.D2")
```


```
Tissues_name <- as.character(metadata$Biosample.term.name)
names(Tissues_name) <- as.character(metadata$File.accession)

Tissues_age <- as.character(metadata$Biosample.Age)
names(Tissues_age) <- as.character(metadata$File.accession)

Tissue_date <- as.character(metadata$Experiment.date.released)
names(Tissue_date) <- as.character(metadata$File.accession)
```


```
#Tissue_clusters <- cutree(hc_cols, k = 16)
#Tissue_clusters <- cbind(Tissue_clusters, Tissues_name[names(Tissue_clusters)], Tissues_age[names(Tissue_clusters)], Tissue_date[names(Tissue_clusters)]) 

#colnames(Tissue_clusters) <- c("cluster", "name", "age", "date")

#Tissue_clusters <- data.frame(Tissue_clusters)

##Tissue_clusters_sum[which(grepl("hindbrain|midbrain|forebrain|neural\ tube", Tissue_clusters_sum$name)), ]

#Tissue_clusters$name <- factor(Tissue_clusters$name, level=c( "skeletal muscle tissue", "heart", "thymus", "spleen", "liver",  "adrenal gland", "intestine", "stomach", "lung",  "kidney", "bladder", "limb", "embryonic facial prominence", "forebrain", "hindbrain", "midbrain", "neural tube", "whole cortex"))


#Tissue_clusters$age <- mapvalues(Tissue_clusters$age, 
#          from = c("10.5 day", "11.5 day", "12.5 day", "13.5 day", "14.5 day", "15.5 day", "16.5 day", "0 day", "8 week", "P0a", "P0b", "P110a", "P110b", "P15a", "P15b", "P30a", "P30b", "P4a", #"P4a", "P7a", "P7b", "21Ma", "21Mb", "E14.5", "E16.5", "P4b"),
#         to = c(10.5, 11.5, 12.5, 13.5, 14.5, 15.5, 16.5, 21, 59, 21, 21, 131, 131, 36, 36, 41,41, 25, 25, 27,27, 651, 651, 14.5, 16.5, 25 ) )


#Tissue_clusters$age <- factor(Tissue_clusters$age, levels = as.character(sort(as.numeric(levels(Tissue_clusters$age)))))
```


```
Tissue_clusters <- cutree(hc_cols, k = 16)
Tissue_clusters <- cbind(Tissue_clusters, Tissues_name[names(Tissue_clusters)], Tissues_age[names(Tissue_clusters)], Tissue_date[names(Tissue_clusters)]) 

colnames(Tissue_clusters) <- c("cluster", "name", "age", "date")

Tissue_clusters <- data.frame(Tissue_clusters)

#Tissue_clusters_sum[which(grepl("hindbrain|midbrain|forebrain|neural\ tube", Tissue_clusters_sum$name)), ]

Tissue_clusters$name <- factor(Tissue_clusters$name, level=c( "skeletal muscle tissue", "heart", "thymus", "spleen", "liver",  "adrenal gland", "intestine", "stomach", "lung",  "kidney", "bladder", "limb", "embryonic facial prominence", "forebrain", "hindbrain", "midbrain", "neural tube", "whole cortex"))

#Tissue_clusters$age <- factor(Tissue_clusters$age, levels=c("10.5 day", "11.5 day", "12.5 day", "13.5 day", "14.5 day", "15.5 day", "16.5 day", "0 day", "8 week"))

Tissue_clusters$File.accession <- row.names(Tissue_clusters)


Tissue_clusters <- data.table(Tissue_clusters)


Tissue_clusters$age <- mapvalues(Tissue_clusters$age, 
          from = c("10.5 day", "11.5 day", "12.5 day", "13.5 day", "14.5 day", "15.5 day", "16.5 day", "0 day", "8 week", "P0a", "P0b", "P110a", "P110b", "P15a", "P15b", "P30a", "P30b", "P4a", "P4a", "P7a", "P7b", "21Ma", "21Mb", "E14.5", "E16.5", "P4b"),
          to = c(10.5, 11.5, 12.5, 13.5, 14.5, 15.5, 16.5, 21, 59, 21, 21, 131, 131, 36, 36, 41,41, 25, 25, 27,27, 651, 651, 14.5, 16.5, 25 ) )
```


```
ME_clusters <- cutree(hc_rows, k = 24)

PCA_loadings <- data.frame(loadings(result))
PCA_loadings$ME <- row.names(PCA_loadings)


PCA_loadings <- data.table(PCA_loadings)
PCA_loadings[, `:=`(PC1=-PC1, PC2=-PC2)]


PCA_loadings$ME_cluster <- ME_clusters[PCA_loadings$ME]


PCA_loadings_stats <- PCA_loadings[ , .(PC1_mean=mean(PC1)) , by="ME_cluster"]
ME_cluster_loading_order <- PCA_loadings_stats[order(PC1_mean)]$ME_cluster

PCA_loadings$ME_cluster <- factor(PCA_loadings$ME_cluster , levels=ME_cluster_loading_order)


ggplot(PCA_loadings) +
  geom_boxplot(aes(as.factor(ME_cluster) , PC1))
```


```
ggplot(PCA_loadings) +
  geom_boxplot(aes(as.factor(ME_cluster) , PC2))
```


```
ggplot(PCA_loadings) +
  geom_boxplot(aes(as.factor(ME_cluster) , PC3))
```


```
PCA_loadings_stats <- PCA_loadings[ , .(PC1_mean=mean(PC1)) , by="ME_cluster"]

PCA_loadings_stats_PC2 <- PCA_loadings[ , .(PC2_mean=mean(PC2)) , by="ME_cluster"]


ME_cluster_loading_order <- PCA_loadings_stats[order(PC1_mean)]$ME_cluster
```


Neuronal Microexons


```
PCA_loadings_stats[abs(PC1_mean)>0.03, ][order(PC1_mean)]
```


```
PCA_loadings_stats_PC2[abs(PC2_mean)>0.03, ][order(-PC2_mean)]
```


```
Tissue_PSI_matrix_melt_ppca <- melt(Tissue_PSI_matrix_dcast_ppca)

colnames(Tissue_PSI_matrix_melt_ppca) <- c("ME", "FILE_NAME", "PSI")

ME_clusters_table <- data.frame(ME_clusters)

ME_clusters_table$ME <- row.names(ME_clusters_table)
ME_clusters_table <- data.table(ME_clusters_table)


ME_clusters_PSI <-  data.table(merge(Tissue_PSI_matrix_melt_ppca, ME_clusters_table , by=c("ME")))


fwrite( ME_clusters_PSI, "../../Paper/Jacob/ME_clusters_PSI.tvs", quote = FALSE, col.names = TRUE, sep="\t")

fwrite( ME_cluster_names, "../../Paper/Jacob/ME_cluster_names.tvs", quote = FALSE, col.names = TRUE, sep="\t")
```


```
ME_clusters_PSI$ME_clusters <- mapvalues(ME_clusters_PSI$ME_clusters, 
          from =1:18,
          to = c("I1", "E1", "E3", "I2", "N1", "M1", "N2", "NM3", "NM2", "N5", "NM1", "N3", "N4", "NN2", "E2", "I4", "I3", "NN1"))
```


```
Tissue_PSI_matrix_melt_ppca <- data.table(Tissue_PSI_matrix_melt_ppca)

Tissue_PSI_matrix_melt_ppca
```


```
PCA_loadings_stats <- PCA_loadings[ , .(PC1_mean=mean(PC1), PC2_mean=mean(PC2) ) , by="ME_cluster"]

#PCA_loadings_stats[, PC1_mean:=-PC1_mean]

PCA_loadings_stats[order(-PC1_mean)]
PCA_loadings_stats[order(PC2_mean)]

#PCA_loadings_stats_PC2[, PC2_mean:=-PC2_mean]
```


```
PCA_loadings_stats[order(PC2_mean)]
```


```
loading_threshold <- 0.010
loading_threshold_PC2 <- 0.010


Neuronal <- PCA_loadings_stats[ round(PC1_mean, 3) >  loading_threshold, ][order(-PC1_mean)]$ME_cluster
Muscular <- PCA_loadings_stats_PC2[round(PC2_mean, 3) < -loading_threshold_PC2, ][order(PC2_mean)]$ME_cluster

NonNeuronal<- PCA_loadings_stats[ round(PC1_mean, 3) <= -loading_threshold, ][order(PC1_mean)]$ME_cluster

#WeakNeuronal <- PCA_loadings_stats[  round(PC1_mean, 3) < loading_threshold & round(PC1_mean, 3) >=  0.01, ][order(PC1_mean)]$ME_cluster

N = 1 
NM = 1
NN = 1
M = 1
WN = 1

ME_cluster_names <- data.table()


for (ME_cluster in Neuronal){
  
  if (ME_cluster %in% Muscular){
    
    ME_cluster.name = paste0("NM", NM)
    ME_cluster.type = "Neuro-Muscular"
    
    ME_cluster_names = rbind(ME_cluster_names, cbind(ME_cluster, ME_cluster.name, ME_cluster.type  ))
    
    NM = NM + 1
    
  }

  
  else {
    
    ME_cluster.name = paste0("N", N)
    ME_cluster.type = "Neuronal"
    
    ME_cluster_names = rbind(ME_cluster_names,  cbind(ME_cluster, ME_cluster.name, ME_cluster.type ))
    
    N = N + 1
    
  }
  
}


for (ME_cluster in NonNeuronal) {
  

  ME_cluster.name = paste0("NN", NN)
  ME_cluster.type = "Non-Neuronal"
  
  ME_cluster_names = rbind(ME_cluster_names,  cbind(ME_cluster, ME_cluster.name, ME_cluster.type ))
  
  NN = NN + 1
  
  
}


#for (ME_cluster in WeakNeuronal) {
  

#  ME_cluster.name = paste0("WN", WN)
#  ME_cluster.type = "Weak-Neuronal"
  
#  ME_cluster_names = rbind(ME_cluster_names,  cbind(ME_cluster, ME_cluster.name, ME_cluster.type ))
  
#  WN = WN + 1
  
  
#}


for (ME_cluster in Muscular){
  
  
  if ( (ME_cluster %in% ME_cluster_names$ME_cluster)==F) {
  
  
  ME_cluster.name = paste0("M", M)
  ME_cluster.type = "Muscular"
  
  ME_cluster_names = rbind(ME_cluster_names,  cbind(ME_cluster, ME_cluster.name, ME_cluster.type ))
  
  M = M + 1
  
  }
  
  
  }


ME_cluster_names


ME_clusters_mean_PSIs <-  ME_clusters_PSI[, .(mean_PSI=mean(PSI), sd_PSI=sd(PSI)), by="ME_clusters" ]

ME_clusters_flat <- ME_clusters_mean_PSIs[ ! ME_clusters %in% ME_cluster_names$ME_cluster,  ]


E = 1
I = 1
O = 1 


for (ME_cluster in ME_clusters_flat[ mean_PSI <= 1/3,  ][order(mean_PSI)]$ME_clusters) {
  
  
  ME_cluster.name = paste0("E", E)
  ME_cluster.type = "Excluded"
  
  ME_cluster_names = rbind(ME_cluster_names,  cbind(ME_cluster, ME_cluster.name, ME_cluster.type ))
  
  E = E + 1
  
  
} 


for (ME_cluster in ME_clusters_flat[ mean_PSI>=2/3,  ][order(-mean_PSI)]$ME_clusters) {
  
  
  ME_cluster.name = paste0("I", I)
  ME_cluster.type = "Included"
  
  ME_cluster_names = rbind(ME_cluster_names,  cbind(ME_cluster, ME_cluster.name, ME_cluster.type ))
  
  I = I + 1
  
  
}


for (ME_cluster in ME_clusters_flat[ mean_PSI > 1/3  &  mean_PSI < 2/3  ,  ][order(mean_PSI)]$ME_clusters) {
  
  
  ME_cluster.name = paste0("O", O)
  ME_cluster.type = "Other"
  
  ME_cluster_names = rbind(ME_cluster_names,  cbind(ME_cluster, ME_cluster.name, ME_cluster.type ))
  
  O = O + 1
  
  
} 
ME_cluster_names
```


```
ME_cluster_names$ME_cluster <- as.numeric(ME_cluster_names$ME_cluster)

ME_cluster_names <- merge( x=ME_cluster_names, y=ME_clusters_mean_PSIs, by.x="ME_cluster", by.y="ME_clusters")

PCA_loadings_stats$ME_cluster <- as.numeric(as.character(PCA_loadings_stats$ME_cluster))

PCA_loadings_stats_PC2$ME_cluster <- as.numeric(as.character(PCA_loadings_stats_PC2$ME_cluster))


ME_cluster_names <- merge(ME_cluster_names, PCA_loadings_stats, by="ME_cluster")
#ME_cluster_names <- merge(ME_cluster_names, PCA_loadings_stats_PC2, by="ME_cluster")

ME_cluster_names$ME_cluster.type <- factor(ME_cluster_names$ME_cluster.type, levels = c("Excluded", "Included", "Neuronal",  "Non-Neuronal", "Muscular",  "Neuro-Muscular", "Other"))
```


```
Fig5.X.2 <- ggplot(ME_cluster_names) +
  geom_text(aes(x=PC1_mean, y=-PC2_mean, label = ME_cluster.name, colour = ME_cluster.type ), ) +
  xlab("Mean PC1 loading") +
  ylab("Mean PC2 loading") +
    theme(legend.position="top")


Fig5.X.2
```


```
Fig5.X.3 <- ggplot(ME_cluster_names) +
  geom_text(aes(x=mean_PSI, y=sd_PSI, label = ME_cluster.name, colour = ME_cluster.type ), ) +
  xlab("Mean PSI") +
  ylab("PSI standar deviation") +
    theme(legend.position="top") 
  


Fig5.X.3
```


```
ME_clusters.table <- cbind(ME_clusters, names(ME_clusters))
colnames(ME_clusters.table) <- c("ME_clusters", "ME")

ME_clusters.table <- data.table(ME_clusters.table)
ME_clusters.table$ME_clusters <- as.numeric(ME_clusters.table$ME_clusters)


ME_cluster_info <- merge(ME_clusters.table, ME_final[, c("ME", "len_micro_exon_seq_found", "U2_scores")], by="ME")
ME_cluster_info <- data.table(ME_cluster_info)


ME_cluster_info_by <- ME_cluster_info[ , .(asym = sum(len_micro_exon_seq_found %% 3),
                    mean_U2_score = mean(U2_scores),
                    total=.N
                    ), by="ME_clusters"  ]

ME_cluster_info_by[, symetrical_fraction:=(total-asym)/total]


ME_cluster_names <- merge(ME_cluster_names, ME_cluster_info_by, by.x="ME_cluster", by.y="ME_clusters")


#ME_cluster_info <- merge(ME_cluster_info_by[, .(ME_clusters=unique(ME_clusters)), by=ME], ME_final[, c("ME", "len_micro_exon_seq_found", "U2_scores")], by="ME")


 


Fig5.X.4 <- ggplot(ME_cluster_names) +
  geom_text(aes(x=symetrical_fraction, y=mean_U2_score, label = ME_cluster.name, colour = ME_cluster.type ), ) +
  xlab("In-frame microexon fraction") +
  ylab("Mean splicing strength") +
    theme(legend.position="top")


Fig5.X.4
```


```
ME_cluster_names[ ME_cluster.type=="Excluded", ]
ME_cluster_names[ ME_cluster.type=="Included", ]
ME_cluster_names[ ME_cluster.type=="Neuronal", ]
ME_cluster_names[ ME_cluster.type=="Neuro-Muscular", ]
ME_cluster_names[ ME_cluster.type=="Neuronal", ]
ME_cluster_names[ ME_cluster.type=="Included", ]
ME_cluster_names[ ME_cluster.type=="Excluded", ]
ME_cluster_names[ ME_cluster.type=="Included", ]
ME_cluster_names[ ME_cluster.type=="Other", ]

levels(factor(ME_cluster_names$ME_cluster.type))
```


```
ME_cluster_names.levels <- c()

for (i in levels(factor(ME_cluster_names$ME_cluster.type))){
  
  
  
  
  ME_cluster_names.levels<- c(ME_cluster_names.levels, ME_cluster_names[ME_cluster.type==i, ME_cluster.name])
  
  
}
```


```
Fig5.X.1.legend
```


```
TableGrob (5 x 5) "guide-box": 2 grobs
                                    z     cells                  name           grob
99_4000b8f4423e777ea3de92bc832bc4ce 1 (3-3,3-3)                guides gtable[layout]
                                    0 (2-4,2-4) legend.box.background zeroGrob[NULL]
```


```
Fig5.X.plots <- plot_grid(Fig5.X.1 + theme(legend.position="none"), 
                    Fig5.X.2 + theme(legend.position="none"), 
                    Fig5.X.3 + theme(legend.position="none"), 
                    Fig5.X.4 + theme(legend.position="none"), 
                    nrow =1,
                    rel_widths = c(1.7,1,1,1),
                    labels = "AUTO" )


Fig5.X <- plot_grid(Fig5.X.1.legend, Fig5.X.plots, ncol = 1, rel_heights = c(0.2, 1))

Fig5.X
```


```
ME_clusters.names <- data.table(ME_clusters)
ME_clusters.names$ME <- names(ME_clusters)
ME_clusters.names <- merge(ME_clusters.names,  ME_cluster_names[, c("ME_cluster", "ME_cluster.name")],  by.x="ME_clusters", by.y="ME_cluster")
```


```
ME_final.ME_clusters <- merge(ME_final, ME_clusters.names, by="ME")

ME_cluster.conservation <- ME_final.ME_clusters[ mean_conservations_vertebrates!="None" ,  .(mean_conservation=mean(as.numeric(mean_conservations_vertebrates)))  , by=c("ME_clusters", "ME_cluster.name")]


ME_cluster_names <-  merge(ME_cluster_names, ME_cluster.conservation,  by.x=c("ME_cluster", "ME_cluster.name"), by.y=c("ME_clusters", "ME_cluster.name"))

round(cor(ME_cluster_names$symetrical_fraction, ME_cluster_names$mean_conservation, method="pearson"), 2)


ggplot(ME_cluster_names, aes(x=symetrical_fraction, y=mean_conservation)) +
  geom_smooth(method=lm, linetype="dashed") +
  geom_text(aes( label = ME_cluster.name, colour = ME_cluster.type ) ) +
  xlab("In-frame microexon fraction") +
  ylab("Mean Phylop conservation score") +
  theme(legend.position="top") +
 labs(color = "Microexon cluster class")
```


```
some_ME_clusters <-c(
ME_clusters[ME_clusters==1][1],
ME_clusters[ME_clusters==2][1],
ME_clusters[ME_clusters==3][1],
ME_clusters[ME_clusters==4][1],
ME_clusters[ME_clusters==5][1],
ME_clusters[ME_clusters==6][1],
ME_clusters[ME_clusters==7][1],
ME_clusters[ME_clusters==8][1],
ME_clusters[ME_clusters==9][1],
ME_clusters[ME_clusters==10][1],
ME_clusters[ME_clusters==11][1],
ME_clusters[ME_clusters==12][1],
ME_clusters[ME_clusters==13][1],
ME_clusters[ME_clusters==14][1],
ME_clusters[ME_clusters==15][1],
ME_clusters[ME_clusters==16][1],
ME_clusters[ME_clusters==17][1],
ME_clusters[ME_clusters==18][1],
ME_clusters[ME_clusters==19][1],
ME_clusters[ME_clusters==20][1],
ME_clusters[ME_clusters==21][1],
ME_clusters[ME_clusters==22][1],
ME_clusters[ME_clusters==23][1],
ME_clusters[ME_clusters==24][1]

)
```


```
col_ann <- data.frame(  Tissue_clusters$cluster, row.names=Tissue_clusters$File.accession)
colnames(col_ann) <- "Tissue clusters"

rownames(col_ann) <- Tissue_clusters$File.accession


tissue_cluster_type = data.frame(cluster=c(1, 6, 8, 14, 12, 7, 15, 5, 11, 4, 9, 3, 13, 10, 16, 2), 
                                 `Tissue cluster type`=c("Non-neuronal",
                                                "Non-neuronal",
                                                "Non-neuronal",
                                                "Non-neuronal",
                                                "Embryonic neuronal",
                                                "Adrenal gland",
                                                "Skeletal muscle",
                                                "Heart",
                                                "Embryonic neuronal",
                                                "Embryonic neuronal",
                                                "Embryonic neuronal",
                                                "Postnatal neuronal",
                                                "Embryonic neuronal",
                                                "Postnatal neuronal",
                                                "Postnatal neuronal",
                                                "Postnatal neuronal"))

col_ann$File <- rownames(col_ann)
col_ann <- merge(col_ann, tissue_cluster_type, by.x="Tissue clusters",by.y="cluster")
rownames(col_ann) <- col_ann$File

colnames(col_ann) <- c("Tissue clusters", "File", "Tissue cluster type")


ann_colors = list(
    cluster = c(black="black"),
    `Tissue.cluster.type` =  c(`Adrenal gland` = "#B79F00",
                      `Skeletal muscle` = "#00BFC4",
                      `Heart` = "#F564E3",
                      `Non-neuronal` = "#A58AFF",
                      `Embryonic neuronal` = "#00BA38",
                      `Postnatal neuronal` = "#F8766D"
                      ),
    GeneClass = c(Path1 = "#7570B3", Path2 = "#E7298A", Path3 = "#66A61E")
)

col_ann$`Tissue cluster type` <- factor(col_ann$`Tissue cluster type` , levels = c("Adrenal gland", "Skeletal muscle", "Heart", "Non-neuronal", "Intermediate", "Embryonic neuronal", "Postnatal neuronal"))

fake_plot <- ggplot(col_ann, aes(as.numeric(`Tissue clusters`), `Tissue clusters`, fill=`Tissue cluster type` )) +
  geom_bar(stat = "identity") +
  scale_fill_manual(values=c("#B79F00", "#00BFC4", "#F564E3", "#A58AFF", "#00BA38", "#F8766D" ), name= "Tissue cluster type") +
   theme(legend.position="top", legend.justification="center") +
  guides(fill=guide_legend(nrow=1,byrow=TRUE)) 


fake_plot.legend <- get_legend(fake_plot)
```


```
library(rlang)


#rownames(col_ann) <- rownames(Tissue_clusters)

Tissue_PSI_matrix_dcast_ppca <- t(cObs)

Tissue_PSI_matrix_dcast_ppca[Tissue_PSI_matrix_dcast_ppca>1] <- 1
Tissue_PSI_matrix_dcast_ppca[Tissue_PSI_matrix_dcast_ppca<0] <- 0


Tissue_PSI_matrix_dcast_ppca_heatmap <- duplicate(Tissue_PSI_matrix_dcast_ppca)


some_ME_clusters_space <- paste("      ", some_ME_clusters, sep = "") 
names(some_ME_clusters_space) <- names(some_ME_clusters)

row.names(Tissue_PSI_matrix_dcast_ppca_heatmap) <- some_ME_clusters_space[row.names(Tissue_PSI_matrix_dcast_ppca_heatmap)]


#tissue_heatmap <- pheatmap::pheatmap(Tissue_PSI_matrix_dcast_ppca_heatmap,  , fontsize = 10 ,  cutree_rows = 24, cutree_cols = 16, clustering_method = "ward.D2" , show_colnames=FALSE, annotation_col= col_ann, annotation_names_col=FALSE, treeheight_col=200, treeheight_row=100)
```


```
plot_grid(fake_plot.legend)
```


```
PCA <- data.frame(scores(result))


PCA$File.accession <- row.names(PCA)
PCA <- data.table(PCA)
PCA <- PCA[, `:=`(PC1=-PC1) ]


PCA <- merge(Tissue_clusters, PCA, by="File.accession")

PCA$age <- as.numeric(as.character(PCA$age))
```


```
PCA_stats <- PCA[ , .(mean_PC1=mean(PC1)) , by=c("name", "age")]


Sup_PCA_age.B <- ggplot() +
  geom_jitter(data = PCA[name %in% c("forebrain", "midbrain", "hindbrain", "neural tube"), ],  aes(age, PC1, colour=name), width=0.1, height = 0 ) +
  geom_line(data = PCA_stats[name %in% c("forebrain", "midbrain", "hindbrain", "neural tube"), ], aes(age, mean_PC1, group=name, colour=name)) +
  labs(colour="Neural tissue")
```


```
PCA_stats<- PCA[ , .(mean_PC1=mean(PC1)) , by=c("name", "age")]

Fig4.C <- ggplot() +
  geom_line(data = PCA_stats[name %in% c("forebrain", "midbrain", "hindbrain", "neural tube", "whole cortex"), ], aes(log(age), mean_PC1, group=name, colour=name)) +
  geom_point(data = PCA_stats[name %in% c("forebrain", "midbrain", "hindbrain", "neural tube", "whole cortex"), ], aes(log(age), mean_PC1, group=name, colour=name)) +
  xlab("log(DPC)") +
  ylab("Mean PC1") +
  xlim(c(log(10), log(50))) +
  theme(legend.position="top") +
  scale_color_discrete(name="Neuronal tissue", labels=c("Forebrain", "Hindbrain", "Midbrain", "Neural tube", "Whole cortex"))
  

Fig4.C
```


```
PCA_stats_PC2<- PCA[ name=="heart" , .(mean_PC2=mean(PC2)) , by=c("name", "age")]
```


```
PC1_cluster <- PCA[ , .(PC1=mean(PC1))  , by=cluster]

tissue_cluster_PCA <- factor(PC1_cluster[order(PC1)]$cluster, levels = PC1_cluster[order(PC1)]$cluster)


my_col <- get_colors(tissue_cluster_PCA, group.col=colorRampPalette(colors = c("black",  "purple", "blue", "seagreen", "gold", "red"))(14))
```


```
PCA$cluster <- factor(PCA$cluster, levels = PC1_cluster[order(PC1)]$cluster)


Sup_PCA.A <- ggplot(PCA) +
  geom_text(aes(PC1, PC2, label=cluster, colour=cluster) ) +
  scale_color_manual(values=my_col) + 
  theme(legend.position = "none")

Sup_PCA.B <-  ggplot(PCA) +
  geom_text(aes(PC1, PC3, label=cluster, colour=cluster) ) +
  scale_color_manual(values=my_col) + 
  theme(legend.position = "none")

Sup_PCA.C <-  ggplot(PCA) +
  geom_text(aes(PC2, PC3, label=cluster, colour=cluster) ) +
  scale_color_manual(values=my_col) + 
  theme(legend.position = "none")
```


```
library(rlang)

PCA_plot <- duplicate(PCA)


PCA_plot[name=="heart", group:="Heart"]
PCA_plot[name=="skeletal muscle tissue", group:="SKM"]
PCA_plot[name=="adrenal gland", group:="Adrenal Gland"]
PCA_plot[name=="whole cortex", group:="Cortex"]
PCA_plot[name %in% c("forebrain", "midbrain", "hindbrain", "neural tube") , group:="Neural"]

PCA_plot$age <- as.numeric(as.character(PCA_plot$age ))


PCA_plot[!name %in% c("forebrain", "midbrain", "hindbrain", "neural tube", "whole cortex", "heart","skeletal muscle tissue", "adrenal gland"), `:=`(group="Other", age=NA )]

ggplot(PCA_plot[name!="whole cortex",]) +
  geom_point(aes(PC1, PC2, colour=age, shape=group ) ) +
  geom_point(aes(PC1, PC2, colour=age, shape=group ) ) +
  scale_color_gradient(low="blue",  high="red")
```


```
library(stringr)

Sup_PCA_age.A <- ggplot() +
  geom_point(data=PCA_plot[group=="Other"], aes(PC1, PC2, colour=age ), alpha=0.5) +
  geom_text(data=PCA_plot[group!="Other" & name!="whole cortex"], aes(PC1, PC2, colour=age, label=str_sub(group, 1,1) ), size=3.5, alpha=0.5 ) +
  scale_color_gradient(low="blue",  high="red") +
  labs(colour="Embryonic stage (DPC)")
```


```
ggplot() +
  geom_point(data=PCA_plot[group=="Other"], aes(PC1, PC2, colour=log(age) ), alpha=0.8) +
  geom_text(data=PCA_plot[group!="Other"], aes(PC1, PC2, colour=log(age), label=str_sub(group, 1,1) ), size=3.5, alpha=0.8 ) +
  scale_color_gradient2(low="blue", mid="gold",  high="red", midpoint = 3.7)
```


```
plot_grid(Sup_PCA_age.A, Sup_PCA_age.B, labels = "AUTO")
```


```
PCA_batch <- merge(PCA, PCA[, .N, by=c("date")][, .(date, batch=frank(date) )], by="date")

my_col_date <- get_colors(tissue_cluster_PCA, group.col=colorRampPalette(colors = c("black",  "purple", "blue", "seagreen", "gold", "red"))(10))


Sup_PCA.D <-  ggplot(PCA_plot) +
  geom_text(aes(PC1, PC2, label=str_sub(group, 1,1) , colour=factor(cluster) ) ) +
  scale_color_manual(values=my_col) + 
  theme(legend.position = "none")


Sup_PCA.E <-  ggplot(PCA_plot) +
  geom_text(aes(PC1, PC3, label=str_sub(group, 1,1) , colour=factor(cluster) ) ) +
  scale_color_manual(values=my_col) + 
  theme(legend.position = "none")

Sup_PCA.F <-  ggplot(PCA_plot) +
  geom_text(aes(PC2, PC3, label=str_sub(group, 1,1) , colour=factor(cluster) ) ) +
  scale_color_manual(values=my_col) + 
  theme(legend.position = "none")


Sup_PCA.G <-  ggplot(PCA_batch) +
  geom_text(aes(PC1, PC2, label=cluster, colour=factor(batch) ) ) +
  scale_color_manual(values=my_col_date) + 
  theme(legend.position = "none")


Sup_PCA.H <-  ggplot(PCA_batch) +
  geom_text(aes(PC1, PC3, label=cluster, colour=factor(batch) ) ) +
  scale_color_manual(values=my_col_date) + 
  theme(legend.position = "none")


Sup_PCA.I <-  ggplot(PCA_batch) +
  geom_text(aes(PC2, PC3, label=cluster, colour=factor(batch) ) ) +
  scale_color_manual(values=my_col_date) + 
  theme(legend.position = "none")
```


```
plot_grid(Sup_PCA.A, Sup_PCA.B, Sup_PCA.C, Sup_PCA.D, Sup_PCA.E, Sup_PCA.F, Sup_PCA.G, Sup_PCA.H, Sup_PCA.I, labels="AUTO" )
```


```
#Tissue_clusters <- cutree(hc_cols, k = 18)
#Tissue_clusters <- cbind(Tissue_clusters, Tissues_name[names(Tissue_clusters)], Tissues_age[names(Tissue_clusters)], Tissue_date[names(Tissue_clusters)]) 

#colnames(Tissue_clusters) <- c("cluster", "name", "age", "date")

#Tissue_clusters <- data.frame(Tissue_clusters)

#Tissue_clusters_sum[which(grepl("hindbrain|midbrain|forebrain|neural\ tube", Tissue_clusters_sum$name)), ]

#Tissue_clusters$name <- factor(Tissue_clusters$name, level=c( "skeletal muscle tissue", "heart", "thymus", "spleen", "liver",  "adrenal gland", "intestine", "stomach", "lung",  "kidney", "bladder", "limb", "embryonic facial prominence", "forebrain", "hindbrain", "midbrain", "neural tube", "whole cortex"))


#Tissue_clusters$age <- factor(Tissue_clusters$age, levels = as.character(sort(as.numeric(levels(Tissue_clusters$age)))))
```


```
#Tissue_clusters_sum <- Tissue_clusters %>% group_by(cluster, name, age ) %>% summarise(count = n())

Tissue_clusters_DT <- data.table(Tissue_clusters)
Tissue_clusters_sum <- Tissue_clusters_DT[, .(count=.N) , by=c("cluster", "name", "age" )]

Tissue_clusters_sum$age <- factor(Tissue_clusters_sum$age, levels= sort(as.numeric(as.character(levels(Tissue_clusters_sum$age)))) )


## NA ###

Tissue_clusters_sum$cluster <- factor(Tissue_clusters_sum$cluster , levels =PCA[, mean(PC1), by="cluster" ][order(V1)]$cluster)


Tissue_clusters_sum <- merge(Tissue_clusters_sum, PCA[, .(mPC1=mean(PC1), mPC2=mean(PC2), mPC3=mean(PC3) ), by="cluster" ][order(mPC1)], by="cluster")
Tissue_clusters_sum[, `:=`(mean_PC1=round(mPC1, 2),mean_PC2=round(mPC2, 2), mean_PC3=round(mPC3, 2))  ]

Sup.Tissue_clusters.b.pre <- ggplot( Tissue_clusters_sum, aes(age, name) ) +
  geom_tile(aes(fill = count)) +
  scale_fill_gradient(low="grey", high="red")+
  facet_grid(. ~ cluster + mean_PC1 + mean_PC2 + mean_PC3, labeller = label_parsed ) +
  theme_bw()+
  labs(fill = "Sample count") +
  theme(axis.text.x = element_text(angle = 90, vjust = 0),
    axis.title = element_text(colour = NA),
    strip.text = element_text(hjust = 0.5))


Sup.Tissue_clusters.c <- plot_grid(fake_plot.legend, Sup.Tissue_clusters.b.pre, ncol=1, rel_heights = c(0.1, 1) )
Sup.Tissue_clusters.c
```


```
library(ggrepel)

PCA_plot[, `:=`(mPC1=mean(PC1), mPC2=mean(PC2)), by="cluster" ]


Sup.Tissue_clusters.a <- ggplot(PCA_plot) +
  geom_point(aes(PC1, PC2 , colour=factor(cluster) ),  alpha=0.3 ) +
  stat_ellipse(aes(PC1, PC2 , colour=factor(cluster) )) +
  geom_text_repel(data=PCA_plot[, .(mPC1=mean(PC1), mPC2=mean(PC2)), by="cluster" ],
                  aes(mPC1, mPC2, label=cluster), size=7, point.padding = NA, fontface = "bold" ) +
  scale_color_manual(values=my_col) + 
  theme(legend.position = "none") 

Sup.Tissue_clusters.b <- ggplot(PCA_plot) +
  geom_point(aes(PC1, PC3 , colour=factor(cluster) ),  alpha=0.3 ) +
  stat_ellipse(aes(PC1, PC3 , colour=factor(cluster) )) +
  geom_text_repel(data=PCA_plot[, .(mPC1=mean(PC1), mPC2=mean(PC3)), by="cluster" ],
                  aes(mPC1, mPC2, label=cluster), size=7, point.padding = NA, fontface = "bold" ) +
  scale_color_manual(values=my_col) + 
  theme(legend.position = "none") 
  

Sup.Tissue_clusters.ab <- plot_grid(Sup.Tissue_clusters.a, Sup.Tissue_clusters.b, labels = c("E", "F"))
Sup.Tissue_clusters.ab
```


```
Fig4.EFG <- plot_grid( Sup.Tissue_clusters.ab, Sup.Tissue_clusters.c, ncol=1, labels=c("", "G"), rel_heights = c(0.8, 1) )
Fig4.EFG
```


```
Fig.4A <- ggplot(PCA_plot) +
  geom_point(aes(PC1, PC2, colour=log(age), shape=group ), size=3 , alpha=0.7) +
  scale_color_gradient2(low="blue", mid="yellow",  high="red", midpoint = 3.7, name="ln(Age)") +
  scale_shape(name="Tissue") +
  theme(legend.position="top")
```


```
plot_grid(Fig4, Fig4.EFG, ncol=1, rel_heights = c(2, 1))
```


Here we sorted microexons by PC1 loadings, but we fliped PC1 we cocider the values as -1 PCA loading on our paper


Loading therhold is abs(0.035)


```
ME_Tissues_clusters_PSI <- merge(ME_clusters_PSI, Tissue_clusters, by.x="FILE_NAME", by.y = "File.accession")
ME_Tissues_clusters_PSI$cluster <- factor(ME_Tissues_clusters_PSI$cluster, levels = levels(Tissue_clusters_sum$cluster))
ME_Tissues_clusters_PSI <- merge( ME_Tissues_clusters_PSI,  ME_cluster_names[ , c("ME_cluster", "ME_cluster.name")], by.x="ME_clusters", by.y="ME_cluster")

ME_Tissues_clusters_PSI$ME_cluster.name <- factor(ME_Tissues_clusters_PSI$ME_cluster.name, levels = sort(as.character(unique(ME_Tissues_clusters_PSI$ME_cluster.name))))

ME_Tissues_clusters_PSI_sub <- ME_Tissues_clusters_PSI[ME_cluster.name %in%  ME_cluster_names[ME_cluster.type %in% c("Neuronal", "Neuro-Muscular", "Muscular",  "Non-Neuronal"), ME_cluster.name], ]
ME_Tissues_clusters_PSI_non_neuronal <- ME_Tissues_clusters_PSI_sub[cluster %in% c(1, 8, 6, 7, 15, 5) , ]

ME_Tissues_clusters_PSI_non_neuronal$cluster <- mapvalues(ME_Tissues_clusters_PSI_non_neuronal$cluster,
          from = c(1, 8, 6, 7, 15, 5),
          to = c( "C1", "C8", "C6", "C7", "C15", "C5") )


ME_Tissues_clusters_PSI_non_neuronal$cluster <- factor(ME_Tissues_clusters_PSI_non_neuronal$cluster, levels=c( "C1", "C8", "C6", "C7", "C15", "C5"))

ME_Tissues_clusters_PSI_non_neuronal_by_ME <- ME_Tissues_clusters_PSI_non_neuronal[ , .(PSI_mean=mean(PSI)), by=c("ME", "cluster", "ME_cluster.name") ]
ME_Tissues_clusters_PSI_non_neuronal_by_ME_cluster <- ME_Tissues_clusters_PSI_non_neuronal[ , .(PSI_mean=mean(PSI)), by=c( "cluster", "ME_cluster.name") ]


  
ggplot( ) +
        geom_line(data= ME_Tissues_clusters_PSI_non_neuronal_by_ME, aes(factor(cluster), PSI_mean, group=ME), colour="grey") +
        facet_grid( ME_cluster.name ~ .)
```


```
TOTAL.ME_level <- rbind(
ME_Tissues_clusters_PSI_non_neuronal_by_ME[ , .(ME, age.name=cluster, ME_cluster.name, PSI_mean) ], 
ME_Tissues_clusters_PSI_neuronal_by_ME )

TOTAL.cluster_level <-  rbind(
ME_Tissues_clusters_PSI_neuronal_by_ME_cluster,
ME_Tissues_clusters_PSI_non_neuronal_by_ME_cluster[ , .(age.name=cluster, ME_cluster.name,  name="Non neuronal", PSI_mean) ] )


TOTAL.cluster_level$name <- mapvalues(TOTAL.cluster_level$name,
          from = c("forebrain", "midbrain", "hindbrain", "neural tube", "whole cortex"),
          to = c("Forebrain", "Midbrain", "Hindbrain", "Neural tube", "Whole cortex") )


TOTAL.cluster_level$name <- factor(TOTAL.cluster_level$name, levels=c("Non neuronal", "Forebrain", "Midbrain", "Hindbrain", "Neural tube", "Whole cortex") )


Fig5.A <- ggplot( ) +
        geom_line(data= TOTAL.ME_level, aes(factor(age.name), PSI_mean, group=ME), colour="grey", alpha=0.5) +
        geom_line(data = TOTAL.cluster_level, aes(factor(age.name), PSI_mean, group=name, colour=name ), size=1.2 ) +
        facet_grid( ME_cluster.name ~ .) +
        labs(colour = "Mean PSI by") +
        ylab("PSI") +
        xlab("") +
        theme(panel.background = element_rect(fill = 'white', colour = 'black')) +
        theme(axis.text.x = element_text(angle = 90), legend.position = "top", legend.direction = "horizontal")

Fig5.A
```


```
ggplot( ) +
        geom_line(data= TOTAL.ME_level, aes(factor(age.name), PSI_mean, group=ME), colour="grey", alpha=0.5) +
        geom_line(data = TOTAL.cluster_level, aes(factor(age.name), PSI_mean, group=name, colour=name ), size=1.2 ) +
        facet_grid( ME_cluster.name ~ .) +
        labs(colour = "Mean PSI by") +
        ylab("PSI") +
        xlab("") +
        theme(panel.background = element_rect(fill = 'white', colour = 'black')) +
        theme(axis.text.x = element_text(angle = 90), legend.position = "top", legend.direction = "horizontal")
```


#### Whippet-delta


```
file.remove('./whippet_delta.yaml')


write.table( "whippet_delta:", file="./whippet_delta.yaml", append=TRUE, quote = FALSE, row.names = FALSE, col.names = FALSE)


wcontrol <- paste(Tissue_clusters[cluster %in% c(1, 8, 6),  ]$File.accession, collapse=",")


for (i in c("forebrain", "midbrain", "hindbrain", "neural tube")){
  
  for (a in c(10.5, 11.5, 12.5, 13.5, 14.5, 15.5, 16.5, 21)) {
    
    if (length(Tissue_clusters[name==i & age==a , ]$File.accession)>=4){
    
      name = paste0("    ", gsub(" ", "_", paste("control_vs", i, paste0(a, ":"), sep=" " )))
      A =  paste0("        A : ", wcontrol )
      B =  paste0("        B : ",  paste(Tissue_clusters[name==i & age==a , ]$File.accession, collapse=","))
      
      write.table(name, file="./whippet_delta.yaml", append=TRUE, quote = FALSE, row.names = FALSE, col.names = FALSE)
      write.table( A, file="./whippet_delta.yaml", append=TRUE, quote = FALSE, row.names = FALSE, col.names = FALSE)
      write.table( B, file="./whippet_delta.yaml", append=TRUE, quote = FALSE, row.names = FALSE, col.names = FALSE)
    
    }

  }
  
}


signal = c("hindbrain", "neural tube")

for (a in c(10.5, 11.5, 12.5, 13.5, 14.5, 15.5, 16.5, 21)) {
    
  if (length(Tissue_clusters[name %in% signal & age==a , ]$File.accession)>=4){
    
    name = paste0("    ", gsub(" ", "_", paste("control_vs", paste(signal, collapse="-" ), paste0(a, ":"), sep=" " )))
    A =  paste0("        A : ", wcontrol )
    B =  paste0("        B : ",  paste(Tissue_clusters[name %in% signal & age==a , ]$File.accession, collapse=","))
      
    write.table(name, file="./whippet_delta.yaml", append=TRUE, quote = FALSE, row.names = FALSE, col.names = FALSE)
    write.table( A, file="./whippet_delta.yaml", append=TRUE, quote = FALSE, row.names = FALSE, col.names = FALSE)
    write.table( B, file="./whippet_delta.yaml", append=TRUE, quote = FALSE, row.names = FALSE, col.names = FALSE)

  }
}
 

signal = c("hindbrain", "neural tube", "midbrain")

for (a in c(10.5, 11.5, 12.5, 13.5, 14.5, 15.5, 16.5, 21)) {
    
  if (length(Tissue_clusters[name %in% signal & age==a , ]$File.accession)>=4){
    
    name = paste0("    ", gsub(" ", "_", paste("control_vs", paste(signal, collapse="-" ), paste0(a, ":"), sep=" " )))
    A =  paste0("        A : ", wcontrol )
    B =  paste0("        B : ",  paste(Tissue_clusters[name %in% signal & age==a , ]$File.accession, collapse=","))
      
    write.table(name, file="./whippet_delta.yaml", append=TRUE, quote = FALSE, row.names = FALSE, col.names = FALSE)
    write.table( A, file="./whippet_delta.yaml", append=TRUE, quote = FALSE, row.names = FALSE, col.names = FALSE)
    write.table( B, file="./whippet_delta.yaml", append=TRUE, quote = FALSE, row.names = FALSE, col.names = FALSE)

  }
}


signal = c("hindbrain", "neural tube", "midbrain","forebrain")

for (a in c(10.5, 11.5, 12.5, 13.5, 14.5, 15.5, 16.5, 21)) {
    
  if (length(Tissue_clusters[name %in% signal & age==a , ]$File.accession)>=4){
    
    name = paste0("    ", gsub(" ", "_", paste("control_vs", paste(signal, collapse="-" ), paste0(a, ":"), sep=" " )))
    A =  paste0("        A : ", wcontrol )
    B =  paste0("        B : ",  paste(Tissue_clusters[name %in% signal & age==a , ]$File.accession, collapse=","))
      
    write.table(name, file="./whippet_delta.yaml", append=TRUE, quote = FALSE, row.names = FALSE, col.names = FALSE)
    write.table( A, file="./whippet_delta.yaml", append=TRUE, quote = FALSE, row.names = FALSE, col.names = FALSE)
    write.table( B, file="./whippet_delta.yaml", append=TRUE, quote = FALSE, row.names = FALSE, col.names = FALSE)

  }
}


signal = c("adrenal gland", "skeletal muscle tissue", "heart")

for (tissue in signal) {
    
  if (length(Tissue_clusters[name==tissue, ]$File.accession)>=4){
    
    name = paste0("    ", gsub(" ", "_", paste("control_vs", paste0(tissue, ":"), sep=" " )))
    A =  paste0("        A : ", wcontrol )
    B =  paste0("        B : ",  paste(Tissue_clusters[name==tissue, ]$File.accession, collapse=","))
      
    write.table(name, file="./whippet_delta.yaml", append=TRUE, quote = FALSE, row.names = FALSE, col.names = FALSE)
    write.table( A, file="./whippet_delta.yaml", append=TRUE, quote = FALSE, row.names = FALSE, col.names = FALSE)
    write.table( B, file="./whippet_delta.yaml", append=TRUE, quote = FALSE, row.names = FALSE, col.names = FALSE)

  }
}


name= "    hindbrain_neural_tube_21_vs_forebrain_midbrain_21:"
A = paste0("        A : ",  paste(Tissue_clusters[age==21 & name %in% c("hindbrain", "neural tube"), ]$File.accession, collapse=","))
B = paste0("        B : ",  paste(Tissue_clusters[age==21 & name %in% c("forebrain", "midbrain"), ]$File.accession, collapse=","))

write.table(name, file="./whippet_delta.yaml", append=TRUE, quote = FALSE, row.names = FALSE, col.names = FALSE)
write.table( A, file="./whippet_delta.yaml", append=TRUE, quote = FALSE, row.names = FALSE, col.names = FALSE)
write.table( B, file="./whippet_delta.yaml", append=TRUE, quote = FALSE, row.names = FALSE, col.names = FALSE)
```

#### Whippet ENCODE


```
Get_delta_table <- function( signal, ages, path, extension ){

  Delta_group <- data.table()
  
  
  for (a in ages) {
      
    if (length(Tissue_clusters[name %in% signal & age==a , ]$File.accession)>=4){
      
      name = gsub(" ", "_", paste("control_vs", paste(signal, collapse="-" ), a, sep=" " ))
  
      delta <- fread ( paste0(path, name, extension))
      
      delta$age <- a
      
      Delta_group <- rbind(Delta_group, delta )
      
    }
  
  }
  return(Delta_group)    
}
```


```
length(unique(Delta_HNM_merge[abs(DeltaPsi.x)>=0.1 & Probability.x>0.9 & abs(DeltaPsi.y)>=0.1 & Probability.y>0.9, exon_ID]))
```


```
[1] 421
```


```
Delta_F_whippet <- Get_delta_table( c("forebrain"),
                                      c(10.5, 11.5, 12.5, 13.5, 14.5, 15.5, 16.5, 21),
                                      "~/Google_Drive/Results/ME/Paper/Final_Report/Reps/Rep1/Whippet/",
                                      ".diff.microexons")


Delta_F_ME <- Get_delta_table( c("forebrain"),
                                      c(10.5, 11.5, 12.5, 13.5, 14.5, 15.5, 16.5, 21),
                                      "~/Google_Drive/Results/ME/Paper/Final_Report/Reps/Rep1/Whippet/",
                                      ".diff.ME.microexons")


Delta_F_merge <- merge(Delta_F_whippet, Delta_F_ME, by=c("exon_ID", "age"))

length(unique(Delta_F_merge[abs(DeltaPsi.x)>=0.1 & Probability.x>0.9 & abs(DeltaPsi.y)>=0.1 & Probability.y>0.9, exon_ID]))
```


```
Sup_vulcano.B <- ggplot( ) +
  geom_point(data=Delta_HNM_ME, aes(-DeltaPsi, Probability)) +
  geom_point(data=Delta_HNM_ME[ DeltaPsi>=0.1 &  Probability >= 0.9 , ], aes(-DeltaPsi, Probability), color="brown") +
  geom_point(data=Delta_HNM_ME[ DeltaPsi<(-0.1) &  Probability >= 0.9 , ], aes(-DeltaPsi, Probability), color="darkgreen") +
  geom_text(data=Delta_HNM_ME[ DeltaPsi>=0.1 &  Probability >= 0.9 , .N, by="age"], aes(-0.85, 0.9, label=N), colour = "brown", fontface = "bold", size=5) +
  geom_text(data=Delta_HNM_ME[ DeltaPsi<(-0.1) &  Probability >= 0.9  , .N, by="age"], aes(0.85, 0.9, label=N), colour = "darkgreen", fontface = "bold", size=5) +
  facet_grid(. ~ age) +
  xlim(c(-1,1)) +
  xlab("Delta PSI") +
  theme_bw()


Sup_vulcano.A <- ggplot( ) +
  geom_point(data=Delta_HNM_whippet, aes(-DeltaPsi, Probability)) +
  geom_point(data=Delta_HNM_whippet[ DeltaPsi>=0.1 &  Probability >= 0.9 , ], aes(-DeltaPsi, Probability), color="brown") +
  geom_point(data=Delta_HNM_whippet[ DeltaPsi<(-0.1) &  Probability >= 0.9 , ], aes(-DeltaPsi, Probability), color="darkgreen") +
  geom_text(data=Delta_HNM_whippet[ DeltaPsi>=0.1 &  Probability >= 0.9 , .N, by="age"], aes(-0.85, 0.9, label=N), colour = "brown", fontface = "bold", size=5) +
  geom_text(data=Delta_HNM_whippet[ DeltaPsi<(-0.1) &  Probability >= 0.9  , .N, by="age"], aes(0.85, 0.9, label=N), colour = "darkgreen", fontface = "bold", size=5) +  
  facet_grid(. ~ age) +
  xlim(c(-1,1)) +
  xlab("Delta PSI") +
  theme_bw()
```

### whipet vs ME


```
ME_AD_AA <- fread("../Final_Report/Reps/Rep1/Whippet/Quant/ME_AD_AA.txt")

Delta_HNM_total <-  merge(
Delta_HNM_whippet[ , c("exon_ID", "Gene", "Node", "Coord", "Strand", "Type", "DeltaPsi", "Probability", "age")],
Delta_HNM_ME[ , c("exon_ID", "Gene", "Node", "Coord", "Strand", "Type", "DeltaPsi", "Probability", "age")],

by=c("exon_ID", "Gene", "Node", "Coord", "Strand", "Type",  "age"))

Delta_HNM_total[ , cor(DeltaPsi.x, DeltaPsi.y, method="pearson") , by=age ]

Delta_HNM_total[paste( exon_ID, age, sep="|") %in% total_diff_HNM , cor(DeltaPsi.x, DeltaPsi.y, method="pearson") , by=age ]


Delta_HNM_total[, Node_type:=paste0("m", Type)]
Delta_HNM_total[ Type=="CE" & exon_ID %in% ME_AD_AA[ Type=="AA" & exon_type=="E" ,  exon_ID  ], Node_type:="mCE_AA" ]
Delta_HNM_total[ Type=="CE" & exon_ID %in% ME_AD_AA[ Type=="AA" & exon_type=="ME" ,  exon_ID  ], Node_type:="mCE_mAA" ]
Delta_HNM_total[ Type=="CE" & exon_ID %in% ME_AD_AA[ Type=="AD" & exon_type=="E" ,  exon_ID  ], Node_type:="mCE_AD" ]
Delta_HNM_total[ Type=="CE" & exon_ID %in% ME_AD_AA[ Type=="AD" & exon_type=="ME" ,  exon_ID  ], Node_type:="mCE_mAD" ]

whippet_ME_corr <- merge( Delta_HNM_total[ , cor(DeltaPsi.x, DeltaPsi.y, method="pearson") , by=age ], 
                          Delta_HNM_total[ (abs(DeltaPsi.x)>=0.1 & Probability.x>=0.9) |(abs(DeltaPsi.y)>=0.1 & Probability.y>=0.9)  ,
                                           cor(DeltaPsi.x, DeltaPsi.y, method="pearson") , by=age ], 
                          by="age")

#whippet_ME_corr.types <- merge( Delta_HNM_total[ , .(N=.N, cor.total=(cor(DeltaPsi.x, DeltaPsi.y, method="pearson"))) , by=c("age", "Node_type") ], 
#                          Delta_HNM_total[paste( exon_ID, age, sep="|") %in% total_diff_HNM , .(N=.N, cor.sig=(cor(DeltaPsi.x, DeltaPsi.y, method="pearson"))) , by=c("age", #"Node_type") ],
#                          by=c("age", "Node_type"))


whippet_ME_corr.types <- Delta_HNM_total[ , .(N=.N, cor.total=(cor(DeltaPsi.x, DeltaPsi.y, method="pearson"))) , by=c("age", "Node_type") ]


#whippet_ME_corr.types[N.y<5, cor.sig:=NA]

#whippet_ME_corr.types.melted <- rbind(whippet_ME_corr.types[, .(age, Node_type, N=N.x, cor=cor.total, Group="Total") ],
 #     whippet_ME_corr.types[, .(age, Node_type, N=N.y, cor=cor.sig, Group="Significant") ])

Delta_HNM_total$Node_type <- factor(Delta_HNM_total$Node_type , levels = c("mCE", "mAA", "mAD", "mCE_mAA", "mCE_mAD", "mCE_AA", "mCE_AD"))

whippet_ME_corr.types$Node_type <- factor(whippet_ME_corr.types$Node_type , levels = c("mCE", "mAA", "mAD", "mCE_mAA", "mCE_mAD", "mCE_AA", "mCE_AD"))


Sup_vulcano.D <- ggplot(whippet_ME_corr.types[N>5, ]) +
  geom_boxplot(aes(Node_type, cor.total, fill=Node_type)) +
  scale_fill_discrete(name = "Microexon node type") +
  xlab("") +
  ylab("Delta PSI correlation") +
  theme_bw() +
  theme(legend.position = "top") +
  theme(axis.text.x = element_text(angle = 45, vjust = 0.6)) +
     guides(fill = guide_legend(nrow = 1))
```


```
Sup_vulcano.C <- ggplot(Delta_HNM_total[!is.na(Node_type)] ) +
  geom_bar(aes(as.factor(age), fill=Node_type), stat="count" ) +
  geom_text( data=Delta_HNM_total[ , .(N=.N, cor=cor(DeltaPsi.x, DeltaPsi.y, method="pearson")) , by=age ],
             aes(x=as.factor(age), y=N+200, label= paste0("R=", round(cor, 2)))) +
  xlab("DPC") +
  ylab("Number of splicing nodes") +
    scale_fill_discrete(name = "Microexon node type") +
  theme_bw() +
  theme(legend.position = "top") +
  guides(fill = guide_legend(nrow = 1))

Sup_vulcano.legend <- get_legend(Sup_vulcano.C)
```


```
library(ggExtra)

ME_AD_AA

Delta_HNM_total.alt_ME <- merge(Delta_HNM_total[ , .(exon_ID, Node_type, age, 
                                                     DeltaPsi.wp=DeltaPsi.x, Probability.wp=Probability.x, 
                                                     DeltaPsi.me=DeltaPsi.y, Probability.me=Probability.y)],
                                ME_AD_AA[ ,c("exon_ID", "exon_ID.alt")], by=c("exon_ID"))

Delta_HNM_total.alt_ME.compare <- merge(Delta_HNM_total.alt_ME,
                                        Delta_HNM_total[ , .(exon_ID, Node_type, age, 
                                                             DeltaPsi.wp.alt=DeltaPsi.x, Probability.wp.alt=Probability.x, 
                                                             DeltaPsi.me.alt=DeltaPsi.y, Probability.me.alt=Probability.y)],
                                        by.x=c("exon_ID.alt", "age"), by.y=c("exon_ID", "age") )


Delta_HNM_total.alt_ME.compare.stats <- Delta_HNM_total.alt_ME.compare[, .( alt_cor.wp=cor(DeltaPsi.wp, DeltaPsi.wp.alt), alt_cor.me=cor(DeltaPsi.me, DeltaPsi.me.alt) ), by=c("exon_ID.alt", "Node_type.x") ]


p <- ggplot(Delta_HNM_total.alt_ME.compare.stats)+
  geom_point(aes(x=alt_cor.wp, y=alt_cor.me), alpha=0.4)+
  theme_bw() +
  geom_density_2d(aes(x=alt_cor.wp, y=alt_cor.me), alpha=0.4, n=500, adjust=1.3) +
  geom_rug() +
  xlab("Whippet short/long microexon correlation") +
  ylab("MicroExonator short/long microexon correlation")


Sup_vulcano.E <- ggMarginal(p, type = "histogram", fill="transparent")
```


```
Sup_vulcano.CD <- plot_grid(Sup_vulcano.C  + theme(legend.position = "NA"),
                                Sup_vulcano.D  + theme(legend.position = "NA"),
                                nrow=1, labels = c("C", "D") )


Sup_vulcano.CD <- plot_grid(Sup_vulcano.legend, Sup_vulcano.CD, ncol=1, rel_heights = c(1, 8) )


Sup_vulcano.bottom <-plot_grid(Sup_vulcano.CD, plot_grid(nullGrob(), Sup_vulcano.E, nullGrob(),  nrow=1, rel_widths = c(1,3,1), labels = c("", "E", "") ), ncol=1)

Sup_vulcano.bottom <-plot_grid(Sup_vulcano.CD, Sup_vulcano.E, labels = c( "", "E"), nrow=1, rel_widths = c(2, 1))


plot_grid(Sup_vulcano.A, Sup_vulcano.B, Sup_vulcano.bottom,  ncol=1, labels = c("A", "B", ""), rel_heights = c(1,1,3))
```


```
library(gridExtra)
library(grid)

plot_grid(Sup_vulcano.CD, plot_grid(nullGrob(), Sup_vulcano.E, nullGrob(),  nrow=1, rel_widths = c(1,3,1), labels = c("", "E", "") ), ncol=1)
```

### 


```
Delta_HNM_whippet_ME <- merge(Delta_HNM_whippet, Delta_HNM_ME, by=c("exon_ID", "Gene", "Node",  "Coord", "Strand", "Type", "age") )

Delta_HNM_whippet_ME[ (abs(DeltaPsi.x)>=0.1 &  Probability.x >= 0.9) &  (abs(DeltaPsi.y)>=0.1 &  Probability.y >= 0.9) , .N , by=(age)]

Delta_HNM_whippet_ME_included <- Delta_HNM_whippet_ME[ (DeltaPsi.x<=-0.1 &  Probability.x >= 0.9) &  (DeltaPsi.y<=-0.1 &  Probability.y >= 0.9)  , .N , by=(age)]
Delta_HNM_whippet_ME_excluded <- Delta_HNM_whippet_ME[ (DeltaPsi.x>=0.1 &  Probability.x >= 0.9) &  (DeltaPsi.y>=0.1 &  Probability.y >= 0.9)  , .N , by=(age)]


Delta_HNM_included_stats <- cbind(Delta_HNM_whippet_included[order(age)], Delta_HNM_ME_included[order(age), 2], Delta_HNM_whippet_ME_included[order(age), 2])
colnames(Delta_HNM_included_stats) <- c("age", "Whippet", "MicroExonator", "Both")
```


```
whippet_ME_age_stats <- function(Delta_S_whippet, Delta_S_ME) { 


  Delta_S_whippet_ME <- merge(Delta_S_whippet, Delta_S_ME, by=c("exon_ID", "Gene", "Node",  "Coord", "Strand", "Type", "age") )
  
  Delta_S_whippet_ME[ (abs(DeltaPsi.x)>=0.1 &  Probability.x >= 0.9) &  (abs(DeltaPsi.y)>=0.1 &  Probability.y >= 0.9) , .N , by=(age)]
  
  Delta_S_whippet_ME_included <- Delta_S_whippet_ME[ (DeltaPsi.x<=-0.1 &  Probability.x >= 0.9) &  (DeltaPsi.y<=-0.1 &  Probability.y >= 0.9)  , .N , by=(age)]
  Delta_S_whippet_ME_excluded <- Delta_S_whippet_ME[ (DeltaPsi.x>=0.1 &  Probability.x >= 0.9) &  (DeltaPsi.y>=0.1 &  Probability.y >= 0.9)  , .N , by=(age)]
  
  
  #Delta_S_included_stats <- cbind(Delta_S_whippet_included[order(age)], Delta_S_ME_included[order(age), 2], Delta_S_whippet_ME_included[order(age), 2])
  #colnames(Delta_S_included_stats) <- c("age", "Whippet", "MicroExonator", "Both")
  
  
  
  Delta_S_whippet_ME[, diff_high:=FALSE]
  Delta_S_whippet_ME[ (abs(DeltaPsi.x)>=0.1 &  Probability.x >= 0.9) &  (abs(DeltaPsi.y)>=0.1 &  Probability.y >= 0.9) , diff_high:=TRUE]
  
  Delta_S_whippet_ME[, diff_low:=FALSE]
  Delta_S_whippet_ME[ (abs(DeltaPsi.x)>=0.1 &  Probability.x >= 0.8) &  (abs(DeltaPsi.y)>=0.1 &  Probability.y >= 0.8) , diff_low:=TRUE]
  
  
  ages <- sort(unique(Delta_S_whippet_ME$age))
  age_int <- c(ages[length(ages)])
  
  
    
  for (a in rev(ages[1:length(ages)-1])){
    
    age_int = cbind(age_int, a)
    
    Delta_S_whippet_ME[ age==a & diff_high==TRUE &  exon_ID %in% Delta_S_whippet_ME[age %in% age_int & diff_high==TRUE, .N, by=c("exon_ID", "Gene") ][N==length(age_int)]$exon_ID   , diff_age:=a ]
  }
    
  Delta_S_whippet_ME_age_diff <- Delta_S_whippet_ME[ diff_high==TRUE, .(diff_age = min(diff_age, na.rm = TRUE)), by=c("exon_ID", "Gene")]
  
  
  Delta_S_whippet_ME$delta_sing <- sign(Delta_S_whippet_ME$DeltaPsi.x)
  
  Delta_S_whippet_ME_diff_count <- Delta_S_whippet_ME[diff_high==TRUE , .N , by=c("exon_ID", "delta_sing")]
  rescue_exons <- Delta_S_whippet_ME_diff_count[N>=2 & !exon_ID %in% Delta_S_whippet_ME_age_diff[diff_age!=Inf, ]$exon_ID , ]$exon_ID
  
  
  #ages <- Delta_S_included_stats$age
  #age_int <- c(ages[length(ages)])
  Delta_S_whippet_ME[exon_ID %in% rescue_exons]
  
    
  for (a in rev(ages[1:length(ages)-1])){
    
    age_int = cbind(age_int, a)
    
    Delta_S_whippet_ME[exon_ID %in% rescue_exons & age==a & diff_low==TRUE &  exon_ID %in% Delta_S_whippet_ME[age %in% age_int & diff_low==TRUE, .N, by=c("exon_ID", "Gene") ][N==length(age_int)]$exon_ID   , diff_age_low:=a ]
  }
    
  
  Delta_S_whippet_ME_age_diff_low <- Delta_S_whippet_ME[ diff_low==TRUE, .(diff_age = min(diff_age_low, na.rm = TRUE)), by=c("exon_ID", "Gene")]
  
  
  diff_ME_sing <- Delta_S_whippet_ME[diff_high==TRUE, -sign(DeltaPsi.y)]
  names(diff_ME_sing) <- Delta_S_whippet_ME[diff_high==TRUE, exon_ID]
  
  
  Delta_S_whippet_ME_age_diff_total <-  rbind(Delta_S_whippet_ME_age_diff[diff_age!=Inf], Delta_S_whippet_ME_age_diff_low[diff_age!=Inf])
  
  Delta_S_whippet_ME_age_diff_total[, change_dir:=diff_ME_sing[exon_ID] ]
  
  return(Delta_S_whippet_ME_age_diff_total)

}
```


```
Delta_HNM_whippet_ME_age_diff_total <-  whippet_ME_age_stats(Delta_HNM_whippet, Delta_HNM_ME)
Delta_F_whippet_ME_age_diff_total <-  whippet_ME_age_stats(Delta_F_whippet, Delta_F_ME)


Delta_F_whippet_ME_age_diff_total_stats <-  Delta_F_whippet_ME_age_diff_total[, .(Count=.N) , by=c("diff_age", "change_dir") ]
Delta_HNM_whippet_ME_age_diff_total_stats <-  Delta_HNM_whippet_ME_age_diff_total[, .(Count=.N) , by=c("diff_age", "change_dir") ]
```


```
ggplot() +
  geom_bar(data=Delta_F_whippet_ME_age_diff_total_stats[change_dir==1], aes(factor(diff_age), Count), stat = "identity", fill="skyblue") +

  geom_bar(data=Delta_F_whippet_ME_age_diff_total_stats[change_dir==-1], aes(factor(diff_age), -Count), stat = "identity", fill="firebrick") +
  theme(axis.text.x = element_text(angle = 90, vjust = 0),
    axis.title = element_text(colour = NA))
```


```
ggplot() +
  geom_bar(data=Delta_HNM_whippet_ME_age_diff_total_stats[change_dir==1], aes(factor(diff_age), Count), stat = "identity", fill="skyblue") +

  geom_bar(data=Delta_HNM_whippet_ME_age_diff_total_stats[change_dir==-1], aes(factor(diff_age), -Count), stat = "identity", fill="firebrick") +
  theme(axis.text.x = element_text(angle = 90, vjust = 0),
    axis.title = element_text(colour = NA))
```


```
ME_diff_age <- Delta_HNM_whippet_ME_age_diff_total$diff_age
names(ME_diff_age) <- Delta_HNM_whippet_ME_age_diff_total$exon_ID


diff_ME_sing <- Delta_HNM_whippet_ME_age_diff_total$change_dir
names(diff_ME_sing) <- Delta_HNM_whippet_ME_age_diff_total$exon_ID

ME_clusters_table[, diff_age:=NA]
ME_clusters_table[, change_dir:=NA]
ME_clusters_table[, diff_age:=ME_diff_age[ME] ]
ME_clusters_table[, change_dir:=diff_ME_sing[ME] ]

ME_clusters_table[!is.na(diff_age)]


ME_clusters_table[ , Total:=.N, by= ME_clusters]

ME_clusters_table_stats <-  ME_clusters_table[, .N , by=c("ME_clusters", "diff_age", "change_dir", "Total")]

ME_clusters_table_stats[, Percentage:=N*100/Total]


ME_clusters_table_stats[ , sum(N), by=c( "change_dir" ) ] #TOTAL
ME_clusters_table_stats[ , sum(N), by=c( "diff_age", "change_dir" ) ] #TOTAL

ME_clusters_table_stats <- merge(ME_clusters_table_stats, ME_cluster_names[, c("ME_cluster", "ME_cluster.name", "ME_cluster.type")], by.x="ME_clusters", by.y="ME_cluster")

ME_clusters_table_stats_HNM <- ME_clusters_table_stats[diff_age!="NA" & change_dir!="NA"]


ME_clusters_table_stats_HNM_sub <- ME_clusters_table_stats_HNM[ME_cluster.name %in%  ME_cluster_names[ME_cluster.type %in% c("Neuronal", "Neuro-Muscular", "Muscular", "Non-Neuronal"), ME_cluster.name], ]
ME_clusters_table_stats_HNM_sub$ME_cluster.name <- factor(ME_clusters_table_stats_HNM_sub$ME_cluster.name, levels = sort(unique(as.character(ME_clusters_table_stats_HNM_sub$ME_cluster.name))))


Fig5.B <- ggplot() +
  geom_bar(data=ME_clusters_table_stats_HNM_sub[change_dir==1], aes(factor(diff_age), Percentage), stat = "identity", fill="skyblue") +
  geom_bar(data=ME_clusters_table_stats_HNM_sub[change_dir==-1], aes(factor(diff_age), -Percentage), stat = "identity", fill="firebrick") +
  facet_grid( ME_cluster.name ~ .) +
  ylim(c(-30, 75)) +
  ylab("Percentage") +
  xlab("") +
  theme(axis.text.x = element_text(angle = 90, vjust = 0))
Fig5.B
```


```
ME_diff_age <- Delta_F_whippet_ME_age_diff_total$diff_age
names(ME_diff_age) <- Delta_F_whippet_ME_age_diff_total$exon_ID


diff_ME_sing <- Delta_F_whippet_ME_age_diff_total$change_dir
names(diff_ME_sing) <- Delta_F_whippet_ME_age_diff_total$exon_ID

ME_clusters_table[, diff_age:=NA]
ME_clusters_table[, change_dir:=NA]
ME_clusters_table[, diff_age:=ME_diff_age[ME] ]
ME_clusters_table[, change_dir:=diff_ME_sing[ME] ]

ME_clusters_table[!is.na(diff_age)]


#ME_clusters_table[ , Total:=.N, by= ME_clusters]

ME_clusters_table_stats <-  ME_clusters_table[, .N , by=c("ME_clusters", "diff_age", "change_dir", "Total")]

ME_clusters_table_stats[, Percentage:=N*100/Total]


 ME_clusters_table_stats[ , sum(N), by=c( "change_dir" ) ] # Total

ME_clusters_table_stats <- merge(ME_clusters_table_stats, ME_cluster_names[, c("ME_cluster", "ME_cluster.name", "ME_cluster.type")], by.x="ME_clusters", by.y="ME_cluster") 
ME_clusters_table_stats_F <- ME_clusters_table_stats[diff_age!="NA" & change_dir!="NA"]


ME_clusters_table_stats_F_sub <- ME_clusters_table_stats_F[ME_cluster.name %in%  ME_cluster_names[ME_cluster.type %in% c("Neuronal", "Neuro-Muscular", "Muscular", "Non-Neuronal"), ME_cluster.name], ]
ME_clusters_table_stats_F_sub$ME_cluster.name <- factor(ME_clusters_table_stats_F_sub$ME_cluster.name, levels = sort(unique(as.character(ME_clusters_table_stats_F_sub$ME_cluster.name))))


Fig5.C <- ggplot() +
  geom_bar(data=ME_clusters_table_stats_F_sub[change_dir==1], aes(factor(diff_age), Percentage), stat = "identity", fill="skyblue") +
  geom_bar(data=ME_clusters_table_stats_F_sub[change_dir==-1], aes(factor(diff_age), -Percentage), stat = "identity", fill="firebrick") +
  facet_grid( ME_cluster.name ~ .) +
  ylim(c(-30, 75)) +
  theme(axis.text.x = element_text(angle = 90, vjust = 0),
    axis.title = element_text(colour = NA))

Fig5.C
```


```
Delta_SKM_whippet <- fread("/Users/gp7/Google_Drive/Results/ME/Paper/Final_Report/Reps/Rep1/Whippet/control_vs_skeletal_muscle_tissue.diff.microexons")
Delta_SKM_ME <- fread("/Users/gp7/Google_Drive/Results/ME/Paper/Final_Report/Reps/Rep1/Whippet/control_vs_skeletal_muscle_tissue.diff.ME.microexons")
Delta_SKM_whippet_ME <- merge(Delta_SKM_whippet, Delta_SKM_ME, by=c("exon_ID", "Gene", "Node",  "Coord", "Strand", "Type")  )
Delta_SKM_whippet_ME[, diff_high:=FALSE]
Delta_SKM_whippet_ME[ (abs(DeltaPsi.x)>=0.1 &  Probability.x >= 0.9) &  (abs(DeltaPsi.y)>=0.1 &  Probability.y >= 0.9) , diff_high:=TRUE]
Delta_SKM_whippet_ME[diff_high==TRUE , change_dir:=sign(DeltaPsi.x) ]
Delta_SKM_whippet_ME[, Tissue:="SKM"]

Delta_Heart_whippet <- fread("/Users/gp7/Google_Drive/Results/ME/Paper/Final_Report/Reps/Rep1/Whippet/control_vs_heart.diff.microexons")
Delta_Heart_ME <- fread("/Users/gp7/Google_Drive/Results/ME/Paper/Final_Report/Reps/Rep1/Whippet/control_vs_heart.diff.ME.microexons")
Delta_Heart_whippet_ME <- merge(Delta_Heart_whippet, Delta_Heart_ME, by=c("exon_ID", "Gene", "Node",  "Coord", "Strand", "Type")  )
Delta_Heart_whippet_ME[, diff_high:=FALSE]
Delta_Heart_whippet_ME[ (abs(DeltaPsi.x)>=0.1 &  Probability.x >= 0.9) &  (abs(DeltaPsi.y)>=0.1 &  Probability.y >= 0.9) , diff_high:=TRUE]
Delta_Heart_whippet_ME[diff_high==TRUE , change_dir:=sign(DeltaPsi.x) ]
Delta_Heart_whippet_ME[, Tissue:="Heart"]


Delta_AG_whippet <- fread("/Users/gp7/Google_Drive/Results/ME/Paper/Final_Report/Reps/Rep1/Whippet/control_vs_adrenal_gland.diff.microexons")
Delta_AG_ME <- fread("/Users/gp7/Google_Drive/Results/ME/Paper/Final_Report/Reps/Rep1/Whippet/control_vs_adrenal_gland.diff.ME.microexons")
Delta_AG_whippet_ME <- merge(Delta_AG_whippet, Delta_AG_ME, by=c("exon_ID", "Gene", "Node",  "Coord", "Strand", "Type")  )
Delta_AG_whippet_ME[, diff_high:=FALSE]
Delta_AG_whippet_ME[ (abs(DeltaPsi.x)>=0.1 &  Probability.x >= 0.9) &  (abs(DeltaPsi.y)>=0.1 &  Probability.y >= 0.9) , diff_high:=TRUE]
Delta_AG_whippet_ME[diff_high==TRUE , change_dir:=sign(DeltaPsi.x)]
Delta_AG_whippet_ME[, Tissue:="Heart"]
```


```
neural_AG_PSI <- merge(merge(
Delta_AG_whippet_ME[abs(DeltaPsi.x)>=0.1 & Probability.x>0.9 & abs(DeltaPsi.y)>=0.1 & Probability.y>0.9, .(exon_ID, PSI_AG=Psi_B.x)  ],

Delta_HNM_merge[abs(DeltaPsi.x)>=0.1 & Probability.x>0.9 & abs(DeltaPsi.y)>=0.1 & Probability.y>0.9,  .(exon_ID, age, PSI_HNM=Psi_B.x)],
by="exon_ID"),

Delta_F_merge[abs(DeltaPsi.x)>=0.1 & Probability.x>0.9 & abs(DeltaPsi.y)>=0.1 & Probability.y>0.9,  .(exon_ID, age, PSI_F=Psi_B.x)],
by=c("exon_ID", "age") )

table(neural_AG_PSI$age) # Over 14.5 there is the highest number of intersections


neural_AG_PSI_melted <- melt(neural_AG_PSI[age==14.5, c("exon_ID", "PSI_AG", "PSI_HNM", "PSI_F")])


Sup.AG_MHNF.A <- ggplot(neural_AG_PSI_melted, aes(variable, value)) +
  geom_boxplot() +
  geom_jitter() +
  #geom_line(aes(group=exon_ID)) +
  xlab("Tissue") +
  ylab("PSI") +
  ggsignif::geom_signif(test = "wilcox.test", comparisons = list(c("PSI_AG", "PSI_HNM"), c("PSI_AG", "PSI_F"))
, step_increase=0.15 , test.args = list(alternative = "two.sided", paired = TRUE), map_signif_level = TRUE) +
  scale_x_discrete(labels = c('Adrenal Gland','Brain MHN','Forebrain'))


Sup.AG_MHNF.B <- ggplot(neural_AG_PSI_melted, aes(variable, value)) +
  #geom_boxplot() +
  #geom_jitter() +
  geom_line(aes(group=exon_ID)) +
  xlab("Tissue") +
  ylab("PSI") +
  ggsignif::geom_signif(test = "wilcox.test", comparisons = list(c("PSI_AG", "PSI_HNM"), c("PSI_AG", "PSI_F"))
, step_increase=0.15 , test.args = list(alternative = "two.sided", paired = TRUE), map_signif_level = TRUE) +
  scale_x_discrete(labels = c('Adrenal Gland','Brain MHN','Forebrain'))


plot_grid(Sup.AG_MHNF.A, Sup.AG_MHNF.B, ncol=1, labels="AUTO")
```


```
ME_clusters_table[ , Diff_SKM:="FALSE"]
ME_clusters_table[ME %in% Delta_SKM_whippet_ME[diff_high==TRUE & change_dir==1, exon_ID], Diff_SKM:="Excluded" ]
ME_clusters_table[ME %in% Delta_SKM_whippet_ME[diff_high==TRUE & change_dir==-1, exon_ID], Diff_SKM:="Included"]

ME_clusters_table[ , Diff_Heart:="FALSE"]
ME_clusters_table[ME %in% Delta_Heart_whippet_ME[diff_high==TRUE & change_dir==1, exon_ID], Diff_Heart:="Excluded" ]
ME_clusters_table[ME %in% Delta_Heart_whippet_ME[diff_high==TRUE & change_dir==-1, exon_ID], Diff_Heart:="Included"]

ME_clusters_table[ , Diff_AG:="FALSE"]
ME_clusters_table[ME %in% Delta_AG_whippet_ME[diff_high==TRUE & change_dir==1, exon_ID], Diff_AG:="Excluded" ]
ME_clusters_table[ME %in% Delta_AG_whippet_ME[diff_high==TRUE & change_dir==-1, exon_ID], Diff_AG:="Included"]


ME_clusters_table_stats_SKM <- ME_clusters_table[ Diff_SKM!="FALSE", .N, by=c("ME_clusters", "Total", "Diff_SKM") ]
ME_clusters_table_stats_SKM[, Percentage:=(N*100/Total)]
ME_clusters_table_stats_SKM[, Tissue:="SKM"]
colnames(ME_clusters_table_stats_SKM) <- c("ME_clusters", "Total", "Diff", "N", "Percentage", "Tissue" )

ME_clusters_table_stats_Heart <- ME_clusters_table[ Diff_Heart!="FALSE", .N, by=c("ME_clusters", "Total", "Diff_Heart") ]
ME_clusters_table_stats_Heart[, Percentage:=(N*100/Total)]
ME_clusters_table_stats_Heart[, Tissue:="HRT"]
colnames(ME_clusters_table_stats_Heart) <- c("ME_clusters", "Total", "Diff", "N", "Percentage", "Tissue" )

ME_clusters_table_stats_AG <- ME_clusters_table[ Diff_AG!="FALSE", .N, by=c("ME_clusters", "Total", "Diff_AG") ]
ME_clusters_table_stats_AG[, Percentage:=(N*100/Total)]
ME_clusters_table_stats_AG[, Tissue:="AD"]
colnames(ME_clusters_table_stats_AG) <- c("ME_clusters", "Total", "Diff", "N", "Percentage", "Tissue" )


ME_clusters_table_stats_SHA <- rbind(ME_clusters_table_stats_SKM, ME_clusters_table_stats_Heart, ME_clusters_table_stats_AG)


ME_clusters_table_stats_SHA <- merge(ME_clusters_table_stats_SHA, ME_cluster_names[, c("ME_cluster", "ME_cluster.name", "ME_cluster.type")], by.x="ME_clusters", by.y="ME_cluster")

ME_clusters_table_stats_SHA_sub <- ME_clusters_table_stats_SHA[ME_cluster.name %in%  ME_cluster_names[ME_cluster.type %in% c("Neuronal", "Neuro-Muscular", "Muscular", "Non-Neuronal"), ME_cluster.name], ]


ME_clusters_table_stats_SHA_sub$ME_cluster.name <- factor(ME_clusters_table_stats_SHA_sub$ME_cluster.name, levels = sort(unique(as.character(ME_clusters_table_stats_SHA_sub$ME_cluster.name))))


Fig5.D <- ggplot() +
  geom_bar(data=ME_clusters_table_stats_SHA_sub[Diff=="Included"], aes(factor(Tissue), Percentage), stat = "identity", fill="skyblue") +
  geom_bar(data=ME_clusters_table_stats_SHA_sub[Diff=="Excluded"], aes(factor(Tissue), -Percentage), stat = "identity", fill="firebrick") +
  facet_grid( ME_cluster.name ~ .) +
  ylim(c(-30, 75)) +
  theme(axis.text.x = element_text(angle = 90, vjust = 0),
    axis.title = element_text(colour = NA))
Fig5.D
```


```
library(UpSetR)

require(UpSetR)

listInput <- list(one = c(1, 2, 3, 5, 7, 8, 11, 12, 13), two = c(1, 2, 4, 5, 
    10), three = c(1, 5, 6, 7, 8, 9, 10, 12, 13))


Delta_HNM_whippet_ME_age_diff_total[,  exon_ID]

Delta_F_whippet_ME_age_diff_total[, exon_ID ]


tissue_intersection <- list(Brain = ME_clusters_table[diff_age!="NA", ME],
                            SKM = ME_clusters_table[Diff_SKM!=FALSE, ME],
                            Heart = ME_clusters_table[Diff_Heart!=FALSE, ME],
                            AG = ME_clusters_table[Diff_AG!=FALSE, ME]
                            )


tissue_intersection <- list(Brain_HNM = Delta_HNM_whippet_ME_age_diff_total[,  exon_ID],
                            Brain_F = Delta_F_whippet_ME_age_diff_total[,  exon_ID],
                            SKM = ME_clusters_table[Diff_SKM!=FALSE, ME],
                            Heart = ME_clusters_table[Diff_Heart!=FALSE, ME],
                            AG = ME_clusters_table[Diff_AG!=FALSE, ME]
                            )


Fig5.y <- upset(fromList(tissue_intersection), order.by = "freq", nsets = 5, keep.order = T)
Fig5.y
```


```
gg_color_hue <- function(n) {
  hues = seq(15, 375, length = n + 1)
  hcl(h = hues, l = 65, c = 100)[1:n]
}
```


```
library(eulerr)


tissue_intersection <- list(Brain = ME_clusters_table[diff_age!="NA", ME],
                            SKM = ME_clusters_table[Diff_SKM!=FALSE, ME],
                            Heart = ME_clusters_table[Diff_Heart!=FALSE, ME],
                            AG = ME_clusters_table[Diff_AG!=FALSE, ME])


tissue_intersection.venn <- euler(c("Brain" = 212,  
                    "SKM" = 35,  
                    "Heart" = 11, 
                    "AG" = 11,
                    "Brain&AG" = 29,
                    "SKM&Heart" = 21,
                    "Brain&Heart" = 18,
                    "Brain&SKM&Heart" = 18,
                    "Brain&SKM" = 14,
                    "Brain&SKM&Heart&AG" = 3,
                    "Brain&Heart&AG" = 2,
                    "Heart&AG" = 1,
                    "SKM&AG" = 1,
                    "Brain&SKM&AG" = 1))


tissue_intersection.venn <- euler(c("Brain_HNM&Brain_F" = 156,
                                    "Brain_HNM" = 71,
                                    "SKM" = 34,
                                    "Brain_HNM&Brain_F&AG" = 29,
                                    "SKM&Heart" = 20,
                                    "Brain_HNM&Brain_F&Heart" = 19,
                                    "Brain_F" = 15,
                                    "Brain_HNM&Brain_F&SKM&Heart" = 14,
                                    "Brain_HNM&Brain_F&SKM" = 13,
                                    "Heart" = 11,
                                    "AG" = 11,
                                    "Brain_HNM&SKM" = 5,
                                    "Brain_HNM&Brain_F&SKM&Heart&AG" = 5,                                   
                                    "Brain_HNM&SKM&Heart" = 4,
                                    "Brain_HNM&Heart" = 3,
                                    "Brain_F&AG" = 2,
                                    "Brain_HNM&Heart&AG" = 2,
                                    "Heart&AG" = 1,
                                    "SKM&AG" = 1 ,
                                    "Brain_F&SKM" = 1,
                                    "Brain_F&SKM&Heart" = 1,
                                    "Brain_HNM&SKM&AG" = 1,
                                    "Brain_HNM&Brain_F&Heart&AG" = 1,
                                    "Brain_HNM&Brain_F&SKM&AG" = 1))

                    
    
ven <- plot(tissue_intersection.venn, counts = FALSE, font=4, cex=1, alpha=0.5,
     fill=gg_color_hue(5), quantities = list(fontsize = 0))

ven
```


```
library(ggplotify)


Fig5.col2.row1 <- plot_grid(Fig5.B, Fig5.C, Fig5.D, nrow=1,  labels = c("F", "G", "H"), rel_widths = c(2, 1.5, 1.5) )

Fig5.col2 <- plot_grid(Fig5.col2.row1, as.grob(Fig5.y), ncol = 1,   labels = c("", "I"), rel_heights = c(3, 1) )

Fig5.bottom <- plot_grid(Fig5.A, Fig5.col2, nrow=1,  rel_widths = c(1, 1), labels = c("E", ""))


#Fig5 <- plot_grid(Fig5.col1, Fig5.B, Fig5.C, Fig5.D, nrow=1, rel_widths = c(10, 2, 1.5, 1.5 ), labels = c("", "F", "G", "H") )
Fig5 <- plot_grid(Fig5.X, Fig5.bottom, ncol=1, rel_heights = c(1, 4) )
Fig5
```

# Error


```
Delta_HNM_whippet_ME_age_diff[diff_age!="Inf", ]$exon_ID

Delta_HNM_whippet_ME$delta_sing <- sign(Delta_HNM_whippet_ME$DeltaPsi.x)

Delta_HNM_whippet_ME_diff_count <- Delta_HNM_whippet_ME[diff_high==TRUE , .N , by=c("exon_ID", "delta_sing")]


Delta_HNM_whippet_ME_diff_count[ N==2 & !exon_ID %in% Delta_HNM_whippet_ME_age_diff[diff_age!="Inf", ]$exon_ID, ]

Delta_HNM_whippet_ME_age_diff_low[diff_age!=Inf]
```


```
library(biomaRt)


MicroExon_genes <- gsub("\\..*","", Delta_HNM_whippet_ME$Gene) 
names(MicroExon_genes) <- Delta_HNM_whippet_ME$exon_ID
 
Delta_HNM_whippet_ME_age_diff_total[, ensembl_gene_id:=MicroExon_genes[exon_ID]]

ensembl = useEnsembl(biomart="ensembl", dataset="mmusculus_gene_ensembl")


gene_table <- data.table(getBM(attributes=c('ensembl_gene_id', "mgi_symbol"),filters = 'ensembl_gene_id', values = Delta_HNM_whippet_ME_age_diff_total$ensembl_gene_id , mart = ensembl))


gene_table <- data.table(getBM(attributes=c('ensembl_gene_id', "mgi_symbol"),filters = 'ensembl_gene_id', values = Delta_HNM_whippet_ME_age_diff_total$ensembl_gene_id , mart = ensembl))


Delta_HNM_whippet_ME_age_diff_total <- merge( Delta_HNM_whippet_ME_age_diff_total, gene_table, by='ensembl_gene_id')


cat(as.character(unique(Delta_HNM_whippet_ME_age_diff_total$mgi_symbol)), sep='\n') 


Delta_HNM_whippet_ME_age_diff_total[mgi_symbol=="Dctn2"]
```

# Input for PPI network


```
genes_background <- unique(c(Delta_HNM_merge$Gene.x, Delta_F_merge$Gene.x))

genes_background <-  gsub("\\..*","", genes_background)


neuronal_ME_genes <- unique(c(Delta_HNM_whippet_ME_age_diff_total$Gene, Delta_F_whippet_ME_age_diff_total$Gene))
neuronal_ME_genes <- gsub("\\..*","", neuronal_ME_genes)

neuronal_ME_gene_names <-  gene_table[ensembl_gene_id %in% neuronal_ME_genes, ]

cat(as.character(unique(neuronal_ME_gene_names$mgi_symbol)), sep='\n') 


fwrite(data.table(genes_background), "../PPI/genes_background.txt", quote = FALSE, col.names = FALSE)
```


```
Delta_HNM_whippet_ME_age_diff_total[ , ensembl_gene_id:=gsub("\\..*","", Gene) ]
Delta_HNM_whippet_ME_age_diff_total[ , ensembl_gene_id:=gsub("\\..*","", Gene) ]


Delta_HNMF_whippet_ME_age_diff_total <- merge(Delta_HNM_whippet_ME_age_diff_total, Delta_F_whippet_ME_age_diff_total, by=c("exon_ID", "Gene", "change_dir"), all=TRUE)
Delta_HNMF_whippet_ME_age_diff_total[, diff_age:=min(diff_age.y, diff_age.x, na.rm=TRUE), by=exon_ID]

Delta_HNMF_whippet_ME_age_diff_total[ , ensembl_gene_id:=gsub("\\..*","", Gene) ]


Delta_HNMF_whippet_ME_age_diff_total <- merge(Delta_HNMF_whippet_ME_age_diff_total, neuronal_ME_gene_names, by="ensembl_gene_id")


#merge( Delta_HNMF_whippet_ME_age_diff_total, PPI_centrality, by="mgi_symbol")


#Delta_HNMF_whippet_ME_age_diff_total[!mgi_symbol %in% PPI_centrality$mgi_symbol]


fwrite(data.table(data.table(unique(Delta_HNMF_whippet_ME_age_diff_total$ensembl_gene_id))), "../Final_Figures/Figure5/microexons_HMNF_genes.txt", quote = FALSE, col.names = FALSE)
```


```
Delta_Heart_whippet_ME[ , ensembl_gene_id:=gsub("\\..*","", Gene) ]
Delta_SKM_whippet_ME[ , ensembl_gene_id:=gsub("\\..*","", Gene) ]
Delta_AG_whippet_ME[ , ensembl_gene_id:=gsub("\\..*","", Gene) ]


Delta_Heart_whippet_ME <- merge(Delta_Heart_whippet_ME, neuronal_ME_gene_names, by="ensembl_gene_id")
Delta_SKM_whippet_ME <- merge(Delta_SKM_whippet_ME, neuronal_ME_gene_names, by="ensembl_gene_id")
Delta_AG_whippet_ME <- merge(Delta_AG_whippet_ME, neuronal_ME_gene_names, by="ensembl_gene_id")

fwrite(data.table(data.table(unique(Delta_Heart_whippet_ME[diff_high==TRUE, ensembl_gene_id]))), "../Final_Figures/Figure5/heart_genes.txt", quote = FALSE, col.names = FALSE)
fwrite(data.table(data.table(unique(Delta_SKM_whippet_ME[diff_high==TRUE, ensembl_gene_id]))), "../Final_Figures/Figure5/SKM_genes.txt", quote = FALSE, col.names = FALSE)
fwrite(data.table(data.table(unique(Delta_AG_whippet_ME[diff_high==TRUE, ensembl_gene_id]))), "../Final_Figures/Figure5/AG_genes.txt", quote = FALSE, col.names = FALSE)


Delta_Heart_whippet_ME[diff_high==TRUE, ensembl_gene_id]
Delta_SKM_whippet_ME[diff_high==TRUE, ensembl_gene_id]
Delta_AG_whippet_ME[diff_high==TRUE, ensembl_gene_id]
```


```
microexons_Vastdb <- read_delim("~/Google_Drive/Results/ME/mm10/VastDB/VastDb.microexons.txt", 
    " ", escape_double = FALSE, col_names = FALSE, 
    trim_ws = TRUE)


microexons_Vastdb <- microexons_Vastdb$X1


microexons_GENCODE <- read_delim("~/Google_Drive/Results/ME/mm10/gencode.vM16.annotation.microexons.txt", 
    " ", escape_double = FALSE, col_names = FALSE, 
    trim_ws = TRUE)


microexons_GENCODE <- microexons_GENCODE$X1
```


```
ME_final.out[, In.10_percent_of_bulk:=ME %in% me_after_10_samples_filter) ]
```


```
Error: unexpected ')' in "ME_final.out[, In.10_percent_of_bulk:=ME %in% me_after_10_samples_filter)"
```


```
ME_final.out[In.10_percent_of_bulk==TRUE, .N, by="type"]
```


```
fwrite(neuronal_ME_HNMF.table, "../../../../Thesis/"neuronal_ME_HNMF.tsv", sep="\t" )
```


```
Error: unexpected symbol in "fwrite(neuronal_ME_HNMF.table, "../../../../Thesis/"neuronal_ME_HNMF.tsv"
```


```
#library("STRINGdb")

#string_db <- STRINGdb$new( version="10", species=9606, score_threshold=0, input_directory="" )


#neuronal_ME_gene_names_mapped <- string_db$map( neuronal_ME_gene_names, "mgi_symbol", removeUnmappedRows = TRUE )

# string_db$plot_network( neuronal_ME_gene_names_mapped )


#hits <- as.character(unique(neuronal_ME_gene_names$mgi_symbol))

#example1_mapped <- string_db$map( hits, "gene", removeUnmappedRows = TRUE )

#plot_network( hits )

#data(diff_exp_example1)


#example1_mapped <- string_db$map( diff_exp_example1, "gene", removeUnmappedRows = TRUE )

#map

#?STRINGdb
```


```
neuronal_clusters_genes <- merge(neuronal_clusters, gene_info, by.x="transcript", by.y="ensembl_transcript_id")

neuronal_clusters_genes$ME_clusters <- mapvalues(neuronal_clusters_genes$ME_clusters, 
          from =1:18,
          to = c("I1", "E1", "E3", "I2", "N1", "M1", "N2", "NM3", "NM2", "N5", "NM1", "N3", "N4", "NN2", "E2", "I4", "I3", "NN1"))

#Generaring values for pie vertics

colour_pallete <- list( c(heat.colors(8), "#1E90FFFF"))
values_clusters = list()

ME_per_gene = list()

vertex_shape = list()
circle_color = list()

label_color = list()

for (i in 1:144){
  

  gene_name = nodes[i, ]$id


  if (i==16){
    
    gene_name = "Sptan1"   # STRING DB changed the name from Spna2 to Spatan1
  }
  
  if (i==49){
    
    gene_name = "Diaph1"   ## STRING DB changed the name from Diap1 to Diaph1
  }
  
  
  ME_per_gene[[i]] <- nrow(neuronal_clusters_genes[mgi_symbol==gene_name, ])


  N1 =  nrow(neuronal_clusters_genes[mgi_symbol==gene_name  & ME_clusters=="N1", ])
  N2 =  nrow(neuronal_clusters_genes[mgi_symbol==gene_name  & ME_clusters=="N2", ])
  N3 =  nrow(neuronal_clusters_genes[mgi_symbol==gene_name  & ME_clusters=="N3", ])
  N4 =  nrow(neuronal_clusters_genes[mgi_symbol==gene_name  & ME_clusters=="N4", ])
  N5 =  nrow(neuronal_clusters_genes[mgi_symbol==gene_name  & ME_clusters=="N5", ])
  NM1 =  nrow(neuronal_clusters_genes[mgi_symbol==gene_name  & ME_clusters=="NM1", ])
  NM2 =  nrow(neuronal_clusters_genes[mgi_symbol==gene_name  & ME_clusters=="NM2", ])
  NM3 =  nrow(neuronal_clusters_genes[mgi_symbol==gene_name  & ME_clusters=="NM3", ])
  NN1 =  nrow(neuronal_clusters_genes[mgi_symbol==gene_name  & ME_clusters=="NN1", ])
  
  
  #values_clusters[[i]] <- c(N1, N2, N3, N4, N5, NM1, NM2, NM3, NN1)
  
  cluster_counts <- c(NM1, N1, NM2,  N2, NM3, N3, N4, N5,  NN1)
  
  values_clusters[[i]] <- cluster_counts
  
  if (length(which(cluster_counts!=0) ) == 1  ) {
    
    vertex_shape[[i]] <- "circle"   #Nodes with only one kind of cluster need to be circles instead of pie to avoid pie border
    circle_color[[i]] <-  colour_pallete[[1]][which(cluster_counts!=0)] 
    
    
  } else {
    
    
    vertex_shape[[i]] <- "pie"
    circle_color[[i]] <-  "#FFFF00FF"   #This colour is not taken in consideration for pies
    
    
  }
  
  
  #if (gene_name %in%  SFARI_Gene_animal_genes[model.species=="Mus musculus" , gene.symbol]) {
    
  if (gene_name %in%  SFARI_Gene_human_genes[ gene.score<=3 , gene.symbol]) {  
    
    
    
    label_color[[i]] <- "darkgreen"
    
    
  } else{
    
     label_color[[i]] <- "black"
    
  }
  
    
    
  if ( sum( c( N1, N2, N3, N4, N5, NM1, NM2, NM3, NN1)) == 0 ) {
    
    print( c(i, nodes[i, ]$id ) )
  }
  

}
```


```
library(igraph)

vertex.pie.color=list(heat.colors(9))


ME_PPI <- fread("../PPI/ME.PPI.tsv")


links <- ME_PPI[, c("#node1", "node2", "combined_score")]


colnames(links) <- c("from", "to", "score")

nodes.1 <- ME_PPI[, c("#node1")]
colnames(nodes.1) <- c("id")

nodes.2 <- ME_PPI[, c("node2")]
colnames(nodes.2) <- c("id")


nodes <- rbind(nodes.1, nodes.2)


nodes <- unique(  nodes[ , , by="id"] )


net <- graph_from_data_frame(d=links, vertices=nodes, directed=F)

E(net)$size <- log(2-  E(net)$score)

E(net)$width <- (1-  E(net)$score)  * 15


#V(net)$size <- log(unlist(ME_per_gene) + 2) * 4

#E(net)$score

#plot(net, vertex.shape= unlist(vertex_shape), vertex.color = unlist(circle_color)  , vertex.pie=values_clusters, rescale=T, pie.lty=2, vertex.pie.color=colour_pallete, vertex.label.font=2, vertex.label.dist=0.4, vertex.label.color=unlist(label_color), vertex.label.cex=1.2)

#colour_pallete


#plot(net,rescale=T, vertex.label.cex=1.2, vertex.size=1 )


#harmonic_centrality <- PPI_diff_age_HNMF$harmonic_centrality
#names(harmonic_centrality) <- PPI_diff_age_HNMF$mapped_gene

#V(net)$size <- log(harmonic_centrality[names(V(net))] +1 )


#V(net)$size <- harmonic_centrality[names(V(net))]/6

#plot(net)


#PPI_diff_age_HNM_central [  %in% names(V(net))

#harmonic_centrality[names(V(net))]


#harmonic_centrality[names(V(net))]


#harmonic_centrality[names(V(net))]


#names(V(net)) 

#PPI_diff_age_HNM_central[  (mgi_symbol %in%  names(V(net)))==FALSE ,  ]

#PPI_diff_age_HNM_central[mgi_symbol=="Itsn1"]

#PPI_diff_age_HNMF
```


```
library(CINNA)

PPI_betweenness <- estimate_betweenness(net, vids = V(net), directed = F, cutoff=-1,
  weights = NULL, nobigint = TRUE)

PPI_eigen_centrality <- eigen_centrality(net, directed = F, scale = T, weights = NULL)$vector


#cbind(as.numeric(PPI_betweenness[V(net)]), as.numeric( PPI_eigen_centrality[V(net)]  ) )

PPI_harmonic_centrality <- harmonic_centrality(net)


PPI_degree_centrality <- centr_degree(net)$res
names(PPI_degree_centrality) <- V(net)$name

PPI_centrality <- data.frame(cbind(  PPI_betweenness[V(net)] ,  PPI_eigen_centrality[V(net)], PPI_harmonic_centrality, PPI_degree_centrality   ))

colnames(PPI_centrality) <- c("betweenness", "eigen_centrality", "harmonic_centrality", "degree_centrality")

PPI_centrality$mapped_gene <-  row.names(PPI_centrality)

PPI_centrality <- data.table(PPI_centrality)
```


```
PPI_diff_age_HNM_stats_EC <- PPI_diff_age_HNMF[is.na(diff_age.x)!=TRUE][,  .(min_dif_age=min(diff_age.x), Number_of_ME=.N), by=c("ensembl_gene_id", "eigen_centrality") ]
PPI_diff_age_HNM_stats_EC[, rank:=frank(-eigen_centrality)]
PPI_diff_age_HNM_stats_EC[ , max_rank:=max(rank)]
PPI_diff_age_HNM_stats_EC[ , percentil:=rank/max_rank*100]
PPI_diff_age_HNM_stats_EC[, central:=FALSE]
PPI_diff_age_HNM_stats_EC[percentil<15, central:=TRUE]


ggplot(PPI_diff_age_HNM_stats_EC) +
  geom_bar(aes(min_dif_age, fill=central), stat = "count", position="fill" ) +
  xlab("Earliest microexon differential inclusion detection per gene (DPC)")+
  ylab("Protein type proportion") +
  scale_x_continuous(breaks=seq(10.5,16.5,1)) +
  scale_fill_discrete(name = "Protein type", labels = c("Non-central", "Central"))  +
            theme(axis.text.x = element_text(angle = 90), legend.position = "top", legend.direction = "horizontal")


PPI_diff_age_HNM_stats_HC <- PPI_diff_age_HNMF[is.na(diff_age.x)!=TRUE][,  .(min_dif_age=min(diff_age.x), Number_of_ME=.N), by=c("ensembl_gene_id", "harmonic_centrality") ]
PPI_diff_age_HNM_stats_HC[, rank:=frank(-harmonic_centrality)]
PPI_diff_age_HNM_stats_HC[ , max_rank:=max(rank)]
PPI_diff_age_HNM_stats_HC[ , percentil:=rank/max_rank*100]
PPI_diff_age_HNM_stats_HC[, central:=FALSE]
PPI_diff_age_HNM_stats_HC[percentil<15, central:=TRUE]


ggplot(PPI_diff_age_HNM_stats_HC) +
  geom_bar(aes(min_dif_age, fill=central), stat = "count", position="fill" ) +
  xlab("Earliest microexon differential inclusion detection per gene (DPC)")+
  ylab("Protein type proportion") +
  scale_x_continuous(breaks=seq(10.5,16.5,1)) +
  scale_fill_discrete(name = "Protein type", labels = c("Non-central", "Central"))  +
            theme(axis.text.x = element_text(angle = 90), legend.position = "top", legend.direction = "horizontal")


centrality_logistic_regression <- glm(formula = central ~ min_dif_age, family = "binomial", data = PPI_diff_age_HNM_stats_EC)
summary(centrality_logistic_regression)


centrality_logistic_regression <- glm(formula = central ~ min_dif_age, family = "binomial", data = PPI_diff_age_HNM_stats_HC)
summary(centrality_logistic_regression)
```


```
var2str <- function(v1) {
  deparse(substitute(v1))
}
```


```
#GO_Reactome <- fread("../Figures/New_Figure 5/STRING/GO/Whole_genome_background/Reactome Pathways.tsv")


Axon_guidance <- unlist(strsplit(GO_Reactome[`term description`=="Axon guidance"]$`matching proteins in your network (labels)`, ","))
L1CAM_interactions <- unlist(strsplit(GO_Reactome[`term description`=="L1CAM interactions"]$`matching proteins in your network (labels)`, ","))
Protein_protein_interactions_at_synapses <- unlist(strsplit(GO_Reactome[`term description`=="Protein-protein interactions at synapses"]$`matching proteins in your network (labels)`, ","))
ER_to_Golgi_Anterograde_Transport <- unlist(strsplit(GO_Reactome[`term description`=="ER to Golgi Anterograde Transport"]$`matching proteins in your network (labels)`, ","))
Clathrin_mediated_endocytosis <- unlist(strsplit(GO_Reactome[`term description`=="Clathrin-mediated endocytosis"]$`matching proteins in your network (labels)`, ","))
Golgi_Associated_Vesicle_Biogenesis <- unlist(strsplit(GO_Reactome[`term description`=="Golgi Associated Vesicle Biogenesis"]$`matching proteins in your network (labels)`, ","))
Membrane_Trafficking <- unlist(strsplit(GO_Reactome[`term description`=="Membrane Trafficking"]$`matching proteins in your network (labels)`, ","))
Intra_Golgi_and_retrograde_Golgi_to_ER_traffic <- unlist(strsplit(GO_Reactome[`term description`=="Intra-Golgi and retrograde Golgi-to-ER traffic"]$`matching proteins in your network (labels)`, ","))

GOs <- list(Axon_guidance, L1CAM_interactions)

PPI_centrality_GO <- PPI_diff_age_HNMF[, c("mapped_gene", "exon_ID", "diff_age",  "betweenness", "eigen_centrality", "harmonic_centrality", "degree_centrality", "type") ]

PPI_centrality_GO <-
  rbind(PPI_diff_age_HNMF[ mapped_gene %in% Axon_guidance, .(mapped_gene, exon_ID, diff_age,  betweenness, eigen_centrality, harmonic_centrality, degree_centrality, type, GO=var2str(Axon_guidance)) ],
PPI_diff_age_HNMF[ mapped_gene %in% L1CAM_interactions, .(mapped_gene, exon_ID, diff_age,  betweenness, eigen_centrality, harmonic_centrality, degree_centrality, type, GO=var2str(L1CAM_interactions)) ],
PPI_diff_age_HNMF[ mapped_gene %in% Protein_protein_interactions_at_synapses, .(mapped_gene, exon_ID, diff_age,  betweenness, eigen_centrality, harmonic_centrality, degree_centrality, type, GO=var2str(Protein_protein_interactions_at_synapses)) ],
PPI_diff_age_HNMF[ mapped_gene %in% ER_to_Golgi_Anterograde_Transport, .(mapped_gene, exon_ID, diff_age,  betweenness, eigen_centrality, harmonic_centrality, degree_centrality, type, GO=var2str(ER_to_Golgi_Anterograde_Transport)) ],
PPI_diff_age_HNMF[ mapped_gene %in% Clathrin_mediated_endocytosis, .(mapped_gene, exon_ID, diff_age,  betweenness, eigen_centrality, harmonic_centrality, degree_centrality, type, GO=var2str(Clathrin_mediated_endocytosis)) ],
PPI_diff_age_HNMF[ mapped_gene %in% Golgi_Associated_Vesicle_Biogenesis, .(mapped_gene, exon_ID, diff_age,  betweenness, eigen_centrality, harmonic_centrality, degree_centrality, type, GO=var2str(Golgi_Associated_Vesicle_Biogenesis)) ],
PPI_diff_age_HNMF[ mapped_gene %in% Membrane_Trafficking, .(mapped_gene, exon_ID, diff_age,  betweenness, eigen_centrality, harmonic_centrality, degree_centrality, type, GO=var2str(Membrane_Trafficking)) ],
PPI_diff_age_HNMF[ mapped_gene %in% Intra_Golgi_and_retrograde_Golgi_to_ER_traffic, .(mapped_gene, exon_ID, diff_age,  betweenness, eigen_centrality, harmonic_centrality, degree_centrality, type, GO=var2str(Intra_Golgi_and_retrograde_Golgi_to_ER_traffic)) ])


ggplot(PPI_centrality_GO, aes(GO, log(betweenness))) +
  geom_jitter() +
  geom_boxplot() +
  theme(axis.text.x = element_text(angle = 90, vjust = 0))


PPI_centrality_GO_gene_centered <- PPI_centrality_GO[ , .(diff_age=min(diff_age)) , by=c("mapped_gene", "betweenness", "eigen_centrality", "harmonic_centrality", "degree_centrality", "GO")]


ggplot(PPI_centrality_GO_gene_centered ) +
  geom_jitter(aes(harmonic_centrality, diff_age, color=GO), width=0.5, height=0.1 ) +
   theme(axis.text.x = element_text(angle = 90), legend.position = "top", legend.direction = "horizontal")
```


```
ggplot(PPI_centrality_GO_gene_centered, aes(GO, diff_age)) +
+     geom_jitter(height = 0.1, width = 0.1) +
+     theme(axis.text.x = element_text(angle = 90, vjust = 0))
```


```
PPI_centrality_GO_gene_centered_stats <-  PPI_centrality_GO_gene_centered[ , .(N=.N),  by=c("GO", "diff_age")]

PPI_centrality_GO_gene_centered_stats[ , Total:=sum(N), by ="GO"]

PPI_centrality_GO_gene_centered_stats[, fraction:=N/Total ]

diff_age_levels <- PPI_centrality_GO_gene_centered_stats[ , .(mean=mean(diff_age)), by="GO"][order(-mean)]$GO


PPI_centrality_GO_gene_centered_stats$GO <- factor(PPI_centrality_GO_gene_centered_stats$GO, levels= diff_age_levels)

ggplot(PPI_centrality_GO_gene_centered_stats) +
  geom_tile(aes( diff_age, GO, fill = fraction)) +
  scale_fill_gradient(low="grey", high="red")+
  theme_bw()+
  labs(fill = "Gene fraction") +
  theme(axis.text.x = element_text(angle = 90, vjust = 0),
    axis.title = element_text(colour = NA),
    strip.text = element_text(hjust = 0.5))
```


```
GO_Reactome_all <- rbind(
Delta_HNMF_whippet_ME_age_diff_total_PPI[ mapped_gene %in% Axon_guidance, .(mapped_gene, exon_ID, diff_age, GO=var2str(Axon_guidance)) ] ,
Delta_HNMF_whippet_ME_age_diff_total_PPI[ mapped_gene %in% L1CAM_interactions, .(mapped_gene, exon_ID, diff_age, GO=var2str(L1CAM_interactions)) ],
Delta_HNMF_whippet_ME_age_diff_total_PPI[ mapped_gene %in% Protein_protein_interactions_at_synapses, .(mapped_gene, exon_ID, diff_age, GO=var2str(Protein_protein_interactions_at_synapses)) ],
Delta_HNMF_whippet_ME_age_diff_total_PPI[ mapped_gene %in% ER_to_Golgi_Anterograde_Transport, .(mapped_gene, exon_ID, diff_age, GO=var2str(ER_to_Golgi_Anterograde_Transport)) ],
Delta_HNMF_whippet_ME_age_diff_total_PPI[ mapped_gene %in% Clathrin_mediated_endocytosis, .(mapped_gene, exon_ID, diff_age, GO=var2str(Clathrin_mediated_endocytosis)) ],
Delta_HNMF_whippet_ME_age_diff_total_PPI[ mapped_gene %in% Golgi_Associated_Vesicle_Biogenesis, .(mapped_gene, exon_ID, diff_age, GO=var2str(Golgi_Associated_Vesicle_Biogenesis)) ],
Delta_HNMF_whippet_ME_age_diff_total_PPI[ mapped_gene %in% Membrane_Trafficking, .(mapped_gene, exon_ID, diff_age, GO=var2str(Membrane_Trafficking)) ],
Delta_HNMF_whippet_ME_age_diff_total_PPI[ mapped_gene %in% Intra_Golgi_and_retrograde_Golgi_to_ER_traffic, .(mapped_gene, exon_ID, diff_age, GO=var2str(Intra_Golgi_and_retrograde_Golgi_to_ER_traffic))] )


GO_Reactome_all_stats <-  GO_Reactome_all[ , .(N=.N),  by=c("GO", "diff_age")]
GO_Reactome_all_stats[ , Total:=sum(N), by ="GO"]
GO_Reactome_all_stats[, fraction:=N/Total ]
diff_age_levels <- GO_Reactome_all_stats[ , .(mean=mean(diff_age)), by="GO"][order(-mean)]$GO

diff_age_levels <- c("Membrane_Trafficking",
                     "Intra_Golgi_and_retrograde_Golgi_to_ER_traffic",
                     "Golgi_Associated_Vesicle_Biogenesis",
                     "Clathrin_mediated_endocytosis",
                     "ER_to_Golgi_Anterograde_Transport",
                      "Axon_guidance",
                      "L1CAM_interactions",
                      "Protein_protein_interactions_at_synapses")


GO_Reactome_all_stats$GO <- factor(GO_Reactome_all_stats$GO, levels= rev(diff_age_levels))


F6G <- ggplot(GO_Reactome_all_stats) +
  geom_tile(aes( diff_age, GO, fill = fraction)) +
  geom_text(aes(diff_age, GO, label = N), color="white") +
  scale_fill_gradient(low="grey", high="red", limits=c(0, 0.7) )+
    scale_x_continuous(breaks=seq(10.5,16.5,1)) +
  theme_bw()+
  xlab("DPC") +
  ylab("") +
  labs(fill = "Gene fraction") +
  theme(axis.text.x = element_text(angle = 90), legend.position = "top", legend.direction = "horizontal")

F6G
```


```
F6FG <- plot_grid(F6F, F6G, ncol=1, labels = c("F", "G"), rel_heights = c(1.3, 1))
```


```
Biological_Process <- fread("../PPI/enrichment.Process.tsv")
Cellular_Component <- fread("../PPI/enrichment.Component.tsv")
GO_Reactome <- fread("../PPI/enrichment.RCTM.tsv")


nervous_system_development <- unlist(strsplit(Biological_Process[`term description`=="nervous system development"]$`matching proteins in your network (labels)`, ","))
vesicle_mediated_transport <- unlist(strsplit(Biological_Process[`term description`=="vesicle-mediated transport"]$`matching proteins in your network (labels)`, ","))
cytoskeleton_organization <- unlist(strsplit(Biological_Process[`term description`=="cytoskeleton organization"]$`matching proteins in your network (labels)`, ","))
synapse_assembly <- unlist(strsplit(Biological_Process[`term description`=="synapse assembly"]$`matching proteins in your network (labels)`, ","))
signal_transduction <- unlist(strsplit(Biological_Process[`term description`=="signal transduction"]$`matching proteins in your network (labels)`, ","))

synapse <- unlist(strsplit(Cellular_Component[`term description`=="synapse"]$`matching proteins in your network (labels)`, ","))
somatodendritic_compartment <- unlist(strsplit(Cellular_Component[`term description`=="somatodendritic compartment"]$`matching proteins in your network (labels)`, ","))
postsynapse <- unlist(strsplit(Cellular_Component[`term description`=="postsynapse"]$`matching proteins in your network (labels)`, ","))
presynapse <- unlist(strsplit(Cellular_Component[`term description`=="presynapse"]$`matching proteins in your network (labels)`, ","))
growth_cone <- unlist(strsplit(Cellular_Component[`term description`=="growth cone"]$`matching proteins in your network (labels)`, ","))

GO_Biological_Process <- rbind(
Delta_HNMF_whippet_ME_age_diff_total_PPI[ mapped_gene %in% nervous_system_development, .(mapped_gene, exon_ID, diff_age, GO=var2str(nervous_system_development)) ],
Delta_HNMF_whippet_ME_age_diff_total_PPI[ mapped_gene %in% vesicle_mediated_transport, .(mapped_gene, exon_ID, diff_age, GO=var2str(vesicle_mediated_transport)) ],
Delta_HNMF_whippet_ME_age_diff_total_PPI[ mapped_gene %in% cytoskeleton_organization, .(mapped_gene, exon_ID, diff_age, GO=var2str(cytoskeleton_organization)) ],
Delta_HNMF_whippet_ME_age_diff_total_PPI[ mapped_gene %in% synapse_assembly, .(mapped_gene, exon_ID, diff_age, GO=var2str(synapse_assembly)) ],
Delta_HNMF_whippet_ME_age_diff_total_PPI[ mapped_gene %in% signal_transduction, .(mapped_gene, exon_ID, diff_age, GO=var2str(signal_transduction)) ] )

GO_Cellular_Component <- rbind(
Delta_HNMF_whippet_ME_age_diff_total_PPI[ mapped_gene %in% synapse, .(mapped_gene, exon_ID, diff_age, GO=var2str(synapse)) ],
Delta_HNMF_whippet_ME_age_diff_total_PPI[ mapped_gene %in% somatodendritic_compartment, .(mapped_gene, exon_ID, diff_age, GO=var2str(somatodendritic_compartment)) ],
Delta_HNMF_whippet_ME_age_diff_total_PPI[ mapped_gene %in% postsynapse, .(mapped_gene, exon_ID, diff_age, GO=var2str(postsynapse)) ],
Delta_HNMF_whippet_ME_age_diff_total_PPI[ mapped_gene %in% presynapse, .(mapped_gene, exon_ID, diff_age, GO=var2str(presynapse)) ],
Delta_HNMF_whippet_ME_age_diff_total_PPI[ mapped_gene %in% growth_cone, .(mapped_gene, exon_ID, diff_age, GO=var2str(growth_cone)) ] )


GO_Biological_Process_stats <-  GO_Biological_Process[ , .(N=.N),  by=c("GO", "diff_age")]
GO_Biological_Process_stats[ , Total:=sum(N), by ="GO"]
GO_Biological_Process_stats[, fraction:=N/Total ]
diff_age_levels <- GO_Biological_Process_stats[ , .(mean=median(diff_age)), by="GO"][order(-mean)]$GO
GO_Biological_Process_stats$GO <- factor(GO_Biological_Process_stats$GO, levels= diff_age_levels)


Sup_GO_time.A <- ggplot(GO_Biological_Process_stats) +
  geom_tile(aes( diff_age, GO, fill = fraction)) +
    geom_text(aes(diff_age, GO, label = N), color="white") +
  scale_fill_gradient(low="grey", high="red" )+
  theme_bw()+
  labs(fill = "Gene fraction") +
  theme(axis.text.x = element_text(angle = 90, vjust = 0),
    axis.title = element_text(colour = NA),
    strip.text = element_text(hjust = 0.5))


GO_Cellular_Component_stats <-  GO_Cellular_Component[ , .(N=.N),  by=c("GO", "diff_age")]
GO_Cellular_Component_stats[ , Total:=sum(N), by ="GO"]
GO_Cellular_Component_stats[, fraction:=N/Total ]
diff_age_levels <- GO_Cellular_Component_stats[ , .(mean=median(diff_age)), by="GO"][order(-mean)]$GO
GO_Cellular_Component_stats$GO <- factor(GO_Cellular_Component_stats$GO, levels= diff_age_levels)


Sup_GO_time.B <- ggplot(GO_Cellular_Component_stats) +
  geom_tile(aes( diff_age, GO, fill = fraction)) +
  geom_text(aes(diff_age, GO, label = N), color="white") +
  scale_fill_gradient(low="grey", high="red" )+
  theme_bw()+
  labs(fill = "Gene fraction") +
  theme(axis.text.x = element_text(angle = 90, vjust = 0),
    axis.title = element_text(colour = NA),
    strip.text = element_text(hjust = 0.5))
```


```
library(ggpubr)


GO_Biological_Process_centrality <-  merge(GO_Biological_Process,  PPI_diff_age_HNMF[, c("exon_ID", "mapped_gene", "betweenness", "eigen_centrality", "harmonic_centrality", "degree_centrality", "type") ], by=c("exon_ID", "mapped_gene"))


GO_Biological_Process_centrality <- unique(GO_Biological_Process_centrality[, c("mapped_gene", "GO", "betweenness", "eigen_centrality", "harmonic_centrality", "degree_centrality")])[harmonic_centrality>10]  #Filterning nodes that are not conected to the main network


GO_Biological_Process_centrality$GO <- factor(GO_Biological_Process_centrality$GO,  levels = GO_Biological_Process_centrality[ , .(median=median(harmonic_centrality)) , by="GO" ][order(-median)]$GO)


ggplot(unique(GO_Biological_Process_centrality[, c("mapped_gene", "GO", "betweenness", "eigen_centrality", "harmonic_centrality", "degree_centrality")])[harmonic_centrality>10] )  +
  geom_boxplot(aes(GO, harmonic_centrality  ) ) +
    ylab("eigencentrality") +
    theme(axis.text.x = element_text(angle = 45, vjust = 0.6),
    axis.title.y = element_text(colour = NA),
    strip.text = element_text(hjust = 0.5))


GO_Biological_Process_centrality$GO <- factor(GO_Biological_Process_centrality$GO,  levels = GO_Biological_Process_centrality[ , .(median=median(eigen_centrality)) , by="GO" ][order(-median)]$GO)


ggplot( GO_Biological_Process_centrality )  +
  geom_boxplot(aes(GO, eigen_centrality  ) ) +
    ylab("eigencentrality") +
    theme(axis.text.x = element_text(angle = 90, vjust = 0.6),
    axis.title.x = element_text(colour = NA),
    strip.text = element_text(hjust = 0.5))

GO_Biological_Process_centrality$GO <- factor(GO_Biological_Process_centrality$GO,  levels = GO_Biological_Process_centrality[ , .(median=median(betweenness)) , by="GO" ][order(-median)]$GO)


ggplot(unique(GO_Biological_Process_centrality[, c("mapped_gene", "GO", "betweenness", "eigen_centrality", "harmonic_centrality", "degree_centrality")])[harmonic_centrality>10] )  +
  geom_boxplot(aes(GO, log10(betweenness))   ) +
    theme(axis.text.x = element_text(angle = 45, vjust = 0.6),
    axis.title = element_text(colour = NA),
    strip.text = element_text(hjust = 0.5))
```


```
GO_Biological_Process_centrality$GO <- factor(GO_Biological_Process_centrality$GO,  levels = GO_Biological_Process_centrality[ , .(median=median(eigen_centrality)) , by="GO" ][order(-median)]$GO)


my_comparisons <- list(c("vesicle_mediated_transport", "cytoskeleton_organization" ),
                       c("vesicle_mediated_transport", "signal_transduction" ),
                       c("vesicle_mediated_transport", "nervous_system_development"),
                       c("vesicle_mediated_transport", "synapse_assembly" ),
                       c("cytoskeleton_organization", "signal_transduction" ),
                       c("cytoskeleton_organization", "nervous_system_development" ),
                       c("cytoskeleton_organization", "synapse_assembly"),
                       c("signal_transduction", "nervous_system_development" ),
                       c("signal_transduction", "nervous_system_development" ),
                       c("nervous_system_development", "synapse_assembly" )
                       )


ggplot( GO_Biological_Process_centrality, aes(GO, eigen_centrality  )  )  +
  geom_boxplot() +
    ylab("eigencentrality") +
    theme(axis.text.x = element_text(angle = 90, vjust = 0.6),
    axis.title.x = element_text(colour = NA),
    strip.text = element_text(hjust = 0.5)) +
  geom_signif(comparisons = my_comparisons, 
              map_signif_level=TRUE , y_position =  seq(0.9, 1.9, 0.1))
```


```
GO_Biological_Process_centrality_HNM_age <- merge(GO_Biological_Process_centrality, Delta_HNMF_whippet_ME_age_diff_total_PPI[is.na(diff_age.x)!=TRUE][ , .(min_age_HNM=min(diff_age.x))  , by="mapped_gene" ], by="mapped_gene")

kruskal.test(data = GO_Biological_Process_centrality_HNM_age,  min_age_HNM ~ GO)


my_comparisons <- list(#c("vesicle_mediated_transport", "cytoskeleton_organization" ),
                       #c("vesicle_mediated_transport", "signal_transduction" ),
                       #c("vesicle_mediated_transport", "nervous_system_development"),
                       #c("vesicle_mediated_transport", "synapse_assembly" ),
                       c("cytoskeleton_organization", "signal_transduction" ),
                       c("cytoskeleton_organization", "nervous_system_development" ),
                       c("cytoskeleton_organization", "synapse_assembly")
                       #c("signal_transduction", "nervous_system_development" ),
                       #c("signal_transduction", "nervous_system_development" ),
                       #c("nervous_system_development", "synapse_assembly" )
                       )


F6I <- ggplot( GO_Biological_Process_centrality_HNM_age, aes(GO, min_age_HNM  )  )  +
  geom_boxplot() +
    ylab("Earliest microexon alternative inclusion per gene") +
   theme_bw() +
    theme(axis.text.x = element_text(angle = 90, vjust = 0.6),
    axis.title.x = element_text(colour = NA),
    strip.text = element_text(hjust = 0.5)) +
      scale_y_continuous(breaks=seq(10.5,16.5,1)) +
  ggsignif::geom_signif(test = "wilcox.test", comparisons = my_comparisons, step_increase=0.15 , test.args = list(alternative = "less", paired = FALSE), map_signif_level = TRUE) +
  scale_x_discrete(labels=c("Vesicle mediated transportt", "Cytoskeleton organization", "Signal transduction", "Nervous system development", "Synapse assembly"))


kruskal.test(GO_Biological_Process_centrality_HNM_age$min_age_HNM, GO_Biological_Process_centrality_HNM_age$GO)
pairwise.wilcox.test(GO_Biological_Process_centrality_HNM_age$min_age_HNM, GO_Biological_Process_centrality_HNM_age$GO)
```


```
library(ggstatsplot)
df_pair <- ggstatsplot::pairwise_p(GO_Biological_Process_centrality, eigen_centrality,  type = "nonparametric", GO)
```


```
kruskal.test(data = GO_Biological_Process_centrality,  eigen_centrality ~ GO)
kruskal.test(data = GO_Biological_Process_centrality,  harmonic_centrality ~ GO)


pairwise.wilcox.test(GO_Biological_Process_centrality$eigen_centrality, GO_Biological_Process_centrality$GO)
pairwise.wilcox.test(GO_Biological_Process_centrality$harmonic_centrality, GO_Biological_Process_centrality$GO)
```


```
pairwise.wilcox.test(GO_Biological_Process_centrality$eigen_centrality, GO_Biological_Process_centrality$GO)
pairwise.wilcox.test(GO_Biological_Process_centrality$harmonic_centrality, GO_Biological_Process_centrality$GO)
```


```
Biological_Process <- fread("../Figures/New_Figure 5/STRING/GO/ME_genes_background/Biological_Process.tsv")
Cellular_Component <- fread("../Figures/New_Figure 5/STRING/GO/ME_genes_background/Cellular_Component.tsv")
Protein_Domains <- fread("../Figures/New_Figure 5/STRING/GO/ME_genes_background/INTERPRO Protein Domains and Features.tsv")


Biological_Process[ , `:=`( minus_log10_FDR=-log10(`false discovery rate`),
                            GO_size=(`observed gene count` + `background gene count`) ,
                            GO_fraction=(`observed gene count`/`background gene count`) ,
                            GO_class="Biological Process") ]


Cellular_Component[ , `:=`( minus_log10_FDR=-log10(`false discovery rate`),
                            GO_size=(`observed gene count` + `background gene count`) ,
                            GO_fraction=(`observed gene count`/`background gene count`) ,
                            GO_class="Cellular Component") ]


Protein_Domains[ , `:=`( minus_log10_FDR=-log10(`false discovery rate`),
                            GO_size=(`observed gene count` + `background gene count`) ,
                            GO_fraction=(`observed gene count`/`background gene count`) ,
                            GO_class="INTERPRO Protein Domains") ]


Total_GO <- rbind(Biological_Process, Cellular_Component, Protein_Domains)


ggplot(Total_GO[GO_size>20]) + 
  geom_point( aes(GO_fraction, minus_log10_FDR, size=log2(GO_size), colour=GO_class),  alpha=0.2) +
  facet_grid( . ~  GO_class ) +
  theme(axis.text.x = element_text(angle = 90), legend.position = "top", legend.direction = "horizontal")
```


```
ggplot(PPI_diff_age_HNM_stats_EC) +
  geom_bar(aes(min_dif_age, fill=central), stat = "count", position="fill" ) +
  xlab("Earliest microexon differential inclusion detection per gene (DPC)")+
  ylab("Protein type proportion") +
  scale_x_continuous(breaks=seq(10.5,16.5,1)) +
  scale_fill_discrete(name = "Protein type", labels = c("Non-central", "Central"))  +
            theme(axis.text.x = element_text(angle = 90), legend.position = "top", legend.direction = "horizontal")


ggplot(PPI_diff_age_HNM_stats_B) +
  geom_bar(aes(min_dif_age, fill=central), stat = "count", position="fill" ) +
  xlab("Earliest microexon differential inclusion detection per gene (DPC)")+
  ylab("Protein type proportion") +
  scale_x_continuous(breaks=seq(10.5,16.5,1)) +
  scale_fill_discrete(name = "Protein type", labels = c("Non-central", "Central"))  +
            theme(axis.text.x = element_text(angle = 90), legend.position = "top", legend.direction = "horizontal")
```

# Zebrafish


```
total_zebra <-  fread("../../Zebrafish/Final_Report/out_filtered_ME.txt")
total_zebra_cov <- fread("../../Zebrafish/Final_Report/out_filtered_ME.cov.txt")


exons.zebra <- fread("../../Zebrafish/danRer11.ensembl.bed12.exons")


mouse_to_zebra <- fread("../../Zebrafish/Conserved/out.high_quality.txt.mm10.bed.liftOver_minMatch_0.01.danRer11.closest_d.out.high_quality.txt.danRer11.bed.formatted")
zebra_to_mouse <- fread("../../Zebrafish/Conserved/out.high_quality.txt.danRer11.bed.liftOver_minMatch_0.01.mm10.closest_d.out.high_quality.txt.mm10.formatted")


mouse_to_zebra <- fread("../../Zebrafish/Conserved/Old/out.high_quality.mm10.bed_liftover_mm10todanRer11.overlap_wo.out.high_quality.bed")
zebra_to_mouse <- fread("../../Zebrafish/Conserved/Old/out.high_quality.bed.liftover_0.001_mm10.overlap_wo.out.high_quality.bed.liftover_0.001_mm10")

Mouse_Zebra.IDs <- unique(rbind(mouse_to_zebra[  , .(ME.mouse=V4, ME.zebra=V10 ) ], zebra_to_mouse[ , .(ME.mouse=V10, ME.zebra=V4) ] ))  #In here we are already applying a filter of 0.8 lengh


#View(Mouse_Zebra.IDs[, .(mouse.len, zebra.len, abs(mouse.len-abs(mouse.len-zebra.len))/max(mouse.len,zebra.len) )] )


mouse_len <- c(mouse_to_zebra[, V3-V2], zebra_to_mouse[, V9-V8])
names(mouse_len) <- c(mouse_to_zebra$V4, zebra_to_mouse$V10)


zebra_len <-  c(zebra_to_mouse[, V3-V2], mouse_to_zebra[, V9-V8])
names(zebra_len) <- c(zebra_to_mouse$V4, mouse_to_zebra$V10)


Mouse_Zebra.IDs[, mouse.len:=mouse_len[ME.mouse]]
Mouse_Zebra.IDs[, zebra.len:=zebra_len[ME.zebra]]


ggplot(Mouse_Zebra.IDs) +
  geom_bin2d(aes(mouse.len, zebra.len), bins = 30, color ="white") +
  theme_bw() +
  scale_fill_gradient(low =  "#00AFBB", high = "#FC4E07")


Mouse_Zebra.IDs <- Mouse_Zebra.IDs[mouse.len==zebra.len, ]

Mouse_Zebra.IDs.table <- merge(Mouse_Zebra.IDs, ME_cluster_names[ , c("ME_cluster.name" , "ME_cluster.type")], by="ME_cluster.name", all = TRUE)


Mouse_Zebra.IDs.table <- Mouse_Zebra.IDs.table[ , .(mouse.coord=ME.mouse, mouse.len=mouse.len, mouse.cluster_name=ME_cluster.name, mouse.cluster_type=ME_cluster.type, 
                           mouse.anno=type, mouse.ensembl=ensemble_mouse, 
                           zebra.coord=ME.zebra, zebra.len=zebra.len, zebra.ensembl=ensemble_zebra)] 


is.data.table(Mouse_Zebra.IDs.table)

Mouse_Zebra.IDs.table <- unique(merge(Mouse_Zebra.IDs.table, gene_table.TOTAL[ , c("ME", "mgi_symbol")], by.x="mouse.coord" , by.y="ME" ))

div_3 <- function(x) x %% 3 == 0]

Mouse_Zebra.IDs.table[ , .(mouse.coord, mgi_symbol, mouse.cluster_name, mouse.cluster_type, mouse.len, mouse.sym=(mouse.len%%3 == 0), mouse.ensembl, zebra.coord, zebra.len, zebra.sym=(zebra.len%%3 == 0), zebra.ensembl)  ]


Mouse_Zebra.IDs.thesis.table <- unique(Mouse_Zebra.IDs.table[ , .(mouse.coord, mgi_symbol, mouse.cluster_name, mouse.cluster_type,
                                                                  mouse.len, mouse.sym=(mouse.len%%3 == 0), mouse.ensembl,
                                                                  zebra.num=.N,
                                                                  zebra.coords=as.character(list(zebra.coord)),
                                                                  zebra.lens=as.character(list(zebra.len)),
                                                                  zebra.syms=as.character(list((zebra.len%%3 == 0))),
                                                                  zebra.ensembls=as.character(list(zebra.ensembl) )),
                                                              by= mouse.coord  ][order(mgi_symbol, mouse.coord)])


Mouse_Zebra.IDs.thesis.table[ , `:=`( zebra.coords=gsub('^c\\(|\\)$|\ |\"' , "", zebra.coords),
                                      zebra.lens=gsub('^c\\(|\\)$|\ |\"' , "", zebra.lens),
                                      zebra.syms=gsub('^c\\(|\\)$|\ |\"' , "", zebra.syms),
                                      zebra.ensembls=gsub('^c\\(|\\)$|\ |\"' , "", zebra.ensembls)
                                                        )]


fwrite(Mouse_Zebra.IDs.table, "../Final_Figures/Supplementary/Mouse_Zebra_microexons.tsv", append = FALSE, quote = "auto", sep = ",",  row.names = FALSE, col.names = TRUE )
fwrite(Mouse_Zebra.IDs.thesis.table, "~/Google_Drive/Thesis/Mouse_zebrafish.tsv", append = FALSE, quote = "auto", sep = "\t",  row.names = FALSE, col.names = TRUE )
```


```
Mouse_Zebra.IDs.thesis.table
```


```
                    mouse.coord                mouse.coord    mgi_symbol mouse.cluster_name mouse.cluster_type mouse.len mouse.sym mouse.ensembl zebra.num
  1: chr2_+_120495235_120495247 chr2_+_120495235_120495247                               O2              Other        12      TRUE          TRUE         1
  2: chr9_+_107346145_107346171 chr9_+_107346145_107346171 6430571L13Rik                 I1           Included        26     FALSE          TRUE         1
  3:   chr1_+_60438941_60438959   chr1_+_60438941_60438959          Abi2                 N4           Neuronal        18      TRUE          TRUE         2
  4:   chr1_+_60438952_60438959   chr1_+_60438952_60438959          Abi2                 E1           Excluded         7     FALSE         FALSE         2
  5:  chr11_+_84290287_84290311  chr11_+_84290287_84290311         Acaca                 I1           Included        24      TRUE          TRUE         1
 ---                                                                                                                                                      
312:   chr3_+_16183921_16183946   chr3_+_16183921_16183946        Ythdf3                 I1           Included        25     FALSE          TRUE         1
313: chr4_+_129340727_129340752 chr4_+_129340727_129340752       Zbtb8os                 I2           Included        25     FALSE          TRUE         1
314:  chr15_-_93395586_93395615  chr15_-_93395586_93395615         Zcrb1                 I1           Included        29     FALSE          TRUE         1
315:  chr19_+_41938807_41938836  chr19_+_41938807_41938836       Zdhhc16                 O2              Other        29     FALSE          TRUE         2
316:   chr8_-_82771338_82771358   chr8_-_82771338_82771358        Zfp330               <NA>               <NA>        20     FALSE          TRUE         1
                                          zebra.coords zebra.lens  zebra.syms zebra.ensembls
  1:                         chr22_-_26308944_26308956         12        TRUE          FALSE
  2:                          chr6_+_53364857_53364883         26       FALSE           TRUE
  3:   chr9_+_13770822_13770840,chr6_+_9985080_9985098      18,18   TRUE,TRUE     FALSE,TRUE
  4:   chr9_+_13770822_13770840,chr6_+_9985080_9985098      18,18   TRUE,TRUE     FALSE,TRUE
  5:                          chr5_-_56372282_56372306         24        TRUE           TRUE
 ---                                                                                        
312:                         chr24_+_24286425_24286450         25       FALSE           TRUE
313:                         chr13_+_44860543_44860568         25       FALSE           TRUE
314:                          chr4_-_13929692_13929721         29       FALSE           TRUE
315: chr12_-_2857061_2857090,chr13_-_31384958_31384987      29,29 FALSE,FALSE      TRUE,TRUE
316:                          chr1_-_53276279_53276299         20       FALSE           TRUE
```


```
Mouse_Zebra.IDs.table.summer[!is.na(mouse.coord), ]
```


```
                     mouse.coord mouse.len mouse.cluster_name mouse.cluster_type         mouse.anno mouse.ensembl               zebra.coord zebra.len zebra.ensembl
  1: chr10_+_106859776_106859806        30                 I1           Included  Missing in VastDB          TRUE  chr4_-_21989573_21989603        30          TRUE
  2:   chr10_+_24883203_24883221        18                NM2     Neuro-Muscular          Annotated          TRUE   chr20_-_2566047_2566065        18         FALSE
  3:   chr10_+_28554769_28554799        30                 N4           Neuronal          Annotated          TRUE   chr20_-_1490720_1490750        30          TRUE
  4:   chr10_+_28570173_28570191        18               <NA>               <NA>          Annotated          TRUE   chr20_-_1472362_1472380        18          TRUE
  5:   chr10_+_69823077_69823101        24                 E2           Excluded          Annotated          TRUE chr17_-_20794173_20794197        24          TRUE
 ---                                                                                                                                                               
398:    chrX_-_13596685_13596694         9                 N6           Neuronal Missing in GENCODE         FALSE  chr9_-_33407086_33407104        18         FALSE
399:    chrX_-_13596685_13596703        18                 N2           Neuronal  Missing in VastDB          TRUE  chr9_-_33407086_33407104        18         FALSE
400:    chrX_-_57241805_57241826        21                 I3           Included          Annotated          TRUE chr14_-_31813517_31813538        21          TRUE
401:    chrX_-_73855651_73855663        12                NM5     Neuro-Muscular          Annotated          TRUE chr23_+_28680393_28680405        12          TRUE
402:    chrX_-_74232966_74232990        24                NM3     Neuro-Muscular          Annotated          TRUE chr23_+_19793592_19793616        24          TRUE
     MHN.diff_age MHN.change_dir F.diff_age F.change_dir
  1:           NA             NA         NA           NA
  2:         13.5              1         NA           NA
  3:         13.5              1       14.5            1
  4:           NA             NA         NA           NA
  5:           NA             NA         NA           NA
 ---                                                    
398:           NA             NA       12.5            1
399:         11.5              1       12.5            1
400:           NA             NA         NA           NA
401:         14.5              1       14.5            1
402:           NA             NA         NA           NA
```


```
Mouse_Zebra.IDs[ME.mouse %in%  names(ME_clusters), ME_cluster:=ME_clusters[ME.mouse] ]


Mouse_Zebra.IDs<- merge(Mouse_Zebra.IDs , 
ME_cluster_names[, c("ME_cluster", "ME_cluster.name")] , by="ME_cluster", all.x=TRUE )


ggplot(Mouse_Zebra.IDs) +
  geom_bar(aes(x=ME_cluster.name), stat="count")


Mouse_Zebra.IDs[ , type:="Annotated"  ]

Mouse_Zebra.IDs[!ME.mouse %in% microexons_Vastdb, type:="Missing in VastDB"   ]
Mouse_Zebra.IDs[!ME.mouse %in% microexons_GENCODE, type:="Missing in GENCODE"   ]
Mouse_Zebra.IDs[!ME.mouse %in% microexons_GENCODE & !ME.mouse %in% microexons_Vastdb , type:="Novel"   ]


Mouse_Zebra.IDs[ , ensemble_zebra:=FALSE  ]
Mouse_Zebra.IDs[ME.zebra %in%  exons.zebra$V4, ensemble_zebra:=TRUE ]

Mouse_Zebra.IDs[ , ensemble_mouse:=FALSE  ]
Mouse_Zebra.IDs[ME.mouse %in%  microexons_GENCODE, ensemble_mouse:=TRUE ]


Delta_HNMF_whippet_ME_age_diff_total[exon_ID %in% Mouse_Zebra.IDs$ME.mouse]


Delta_HNMF_whippet_ME_age_diff_total[]


Mouse_Zebra.IDs[ME.mouse %in% Delta_HNMF_whippet_ME_age_diff_total$exon_ID][ ME.zebra %in% exons.zebra$V4]

 

ggplot(Mouse_Zebra.IDs) +
  geom_bar(aes(x=ME_cluster.name, fill=type), stat="count", position="fill")


length(unique(Mouse_Zebra.IDs$ME.mouse))

length(unique(Mouse_Zebra.IDs$ME.zebra))

PPI_diff_age_HNMF_central[ , conserved:=FALSE ]
PPI_diff_age_HNMF_central[exon_ID %in% Mouse_Zebra.IDs$ME.mouse, conserved:=TRUE ]

ggplot(PPI_diff_age_HNMF_central) +
  geom_jitter(aes(harmonic_centrality, diff_age,  shape= type, colour=conserved  ), width=0, height=0.1) + 
  xlab("Harmonic Centrality") +
  ylab("Earliest microexon differential inclusion detection (DPC)") +
   scale_y_continuous(breaks=seq(10.5,16.5,1)) +
    geom_text_repel(data=PPI_diff_age_HNMF_central[  central==TRUE  ], 
                  aes(x=harmonic_centrality, y=diff_age,  colour=conserved  ), 
                  nudge_y      = 0.5,
                  direction    = "x",
                  angle        = 90,
                  segment.size = 0.2,
                  label=PPI_diff_age_HNMF_central[central==TRUE  , mapped_gene],
                  show.legend = FALSE) +
          theme(axis.text.x = element_text(angle = 90), legend.position = "top", legend.direction = "horizontal")


ggplot(PPI_diff_age_HNMF_central) +
  geom_boxplot(aes(conserved, log2(betweenness) ))
```


```
Mouse_Zebra.IDs[ensemble_zebra==FALSE]


zebra.zebra <-  ggplot(Mouse_Zebra.IDs) + 
  geom_bar(aes(ME_cluster.name, fill=ensemble_zebra), stat="count" ) +
    theme(axis.text.x = element_text(angle = 45), legend.position = "top", legend.direction = "horizontal")


zebra.mouse <- ggplot(Mouse_Zebra.IDs) + 
  geom_bar(aes(ME_cluster.name, fill=ensemble_mouse), stat="count") +
    theme(axis.text.x = element_text(angle = 45), legend.position = "top", legend.direction = "horizontal")
```


```
Mouse_Zebra.IDs.cluster_names <- merge(Mouse_Zebra.IDs, ME_cluster_names[, c("ME_cluster.name", "ME_cluster.type")], by="ME_cluster.name")
Mouse_Zebra_stats  <- Mouse_Zebra.IDs.cluster_names[ , .N , by=c("ME_cluster.type", "ensemble_zebra", "ensemble_mouse")]

Mouse_Zebra_stats[ , Type:=paste(ensemble_zebra, ensemble_mouse, sep = "_") ]

Mouse_Zebra_stats$ME_cluster.type <- factor(Mouse_Zebra_stats$ME_cluster.type,  levels=Mouse_Zebra_stats[!ensemble_zebra  | !ensemble_mouse , sum(N), by=c("ME_cluster.type")][order(V1), ME_cluster.type])

  
  

Fig7.E <- ggplot(Mouse_Zebra_stats[!ensemble_zebra  | !ensemble_mouse]) +
  geom_bar(aes(x=ME_cluster.type, y=N, fill=Type), stat = "identity", position="stack"  ) + 
  ylab("Number of microexons") +
  xlab("Microexon clusters") + 
   scale_fill_discrete(name = "Type", labels = c("Missing in both", "Missing in zebrafish", "Missing in mouse")) +
            theme(axis.text.x = element_text(angle = 90), legend.position = "top", legend.direction = "horizontal") +
    theme_bw() + 
  theme(axis.text.x = element_text(angle = 90))


Fig7.E
```


```
length(unique(Mouse_Zebra.IDs$ME.mouse))
length(unique(Mouse_Zebra.IDs$ME.zebra))

round((Mouse_Zebra_stats[ensemble_mouse==FALSE, sum(N) ] * 100) / length(unique(Mouse_Zebra.IDs$ME.mouse)), 1)
round((Mouse_Zebra_stats[ensemble_zebra==FALSE, sum(N) ] * 100 ) / length(unique(Mouse_Zebra.IDs$ME.zebra)), 1)
```


```
library(ggsignif)

#Mouse_Zebra.IDs[, ME_cluster.name:=ME_cluster.name.x]

Mouse_Zebra.conserved_frac <- unique(Mouse_Zebra.IDs[, c("ME.mouse", "ME_cluster.name")])[ ,.N , by=ME_cluster.name ]

Mouse_Zebra.conserved_frac <-  merge( Mouse_Zebra.conserved_frac , ME_clusters.names[ , .(Total=.N) , by=ME_cluster.name], by="ME_cluster.name")

Mouse_Zebra.conserved_frac[, percetage:=(N*100/Total)]

Mouse_Zebra.conserved_frac[order(percetage)]

Mouse_Zebra.conserved_frac <- merge(Mouse_Zebra.conserved_frac, ME_cluster_names[, c("ME_cluster.name", "ME_cluster.type")], by="ME_cluster.name" )

Mouse_Zebra.conserved_frac[, broad_class:="Other"]
Mouse_Zebra.conserved_frac[ME_cluster.type %in% c("Neuronal", "Neuro-Muscular", "Non-Neuronal", "Weak-Neuronal"), broad_class:="Neuronal"]
Mouse_Zebra.conserved_frac[ME_cluster.type %in% c("Neuro-Muscular"), broad_class:="Neuro-Muscular"]

Fig7.D <- ggplot(Mouse_Zebra.conserved_frac, aes(x = broad_class,  y = percetage )) +
  geom_jitter( aes( colour = ME_cluster.type), width=0.1) +
  geom_signif(comparisons = list(c("Neuronal", "Other")), map_signif_level=TRUE, test="wilcox.test", margin_top=5,  y_position=35, test.args = list(alternative = "two.sided", paired = FALSE)) +
  xlab("Microexon cluster with neuronal pattern") +
  ylab("Percentage of conserved microexons") +
  theme_bw() + 
  theme(axis.text.x = element_text(angle = 90))

Fig7.D
```


Exploring lift zebra that do not overlap with mouse microexons


```
zebra.total_lift <- fread("../../Zebrafish/Conserved/out.high_quality.bed.liftover_0.001_mm10")

zebra.total_lift[!V4 %in%  zebra_to_mouse$V4, ]
```


```
plot_grid(Fig7.D, Fig7.E, ncol=1)
```


#### Zebra diff


```
diff.W.file <-  "../../Zebrafish/Final_Report/Whippet/Delta/16Mo_vs_4Mo-TOTAL.diff.microexons"
diff.ME.file <- "../../Zebrafish/Final_Report/Whippet/Delta/16Mo_vs_4Mo-TOTAL.diff.ME.microexons"
comp_name = "16Mo_vs_4Mo-TOTAL"


diff_zebra <- function(diff.W.file, diff.ME.file, comp_name) {


diff.W <- fread(diff.W.file)
diff.ME <- fread(diff.ME.file)

diff.WME <- merge(diff.W, diff.ME, by=c("exon_ID", "Gene", "Node",  "Coord", "Strand", "Type"))

diff.WME[, Name:=comp_name]

diff.WME[ , diff:="NA"]
diff.WME[ (DeltaPsi.x>=0.1 &  Probability.x >= 0.9) &  (DeltaPsi.y>=0.1 &  Probability.y >= 0.9), diff:="Included"]
diff.WME[ (DeltaPsi.x<=-0.1 &  Probability.x >= 0.9) &  (DeltaPsi.y<=-0.1 &  Probability.y >= 0.9)  , diff:="Excluded" ]

return(diff.WME)
}
```


```
Zebra.16Mo_vs_4Mo.TOTAL <- diff_zebra("../../Zebrafish/Final_Report/Whippet/Delta/16Mo_vs_4Mo-TOTAL.diff.microexons",
                                "../../Zebrafish/Final_Report/Whippet/Delta/16Mo_vs_4Mo-TOTAL.diff.ME.microexons", 
                                "16Mo_vs_4Mo-TOTAL")


Zebra.20Mo_vs_16Mo.ZT16 <- diff_zebra("../../Zebrafish/Final_Report/Whippet/Delta/20Mo_vs_16Mo-ZT16.diff.microexons",
                                "../../Zebrafish/Final_Report/Whippet/Delta/20Mo_vs_16Mo-ZT16.diff.ME.microexons", 
                                "20Mo_vs_16Mo-ZT16")


Zebra.20Mo_vs_4M.ZT16 <- diff_zebra("../../Zebrafish/Final_Report/Whippet/Delta/20Mo_vs_4M-ZT16.diff.microexons",
                                "../../Zebrafish/Final_Report/Whippet/Delta/20Mo_vs_4M-ZT16.diff.ME.microexons", 
                                "20Mo_vs_4M-ZT16")


Zebra.16Mo_vs_4Mo.ZT16 <- diff_zebra("../../Zebrafish/Final_Report/Whippet/Delta/16Mo_vs_4Mo-ZT16.diff.microexons",
                                "../../Zebrafish/Final_Report/Whippet/Delta/16Mo_vs_4Mo-ZT16.diff.ME.microexons", 
                                "16Mo_vs_4Mo-ZT16")


Zebra.20Mo_vs_16Mo.ZT4 <- diff_zebra("../../Zebrafish/Final_Report/Whippet/Delta/20Mo_vs_16Mo-ZT4.diff.microexons",
                                "../../Zebrafish/Final_Report/Whippet/Delta/20Mo_vs_16Mo-ZT4.diff.ME.microexons", 
                                "20Mo_vs_16Mo-ZT4")


Zebra.20Mo_vs_4M.ZT4 <- diff_zebra("../../Zebrafish/Final_Report/Whippet/Delta/20Mo_vs_4M-ZT4.diff.microexons",
                                "../../Zebrafish/Final_Report/Whippet/Delta/20Mo_vs_4M-ZT4.diff.ME.microexons", 
                                "20Mo_vs_4M-ZT4")


Zebra.16Mo_vs_4Mo.ZT4 <- diff_zebra("../../Zebrafish/Final_Report/Whippet/Delta/16Mo_vs_4Mo-ZT4.diff.microexons",
                                "../../Zebrafish/Final_Report/Whippet/Delta/16Mo_vs_4Mo-ZT4.diff.ME.microexons", 
                                "16Mo_vs_4Mo-ZT4")


Zebra.ZT16_vs_ZT4.20M <- diff_zebra("../../Zebrafish/Final_Report/Whippet/Delta/ZT16_vs_ZT4-20Mo.diff.microexons",
                                "../../Zebrafish/Final_Report/Whippet/Delta/ZT16_vs_ZT4-20Mo.diff.ME.microexons", 
                                "ZT16_vs_ZT4-20Mo")

Zebra.ZT16_vs_ZT4.16M <- diff_zebra("../../Zebrafish/Final_Report/Whippet/Delta/ZT16_vs_ZT4-16Mo.diff.microexons",
                                "../../Zebrafish/Final_Report/Whippet/Delta/ZT16_vs_ZT4-16Mo.diff.ME.microexons", 
                                "ZT16_vs_ZT4-16Mo")

Zebra.ZT16_vs_ZT4.4M <- diff_zebra("../../Zebrafish/Final_Report/Whippet/Delta/ZT16_vs_ZT4-4Mo.diff.microexons",
                                "../../Zebrafish/Final_Report/Whippet/Delta/ZT16_vs_ZT4-4Mo.diff.ME.microexons", 
                                "ZT16_vs_ZT4-4Mo")


Zebra.TOTAL_diff <- rbind(
Zebra.20Mo_vs_16Mo.ZT16[diff!="NA"],
Zebra.20Mo_vs_4M.ZT16[diff!="NA"],
Zebra.16Mo_vs_4Mo.ZT16[diff!="NA"],
Zebra.20Mo_vs_16Mo.ZT4[diff!="NA"],
Zebra.20Mo_vs_4M.ZT4[diff!="NA"],
Zebra.16Mo_vs_4Mo.ZT4[diff!="NA"],
Zebra.ZT16_vs_ZT4.20M[diff!="NA"],
Zebra.ZT16_vs_ZT4.16M[diff!="NA"],
Zebra.ZT16_vs_ZT4.4M[diff!="NA"] )


Zebra.TOTAL_diff <- Zebra.TOTAL_diff[ , lapply(.SD, paste0, collapse=",") , by="exon_ID"]


Mouse_Zebra.IDs[ME.zebra %in% Zebra.TOTAL_diff$exon_ID]
```


```
Delta_HNM_whippet_ME <- merge(Delta_HNM_whippet, Delta_HNM_ME, by=c("exon_ID", "Gene", "Node",  "Coord", "Strand", "Type", "age") )

Delta_HNM_whippet_ME[ (abs(DeltaPsi.x)>=0.1 &  Probability.x >= 0.9) &  (abs(DeltaPsi.y)>=0.1 &  Probability.y >= 0.9) , .N , by=(age)]

Delta_HNM_whippet_ME_included <- Delta_HNM_whippet_ME[ (DeltaPsi.x<=-0.1 &  Probability.x >= 0.9) &  (DeltaPsi.y<=-0.1 &  Probability.y >= 0.9)  , .N , by=(age)]
Delta_HNM_whippet_ME_excluded <- Delta_HNM_whippet_ME[ (DeltaPsi.x>=0.1 &  Probability.x >= 0.9) &  (DeltaPsi.y>=0.1 &  Probability.y >= 0.9)  , .N , by=(age)]


Delta_HNM_included_stats <- cbind(Delta_HNM_whippet_included[order(age)], Delta_HNM_ME_included[order(age), 2], Delta_HNM_whippet_ME_included[order(age), 2])
colnames(Delta_HNM_included_stats) <- c("age", "Whippet", "MicroExonator", "Both")
```


##### 


```
centrality_logistic_regression_HC <- glm(formula = central ~ min_dif_age, family = "binomial", data = PPI_diff_age_HNM_stats_HC)
summary(centrality_logistic_regression_HC)


centrality_logistic_regression_EC <- glm(formula = central ~ min_dif_age, family = "binomial", data = PPI_diff_age_HNM_stats_EC)
summary(centrality_logistic_regression_EC)


centrality_logistic_regression_B <- glm(formula = central ~ min_dif_age, family = "binomial", data = PPI_diff_age_HNM_stats_B)
summary(centrality_logistic_regression_B)
```


```
PPI_diff_age_HNM$L1cam_and_interactors <- factor(PPI_diff_age_HNM$L1cam_and_interactors, levels=c(TRUE, FALSE))


F6G <- ggplot(PPI_diff_age_HNM) +
  geom_point(aes(eigen_centrality, betweenness, colour=L1cam_and_interactors)) +
  facet_wrap( . ~ diff_age, ncol = 2)  +
      geom_text_repel(data=PPI_diff_age_HNM[ L1cam_and_interactors==TRUE,  ], 
                  aes(x=eigen_centrality, y=betweenness, colour=L1cam_and_interactors ), 
                  nudge_y      = 3000,
                  direction    = "x",
                  angle        = 90,
                  segment.size = 0.2,
                  label=PPI_diff_age_HNM[L1cam_and_interactors==TRUE  , mgi_symbol],
                  show.legend = FALSE) +
          theme(axis.text.x = element_text(angle = 90), legend.position = "top", legend.direction = "horizontal") +
    scale_fill_discrete(name = "L1CAM pathway", labels = c("Member", "Non-member"))
```


```
F6G <- ggplot(PPI_diff_age_HNM) +
  geom_point(aes(eigen_centrality, betweenness, colour=L1cam_and_interactors)) +
  facet_wrap( . ~ diff_age, ncol = 2)  +
      geom_text_repel(data=PPI_diff_age_HNM[ L1cam_and_interactors==TRUE,  ], 
                  aes(x=eigen_centrality, y=betweenness, colour=L1cam_and_interactors ), 
                  nudge_y      = 3000,
                  direction    = "x",
                  angle        = 90,
                  segment.size = 0.2,
                  label=PPI_diff_age_HNM[L1cam_and_interactors==TRUE  , mgi_symbol],
                  show.legend = FALSE) +
          theme(axis.text.x = element_text(angle = 90), legend.position = "top", legend.direction = "horizontal") +
    scale_fill_discrete(name = "L1CAM pathway", labels = c("Member", "Non-member"))
```


```
Error in ggplot(PPI_diff_age_HNM) : object 'PPI_diff_age_HNM' not found
```


```
 ggplot(PPI_diff_age_HNM) +
  geom_jitter(aes(log(betweenness), diff_age, color=type ), width=0, height=0.1) +
  xlab("Betweenness") +
  ylab("Earliest microexon differential inclusion detection (DPC)") +
   scale_y_continuous(breaks=seq(10.5,16.5,1)) +
    geom_text_repel(data=PPI_diff_age_HNM[  log(betweenness)>6,  ],
                  aes(x=log(betweenness), y=diff_age, colour=type), 
                  nudge_y      = 0.5,
                  direction    = "x",
                  angle        = 90,
                  segment.size = 0.2,
                  show.legend = FALSE,
                  label=PPI_diff_age_HNM[log(betweenness)>6  , mgi_symbol] )
```

## Tasic


```
ensembl = useEnsembl(biomart="ensembl", dataset="mmusculus_gene_ensembl", host = "www.ensembl.org")


ME_final.transcript <- gsub("\\..*","", ME_final$transcript)

ME_final$ensembl_transcript_id <- gsub("\\..*","", ME_final$transcript) 

gene_table.TOTAL <- data.table(getBM(attributes=c('ensembl_transcript_id', 'ensembl_gene_id', "mgi_symbol"),filters = 'ensembl_transcript_id', values = ME_final.transcript[nchar(ME_final.transcript)!=0], mart = ensembl,  ))


gene_table.TOTAL <- merge(gene_table.TOTAL, ME_final[, c("ensembl_transcript_id", "ME")], by="ensembl_transcript_id")
```


```
ensembl = useEnsembl(biomart="ensembl", dataset="mmusculus_gene_ensembl", host = "www.ensembl.org")
ME_final.transcript <- gsub("\\..*","", ME_final$transcript)
ME_final$ensembl_transcript_id <- gsub("\\..*","", ME_final$transcript) 
gene_table.TOTAL <- data.table(getBM(attributes=c('ensembl_transcript_id', 'ensembl_gene_id', "mgi_symbol"),filters = 'ensembl_transcript_id', values = ME_final.transcript[nchar(ME_final.transcript)!=0], mart = ensembl,  ))
```


```
Batch submitting query [==================================>----------------------------------------------------------------------------------------]  29% eta: 26s
Batch submitting query [====================================================>----------------------------------------------------------------------]  43% eta: 22s
Batch submitting query [=====================================================================>-----------------------------------------------------]  57% eta: 16s
Batch submitting query [=======================================================================================>-----------------------------------]  71% eta: 11s
Batch submitting query [========================================================================================================>------------------]  86% eta:  5s
Batch submitting query [===========================================================================================================================] 100% eta:  0s
```


```
gene_table.TOTAL <- merge(gene_table.TOTAL, ME_final[, c("ensembl_transcript_id", "ME")], by="ensembl_transcript_id")
```


```
ME_clusters.info <- merge(PCA_loadings, ME_cluster_names, by="ME_cluster")
```


```
Error in bmerge(i, x, leftcols, rightcols, roll, rollends, nomatch, mult,  : 
  Incompatible join types: x.ME_cluster (integer) and i.ME_cluster (factor). Factor columns must join to factor or character columns.
```


```
#C7_interesting <- c("chr1_-_93026831_93026855", "chr1_+_118512704_118512728", "chr11_-_106180954_106180983", "chr11_-_3352899_3352917", "chr11_+_54671654_54671678", "chr3_-_148827211_148827238"," chr4_-_76138555_76138573", "chr5_+_143703372_143703375", "chr5_+_48263640_48263667", "chr8_-_54645337_54645358")


#ggplot(ME_cov_filtered_tasic_primary_type[ ME %in% C7_interesting, ]  ) +
#  geom_pointrange(aes(x=primary_type, y=PSI, colour= broad_type, group=broad_type, shape=broad_type, ymin=lower, ymax=upper))+
#  theme(axis.text.x = element_text(angle = 90, hjust = 0, vjust = 0.5)) +
#  facet_grid( gene_names[ME] ~ . )


target_genes <- c("Kif1a", "Dctn1", "Kdm1a", "Kdm2a",  "Itsn1", "Dnm1", "Dnm3", "Trrap", "Dclk2")

target_genes <- c("Kif1a", "Dctn1", "Kdm1a", "Kdm2a",  "Itsn1", "Dnm1", "Dnm3", "Trrap", "Dclk2")


ggplot(ME_cov_filtered_tasic_primary_type[ mgi_symbol %in% target_genes &  ME %in% Delta_HNMF_whippet_ME_age_diff_total$exon_ID , ]  ) +
  geom_pointrange(aes(x=primary_type, y=PSI, colour= broad_type, group=broad_type, shape=broad_type, ymin=lower, ymax=upper))+
  facet_grid( paste0( mgi_symbol, ME) ~ . ) +
  theme_bw() +
  theme(axis.text.x = element_text(angle = 90, hjust = 0, vjust = 0.5))


ggplot(ME_cov_filtered_tasic_primary_type[ mgi_symbol %in% target_genes, ]  ) +
  geom_pointrange(aes(x=primary_type, y=PSI, colour= broad_type, group=broad_type, shape=broad_type, ymin=lower, ymax=upper))+
  facet_grid( paste0( mgi_symbol, ME) ~ . ) +
  theme_bw() +
  theme(axis.text.x = element_text(angle = 90, hjust = 0, vjust = 0.5))
```


######

## New Tasic


```
Tasic_total_diff_nodes <- fread("/Users/gp7/Google_Drive/Results/ME/Paper/Single_cell/GABA-ergic_Neuron_vs_Glutamatergic_Neuron.all_nodes.microexons.txt")


Tasic_total_diff_nodes.microexons <- merge( Tasic_total_diff_nodes[is.diff=="TRUE" & !is.na(microexon_ID), ], 
                                            unique(gene_table.TOTAL[, c("ME", "mgi_symbol")]),
                                            by.x="microexon_ID", by.y="ME")

Tasic_total_diff_nodes.microexons[!microexon_ID %in% Sig_GABAergic_Neuron_vs_Glutamatergic_Neuron$exon_ID, ]
```


```
Tasic_total_diff_nodes <- fread("/Users/gp7/Google_Drive/Results/ME/Paper/Single_cell/GABA-ergic_Neuron_vs_Glutamatergic_Neuron.all_nodes.microexons.txt")
Tasic_total_diff_nodes.microexons <- merge( Tasic_total_diff_nodes[is.diff=="TRUE" & !is.na(microexon_ID), ], 
                                            unique(gene_table.TOTAL[, c("ME", "mgi_symbol")]),
                                            by.x="microexon_ID", by.y="ME")
Tasic_total_diff_nodes.microexons[!microexon_ID %in% Sig_GABAergic_Neuron_vs_Glutamatergic_Neuron$exon_ID, ]
```


```
                  microexon_ID                  Gene Node                     Coord Strand Type Psi_A.mean Psi_B.mean DeltaPsi.mean DeltaPsi.sd Probability.mean
1: chr10_+_127728446_127728476 ENSMUSG00000025400.11    9 chr10:127728447-127728476      +   CE  0.9988179  0.8949054     0.1039116 0.005232909        0.9864583
2:    chr2_+_76569019_76569043 ENSMUSG00000042359.18   25    chr2:76569020-76569043      +   CE  0.4229608  0.9362494    -0.5132894 0.029304946        0.9067200
   Probability.sd Probability.var N.detected.reps     cdf.beta is.diff mgi_symbol
1:    0.005295355    2.804078e-05              48 8.007671e-38    TRUE       Tac2
2:    0.026344685    6.940424e-04              50 5.305384e-04    TRUE     Osbpl6
```


```
#ref Slc4a10 https://www.frontiersin.org/articles/10.3389/fncel.2015.00223/full
#ref Ank3 https://www.sciencedirect.com/science/article/pii/S0896627314009088
#ref KCNMA1 https://www.sciencedirect.com/science/article/pii/S0896627313001852
#ref Kif3a https://www.sciencedirect.com/science/article/pii/S089662730300062X
# ref DLGAP https://molecularbrain.biomedcentral.com/articles/10.1186/s13041-017-0324-9
Pre_synaptic <- c( "Ptprd" , "Ppfia2", "Dlgap1", "Gabrg2", "Kcnma1", "Kif3a", "Cadps") 
Post_synaptic <- c( "Nrxn3" , "Nrxn1")
PrePost_synaptic <- c("Ank3","Slc4a10")
```


```
Tasic_total_diff_nodes.microexons
```


```
                   microexon_ID                  Gene Node                     Coord Strand Type Psi_A.mean Psi_B.mean DeltaPsi.mean DeltaPsi.sd Probability.mean
 1: chr10_+_106882735_106882744 ENSMUSG00000053825.15   26 chr10:106882736-106882744      +   CE 0.04254298 0.40015960    -0.3576170 0.013543752        0.9423000
 2: chr10_+_127728446_127728476 ENSMUSG00000025400.11    9 chr10:127728447-127728476      +   CE 0.99881792 0.89490542     0.1039116 0.005232909        0.9864583
 3:   chr10_+_59767083_59767089 ENSMUSG00000020111.15   21   chr10:59767084-59767089      +   CE 0.50434320 0.90624060    -0.4018980 0.014786698        0.9524600
 4:   chr10_+_69980823_69980850 ENSMUSG00000069601.14   87   chr10:69980824-69980850      +   CE 0.83555840 0.14347140     0.6920866 0.020767159        0.9713200
 5:   chr11_+_53590716_53590725 ENSMUSG00000018395.19   21   chr11:53590717-53590725      +   CE 0.66200620 0.22252240     0.4394834 0.007520063        0.9907000
 6: chr11_-_112680921_112680950 ENSMUSG00000041674.16   31 chr11:112680922-112680950      -   CE 0.91463532 0.13275472     0.7818815 0.028238359        0.9804894
 7:   chr11_-_41913969_41913993 ENSMUSG00000020436.17   13   chr11:41913970-41913993      -   CE 0.78832760 0.49438140     0.2939452 0.003662291        0.9883000
 8:   chr12_+_89354452_89354479 ENSMUSG00000066392.11   28   chr12:89354453-89354479      +   CE 0.61528060 0.99319860    -0.3779194 0.015058715        0.9912800
 9: chr12_-_100340496_100340499  ENSMUSG00000033530.8   33 chr12:100340497-100340499      -   CE 0.08897032 0.45795740    -0.3689868 0.015599731        0.9196800
10:   chr14_-_12472397_12472406 ENSMUSG00000054423.14   37   chr14:12472398-12472406      -   CE 0.67774640 0.25092260     0.4268242 0.011500528        0.9458400
11:   chr14_-_23449199_23449208 ENSMUSG00000063142.15   37   chr14:23449200-23449208      -   CE 0.12223500 0.79508280    -0.6728472 0.011630571        0.9913000
12:   chr14_-_23449199_23449211 ENSMUSG00000063142.15   36   chr14:23449209-23449211      -   AA 0.12223200 0.79455880    -0.6723272 0.011685279        0.9914800
13:   chr16_+_58729215_58729223 ENSMUSG00000022744.12    9   chr16:58729216-58729223      +   CE 0.84074200 0.48995680     0.3507854 0.005506084        0.9701000
14:   chr17_+_70718195_70718225 ENSMUSG00000003279.16   29   chr17:70718196-70718225      +   CE 0.55267380 0.94271520    -0.3900420 0.010741757        0.9834200
15:   chr17_-_90561801_90561828 ENSMUSG00000024109.18   40   chr17:90561802-90561828      -   CE 0.05202180 0.42399920    -0.3719784 0.011344171        0.9733200
16:   chr17_-_90620864_90620891 ENSMUSG00000024109.18   30   chr17:90620865-90620891      -   CE 0.47302620 0.99741340    -0.5243858 0.024613597        0.9946200
17:    chr1_-_51786572_51786578 ENSMUSG00000018417.14   30    chr1:51786573-51786578      -   CE 0.28201560 0.90874460    -0.6267280 0.027523168        0.9336200
18:    chr2_+_62317497_62317514 ENSMUSG00000026904.17   35    chr2:62317498-62317514      +   CE 0.79882300 0.97748240    -0.1786596 0.005950109        0.9637600
19:    chr2_+_76569019_76569043 ENSMUSG00000042359.18   25    chr2:76569020-76569043      +   CE 0.42296080 0.93624940    -0.5132894 0.029304946        0.9067200
20:    chr3_+_69724256_69724270 ENSMUSG00000027787.14    8    chr3:69724257-69724270      +   CE 0.97109880 0.76964180     0.2014566 0.006564793        0.9468600
21:    chr4_-_76054588_76054600 ENSMUSG00000028399.18   71    chr4:76054589-76054600      -   CE 0.80153800 0.41104260     0.3904962 0.011873125        0.9194800
22:    chr4_-_76138555_76138573 ENSMUSG00000028399.18   37    chr4:76138556-76138573      -   CE 0.17602620 0.98994200    -0.8139158 0.021469308        0.9953000
23:    chr4_-_76139293_76139302 ENSMUSG00000028399.18   36    chr4:76139294-76139302      -   CE 0.07221076 0.92935940    -0.8571476 0.008669427        0.9976400
24:    chr5_+_48263640_48263667 ENSMUSG00000031558.15   66    chr5:48263641-48263667      +   CE 0.78063060 0.04944636     0.7311840 0.023120612        0.9710400
25:    chr5_-_73050866_73050875 ENSMUSG00000070733.13   85    chr5:73050867-73050875      -   CE 0.85641940 0.08213788     0.7742824 0.016144319        0.9724600
26:    chr7_+_75696321_75696338 ENSMUSG00000066406.15   37    chr7:75696322-75696338      +   CE 0.50410580 0.93627240    -0.4321666 0.027574986        0.9034200
27:  chr9_-_105493542_105493554 ENSMUSG00000032570.17   18  chr9:105493543-105493554      -   CE 0.31801920 0.93770440    -0.6196862 0.025207937        0.9588800
28:    chrX_+_71376886_71376910 ENSMUSG00000015214.14    5    chrX:71376887-71376910      +   CE 0.07470168 0.94029960    -0.8655980 0.010244770        0.9999800
29:  chrX_-_155214830_155214858 ENSMUSG00000025283.15   13  chrX:155214831-155214858      -   CE 0.25803900 0.47274340    -0.2147052 0.005831721        0.9018000
                   microexon_ID                  Gene Node                     Coord Strand Type Psi_A.mean Psi_B.mean DeltaPsi.mean DeltaPsi.sd Probability.mean
    Probability.sd Probability.var N.detected.reps      cdf.beta is.diff mgi_symbol     Location
 1:   0.0131028117    1.716837e-04              50  1.724799e-13    TRUE     Ppfia2  Presynaptic
 2:   0.0052953546    2.804078e-05              48  8.007671e-38    TRUE       Tac2         <NA>
 3:   0.0149054298    2.221718e-04              50  9.056338e-11    TRUE      Micu1         <NA>
 4:   0.0118484305    1.403853e-04              50  1.093610e-13    TRUE       Ank3         Both
 5:   0.0058458078    3.417347e-05              50  4.859664e-24    TRUE      Kif3a  Presynaptic
 6:   0.0129478424    1.676466e-04              47  7.190604e-10    TRUE   BC006965         <NA>
 7:   0.0056757630    3.221429e-05              50  5.694298e-30    TRUE     Gabrg2  Presynaptic
 8:   0.0057783551    3.338939e-05              50  2.074737e-23    TRUE      Nrxn3 Postsynaptic
 9:   0.0145116983    2.105894e-04              50  2.114484e-10    TRUE      Ttc7b         <NA>
10:   0.0147832779    2.185453e-04              50  5.108218e-11    TRUE      Cadps  Presynaptic
11:   0.0060684867    3.682653e-05              50  2.067205e-21    TRUE     Kcnma1  Presynaptic
12:   0.0063317793    4.009143e-05              50  1.461345e-19    TRUE     Kcnma1  Presynaptic
13:   0.0096895694    9.388776e-05              50  8.572693e-20    TRUE     Cldnd1         <NA>
14:   0.0092517842    8.559551e-05              50  9.431345e-16    TRUE     Dlgap1  Presynaptic
15:   0.0140006414    1.960180e-04              50  4.500230e-10    TRUE      Nrxn1 Postsynaptic
16:   0.0060132167    3.615878e-05              50  2.836469e-15    TRUE      Nrxn1 Postsynaptic
17:   0.0155404541    2.415057e-04              50  5.886836e-10    TRUE      Myo1b         <NA>
18:   0.0108035519    1.167167e-04              50  1.513312e-17    TRUE    Slc4a10         Both
19:   0.0263446854    6.940424e-04              50  5.305384e-04    TRUE     Osbpl6         <NA>
20:   0.0105907353    1.121637e-04              50  1.320554e-19    TRUE       Nmd3         <NA>
21:   0.0168925053    2.853567e-04              50  3.888772e-08    TRUE      Ptprd  Presynaptic
22:   0.0048075196    2.311224e-05              50  2.975151e-20    TRUE      Ptprd  Presynaptic
23:   0.0022835704    5.214694e-06              50  3.830921e-44    TRUE      Ptprd  Presynaptic
24:   0.0167891607    2.818759e-04              50  7.775889e-08    TRUE      Slit2         <NA>
25:   0.0154195873    2.377637e-04              50  9.252242e-09    TRUE       Fryl         <NA>
26:   0.0190135183    3.615139e-04              50  5.180120e-06    TRUE     Akap13         <NA>
27:   0.0152992463    2.340669e-04              50  4.602062e-10    TRUE     Atp2c1         <NA>
28:   0.0001414214    2.000000e-08              50 1.737462e-101    TRUE      Mtmr1         <NA>
29:   0.0184777859    3.414286e-04              50  3.573953e-06    TRUE       Sat1         <NA>
    Probability.sd Probability.var N.detected.reps      cdf.beta is.diff mgi_symbol     Location
```


```
ME_clusters.info <- merge(PCA_loadings, ME_cluster_names, by="ME_cluster")
```


```
Error in bmerge(i, x, leftcols, rightcols, roll, rollends, nomatch, mult,  : 
  Incompatible join types: x.ME_cluster (integer) and i.ME_cluster (factor). Factor columns must join to factor or character columns.
```


```
primary_type_levels <- c(unique(ME_cov_filtered_tasic_primary_type[broad_type=="Endothelial Cell", primary_type]),
unique(ME_cov_filtered_tasic_primary_type[broad_type=="Oligodendrocyte", primary_type]),
unique(ME_cov_filtered_tasic_primary_type[broad_type=="Microglia", primary_type]),
unique(ME_cov_filtered_tasic_primary_type[broad_type=="Oligodendrocyte Precursor Cell", primary_type]),
sort(unique(ME_cov_filtered_tasic_primary_type[broad_type=="Astrocyte", primary_type])),
sort(unique(ME_cov_filtered_tasic_primary_type[broad_type=="Glutamatergic Neuron", primary_type])),
unique(ME_cov_filtered_tasic_primary_type[broad_type=="GABA-ergic Neuron", primary_type]) )
ME_cov_filtered_tasic_primary_type$primary_type <- factor(ME_cov_filtered_tasic_primary_type$primary_type , levels = primary_type_levels)
```


```
target_genes <- c("Gabrg2", "Nrxn1", "Nrxn3", "Ptprd")
ME_cov_filtered_tasic_primary_type[(mgi_symbol %in% target_genes) & (ME %in% Sig_GABAergic_Neuron_vs_Glutamatergic_Neuron$exon_ID)]
```


```
                            ME primary_type           broad_type sum_ME_coverage sum_SJ_coverage total_cov_alternatives_3 total_cov_alternatives_5       PSI     lower
  1: chr11_-_41913969_41913993   Vip Mybpc1    GABA-ergic Neuron            5881             887                        0                        0 0.8689421 0.8606926
  2: chr11_-_41913969_41913993    Vip Parm1    GABA-ergic Neuron            5465            3692                        0                        0 0.5968112 0.5867254
  3: chr11_-_41913969_41913993     L4 Ctxn3 Glutamatergic Neuron           12660            7816                        0                        0 0.6182848 0.6116091
  4: chr11_-_41913969_41913993     Vip Chat    GABA-ergic Neuron            5390            3757                        0                        0 0.5892642 0.5791469
  5: chr11_-_41913969_41913993   L2/3 Ptgs2 Glutamatergic Neuron            8799            3910                        0                        0 0.6923440 0.6842630
 ---                                                                                                                                                                  
339:  chr4_-_76139293_76139302  Pvalb Obox3    GABA-ergic Neuron               0               5                        0                        0 0.0000000 0.0000000
340:  chr4_-_76139293_76139302     L5b Tph2 Glutamatergic Neuron               3               0                        0                        0       NaN        NA
341:  chr4_-_76139293_76139302      L5 Ucma Glutamatergic Neuron              10               0                        0                        0 1.0000000 0.7224672
342:  chr4_-_76139293_76139302    L5 Chrna6 Glutamatergic Neuron               3               0                        0                        0       NaN        NA
343:  chr4_-_76139293_76139302    L5b Cdh13 Glutamatergic Neuron              19               0                        0                        0 1.0000000 0.8318208
         upper ensembl_transcript_id    ensembl_gene_id mgi_symbol
  1: 0.8767730    ENSMUST00000070725 ENSMUSG00000020436     Gabrg2
  2: 0.6068157    ENSMUST00000070725 ENSMUSG00000020436     Gabrg2
  3: 0.6249162    ENSMUST00000070725 ENSMUSG00000020436     Gabrg2
  4: 0.5993067    ENSMUST00000070725 ENSMUSG00000020436     Gabrg2
  5: 0.7003088    ENSMUST00000070725 ENSMUSG00000020436     Gabrg2
 ---                                                              
339: 0.4344825    ENSMUST00000174180 ENSMUSG00000028399      Ptprd
340:        NA    ENSMUST00000174180 ENSMUSG00000028399      Ptprd
341: 1.0000000    ENSMUST00000174180 ENSMUSG00000028399      Ptprd
342:        NA    ENSMUST00000174180 ENSMUSG00000028399      Ptprd
343: 1.0000000    ENSMUST00000174180 ENSMUSG00000028399      Ptprd
```


```
ME_cov_filtered_tasic_primary_type[is.na(PSI), `:=`(lower=NA, upper=NA) ]
Fig8.c.top <- ggplot(ME_cov_filtered_tasic_primary_type[ mgi_symbol %in% target_genes & ME %in% Sig_GABAergic_Neuron_vs_Glutamatergic_Neuron$exon_ID, ]  ) +
  geom_pointrange(aes(x=primary_type, y=PSI, colour= broad_type, group=broad_type, ymin=lower, ymax=upper))+
  facet_grid( paste0( mgi_symbol, ME) ~ . ) +
  theme_bw() +
  theme(axis.text.x = element_text(angle = 90, hjust = 0, vjust = 0.5)) +
  theme(axis.text.x = element_text(angle = 90), legend.position = "top", legend.direction = "horizontal") +
  theme(axis.title.x=element_blank(),
        axis.text.x=element_blank(),
        axis.ticks.x=element_blank())
Fig8.c.top
```


```
Tasic_clustering$primary_type <- factor(Tasic_clustering$primary_type , levels = primary_type_levels)

Fig8.c.bottom <- ggplot(Tasic_clustering[ broad_type!="Unclassified", .N , by=c("primary_type", "broad_type") ]) +
  geom_bar( aes(x=primary_type, y=N,  fill=broad_type) , stat="identity") +
    theme_bw() +
  theme(axis.text.x = element_text(angle = 90, hjust = 0, vjust = 0.5)) +
  theme(axis.text.x = element_text(angle = 90), legend.position = "none", legend.direction = "horizontal", panel.grid.major = element_blank(), panel.grid.minor = element_blank())

Fig8.c.bottom
```


##### 

Single cell stast


```
plot_grid(Fig8.c.top, Fig8.c.bottom, ncol=1, rel_heights = c(7,2) )
```


```
Removed 65 rows containing missing values (geom_pointrange).
```


```
nrow(Tasic_total_diff_nodes)
```


```
[1] 195441
```


```
table(Tasic_total_diff_nodes$is.microexon)
```


```
 FALSE   TRUE 
193176   2265
```


```
Tasic_total_diff_nodes[  , is.microexon:=!is.na(microexon_ID) ]
Tasic_total_diff_nodes[ is.na(is.diff) , is.diff:="FALSE"  ]
```


```
Coercing 'character' RHS to 'logical' to match the type of the target column (column 15 named 'is.diff').
```


```
Tasic_total_diff_nodes.stats <- Tasic_total_diff_nodes[ , .N , by=c("Type", "is.diff", "is.microexon")]
Tasic_total_diff_nodes.stats[ , Total:=sum(N), by=c("Type", "is.microexon")]
Tasic_total_diff_nodes.stats[is.diff=="TRUE", ]
```


```
   Type is.diff is.microexon  N  Total
1:   AF    TRUE        FALSE 36   4121
2:   CE    TRUE        FALSE 97 100168
3:   CE    TRUE         TRUE 28   2204
4:   AD    TRUE        FALSE 13   4749
5:   RI    TRUE        FALSE 18   7342
6:   AL    TRUE        FALSE  3   1708
7:   AA    TRUE        FALSE 12   5772
8:   AA    TRUE         TRUE  1     50
```


```
Tasic_total_diff_nodes.stats[, ratio:=N/Total]
Tasic_total_diff_nodes.stats <- Tasic_total_diff_nodes.stats[is.diff=="TRUE", ]
Tasic_total_diff_nodes.stats[is.diff=="TRUE", ]
```


```
   Type is.diff is.microexon  N  Total        ratio
1:   AF    TRUE        FALSE 36   4121 0.0087357438
2:   CE    TRUE        FALSE 97 100168 0.0009683731
3:   CE    TRUE         TRUE 28   2204 0.0127041742
4:   AD    TRUE        FALSE 13   4749 0.0027374184
5:   RI    TRUE        FALSE 18   7342 0.0024516481
6:   AL    TRUE        FALSE  3   1708 0.0017564403
7:   AA    TRUE        FALSE 12   5772 0.0020790021
8:   AA    TRUE         TRUE  1     50 0.0200000000
```


```
CE.cont <- matrix(nrow=2, c(Tasic_total_diff_nodes.stats[Type=="CE" & is.microexon=="TRUE", N ],
Tasic_total_diff_nodes.stats[Type=="CE" & is.microexon=="TRUE", Total-N ],
Tasic_total_diff_nodes.stats[Type=="CE" & is.microexon=="FALSE", N ],
Tasic_total_diff_nodes.stats[Type=="CE" & is.microexon=="FALSE", Total-N ]))
chisq.test(CE.cont)
```


```
Chi-squared approximation may be incorrect
```


```
    Pearson's Chi-squared test with Yates' continuity correction

data:  CE.cont
X-squared = 234.02, df = 1, p-value < 2.2e-16
```


```
CE.cont.total <- matrix(nrow=2, c(sum(Tasic_total_diff_nodes.stats[ is.microexon=="TRUE", N ]),
sum(Tasic_total_diff_nodes.stats[ is.microexon=="TRUE", Total-N ]),
sum(Tasic_total_diff_nodes.stats[is.microexon=="FALSE", N ]),
sum(Tasic_total_diff_nodes.stats[ is.microexon=="FALSE", Total-N ])))
chisq.test(CE.cont.total)
```


```
Chi-squared approximation may be incorrect
```


```
    Pearson's Chi-squared test with Yates' continuity correction

data:  CE.cont.total
X-squared = 168.49, df = 1, p-value < 2.2e-16
```


```
n=nrow(Tasic_total_diff_nodes)
a=nrow(Tasic_total_diff_nodes[ is.diff=="TRUE", ])
b=nrow(Tasic_total_diff_nodes[ is.microexon=="TRUE", ])
t=nrow(Tasic_total_diff_nodes[ is.microexon=="TRUE" &  is.diff=="TRUE", ])
dhyper(t, a, n - a, b)
```


```
[1] 1.875713e-22
```


```
sum(dhyper(t:b, a, n - a, b))
```


```
[1] 2.014586e-22
```


```
n=nrow(Tasic_total_diff_nodes[Type=="CE", ])
a=nrow(Tasic_total_diff_nodes[Type=="CE" & is.diff=="TRUE", ])
b=nrow(Tasic_total_diff_nodes[Type=="CE" & is.microexon=="TRUE", ])
t=nrow(Tasic_total_diff_nodes[Type=="CE" & is.microexon=="TRUE" &  is.diff=="TRUE", ])
dhyper(t, a, n - a, b)
```


```
[1] 1.423587e-20
```


```
sum(dhyper(t:b, a, n - a, b))
```


```
[1] 1.534839e-20
```


```
nrow(Tasic_total_diff_nodes[ is.microexon=="TRUE" &  is.diff=="TRUE", ])
```


```
[1] 29
```


```
Tasic_total_diff_nodes[is.diff==TRUE & is.microexon==TRUE, ]


Tasic_unpooled_diff_exons <- fread("/Users/gp7/Google_Drive/Results/ME/Paper/Final_Report/Reps/Rep1/Single_Cell/Unpooled/GABA-ergic_Neuron_vs_Glutamatergic_Neuron.diff.microexons")

Tasic_unpooled_pooled <- merge(Tasic_unpooled_diff_exons[ , c("exon_ID", "Probability", "DeltaPsi")], 
Tasic_total_diff_nodes[ is.microexon==TRUE , .(exon_ID=microexon_ID, mean_Probability=Probability.mean, mean_DeltaPsi=DeltaPsi.mean, sig_pool=is.diff)  ],
by="exon_ID")

Tasic_unpooled_pooled[ , sig_unpool:=FALSE ]
Tasic_unpooled_pooled[ abs(DeltaPsi) >= 0.1 &  Probability>=0.9, sig_unpool:=TRUE ]


 Tasic_unpooled_pooled[  ,.N, by=c( "sig_unpool", "sig_pool") ]
```


```
 Tasic_unpooled_pooled[  ,.N, by=c( "sig_unpool", "sig_pool") ]
```


```
   sig_unpool sig_pool    N
1:      FALSE    FALSE 2291
2:      FALSE     TRUE   19
3:       TRUE     TRUE   10
4:       TRUE    FALSE    4
```

## Tasic


```
 Tasic_unpooled_pooled[  ,.N, by=c( "sig_unpool", "sig_pool") ]
```


```
   sig_unpool sig_pool    N
1:      FALSE    FALSE 2291
2:      FALSE     TRUE   19
3:       TRUE     TRUE   10
4:       TRUE    FALSE    4
```


```
library(ggplot2)
library(data.table)

ggplot(Tasic_unpooled_diff_exons) +
  geom_histogram(aes(Entropy), binwidth=0.1)
```


```
Tasic_unpooled_pooled <- merge(Tasic_unpooled_diff_exons[ , c("exon_ID", "Probability", "DeltaPsi")], 
Tasic_pooled_diff_exons[ , .( mean_Probability=mean(Probability), mean_DeltaPsi=mean(DeltaPsi)) , by="exon_ID"  ],
by="exon_ID")

Tasic_unpooled_pooled[ , sig_unpool:=FALSE ]
Tasic_unpooled_pooled[ abs(DeltaPsi) >= 0.1 &  Probability>=0.9, sig_unpool:=TRUE ]

Tasic_unpooled_pooled[ , sig_pool:=FALSE ]
Tasic_unpooled_pooled[ abs(mean_DeltaPsi) >= 0.1 &  mean_Probability>=0.9, sig_pool:=TRUE ]

Tasic_pooled_diff_exons[, sd_probability:=sd(Probability) , by="Coord"]

ggplot(Tasic_pooled_diff_exons) +
  geom_histogram(aes(x=sd_probability))
```


```
nrow(Tasic_unpooled_pooled[sig_unpool=="TRUE"])
nrow(Tasic_unpooled_pooled[sig_pool=="TRUE"])
```


```
ggplot() +
  
  geom_point( data=Tasic_unpooled_pooled, aes(mean_DeltaPsi - DeltaPsi , mean_Probability -Probability )) +
  geom_point( data=Tasic_unpooled_pooled[sig_unpool==FALSE & sig_pool==TRUE]  , aes(mean_DeltaPsi - DeltaPsi , mean_Probability -Probability ), color="red" ) +
  geom_point( data=Tasic_unpooled_pooled[sig_unpool==TRUE & sig_pool==TRUE]  , aes(mean_DeltaPsi - DeltaPsi , mean_Probability -Probability ), color="green" ) +
  geom_point( data=Tasic_unpooled_pooled[sig_unpool==TRUE & sig_pool==FALSE]  , aes(mean_DeltaPsi - DeltaPsi , mean_Probability -Probability ), color="blue" ) +
  theme_bw()
```


```
Sig_GABAergic_Neuron_vs_Glutamatergic_Neuron_old <- Sig_GABAergic_Neuron_vs_Glutamatergic_Neuron

Sig_GABAergic_Neuron_vs_Glutamatergic_Neuron_old[ order(-abs(mean_DeltaPsi)), c("exon_ID", "mgi_symbol", "wikigene_description", "mean_DeltaPsi", "mean_Probability")]
```


```
Sig_GABAergic_Neuron_vs_Glutamatergic_Neuron <- Tasic_unpooled_pooled[abs(mean_DeltaPsi - DeltaPsi) <=0.25 &  sig_pool==TRUE, ]

Sig_GABAergic_Neuron_vs_Glutamatergic_Neuron <- merge( Sig_GABAergic_Neuron_vs_Glutamatergic_Neuron, unique(gene_table.TOTAL[, c("ME", "mgi_symbol")]), by.x="exon_ID", by.y="ME")

#Sig_GABAergic_Neuron_vs_Glutamatergic_Neuron <- merge( Sig_GABAergic_Neuron_vs_Glutamatergic_Neuron , unique(gene_info_total[ , c("mgi_symbol", "wikigene_description")]), by= "mgi_symbol")


Sig_GABAergic_Neuron_vs_Glutamatergic_Neuron[ order(-abs(mean_DeltaPsi))]

length(unique(Sig_GABAergic_Neuron_vs_Glutamatergic_Neuron[, mgi_symbol]))
```


```
#ref Slc4a10 https://www.frontiersin.org/articles/10.3389/fncel.2015.00223/full
#ref Ank3 https://www.sciencedirect.com/science/article/pii/S0896627314009088
#ref KCNMA1 https://www.sciencedirect.com/science/article/pii/S0896627313001852
#ref Kif3a https://www.sciencedirect.com/science/article/pii/S089662730300062X
# ref DLGAP https://molecularbrain.biomedcentral.com/articles/10.1186/s13041-017-0324-9


Pre_synaptic <- c( "Ptprd" , "Ppfia2", "Dlgap1", "Gabrg2", "Kcnma1", "Kif3a", "Cadps") 
Post_synaptic <- c( "Nrxn3" , "Nrxn1")
PrePost_synaptic <- c("Ank3","Slc4a10")
```


```
Sig_GABAergic_Neuron_vs_Glutamatergic_Neuron[  !exon_ID %in% microexons_GENCODE &  !exon_ID %in% microexons_Vastdb , c("exon_ID", "mgi_symbol", "wikigene_description", "mean_DeltaPsi", "mean_Probability")][order(-abs(mean_DeltaPsi))]

microexons_GENCODE
microexons_Vastdb
```


```
Brain_string_interactions_out[mgi_symbol=="Akap13",]
```


```
write.table(Sig_GABAergic_Neuron_vs_Glutamatergic_Neuron, file = "~/Desktop/Sig_GABAergic_Neuron_vs_Glutamatergic_Neuron.txt", append = FALSE, quote = F, sep = "\t",
            eol = "\n", na = "NA", dec = ".", row.names = F,
            col.names = TRUE, qmethod = c("escape", "double"),
            fileEncoding = "")
```


```
ggplot()+
  geom_point(data=Tasic_unpooled_pooled, aes(mean_DeltaPsi, mean_Probability)) +
  geom_point(data=Sig_GABAergic_Neuron_vs_Glutamatergic_Neuron, aes(mean_DeltaPsi, mean_Probability), color="red")
```


```
library("ggrepel")


Sig_GABAergic_Neuron_vs_Glutamatergic_Neuron[mgi_symbol %in% Pre_synaptic, Location:="Presynaptic"]
Sig_GABAergic_Neuron_vs_Glutamatergic_Neuron[mgi_symbol %in% Post_synaptic, Location:="Postsynaptic"]
Sig_GABAergic_Neuron_vs_Glutamatergic_Neuron[mgi_symbol %in% PrePost_synaptic, Location:="Both"]


#Sig_GABAergic_Neuron_vs_Glutamatergic_Neuron <- Sig_GABAergic_Neuron_vs_Glutamatergic_Neuron[!exon_ID %in% ME_blacklist$ME]

ggplot()+
  geom_point(data=Tasic_unpooled_pooled, aes(mean_DeltaPsi, mean_Probability), color="grey") +
  geom_point(data=Sig_GABAergic_Neuron_vs_Glutamatergic_Neuron, aes(mean_DeltaPsi, mean_Probability), color="black") +
  geom_text_repel(data = Sig_GABAergic_Neuron_vs_Glutamatergic_Neuron[mgi_symbol %in% Pre_synaptic],
                  colour="forestgreen", aes(x=mean_DeltaPsi, y=mean_Probability), 
                  nudge_y      = 3,
                  direction    = "x",
                  angle        = 90,
                  vjust        = 1,
                  segment.size = 0.2,
                  label=Sig_GABAergic_Neuron_vs_Glutamatergic_Neuron[mgi_symbol %in% Pre_synaptic , mgi_symbol] ) +
  
  geom_text_repel(data = Sig_GABAergic_Neuron_vs_Glutamatergic_Neuron[mgi_symbol %in% Post_synaptic],
                  colour="firebrick4", aes(x=mean_DeltaPsi, y=mean_Probability), 
                  nudge_y      = 3,
                  direction    = "x",
                  angle        = 90,
                  vjust        = 1,
                  segment.size = 0.2,
                  label=Sig_GABAergic_Neuron_vs_Glutamatergic_Neuron[mgi_symbol %in% Post_synaptic , mgi_symbol] ) +
  
  
  geom_text_repel(data = Sig_GABAergic_Neuron_vs_Glutamatergic_Neuron[mgi_symbol %in% PrePost_synaptic],
                  colour="darkgoldenrod", aes(x=mean_DeltaPsi, y=mean_Probability), 
                  nudge_y      = 3,
                  direction    = "x",
                  angle        = 90,
                  vjust        = 1,
                  segment.size = 0.2,
                  label=Sig_GABAergic_Neuron_vs_Glutamatergic_Neuron[mgi_symbol %in% PrePost_synaptic , mgi_symbol] ) +
    
  
  ylim(0.5, 1.15) +
  theme_bw() +
  xlab("") +
  xlab("Mean delta PSI") +
  ylab("Mean probability")
```


```
ggplot()+
  geom_point(data=Tasic_unpooled_pooled, aes(mean_DeltaPsi, mean_Probability), color="grey") +
  geom_point(data=Sig_GABAergic_Neuron_vs_Glutamatergic_Neuron, aes(mean_DeltaPsi, mean_Probability), color="black") +
  geom_text_repel(data = Sig_GABAergic_Neuron_vs_Glutamatergic_Neuron[mgi_symbol %in% Pre_synaptic],
                  colour="forestgreen", aes(x=mean_DeltaPsi, y=mean_Probability), 
                  nudge_y      = 3,
                  direction    = "x",
                  angle        = 90,
                  vjust        = 1,
                  segment.size = 0.2,
                  label=Sig_GABAergic_Neuron_vs_Glutamatergic_Neuron[mgi_symbol %in% Pre_synaptic , mgi_symbol] ) +
  
  geom_text_repel(data = Sig_GABAergic_Neuron_vs_Glutamatergic_Neuron[mgi_symbol %in% Post_synaptic],
                  colour="firebrick4", aes(x=mean_DeltaPsi, y=mean_Probability), 
                  nudge_y      = 3,
                  direction    = "x",
                  angle        = 90,
                  vjust        = 1,
                  segment.size = 0.2,
                  label=Sig_GABAergic_Neuron_vs_Glutamatergic_Neuron[mgi_symbol %in% Post_synaptic , mgi_symbol] ) +
  
  
  geom_text_repel(data = Sig_GABAergic_Neuron_vs_Glutamatergic_Neuron[mgi_symbol %in% PrePost_synaptic],
                  colour="darkgoldenrod", aes(x=mean_DeltaPsi, y=mean_Probability), 
                  nudge_y      = 3,
                  direction    = "x",
                  angle        = 90,
                  vjust        = 1,
                  segment.size = 0.2,
                  label=Sig_GABAergic_Neuron_vs_Glutamatergic_Neuron[mgi_symbol %in% PrePost_synaptic , mgi_symbol] ) +
    
  
  ylim(0.5, 1.15) +
  theme_bw() +
  xlab("") +
  xlab("Mean delta PSI") +
  ylab("Mean probability")
```


```
Error in geom_text_repel(data = Sig_GABAergic_Neuron_vs_Glutamatergic_Neuron[mgi_symbol %in%  : 
  could not find function "geom_text_repel"
```


```
Sig_GABAergic_Neuron_vs_Glutamatergic_Neuron[mgi_symbol %in% Pre_synaptic, Location:="Presynaptic"]
```


```
Error in .checkTypos(e, names_x) : 
  Object 'mgi_symbol' not found amongst exon_ID, Probability, DeltaPsi, mean_Probability, mean_DeltaPsi and 2 more
```


```
primary_type_levels <- c(unique(ME_cov_filtered_tasic_primary_type[broad_type=="Endothelial Cell", primary_type]),
unique(ME_cov_filtered_tasic_primary_type[broad_type=="Oligodendrocyte", primary_type]),
unique(ME_cov_filtered_tasic_primary_type[broad_type=="Microglia", primary_type]),
unique(ME_cov_filtered_tasic_primary_type[broad_type=="Oligodendrocyte Precursor Cell", primary_type]),
sort(unique(ME_cov_filtered_tasic_primary_type[broad_type=="Astrocyte", primary_type])),
sort(unique(ME_cov_filtered_tasic_primary_type[broad_type=="Glutamatergic Neuron", primary_type])),
unique(ME_cov_filtered_tasic_primary_type[broad_type=="GABA-ergic Neuron", primary_type]) )

ME_cov_filtered_tasic_primary_type$primary_type <- factor(ME_cov_filtered_tasic_primary_type$primary_type , levels = primary_type_levels)
```


```
primary_type_levels <- c(unique(ME_cov_filtered_tasic_primary_type[broad_type=="Endothelial Cell", primary_type]),
unique(ME_cov_filtered_tasic_primary_type[broad_type=="Oligodendrocyte", primary_type]),
unique(ME_cov_filtered_tasic_primary_type[broad_type=="Microglia", primary_type]),
unique(ME_cov_filtered_tasic_primary_type[broad_type=="Oligodendrocyte Precursor Cell", primary_type]),
sort(unique(ME_cov_filtered_tasic_primary_type[broad_type=="Astrocyte", primary_type])),
sort(unique(ME_cov_filtered_tasic_primary_type[broad_type=="Glutamatergic Neuron", primary_type])),
unique(ME_cov_filtered_tasic_primary_type[broad_type=="GABA-ergic Neuron", primary_type]) )
```


```
Error in unique(ME_cov_filtered_tasic_primary_type[broad_type == "Endothelial Cell",  : 
  object 'ME_cov_filtered_tasic_primary_type' not found
```


```
target_genes <- c("Gabrg2", "Nrxn1", "Nrxn3", "Ptprd")
ME_cov_filtered_tasic_primary_type[(mgi_symbol %in% target_genes) & (ME %in% Sig_GABAergic_Neuron_vs_Glutamatergic_Neuron$exon_ID)]
```


```
                            ME primary_type           broad_type sum_ME_coverage sum_SJ_coverage total_cov_alternatives_3 total_cov_alternatives_5       PSI     lower
  1: chr11_-_41913969_41913993   Vip Mybpc1    GABA-ergic Neuron            5881             887                        0                        0 0.8689421 0.8606926
  2: chr11_-_41913969_41913993    Vip Parm1    GABA-ergic Neuron            5465            3692                        0                        0 0.5968112 0.5867254
  3: chr11_-_41913969_41913993     L4 Ctxn3 Glutamatergic Neuron           12660            7816                        0                        0 0.6182848 0.6116091
  4: chr11_-_41913969_41913993     Vip Chat    GABA-ergic Neuron            5390            3757                        0                        0 0.5892642 0.5791469
  5: chr11_-_41913969_41913993   L2/3 Ptgs2 Glutamatergic Neuron            8799            3910                        0                        0 0.6923440 0.6842630
 ---                                                                                                                                                                  
339:  chr4_-_76139293_76139302  Pvalb Obox3    GABA-ergic Neuron               0               5                        0                        0 0.0000000 0.0000000
340:  chr4_-_76139293_76139302     L5b Tph2 Glutamatergic Neuron               3               0                        0                        0       NaN 0.4385030
341:  chr4_-_76139293_76139302      L5 Ucma Glutamatergic Neuron              10               0                        0                        0 1.0000000 0.7224672
342:  chr4_-_76139293_76139302    L5 Chrna6 Glutamatergic Neuron               3               0                        0                        0       NaN 0.4385030
343:  chr4_-_76139293_76139302    L5b Cdh13 Glutamatergic Neuron              19               0                        0                        0 1.0000000 0.8318208
         upper ensembl_transcript_id    ensembl_gene_id mgi_symbol
  1: 0.8767730    ENSMUST00000070725 ENSMUSG00000020436     Gabrg2
  2: 0.6068157    ENSMUST00000070725 ENSMUSG00000020436     Gabrg2
  3: 0.6249162    ENSMUST00000070725 ENSMUSG00000020436     Gabrg2
  4: 0.5993067    ENSMUST00000070725 ENSMUSG00000020436     Gabrg2
  5: 0.7003088    ENSMUST00000070725 ENSMUSG00000020436     Gabrg2
 ---                                                              
339: 0.4344825    ENSMUST00000174180 ENSMUSG00000028399      Ptprd
340: 1.0000000    ENSMUST00000174180 ENSMUSG00000028399      Ptprd
341: 1.0000000    ENSMUST00000174180 ENSMUSG00000028399      Ptprd
342: 1.0000000    ENSMUST00000174180 ENSMUSG00000028399      Ptprd
343: 1.0000000    ENSMUST00000174180 ENSMUSG00000028399      Ptprd
```


```
ME_cov_filtered_tasic_primary_type[is.na(PSI), `:=`(lower=NA, upper=NA) ]
ggplot(ME_cov_filtered_tasic_primary_type[ mgi_symbol %in% target_genes & ME %in% Sig_GABAergic_Neuron_vs_Glutamatergic_Neuron$exon_ID, ]  ) +
  geom_pointrange(aes(x=primary_type, y=PSI, colour= broad_type, group=broad_type, ymin=lower, ymax=upper))+
  facet_grid( paste0( mgi_symbol, ME) ~ . ) +
  theme_bw() +
  theme(axis.text.x = element_text(angle = 90, hjust = 0, vjust = 0.5)) +
  theme(axis.text.x = element_text(angle = 90), legend.position = "top", legend.direction = "horizontal")
```


###### 

Numbers


```
###Abstract


Bulk_RNA_SEQ.Table <-  table(grepl("ENC",  unique(ME_cov_filtered[, FILE_NAME])))

Total_Bulk_RNA_seq <- sum(Bulk_RNA_SEQ.Table)
Total_scRNA_seq <-nrow(tasic_metadata)

Total_microexon.number <- length(ME_final[ , unique(ME)])
#HNMF_microexons.number <- length(Delta_HNMF_whippet_ME_age_diff_total_PPI[ , unique(exon_ID)])


diff_spliced.brain_dev <- length(unique(Delta_HNM_whippet_ME_age_diff_total$exon_ID , Delta_F_whippet_ME_age_diff_total$exon_ID))


diff_spliced.brain_dev.novel <-  length(unique(Delta_HNM_whippet_ME_age_diff_total[ ! exon_ID %in% c(microexons_Vastdb, microexons_GENCODE) ,  exon_ID  ], Delta_F_whippet_ME_age_diff_total[ ! exon_ID %in% c(microexons_Vastdb, microexons_GENCODE) ,  exon_ID  ]))


### Introduction

Total_microexon.number.not_gencode <- length(ME_final[ !ME %in% microexons_GENCODE , unique(ME)])
Total_microexon.number.not_gencode.percentage <- (Total_microexon.number.not_gencode / Total_microexon.number)*100


diff_spliced.brain_dev.not_vastdb <-  length(unique(Delta_HNM_whippet_ME_age_diff_total[ ! exon_ID %in% c(microexons_Vastdb) ,  exon_ID  ], Delta_F_whippet_ME_age_diff_total[ ! exon_ID %in% c(microexons_Vastdb) ,  exon_ID  ]))


diff_spliced.brain_dev.not_vastdb.percentage <- (diff_spliced.brain_dev.not_vastdb/diff_spliced.brain_dev)*100


conserved_zebra <-  length(unique(Mouse_Zebra.IDs[ , ME.zebra]))


conserved_zebra.not_ensembl <- length(unique(Mouse_Zebra.IDs[ensemble_zebra==FALSE, ME.zebra]))


#Results 

me_after_10_samples_filter <- rownames(Tissue_PSI_matrix_dcast[apply(Tissue_PSI_matrix_dcast, 1, function(x) length(which(is.na(x)))) < 289*0.9, ])

ME_final.out[ME %in% me_after_10_samples_filter, .N,  by="type"]


sym_percentage.neuronal_neuromuscular_muscular <- ME_cluster_names[ ME_cluster.type %in% c("Neuronal", "Neuro-Muscular", "Muscular") , 100- (sum(asym)/sum(total))*100]


Delta_HNM_whippet_ME_age_diff_total
Delta_HNMF_whippet_ME_age_diff_total

HNMF_microexons.not_vastdb.number <- nrow(Delta_HNMF_whippet_ME_age_diff_total_PPI[!exon_ID %in% microexons_Vastdb, ])
HNMF_microexons.not_vastdb.percentage <- round((HNMF_microexons.not_vastdb.number/HNMF_microexons.number)*100, 2)


Novel_gencode.number <- nrow(ME_final[ , .N, by=ME ][ !ME %in% microexons_GENCODE, ])
Novel_gencode.percentage <- (Novel_gencode.number / Total_microexon.number) *100


Novel_vastdb.number <- nrow(ME_final[ , .N, by=ME ][ !ME %in% microexons_Vastdb, ])
Novel_vastdb.percentage <- (Novel_vastdb.number / Total_microexon.number) *100


MHN_diff.whippet_ME <- length( unique(Delta_HNM_whippet[ abs(DeltaPsi)>=0.1 &  Probability >= 0.9 & exon_ID %in%  Delta_HNM_ME[ abs(DeltaPsi)>=0.1 &  Probability >= 0.9 ,  exon_ID ]   ,  exon_ID ]))
F_diff.whippet_ME <- length( unique(Delta_F_whippet[ abs(DeltaPsi)>=0.1 &  Probability >= 0.9 & exon_ID %in%  Delta_F_ME[ abs(DeltaPsi)>=0.1 &  Probability >= 0.9 ,  exon_ID ]   ,  exon_ID ]))


diff_heart <- nrow(Delta_Heart_whippet_ME[diff_high==TRUE, ])
diff_SKM <- nrow(Delta_SKM_whippet_ME[diff_high==TRUE, ])
diff_AG <- nrow(Delta_AG_whippet_ME[diff_high==TRUE, ])


Delta_HNMF_whippet_ME_age_diff_total[ , type:="Annotated"  ]
Delta_HNMF_whippet_ME_age_diff_total[!exon_ID %in% microexons_Vastdb, type:="Missing in VastDB"   ]
Delta_HNMF_whippet_ME_age_diff_total[!exon_ID %in% microexons_GENCODE, type:="Missing in GENCODE"   ]
Delta_HNMF_whippet_ME_age_diff_total[!exon_ID %in% microexons_GENCODE & !exon_ID %in% microexons_Vastdb , type:="Novel"   ]

table(Delta_HNMF_whippet_ME_age_diff_total$type)
ME_sizes <- ME_final.rep1$len_micro_exon_seq_found
names(ME_sizes) <- ME_final.rep1$ME


Delta_HNMF_whippet_ME_age_diff_total[ type=="Novel", .N , by=c("type", "ME_len") ]


Sig_GABAergic_Neuron_vs_Glutamatergic_Neuron[ sig_unpool==TRUE ,  ]
```


```
ggplot(Delta_HNMF_whippet_ME_age_diff_total) +
  geom_histogram(aes(x=ME_len, fill=type), binwidth=1) +
  facet_grid(  type ~ .) +
  scale_fill_discrete(name="Microexon type") +
  xlab("Microexon length") +
  theme(legend.position="top") +
  theme(strip.background = element_blank(), strip.text = element_blank())
```


```
me_after_10_samples_filter <- rownames(Tissue_PSI_matrix_dcast[apply(Tissue_PSI_matrix_dcast, 1, function(x) length(which(is.na(x)))) < 289*0.9, ])
me_after_10_samples_filter <- rownames(Tissue_PSI_matrix_dcast[apply(Tissue_PSI_matrix_dcast, 1, function(x) length(which(is.na(x)))) < 289*0.9, ])
ME_final.out[ME %in% me_after_10_samples_filter, .N,  by="type"]
```


```
                 type    N
1:              Novel  866
2:          Annotated 1556
3: Missing in GENCODE  177
```


```
#rationale: https://www.gungorbudak.com/blog/2016/05/25/computing-significance-of-overlap/

AG_brain.intersect.number <- length(intersect(Delta_AG_whippet_ME[diff_high==TRUE]$exon_ID, Delta_HNMF_whippet_ME_age_diff_total$exon_ID))


AG.number <- length(unique(Delta_AG_whippet_ME[diff_high==TRUE, exon_ID]))
brain.number <- length(unique(Delta_HNMF_whippet_ME_age_diff_total$exon_ID))

Microexons.PPCA.number

phyper( AG_brain.intersect.number , AG.number,  Microexons.PPCA.number-AG.number, brain.number, lower.tail = FALSE  )
```

##### Supplemental


```
ME_clusters_table <- data.frame(ME_clusters)

ME_clusters_table$ME <- row.names(ME_clusters_table)
ME_clusters_table <- data.table(ME_clusters_table)


ME_clusters_PSI <-  data.table(merge(Tissue_PSI_matrix_melt_ppca, ME_clusters_table , by=c("ME")))


#ME_clusters_PSI <- ME_clusters_PSI[, c("ME", "FILE_NAME", "ME_clusters", "PSI")]


ME_Tissues_clusters_PSI<- merge(ME_clusters_PSI, PCA, by.x="FILE_NAME", by.y="File.accession")

ME_Tissues_clusters_PSI[, Tissue_clusters:=cluster]

PCA_loadings_stats$ME_cluster <- factor(PCA_loadings_stats$ME_cluster)
ME_Tissues_clusters_PSI$ME_clusters <- factor(ME_Tissues_clusters_PSI$ME_clusters)

ME_Tissues_clusters_PSI <- merge(ME_Tissues_clusters_PSI, PCA_loadings_stats, by.x="ME_clusters", by.y="ME_cluster" )

ME_Tissues_clusters_PSI$Tissue_clusters <- factor(ME_Tissues_clusters_PSI$Tissue_clusters, levels=PCA[, mean(PC1), by="cluster" ][order(V1)]$cluster) 


sub_ME_Tissues_clusters_PSI <- ME_Tissues_clusters_PSI[abs(PC1_mean)>0, ]
sub_ME_Tissues_clusters_PSI <- data.table(sub_ME_Tissues_clusters_PSI)
sub_ME_Tissues_clusters_PSI$ME_clusters <- factor(sub_ME_Tissues_clusters_PSI$ME_clusters , levels=ME_cluster_loading_order )


sub_ME_Tissues_clusters_PSI_aggregated <-  sub_ME_Tissues_clusters_PSI[, list(PSI=mean(PSI, na.rm = TRUE)), by=list(ME, ME_clusters,  Tissue_clusters) ]
sub_ME_Tissues_clusters_PSI_aggregated_mean <-  sub_ME_Tissues_clusters_PSI_aggregated[, list(PSI= mean(PSI, na.rm=TRUE) ), by=list(ME_clusters,  Tissue_clusters) ]


ggplot( ) +
        geom_line(data= sub_ME_Tissues_clusters_PSI_aggregated, aes(factor(Tissue_clusters), PSI, group=ME), colour="grey") +
        geom_line(data = sub_ME_Tissues_clusters_PSI_aggregated_mean, aes(factor(Tissue_clusters), PSI, group=ME_clusters),  colour="red" ) +
        facet_grid( ME_clusters ~ .) +
        xlab("Sample clusters (sorted by PC1)") +
        theme(panel.background = element_rect(fill = 'white', colour = 'black'))


col1 <- c("E1", "E2", "E3", "E4", "E5", "E6", "I1", "I2", "I3", "M1", "M2", "M3")
col2 <- c("N1", "N2", "N3", "N4", "NM1", "NM2", "NM3", "NN1", "O1", "O2", "WN1", "WN2")


sub_ME_Tissues_clusters_PSI_aggregated$ME_cluster <- as.numeric(as.character(sub_ME_Tissues_clusters_PSI_aggregated$ME_clusters)) 
sub_ME_Tissues_clusters_PSI_aggregated_mean$ME_cluster <- as.numeric(as.character(sub_ME_Tissues_clusters_PSI_aggregated_mean$ME_clusters)) 

plot_grid(
ggplot( ) +
        geom_line(data= merge(sub_ME_Tissues_clusters_PSI_aggregated, ME_cluster_names, by.x="ME_cluster",  by.y="ME_cluster")[ME_cluster.name %in% col1] , aes(factor(Tissue_clusters), PSI, group=ME), colour="grey") +
        geom_line(data = merge(sub_ME_Tissues_clusters_PSI_aggregated_mean, ME_cluster_names, by.x="ME_cluster",  by.y="ME_cluster")[ME_cluster.name %in% col1], aes(factor(Tissue_clusters), PSI, group=ME_clusters),  colour="red" ) +
        facet_grid( ME_cluster.name ~ .) +
        xlab("Sample clusters (sorted by PC1)") +
        theme(panel.background = element_rect(fill = 'white', colour = 'black')),

ggplot( ) +
        geom_line(data= merge(sub_ME_Tissues_clusters_PSI_aggregated, ME_cluster_names, by.x="ME_cluster",  by.y="ME_cluster")[ME_cluster.name %in% col2] , aes(factor(Tissue_clusters), PSI, group=ME), colour="grey") +
        geom_line(data = merge(sub_ME_Tissues_clusters_PSI_aggregated_mean, ME_cluster_names, by.x="ME_cluster",  by.y="ME_cluster")[ME_cluster.name %in% col2], aes(factor(Tissue_clusters), PSI, group=ME_clusters),  colour="red" ) +
        facet_grid( ME_cluster.name ~ .) +
        xlab("Sample clusters (sorted by PC1)") +
        theme(panel.background = element_rect(fill = 'white', colour = 'black'))

)
```


```
PCA_loadings$ME_clusters <- as.numeric(as.character(PCA_loadings$ME_cluster)) 


Sup_loading.A <- ggplot(merge(PCA_loadings, ME_cluster_names, by.x="ME_clusters",  by.y="ME_cluster")) +
  geom_boxplot(aes(ME_cluster.name , -PC1)) +
  ylab("Loading factors in PC1") +
  xlab("Microexon cluster") +
  theme(axis.text.x = element_text(angle = 90))


Sup_loading.B <-  ggplot(merge(PCA_loadings, ME_cluster_names, by.x="ME_clusters",  by.y="ME_cluster")) +
  geom_boxplot(aes(ME_cluster.name , PC2)) +
  ylab("Loading factors in PC2") +
  xlab("Microexon cluster") +
  theme(axis.text.x = element_text(angle = 90))


Sup_loading.C <-  ggplot(merge(PCA_loadings, ME_cluster_names, by.x="ME_clusters",  by.y="ME_cluster")) +
  geom_boxplot(aes(ME_cluster.name , PC3)) +
  ylab("Loading factors in PC3") +
  xlab("Microexon cluster") +
  theme(axis.text.x = element_text(angle = 90))


plot_grid(Sup_loading.A, Sup_loading.B, Sup_loading.C, labels = "AUTO", ncol=1)


PPI_centrality_GO[GO=="Axon_guidance", .N, by="mapped_gene" ]
```


Tables


```
TS2 <- unique(merge(Sig_GABAergic_Neuron_vs_Glutamatergic_Neuron, gene_table.TOTAL, by.x="exon_ID", by.y="ME"  ))


colnames(TS2) <- c("Coordinates", "Probability", "DeltaPsi", "Mean Probability", "Mean DeltaPsi", "Significance unpool", "Significance Pool", "Ensembl transcript ID",    "Ensembl gene ID", "Gene name")

fwrite(TS2, file = "../Final_Figures/Supplementary/Table_S2.csv", append = FALSE, quote = "auto", sep = ",",  row.names = FALSE, col.names = TRUE)
```


```
#R -e  'rmarkdown::render("src/final_filters2.Rmd",params = list(ME_table="/lustre/scratch117/cellgen/team218/gp7/Micro-exons/Runs/Paper/MicroExonator/Round2/TOTAL.ME_centric.txt", ME_coverage="/lustre/scratch117/cellgen/team218/gp7/Micro-exons/Runs/Paper/MicroExonator/Round2/TOTAL.filter1.ME_SJ_coverage", ME_matches_file="/lustre/scratch117/cellgen/team218/gp7/Micro-exons/Runs/Paper/MicroExonator/Round2/TOTAL.ME_centric.ME_matches.txt", out_filtered_ME="/lustre/scratch117/cellgen/team218/gp7/Micro-exons/Runs/Paper/MicroExonator/Report/test/out_filtered_ME.txt", out_low_scored_ME="/lustre/scratch117/cellgen/team218/gp7/Micro-exons/Runs/Paper/MicroExonator/Report/test/out_low_scored_ME.txt", out_shorter_than_3_ME="/lustre/scratch117/cellgen/team218/gp7/Micro-exons/Runs/Paper/MicroExonator/Report/test/out_shorter_than_3_ME.txt", min_number_files_detected=3, out_filtered_ME_cov="/lustre/scratch117/cellgen/team218/gp7/Micro-exons/Runs/Paper/MicroExonator/Report/test/out_filtered_ME.cov.txt" ), output_format="pdf_document", output_file="/lustre/scratch117/cellgen /team218/gp7/Micro-exons/Runs/Paper/MicroExonator/Report/report.pdf")'
```

### BetaDist


```
tasic.betadist <- fread("../../Single_cell/BetaDist/Sig_nodes/GABA-ergic_Neuron_vs_Glutamatergic_Neuron.txt")
ME_final[, .( stri_split_fixed(ME, "_")) ]
Sig_GABAergic_Neuron_vs_Glutamatergic_Neuron

library(splitstackshape)
ME_final.chords <- cSplit(ME_final, 'ME', '_')[ is.na(ME_5) , .( paste0(ME_1, ":", as.numeric(as.character(ME_3))+1,  "-", ME_4) ) ]$V1


ME_final.chords <- cSplit(Sig_GABAergic_Neuron_vs_Glutamatergic_Neuron, 'exon_ID', '_')[  , .( paste0(exon_ID_1, ":", as.numeric(as.character(exon_ID_3))+1,  "-", exon_ID_4) ) ]$V1

tasic.betadist[ Coord %in%  ME_final.chords,]

cSplit(ME_final, 'ME', '_')[ is.na(ME_5) , .( paste0(ME_1, ":", ME_3,  "-", ME_4) ) ]


ME_final.chords[, .(as.numeric(as.character(ME_3)), ) ]

ME_final[ .(ME)]
```

# Supplementary tables.


```
MHN.diff <- unique(Delta_HNM_merge[abs(DeltaPsi.x)>=0.1 & Probability.x>0.9 & abs(DeltaPsi.y)>=0.1 & Probability.y>0.9, exon_ID])
F.diff <- unique(Delta_F_merge[abs(DeltaPsi.x)>=0.1 & Probability.x>0.9 & abs(DeltaPsi.y)>=0.1 & Probability.y>0.9, exon_ID])
Delta_HNM_whippet_ME_age_diff_total[ , .(ME=exon_ID, HNM.diff_age=diff_age, HNM.change_dir=change_dir)] 
Delta_F_whippet_ME_age_diff_total[ , .(ME=exon_ID, F.diff_age=diff_age, F.change_dir=change_dir)] 
Delta_SKM_whippet_ME[ , .(ME=exon_ID, SKM.diff_age=diff_high)] 
Delta_Heart_whippet_ME[ , .(ME=exon_ID, Heart.diff_age=diff_high)] 
Delta_AG_whippet_ME[ , .(ME=exon_ID, AG.diff_age=diff_high)] 

TS1 <- ME_final.out[, .(ME,
             transcript,
             sum_total_coverage,
             total_SJs,
             total_coverages,
             len_micro_exon_seq_found,
             micro_exon_seq_found,
             total_number_of_micro_exons_matches,
             U2_scores,
             mean_conservations_vertebrates,
             P_MEs,
             total_ME,
             ME_P_value,
             ME_type,
             ensembl_transcript_id,
             type,
             Alt5_3,
             ME_cluster.type,
             ME_cluster.name,
             ME_cluster.number,
             PC1,
             PC2,
             PC3, 
             In.10_percent_of_bulk)]


TS1[ , MHN.diff:=(ME %in% MHN.diff) ]
TS1[ , F.diff:=(ME %in% F.diff) ]

TS1 <- merge(TS1, Delta_HNM_whippet_ME_age_diff_total[ , .(ME=exon_ID, HNM.diff_age=diff_age, HNM.change_dir=change_dir)], by="ME", all.x=TRUE) 
TS1 <- merge(TS1, Delta_F_whippet_ME_age_diff_total[ , .(ME=exon_ID, F.diff_age=diff_age, F.change_dir=change_dir)] , by="ME", all.x=TRUE) 
TS1 <- merge(TS1, Delta_SKM_whippet_ME[ , .(ME=exon_ID, SKM.diff=diff_high)] , by="ME", all.x=TRUE)
TS1 <- merge(TS1, Delta_Heart_whippet_ME[ , .(ME=exon_ID, Heart.diff=diff_high)] , by="ME", all.x=TRUE )
TS1 <- merge(TS1, Delta_AG_whippet_ME[ , .(ME=exon_ID, AG.diff=diff_high)] , by="ME", all.x=TRUE)

TS1[is.na(SKM.diff), SKM.diff:=FALSE ]
TS1[is.na(Heart.diff), Heart.diff:=FALSE ]
TS1[is.na(AG.diff), AG.diff:=FALSE ]

TS4 <- total_zebra

TS5 <- Mouse_Zebra.IDs.table.summer[!is.na(mouse.coord), .(mouse.coord,
                                                    mouse.len,
                                                    mouse.cluster_name,
                                                    mouse.cluster_type,
                                                    mouse.anno,
                                                    mouse.ensembl,
                                                    zebra.coord,
                                                    zebra.len,
                                                    zebra.ensembl)]


non_published <- Delta_HNMF_whippet_ME_age_diff_total[, .( ENSEMBL_gene_id=ensembl_gene_id,
                                          mgi_symbol=mgi_symbol.x,
                                          exon_ID,
                                          microexon.length=ME_len,
                                          change.direction=change_dir, 
                                          MHN.diff_start=diff_age.x,
                                          F.MHN.diff_start=diff_age.y,
                                          annotation.status=type
                                          )
                                     ]


TS6 <- Tasic_total_diff_nodes


ENCODE_metadata


Tissue_pool.table <- Tissue_clusters[ , .(File.accession, 
                                         Tissue=name,
                                         Age.DPC=age,
                                         Tissue.cluster=cluster)]


Tissue_pool.table[ Tissue %in% c("hindbrain", "neural tube", "midbrain") & Age.DPC %in% c(10.5, 11.5, 12.5, 13.5, 14.5, 15.5, 16.5),
                   Bulk.sample.group:=paste("MHN", Age.DPC, sep="_") ]
Tissue_pool.table[ Tissue %in% c("forebrain") & Age.DPC %in% c(10.5, 11.5, 12.5, 13.5, 14.5, 15.5, 16.5, 21) , 
                   Bulk.sample.group:=paste("F", Age.DPC, sep="_") ]

Tissue_pool.table[Tissue.cluster %in% c(1, 8, 6), Bulk.sample.group:="Control"]

Tissue_pool.table[Tissue=="heart" , Bulk.sample.group:="Heart" ]
Tissue_pool.table[Tissue=="skeletal muscle tissue" , Bulk.sample.group:="SKM" ]
Tissue_pool.table[Tissue=="adrenal gland" , Bulk.sample.group:="AD" ]

TS2 <- Tissue_pool.table
View(Tissue_pool.table)
```


```
TS1 
TS2
TS3
TS4
TS5
TS6[!is.na(microexon_ID) & is.diff==TRUE, ]


fwrite(TS1, file = "../Final_Figures/Supplementary/TS1.tsv", append = FALSE, quote = "auto", sep = "\t",  row.names = FALSE, col.names = TRUE)
fwrite(TS2, file = "../Final_Figures/Supplementary/TS2.tsv", append = FALSE, quote = "auto", sep = "\t",  row.names = FALSE, col.names = TRUE)
fwrite(TS4, file = "../Final_Figures/Supplementary/TS4.tsv", append = FALSE, quote = "auto", sep = "\t",  row.names = FALSE, col.names = TRUE)
fwrite(TS5, file = "../Final_Figures/Supplementary/TS5.tsv", append = FALSE, quote = "auto", sep = "\t",  row.names = FALSE, col.names = TRUE)
fwrite(TS6, file = "../Final_Figures/Supplementary/TS6.tsv", append = FALSE, quote = "auto", sep = "\t",  row.names = FALSE, col.names = TRUE)
```

LS0tCnRpdGxlOiAiUiBOb3RlYm9vayIKb3V0cHV0OiBodG1sX25vdGVib29rCi0tLQoKCgpgYGB7cn0KbGlicmFyeShyZXNoYXBlMikKbGlicmFyeShnZ3Bsb3QyKQpsaWJyYXJ5KGdwbG90cykKbGlicmFyeShwaGVhdG1hcCkKbGlicmFyeShwbHlyKQpzZXQuc2VlZCgxMjMpCmxpYnJhcnkoZGF0YS50YWJsZSkKbGlicmFyeShtYWdyaXR0cikKbGlicmFyeShkcGx5cikKbGlicmFyeShyZWFkcikKbGlicmFyeShjb3dwbG90KQpsaWJyYXJ5KGlncmFwaCkKdGhlbWVfc2V0KHRoZW1lX2NsYXNzaWMoKSApCmBgYAoKCgoKYGBge3J9CgoKZ2V0X2NvbG9ycyA8LSBmdW5jdGlvbihncm91cHMsIGdyb3VwLmNvbCA9IHBhbGV0dGUoKSl7CiAgZ3JvdXBzIDwtIGFzLmZhY3Rvcihncm91cHMpCiAgbmdycHMgPC0gbGVuZ3RoKGxldmVscyhncm91cHMpKQogIGlmKG5ncnBzID4gbGVuZ3RoKGdyb3VwLmNvbCkpIAogICAgZ3JvdXAuY29sIDwtIHJlcChncm91cC5jb2wsIG5ncnBzKQogIGNvbG9yIDwtIGdyb3VwLmNvbFthcy5udW1lcmljKGdyb3VwcyldCiAgbmFtZXMoY29sb3IpIDwtIGFzLnZlY3Rvcihncm91cHMpCiAgcmV0dXJuKGNvbG9yKQp9CgoKZ2dfY29sb3JfaHVlIDwtIGZ1bmN0aW9uKG4pIHsKICBodWVzID0gc2VxKDE1LCAzNzUsIGxlbmd0aCA9IG4gKyAxKQogIGhjbChoID0gaHVlcywgbCA9IDY1LCBjID0gMTAwKVsxOm5dCn0KYGBgCgoKCgpJbXBvcnRpbmcgbWV0YWRhdGEKCgpgYGB7cn0KCkVOQ09ERV9tZXRhZGF0YSA8LSByZWFkLmRlbGltKCJ+L0dvb2dsZV9Ecml2ZS9SZXN1bHRzL01FL21tMTAvbWV0YWRhdGEudHN2IiwgIGhlYWRlcj1UUlVFLCBzZXA9Ilx0IikKCkVOQ09ERV9tZXRhZGF0YSA8LSBkYXRhLnRhYmxlKEVOQ09ERV9tZXRhZGF0YSkKCgoKV2V5bl9lbF9hbF9tZXRhZGF0YSA8LSBkYXRhLnRhYmxlKHJlYWRfZGVsaW0oIn4vR29vZ2xlX0RyaXZlL1Jlc3VsdHMvTUUvUGFwZXIvV2V5bl9lbF9hbC5tZXRhZGF0YS50eHQiLCAgIlx0IiwgZXNjYXBlX2RvdWJsZSA9IEZBTFNFLCB0cmltX3dzID0gVFJVRSkpCgpXZXluX2VsX2FsX21ldGFkYXRhWywgYDo9YChGaWxlLmFjY2Vzc2lvbj1SdW4sCiAgICAgICAgICAgICAgICAgICAgQmlvc2FtcGxlLkFnZT1hZ2UsCiAgICAgICAgICAgICAgICAgICAgRXhwZXJpbWVudC5kYXRlLnJlbGVhc2VkPUxvYWREYXRlLAogICAgICAgICAgICAgICAgICAgIEJpb3NhbXBsZS50ZXJtLm5hbWU9dGlzc3VlLAogICAgICAgICAgICAgICAgICAgIFBhaXJlZC5lbmQ9TkEsCiAgICAgICAgICAgICAgICAgICAgUGFpcmVkLndpdGg9IiIsCiAgICAgICAgICAgICAgICAgICAgQXVkaXQuV0FSTklORz0iIikgXQoKbWV0YWRhdGEgPC0gcmJpbmQoIEVOQ09ERV9tZXRhZGF0YVssIGMoIkZpbGUuYWNjZXNzaW9uIiwgIkJpb3NhbXBsZS5BZ2UiLCAiRXhwZXJpbWVudC5kYXRlLnJlbGVhc2VkIiwgIkJpb3NhbXBsZS50ZXJtLm5hbWUiLCAiUGFpcmVkLmVuZCIsICJQYWlyZWQud2l0aCIsICJBdWRpdC5XQVJOSU5HIildLAogICAgICAgICAgICAgICAgICBXZXluX2VsX2FsX21ldGFkYXRhWywgYygiRmlsZS5hY2Nlc3Npb24iLCAiQmlvc2FtcGxlLkFnZSIsICJFeHBlcmltZW50LmRhdGUucmVsZWFzZWQiLCAiQmlvc2FtcGxlLnRlcm0ubmFtZSIsICJQYWlyZWQuZW5kIiwgIlBhaXJlZC53aXRoIiwgIkF1ZGl0LldBUk5JTkciKV0gKQoKCm1ldGFkYXRhIDwtIG1ldGFkYXRhW0F1ZGl0LldBUk5JTkchPSJsb3cgcmVwbGljYXRlIGNvbmNvcmRhbmNlLCBtaXNzaW5nIGZsb3djZWxsX2RldGFpbHMiLF0KCgoKYGBgCgoKCgoKCgpgYGB7cn0KCk1FX2ZpbmFsLnJlcDEgPC0gZnJlYWQoIn4vR29vZ2xlX0RyaXZlL1Jlc3VsdHMvTUUvUGFwZXIvRmluYWxfUmVwb3J0L1JlcHMvUmVwMS9vdXQuaGlnaF9xdWFsaXR5LnR4dCIpCk1FX2ZpbmFsLnJlcDIgPC0gZnJlYWQoIn4vR29vZ2xlX0RyaXZlL1Jlc3VsdHMvTUUvUGFwZXIvRmluYWxfUmVwb3J0L1JlcHMvUmVwMi9vdXQuaGlnaF9xdWFsaXR5LnR4dCIpCgpNRV9maW5hbCA8LSBNRV9maW5hbC5yZXAxWyBNRSAlaW4lIE1FX2ZpbmFsLnJlcDIkTUUsIF0KCgpNRV9jb3YgPC0gZnJlYWQoIn4vR29vZ2xlX0RyaXZlL1Jlc3VsdHMvTUUvUGFwZXIvRmluYWxfUmVwb3J0L1JlcHMvUmVwMS9vdXRfZmlsdGVyZWRfTUUuUFNJLnR4dCIpCk1FX2NvdlssIGA6PWAoRklMRV9OQU1FPUZpbGUsIE1FPU1FX2Nvb3JkcyldCgpudW1iZXJfb2ZfbWljcm9leG9ucyA8LSBsZW5ndGgoTUVfZmluYWxbICwgdW5pcXVlKE1FKV0pCgoKCk1FX2Nvdl9maWx0ZXJlZCA8LSBNRV9jb3ZbTUUgJWluJSBNRV9maW5hbFssIE1FXSAgLCBdCgoKYGBgCgoKYGBge3J9CmxpYnJhcnkoc3RyaW5naSkKCgoKCiNtYXQgPC0gc3RyaV9zcGxpdF9maXhlZChNRV9jb3ZfZmlsdGVyZWQkTUVfY292ZXJhZ2VzLCAnLCcsIHNpbXBsaWZ5PVQpCiNtYXQgPC0gYGRpbTwtYChhcy5udW1lcmljKG1hdCksIGRpbShtYXQpKSAgIyBjb252ZXJ0IHRvIG51bWVyaWMgYW5kIHNhdmUgZGltcwojcm93c3VtKG1hdCwgbmEucm09VCkKCiNzdW0obWF0LCBuYS5ybT1UKQoKCiNzYXBwbHkoc3Ryc3BsaXQoYXMuY2hhcmFjdGVyKE1FX2Nvdl9maWx0ZXJlZCRNRV9jb3ZlcmFnZXMpLCAiLCIsIGZpeGVkPVQpLCBmdW5jdGlvbih4KSBzdW0oYXMubnVtZXJpYyh4KSkpCmBgYAoKCmBgYHtyfQojTUVfY292X2ZpbHRlcmVkWywgc3Ryc3BsaXQoIE1FX2NvdmVyYWdlcywgIiwiKSBdCgoKI2xpYnJhcnkoZHBseXIpCiNsaWJyYXJ5KHRpZHlyKQojZGYgJT4lCiMgICBzZXBhcmF0ZV9yb3dzKHksIHosIGNvbnZlcnQgPSBUUlVFKSAlPiUKIyAgIGdyb3VwX2J5KHgpICU+JSAKIyAgIHN1bW1hcmlzZV9hbGwoc3VtKQpgYGAKCgoKT2J0YWluaW4gUFNJIG1hdHJpeAoKYGBge3J9CgoKClRpc3N1ZXMgPC0gYXMuY2hhcmFjdGVyKG1ldGFkYXRhJEJpb3NhbXBsZS50ZXJtLm5hbWUpCm5hbWVzKFRpc3N1ZXMpIDwtIGFzLmNoYXJhY3RlcihtZXRhZGF0YSRGaWxlLmFjY2Vzc2lvbikKCkFnZXMgPC0gYXMuY2hhcmFjdGVyKG1ldGFkYXRhJEJpb3NhbXBsZS5BZ2UpCm5hbWVzKEFnZXMpIDwtIGFzLmNoYXJhY3RlcihtZXRhZGF0YSRGaWxlLmFjY2Vzc2lvbikKCgoKTUVfY292X2ZpbHRlcmVkICA8LSB1bmlxdWUoTUVfY292X2ZpbHRlcmVkW0ZJTEVfTkFNRSAlaW4lIG1ldGFkYXRhWyAsIEZpbGUuYWNjZXNzaW9uXSxdKQojTUVfY292X2ZpbHRlcmVkX0VOQ09ERSA8LSB1bmlxdWUoTUVfY292X2ZpbHRlcmVkW0ZJTEVfTkFNRSAlaW4lIG1ldGFkYXRhWyAsIEZpbGUuYWNjZXNzaW9uXSxdKQoKCgojY29sbmFtZXMoVGlzc3VlX1BTSV9tYXRyaXhfZGNhc3QpIDwtIGFzLmNoYXJhY3RlciggcGFzdGUoc2VwID0gIiAiLCBUaXNzdWVzW2NvbG5hbWVzKFRpc3N1ZV9QU0lfbWF0cml4X2RjYXN0KV0sIEFnZXNbY29sbmFtZXMoVGlzc3VlX1BTSV9tYXRyaXhfZGNhc3QpXSkpICNUbyByZXBsYXNlIHRoZSBmaWxlIG5hbWUgYnkgdGhlIGJpb2xvZ2ljYWwgc2FtcGxlIG5hbWUKCiNUaXNzdWVfUFNJX21hdHJpeF9kY2FzdCA8LSBUaXNzdWVfUFNJX21hdHJpeF9kY2FzdFssIG9yZGVyKHBhc3RlKHNlcCA9ICIgIiwgVGlzc3Vlc1tjb2xuYW1lcyhUaXNzdWVfUFNJX21hdHJpeF9kY2FzdCldLCBBZ2VzW2NvbG5hbWVzKFRpc3N1ZV9QU0lfbWF0cml4X2RjYXN0KV0pKV0KCgpgYGAKCgoKCgpQcm9iYWJpbGlzdGljIFBDQQoKCgoKYGBge3J9CgpsaWJyYXJ5KHBjYU1ldGhvZHMpCgpUaXNzdWVfUFNJX21hdHJpeF9tZWx0IDwtIE1FX2Nvdl9maWx0ZXJlZFssIGMoIk1FIiwgIkZJTEVfTkFNRSIsICJQU0kiKSBdClRpc3N1ZV9QU0lfbWF0cml4X2RjYXN0IDwtIHJlc2hhcGUyOjpkY2FzdChUaXNzdWVfUFNJX21hdHJpeF9tZWx0LCBNRSB+IEZJTEVfTkFNRSkKcm93Lm5hbWVzKFRpc3N1ZV9QU0lfbWF0cml4X2RjYXN0KSA8LSBUaXNzdWVfUFNJX21hdHJpeF9kY2FzdCRNRQpUaXNzdWVfUFNJX21hdHJpeF9kY2FzdCA8LSBkYXRhLm1hdHJpeChUaXNzdWVfUFNJX21hdHJpeF9kY2FzdClbLC0xXQoKClRpc3N1ZV9QU0lfbWF0cml4X2RjYXN0W1Rpc3N1ZV9QU0lfbWF0cml4X2RjYXN0PT0iTmFOIl0gPC0gTkEKCmRpbShUaXNzdWVfUFNJX21hdHJpeF9kY2FzdCkKCmRpbShUaXNzdWVfUFNJX21hdHJpeF9kY2FzdFthcHBseShUaXNzdWVfUFNJX21hdHJpeF9kY2FzdCwgMSwgZnVuY3Rpb24oeCkgbGVuZ3RoKHdoaWNoKGlzLm5hKHgpKSkpIDwgMjcxKjAuOSwgXSkKCmRpbShUaXNzdWVfUFNJX21hdHJpeF9kY2FzdFthcHBseShUaXNzdWVfUFNJX21hdHJpeF9kY2FzdCwgMSwgZnVuY3Rpb24oeCkgbGVuZ3RoKHdoaWNoKGlzLm5hKHgpKSkpIDwgMjg5KjAuOSwgXSkKCnJlc3VsdCA8LSBwY2EodChUaXNzdWVfUFNJX21hdHJpeF9kY2FzdFthcHBseShUaXNzdWVfUFNJX21hdHJpeF9kY2FzdCwgMSwgZnVuY3Rpb24oeCkgbGVuZ3RoKHdoaWNoKGlzLm5hKHgpKSkpIDwgMjg5KjAuOSwgXSksIG1ldGhvZD0icHBjYSIsIG5QY3M9Mywgc2VlZD0xMjMpCiMjIEdldCB0aGUgZXN0aW1hdGVkIGNvbXBsZXRlIG9ic2VydmF0aW9ucwpjT2JzIDwtIGNvbXBsZXRlT2JzKHJlc3VsdCkKIyMgUGxvdCB0aGUgc2NvcmVzCnBsb3RQY3MocmVzdWx0LCB0eXBlID0gInNjb3JlcyIpCgoKCgpzdW1tYXJ5KHJlc3VsdCkKCgoKCmBgYAoKCgoKYGBge3J9CgpUaXNzdWVfUFNJX21hdHJpeF9kY2FzdF9wcGNhIDwtIHQoY09icykKClRpc3N1ZV9QU0lfbWF0cml4X2RjYXN0X3BwY2FbVGlzc3VlX1BTSV9tYXRyaXhfZGNhc3RfcHBjYT4xXSA8LSAxClRpc3N1ZV9QU0lfbWF0cml4X2RjYXN0X3BwY2FbVGlzc3VlX1BTSV9tYXRyaXhfZGNhc3RfcHBjYTwwXSA8LSAwCgoKIyNjb2xuYW1lcyhUaXNzdWVfUFNJX21hdHJpeF9kY2FzdF9wcGNhKSA8LSBhcy5jaGFyYWN0ZXIoIHBhc3RlKHNlcCA9ICIgIiwgVGlzc3Vlc1tjb2xuYW1lcyhUaXNzdWVfUFNJX21hdHJpeF9kY2FzdF9wcGNhKV0sIEFnZXNbY29sbmFtZXMoVGlzc3VlX1BTSV9tYXRyaXhfZGNhc3RfcHBjYSldKSkgI1RvIHJlcGxhc2UgdGhlIGZpbGUgbmFtZSBieSB0aGUgYmlvbG9naWNhbCBzYW1wbGUgbmFtZQoKCgojdGlzc3VlX2hlYXRtYXAgPC0gcGhlYXRtYXA6OnBoZWF0bWFwKFRpc3N1ZV9QU0lfbWF0cml4X2RjYXN0X3BwY2EsICAsIGZvbnRzaXplID0gNCwgIGN1dHJlZV9yb3dzID0gMjQsIGN1dHJlZV9jb2xzID0gMTYsIGNsdXN0ZXJpbmdfbWV0aG9kID0gIndhcmQuRDIiKQoKCgpgYGAKCgoKCmBgYHtyfQoKI1Rpc3N1ZV9QU0lfbWF0cml4X2RjYXN0X3BwY2EgPC0gdChjT2JzKQoKI1Rpc3N1ZV9QU0lfbWF0cml4X2RjYXN0X3BwY2FbVGlzc3VlX1BTSV9tYXRyaXhfZGNhc3RfcHBjYT4xXSA8LSAxCiNUaXNzdWVfUFNJX21hdHJpeF9kY2FzdF9wcGNhW1Rpc3N1ZV9QU0lfbWF0cml4X2RjYXN0X3BwY2E8MF0gPC0gMAoKCiMjY29sbmFtZXMoVGlzc3VlX1BTSV9tYXRyaXhfZGNhc3RfcHBjYSkgPC0gYXMuY2hhcmFjdGVyKCBwYXN0ZShzZXAgPSAiICIsIFRpc3N1ZXNbY29sbmFtZXMoVGlzc3VlX1BTSV9tYXRyaXhfZGNhc3RfcHBjYSldLCBBZ2VzW2NvbG5hbWVzKFRpc3N1ZV9QU0lfbWF0cml4X2RjYXN0X3BwY2EpXSkpICNUbyByZXBsYXNlIHRoZSBmaWxlIG5hbWUgYnkgdGhlIGJpb2xvZ2ljYWwgc2FtcGxlIG5hbWUKCgoKI3Rpc3N1ZV9oZWF0bWFwIDwtIHBoZWF0bWFwOjpwaGVhdG1hcChUaXNzdWVfUFNJX21hdHJpeF9kY2FzdF9wcGNhLCAgLCBmb250c2l6ZSA9IDQsICBjdXRyZWVfcm93cyA9IDI0LCBjdXRyZWVfY29scyA9IDE3LCBjbHVzdGVyaW5nX21ldGhvZCA9ICJ3YXJkLkQyIikKCgoKYGBgCgoKCmBgYHtyfQoKaGNfY29scyA8LSBoY2x1c3QoZGlzdCh0KFRpc3N1ZV9QU0lfbWF0cml4X2RjYXN0X3BwY2EpKSwgbWV0aG9kID0gIndhcmQuRDIiKQpoY19yb3dzIDwtIGhjbHVzdChkaXN0KFRpc3N1ZV9QU0lfbWF0cml4X2RjYXN0X3BwY2EpLCBtZXRob2QgPSAid2FyZC5EMiIpCgpgYGAKCgoKCgpgYGB7cn0KVGlzc3Vlc19uYW1lIDwtIGFzLmNoYXJhY3RlcihtZXRhZGF0YSRCaW9zYW1wbGUudGVybS5uYW1lKQpuYW1lcyhUaXNzdWVzX25hbWUpIDwtIGFzLmNoYXJhY3RlcihtZXRhZGF0YSRGaWxlLmFjY2Vzc2lvbikKClRpc3N1ZXNfYWdlIDwtIGFzLmNoYXJhY3RlcihtZXRhZGF0YSRCaW9zYW1wbGUuQWdlKQpuYW1lcyhUaXNzdWVzX2FnZSkgPC0gYXMuY2hhcmFjdGVyKG1ldGFkYXRhJEZpbGUuYWNjZXNzaW9uKQoKVGlzc3VlX2RhdGUgPC0gYXMuY2hhcmFjdGVyKG1ldGFkYXRhJEV4cGVyaW1lbnQuZGF0ZS5yZWxlYXNlZCkKbmFtZXMoVGlzc3VlX2RhdGUpIDwtIGFzLmNoYXJhY3RlcihtZXRhZGF0YSRGaWxlLmFjY2Vzc2lvbikKCmBgYAoKCgoKYGBge3J9CiNUaXNzdWVfY2x1c3RlcnMgPC0gY3V0cmVlKGhjX2NvbHMsIGsgPSAxNikKI1Rpc3N1ZV9jbHVzdGVycyA8LSBjYmluZChUaXNzdWVfY2x1c3RlcnMsIFRpc3N1ZXNfbmFtZVtuYW1lcyhUaXNzdWVfY2x1c3RlcnMpXSwgVGlzc3Vlc19hZ2VbbmFtZXMoVGlzc3VlX2NsdXN0ZXJzKV0sIFRpc3N1ZV9kYXRlW25hbWVzKFRpc3N1ZV9jbHVzdGVycyldKSAKCiNjb2xuYW1lcyhUaXNzdWVfY2x1c3RlcnMpIDwtIGMoImNsdXN0ZXIiLCAibmFtZSIsICJhZ2UiLCAiZGF0ZSIpCgojVGlzc3VlX2NsdXN0ZXJzIDwtIGRhdGEuZnJhbWUoVGlzc3VlX2NsdXN0ZXJzKQoKIyNUaXNzdWVfY2x1c3RlcnNfc3VtW3doaWNoKGdyZXBsKCJoaW5kYnJhaW58bWlkYnJhaW58Zm9yZWJyYWlufG5ldXJhbFwgdHViZSIsIFRpc3N1ZV9jbHVzdGVyc19zdW0kbmFtZSkpLCBdCgojVGlzc3VlX2NsdXN0ZXJzJG5hbWUgPC0gZmFjdG9yKFRpc3N1ZV9jbHVzdGVycyRuYW1lLCBsZXZlbD1jKCAic2tlbGV0YWwgbXVzY2xlIHRpc3N1ZSIsICJoZWFydCIsICJ0aHltdXMiLCAic3BsZWVuIiwgImxpdmVyIiwgICJhZHJlbmFsIGdsYW5kIiwgImludGVzdGluZSIsICJzdG9tYWNoIiwgImx1bmciLCAgImtpZG5leSIsICJibGFkZGVyIiwgImxpbWIiLCAiZW1icnlvbmljIGZhY2lhbCBwcm9taW5lbmNlIiwgImZvcmVicmFpbiIsICJoaW5kYnJhaW4iLCAibWlkYnJhaW4iLCAibmV1cmFsIHR1YmUiLCAid2hvbGUgY29ydGV4IikpCgoKI1Rpc3N1ZV9jbHVzdGVycyRhZ2UgPC0gbWFwdmFsdWVzKFRpc3N1ZV9jbHVzdGVycyRhZ2UsIAojICAgICAgICAgIGZyb20gPSBjKCIxMC41IGRheSIsICIxMS41IGRheSIsICIxMi41IGRheSIsICIxMy41IGRheSIsICIxNC41IGRheSIsICIxNS41IGRheSIsICIxNi41IGRheSIsICIwIGRheSIsICI4IHdlZWsiLCAiUDBhIiwgIlAwYiIsICJQMTEwYSIsICJQMTEwYiIsICJQMTVhIiwgIlAxNWIiLCAiUDMwYSIsICJQMzBiIiwgIlA0YSIsICMiUDRhIiwgIlA3YSIsICJQN2IiLCAiMjFNYSIsICIyMU1iIiwgIkUxNC41IiwgIkUxNi41IiwgIlA0YiIpLAojICAgICAgICAgdG8gPSBjKDEwLjUsIDExLjUsIDEyLjUsIDEzLjUsIDE0LjUsIDE1LjUsIDE2LjUsIDIxLCA1OSwgMjEsIDIxLCAxMzEsIDEzMSwgMzYsIDM2LCA0MSw0MSwgMjUsIDI1LCAyNywyNywgNjUxLCA2NTEsIDE0LjUsIDE2LjUsIDI1ICkgKQoKCgoKI1Rpc3N1ZV9jbHVzdGVycyRhZ2UgPC0gZmFjdG9yKFRpc3N1ZV9jbHVzdGVycyRhZ2UsIGxldmVscyA9IGFzLmNoYXJhY3Rlcihzb3J0KGFzLm51bWVyaWMobGV2ZWxzKFRpc3N1ZV9jbHVzdGVycyRhZ2UpKSkpKQoKCgoKYGBgCgoKCmBgYHtyfQpUaXNzdWVfY2x1c3RlcnMgPC0gY3V0cmVlKGhjX2NvbHMsIGsgPSAxNikKVGlzc3VlX2NsdXN0ZXJzIDwtIGNiaW5kKFRpc3N1ZV9jbHVzdGVycywgVGlzc3Vlc19uYW1lW25hbWVzKFRpc3N1ZV9jbHVzdGVycyldLCBUaXNzdWVzX2FnZVtuYW1lcyhUaXNzdWVfY2x1c3RlcnMpXSwgVGlzc3VlX2RhdGVbbmFtZXMoVGlzc3VlX2NsdXN0ZXJzKV0pIAoKY29sbmFtZXMoVGlzc3VlX2NsdXN0ZXJzKSA8LSBjKCJjbHVzdGVyIiwgIm5hbWUiLCAiYWdlIiwgImRhdGUiKQoKVGlzc3VlX2NsdXN0ZXJzIDwtIGRhdGEuZnJhbWUoVGlzc3VlX2NsdXN0ZXJzKQoKI1Rpc3N1ZV9jbHVzdGVyc19zdW1bd2hpY2goZ3JlcGwoImhpbmRicmFpbnxtaWRicmFpbnxmb3JlYnJhaW58bmV1cmFsXCB0dWJlIiwgVGlzc3VlX2NsdXN0ZXJzX3N1bSRuYW1lKSksIF0KClRpc3N1ZV9jbHVzdGVycyRuYW1lIDwtIGZhY3RvcihUaXNzdWVfY2x1c3RlcnMkbmFtZSwgbGV2ZWw9YyggInNrZWxldGFsIG11c2NsZSB0aXNzdWUiLCAiaGVhcnQiLCAidGh5bXVzIiwgInNwbGVlbiIsICJsaXZlciIsICAiYWRyZW5hbCBnbGFuZCIsICJpbnRlc3RpbmUiLCAic3RvbWFjaCIsICJsdW5nIiwgICJraWRuZXkiLCAiYmxhZGRlciIsICJsaW1iIiwgImVtYnJ5b25pYyBmYWNpYWwgcHJvbWluZW5jZSIsICJmb3JlYnJhaW4iLCAiaGluZGJyYWluIiwgIm1pZGJyYWluIiwgIm5ldXJhbCB0dWJlIiwgIndob2xlIGNvcnRleCIpKQoKI1Rpc3N1ZV9jbHVzdGVycyRhZ2UgPC0gZmFjdG9yKFRpc3N1ZV9jbHVzdGVycyRhZ2UsIGxldmVscz1jKCIxMC41IGRheSIsICIxMS41IGRheSIsICIxMi41IGRheSIsICIxMy41IGRheSIsICIxNC41IGRheSIsICIxNS41IGRheSIsICIxNi41IGRheSIsICIwIGRheSIsICI4IHdlZWsiKSkKClRpc3N1ZV9jbHVzdGVycyRGaWxlLmFjY2Vzc2lvbiA8LSByb3cubmFtZXMoVGlzc3VlX2NsdXN0ZXJzKQoKClRpc3N1ZV9jbHVzdGVycyA8LSBkYXRhLnRhYmxlKFRpc3N1ZV9jbHVzdGVycykKCgpUaXNzdWVfY2x1c3RlcnMkYWdlIDwtIG1hcHZhbHVlcyhUaXNzdWVfY2x1c3RlcnMkYWdlLCAKICAgICAgICAgIGZyb20gPSBjKCIxMC41IGRheSIsICIxMS41IGRheSIsICIxMi41IGRheSIsICIxMy41IGRheSIsICIxNC41IGRheSIsICIxNS41IGRheSIsICIxNi41IGRheSIsICIwIGRheSIsICI4IHdlZWsiLCAiUDBhIiwgIlAwYiIsICJQMTEwYSIsICJQMTEwYiIsICJQMTVhIiwgIlAxNWIiLCAiUDMwYSIsICJQMzBiIiwgIlA0YSIsICJQNGEiLCAiUDdhIiwgIlA3YiIsICIyMU1hIiwgIjIxTWIiLCAiRTE0LjUiLCAiRTE2LjUiLCAiUDRiIiksCiAgICAgICAgICB0byA9IGMoMTAuNSwgMTEuNSwgMTIuNSwgMTMuNSwgMTQuNSwgMTUuNSwgMTYuNSwgMjEsIDU5LCAyMSwgMjEsIDEzMSwgMTMxLCAzNiwgMzYsIDQxLDQxLCAyNSwgMjUsIDI3LDI3LCA2NTEsIDY1MSwgMTQuNSwgMTYuNSwgMjUgKSApCgoKCmBgYAoKCgoKCgoKCmBgYHtyfQoKTUVfY2x1c3RlcnMgPC0gY3V0cmVlKGhjX3Jvd3MsIGsgPSAyNCkKClBDQV9sb2FkaW5ncyA8LSBkYXRhLmZyYW1lKGxvYWRpbmdzKHJlc3VsdCkpClBDQV9sb2FkaW5ncyRNRSA8LSByb3cubmFtZXMoUENBX2xvYWRpbmdzKQoKClBDQV9sb2FkaW5ncyA8LSBkYXRhLnRhYmxlKFBDQV9sb2FkaW5ncykKUENBX2xvYWRpbmdzWywgYDo9YChQQzE9LVBDMSwgUEMyPS1QQzIpXQoKClBDQV9sb2FkaW5ncyRNRV9jbHVzdGVyIDwtIE1FX2NsdXN0ZXJzW1BDQV9sb2FkaW5ncyRNRV0KCgpQQ0FfbG9hZGluZ3Nfc3RhdHMgPC0gUENBX2xvYWRpbmdzWyAsIC4oUEMxX21lYW49bWVhbihQQzEpKSAsIGJ5PSJNRV9jbHVzdGVyIl0KTUVfY2x1c3Rlcl9sb2FkaW5nX29yZGVyIDwtIFBDQV9sb2FkaW5nc19zdGF0c1tvcmRlcihQQzFfbWVhbildJE1FX2NsdXN0ZXIKClBDQV9sb2FkaW5ncyRNRV9jbHVzdGVyIDwtIGZhY3RvcihQQ0FfbG9hZGluZ3MkTUVfY2x1c3RlciAsIGxldmVscz1NRV9jbHVzdGVyX2xvYWRpbmdfb3JkZXIpCgoKZ2dwbG90KFBDQV9sb2FkaW5ncykgKwogIGdlb21fYm94cGxvdChhZXMoYXMuZmFjdG9yKE1FX2NsdXN0ZXIpICwgUEMxKSkKCgpgYGAKCgoKYGBge3J9CmdncGxvdChQQ0FfbG9hZGluZ3MpICsKICBnZW9tX2JveHBsb3QoYWVzKGFzLmZhY3RvcihNRV9jbHVzdGVyKSAsIFBDMikpCmBgYAoKCgoKYGBge3J9CmdncGxvdChQQ0FfbG9hZGluZ3MpICsKICBnZW9tX2JveHBsb3QoYWVzKGFzLmZhY3RvcihNRV9jbHVzdGVyKSAsIFBDMykpCmBgYAoKCgoKCmBgYHtyfQoKUENBX2xvYWRpbmdzX3N0YXRzIDwtIFBDQV9sb2FkaW5nc1sgLCAuKFBDMV9tZWFuPW1lYW4oUEMxKSkgLCBieT0iTUVfY2x1c3RlciJdCgpQQ0FfbG9hZGluZ3Nfc3RhdHNfUEMyIDwtIFBDQV9sb2FkaW5nc1sgLCAuKFBDMl9tZWFuPW1lYW4oUEMyKSkgLCBieT0iTUVfY2x1c3RlciJdCgoKCk1FX2NsdXN0ZXJfbG9hZGluZ19vcmRlciA8LSBQQ0FfbG9hZGluZ3Nfc3RhdHNbb3JkZXIoUEMxX21lYW4pXSRNRV9jbHVzdGVyCmBgYAoKTmV1cm9uYWwgTWljcm9leG9ucwoKYGBge3J9ClBDQV9sb2FkaW5nc19zdGF0c1thYnMoUEMxX21lYW4pPjAuMDMsIF1bb3JkZXIoUEMxX21lYW4pXQpgYGAKCgoKYGBge3J9ClBDQV9sb2FkaW5nc19zdGF0c19QQzJbYWJzKFBDMl9tZWFuKT4wLjAzLCBdW29yZGVyKC1QQzJfbWVhbildCmBgYAoKCgoKYGBge3J9ClRpc3N1ZV9QU0lfbWF0cml4X21lbHRfcHBjYSA8LSBtZWx0KFRpc3N1ZV9QU0lfbWF0cml4X2RjYXN0X3BwY2EpCgpjb2xuYW1lcyhUaXNzdWVfUFNJX21hdHJpeF9tZWx0X3BwY2EpIDwtIGMoIk1FIiwgIkZJTEVfTkFNRSIsICJQU0kiKQoKTUVfY2x1c3RlcnNfdGFibGUgPC0gZGF0YS5mcmFtZShNRV9jbHVzdGVycykKCk1FX2NsdXN0ZXJzX3RhYmxlJE1FIDwtIHJvdy5uYW1lcyhNRV9jbHVzdGVyc190YWJsZSkKTUVfY2x1c3RlcnNfdGFibGUgPC0gZGF0YS50YWJsZShNRV9jbHVzdGVyc190YWJsZSkKCgpNRV9jbHVzdGVyc19QU0kgPC0gIGRhdGEudGFibGUobWVyZ2UoVGlzc3VlX1BTSV9tYXRyaXhfbWVsdF9wcGNhLCBNRV9jbHVzdGVyc190YWJsZSAsIGJ5PWMoIk1FIikpKQoKCgpmd3JpdGUoIE1FX2NsdXN0ZXJzX1BTSSwgIi4uLy4uL1BhcGVyL0phY29iL01FX2NsdXN0ZXJzX1BTSS50dnMiLCBxdW90ZSA9IEZBTFNFLCBjb2wubmFtZXMgPSBUUlVFLCBzZXA9Ilx0IikKCmZ3cml0ZSggTUVfY2x1c3Rlcl9uYW1lcywgIi4uLy4uL1BhcGVyL0phY29iL01FX2NsdXN0ZXJfbmFtZXMudHZzIiwgcXVvdGUgPSBGQUxTRSwgY29sLm5hbWVzID0gVFJVRSwgc2VwPSJcdCIpCgpgYGAKCgpgYGB7cn0KTUVfY2x1c3RlcnNfUFNJJE1FX2NsdXN0ZXJzIDwtIG1hcHZhbHVlcyhNRV9jbHVzdGVyc19QU0kkTUVfY2x1c3RlcnMsIAogICAgICAgICAgZnJvbSA9MToxOCwKICAgICAgICAgIHRvID0gYygiSTEiLCAiRTEiLCAiRTMiLCAiSTIiLCAiTjEiLCAiTTEiLCAiTjIiLCAiTk0zIiwgIk5NMiIsICJONSIsICJOTTEiLCAiTjMiLCAiTjQiLCAiTk4yIiwgIkUyIiwgIkk0IiwgIkkzIiwgIk5OMSIpKQpgYGAKCgoKYGBge3J9ClRpc3N1ZV9QU0lfbWF0cml4X21lbHRfcHBjYSA8LSBkYXRhLnRhYmxlKFRpc3N1ZV9QU0lfbWF0cml4X21lbHRfcHBjYSkKClRpc3N1ZV9QU0lfbWF0cml4X21lbHRfcHBjYQoKYGBgCgoKCgpgYGB7cn0KClBDQV9sb2FkaW5nc19zdGF0cyA8LSBQQ0FfbG9hZGluZ3NbICwgLihQQzFfbWVhbj1tZWFuKFBDMSksIFBDMl9tZWFuPW1lYW4oUEMyKSApICwgYnk9Ik1FX2NsdXN0ZXIiXQoKI1BDQV9sb2FkaW5nc19zdGF0c1ssIFBDMV9tZWFuOj0tUEMxX21lYW5dCgpQQ0FfbG9hZGluZ3Nfc3RhdHNbb3JkZXIoLVBDMV9tZWFuKV0KUENBX2xvYWRpbmdzX3N0YXRzW29yZGVyKFBDMl9tZWFuKV0KCiNQQ0FfbG9hZGluZ3Nfc3RhdHNfUEMyWywgUEMyX21lYW46PS1QQzJfbWVhbl0KYGBgCmBgYHtyfQpQQ0FfbG9hZGluZ3Nfc3RhdHNbb3JkZXIoUEMyX21lYW4pXQoKYGBgCgoKYGBge3J9CgoKCmxvYWRpbmdfdGhyZXNob2xkIDwtIDAuMDEwCmxvYWRpbmdfdGhyZXNob2xkX1BDMiA8LSAwLjAxMAoKCk5ldXJvbmFsIDwtIFBDQV9sb2FkaW5nc19zdGF0c1sgcm91bmQoUEMxX21lYW4sIDMpID4gIGxvYWRpbmdfdGhyZXNob2xkLCBdW29yZGVyKC1QQzFfbWVhbildJE1FX2NsdXN0ZXIKTXVzY3VsYXIgPC0gUENBX2xvYWRpbmdzX3N0YXRzX1BDMltyb3VuZChQQzJfbWVhbiwgMykgPCAtbG9hZGluZ190aHJlc2hvbGRfUEMyLCBdW29yZGVyKFBDMl9tZWFuKV0kTUVfY2x1c3RlcgoKTm9uTmV1cm9uYWw8LSBQQ0FfbG9hZGluZ3Nfc3RhdHNbIHJvdW5kKFBDMV9tZWFuLCAzKSA8PSAtbG9hZGluZ190aHJlc2hvbGQsIF1bb3JkZXIoUEMxX21lYW4pXSRNRV9jbHVzdGVyCgojV2Vha05ldXJvbmFsIDwtIFBDQV9sb2FkaW5nc19zdGF0c1sgIHJvdW5kKFBDMV9tZWFuLCAzKSA8IGxvYWRpbmdfdGhyZXNob2xkICYgcm91bmQoUEMxX21lYW4sIDMpID49ICAwLjAxLCBdW29yZGVyKFBDMV9tZWFuKV0kTUVfY2x1c3RlcgoKTiA9IDEgCk5NID0gMQpOTiA9IDEKTSA9IDEKV04gPSAxCgpNRV9jbHVzdGVyX25hbWVzIDwtIGRhdGEudGFibGUoKQoKCmZvciAoTUVfY2x1c3RlciBpbiBOZXVyb25hbCl7CiAgCiAgaWYgKE1FX2NsdXN0ZXIgJWluJSBNdXNjdWxhcil7CiAgICAKICAgIE1FX2NsdXN0ZXIubmFtZSA9IHBhc3RlMCgiTk0iLCBOTSkKICAgIE1FX2NsdXN0ZXIudHlwZSA9ICJOZXVyby1NdXNjdWxhciIKICAgIAogICAgTUVfY2x1c3Rlcl9uYW1lcyA9IHJiaW5kKE1FX2NsdXN0ZXJfbmFtZXMsIGNiaW5kKE1FX2NsdXN0ZXIsIE1FX2NsdXN0ZXIubmFtZSwgTUVfY2x1c3Rlci50eXBlICApKQogICAgCiAgICBOTSA9IE5NICsgMQogICAgCiAgfQoKICAKICBlbHNlIHsKICAgIAogICAgTUVfY2x1c3Rlci5uYW1lID0gcGFzdGUwKCJOIiwgTikKICAgIE1FX2NsdXN0ZXIudHlwZSA9ICJOZXVyb25hbCIKICAgIAogICAgTUVfY2x1c3Rlcl9uYW1lcyA9IHJiaW5kKE1FX2NsdXN0ZXJfbmFtZXMsICBjYmluZChNRV9jbHVzdGVyLCBNRV9jbHVzdGVyLm5hbWUsIE1FX2NsdXN0ZXIudHlwZSApKQogICAgCiAgICBOID0gTiArIDEKICAgIAogIH0KICAKfQoKCmZvciAoTUVfY2x1c3RlciBpbiBOb25OZXVyb25hbCkgewogIAoKICBNRV9jbHVzdGVyLm5hbWUgPSBwYXN0ZTAoIk5OIiwgTk4pCiAgTUVfY2x1c3Rlci50eXBlID0gIk5vbi1OZXVyb25hbCIKICAKICBNRV9jbHVzdGVyX25hbWVzID0gcmJpbmQoTUVfY2x1c3Rlcl9uYW1lcywgIGNiaW5kKE1FX2NsdXN0ZXIsIE1FX2NsdXN0ZXIubmFtZSwgTUVfY2x1c3Rlci50eXBlICkpCiAgCiAgTk4gPSBOTiArIDEKICAKICAKfQoKCiNmb3IgKE1FX2NsdXN0ZXIgaW4gV2Vha05ldXJvbmFsKSB7CiAgCgojICBNRV9jbHVzdGVyLm5hbWUgPSBwYXN0ZTAoIldOIiwgV04pCiMgIE1FX2NsdXN0ZXIudHlwZSA9ICJXZWFrLU5ldXJvbmFsIgogIAojICBNRV9jbHVzdGVyX25hbWVzID0gcmJpbmQoTUVfY2x1c3Rlcl9uYW1lcywgIGNiaW5kKE1FX2NsdXN0ZXIsIE1FX2NsdXN0ZXIubmFtZSwgTUVfY2x1c3Rlci50eXBlICkpCiAgCiMgIFdOID0gV04gKyAxCiAgCiAgCiN9CgoKCmZvciAoTUVfY2x1c3RlciBpbiBNdXNjdWxhcil7CiAgCiAgCiAgaWYgKCAoTUVfY2x1c3RlciAlaW4lIE1FX2NsdXN0ZXJfbmFtZXMkTUVfY2x1c3Rlcik9PUYpIHsKICAKICAKICBNRV9jbHVzdGVyLm5hbWUgPSBwYXN0ZTAoIk0iLCBNKQogIE1FX2NsdXN0ZXIudHlwZSA9ICJNdXNjdWxhciIKICAKICBNRV9jbHVzdGVyX25hbWVzID0gcmJpbmQoTUVfY2x1c3Rlcl9uYW1lcywgIGNiaW5kKE1FX2NsdXN0ZXIsIE1FX2NsdXN0ZXIubmFtZSwgTUVfY2x1c3Rlci50eXBlICkpCiAgCiAgTSA9IE0gKyAxCiAgCiAgfQogIAogIAogIH0KCgpNRV9jbHVzdGVyX25hbWVzCgoKTUVfY2x1c3RlcnNfbWVhbl9QU0lzIDwtICBNRV9jbHVzdGVyc19QU0lbLCAuKG1lYW5fUFNJPW1lYW4oUFNJKSwgc2RfUFNJPXNkKFBTSSkpLCBieT0iTUVfY2x1c3RlcnMiIF0KCk1FX2NsdXN0ZXJzX2ZsYXQgPC0gTUVfY2x1c3RlcnNfbWVhbl9QU0lzWyAhIE1FX2NsdXN0ZXJzICVpbiUgTUVfY2x1c3Rlcl9uYW1lcyRNRV9jbHVzdGVyLCAgXQoKCkUgPSAxCkkgPSAxCk8gPSAxIAoKCgpmb3IgKE1FX2NsdXN0ZXIgaW4gTUVfY2x1c3RlcnNfZmxhdFsgbWVhbl9QU0kgPD0gMS8zLCAgXVtvcmRlcihtZWFuX1BTSSldJE1FX2NsdXN0ZXJzKSB7CiAgCiAgCiAgTUVfY2x1c3Rlci5uYW1lID0gcGFzdGUwKCJFIiwgRSkKICBNRV9jbHVzdGVyLnR5cGUgPSAiRXhjbHVkZWQiCiAgCiAgTUVfY2x1c3Rlcl9uYW1lcyA9IHJiaW5kKE1FX2NsdXN0ZXJfbmFtZXMsICBjYmluZChNRV9jbHVzdGVyLCBNRV9jbHVzdGVyLm5hbWUsIE1FX2NsdXN0ZXIudHlwZSApKQogIAogIEUgPSBFICsgMQogIAogIAp9IAoKCgpmb3IgKE1FX2NsdXN0ZXIgaW4gTUVfY2x1c3RlcnNfZmxhdFsgbWVhbl9QU0k+PTIvMywgIF1bb3JkZXIoLW1lYW5fUFNJKV0kTUVfY2x1c3RlcnMpIHsKICAKICAKICBNRV9jbHVzdGVyLm5hbWUgPSBwYXN0ZTAoIkkiLCBJKQogIE1FX2NsdXN0ZXIudHlwZSA9ICJJbmNsdWRlZCIKICAKICBNRV9jbHVzdGVyX25hbWVzID0gcmJpbmQoTUVfY2x1c3Rlcl9uYW1lcywgIGNiaW5kKE1FX2NsdXN0ZXIsIE1FX2NsdXN0ZXIubmFtZSwgTUVfY2x1c3Rlci50eXBlICkpCiAgCiAgSSA9IEkgKyAxCiAgCiAgCn0KCgoKCmZvciAoTUVfY2x1c3RlciBpbiBNRV9jbHVzdGVyc19mbGF0WyBtZWFuX1BTSSA+IDEvMyAgJiAgbWVhbl9QU0kgPCAyLzMgICwgIF1bb3JkZXIobWVhbl9QU0kpXSRNRV9jbHVzdGVycykgewogIAogIAogIE1FX2NsdXN0ZXIubmFtZSA9IHBhc3RlMCgiTyIsIE8pCiAgTUVfY2x1c3Rlci50eXBlID0gIk90aGVyIgogIAogIE1FX2NsdXN0ZXJfbmFtZXMgPSByYmluZChNRV9jbHVzdGVyX25hbWVzLCAgY2JpbmQoTUVfY2x1c3RlciwgTUVfY2x1c3Rlci5uYW1lLCBNRV9jbHVzdGVyLnR5cGUgKSkKICAKICBPID0gTyArIDEKICAKICAKfSAKTUVfY2x1c3Rlcl9uYW1lcwpgYGAKCgoKYGBge3J9CgpNRV9jbHVzdGVyX25hbWVzJE1FX2NsdXN0ZXIgPC0gYXMubnVtZXJpYyhNRV9jbHVzdGVyX25hbWVzJE1FX2NsdXN0ZXIpCgpNRV9jbHVzdGVyX25hbWVzIDwtIG1lcmdlKCB4PU1FX2NsdXN0ZXJfbmFtZXMsIHk9TUVfY2x1c3RlcnNfbWVhbl9QU0lzLCBieS54PSJNRV9jbHVzdGVyIiwgYnkueT0iTUVfY2x1c3RlcnMiKQoKUENBX2xvYWRpbmdzX3N0YXRzJE1FX2NsdXN0ZXIgPC0gYXMubnVtZXJpYyhhcy5jaGFyYWN0ZXIoUENBX2xvYWRpbmdzX3N0YXRzJE1FX2NsdXN0ZXIpKQoKUENBX2xvYWRpbmdzX3N0YXRzX1BDMiRNRV9jbHVzdGVyIDwtIGFzLm51bWVyaWMoYXMuY2hhcmFjdGVyKFBDQV9sb2FkaW5nc19zdGF0c19QQzIkTUVfY2x1c3RlcikpCgoKTUVfY2x1c3Rlcl9uYW1lcyA8LSBtZXJnZShNRV9jbHVzdGVyX25hbWVzLCBQQ0FfbG9hZGluZ3Nfc3RhdHMsIGJ5PSJNRV9jbHVzdGVyIikKI01FX2NsdXN0ZXJfbmFtZXMgPC0gbWVyZ2UoTUVfY2x1c3Rlcl9uYW1lcywgUENBX2xvYWRpbmdzX3N0YXRzX1BDMiwgYnk9Ik1FX2NsdXN0ZXIiKQoKTUVfY2x1c3Rlcl9uYW1lcyRNRV9jbHVzdGVyLnR5cGUgPC0gZmFjdG9yKE1FX2NsdXN0ZXJfbmFtZXMkTUVfY2x1c3Rlci50eXBlLCBsZXZlbHMgPSBjKCJFeGNsdWRlZCIsICJJbmNsdWRlZCIsICJOZXVyb25hbCIsICAiTm9uLU5ldXJvbmFsIiwgIk11c2N1bGFyIiwgICJOZXVyby1NdXNjdWxhciIsICJPdGhlciIpKQoKCgpgYGAKCgoKCmBgYHtyfQpGaWc1LlguMiA8LSBnZ3Bsb3QoTUVfY2x1c3Rlcl9uYW1lcykgKwogIGdlb21fdGV4dChhZXMoeD1QQzFfbWVhbiwgeT0tUEMyX21lYW4sIGxhYmVsID0gTUVfY2x1c3Rlci5uYW1lLCBjb2xvdXIgPSBNRV9jbHVzdGVyLnR5cGUgKSwgKSArCiAgeGxhYigiTWVhbiBQQzEgbG9hZGluZyIpICsKICB5bGFiKCJNZWFuIFBDMiBsb2FkaW5nIikgKwogICAgdGhlbWUobGVnZW5kLnBvc2l0aW9uPSJ0b3AiKQoKCkZpZzUuWC4yCgpgYGAKCgpgYGB7cn0KRmlnNS5YLjMgPC0gZ2dwbG90KE1FX2NsdXN0ZXJfbmFtZXMpICsKICBnZW9tX3RleHQoYWVzKHg9bWVhbl9QU0ksIHk9c2RfUFNJLCBsYWJlbCA9IE1FX2NsdXN0ZXIubmFtZSwgY29sb3VyID0gTUVfY2x1c3Rlci50eXBlICksICkgKwogIHhsYWIoIk1lYW4gUFNJIikgKwogIHlsYWIoIlBTSSBzdGFuZGFyIGRldmlhdGlvbiIpICsKICAgIHRoZW1lKGxlZ2VuZC5wb3NpdGlvbj0idG9wIikgCiAgCgoKRmlnNS5YLjMKYGBgCgoKCgoKCmBgYHtyfQoKCk1FX2NsdXN0ZXJzLnRhYmxlIDwtIGNiaW5kKE1FX2NsdXN0ZXJzLCBuYW1lcyhNRV9jbHVzdGVycykpCmNvbG5hbWVzKE1FX2NsdXN0ZXJzLnRhYmxlKSA8LSBjKCJNRV9jbHVzdGVycyIsICJNRSIpCgpNRV9jbHVzdGVycy50YWJsZSA8LSBkYXRhLnRhYmxlKE1FX2NsdXN0ZXJzLnRhYmxlKQpNRV9jbHVzdGVycy50YWJsZSRNRV9jbHVzdGVycyA8LSBhcy5udW1lcmljKE1FX2NsdXN0ZXJzLnRhYmxlJE1FX2NsdXN0ZXJzKQoKCk1FX2NsdXN0ZXJfaW5mbyA8LSBtZXJnZShNRV9jbHVzdGVycy50YWJsZSwgTUVfZmluYWxbLCBjKCJNRSIsICJsZW5fbWljcm9fZXhvbl9zZXFfZm91bmQiLCAiVTJfc2NvcmVzIildLCBieT0iTUUiKQpNRV9jbHVzdGVyX2luZm8gPC0gZGF0YS50YWJsZShNRV9jbHVzdGVyX2luZm8pCgoKTUVfY2x1c3Rlcl9pbmZvX2J5IDwtIE1FX2NsdXN0ZXJfaW5mb1sgLCAuKGFzeW0gPSBzdW0obGVuX21pY3JvX2V4b25fc2VxX2ZvdW5kICUlIDMpLAogICAgICAgICAgICAgICAgICAgIG1lYW5fVTJfc2NvcmUgPSBtZWFuKFUyX3Njb3JlcyksCiAgICAgICAgICAgICAgICAgICAgdG90YWw9Lk4KICAgICAgICAgICAgICAgICAgICApLCBieT0iTUVfY2x1c3RlcnMiICBdCgpNRV9jbHVzdGVyX2luZm9fYnlbLCBzeW1ldHJpY2FsX2ZyYWN0aW9uOj0odG90YWwtYXN5bSkvdG90YWxdCgoKTUVfY2x1c3Rlcl9uYW1lcyA8LSBtZXJnZShNRV9jbHVzdGVyX25hbWVzLCBNRV9jbHVzdGVyX2luZm9fYnksIGJ5Lng9Ik1FX2NsdXN0ZXIiLCBieS55PSJNRV9jbHVzdGVycyIpCgoKCgojTUVfY2x1c3Rlcl9pbmZvIDwtIG1lcmdlKE1FX2NsdXN0ZXJfaW5mb19ieVssIC4oTUVfY2x1c3RlcnM9dW5pcXVlKE1FX2NsdXN0ZXJzKSksIGJ5PU1FXSwgTUVfZmluYWxbLCBjKCJNRSIsICJsZW5fbWljcm9fZXhvbl9zZXFfZm91bmQiLCAiVTJfc2NvcmVzIildLCBieT0iTUUiKQoKCiAKCgoKCgoKRmlnNS5YLjQgPC0gZ2dwbG90KE1FX2NsdXN0ZXJfbmFtZXMpICsKICBnZW9tX3RleHQoYWVzKHg9c3ltZXRyaWNhbF9mcmFjdGlvbiwgeT1tZWFuX1UyX3Njb3JlLCBsYWJlbCA9IE1FX2NsdXN0ZXIubmFtZSwgY29sb3VyID0gTUVfY2x1c3Rlci50eXBlICksICkgKwogIHhsYWIoIkluLWZyYW1lIG1pY3JvZXhvbiBmcmFjdGlvbiIpICsKICB5bGFiKCJNZWFuIHNwbGljaW5nIHN0cmVuZ3RoIikgKwogICAgdGhlbWUobGVnZW5kLnBvc2l0aW9uPSJ0b3AiKQoKCkZpZzUuWC40CiAgCmBgYAoKCmBgYHtyfQpNRV9jbHVzdGVyX25hbWVzWyBNRV9jbHVzdGVyLnR5cGU9PSJFeGNsdWRlZCIsIF0KTUVfY2x1c3Rlcl9uYW1lc1sgTUVfY2x1c3Rlci50eXBlPT0iSW5jbHVkZWQiLCBdCk1FX2NsdXN0ZXJfbmFtZXNbIE1FX2NsdXN0ZXIudHlwZT09Ik5ldXJvbmFsIiwgXQpNRV9jbHVzdGVyX25hbWVzWyBNRV9jbHVzdGVyLnR5cGU9PSJOZXVyby1NdXNjdWxhciIsIF0KTUVfY2x1c3Rlcl9uYW1lc1sgTUVfY2x1c3Rlci50eXBlPT0iTmV1cm9uYWwiLCBdCk1FX2NsdXN0ZXJfbmFtZXNbIE1FX2NsdXN0ZXIudHlwZT09IkluY2x1ZGVkIiwgXQpNRV9jbHVzdGVyX25hbWVzWyBNRV9jbHVzdGVyLnR5cGU9PSJFeGNsdWRlZCIsIF0KTUVfY2x1c3Rlcl9uYW1lc1sgTUVfY2x1c3Rlci50eXBlPT0iSW5jbHVkZWQiLCBdCk1FX2NsdXN0ZXJfbmFtZXNbIE1FX2NsdXN0ZXIudHlwZT09Ik90aGVyIiwgXQoKbGV2ZWxzKGZhY3RvcihNRV9jbHVzdGVyX25hbWVzJE1FX2NsdXN0ZXIudHlwZSkpCmBgYAoKYGBge3J9Ck1FX2NsdXN0ZXJfbmFtZXMubGV2ZWxzIDwtIGMoKQoKZm9yIChpIGluIGxldmVscyhmYWN0b3IoTUVfY2x1c3Rlcl9uYW1lcyRNRV9jbHVzdGVyLnR5cGUpKSl7CiAgCiAgCiAgCiAgCiAgTUVfY2x1c3Rlcl9uYW1lcy5sZXZlbHM8LSBjKE1FX2NsdXN0ZXJfbmFtZXMubGV2ZWxzLCBNRV9jbHVzdGVyX25hbWVzW01FX2NsdXN0ZXIudHlwZT09aSwgTUVfY2x1c3Rlci5uYW1lXSkKICAKICAKfQpgYGAKCgpgYGB7cn0KCk1FX2NsdXN0ZXJfbmFtZXMkTUVfY2x1c3Rlci5uYW1lIDwtIGZhY3RvcihNRV9jbHVzdGVyX25hbWVzJE1FX2NsdXN0ZXIubmFtZSwgCiAgICAgICBsZXZlbHMgPSBNRV9jbHVzdGVyX25hbWVzLmxldmVscykKCkZpZzUuWC4xIDwtIGdncGxvdChNRV9jbHVzdGVyX25hbWVzKSArCiAgZ2VvbV9iYXIoYWVzKE1FX2NsdXN0ZXIubmFtZSwgdG90YWwsICBmaWxsID0gTUVfY2x1c3Rlci50eXBlICksIHN0YXQgPSAiaWRlbnRpdHkiKSArCiAgdGhlbWUobGVnZW5kLnBvc2l0aW9uPSJ0b3AiKSArIAogIHhsYWIoIk51bWJlciBvZiBtaWNyb2V4b25zIikgKwogIHlsYWIoIk1pY3JvZXhvbiBjbHVzdGVyIikgKwogIHRoZW1lKGF4aXMudGV4dC54ID0gZWxlbWVudF90ZXh0KGFuZ2xlID0gNDUpKSArCiAgICBzY2FsZV9maWxsX2Rpc2NyZXRlKG5hbWU9Ik1pY3JvZXhvbiBjbHVzdGVyIGNsYXNzIikgCgpGaWc1LlguMQoKCkZpZzUuWC4xLmxlZ2VuZCA8LSBnZXRfbGVnZW5kKEZpZzUuWC4xICsgCiAgICAgICAgICAgICAgICAgICAgICAgICAgICAgIHRoZW1lKGxlZ2VuZC5kaXJlY3Rpb24gPSAiaG9yaXpvbnRhbCIsbGVnZW5kLmp1c3RpZmljYXRpb249ImNlbnRlciIgLGxlZ2VuZC5ib3guanVzdCA9ICJib3R0b20iKSArCiAgICAgICAgICAgICAgICAgICAgICAgICAgICAgIGd1aWRlcyhmaWxsPWd1aWRlX2xlZ2VuZChucm93PTEsYnlyb3c9VFJVRSkpKQpgYGAKCgoKCgpgYGB7ciwgZmlnLmhlaWdodD01LCBmaWcud2lkdGg9MjB9CgpGaWc1LlgucGxvdHMgPC0gcGxvdF9ncmlkKEZpZzUuWC4xICsgdGhlbWUobGVnZW5kLnBvc2l0aW9uPSJub25lIiksIAogICAgICAgICAgICAgICAgICAgIEZpZzUuWC4yICsgdGhlbWUobGVnZW5kLnBvc2l0aW9uPSJub25lIiksIAogICAgICAgICAgICAgICAgICAgIEZpZzUuWC4zICsgdGhlbWUobGVnZW5kLnBvc2l0aW9uPSJub25lIiksIAogICAgICAgICAgICAgICAgICAgIEZpZzUuWC40ICsgdGhlbWUobGVnZW5kLnBvc2l0aW9uPSJub25lIiksIAogICAgICAgICAgICAgICAgICAgIG5yb3cgPTEsCiAgICAgICAgICAgICAgICAgICAgcmVsX3dpZHRocyA9IGMoMS43LDEsMSwxKSwKICAgICAgICAgICAgICAgICAgICBsYWJlbHMgPSAiQVVUTyIgKQoKCkZpZzUuWCA8LSBwbG90X2dyaWQoRmlnNS5YLjEubGVnZW5kLCBGaWc1LlgucGxvdHMsIG5jb2wgPSAxLCByZWxfaGVpZ2h0cyA9IGMoMC4yLCAxKSkKCkZpZzUuWApgYGAKCgoKCgpgYGB7cn0KTUVfY2x1c3RlcnMubmFtZXMgPC0gZGF0YS50YWJsZShNRV9jbHVzdGVycykKTUVfY2x1c3RlcnMubmFtZXMkTUUgPC0gbmFtZXMoTUVfY2x1c3RlcnMpCk1FX2NsdXN0ZXJzLm5hbWVzIDwtIG1lcmdlKE1FX2NsdXN0ZXJzLm5hbWVzLCAgTUVfY2x1c3Rlcl9uYW1lc1ssIGMoIk1FX2NsdXN0ZXIiLCAiTUVfY2x1c3Rlci5uYW1lIildLCAgYnkueD0iTUVfY2x1c3RlcnMiLCBieS55PSJNRV9jbHVzdGVyIikKCgpgYGAKCgpgYGB7cn0KTUVfZmluYWwuTUVfY2x1c3RlcnMgPC0gbWVyZ2UoTUVfZmluYWwsIE1FX2NsdXN0ZXJzLm5hbWVzLCBieT0iTUUiKQoKTUVfY2x1c3Rlci5jb25zZXJ2YXRpb24gPC0gTUVfZmluYWwuTUVfY2x1c3RlcnNbIG1lYW5fY29uc2VydmF0aW9uc192ZXJ0ZWJyYXRlcyE9Ik5vbmUiICwgIC4obWVhbl9jb25zZXJ2YXRpb249bWVhbihhcy5udW1lcmljKG1lYW5fY29uc2VydmF0aW9uc192ZXJ0ZWJyYXRlcykpKSAgLCBieT1jKCJNRV9jbHVzdGVycyIsICJNRV9jbHVzdGVyLm5hbWUiKV0KCgpNRV9jbHVzdGVyX25hbWVzIDwtICBtZXJnZShNRV9jbHVzdGVyX25hbWVzLCBNRV9jbHVzdGVyLmNvbnNlcnZhdGlvbiwgIGJ5Lng9YygiTUVfY2x1c3RlciIsICJNRV9jbHVzdGVyLm5hbWUiKSwgYnkueT1jKCJNRV9jbHVzdGVycyIsICJNRV9jbHVzdGVyLm5hbWUiKSkKCnJvdW5kKGNvcihNRV9jbHVzdGVyX25hbWVzJHN5bWV0cmljYWxfZnJhY3Rpb24sIE1FX2NsdXN0ZXJfbmFtZXMkbWVhbl9jb25zZXJ2YXRpb24sIG1ldGhvZD0icGVhcnNvbiIpLCAyKQoKCmdncGxvdChNRV9jbHVzdGVyX25hbWVzLCBhZXMoeD1zeW1ldHJpY2FsX2ZyYWN0aW9uLCB5PW1lYW5fY29uc2VydmF0aW9uKSkgKwogIGdlb21fc21vb3RoKG1ldGhvZD1sbSwgbGluZXR5cGU9ImRhc2hlZCIpICsKICBnZW9tX3RleHQoYWVzKCBsYWJlbCA9IE1FX2NsdXN0ZXIubmFtZSwgY29sb3VyID0gTUVfY2x1c3Rlci50eXBlICkgKSArCiAgeGxhYigiSW4tZnJhbWUgbWljcm9leG9uIGZyYWN0aW9uIikgKwogIHlsYWIoIk1lYW4gUGh5bG9wIGNvbnNlcnZhdGlvbiBzY29yZSIpICsKICB0aGVtZShsZWdlbmQucG9zaXRpb249InRvcCIpICsKIGxhYnMoY29sb3IgPSAiTWljcm9leG9uIGNsdXN0ZXIgY2xhc3MiKQoKCgpgYGAKCgoKYGBge3J9Cgpzb21lX01FX2NsdXN0ZXJzIDwtYygKTUVfY2x1c3RlcnNbTUVfY2x1c3RlcnM9PTFdWzFdLApNRV9jbHVzdGVyc1tNRV9jbHVzdGVycz09Ml1bMV0sCk1FX2NsdXN0ZXJzW01FX2NsdXN0ZXJzPT0zXVsxXSwKTUVfY2x1c3RlcnNbTUVfY2x1c3RlcnM9PTRdWzFdLApNRV9jbHVzdGVyc1tNRV9jbHVzdGVycz09NV1bMV0sCk1FX2NsdXN0ZXJzW01FX2NsdXN0ZXJzPT02XVsxXSwKTUVfY2x1c3RlcnNbTUVfY2x1c3RlcnM9PTddWzFdLApNRV9jbHVzdGVyc1tNRV9jbHVzdGVycz09OF1bMV0sCk1FX2NsdXN0ZXJzW01FX2NsdXN0ZXJzPT05XVsxXSwKTUVfY2x1c3RlcnNbTUVfY2x1c3RlcnM9PTEwXVsxXSwKTUVfY2x1c3RlcnNbTUVfY2x1c3RlcnM9PTExXVsxXSwKTUVfY2x1c3RlcnNbTUVfY2x1c3RlcnM9PTEyXVsxXSwKTUVfY2x1c3RlcnNbTUVfY2x1c3RlcnM9PTEzXVsxXSwKTUVfY2x1c3RlcnNbTUVfY2x1c3RlcnM9PTE0XVsxXSwKTUVfY2x1c3RlcnNbTUVfY2x1c3RlcnM9PTE1XVsxXSwKTUVfY2x1c3RlcnNbTUVfY2x1c3RlcnM9PTE2XVsxXSwKTUVfY2x1c3RlcnNbTUVfY2x1c3RlcnM9PTE3XVsxXSwKTUVfY2x1c3RlcnNbTUVfY2x1c3RlcnM9PTE4XVsxXSwKTUVfY2x1c3RlcnNbTUVfY2x1c3RlcnM9PTE5XVsxXSwKTUVfY2x1c3RlcnNbTUVfY2x1c3RlcnM9PTIwXVsxXSwKTUVfY2x1c3RlcnNbTUVfY2x1c3RlcnM9PTIxXVsxXSwKTUVfY2x1c3RlcnNbTUVfY2x1c3RlcnM9PTIyXVsxXSwKTUVfY2x1c3RlcnNbTUVfY2x1c3RlcnM9PTIzXVsxXSwKTUVfY2x1c3RlcnNbTUVfY2x1c3RlcnM9PTI0XVsxXQoKKQpgYGAKCgoKCmBgYHtyfQoKCmNvbF9hbm4gPC0gZGF0YS5mcmFtZSggIFRpc3N1ZV9jbHVzdGVycyRjbHVzdGVyLCByb3cubmFtZXM9VGlzc3VlX2NsdXN0ZXJzJEZpbGUuYWNjZXNzaW9uKQpjb2xuYW1lcyhjb2xfYW5uKSA8LSAiVGlzc3VlIGNsdXN0ZXJzIgoKcm93bmFtZXMoY29sX2FubikgPC0gVGlzc3VlX2NsdXN0ZXJzJEZpbGUuYWNjZXNzaW9uCgoKCnRpc3N1ZV9jbHVzdGVyX3R5cGUgPSBkYXRhLmZyYW1lKGNsdXN0ZXI9YygxLCA2LCA4LCAxNCwgMTIsIDcsIDE1LCA1LCAxMSwgNCwgOSwgMywgMTMsIDEwLCAxNiwgMiksIAogICAgICAgICAgICAgICAgICAgICAgICAgICAgICAgICBgVGlzc3VlIGNsdXN0ZXIgdHlwZWA9YygiTm9uLW5ldXJvbmFsIiwKICAgICAgICAgICAgICAgICAgICAgICAgICAgICAgICAgICAgICAgICAgICAgICAgIk5vbi1uZXVyb25hbCIsCiAgICAgICAgICAgICAgICAgICAgICAgICAgICAgICAgICAgICAgICAgICAgICAgICJOb24tbmV1cm9uYWwiLAogICAgICAgICAgICAgICAgICAgICAgICAgICAgICAgICAgICAgICAgICAgICAgICAiTm9uLW5ldXJvbmFsIiwKICAgICAgICAgICAgICAgICAgICAgICAgICAgICAgICAgICAgICAgICAgICAgICAgIkVtYnJ5b25pYyBuZXVyb25hbCIsCiAgICAgICAgICAgICAgICAgICAgICAgICAgICAgICAgICAgICAgICAgICAgICAgICJBZHJlbmFsIGdsYW5kIiwKICAgICAgICAgICAgICAgICAgICAgICAgICAgICAgICAgICAgICAgICAgICAgICAgIlNrZWxldGFsIG11c2NsZSIsCiAgICAgICAgICAgICAgICAgICAgICAgICAgICAgICAgICAgICAgICAgICAgICAgICJIZWFydCIsCiAgICAgICAgICAgICAgICAgICAgICAgICAgICAgICAgICAgICAgICAgICAgICAgICJFbWJyeW9uaWMgbmV1cm9uYWwiLAogICAgICAgICAgICAgICAgICAgICAgICAgICAgICAgICAgICAgICAgICAgICAgICAiRW1icnlvbmljIG5ldXJvbmFsIiwKICAgICAgICAgICAgICAgICAgICAgICAgICAgICAgICAgICAgICAgICAgICAgICAgIkVtYnJ5b25pYyBuZXVyb25hbCIsCiAgICAgICAgICAgICAgICAgICAgICAgICAgICAgICAgICAgICAgICAgICAgICAgICJQb3N0bmF0YWwgbmV1cm9uYWwiLAogICAgICAgICAgICAgICAgICAgICAgICAgICAgICAgICAgICAgICAgICAgICAgICAiRW1icnlvbmljIG5ldXJvbmFsIiwKICAgICAgICAgICAgICAgICAgICAgICAgICAgICAgICAgICAgICAgICAgICAgICAgIlBvc3RuYXRhbCBuZXVyb25hbCIsCiAgICAgICAgICAgICAgICAgICAgICAgICAgICAgICAgICAgICAgICAgICAgICAgICJQb3N0bmF0YWwgbmV1cm9uYWwiLAogICAgICAgICAgICAgICAgICAgICAgICAgICAgICAgICAgICAgICAgICAgICAgICAiUG9zdG5hdGFsIG5ldXJvbmFsIikpCgpjb2xfYW5uJEZpbGUgPC0gcm93bmFtZXMoY29sX2FubikKY29sX2FubiA8LSBtZXJnZShjb2xfYW5uLCB0aXNzdWVfY2x1c3Rlcl90eXBlLCBieS54PSJUaXNzdWUgY2x1c3RlcnMiLGJ5Lnk9ImNsdXN0ZXIiKQpyb3duYW1lcyhjb2xfYW5uKSA8LSBjb2xfYW5uJEZpbGUKCmNvbG5hbWVzKGNvbF9hbm4pIDwtIGMoIlRpc3N1ZSBjbHVzdGVycyIsICJGaWxlIiwgIlRpc3N1ZSBjbHVzdGVyIHR5cGUiKQoKCgphbm5fY29sb3JzID0gbGlzdCgKICAgIGNsdXN0ZXIgPSBjKGJsYWNrPSJibGFjayIpLAogICAgYFRpc3N1ZS5jbHVzdGVyLnR5cGVgID0gIGMoYEFkcmVuYWwgZ2xhbmRgID0gIiNCNzlGMDAiLAogICAgICAgICAgICAgICAgICAgICAgYFNrZWxldGFsIG11c2NsZWAgPSAiIzAwQkZDNCIsCiAgICAgICAgICAgICAgICAgICAgICBgSGVhcnRgID0gIiNGNTY0RTMiLAogICAgICAgICAgICAgICAgICAgICAgYE5vbi1uZXVyb25hbGAgPSAiI0E1OEFGRiIsCiAgICAgICAgICAgICAgICAgICAgICBgRW1icnlvbmljIG5ldXJvbmFsYCA9ICIjMDBCQTM4IiwKICAgICAgICAgICAgICAgICAgICAgIGBQb3N0bmF0YWwgbmV1cm9uYWxgID0gIiNGODc2NkQiCiAgICAgICAgICAgICAgICAgICAgICApLAogICAgR2VuZUNsYXNzID0gYyhQYXRoMSA9ICIjNzU3MEIzIiwgUGF0aDIgPSAiI0U3Mjk4QSIsIFBhdGgzID0gIiM2NkE2MUUiKQopCgpjb2xfYW5uJGBUaXNzdWUgY2x1c3RlciB0eXBlYCA8LSBmYWN0b3IoY29sX2FubiRgVGlzc3VlIGNsdXN0ZXIgdHlwZWAgLCBsZXZlbHMgPSBjKCJBZHJlbmFsIGdsYW5kIiwgIlNrZWxldGFsIG11c2NsZSIsICJIZWFydCIsICJOb24tbmV1cm9uYWwiLCAiSW50ZXJtZWRpYXRlIiwgIkVtYnJ5b25pYyBuZXVyb25hbCIsICJQb3N0bmF0YWwgbmV1cm9uYWwiKSkKCmZha2VfcGxvdCA8LSBnZ3Bsb3QoY29sX2FubiwgYWVzKGFzLm51bWVyaWMoYFRpc3N1ZSBjbHVzdGVyc2ApLCBgVGlzc3VlIGNsdXN0ZXJzYCwgZmlsbD1gVGlzc3VlIGNsdXN0ZXIgdHlwZWAgKSkgKwogIGdlb21fYmFyKHN0YXQgPSAiaWRlbnRpdHkiKSArCiAgc2NhbGVfZmlsbF9tYW51YWwodmFsdWVzPWMoIiNCNzlGMDAiLCAiIzAwQkZDNCIsICIjRjU2NEUzIiwgIiNBNThBRkYiLCAiIzAwQkEzOCIsICIjRjg3NjZEIiApLCBuYW1lPSAiVGlzc3VlIGNsdXN0ZXIgdHlwZSIpICsKICAgdGhlbWUobGVnZW5kLnBvc2l0aW9uPSJ0b3AiLCBsZWdlbmQuanVzdGlmaWNhdGlvbj0iY2VudGVyIikgKwogIGd1aWRlcyhmaWxsPWd1aWRlX2xlZ2VuZChucm93PTEsYnlyb3c9VFJVRSkpIAoKCmZha2VfcGxvdC5sZWdlbmQgPC0gZ2V0X2xlZ2VuZChmYWtlX3Bsb3QpCgoKCmBgYAoKCgpgYGB7ciwgZmlnLndpZHRoPTIwLCBmaWcuaGVpZ2h0PTE1fQoKbGlicmFyeShybGFuZykKCgoKI3Jvd25hbWVzKGNvbF9hbm4pIDwtIHJvd25hbWVzKFRpc3N1ZV9jbHVzdGVycykKClRpc3N1ZV9QU0lfbWF0cml4X2RjYXN0X3BwY2EgPC0gdChjT2JzKQoKVGlzc3VlX1BTSV9tYXRyaXhfZGNhc3RfcHBjYVtUaXNzdWVfUFNJX21hdHJpeF9kY2FzdF9wcGNhPjFdIDwtIDEKVGlzc3VlX1BTSV9tYXRyaXhfZGNhc3RfcHBjYVtUaXNzdWVfUFNJX21hdHJpeF9kY2FzdF9wcGNhPDBdIDwtIDAKCgpUaXNzdWVfUFNJX21hdHJpeF9kY2FzdF9wcGNhX2hlYXRtYXAgPC0gZHVwbGljYXRlKFRpc3N1ZV9QU0lfbWF0cml4X2RjYXN0X3BwY2EpCgoKc29tZV9NRV9jbHVzdGVyc19zcGFjZSA8LSBwYXN0ZSgiICAgICAgIiwgc29tZV9NRV9jbHVzdGVycywgc2VwID0gIiIpIApuYW1lcyhzb21lX01FX2NsdXN0ZXJzX3NwYWNlKSA8LSBuYW1lcyhzb21lX01FX2NsdXN0ZXJzKQoKcm93Lm5hbWVzKFRpc3N1ZV9QU0lfbWF0cml4X2RjYXN0X3BwY2FfaGVhdG1hcCkgPC0gc29tZV9NRV9jbHVzdGVyc19zcGFjZVtyb3cubmFtZXMoVGlzc3VlX1BTSV9tYXRyaXhfZGNhc3RfcHBjYV9oZWF0bWFwKV0KCgoKI3Rpc3N1ZV9oZWF0bWFwIDwtIHBoZWF0bWFwOjpwaGVhdG1hcChUaXNzdWVfUFNJX21hdHJpeF9kY2FzdF9wcGNhX2hlYXRtYXAsICAsIGZvbnRzaXplID0gMTAgLCAgY3V0cmVlX3Jvd3MgPSAyNCwgY3V0cmVlX2NvbHMgPSAxNiwgY2x1c3RlcmluZ19tZXRob2QgPSAid2FyZC5EMiIgLCBzaG93X2NvbG5hbWVzPUZBTFNFLCBhbm5vdGF0aW9uX2NvbD0gY29sX2FubiwgYW5ub3RhdGlvbl9uYW1lc19jb2w9RkFMU0UsIHRyZWVoZWlnaHRfY29sPTIwMCwgdHJlZWhlaWdodF9yb3c9MTAwKQoKYGBgCgoKCgpgYGB7ciwgZmlnLndpZHRoPTEwLCBmaWcuaGVpZ2h0PTd9CgoKCgoKY29sX2Fubl8xYyA8LSBkYXRhLmZyYW1lKGNsdXN0ZXI9cmVwKCJibGFjayIgLG5yb3coY29sX2FubikpKSAKcm93bmFtZXMoY29sX2FubikgPC0gcm93bmFtZXMoY29sX2FubikKCgpjb2xfYW5uIDwtbWVyZ2UoY29sX2FubiwgVGlzc3VlX2NsdXN0ZXJzLCBieS54PSJGaWxlIiwgYnkueT0iRmlsZS5hY2Nlc3Npb24iKQoKY29sX2FubiA8LSBkYXRhLnRhYmxlKGNvbF9hbm4pCgpjb2xfYW5uWyFuYW1lICVpbiUgYygiZm9yZWJyYWluIiwgImhpbmRicmFpbiIsICJtaWRicmFpbiIsICJuZXVyYWwgdHViZSIsICJ3aG9sZSBjb3J0ZXgiKSwgYFRpc3N1ZSBjbHVzdGVyIHR5cGVgOj0iTm9uLW5ldXJvbmFsIl0KY29sX2FubltuYW1lPT0iaGVhcnQiLCBgVGlzc3VlIGNsdXN0ZXIgdHlwZWA6PSJIZWFydCJdCmNvbF9hbm5bbmFtZT09InNrZWxldGFsIG11c2NsZSB0aXNzdWUiLCBgVGlzc3VlIGNsdXN0ZXIgdHlwZWA6PSJTa2VsZXRhbCBtdXNjbGUiXQpjb2xfYW5uW25hbWU9PSJhZHJlbmFsIGdsYW5kIiwgYFRpc3N1ZSBjbHVzdGVyIHR5cGVgOj0iQWRyZW5hbCBnbGFuZCJdCmNvbF9hbm5bKG5hbWUgJWluJSBjKCJmb3JlYnJhaW4iLCAiaGluZGJyYWluIiwgIm1pZGJyYWluIiwgIm5ldXJhbCB0dWJlIiwgIndob2xlIGNvcnRleCIpKSAmIGFnZSAlaW4lIGMoMTAuNSwgMTEuNSwgMTIuNSwgMTMuNSwgMTQuNSwgMTUuNSwgMTYuNSkgLCAgYFRpc3N1ZSBjbHVzdGVyIHR5cGVgOj0iRW1icnlvbmljIG5ldXJvbmFsIl0KY29sX2FublsobmFtZSAlaW4lIGMoImZvcmVicmFpbiIsICJoaW5kYnJhaW4iLCAibWlkYnJhaW4iLCAibmV1cmFsIHR1YmUiLCAid2hvbGUgY29ydGV4IikpICYgIWFnZSAlaW4lIGMoMTAuNSwgMTEuNSwgMTIuNSwgMTMuNSwgMTQuNSwgMTUuNSwgMTYuNSkgLCAgYFRpc3N1ZSBjbHVzdGVyIHR5cGVgOj0iUG9zdG5hdGFsIG5ldXJvbmFsIl0KCgpjb2xfYW5uIDwtIGRhdGEuZnJhbWUoY29sX2FubikKcm93bmFtZXMoY29sX2FubikgPC0gY29sX2FubiRGaWxlCgoKCgp0aXNzdWVfaGVhdG1hcCA8LSBwaGVhdG1hcDo6cGhlYXRtYXAoVGlzc3VlX1BTSV9tYXRyaXhfZGNhc3RfcHBjYV9oZWF0bWFwLCBjdXRyZWVfcm93cyA9IDI0LCBjdXRyZWVfY29scyA9IDE2LCBjbHVzdGVyaW5nX21ldGhvZCA9ICJ3YXJkLkQyIiAsIHNob3dfY29sbmFtZXM9RkFMU0UsIHNob3dfcm93bmFtZXM9RkFMU0UsIGFubm90YXRpb25fbGVnZW5kPUZBTFNFLCBhbm5vdGF0aW9uX2NvbD0gY29sX2Fubl8xYywgYW5ub3RhdGlvbl9uYW1lc19jb2w9RkFMU0UsIGFubm90YXRpb25fY29sb3JzPWFubl9jb2xvcnMpCgp0aXNzdWVfaGVhdG1hcCA8LSBwaGVhdG1hcDo6cGhlYXRtYXAoVGlzc3VlX1BTSV9tYXRyaXhfZGNhc3RfcHBjYV9oZWF0bWFwLCBjdXRyZWVfcm93cyA9IDI0LCBjdXRyZWVfY29scyA9IDE2LCBjbHVzdGVyaW5nX21ldGhvZCA9ICJ3YXJkLkQyIiAsIHNob3dfY29sbmFtZXM9RkFMU0UsIHNob3dfcm93bmFtZXM9RkFMU0UsIGFubm90YXRpb25fbGVnZW5kPUYsIGFubm90YXRpb25fY29sPSBjb2xfYW5uWyJUaXNzdWUuY2x1c3Rlci50eXBlIl0sIGFubm90YXRpb25fbmFtZXNfY29sPUYsIGFubm90YXRpb25fY29sb3JzPWFubl9jb2xvcnMpCgojODc3LjI0MQojNTcxLjQ5CgpwaGVhdG1hcDo6cGhlYXRtYXAoVGlzc3VlX1BTSV9tYXRyaXhfZGNhc3RfcHBjYV9oZWF0bWFwLCBjdXRyZWVfcm93cyA9IDI0LCBjdXRyZWVfY29scyA9IDE2LCBjbHVzdGVyaW5nX21ldGhvZCA9ICJ3YXJkLkQyIiAsIHNob3dfY29sbmFtZXM9RkFMU0UsIHNob3dfcm93bmFtZXM9RkFMU0UsIGFubm90YXRpb25fbGVnZW5kPVQsIGFubm90YXRpb25fY29sPSBjb2xfYW5uWyJUaXNzdWUuY2x1c3RlcnMiXSwgYW5ub3RhdGlvbl9uYW1lc19jb2w9VCwgYW5ub3RhdGlvbl9jb2xvcnM9YW5uX2NvbG9ycykKCgpgYGAKCgoKCmBgYHtyLCBmaWcud2lkdGg9MjAsIGZpZy5oZWlnaHQ9Mn0KcGxvdF9ncmlkKGZha2VfcGxvdC5sZWdlbmQpCmBgYAoKCgpgYGB7cn0KCgoKUENBIDwtIGRhdGEuZnJhbWUoc2NvcmVzKHJlc3VsdCkpCgoKCgoKUENBJEZpbGUuYWNjZXNzaW9uIDwtIHJvdy5uYW1lcyhQQ0EpClBDQSA8LSBkYXRhLnRhYmxlKFBDQSkKUENBIDwtIFBDQVssIGA6PWAoUEMxPS1QQzEpIF0KCgpQQ0EgPC0gbWVyZ2UoVGlzc3VlX2NsdXN0ZXJzLCBQQ0EsIGJ5PSJGaWxlLmFjY2Vzc2lvbiIpCgpQQ0EkYWdlIDwtIGFzLm51bWVyaWMoYXMuY2hhcmFjdGVyKFBDQSRhZ2UpKQoKCgpgYGAKCgoKCmBgYHtyfQpQQ0Ffc3RhdHMgPC0gUENBWyAsIC4obWVhbl9QQzE9bWVhbihQQzEpKSAsIGJ5PWMoIm5hbWUiLCAiYWdlIildCgoKU3VwX1BDQV9hZ2UuQiA8LSBnZ3Bsb3QoKSArCiAgZ2VvbV9qaXR0ZXIoZGF0YSA9IFBDQVtuYW1lICVpbiUgYygiZm9yZWJyYWluIiwgIm1pZGJyYWluIiwgImhpbmRicmFpbiIsICJuZXVyYWwgdHViZSIpLCBdLCAgYWVzKGFnZSwgUEMxLCBjb2xvdXI9bmFtZSksIHdpZHRoPTAuMSwgaGVpZ2h0ID0gMCApICsKICBnZW9tX2xpbmUoZGF0YSA9IFBDQV9zdGF0c1tuYW1lICVpbiUgYygiZm9yZWJyYWluIiwgIm1pZGJyYWluIiwgImhpbmRicmFpbiIsICJuZXVyYWwgdHViZSIpLCBdLCBhZXMoYWdlLCBtZWFuX1BDMSwgZ3JvdXA9bmFtZSwgY29sb3VyPW5hbWUpKSArCiAgbGFicyhjb2xvdXI9Ik5ldXJhbCB0aXNzdWUiKQoKCgpgYGAKCmBgYHtyfQpQQ0Ffc3RhdHM8LSBQQ0FbICwgLihtZWFuX1BDMT1tZWFuKFBDMSkpICwgYnk9YygibmFtZSIsICJhZ2UiKV0KCkZpZzQuQyA8LSBnZ3Bsb3QoKSArCiAgZ2VvbV9saW5lKGRhdGEgPSBQQ0Ffc3RhdHNbbmFtZSAlaW4lIGMoImZvcmVicmFpbiIsICJtaWRicmFpbiIsICJoaW5kYnJhaW4iLCAibmV1cmFsIHR1YmUiLCAid2hvbGUgY29ydGV4IiksIF0sIGFlcyhsb2coYWdlKSwgbWVhbl9QQzEsIGdyb3VwPW5hbWUsIGNvbG91cj1uYW1lKSkgKwogIGdlb21fcG9pbnQoZGF0YSA9IFBDQV9zdGF0c1tuYW1lICVpbiUgYygiZm9yZWJyYWluIiwgIm1pZGJyYWluIiwgImhpbmRicmFpbiIsICJuZXVyYWwgdHViZSIsICJ3aG9sZSBjb3J0ZXgiKSwgXSwgYWVzKGxvZyhhZ2UpLCBtZWFuX1BDMSwgZ3JvdXA9bmFtZSwgY29sb3VyPW5hbWUpKSArCiAgeGxhYigibG9nKERQQykiKSArCiAgeWxhYigiTWVhbiBQQzEiKSArCiAgeGxpbShjKGxvZygxMCksIGxvZyg1MCkpKSArCiAgdGhlbWUobGVnZW5kLnBvc2l0aW9uPSJ0b3AiKSArCiAgc2NhbGVfY29sb3JfZGlzY3JldGUobmFtZT0iTmV1cm9uYWwgdGlzc3VlIiwgbGFiZWxzPWMoIkZvcmVicmFpbiIsICJIaW5kYnJhaW4iLCAiTWlkYnJhaW4iLCAiTmV1cmFsIHR1YmUiLCAiV2hvbGUgY29ydGV4IikpCiAgCgpGaWc0LkMKYGBgCgoKCmBgYHtyfQoKUENBX3N0YXRzX1BDMjwtIFBDQVsgbmFtZT09ImhlYXJ0IiAsIC4obWVhbl9QQzI9bWVhbihQQzIpKSAsIGJ5PWMoIm5hbWUiLCAiYWdlIildCgoKYGBgCgoKCgoKYGBge3J9ClBDMV9jbHVzdGVyIDwtIFBDQVsgLCAuKFBDMT1tZWFuKFBDMSkpICAsIGJ5PWNsdXN0ZXJdCgp0aXNzdWVfY2x1c3Rlcl9QQ0EgPC0gZmFjdG9yKFBDMV9jbHVzdGVyW29yZGVyKFBDMSldJGNsdXN0ZXIsIGxldmVscyA9IFBDMV9jbHVzdGVyW29yZGVyKFBDMSldJGNsdXN0ZXIpCgoKbXlfY29sIDwtIGdldF9jb2xvcnModGlzc3VlX2NsdXN0ZXJfUENBLCBncm91cC5jb2w9Y29sb3JSYW1wUGFsZXR0ZShjb2xvcnMgPSBjKCJibGFjayIsICAicHVycGxlIiwgImJsdWUiLCAic2VhZ3JlZW4iLCAiZ29sZCIsICJyZWQiKSkoMTQpKQoKCmBgYAoKCmBgYHtyLCBmaWcuaGVpZ2h0PTYsIGZpZy53aWR0aD0xMH0KClBDQSRjbHVzdGVyIDwtIGZhY3RvcihQQ0EkY2x1c3RlciwgbGV2ZWxzID0gUEMxX2NsdXN0ZXJbb3JkZXIoUEMxKV0kY2x1c3RlcikKCgpTdXBfUENBLkEgPC0gZ2dwbG90KFBDQSkgKwogIGdlb21fdGV4dChhZXMoUEMxLCBQQzIsIGxhYmVsPWNsdXN0ZXIsIGNvbG91cj1jbHVzdGVyKSApICsKICBzY2FsZV9jb2xvcl9tYW51YWwodmFsdWVzPW15X2NvbCkgKyAKICB0aGVtZShsZWdlbmQucG9zaXRpb24gPSAibm9uZSIpCgpTdXBfUENBLkIgPC0gIGdncGxvdChQQ0EpICsKICBnZW9tX3RleHQoYWVzKFBDMSwgUEMzLCBsYWJlbD1jbHVzdGVyLCBjb2xvdXI9Y2x1c3RlcikgKSArCiAgc2NhbGVfY29sb3JfbWFudWFsKHZhbHVlcz1teV9jb2wpICsgCiAgdGhlbWUobGVnZW5kLnBvc2l0aW9uID0gIm5vbmUiKQoKU3VwX1BDQS5DIDwtICBnZ3Bsb3QoUENBKSArCiAgZ2VvbV90ZXh0KGFlcyhQQzIsIFBDMywgbGFiZWw9Y2x1c3RlciwgY29sb3VyPWNsdXN0ZXIpICkgKwogIHNjYWxlX2NvbG9yX21hbnVhbCh2YWx1ZXM9bXlfY29sKSArIAogIHRoZW1lKGxlZ2VuZC5wb3NpdGlvbiA9ICJub25lIikKYGBgCgoKCgpgYGB7cn0KCmxpYnJhcnkocmxhbmcpCgpQQ0FfcGxvdCA8LSBkdXBsaWNhdGUoUENBKQoKClBDQV9wbG90W25hbWU9PSJoZWFydCIsIGdyb3VwOj0iSGVhcnQiXQpQQ0FfcGxvdFtuYW1lPT0ic2tlbGV0YWwgbXVzY2xlIHRpc3N1ZSIsIGdyb3VwOj0iU0tNIl0KUENBX3Bsb3RbbmFtZT09ImFkcmVuYWwgZ2xhbmQiLCBncm91cDo9IkFkcmVuYWwgR2xhbmQiXQpQQ0FfcGxvdFtuYW1lPT0id2hvbGUgY29ydGV4IiwgZ3JvdXA6PSJDb3J0ZXgiXQpQQ0FfcGxvdFtuYW1lICVpbiUgYygiZm9yZWJyYWluIiwgIm1pZGJyYWluIiwgImhpbmRicmFpbiIsICJuZXVyYWwgdHViZSIpICwgZ3JvdXA6PSJOZXVyYWwiXQoKUENBX3Bsb3QkYWdlIDwtIGFzLm51bWVyaWMoYXMuY2hhcmFjdGVyKFBDQV9wbG90JGFnZSApKQoKClBDQV9wbG90WyFuYW1lICVpbiUgYygiZm9yZWJyYWluIiwgIm1pZGJyYWluIiwgImhpbmRicmFpbiIsICJuZXVyYWwgdHViZSIsICJ3aG9sZSBjb3J0ZXgiLCAiaGVhcnQiLCJza2VsZXRhbCBtdXNjbGUgdGlzc3VlIiwgImFkcmVuYWwgZ2xhbmQiKSwgYDo9YChncm91cD0iT3RoZXIiLCBhZ2U9TkEgKV0KCmdncGxvdChQQ0FfcGxvdFtuYW1lIT0id2hvbGUgY29ydGV4IixdKSArCiAgZ2VvbV9wb2ludChhZXMoUEMxLCBQQzIsIGNvbG91cj1hZ2UsIHNoYXBlPWdyb3VwICkgKSArCiAgZ2VvbV9wb2ludChhZXMoUEMxLCBQQzIsIGNvbG91cj1hZ2UsIHNoYXBlPWdyb3VwICkgKSArCiAgc2NhbGVfY29sb3JfZ3JhZGllbnQobG93PSJibHVlIiwgIGhpZ2g9InJlZCIpCgoKCmBgYAoKYGBge3J9CgpsaWJyYXJ5KHN0cmluZ3IpCgpTdXBfUENBX2FnZS5BIDwtIGdncGxvdCgpICsKICBnZW9tX3BvaW50KGRhdGE9UENBX3Bsb3RbZ3JvdXA9PSJPdGhlciJdLCBhZXMoUEMxLCBQQzIsIGNvbG91cj1hZ2UgKSwgYWxwaGE9MC41KSArCiAgZ2VvbV90ZXh0KGRhdGE9UENBX3Bsb3RbZ3JvdXAhPSJPdGhlciIgJiBuYW1lIT0id2hvbGUgY29ydGV4Il0sIGFlcyhQQzEsIFBDMiwgY29sb3VyPWFnZSwgbGFiZWw9c3RyX3N1Yihncm91cCwgMSwxKSApLCBzaXplPTMuNSwgYWxwaGE9MC41ICkgKwogIHNjYWxlX2NvbG9yX2dyYWRpZW50KGxvdz0iYmx1ZSIsICBoaWdoPSJyZWQiKSArCiAgbGFicyhjb2xvdXI9IkVtYnJ5b25pYyBzdGFnZSAoRFBDKSIpCmBgYAoKCgoKYGBge3J9CmdncGxvdCgpICsKICBnZW9tX3BvaW50KGRhdGE9UENBX3Bsb3RbZ3JvdXA9PSJPdGhlciJdLCBhZXMoUEMxLCBQQzIsIGNvbG91cj1sb2coYWdlKSApLCBhbHBoYT0wLjgpICsKICBnZW9tX3RleHQoZGF0YT1QQ0FfcGxvdFtncm91cCE9Ik90aGVyIl0sIGFlcyhQQzEsIFBDMiwgY29sb3VyPWxvZyhhZ2UpLCBsYWJlbD1zdHJfc3ViKGdyb3VwLCAxLDEpICksIHNpemU9My41LCBhbHBoYT0wLjggKSArCiAgc2NhbGVfY29sb3JfZ3JhZGllbnQyKGxvdz0iYmx1ZSIsIG1pZD0iZ29sZCIsICBoaWdoPSJyZWQiLCBtaWRwb2ludCA9IDMuNykKYGBgCgoKCmBgYHtyLCBmaWcud2lkdGg9MTUsIGZpZy5oZWlnaHQ9NX0KcGxvdF9ncmlkKFN1cF9QQ0FfYWdlLkEsIFN1cF9QQ0FfYWdlLkIsIGxhYmVscyA9ICJBVVRPIikKYGBgCgoKCmBgYHtyfQoKClBDQV9iYXRjaCA8LSBtZXJnZShQQ0EsIFBDQVssIC5OLCBieT1jKCJkYXRlIildWywgLihkYXRlLCBiYXRjaD1mcmFuayhkYXRlKSApXSwgYnk9ImRhdGUiKQoKbXlfY29sX2RhdGUgPC0gZ2V0X2NvbG9ycyh0aXNzdWVfY2x1c3Rlcl9QQ0EsIGdyb3VwLmNvbD1jb2xvclJhbXBQYWxldHRlKGNvbG9ycyA9IGMoImJsYWNrIiwgICJwdXJwbGUiLCAiYmx1ZSIsICJzZWFncmVlbiIsICJnb2xkIiwgInJlZCIpKSgxMCkpCgoKCgoKU3VwX1BDQS5EIDwtICBnZ3Bsb3QoUENBX3Bsb3QpICsKICBnZW9tX3RleHQoYWVzKFBDMSwgUEMyLCBsYWJlbD1zdHJfc3ViKGdyb3VwLCAxLDEpICwgY29sb3VyPWZhY3RvcihjbHVzdGVyKSApICkgKwogIHNjYWxlX2NvbG9yX21hbnVhbCh2YWx1ZXM9bXlfY29sKSArIAogIHRoZW1lKGxlZ2VuZC5wb3NpdGlvbiA9ICJub25lIikKCgpTdXBfUENBLkUgPC0gIGdncGxvdChQQ0FfcGxvdCkgKwogIGdlb21fdGV4dChhZXMoUEMxLCBQQzMsIGxhYmVsPXN0cl9zdWIoZ3JvdXAsIDEsMSkgLCBjb2xvdXI9ZmFjdG9yKGNsdXN0ZXIpICkgKSArCiAgc2NhbGVfY29sb3JfbWFudWFsKHZhbHVlcz1teV9jb2wpICsgCiAgdGhlbWUobGVnZW5kLnBvc2l0aW9uID0gIm5vbmUiKQoKU3VwX1BDQS5GIDwtICBnZ3Bsb3QoUENBX3Bsb3QpICsKICBnZW9tX3RleHQoYWVzKFBDMiwgUEMzLCBsYWJlbD1zdHJfc3ViKGdyb3VwLCAxLDEpICwgY29sb3VyPWZhY3RvcihjbHVzdGVyKSApICkgKwogIHNjYWxlX2NvbG9yX21hbnVhbCh2YWx1ZXM9bXlfY29sKSArIAogIHRoZW1lKGxlZ2VuZC5wb3NpdGlvbiA9ICJub25lIikKCgoKClN1cF9QQ0EuRyA8LSAgZ2dwbG90KFBDQV9iYXRjaCkgKwogIGdlb21fdGV4dChhZXMoUEMxLCBQQzIsIGxhYmVsPWNsdXN0ZXIsIGNvbG91cj1mYWN0b3IoYmF0Y2gpICkgKSArCiAgc2NhbGVfY29sb3JfbWFudWFsKHZhbHVlcz1teV9jb2xfZGF0ZSkgKyAKICB0aGVtZShsZWdlbmQucG9zaXRpb24gPSAibm9uZSIpCgoKU3VwX1BDQS5IIDwtICBnZ3Bsb3QoUENBX2JhdGNoKSArCiAgZ2VvbV90ZXh0KGFlcyhQQzEsIFBDMywgbGFiZWw9Y2x1c3RlciwgY29sb3VyPWZhY3RvcihiYXRjaCkgKSApICsKICBzY2FsZV9jb2xvcl9tYW51YWwodmFsdWVzPW15X2NvbF9kYXRlKSArIAogIHRoZW1lKGxlZ2VuZC5wb3NpdGlvbiA9ICJub25lIikKCgpTdXBfUENBLkkgPC0gIGdncGxvdChQQ0FfYmF0Y2gpICsKICBnZW9tX3RleHQoYWVzKFBDMiwgUEMzLCBsYWJlbD1jbHVzdGVyLCBjb2xvdXI9ZmFjdG9yKGJhdGNoKSApICkgKwogIHNjYWxlX2NvbG9yX21hbnVhbCh2YWx1ZXM9bXlfY29sX2RhdGUpICsgCiAgdGhlbWUobGVnZW5kLnBvc2l0aW9uID0gIm5vbmUiKQoKCmBgYAoKCgpgYGB7ciwgZmlnLmhlaWdodD05LCBmaWcud2lkdGg9MTV9CnBsb3RfZ3JpZChTdXBfUENBLkEsIFN1cF9QQ0EuQiwgU3VwX1BDQS5DLCBTdXBfUENBLkQsIFN1cF9QQ0EuRSwgU3VwX1BDQS5GLCBTdXBfUENBLkcsIFN1cF9QQ0EuSCwgU3VwX1BDQS5JLCBsYWJlbHM9IkFVVE8iICkKYGBgCgoKCgoKCgpgYGB7cn0KI1Rpc3N1ZV9jbHVzdGVycyA8LSBjdXRyZWUoaGNfY29scywgayA9IDE4KQojVGlzc3VlX2NsdXN0ZXJzIDwtIGNiaW5kKFRpc3N1ZV9jbHVzdGVycywgVGlzc3Vlc19uYW1lW25hbWVzKFRpc3N1ZV9jbHVzdGVycyldLCBUaXNzdWVzX2FnZVtuYW1lcyhUaXNzdWVfY2x1c3RlcnMpXSwgVGlzc3VlX2RhdGVbbmFtZXMoVGlzc3VlX2NsdXN0ZXJzKV0pIAoKI2NvbG5hbWVzKFRpc3N1ZV9jbHVzdGVycykgPC0gYygiY2x1c3RlciIsICJuYW1lIiwgImFnZSIsICJkYXRlIikKCiNUaXNzdWVfY2x1c3RlcnMgPC0gZGF0YS5mcmFtZShUaXNzdWVfY2x1c3RlcnMpCgojVGlzc3VlX2NsdXN0ZXJzX3N1bVt3aGljaChncmVwbCgiaGluZGJyYWlufG1pZGJyYWlufGZvcmVicmFpbnxuZXVyYWxcIHR1YmUiLCBUaXNzdWVfY2x1c3RlcnNfc3VtJG5hbWUpKSwgXQoKI1Rpc3N1ZV9jbHVzdGVycyRuYW1lIDwtIGZhY3RvcihUaXNzdWVfY2x1c3RlcnMkbmFtZSwgbGV2ZWw9YyggInNrZWxldGFsIG11c2NsZSB0aXNzdWUiLCAiaGVhcnQiLCAidGh5bXVzIiwgInNwbGVlbiIsICJsaXZlciIsICAiYWRyZW5hbCBnbGFuZCIsICJpbnRlc3RpbmUiLCAic3RvbWFjaCIsICJsdW5nIiwgICJraWRuZXkiLCAiYmxhZGRlciIsICJsaW1iIiwgImVtYnJ5b25pYyBmYWNpYWwgcHJvbWluZW5jZSIsICJmb3JlYnJhaW4iLCAiaGluZGJyYWluIiwgIm1pZGJyYWluIiwgIm5ldXJhbCB0dWJlIiwgIndob2xlIGNvcnRleCIpKQoKCiNUaXNzdWVfY2x1c3RlcnMkYWdlIDwtIGZhY3RvcihUaXNzdWVfY2x1c3RlcnMkYWdlLCBsZXZlbHMgPSBhcy5jaGFyYWN0ZXIoc29ydChhcy5udW1lcmljKGxldmVscyhUaXNzdWVfY2x1c3RlcnMkYWdlKSkpKSkKCgoKCmBgYAoKCgoKYGBge3IsICBmaWcuaGVpZ2h0PTQsIGZpZy53aWR0aD0yMH0KCgojVGlzc3VlX2NsdXN0ZXJzX3N1bSA8LSBUaXNzdWVfY2x1c3RlcnMgJT4lIGdyb3VwX2J5KGNsdXN0ZXIsIG5hbWUsIGFnZSApICU+JSBzdW1tYXJpc2UoY291bnQgPSBuKCkpCgpUaXNzdWVfY2x1c3RlcnNfRFQgPC0gZGF0YS50YWJsZShUaXNzdWVfY2x1c3RlcnMpClRpc3N1ZV9jbHVzdGVyc19zdW0gPC0gVGlzc3VlX2NsdXN0ZXJzX0RUWywgLihjb3VudD0uTikgLCBieT1jKCJjbHVzdGVyIiwgIm5hbWUiLCAiYWdlIiApXQoKVGlzc3VlX2NsdXN0ZXJzX3N1bSRhZ2UgPC0gZmFjdG9yKFRpc3N1ZV9jbHVzdGVyc19zdW0kYWdlLCBsZXZlbHM9IHNvcnQoYXMubnVtZXJpYyhhcy5jaGFyYWN0ZXIobGV2ZWxzKFRpc3N1ZV9jbHVzdGVyc19zdW0kYWdlKSkpKSApCgoKIyMgTkEgIyMjCgpUaXNzdWVfY2x1c3RlcnNfc3VtJGNsdXN0ZXIgPC0gZmFjdG9yKFRpc3N1ZV9jbHVzdGVyc19zdW0kY2x1c3RlciAsIGxldmVscyA9UENBWywgbWVhbihQQzEpLCBieT0iY2x1c3RlciIgXVtvcmRlcihWMSldJGNsdXN0ZXIpCgoKVGlzc3VlX2NsdXN0ZXJzX3N1bSA8LSBtZXJnZShUaXNzdWVfY2x1c3RlcnNfc3VtLCBQQ0FbLCAuKG1QQzE9bWVhbihQQzEpLCBtUEMyPW1lYW4oUEMyKSwgbVBDMz1tZWFuKFBDMykgKSwgYnk9ImNsdXN0ZXIiIF1bb3JkZXIobVBDMSldLCBieT0iY2x1c3RlciIpClRpc3N1ZV9jbHVzdGVyc19zdW1bLCBgOj1gKG1lYW5fUEMxPXJvdW5kKG1QQzEsIDIpLG1lYW5fUEMyPXJvdW5kKG1QQzIsIDIpLCBtZWFuX1BDMz1yb3VuZChtUEMzLCAyKSkgIF0KClN1cC5UaXNzdWVfY2x1c3RlcnMuYi5wcmUgPC0gZ2dwbG90KCBUaXNzdWVfY2x1c3RlcnNfc3VtLCBhZXMoYWdlLCBuYW1lKSApICsKICBnZW9tX3RpbGUoYWVzKGZpbGwgPSBjb3VudCkpICsKICBzY2FsZV9maWxsX2dyYWRpZW50KGxvdz0iZ3JleSIsIGhpZ2g9InJlZCIpKwogIGZhY2V0X2dyaWQoLiB+IGNsdXN0ZXIgKyBtZWFuX1BDMSArIG1lYW5fUEMyICsgbWVhbl9QQzMsIGxhYmVsbGVyID0gbGFiZWxfcGFyc2VkICkgKwogIHRoZW1lX2J3KCkrCiAgbGFicyhmaWxsID0gIlNhbXBsZSBjb3VudCIpICsKICB0aGVtZShheGlzLnRleHQueCA9IGVsZW1lbnRfdGV4dChhbmdsZSA9IDkwLCB2anVzdCA9IDApLAogICAgYXhpcy50aXRsZSA9IGVsZW1lbnRfdGV4dChjb2xvdXIgPSBOQSksCiAgICBzdHJpcC50ZXh0ID0gZWxlbWVudF90ZXh0KGhqdXN0ID0gMC41KSkKCgpTdXAuVGlzc3VlX2NsdXN0ZXJzLmMgPC0gcGxvdF9ncmlkKGZha2VfcGxvdC5sZWdlbmQsIFN1cC5UaXNzdWVfY2x1c3RlcnMuYi5wcmUsIG5jb2w9MSwgcmVsX2hlaWdodHMgPSBjKDAuMSwgMSkgKQpTdXAuVGlzc3VlX2NsdXN0ZXJzLmMKYGBgCgoKCmBgYHtyLCBmaWcuaGVpZ2h0PTQsIGZpZy53aWR0aD0yMH0KCmxpYnJhcnkoZ2dyZXBlbCkKClBDQV9wbG90WywgYDo9YChtUEMxPW1lYW4oUEMxKSwgbVBDMj1tZWFuKFBDMikpLCBieT0iY2x1c3RlciIgXQoKCgoKU3VwLlRpc3N1ZV9jbHVzdGVycy5hIDwtIGdncGxvdChQQ0FfcGxvdCkgKwogIGdlb21fcG9pbnQoYWVzKFBDMSwgUEMyICwgY29sb3VyPWZhY3RvcihjbHVzdGVyKSApLCAgYWxwaGE9MC4zICkgKwogIHN0YXRfZWxsaXBzZShhZXMoUEMxLCBQQzIgLCBjb2xvdXI9ZmFjdG9yKGNsdXN0ZXIpICkpICsKICBnZW9tX3RleHRfcmVwZWwoZGF0YT1QQ0FfcGxvdFssIC4obVBDMT1tZWFuKFBDMSksIG1QQzI9bWVhbihQQzIpKSwgYnk9ImNsdXN0ZXIiIF0sCiAgICAgICAgICAgICAgICAgIGFlcyhtUEMxLCBtUEMyLCBsYWJlbD1jbHVzdGVyKSwgc2l6ZT03LCBwb2ludC5wYWRkaW5nID0gTkEsIGZvbnRmYWNlID0gImJvbGQiICkgKwogIHNjYWxlX2NvbG9yX21hbnVhbCh2YWx1ZXM9bXlfY29sKSArIAogIHRoZW1lKGxlZ2VuZC5wb3NpdGlvbiA9ICJub25lIikgCgpTdXAuVGlzc3VlX2NsdXN0ZXJzLmIgPC0gZ2dwbG90KFBDQV9wbG90KSArCiAgZ2VvbV9wb2ludChhZXMoUEMxLCBQQzMgLCBjb2xvdXI9ZmFjdG9yKGNsdXN0ZXIpICksICBhbHBoYT0wLjMgKSArCiAgc3RhdF9lbGxpcHNlKGFlcyhQQzEsIFBDMyAsIGNvbG91cj1mYWN0b3IoY2x1c3RlcikgKSkgKwogIGdlb21fdGV4dF9yZXBlbChkYXRhPVBDQV9wbG90WywgLihtUEMxPW1lYW4oUEMxKSwgbVBDMj1tZWFuKFBDMykpLCBieT0iY2x1c3RlciIgXSwKICAgICAgICAgICAgICAgICAgYWVzKG1QQzEsIG1QQzIsIGxhYmVsPWNsdXN0ZXIpLCBzaXplPTcsIHBvaW50LnBhZGRpbmcgPSBOQSwgZm9udGZhY2UgPSAiYm9sZCIgKSArCiAgc2NhbGVfY29sb3JfbWFudWFsKHZhbHVlcz1teV9jb2wpICsgCiAgdGhlbWUobGVnZW5kLnBvc2l0aW9uID0gIm5vbmUiKSAKICAKClN1cC5UaXNzdWVfY2x1c3RlcnMuYWIgPC0gcGxvdF9ncmlkKFN1cC5UaXNzdWVfY2x1c3RlcnMuYSwgU3VwLlRpc3N1ZV9jbHVzdGVycy5iLCBsYWJlbHMgPSBjKCJFIiwgIkYiKSkKU3VwLlRpc3N1ZV9jbHVzdGVycy5hYgpgYGAKCgpgYGB7ciwgIGZpZy5oZWlnaHQ9NywgZmlnLndpZHRoPTE5fQpGaWc0LkVGRyA8LSBwbG90X2dyaWQoIFN1cC5UaXNzdWVfY2x1c3RlcnMuYWIsIFN1cC5UaXNzdWVfY2x1c3RlcnMuYywgbmNvbD0xLCBsYWJlbHM9YygiIiwgIkciKSwgcmVsX2hlaWdodHMgPSBjKDAuOCwgMSkgKQpGaWc0LkVGRwpgYGAKCgoKCgoKCmBgYHtyfQpGaWcuNEEgPC0gZ2dwbG90KFBDQV9wbG90KSArCiAgZ2VvbV9wb2ludChhZXMoUEMxLCBQQzIsIGNvbG91cj1sb2coYWdlKSwgc2hhcGU9Z3JvdXAgKSwgc2l6ZT0zICwgYWxwaGE9MC43KSArCiAgc2NhbGVfY29sb3JfZ3JhZGllbnQyKGxvdz0iYmx1ZSIsIG1pZD0ieWVsbG93IiwgIGhpZ2g9InJlZCIsIG1pZHBvaW50ID0gMy43LCBuYW1lPSJsbihBZ2UpIikgKwogIHNjYWxlX3NoYXBlKG5hbWU9IlRpc3N1ZSIpICsKICB0aGVtZShsZWdlbmQucG9zaXRpb249InRvcCIpIApgYGAKCgoKCgoKYGBge3IsIGZpZy5oZWlnaHQ9MTUsIGZpZy53aWR0aD0yMH0KCmxpYnJhcnkoZ2dwbG90aWZ5KQoKRmlnLjRBIDwtIGdncGxvdChQQ0FfcGxvdCkgKwogIGdlb21fcG9pbnQoYWVzKFBDMSwgUEMyLCBjb2xvdXI9bG9nKGFnZSksIHNoYXBlPWdyb3VwICksIHNpemU9MyAsIGFscGhhPTAuNykgKwogIHNjYWxlX2NvbG9yX2dyYWRpZW50Mihsb3c9ImJsdWUiLCBtaWQ9InllbGxvdyIsICBoaWdoPSJyZWQiLCBtaWRwb2ludCA9IDMuNywgbmFtZT0ibG4oQWdlKSIpICsKICBzY2FsZV9zaGFwZShuYW1lPSJUaXNzdWUiKSArCiAgdGhlbWUobGVnZW5kLnBvc2l0aW9uPSJ0b3AiKSAKCgojZ2dwbG90KCkgKwojICBnZW9tX3BvaW50KGRhdGE9UENBX3Bsb3RbZ3JvdXA9PSJPdGhlciJdLCBhZXMoUEMxLCBQQzIsIGNvbG91cj1hZ2UgKSwgYWxwaGE9MC41KSArCiMgIGdlb21fdGV4dChkYXRhPVBDQV9wbG90W2dyb3VwIT0iT3RoZXIiXSwgYWVzKFBDMSwgUEMyLCBjb2xvdXI9YWdlLCBsYWJlbD1zdHJfc3ViKGdyb3VwLCAxLDEpICksIHNpemU9My41LCBhbHBoYT0wLjUgKSArCiMgIHNjYWxlX2NvbG9yX2dyYWRpZW50KGxvdz0iYmx1ZSIsICBoaWdoPSJyZWQiKQoKRmlnLjRCIDwtIGdncGxvdChQQ0FfcGxvdCkgKwogIGdlb21fcG9pbnQoYWVzKFBDMSwgUEMzLCBjb2xvdXI9bG9nKGFnZSksIHNoYXBlPWdyb3VwICksIHNpemU9MyAsIGFscGhhPTAuNykgKwogIHNjYWxlX2NvbG9yX2dyYWRpZW50Mihsb3c9ImJsdWUiLCBtaWQ9InllbGxvdyIsICBoaWdoPSJyZWQiLCBtaWRwb2ludCA9IDMuNywgbmFtZT0ibG4oQWdlKSIpICsKICBzY2FsZV9zaGFwZShuYW1lPSJUaXNzdWUiKSArCiAgdGhlbWUobGVnZW5kLnBvc2l0aW9uPSJ0b3AiKSAKCgpGaWcuNEFCLmxlZ2VuZCA8LSBnZXRfbGVnZW5kKEZpZy40QSArIHRoZW1lKGxlZ2VuZC5qdXN0aWZpY2F0aW9uPSJjZW50ZXIiKSApCgoKRmlnLjRBQi5wcmUgPC0gcGxvdF9ncmlkKEZpZy40QSArIHRoZW1lKGxlZ2VuZC5wb3NpdGlvbj0ibm9uZSIpLCBGaWcuNEIgKyB0aGVtZShsZWdlbmQucG9zaXRpb249Im5vbmUiKSwgbnJvdz0xLCBsYWJlbHMgPSAiQVVUTyIpCkZpZy40QUIgPC0gcGxvdF9ncmlkKEZpZy40QUIubGVnZW5kLCBGaWcuNEFCLnByZSwgcmVsX2hlaWdodHMgPSBjKDAuMiwgMSksIG5jb2w9MSApCgoKCgpGaWc0LkMubGVnZW5kIDwtIGdldF9sZWdlbmQoIEZpZzQuQyArIHRoZW1lKGxlZ2VuZC5qdXN0aWZpY2F0aW9uPSJjZW50ZXIiKSkKRmlnNC5DLmNvbXAgPC0gcGxvdF9ncmlkKCBGaWc0LkMubGVnZW5kICwgRmlnNC5DICsgdGhlbWUobGVnZW5kLnBvc2l0aW9uPSJub25lIiksIHJlbF9oZWlnaHRzID0gYygwLjIsIDEpLCBuY29sPTEgLCBsYWJlbHMgPSBjKCIiLCAiQyIpICkKCkZpZzQudG9wcm93IDwtcGxvdF9ncmlkKEZpZy40QUIsIEZpZzQuQy5jb21wLCByZWxfd2lkdGhzID0gYygxLjcsIDEpKQoKI0ZpZzQudG9wcm93IDwtIHBsb3RfZ3JpZChGaWcuNEEsIEZpZy40QiwgRmlnNC5DLCBsYWJlbHMgPSAiQVVUTyIsICBucm93ID0gMSkKRmlnNCA8LSBwbG90X2dyaWQoRmlnNC50b3Byb3csIGZha2VfcGxvdC5sZWdlbmQsIGFzLmdyb2IodGlzc3VlX2hlYXRtYXApLCBuY29sPTEsIHJlbF9oZWlnaHRzID0gYygxLCAwLjIsIDIuMikgLCBsYWJlbHMgPSBjKCIiLCAiIiwgIkQiKSkKRmlnNAoKIzE5MDAgeCAxMzAwCmBgYAoKCgpgYGB7ciwgZmlnLmhlaWdodD0yMCwgZmlnLndpZHRoPTE1fQpwbG90X2dyaWQoRmlnNCwgRmlnNC5FRkcsIG5jb2w9MSwgcmVsX2hlaWdodHMgPSBjKDIsIDEpKQpgYGAKCgpIZXJlIHdlIHNvcnRlZCBtaWNyb2V4b25zIGJ5IFBDMSBsb2FkaW5ncywgYnV0IHdlIGZsaXBlZCBQQzEgd2UgY29jaWRlciB0aGUgdmFsdWVzIGFzIC0xIFBDQSBsb2FkaW5nIG9uIG91ciBwYXBlcgpgYGB7cn0KCmBgYAoKCgpMb2FkaW5nIHRoZXJob2xkIGlzIGFicygwLjAzNSkKCgpgYGB7ciwgZmlnLmhlaWdodD05LCBmaWcud2lkdGg9N30KCk1FX1Rpc3N1ZXNfY2x1c3RlcnNfUFNJIDwtIG1lcmdlKE1FX2NsdXN0ZXJzX1BTSSwgVGlzc3VlX2NsdXN0ZXJzLCBieS54PSJGSUxFX05BTUUiLCBieS55ID0gIkZpbGUuYWNjZXNzaW9uIikKTUVfVGlzc3Vlc19jbHVzdGVyc19QU0kkY2x1c3RlciA8LSBmYWN0b3IoTUVfVGlzc3Vlc19jbHVzdGVyc19QU0kkY2x1c3RlciwgbGV2ZWxzID0gbGV2ZWxzKFRpc3N1ZV9jbHVzdGVyc19zdW0kY2x1c3RlcikpCk1FX1Rpc3N1ZXNfY2x1c3RlcnNfUFNJIDwtIG1lcmdlKCBNRV9UaXNzdWVzX2NsdXN0ZXJzX1BTSSwgIE1FX2NsdXN0ZXJfbmFtZXNbICwgYygiTUVfY2x1c3RlciIsICJNRV9jbHVzdGVyLm5hbWUiKV0sIGJ5Lng9Ik1FX2NsdXN0ZXJzIiwgYnkueT0iTUVfY2x1c3RlciIpCgpNRV9UaXNzdWVzX2NsdXN0ZXJzX1BTSSRNRV9jbHVzdGVyLm5hbWUgPC0gZmFjdG9yKE1FX1Rpc3N1ZXNfY2x1c3RlcnNfUFNJJE1FX2NsdXN0ZXIubmFtZSwgbGV2ZWxzID0gc29ydChhcy5jaGFyYWN0ZXIodW5pcXVlKE1FX1Rpc3N1ZXNfY2x1c3RlcnNfUFNJJE1FX2NsdXN0ZXIubmFtZSkpKSkKCk1FX1Rpc3N1ZXNfY2x1c3RlcnNfUFNJX3N1YiA8LSBNRV9UaXNzdWVzX2NsdXN0ZXJzX1BTSVtNRV9jbHVzdGVyLm5hbWUgJWluJSAgTUVfY2x1c3Rlcl9uYW1lc1tNRV9jbHVzdGVyLnR5cGUgJWluJSBjKCJOZXVyb25hbCIsICJOZXVyby1NdXNjdWxhciIsICJNdXNjdWxhciIsICAiTm9uLU5ldXJvbmFsIiksIE1FX2NsdXN0ZXIubmFtZV0sIF0KTUVfVGlzc3Vlc19jbHVzdGVyc19QU0lfbm9uX25ldXJvbmFsIDwtIE1FX1Rpc3N1ZXNfY2x1c3RlcnNfUFNJX3N1YltjbHVzdGVyICVpbiUgYygxLCA4LCA2LCA3LCAxNSwgNSkgLCBdCgpNRV9UaXNzdWVzX2NsdXN0ZXJzX1BTSV9ub25fbmV1cm9uYWwkY2x1c3RlciA8LSBtYXB2YWx1ZXMoTUVfVGlzc3Vlc19jbHVzdGVyc19QU0lfbm9uX25ldXJvbmFsJGNsdXN0ZXIsCiAgICAgICAgICBmcm9tID0gYygxLCA4LCA2LCA3LCAxNSwgNSksCiAgICAgICAgICB0byA9IGMoICJDMSIsICJDOCIsICJDNiIsICJDNyIsICJDMTUiLCAiQzUiKSApCgoKTUVfVGlzc3Vlc19jbHVzdGVyc19QU0lfbm9uX25ldXJvbmFsJGNsdXN0ZXIgPC0gZmFjdG9yKE1FX1Rpc3N1ZXNfY2x1c3RlcnNfUFNJX25vbl9uZXVyb25hbCRjbHVzdGVyLCBsZXZlbHM9YyggIkMxIiwgIkM4IiwgIkM2IiwgIkM3IiwgIkMxNSIsICJDNSIpKQoKTUVfVGlzc3Vlc19jbHVzdGVyc19QU0lfbm9uX25ldXJvbmFsX2J5X01FIDwtIE1FX1Rpc3N1ZXNfY2x1c3RlcnNfUFNJX25vbl9uZXVyb25hbFsgLCAuKFBTSV9tZWFuPW1lYW4oUFNJKSksIGJ5PWMoIk1FIiwgImNsdXN0ZXIiLCAiTUVfY2x1c3Rlci5uYW1lIikgXQpNRV9UaXNzdWVzX2NsdXN0ZXJzX1BTSV9ub25fbmV1cm9uYWxfYnlfTUVfY2x1c3RlciA8LSBNRV9UaXNzdWVzX2NsdXN0ZXJzX1BTSV9ub25fbmV1cm9uYWxbICwgLihQU0lfbWVhbj1tZWFuKFBTSSkpLCBieT1jKCAiY2x1c3RlciIsICJNRV9jbHVzdGVyLm5hbWUiKSBdCgoKCiAgCmdncGxvdCggKSArCiAgICAgICAgZ2VvbV9saW5lKGRhdGE9IE1FX1Rpc3N1ZXNfY2x1c3RlcnNfUFNJX25vbl9uZXVyb25hbF9ieV9NRSwgYWVzKGZhY3RvcihjbHVzdGVyKSwgUFNJX21lYW4sIGdyb3VwPU1FKSwgY29sb3VyPSJncmV5IikgKwogICAgICAgIGZhY2V0X2dyaWQoIE1FX2NsdXN0ZXIubmFtZSB+IC4pCiAgCmBgYAoKCmBgYHtyLCBmaWcuaGVpZ2h0PTksIGZpZy53aWR0aD03fQoKTUVfVGlzc3Vlc19jbHVzdGVyc19QU0lfbmV1cm9uYWwgPC0gTUVfVGlzc3Vlc19jbHVzdGVyc19QU0lbbmFtZSAlaW4lIGMoImZvcmVicmFpbiIsICJtaWRicmFpbiIsICJoaW5kYnJhaW4iLCAibmV1cmFsIHR1YmUiLCAid2hvbGUgY29ydGV4IiksIF0KCk1FX1Rpc3N1ZXNfY2x1c3RlcnNfUFNJX25ldXJvbmFsJGFnZS5uYW1lIDwtIG1hcHZhbHVlcyhNRV9UaXNzdWVzX2NsdXN0ZXJzX1BTSV9uZXVyb25hbCRhZ2UsCiAgICAgICAgICBmcm9tID0gYygxMC41LCAxMS41LCAxMi41LCAxMy41LCAxNC41LCAxNS41LCAxNi41LCAyMSwgMjUsIDI3LCAzNiwgNDEsIDEzMSwgNjUxKSwKICAgICAgICAgIHRvID0gYyggIkUxMC41IiwgIkUxMS41IiwgIkUxMi41IiwgIkUxMy41IiwgIkUxNC41IiwgIkUxNS41IiwgIkUxNi41IiwgIlAwIiwgIlA0IiwgIlA3IiwgIlAxNSIsICJQMzAiLCAiUDExMCIsICIyMU1iIikgKQoKCk1FX1Rpc3N1ZXNfY2x1c3RlcnNfUFNJX25ldXJvbmFsIDwtIE1FX1Rpc3N1ZXNfY2x1c3RlcnNfUFNJX25ldXJvbmFsW01FX2NsdXN0ZXIubmFtZSAlaW4lICBNRV9jbHVzdGVyX25hbWVzW01FX2NsdXN0ZXIudHlwZSAlaW4lIGMoIk5ldXJvbmFsIiwgIk5ldXJvLU11c2N1bGFyIiwgIk11c2N1bGFyIiwgIk5vbi1OZXVyb25hbCIpLCBNRV9jbHVzdGVyLm5hbWVdLCBdCk1FX1Rpc3N1ZXNfY2x1c3RlcnNfUFNJX25ldXJvbmFsJGFnZS5uYW1lIDwtIGZhY3RvcihNRV9UaXNzdWVzX2NsdXN0ZXJzX1BTSV9uZXVyb25hbCRhZ2UubmFtZSwgbGV2ZWxzPSBjKCAiRTEwLjUiLCAiRTExLjUiLCAiRTEyLjUiLCAiRTEzLjUiLCAiRTE0LjUiLCAiRTE1LjUiLCAiRTE2LjUiLCAiUDAiLCAiUDQiLCAiUDciLCAiUDE1IiwgIlAzMCIsICJQMTEwIiwgIjIxTWIiKSApCgpNRV9UaXNzdWVzX2NsdXN0ZXJzX1BTSV9uZXVyb25hbF9ieV9NRSA8LSBNRV9UaXNzdWVzX2NsdXN0ZXJzX1BTSV9uZXVyb25hbFsgLCAuKFBTSV9tZWFuPW1lYW4oUFNJKSksIGJ5PWMoIk1FIiwgImFnZS5uYW1lIiwgIk1FX2NsdXN0ZXIubmFtZSIpIF0KCgpNRV9UaXNzdWVzX2NsdXN0ZXJzX1BTSV9uZXVyb25hbF9ieV9NRV9jbHVzdGVyIDwtIE1FX1Rpc3N1ZXNfY2x1c3RlcnNfUFNJX25ldXJvbmFsWyAsIC4oUFNJX21lYW49bWVhbihQU0kpKSwgYnk9YygiYWdlLm5hbWUiLCAiTUVfY2x1c3Rlci5uYW1lIiwgIm5hbWUiKSBdCgoKICAKZ2dwbG90KCApICsKICAgICAgICBnZW9tX2xpbmUoZGF0YT0gTUVfVGlzc3Vlc19jbHVzdGVyc19QU0lfbmV1cm9uYWxfYnlfTUUsIGFlcyhmYWN0b3IoYWdlLm5hbWUpLCBQU0lfbWVhbiwgZ3JvdXA9TUUpLCBjb2xvdXI9ImdyZXkiKSArCiAgICAgICAgZ2VvbV9saW5lKGRhdGEgPSBNRV9UaXNzdWVzX2NsdXN0ZXJzX1BTSV9uZXVyb25hbF9ieV9NRV9jbHVzdGVyLCBhZXMoZmFjdG9yKGFnZS5uYW1lKSwgUFNJX21lYW4sIGdyb3VwPW5hbWUsIGNvbG91cj1uYW1lICksIHNpemU9MS4yICkgKwogICAgICAgIGZhY2V0X2dyaWQoIE1FX2NsdXN0ZXIubmFtZSB+IC4pCgoKCmBgYAoKYGBge3IsIGZpZy5oZWlnaHQ9MTUsIGZpZy53aWR0aD03fQoKVE9UQUwuTUVfbGV2ZWwgPC0gcmJpbmQoCk1FX1Rpc3N1ZXNfY2x1c3RlcnNfUFNJX25vbl9uZXVyb25hbF9ieV9NRVsgLCAuKE1FLCBhZ2UubmFtZT1jbHVzdGVyLCBNRV9jbHVzdGVyLm5hbWUsIFBTSV9tZWFuKSBdLCAKTUVfVGlzc3Vlc19jbHVzdGVyc19QU0lfbmV1cm9uYWxfYnlfTUUgKQoKVE9UQUwuY2x1c3Rlcl9sZXZlbCA8LSAgcmJpbmQoCk1FX1Rpc3N1ZXNfY2x1c3RlcnNfUFNJX25ldXJvbmFsX2J5X01FX2NsdXN0ZXIsCk1FX1Rpc3N1ZXNfY2x1c3RlcnNfUFNJX25vbl9uZXVyb25hbF9ieV9NRV9jbHVzdGVyWyAsIC4oYWdlLm5hbWU9Y2x1c3RlciwgTUVfY2x1c3Rlci5uYW1lLCAgbmFtZT0iTm9uIG5ldXJvbmFsIiwgUFNJX21lYW4pIF0gKQoKClRPVEFMLmNsdXN0ZXJfbGV2ZWwkbmFtZSA8LSBtYXB2YWx1ZXMoVE9UQUwuY2x1c3Rlcl9sZXZlbCRuYW1lLAogICAgICAgICAgZnJvbSA9IGMoImZvcmVicmFpbiIsICJtaWRicmFpbiIsICJoaW5kYnJhaW4iLCAibmV1cmFsIHR1YmUiLCAid2hvbGUgY29ydGV4IiksCiAgICAgICAgICB0byA9IGMoIkZvcmVicmFpbiIsICJNaWRicmFpbiIsICJIaW5kYnJhaW4iLCAiTmV1cmFsIHR1YmUiLCAiV2hvbGUgY29ydGV4IikgKQoKClRPVEFMLmNsdXN0ZXJfbGV2ZWwkbmFtZSA8LSBmYWN0b3IoVE9UQUwuY2x1c3Rlcl9sZXZlbCRuYW1lLCBsZXZlbHM9YygiTm9uIG5ldXJvbmFsIiwgIkZvcmVicmFpbiIsICJNaWRicmFpbiIsICJIaW5kYnJhaW4iLCAiTmV1cmFsIHR1YmUiLCAiV2hvbGUgY29ydGV4IikgKQoKCgpGaWc1LkEgPC0gZ2dwbG90KCApICsKICAgICAgICBnZW9tX2xpbmUoZGF0YT0gVE9UQUwuTUVfbGV2ZWwsIGFlcyhmYWN0b3IoYWdlLm5hbWUpLCBQU0lfbWVhbiwgZ3JvdXA9TUUpLCBjb2xvdXI9ImdyZXkiLCBhbHBoYT0wLjUpICsKICAgICAgICBnZW9tX2xpbmUoZGF0YSA9IFRPVEFMLmNsdXN0ZXJfbGV2ZWwsIGFlcyhmYWN0b3IoYWdlLm5hbWUpLCBQU0lfbWVhbiwgZ3JvdXA9bmFtZSwgY29sb3VyPW5hbWUgKSwgc2l6ZT0xLjIgKSArCiAgICAgICAgZmFjZXRfZ3JpZCggTUVfY2x1c3Rlci5uYW1lIH4gLikgKwogICAgICAgIGxhYnMoY29sb3VyID0gIk1lYW4gUFNJIGJ5IikgKwogICAgICAgIHlsYWIoIlBTSSIpICsKICAgICAgICB4bGFiKCIiKSArCiAgICAgICAgdGhlbWUocGFuZWwuYmFja2dyb3VuZCA9IGVsZW1lbnRfcmVjdChmaWxsID0gJ3doaXRlJywgY29sb3VyID0gJ2JsYWNrJykpICsKICAgICAgICB0aGVtZShheGlzLnRleHQueCA9IGVsZW1lbnRfdGV4dChhbmdsZSA9IDkwKSwgbGVnZW5kLnBvc2l0aW9uID0gInRvcCIsIGxlZ2VuZC5kaXJlY3Rpb24gPSAiaG9yaXpvbnRhbCIpCgpGaWc1LkEKYGBgCgoKYGBge3J9CgoKCmdncGxvdCggKSArCiAgICAgICAgZ2VvbV9saW5lKGRhdGE9IFRPVEFMLk1FX2xldmVsLCBhZXMoZmFjdG9yKGFnZS5uYW1lKSwgUFNJX21lYW4sIGdyb3VwPU1FKSwgY29sb3VyPSJncmV5IiwgYWxwaGE9MC41KSArCiAgICAgICAgZ2VvbV9saW5lKGRhdGEgPSBUT1RBTC5jbHVzdGVyX2xldmVsLCBhZXMoZmFjdG9yKGFnZS5uYW1lKSwgUFNJX21lYW4sIGdyb3VwPW5hbWUsIGNvbG91cj1uYW1lICksIHNpemU9MS4yICkgKwogICAgICAgIGZhY2V0X2dyaWQoIE1FX2NsdXN0ZXIubmFtZSB+IC4pICsKICAgICAgICBsYWJzKGNvbG91ciA9ICJNZWFuIFBTSSBieSIpICsKICAgICAgICB5bGFiKCJQU0kiKSArCiAgICAgICAgeGxhYigiIikgKwogICAgICAgIHRoZW1lKHBhbmVsLmJhY2tncm91bmQgPSBlbGVtZW50X3JlY3QoZmlsbCA9ICd3aGl0ZScsIGNvbG91ciA9ICdibGFjaycpKSArCiAgICAgICAgdGhlbWUoYXhpcy50ZXh0LnggPSBlbGVtZW50X3RleHQoYW5nbGUgPSA5MCksIGxlZ2VuZC5wb3NpdGlvbiA9ICJ0b3AiLCBsZWdlbmQuZGlyZWN0aW9uID0gImhvcml6b250YWwiKQpgYGAKCgojIyMjIFdoaXBwZXQtZGVsdGEKCgoKCgpgYGB7cn0KCgoKZmlsZS5yZW1vdmUoJy4vd2hpcHBldF9kZWx0YS55YW1sJykKCgoKd3JpdGUudGFibGUoICJ3aGlwcGV0X2RlbHRhOiIsIGZpbGU9Ii4vd2hpcHBldF9kZWx0YS55YW1sIiwgYXBwZW5kPVRSVUUsIHF1b3RlID0gRkFMU0UsIHJvdy5uYW1lcyA9IEZBTFNFLCBjb2wubmFtZXMgPSBGQUxTRSkKCgp3Y29udHJvbCA8LSBwYXN0ZShUaXNzdWVfY2x1c3RlcnNbY2x1c3RlciAlaW4lIGMoMSwgOCwgNiksICBdJEZpbGUuYWNjZXNzaW9uLCBjb2xsYXBzZT0iLCIpCgoKZm9yIChpIGluIGMoImZvcmVicmFpbiIsICJtaWRicmFpbiIsICJoaW5kYnJhaW4iLCAibmV1cmFsIHR1YmUiKSl7CiAgCiAgZm9yIChhIGluIGMoMTAuNSwgMTEuNSwgMTIuNSwgMTMuNSwgMTQuNSwgMTUuNSwgMTYuNSwgMjEpKSB7CiAgICAKICAgIGlmIChsZW5ndGgoVGlzc3VlX2NsdXN0ZXJzW25hbWU9PWkgJiBhZ2U9PWEgLCBdJEZpbGUuYWNjZXNzaW9uKT49NCl7CiAgICAKICAgICAgbmFtZSA9IHBhc3RlMCgiICAgICIsIGdzdWIoIiAiLCAiXyIsIHBhc3RlKCJjb250cm9sX3ZzIiwgaSwgcGFzdGUwKGEsICI6IiksIHNlcD0iICIgKSkpCiAgICAgIEEgPSAgcGFzdGUwKCIgICAgICAgIEEgOiAiLCB3Y29udHJvbCApCiAgICAgIEIgPSAgcGFzdGUwKCIgICAgICAgIEIgOiAiLCAgcGFzdGUoVGlzc3VlX2NsdXN0ZXJzW25hbWU9PWkgJiBhZ2U9PWEgLCBdJEZpbGUuYWNjZXNzaW9uLCBjb2xsYXBzZT0iLCIpKQogICAgICAKICAgICAgd3JpdGUudGFibGUobmFtZSwgZmlsZT0iLi93aGlwcGV0X2RlbHRhLnlhbWwiLCBhcHBlbmQ9VFJVRSwgcXVvdGUgPSBGQUxTRSwgcm93Lm5hbWVzID0gRkFMU0UsIGNvbC5uYW1lcyA9IEZBTFNFKQogICAgICB3cml0ZS50YWJsZSggQSwgZmlsZT0iLi93aGlwcGV0X2RlbHRhLnlhbWwiLCBhcHBlbmQ9VFJVRSwgcXVvdGUgPSBGQUxTRSwgcm93Lm5hbWVzID0gRkFMU0UsIGNvbC5uYW1lcyA9IEZBTFNFKQogICAgICB3cml0ZS50YWJsZSggQiwgZmlsZT0iLi93aGlwcGV0X2RlbHRhLnlhbWwiLCBhcHBlbmQ9VFJVRSwgcXVvdGUgPSBGQUxTRSwgcm93Lm5hbWVzID0gRkFMU0UsIGNvbC5uYW1lcyA9IEZBTFNFKQogICAgCiAgICB9CgogIH0KICAKfQoKCnNpZ25hbCA9IGMoImhpbmRicmFpbiIsICJuZXVyYWwgdHViZSIpCgpmb3IgKGEgaW4gYygxMC41LCAxMS41LCAxMi41LCAxMy41LCAxNC41LCAxNS41LCAxNi41LCAyMSkpIHsKICAgIAogIGlmIChsZW5ndGgoVGlzc3VlX2NsdXN0ZXJzW25hbWUgJWluJSBzaWduYWwgJiBhZ2U9PWEgLCBdJEZpbGUuYWNjZXNzaW9uKT49NCl7CiAgICAKICAgIG5hbWUgPSBwYXN0ZTAoIiAgICAiLCBnc3ViKCIgIiwgIl8iLCBwYXN0ZSgiY29udHJvbF92cyIsIHBhc3RlKHNpZ25hbCwgY29sbGFwc2U9Ii0iICksIHBhc3RlMChhLCAiOiIpLCBzZXA9IiAiICkpKQogICAgQSA9ICBwYXN0ZTAoIiAgICAgICAgQSA6ICIsIHdjb250cm9sICkKICAgIEIgPSAgcGFzdGUwKCIgICAgICAgIEIgOiAiLCAgcGFzdGUoVGlzc3VlX2NsdXN0ZXJzW25hbWUgJWluJSBzaWduYWwgJiBhZ2U9PWEgLCBdJEZpbGUuYWNjZXNzaW9uLCBjb2xsYXBzZT0iLCIpKQogICAgICAKICAgIHdyaXRlLnRhYmxlKG5hbWUsIGZpbGU9Ii4vd2hpcHBldF9kZWx0YS55YW1sIiwgYXBwZW5kPVRSVUUsIHF1b3RlID0gRkFMU0UsIHJvdy5uYW1lcyA9IEZBTFNFLCBjb2wubmFtZXMgPSBGQUxTRSkKICAgIHdyaXRlLnRhYmxlKCBBLCBmaWxlPSIuL3doaXBwZXRfZGVsdGEueWFtbCIsIGFwcGVuZD1UUlVFLCBxdW90ZSA9IEZBTFNFLCByb3cubmFtZXMgPSBGQUxTRSwgY29sLm5hbWVzID0gRkFMU0UpCiAgICB3cml0ZS50YWJsZSggQiwgZmlsZT0iLi93aGlwcGV0X2RlbHRhLnlhbWwiLCBhcHBlbmQ9VFJVRSwgcXVvdGUgPSBGQUxTRSwgcm93Lm5hbWVzID0gRkFMU0UsIGNvbC5uYW1lcyA9IEZBTFNFKQoKICB9Cn0KIAoKc2lnbmFsID0gYygiaGluZGJyYWluIiwgIm5ldXJhbCB0dWJlIiwgIm1pZGJyYWluIikKCmZvciAoYSBpbiBjKDEwLjUsIDExLjUsIDEyLjUsIDEzLjUsIDE0LjUsIDE1LjUsIDE2LjUsIDIxKSkgewogICAgCiAgaWYgKGxlbmd0aChUaXNzdWVfY2x1c3RlcnNbbmFtZSAlaW4lIHNpZ25hbCAmIGFnZT09YSAsIF0kRmlsZS5hY2Nlc3Npb24pPj00KXsKICAgIAogICAgbmFtZSA9IHBhc3RlMCgiICAgICIsIGdzdWIoIiAiLCAiXyIsIHBhc3RlKCJjb250cm9sX3ZzIiwgcGFzdGUoc2lnbmFsLCBjb2xsYXBzZT0iLSIgKSwgcGFzdGUwKGEsICI6IiksIHNlcD0iICIgKSkpCiAgICBBID0gIHBhc3RlMCgiICAgICAgICBBIDogIiwgd2NvbnRyb2wgKQogICAgQiA9ICBwYXN0ZTAoIiAgICAgICAgQiA6ICIsICBwYXN0ZShUaXNzdWVfY2x1c3RlcnNbbmFtZSAlaW4lIHNpZ25hbCAmIGFnZT09YSAsIF0kRmlsZS5hY2Nlc3Npb24sIGNvbGxhcHNlPSIsIikpCiAgICAgIAogICAgd3JpdGUudGFibGUobmFtZSwgZmlsZT0iLi93aGlwcGV0X2RlbHRhLnlhbWwiLCBhcHBlbmQ9VFJVRSwgcXVvdGUgPSBGQUxTRSwgcm93Lm5hbWVzID0gRkFMU0UsIGNvbC5uYW1lcyA9IEZBTFNFKQogICAgd3JpdGUudGFibGUoIEEsIGZpbGU9Ii4vd2hpcHBldF9kZWx0YS55YW1sIiwgYXBwZW5kPVRSVUUsIHF1b3RlID0gRkFMU0UsIHJvdy5uYW1lcyA9IEZBTFNFLCBjb2wubmFtZXMgPSBGQUxTRSkKICAgIHdyaXRlLnRhYmxlKCBCLCBmaWxlPSIuL3doaXBwZXRfZGVsdGEueWFtbCIsIGFwcGVuZD1UUlVFLCBxdW90ZSA9IEZBTFNFLCByb3cubmFtZXMgPSBGQUxTRSwgY29sLm5hbWVzID0gRkFMU0UpCgogIH0KfQoKCnNpZ25hbCA9IGMoImhpbmRicmFpbiIsICJuZXVyYWwgdHViZSIsICJtaWRicmFpbiIsImZvcmVicmFpbiIpCgpmb3IgKGEgaW4gYygxMC41LCAxMS41LCAxMi41LCAxMy41LCAxNC41LCAxNS41LCAxNi41LCAyMSkpIHsKICAgIAogIGlmIChsZW5ndGgoVGlzc3VlX2NsdXN0ZXJzW25hbWUgJWluJSBzaWduYWwgJiBhZ2U9PWEgLCBdJEZpbGUuYWNjZXNzaW9uKT49NCl7CiAgICAKICAgIG5hbWUgPSBwYXN0ZTAoIiAgICAiLCBnc3ViKCIgIiwgIl8iLCBwYXN0ZSgiY29udHJvbF92cyIsIHBhc3RlKHNpZ25hbCwgY29sbGFwc2U9Ii0iICksIHBhc3RlMChhLCAiOiIpLCBzZXA9IiAiICkpKQogICAgQSA9ICBwYXN0ZTAoIiAgICAgICAgQSA6ICIsIHdjb250cm9sICkKICAgIEIgPSAgcGFzdGUwKCIgICAgICAgIEIgOiAiLCAgcGFzdGUoVGlzc3VlX2NsdXN0ZXJzW25hbWUgJWluJSBzaWduYWwgJiBhZ2U9PWEgLCBdJEZpbGUuYWNjZXNzaW9uLCBjb2xsYXBzZT0iLCIpKQogICAgICAKICAgIHdyaXRlLnRhYmxlKG5hbWUsIGZpbGU9Ii4vd2hpcHBldF9kZWx0YS55YW1sIiwgYXBwZW5kPVRSVUUsIHF1b3RlID0gRkFMU0UsIHJvdy5uYW1lcyA9IEZBTFNFLCBjb2wubmFtZXMgPSBGQUxTRSkKICAgIHdyaXRlLnRhYmxlKCBBLCBmaWxlPSIuL3doaXBwZXRfZGVsdGEueWFtbCIsIGFwcGVuZD1UUlVFLCBxdW90ZSA9IEZBTFNFLCByb3cubmFtZXMgPSBGQUxTRSwgY29sLm5hbWVzID0gRkFMU0UpCiAgICB3cml0ZS50YWJsZSggQiwgZmlsZT0iLi93aGlwcGV0X2RlbHRhLnlhbWwiLCBhcHBlbmQ9VFJVRSwgcXVvdGUgPSBGQUxTRSwgcm93Lm5hbWVzID0gRkFMU0UsIGNvbC5uYW1lcyA9IEZBTFNFKQoKICB9Cn0KCgoKc2lnbmFsID0gYygiYWRyZW5hbCBnbGFuZCIsICJza2VsZXRhbCBtdXNjbGUgdGlzc3VlIiwgImhlYXJ0IikKCmZvciAodGlzc3VlIGluIHNpZ25hbCkgewogICAgCiAgaWYgKGxlbmd0aChUaXNzdWVfY2x1c3RlcnNbbmFtZT09dGlzc3VlLCBdJEZpbGUuYWNjZXNzaW9uKT49NCl7CiAgICAKICAgIG5hbWUgPSBwYXN0ZTAoIiAgICAiLCBnc3ViKCIgIiwgIl8iLCBwYXN0ZSgiY29udHJvbF92cyIsIHBhc3RlMCh0aXNzdWUsICI6IiksIHNlcD0iICIgKSkpCiAgICBBID0gIHBhc3RlMCgiICAgICAgICBBIDogIiwgd2NvbnRyb2wgKQogICAgQiA9ICBwYXN0ZTAoIiAgICAgICAgQiA6ICIsICBwYXN0ZShUaXNzdWVfY2x1c3RlcnNbbmFtZT09dGlzc3VlLCBdJEZpbGUuYWNjZXNzaW9uLCBjb2xsYXBzZT0iLCIpKQogICAgICAKICAgIHdyaXRlLnRhYmxlKG5hbWUsIGZpbGU9Ii4vd2hpcHBldF9kZWx0YS55YW1sIiwgYXBwZW5kPVRSVUUsIHF1b3RlID0gRkFMU0UsIHJvdy5uYW1lcyA9IEZBTFNFLCBjb2wubmFtZXMgPSBGQUxTRSkKICAgIHdyaXRlLnRhYmxlKCBBLCBmaWxlPSIuL3doaXBwZXRfZGVsdGEueWFtbCIsIGFwcGVuZD1UUlVFLCBxdW90ZSA9IEZBTFNFLCByb3cubmFtZXMgPSBGQUxTRSwgY29sLm5hbWVzID0gRkFMU0UpCiAgICB3cml0ZS50YWJsZSggQiwgZmlsZT0iLi93aGlwcGV0X2RlbHRhLnlhbWwiLCBhcHBlbmQ9VFJVRSwgcXVvdGUgPSBGQUxTRSwgcm93Lm5hbWVzID0gRkFMU0UsIGNvbC5uYW1lcyA9IEZBTFNFKQoKICB9Cn0KCgpuYW1lPSAiICAgIGhpbmRicmFpbl9uZXVyYWxfdHViZV8yMV92c19mb3JlYnJhaW5fbWlkYnJhaW5fMjE6IgpBID0gcGFzdGUwKCIgICAgICAgIEEgOiAiLCAgcGFzdGUoVGlzc3VlX2NsdXN0ZXJzW2FnZT09MjEgJiBuYW1lICVpbiUgYygiaGluZGJyYWluIiwgIm5ldXJhbCB0dWJlIiksIF0kRmlsZS5hY2Nlc3Npb24sIGNvbGxhcHNlPSIsIikpCkIgPSBwYXN0ZTAoIiAgICAgICAgQiA6ICIsICBwYXN0ZShUaXNzdWVfY2x1c3RlcnNbYWdlPT0yMSAmIG5hbWUgJWluJSBjKCJmb3JlYnJhaW4iLCAibWlkYnJhaW4iKSwgXSRGaWxlLmFjY2Vzc2lvbiwgY29sbGFwc2U9IiwiKSkKCndyaXRlLnRhYmxlKG5hbWUsIGZpbGU9Ii4vd2hpcHBldF9kZWx0YS55YW1sIiwgYXBwZW5kPVRSVUUsIHF1b3RlID0gRkFMU0UsIHJvdy5uYW1lcyA9IEZBTFNFLCBjb2wubmFtZXMgPSBGQUxTRSkKd3JpdGUudGFibGUoIEEsIGZpbGU9Ii4vd2hpcHBldF9kZWx0YS55YW1sIiwgYXBwZW5kPVRSVUUsIHF1b3RlID0gRkFMU0UsIHJvdy5uYW1lcyA9IEZBTFNFLCBjb2wubmFtZXMgPSBGQUxTRSkKd3JpdGUudGFibGUoIEIsIGZpbGU9Ii4vd2hpcHBldF9kZWx0YS55YW1sIiwgYXBwZW5kPVRSVUUsIHF1b3RlID0gRkFMU0UsIHJvdy5uYW1lcyA9IEZBTFNFLCBjb2wubmFtZXMgPSBGQUxTRSkKCgoKYGBgCgoKCiMjIyMgV2hpcHBldCBFTkNPREUgIyMjIwoKCgpgYGB7cn0KCkdldF9kZWx0YV90YWJsZSA8LSBmdW5jdGlvbiggc2lnbmFsLCBhZ2VzLCBwYXRoLCBleHRlbnNpb24gKXsKCiAgRGVsdGFfZ3JvdXAgPC0gZGF0YS50YWJsZSgpCiAgCiAgCiAgZm9yIChhIGluIGFnZXMpIHsKICAgICAgCiAgICBpZiAobGVuZ3RoKFRpc3N1ZV9jbHVzdGVyc1tuYW1lICVpbiUgc2lnbmFsICYgYWdlPT1hICwgXSRGaWxlLmFjY2Vzc2lvbik+PTQpewogICAgICAKICAgICAgbmFtZSA9IGdzdWIoIiAiLCAiXyIsIHBhc3RlKCJjb250cm9sX3ZzIiwgcGFzdGUoc2lnbmFsLCBjb2xsYXBzZT0iLSIgKSwgYSwgc2VwPSIgIiApKQogIAogICAgICBkZWx0YSA8LSBmcmVhZCAoIHBhc3RlMChwYXRoLCBuYW1lLCBleHRlbnNpb24pKQogICAgICAKICAgICAgZGVsdGEkYWdlIDwtIGEKICAgICAgCiAgICAgIERlbHRhX2dyb3VwIDwtIHJiaW5kKERlbHRhX2dyb3VwLCBkZWx0YSApCiAgICAgIAogICAgfQogIAogIH0KICByZXR1cm4oRGVsdGFfZ3JvdXApICAgIAp9CgpgYGAKCgpgYGB7cn0KCkRlbHRhX0hOTV93aGlwcGV0IDwtIEdldF9kZWx0YV90YWJsZSggYygiaGluZGJyYWluIiwgIm5ldXJhbCB0dWJlIiwgIm1pZGJyYWluIiksCiAgICAgICAgICAgICAgICAgICAgICAgICAgICAgICAgICAgICAgYygxMC41LCAxMS41LCAxMi41LCAxMy41LCAxNC41LCAxNS41LCAxNi41KSwKICAgICAgICAgICAgICAgICAgICAgICAgICAgICAgICAgICAgICAifi9Hb29nbGVfRHJpdmUvUmVzdWx0cy9NRS9QYXBlci9GaW5hbF9SZXBvcnQvUmVwcy9SZXAxL1doaXBwZXQvIiwKICAgICAgICAgICAgICAgICAgICAgICAgICAgICAgICAgICAgICAiLmRpZmYubWljcm9leG9ucyIpCgoKRGVsdGFfSE5NX01FIDwtIEdldF9kZWx0YV90YWJsZSggYygiaGluZGJyYWluIiwgIm5ldXJhbCB0dWJlIiwgIm1pZGJyYWluIiksCiAgICAgICAgICAgICAgICAgICAgICAgICAgICAgICAgICAgICAgYygxMC41LCAxMS41LCAxMi41LCAxMy41LCAxNC41LCAxNS41LCAxNi41KSwKICAgICAgICAgICAgICAgICAgICAgICAgICAgICAgICAgICAgICAifi9Hb29nbGVfRHJpdmUvUmVzdWx0cy9NRS9QYXBlci9GaW5hbF9SZXBvcnQvUmVwcy9SZXAxL1doaXBwZXQvIiwKICAgICAgICAgICAgICAgICAgICAgICAgICAgICAgICAgICAgICAiLmRpZmYuTUUubWljcm9leG9ucyIpCgoKRGVsdGFfSE5NX21lcmdlIDwtIG1lcmdlKERlbHRhX0hOTV93aGlwcGV0LCBEZWx0YV9ITk1fTUUsIGJ5PWMoImV4b25fSUQiLCAiYWdlIikpCgoKbGVuZ3RoKHVuaXF1ZShEZWx0YV9ITk1fbWVyZ2VbYWJzKERlbHRhUHNpLngpPj0wLjEgJiBQcm9iYWJpbGl0eS54PjAuOSAmIGFicyhEZWx0YVBzaS55KT49MC4xICYgUHJvYmFiaWxpdHkueT4wLjksIGV4b25fSURdKSkKCmBgYAoKCgoKYGBge3J9CgpEZWx0YV9GX3doaXBwZXQgPC0gR2V0X2RlbHRhX3RhYmxlKCBjKCJmb3JlYnJhaW4iKSwKICAgICAgICAgICAgICAgICAgICAgICAgICAgICAgICAgICAgICBjKDEwLjUsIDExLjUsIDEyLjUsIDEzLjUsIDE0LjUsIDE1LjUsIDE2LjUsIDIxKSwKICAgICAgICAgICAgICAgICAgICAgICAgICAgICAgICAgICAgICAifi9Hb29nbGVfRHJpdmUvUmVzdWx0cy9NRS9QYXBlci9GaW5hbF9SZXBvcnQvUmVwcy9SZXAxL1doaXBwZXQvIiwKICAgICAgICAgICAgICAgICAgICAgICAgICAgICAgICAgICAgICAiLmRpZmYubWljcm9leG9ucyIpCgoKRGVsdGFfRl9NRSA8LSBHZXRfZGVsdGFfdGFibGUoIGMoImZvcmVicmFpbiIpLAogICAgICAgICAgICAgICAgICAgICAgICAgICAgICAgICAgICAgIGMoMTAuNSwgMTEuNSwgMTIuNSwgMTMuNSwgMTQuNSwgMTUuNSwgMTYuNSwgMjEpLAogICAgICAgICAgICAgICAgICAgICAgICAgICAgICAgICAgICAgICJ+L0dvb2dsZV9Ecml2ZS9SZXN1bHRzL01FL1BhcGVyL0ZpbmFsX1JlcG9ydC9SZXBzL1JlcDEvV2hpcHBldC8iLAogICAgICAgICAgICAgICAgICAgICAgICAgICAgICAgICAgICAgICIuZGlmZi5NRS5taWNyb2V4b25zIikKCgpEZWx0YV9GX21lcmdlIDwtIG1lcmdlKERlbHRhX0Zfd2hpcHBldCwgRGVsdGFfRl9NRSwgYnk9YygiZXhvbl9JRCIsICJhZ2UiKSkKCmxlbmd0aCh1bmlxdWUoRGVsdGFfRl9tZXJnZVthYnMoRGVsdGFQc2kueCk+PTAuMSAmIFByb2JhYmlsaXR5Lng+MC45ICYgYWJzKERlbHRhUHNpLnkpPj0wLjEgJiBQcm9iYWJpbGl0eS55PjAuOSwgZXhvbl9JRF0pKQpgYGAKCgoKCgoKYGBge3IsIGZpZy5oZWlnaHQ9MTIsIGZpZy53aWR0aD0xNX0KClN1cF92dWxjYW5vLkIgPC0gZ2dwbG90KCApICsKICBnZW9tX3BvaW50KGRhdGE9RGVsdGFfSE5NX01FLCBhZXMoLURlbHRhUHNpLCBQcm9iYWJpbGl0eSkpICsKICBnZW9tX3BvaW50KGRhdGE9RGVsdGFfSE5NX01FWyBEZWx0YVBzaT49MC4xICYgIFByb2JhYmlsaXR5ID49IDAuOSAsIF0sIGFlcygtRGVsdGFQc2ksIFByb2JhYmlsaXR5KSwgY29sb3I9ImJyb3duIikgKwogIGdlb21fcG9pbnQoZGF0YT1EZWx0YV9ITk1fTUVbIERlbHRhUHNpPCgtMC4xKSAmICBQcm9iYWJpbGl0eSA+PSAwLjkgLCBdLCBhZXMoLURlbHRhUHNpLCBQcm9iYWJpbGl0eSksIGNvbG9yPSJkYXJrZ3JlZW4iKSArCiAgZ2VvbV90ZXh0KGRhdGE9RGVsdGFfSE5NX01FWyBEZWx0YVBzaT49MC4xICYgIFByb2JhYmlsaXR5ID49IDAuOSAsIC5OLCBieT0iYWdlIl0sIGFlcygtMC44NSwgMC45LCBsYWJlbD1OKSwgY29sb3VyID0gImJyb3duIiwgZm9udGZhY2UgPSAiYm9sZCIsIHNpemU9NSkgKwogIGdlb21fdGV4dChkYXRhPURlbHRhX0hOTV9NRVsgRGVsdGFQc2k8KC0wLjEpICYgIFByb2JhYmlsaXR5ID49IDAuOSAgLCAuTiwgYnk9ImFnZSJdLCBhZXMoMC44NSwgMC45LCBsYWJlbD1OKSwgY29sb3VyID0gImRhcmtncmVlbiIsIGZvbnRmYWNlID0gImJvbGQiLCBzaXplPTUpICsKICBmYWNldF9ncmlkKC4gfiBhZ2UpICsKICB4bGltKGMoLTEsMSkpICsKICB4bGFiKCJEZWx0YSBQU0kiKSArCiAgdGhlbWVfYncoKQoKClN1cF92dWxjYW5vLkEgPC0gZ2dwbG90KCApICsKICBnZW9tX3BvaW50KGRhdGE9RGVsdGFfSE5NX3doaXBwZXQsIGFlcygtRGVsdGFQc2ksIFByb2JhYmlsaXR5KSkgKwogIGdlb21fcG9pbnQoZGF0YT1EZWx0YV9ITk1fd2hpcHBldFsgRGVsdGFQc2k+PTAuMSAmICBQcm9iYWJpbGl0eSA+PSAwLjkgLCBdLCBhZXMoLURlbHRhUHNpLCBQcm9iYWJpbGl0eSksIGNvbG9yPSJicm93biIpICsKICBnZW9tX3BvaW50KGRhdGE9RGVsdGFfSE5NX3doaXBwZXRbIERlbHRhUHNpPCgtMC4xKSAmICBQcm9iYWJpbGl0eSA+PSAwLjkgLCBdLCBhZXMoLURlbHRhUHNpLCBQcm9iYWJpbGl0eSksIGNvbG9yPSJkYXJrZ3JlZW4iKSArCiAgZ2VvbV90ZXh0KGRhdGE9RGVsdGFfSE5NX3doaXBwZXRbIERlbHRhUHNpPj0wLjEgJiAgUHJvYmFiaWxpdHkgPj0gMC45ICwgLk4sIGJ5PSJhZ2UiXSwgYWVzKC0wLjg1LCAwLjksIGxhYmVsPU4pLCBjb2xvdXIgPSAiYnJvd24iLCBmb250ZmFjZSA9ICJib2xkIiwgc2l6ZT01KSArCiAgZ2VvbV90ZXh0KGRhdGE9RGVsdGFfSE5NX3doaXBwZXRbIERlbHRhUHNpPCgtMC4xKSAmICBQcm9iYWJpbGl0eSA+PSAwLjkgICwgLk4sIGJ5PSJhZ2UiXSwgYWVzKDAuODUsIDAuOSwgbGFiZWw9TiksIGNvbG91ciA9ICJkYXJrZ3JlZW4iLCBmb250ZmFjZSA9ICJib2xkIiwgc2l6ZT01KSArICAKICBmYWNldF9ncmlkKC4gfiBhZ2UpICsKICB4bGltKGMoLTEsMSkpICsKICB4bGFiKCJEZWx0YSBQU0kiKSArCiAgdGhlbWVfYncoKQoKCgoKCmBgYAoKCiMjIyB3aGlwZXQgdnMgTUUKCmBgYHtyfQpNRV9BRF9BQSA8LSBmcmVhZCgiLi4vRmluYWxfUmVwb3J0L1JlcHMvUmVwMS9XaGlwcGV0L1F1YW50L01FX0FEX0FBLnR4dCIpCgpEZWx0YV9ITk1fdG90YWwgPC0gIG1lcmdlKApEZWx0YV9ITk1fd2hpcHBldFsgLCBjKCJleG9uX0lEIiwgIkdlbmUiLCAiTm9kZSIsICJDb29yZCIsICJTdHJhbmQiLCAiVHlwZSIsICJEZWx0YVBzaSIsICJQcm9iYWJpbGl0eSIsICJhZ2UiKV0sCkRlbHRhX0hOTV9NRVsgLCBjKCJleG9uX0lEIiwgIkdlbmUiLCAiTm9kZSIsICJDb29yZCIsICJTdHJhbmQiLCAiVHlwZSIsICJEZWx0YVBzaSIsICJQcm9iYWJpbGl0eSIsICJhZ2UiKV0sCgpieT1jKCJleG9uX0lEIiwgIkdlbmUiLCAiTm9kZSIsICJDb29yZCIsICJTdHJhbmQiLCAiVHlwZSIsICAiYWdlIikpCgpEZWx0YV9ITk1fdG90YWxbICwgY29yKERlbHRhUHNpLngsIERlbHRhUHNpLnksIG1ldGhvZD0icGVhcnNvbiIpICwgYnk9YWdlIF0KCkRlbHRhX0hOTV90b3RhbFtwYXN0ZSggZXhvbl9JRCwgYWdlLCBzZXA9InwiKSAlaW4lIHRvdGFsX2RpZmZfSE5NICwgY29yKERlbHRhUHNpLngsIERlbHRhUHNpLnksIG1ldGhvZD0icGVhcnNvbiIpICwgYnk9YWdlIF0KCgpEZWx0YV9ITk1fdG90YWxbLCBOb2RlX3R5cGU6PXBhc3RlMCgibSIsIFR5cGUpXQpEZWx0YV9ITk1fdG90YWxbIFR5cGU9PSJDRSIgJiBleG9uX0lEICVpbiUgTUVfQURfQUFbIFR5cGU9PSJBQSIgJiBleG9uX3R5cGU9PSJFIiAsICBleG9uX0lEICBdLCBOb2RlX3R5cGU6PSJtQ0VfQUEiIF0KRGVsdGFfSE5NX3RvdGFsWyBUeXBlPT0iQ0UiICYgZXhvbl9JRCAlaW4lIE1FX0FEX0FBWyBUeXBlPT0iQUEiICYgZXhvbl90eXBlPT0iTUUiICwgIGV4b25fSUQgIF0sIE5vZGVfdHlwZTo9Im1DRV9tQUEiIF0KRGVsdGFfSE5NX3RvdGFsWyBUeXBlPT0iQ0UiICYgZXhvbl9JRCAlaW4lIE1FX0FEX0FBWyBUeXBlPT0iQUQiICYgZXhvbl90eXBlPT0iRSIgLCAgZXhvbl9JRCAgXSwgTm9kZV90eXBlOj0ibUNFX0FEIiBdCkRlbHRhX0hOTV90b3RhbFsgVHlwZT09IkNFIiAmIGV4b25fSUQgJWluJSBNRV9BRF9BQVsgVHlwZT09IkFEIiAmIGV4b25fdHlwZT09Ik1FIiAsICBleG9uX0lEICBdLCBOb2RlX3R5cGU6PSJtQ0VfbUFEIiBdCgp3aGlwcGV0X01FX2NvcnIgPC0gbWVyZ2UoIERlbHRhX0hOTV90b3RhbFsgLCBjb3IoRGVsdGFQc2kueCwgRGVsdGFQc2kueSwgbWV0aG9kPSJwZWFyc29uIikgLCBieT1hZ2UgXSwgCiAgICAgICAgICAgICAgICAgICAgICAgICAgRGVsdGFfSE5NX3RvdGFsWyAoYWJzKERlbHRhUHNpLngpPj0wLjEgJiBQcm9iYWJpbGl0eS54Pj0wLjkpIHwoYWJzKERlbHRhUHNpLnkpPj0wLjEgJiBQcm9iYWJpbGl0eS55Pj0wLjkpICAsCiAgICAgICAgICAgICAgICAgICAgICAgICAgICAgICAgICAgICAgICAgICBjb3IoRGVsdGFQc2kueCwgRGVsdGFQc2kueSwgbWV0aG9kPSJwZWFyc29uIikgLCBieT1hZ2UgXSwgCiAgICAgICAgICAgICAgICAgICAgICAgICAgYnk9ImFnZSIpCgojd2hpcHBldF9NRV9jb3JyLnR5cGVzIDwtIG1lcmdlKCBEZWx0YV9ITk1fdG90YWxbICwgLihOPS5OLCBjb3IudG90YWw9KGNvcihEZWx0YVBzaS54LCBEZWx0YVBzaS55LCBtZXRob2Q9InBlYXJzb24iKSkpICwgYnk9YygiYWdlIiwgIk5vZGVfdHlwZSIpIF0sIAojICAgICAgICAgICAgICAgICAgICAgICAgICBEZWx0YV9ITk1fdG90YWxbcGFzdGUoIGV4b25fSUQsIGFnZSwgc2VwPSJ8IikgJWluJSB0b3RhbF9kaWZmX0hOTSAsIC4oTj0uTiwgY29yLnNpZz0oY29yKERlbHRhUHNpLngsIERlbHRhUHNpLnksIG1ldGhvZD0icGVhcnNvbiIpKSkgLCBieT1jKCJhZ2UiLCAjIk5vZGVfdHlwZSIpIF0sCiMgICAgICAgICAgICAgICAgICAgICAgICAgIGJ5PWMoImFnZSIsICJOb2RlX3R5cGUiKSkKCgoKd2hpcHBldF9NRV9jb3JyLnR5cGVzIDwtIERlbHRhX0hOTV90b3RhbFsgLCAuKE49Lk4sIGNvci50b3RhbD0oY29yKERlbHRhUHNpLngsIERlbHRhUHNpLnksIG1ldGhvZD0icGVhcnNvbiIpKSkgLCBieT1jKCJhZ2UiLCAiTm9kZV90eXBlIikgXQoKCiN3aGlwcGV0X01FX2NvcnIudHlwZXNbTi55PDUsIGNvci5zaWc6PU5BXQoKI3doaXBwZXRfTUVfY29yci50eXBlcy5tZWx0ZWQgPC0gcmJpbmQod2hpcHBldF9NRV9jb3JyLnR5cGVzWywgLihhZ2UsIE5vZGVfdHlwZSwgTj1OLngsIGNvcj1jb3IudG90YWwsIEdyb3VwPSJUb3RhbCIpIF0sCiAjICAgICB3aGlwcGV0X01FX2NvcnIudHlwZXNbLCAuKGFnZSwgTm9kZV90eXBlLCBOPU4ueSwgY29yPWNvci5zaWcsIEdyb3VwPSJTaWduaWZpY2FudCIpIF0pCgpEZWx0YV9ITk1fdG90YWwkTm9kZV90eXBlIDwtIGZhY3RvcihEZWx0YV9ITk1fdG90YWwkTm9kZV90eXBlICwgbGV2ZWxzID0gYygibUNFIiwgIm1BQSIsICJtQUQiLCAibUNFX21BQSIsICJtQ0VfbUFEIiwgIm1DRV9BQSIsICJtQ0VfQUQiKSkKCndoaXBwZXRfTUVfY29yci50eXBlcyROb2RlX3R5cGUgPC0gZmFjdG9yKHdoaXBwZXRfTUVfY29yci50eXBlcyROb2RlX3R5cGUgLCBsZXZlbHMgPSBjKCJtQ0UiLCAibUFBIiwgIm1BRCIsICJtQ0VfbUFBIiwgIm1DRV9tQUQiLCAibUNFX0FBIiwgIm1DRV9BRCIpKQoKClN1cF92dWxjYW5vLkQgPC0gZ2dwbG90KHdoaXBwZXRfTUVfY29yci50eXBlc1tOPjUsIF0pICsKICBnZW9tX2JveHBsb3QoYWVzKE5vZGVfdHlwZSwgY29yLnRvdGFsLCBmaWxsPU5vZGVfdHlwZSkpICsKICBzY2FsZV9maWxsX2Rpc2NyZXRlKG5hbWUgPSAiTWljcm9leG9uIG5vZGUgdHlwZSIpICsKICB4bGFiKCIiKSArCiAgeWxhYigiRGVsdGEgUFNJIGNvcnJlbGF0aW9uIikgKwogIHRoZW1lX2J3KCkgKwogIHRoZW1lKGxlZ2VuZC5wb3NpdGlvbiA9ICJ0b3AiKSArCiAgdGhlbWUoYXhpcy50ZXh0LnggPSBlbGVtZW50X3RleHQoYW5nbGUgPSA0NSwgdmp1c3QgPSAwLjYpKSArCiAgICAgZ3VpZGVzKGZpbGwgPSBndWlkZV9sZWdlbmQobnJvdyA9IDEpKQoKCmBgYAoKCmBgYHtyfQoKU3VwX3Z1bGNhbm8uQyA8LSBnZ3Bsb3QoRGVsdGFfSE5NX3RvdGFsWyFpcy5uYShOb2RlX3R5cGUpXSApICsKICBnZW9tX2JhcihhZXMoYXMuZmFjdG9yKGFnZSksIGZpbGw9Tm9kZV90eXBlKSwgc3RhdD0iY291bnQiICkgKwogIGdlb21fdGV4dCggZGF0YT1EZWx0YV9ITk1fdG90YWxbICwgLihOPS5OLCBjb3I9Y29yKERlbHRhUHNpLngsIERlbHRhUHNpLnksIG1ldGhvZD0icGVhcnNvbiIpKSAsIGJ5PWFnZSBdLAogICAgICAgICAgICAgYWVzKHg9YXMuZmFjdG9yKGFnZSksIHk9TisyMDAsIGxhYmVsPSBwYXN0ZTAoIlI9Iiwgcm91bmQoY29yLCAyKSkpKSArCiAgeGxhYigiRFBDIikgKwogIHlsYWIoIk51bWJlciBvZiBzcGxpY2luZyBub2RlcyIpICsKICAgIHNjYWxlX2ZpbGxfZGlzY3JldGUobmFtZSA9ICJNaWNyb2V4b24gbm9kZSB0eXBlIikgKwogIHRoZW1lX2J3KCkgKwogIHRoZW1lKGxlZ2VuZC5wb3NpdGlvbiA9ICJ0b3AiKSArCiAgZ3VpZGVzKGZpbGwgPSBndWlkZV9sZWdlbmQobnJvdyA9IDEpKQoKU3VwX3Z1bGNhbm8ubGVnZW5kIDwtIGdldF9sZWdlbmQoU3VwX3Z1bGNhbm8uQykKYGBgCgoKCgoKCmBgYHtyfQoKbGlicmFyeShnZ0V4dHJhKQoKTUVfQURfQUEKCkRlbHRhX0hOTV90b3RhbC5hbHRfTUUgPC0gbWVyZ2UoRGVsdGFfSE5NX3RvdGFsWyAsIC4oZXhvbl9JRCwgTm9kZV90eXBlLCBhZ2UsIAogICAgICAgICAgICAgICAgICAgICAgICAgICAgICAgICAgICAgICAgICAgICAgICAgICAgIERlbHRhUHNpLndwPURlbHRhUHNpLngsIFByb2JhYmlsaXR5LndwPVByb2JhYmlsaXR5LngsIAogICAgICAgICAgICAgICAgICAgICAgICAgICAgICAgICAgICAgICAgICAgICAgICAgICAgIERlbHRhUHNpLm1lPURlbHRhUHNpLnksIFByb2JhYmlsaXR5Lm1lPVByb2JhYmlsaXR5LnkpXSwKICAgICAgICAgICAgICAgICAgICAgICAgICAgICAgICBNRV9BRF9BQVsgLGMoImV4b25fSUQiLCAiZXhvbl9JRC5hbHQiKV0sIGJ5PWMoImV4b25fSUQiKSkKCkRlbHRhX0hOTV90b3RhbC5hbHRfTUUuY29tcGFyZSA8LSBtZXJnZShEZWx0YV9ITk1fdG90YWwuYWx0X01FLAogICAgICAgICAgICAgICAgICAgICAgICAgICAgICAgICAgICAgICAgRGVsdGFfSE5NX3RvdGFsWyAsIC4oZXhvbl9JRCwgTm9kZV90eXBlLCBhZ2UsIAogICAgICAgICAgICAgICAgICAgICAgICAgICAgICAgICAgICAgICAgICAgICAgICAgICAgICAgICAgICAgRGVsdGFQc2kud3AuYWx0PURlbHRhUHNpLngsIFByb2JhYmlsaXR5LndwLmFsdD1Qcm9iYWJpbGl0eS54LCAKICAgICAgICAgICAgICAgICAgICAgICAgICAgICAgICAgICAgICAgICAgICAgICAgICAgICAgICAgICAgIERlbHRhUHNpLm1lLmFsdD1EZWx0YVBzaS55LCBQcm9iYWJpbGl0eS5tZS5hbHQ9UHJvYmFiaWxpdHkueSldLAogICAgICAgICAgICAgICAgICAgICAgICAgICAgICAgICAgICAgICAgYnkueD1jKCJleG9uX0lELmFsdCIsICJhZ2UiKSwgYnkueT1jKCJleG9uX0lEIiwgImFnZSIpICkKCgoKRGVsdGFfSE5NX3RvdGFsLmFsdF9NRS5jb21wYXJlLnN0YXRzIDwtIERlbHRhX0hOTV90b3RhbC5hbHRfTUUuY29tcGFyZVssIC4oIGFsdF9jb3Iud3A9Y29yKERlbHRhUHNpLndwLCBEZWx0YVBzaS53cC5hbHQpLCBhbHRfY29yLm1lPWNvcihEZWx0YVBzaS5tZSwgRGVsdGFQc2kubWUuYWx0KSApLCBieT1jKCJleG9uX0lELmFsdCIsICJOb2RlX3R5cGUueCIpIF0KCgpwIDwtIGdncGxvdChEZWx0YV9ITk1fdG90YWwuYWx0X01FLmNvbXBhcmUuc3RhdHMpKwogIGdlb21fcG9pbnQoYWVzKHg9YWx0X2Nvci53cCwgeT1hbHRfY29yLm1lKSwgYWxwaGE9MC40KSsKICB0aGVtZV9idygpICsKICBnZW9tX2RlbnNpdHlfMmQoYWVzKHg9YWx0X2Nvci53cCwgeT1hbHRfY29yLm1lKSwgYWxwaGE9MC40LCBuPTUwMCwgYWRqdXN0PTEuMykgKwogIGdlb21fcnVnKCkgKwogIHhsYWIoIldoaXBwZXQgc2hvcnQvbG9uZyBtaWNyb2V4b24gY29ycmVsYXRpb24iKSArCiAgeWxhYigiTWljcm9FeG9uYXRvciBzaG9ydC9sb25nIG1pY3JvZXhvbiBjb3JyZWxhdGlvbiIpCgoKU3VwX3Z1bGNhbm8uRSA8LSBnZ01hcmdpbmFsKHAsIHR5cGUgPSAiaGlzdG9ncmFtIiwgZmlsbD0idHJhbnNwYXJlbnQiKQoKCmBgYAoKCgoKYGBge3IsICBmaWcuaGVpZ2h0PTE1LCBmaWcud2lkdGg9MTJ9CgoKU3VwX3Z1bGNhbm8uQ0QgPC0gcGxvdF9ncmlkKFN1cF92dWxjYW5vLkMgICsgdGhlbWUobGVnZW5kLnBvc2l0aW9uID0gIk5BIiksCiAgICAgICAgICAgICAgICAgICAgICAgICAgICAgICAgU3VwX3Z1bGNhbm8uRCAgKyB0aGVtZShsZWdlbmQucG9zaXRpb24gPSAiTkEiKSwKICAgICAgICAgICAgICAgICAgICAgICAgICAgICAgICBucm93PTEsIGxhYmVscyA9IGMoIkMiLCAiRCIpICkKCgpTdXBfdnVsY2Fuby5DRCA8LSBwbG90X2dyaWQoU3VwX3Z1bGNhbm8ubGVnZW5kLCBTdXBfdnVsY2Fuby5DRCwgbmNvbD0xLCByZWxfaGVpZ2h0cyA9IGMoMSwgOCkgKQoKClN1cF92dWxjYW5vLmJvdHRvbSA8LXBsb3RfZ3JpZChTdXBfdnVsY2Fuby5DRCwgcGxvdF9ncmlkKG51bGxHcm9iKCksIFN1cF92dWxjYW5vLkUsIG51bGxHcm9iKCksICBucm93PTEsIHJlbF93aWR0aHMgPSBjKDEsMywxKSwgbGFiZWxzID0gYygiIiwgIkUiLCAiIikgKSwgbmNvbD0xKQoKU3VwX3Z1bGNhbm8uYm90dG9tIDwtcGxvdF9ncmlkKFN1cF92dWxjYW5vLkNELCBTdXBfdnVsY2Fuby5FLCBsYWJlbHMgPSBjKCAiIiwgIkUiKSwgbnJvdz0xLCByZWxfd2lkdGhzID0gYygyLCAxKSkKCgoKCnBsb3RfZ3JpZChTdXBfdnVsY2Fuby5BLCBTdXBfdnVsY2Fuby5CLCBTdXBfdnVsY2Fuby5ib3R0b20sICBuY29sPTEsIGxhYmVscyA9IGMoIkEiLCAiQiIsICIiKSwgcmVsX2hlaWdodHMgPSBjKDEsMSwzKSkKYGBgCgoKYGBge3IsIGZpZy5oZWlnaHQ9OCwgZmlnLndpZHRoPTEwfQoKbGlicmFyeShncmlkRXh0cmEpCmxpYnJhcnkoZ3JpZCkKCnBsb3RfZ3JpZChTdXBfdnVsY2Fuby5DRCwgcGxvdF9ncmlkKG51bGxHcm9iKCksIFN1cF92dWxjYW5vLkUsIG51bGxHcm9iKCksICBucm93PTEsIHJlbF93aWR0aHMgPSBjKDEsMywxKSwgbGFiZWxzID0gYygiIiwgIkUiLCAiIikgKSwgbmNvbD0xKQpgYGAKCgojIyMKCgoKCmBgYHtyfQpEZWx0YV9ITk1fd2hpcHBldF9NRSA8LSBtZXJnZShEZWx0YV9ITk1fd2hpcHBldCwgRGVsdGFfSE5NX01FLCBieT1jKCJleG9uX0lEIiwgIkdlbmUiLCAiTm9kZSIsICAiQ29vcmQiLCAiU3RyYW5kIiwgIlR5cGUiLCAiYWdlIikgKQoKRGVsdGFfSE5NX3doaXBwZXRfTUVbIChhYnMoRGVsdGFQc2kueCk+PTAuMSAmICBQcm9iYWJpbGl0eS54ID49IDAuOSkgJiAgKGFicyhEZWx0YVBzaS55KT49MC4xICYgIFByb2JhYmlsaXR5LnkgPj0gMC45KSAsIC5OICwgYnk9KGFnZSldCgpEZWx0YV9ITk1fd2hpcHBldF9NRV9pbmNsdWRlZCA8LSBEZWx0YV9ITk1fd2hpcHBldF9NRVsgKERlbHRhUHNpLng8PS0wLjEgJiAgUHJvYmFiaWxpdHkueCA+PSAwLjkpICYgIChEZWx0YVBzaS55PD0tMC4xICYgIFByb2JhYmlsaXR5LnkgPj0gMC45KSAgLCAuTiAsIGJ5PShhZ2UpXQpEZWx0YV9ITk1fd2hpcHBldF9NRV9leGNsdWRlZCA8LSBEZWx0YV9ITk1fd2hpcHBldF9NRVsgKERlbHRhUHNpLng+PTAuMSAmICBQcm9iYWJpbGl0eS54ID49IDAuOSkgJiAgKERlbHRhUHNpLnk+PTAuMSAmICBQcm9iYWJpbGl0eS55ID49IDAuOSkgICwgLk4gLCBieT0oYWdlKV0KCgpEZWx0YV9ITk1faW5jbHVkZWRfc3RhdHMgPC0gY2JpbmQoRGVsdGFfSE5NX3doaXBwZXRfaW5jbHVkZWRbb3JkZXIoYWdlKV0sIERlbHRhX0hOTV9NRV9pbmNsdWRlZFtvcmRlcihhZ2UpLCAyXSwgRGVsdGFfSE5NX3doaXBwZXRfTUVfaW5jbHVkZWRbb3JkZXIoYWdlKSwgMl0pCmNvbG5hbWVzKERlbHRhX0hOTV9pbmNsdWRlZF9zdGF0cykgPC0gYygiYWdlIiwgIldoaXBwZXQiLCAiTWljcm9FeG9uYXRvciIsICJCb3RoIikKCmBgYAoKCgoKYGBge3J9CgoKd2hpcHBldF9NRV9hZ2Vfc3RhdHMgPC0gZnVuY3Rpb24oRGVsdGFfU193aGlwcGV0LCBEZWx0YV9TX01FKSB7IAoKCiAgRGVsdGFfU193aGlwcGV0X01FIDwtIG1lcmdlKERlbHRhX1Nfd2hpcHBldCwgRGVsdGFfU19NRSwgYnk9YygiZXhvbl9JRCIsICJHZW5lIiwgIk5vZGUiLCAgIkNvb3JkIiwgIlN0cmFuZCIsICJUeXBlIiwgImFnZSIpICkKICAKICBEZWx0YV9TX3doaXBwZXRfTUVbIChhYnMoRGVsdGFQc2kueCk+PTAuMSAmICBQcm9iYWJpbGl0eS54ID49IDAuOSkgJiAgKGFicyhEZWx0YVBzaS55KT49MC4xICYgIFByb2JhYmlsaXR5LnkgPj0gMC45KSAsIC5OICwgYnk9KGFnZSldCiAgCiAgRGVsdGFfU193aGlwcGV0X01FX2luY2x1ZGVkIDwtIERlbHRhX1Nfd2hpcHBldF9NRVsgKERlbHRhUHNpLng8PS0wLjEgJiAgUHJvYmFiaWxpdHkueCA+PSAwLjkpICYgIChEZWx0YVBzaS55PD0tMC4xICYgIFByb2JhYmlsaXR5LnkgPj0gMC45KSAgLCAuTiAsIGJ5PShhZ2UpXQogIERlbHRhX1Nfd2hpcHBldF9NRV9leGNsdWRlZCA8LSBEZWx0YV9TX3doaXBwZXRfTUVbIChEZWx0YVBzaS54Pj0wLjEgJiAgUHJvYmFiaWxpdHkueCA+PSAwLjkpICYgIChEZWx0YVBzaS55Pj0wLjEgJiAgUHJvYmFiaWxpdHkueSA+PSAwLjkpICAsIC5OICwgYnk9KGFnZSldCiAgCiAgCiAgI0RlbHRhX1NfaW5jbHVkZWRfc3RhdHMgPC0gY2JpbmQoRGVsdGFfU193aGlwcGV0X2luY2x1ZGVkW29yZGVyKGFnZSldLCBEZWx0YV9TX01FX2luY2x1ZGVkW29yZGVyKGFnZSksIDJdLCBEZWx0YV9TX3doaXBwZXRfTUVfaW5jbHVkZWRbb3JkZXIoYWdlKSwgMl0pCiAgI2NvbG5hbWVzKERlbHRhX1NfaW5jbHVkZWRfc3RhdHMpIDwtIGMoImFnZSIsICJXaGlwcGV0IiwgIk1pY3JvRXhvbmF0b3IiLCAiQm90aCIpCiAgCiAgCiAgCiAgRGVsdGFfU193aGlwcGV0X01FWywgZGlmZl9oaWdoOj1GQUxTRV0KICBEZWx0YV9TX3doaXBwZXRfTUVbIChhYnMoRGVsdGFQc2kueCk+PTAuMSAmICBQcm9iYWJpbGl0eS54ID49IDAuOSkgJiAgKGFicyhEZWx0YVBzaS55KT49MC4xICYgIFByb2JhYmlsaXR5LnkgPj0gMC45KSAsIGRpZmZfaGlnaDo9VFJVRV0KICAKICBEZWx0YV9TX3doaXBwZXRfTUVbLCBkaWZmX2xvdzo9RkFMU0VdCiAgRGVsdGFfU193aGlwcGV0X01FWyAoYWJzKERlbHRhUHNpLngpPj0wLjEgJiAgUHJvYmFiaWxpdHkueCA+PSAwLjgpICYgIChhYnMoRGVsdGFQc2kueSk+PTAuMSAmICBQcm9iYWJpbGl0eS55ID49IDAuOCkgLCBkaWZmX2xvdzo9VFJVRV0KICAKICAKICBhZ2VzIDwtIHNvcnQodW5pcXVlKERlbHRhX1Nfd2hpcHBldF9NRSRhZ2UpKQogIGFnZV9pbnQgPC0gYyhhZ2VzW2xlbmd0aChhZ2VzKV0pCiAgCiAgCiAgICAKICBmb3IgKGEgaW4gcmV2KGFnZXNbMTpsZW5ndGgoYWdlcyktMV0pKXsKICAgIAogICAgYWdlX2ludCA9IGNiaW5kKGFnZV9pbnQsIGEpCiAgICAKICAgIERlbHRhX1Nfd2hpcHBldF9NRVsgYWdlPT1hICYgZGlmZl9oaWdoPT1UUlVFICYgIGV4b25fSUQgJWluJSBEZWx0YV9TX3doaXBwZXRfTUVbYWdlICVpbiUgYWdlX2ludCAmIGRpZmZfaGlnaD09VFJVRSwgLk4sIGJ5PWMoImV4b25fSUQiLCAiR2VuZSIpIF1bTj09bGVuZ3RoKGFnZV9pbnQpXSRleG9uX0lEICAgLCBkaWZmX2FnZTo9YSBdCiAgfQogICAgCiAgRGVsdGFfU193aGlwcGV0X01FX2FnZV9kaWZmIDwtIERlbHRhX1Nfd2hpcHBldF9NRVsgZGlmZl9oaWdoPT1UUlVFLCAuKGRpZmZfYWdlID0gbWluKGRpZmZfYWdlLCBuYS5ybSA9IFRSVUUpKSwgYnk9YygiZXhvbl9JRCIsICJHZW5lIildCiAgCiAgCiAgRGVsdGFfU193aGlwcGV0X01FJGRlbHRhX3NpbmcgPC0gc2lnbihEZWx0YV9TX3doaXBwZXRfTUUkRGVsdGFQc2kueCkKICAKICBEZWx0YV9TX3doaXBwZXRfTUVfZGlmZl9jb3VudCA8LSBEZWx0YV9TX3doaXBwZXRfTUVbZGlmZl9oaWdoPT1UUlVFICwgLk4gLCBieT1jKCJleG9uX0lEIiwgImRlbHRhX3NpbmciKV0KICByZXNjdWVfZXhvbnMgPC0gRGVsdGFfU193aGlwcGV0X01FX2RpZmZfY291bnRbTj49MiAmICFleG9uX0lEICVpbiUgRGVsdGFfU193aGlwcGV0X01FX2FnZV9kaWZmW2RpZmZfYWdlIT1JbmYsIF0kZXhvbl9JRCAsIF0kZXhvbl9JRAogIAogIAogICNhZ2VzIDwtIERlbHRhX1NfaW5jbHVkZWRfc3RhdHMkYWdlCiAgI2FnZV9pbnQgPC0gYyhhZ2VzW2xlbmd0aChhZ2VzKV0pCiAgRGVsdGFfU193aGlwcGV0X01FW2V4b25fSUQgJWluJSByZXNjdWVfZXhvbnNdCiAgCiAgICAKICBmb3IgKGEgaW4gcmV2KGFnZXNbMTpsZW5ndGgoYWdlcyktMV0pKXsKICAgIAogICAgYWdlX2ludCA9IGNiaW5kKGFnZV9pbnQsIGEpCiAgICAKICAgIERlbHRhX1Nfd2hpcHBldF9NRVtleG9uX0lEICVpbiUgcmVzY3VlX2V4b25zICYgYWdlPT1hICYgZGlmZl9sb3c9PVRSVUUgJiAgZXhvbl9JRCAlaW4lIERlbHRhX1Nfd2hpcHBldF9NRVthZ2UgJWluJSBhZ2VfaW50ICYgZGlmZl9sb3c9PVRSVUUsIC5OLCBieT1jKCJleG9uX0lEIiwgIkdlbmUiKSBdW049PWxlbmd0aChhZ2VfaW50KV0kZXhvbl9JRCAgICwgZGlmZl9hZ2VfbG93Oj1hIF0KICB9CiAgICAKICAKICBEZWx0YV9TX3doaXBwZXRfTUVfYWdlX2RpZmZfbG93IDwtIERlbHRhX1Nfd2hpcHBldF9NRVsgZGlmZl9sb3c9PVRSVUUsIC4oZGlmZl9hZ2UgPSBtaW4oZGlmZl9hZ2VfbG93LCBuYS5ybSA9IFRSVUUpKSwgYnk9YygiZXhvbl9JRCIsICJHZW5lIildCiAgCiAgCiAgZGlmZl9NRV9zaW5nIDwtIERlbHRhX1Nfd2hpcHBldF9NRVtkaWZmX2hpZ2g9PVRSVUUsIC1zaWduKERlbHRhUHNpLnkpXQogIG5hbWVzKGRpZmZfTUVfc2luZykgPC0gRGVsdGFfU193aGlwcGV0X01FW2RpZmZfaGlnaD09VFJVRSwgZXhvbl9JRF0KICAKICAKICBEZWx0YV9TX3doaXBwZXRfTUVfYWdlX2RpZmZfdG90YWwgPC0gIHJiaW5kKERlbHRhX1Nfd2hpcHBldF9NRV9hZ2VfZGlmZltkaWZmX2FnZSE9SW5mXSwgRGVsdGFfU193aGlwcGV0X01FX2FnZV9kaWZmX2xvd1tkaWZmX2FnZSE9SW5mXSkKICAKICBEZWx0YV9TX3doaXBwZXRfTUVfYWdlX2RpZmZfdG90YWxbLCBjaGFuZ2VfZGlyOj1kaWZmX01FX3NpbmdbZXhvbl9JRF0gXQogIAogIHJldHVybihEZWx0YV9TX3doaXBwZXRfTUVfYWdlX2RpZmZfdG90YWwpCgp9CgpgYGAKCgoKCmBgYHtyfQoKRGVsdGFfSE5NX3doaXBwZXRfTUVfYWdlX2RpZmZfdG90YWwgPC0gIHdoaXBwZXRfTUVfYWdlX3N0YXRzKERlbHRhX0hOTV93aGlwcGV0LCBEZWx0YV9ITk1fTUUpCkRlbHRhX0Zfd2hpcHBldF9NRV9hZ2VfZGlmZl90b3RhbCA8LSAgd2hpcHBldF9NRV9hZ2Vfc3RhdHMoRGVsdGFfRl93aGlwcGV0LCBEZWx0YV9GX01FKQoKCkRlbHRhX0Zfd2hpcHBldF9NRV9hZ2VfZGlmZl90b3RhbF9zdGF0cyA8LSAgRGVsdGFfRl93aGlwcGV0X01FX2FnZV9kaWZmX3RvdGFsWywgLihDb3VudD0uTikgLCBieT1jKCJkaWZmX2FnZSIsICJjaGFuZ2VfZGlyIikgXQpEZWx0YV9ITk1fd2hpcHBldF9NRV9hZ2VfZGlmZl90b3RhbF9zdGF0cyA8LSAgRGVsdGFfSE5NX3doaXBwZXRfTUVfYWdlX2RpZmZfdG90YWxbLCAuKENvdW50PS5OKSAsIGJ5PWMoImRpZmZfYWdlIiwgImNoYW5nZV9kaXIiKSBdCgpgYGAKCgoKCgoKCgoKCgpgYGB7cn0KCmdncGxvdCgpICsKICBnZW9tX2JhcihkYXRhPURlbHRhX0Zfd2hpcHBldF9NRV9hZ2VfZGlmZl90b3RhbF9zdGF0c1tjaGFuZ2VfZGlyPT0xXSwgYWVzKGZhY3RvcihkaWZmX2FnZSksIENvdW50KSwgc3RhdCA9ICJpZGVudGl0eSIsIGZpbGw9InNreWJsdWUiKSArCgogIGdlb21fYmFyKGRhdGE9RGVsdGFfRl93aGlwcGV0X01FX2FnZV9kaWZmX3RvdGFsX3N0YXRzW2NoYW5nZV9kaXI9PS0xXSwgYWVzKGZhY3RvcihkaWZmX2FnZSksIC1Db3VudCksIHN0YXQgPSAiaWRlbnRpdHkiLCBmaWxsPSJmaXJlYnJpY2siKSArCiAgdGhlbWUoYXhpcy50ZXh0LnggPSBlbGVtZW50X3RleHQoYW5nbGUgPSA5MCwgdmp1c3QgPSAwKSwKICAgIGF4aXMudGl0bGUgPSBlbGVtZW50X3RleHQoY29sb3VyID0gTkEpKQoKYGBgCgoKCgoKCgoKCgoKCgpgYGB7cn0KCgoKCgoKZ2dwbG90KCkgKwogIGdlb21fYmFyKGRhdGE9RGVsdGFfSE5NX3doaXBwZXRfTUVfYWdlX2RpZmZfdG90YWxfc3RhdHNbY2hhbmdlX2Rpcj09MV0sIGFlcyhmYWN0b3IoZGlmZl9hZ2UpLCBDb3VudCksIHN0YXQgPSAiaWRlbnRpdHkiLCBmaWxsPSJza3libHVlIikgKwoKICBnZW9tX2JhcihkYXRhPURlbHRhX0hOTV93aGlwcGV0X01FX2FnZV9kaWZmX3RvdGFsX3N0YXRzW2NoYW5nZV9kaXI9PS0xXSwgYWVzKGZhY3RvcihkaWZmX2FnZSksIC1Db3VudCksIHN0YXQgPSAiaWRlbnRpdHkiLCBmaWxsPSJmaXJlYnJpY2siKSArCiAgdGhlbWUoYXhpcy50ZXh0LnggPSBlbGVtZW50X3RleHQoYW5nbGUgPSA5MCwgdmp1c3QgPSAwKSwKICAgIGF4aXMudGl0bGUgPSBlbGVtZW50X3RleHQoY29sb3VyID0gTkEpKQoKYGBgCgoKCgpgYGB7ciwgIGZpZy5oZWlnaHQ9MTAsIGZpZy53aWR0aD0zfQoKCgpNRV9kaWZmX2FnZSA8LSBEZWx0YV9ITk1fd2hpcHBldF9NRV9hZ2VfZGlmZl90b3RhbCRkaWZmX2FnZQpuYW1lcyhNRV9kaWZmX2FnZSkgPC0gRGVsdGFfSE5NX3doaXBwZXRfTUVfYWdlX2RpZmZfdG90YWwkZXhvbl9JRAoKCmRpZmZfTUVfc2luZyA8LSBEZWx0YV9ITk1fd2hpcHBldF9NRV9hZ2VfZGlmZl90b3RhbCRjaGFuZ2VfZGlyCm5hbWVzKGRpZmZfTUVfc2luZykgPC0gRGVsdGFfSE5NX3doaXBwZXRfTUVfYWdlX2RpZmZfdG90YWwkZXhvbl9JRAoKTUVfY2x1c3RlcnNfdGFibGVbLCBkaWZmX2FnZTo9TkFdCk1FX2NsdXN0ZXJzX3RhYmxlWywgY2hhbmdlX2Rpcjo9TkFdCk1FX2NsdXN0ZXJzX3RhYmxlWywgZGlmZl9hZ2U6PU1FX2RpZmZfYWdlW01FXSBdCk1FX2NsdXN0ZXJzX3RhYmxlWywgY2hhbmdlX2Rpcjo9ZGlmZl9NRV9zaW5nW01FXSBdCgpNRV9jbHVzdGVyc190YWJsZVshaXMubmEoZGlmZl9hZ2UpXQoKCk1FX2NsdXN0ZXJzX3RhYmxlWyAsIFRvdGFsOj0uTiwgYnk9IE1FX2NsdXN0ZXJzXQoKTUVfY2x1c3RlcnNfdGFibGVfc3RhdHMgPC0gIE1FX2NsdXN0ZXJzX3RhYmxlWywgLk4gLCBieT1jKCJNRV9jbHVzdGVycyIsICJkaWZmX2FnZSIsICJjaGFuZ2VfZGlyIiwgIlRvdGFsIildCgpNRV9jbHVzdGVyc190YWJsZV9zdGF0c1ssIFBlcmNlbnRhZ2U6PU4qMTAwL1RvdGFsXQoKCk1FX2NsdXN0ZXJzX3RhYmxlX3N0YXRzWyAsIHN1bShOKSwgYnk9YyggImNoYW5nZV9kaXIiICkgXSAjVE9UQUwKTUVfY2x1c3RlcnNfdGFibGVfc3RhdHNbICwgc3VtKE4pLCBieT1jKCAiZGlmZl9hZ2UiLCAiY2hhbmdlX2RpciIgKSBdICNUT1RBTAoKTUVfY2x1c3RlcnNfdGFibGVfc3RhdHMgPC0gbWVyZ2UoTUVfY2x1c3RlcnNfdGFibGVfc3RhdHMsIE1FX2NsdXN0ZXJfbmFtZXNbLCBjKCJNRV9jbHVzdGVyIiwgIk1FX2NsdXN0ZXIubmFtZSIsICJNRV9jbHVzdGVyLnR5cGUiKV0sIGJ5Lng9Ik1FX2NsdXN0ZXJzIiwgYnkueT0iTUVfY2x1c3RlciIpCgpNRV9jbHVzdGVyc190YWJsZV9zdGF0c19ITk0gPC0gTUVfY2x1c3RlcnNfdGFibGVfc3RhdHNbZGlmZl9hZ2UhPSJOQSIgJiBjaGFuZ2VfZGlyIT0iTkEiXQoKCgpNRV9jbHVzdGVyc190YWJsZV9zdGF0c19ITk1fc3ViIDwtIE1FX2NsdXN0ZXJzX3RhYmxlX3N0YXRzX0hOTVtNRV9jbHVzdGVyLm5hbWUgJWluJSAgTUVfY2x1c3Rlcl9uYW1lc1tNRV9jbHVzdGVyLnR5cGUgJWluJSBjKCJOZXVyb25hbCIsICJOZXVyby1NdXNjdWxhciIsICJNdXNjdWxhciIsICJOb24tTmV1cm9uYWwiKSwgTUVfY2x1c3Rlci5uYW1lXSwgXQpNRV9jbHVzdGVyc190YWJsZV9zdGF0c19ITk1fc3ViJE1FX2NsdXN0ZXIubmFtZSA8LSBmYWN0b3IoTUVfY2x1c3RlcnNfdGFibGVfc3RhdHNfSE5NX3N1YiRNRV9jbHVzdGVyLm5hbWUsIGxldmVscyA9IHNvcnQodW5pcXVlKGFzLmNoYXJhY3RlcihNRV9jbHVzdGVyc190YWJsZV9zdGF0c19ITk1fc3ViJE1FX2NsdXN0ZXIubmFtZSkpKSkKCgoKRmlnNS5CIDwtIGdncGxvdCgpICsKICBnZW9tX2JhcihkYXRhPU1FX2NsdXN0ZXJzX3RhYmxlX3N0YXRzX0hOTV9zdWJbY2hhbmdlX2Rpcj09MV0sIGFlcyhmYWN0b3IoZGlmZl9hZ2UpLCBQZXJjZW50YWdlKSwgc3RhdCA9ICJpZGVudGl0eSIsIGZpbGw9InNreWJsdWUiKSArCiAgZ2VvbV9iYXIoZGF0YT1NRV9jbHVzdGVyc190YWJsZV9zdGF0c19ITk1fc3ViW2NoYW5nZV9kaXI9PS0xXSwgYWVzKGZhY3RvcihkaWZmX2FnZSksIC1QZXJjZW50YWdlKSwgc3RhdCA9ICJpZGVudGl0eSIsIGZpbGw9ImZpcmVicmljayIpICsKICBmYWNldF9ncmlkKCBNRV9jbHVzdGVyLm5hbWUgfiAuKSArCiAgeWxpbShjKC0zMCwgNzUpKSArCiAgeWxhYigiUGVyY2VudGFnZSIpICsKICB4bGFiKCIiKSArCiAgdGhlbWUoYXhpcy50ZXh0LnggPSBlbGVtZW50X3RleHQoYW5nbGUgPSA5MCwgdmp1c3QgPSAwKSkKRmlnNS5CCgpgYGAKCgoKYGBge3IsICBmaWcuaGVpZ2h0PTEwLCBmaWcud2lkdGg9M30KCgoKTUVfZGlmZl9hZ2UgPC0gRGVsdGFfRl93aGlwcGV0X01FX2FnZV9kaWZmX3RvdGFsJGRpZmZfYWdlCm5hbWVzKE1FX2RpZmZfYWdlKSA8LSBEZWx0YV9GX3doaXBwZXRfTUVfYWdlX2RpZmZfdG90YWwkZXhvbl9JRAoKCmRpZmZfTUVfc2luZyA8LSBEZWx0YV9GX3doaXBwZXRfTUVfYWdlX2RpZmZfdG90YWwkY2hhbmdlX2RpcgpuYW1lcyhkaWZmX01FX3NpbmcpIDwtIERlbHRhX0Zfd2hpcHBldF9NRV9hZ2VfZGlmZl90b3RhbCRleG9uX0lECgpNRV9jbHVzdGVyc190YWJsZVssIGRpZmZfYWdlOj1OQV0KTUVfY2x1c3RlcnNfdGFibGVbLCBjaGFuZ2VfZGlyOj1OQV0KTUVfY2x1c3RlcnNfdGFibGVbLCBkaWZmX2FnZTo9TUVfZGlmZl9hZ2VbTUVdIF0KTUVfY2x1c3RlcnNfdGFibGVbLCBjaGFuZ2VfZGlyOj1kaWZmX01FX3NpbmdbTUVdIF0KCk1FX2NsdXN0ZXJzX3RhYmxlWyFpcy5uYShkaWZmX2FnZSldCgoKCgojTUVfY2x1c3RlcnNfdGFibGVbICwgVG90YWw6PS5OLCBieT0gTUVfY2x1c3RlcnNdCgpNRV9jbHVzdGVyc190YWJsZV9zdGF0cyA8LSAgTUVfY2x1c3RlcnNfdGFibGVbLCAuTiAsIGJ5PWMoIk1FX2NsdXN0ZXJzIiwgImRpZmZfYWdlIiwgImNoYW5nZV9kaXIiLCAiVG90YWwiKV0KCk1FX2NsdXN0ZXJzX3RhYmxlX3N0YXRzWywgUGVyY2VudGFnZTo9TioxMDAvVG90YWxdCgoKIE1FX2NsdXN0ZXJzX3RhYmxlX3N0YXRzWyAsIHN1bShOKSwgYnk9YyggImNoYW5nZV9kaXIiICkgXSAjIFRvdGFsCgpNRV9jbHVzdGVyc190YWJsZV9zdGF0cyA8LSBtZXJnZShNRV9jbHVzdGVyc190YWJsZV9zdGF0cywgTUVfY2x1c3Rlcl9uYW1lc1ssIGMoIk1FX2NsdXN0ZXIiLCAiTUVfY2x1c3Rlci5uYW1lIiwgIk1FX2NsdXN0ZXIudHlwZSIpXSwgYnkueD0iTUVfY2x1c3RlcnMiLCBieS55PSJNRV9jbHVzdGVyIikgCk1FX2NsdXN0ZXJzX3RhYmxlX3N0YXRzX0YgPC0gTUVfY2x1c3RlcnNfdGFibGVfc3RhdHNbZGlmZl9hZ2UhPSJOQSIgJiBjaGFuZ2VfZGlyIT0iTkEiXQoKCk1FX2NsdXN0ZXJzX3RhYmxlX3N0YXRzX0Zfc3ViIDwtIE1FX2NsdXN0ZXJzX3RhYmxlX3N0YXRzX0ZbTUVfY2x1c3Rlci5uYW1lICVpbiUgIE1FX2NsdXN0ZXJfbmFtZXNbTUVfY2x1c3Rlci50eXBlICVpbiUgYygiTmV1cm9uYWwiLCAiTmV1cm8tTXVzY3VsYXIiLCAiTXVzY3VsYXIiLCAiTm9uLU5ldXJvbmFsIiksIE1FX2NsdXN0ZXIubmFtZV0sIF0KTUVfY2x1c3RlcnNfdGFibGVfc3RhdHNfRl9zdWIkTUVfY2x1c3Rlci5uYW1lIDwtIGZhY3RvcihNRV9jbHVzdGVyc190YWJsZV9zdGF0c19GX3N1YiRNRV9jbHVzdGVyLm5hbWUsIGxldmVscyA9IHNvcnQodW5pcXVlKGFzLmNoYXJhY3RlcihNRV9jbHVzdGVyc190YWJsZV9zdGF0c19GX3N1YiRNRV9jbHVzdGVyLm5hbWUpKSkpCgoKCgpGaWc1LkMgPC0gZ2dwbG90KCkgKwogIGdlb21fYmFyKGRhdGE9TUVfY2x1c3RlcnNfdGFibGVfc3RhdHNfRl9zdWJbY2hhbmdlX2Rpcj09MV0sIGFlcyhmYWN0b3IoZGlmZl9hZ2UpLCBQZXJjZW50YWdlKSwgc3RhdCA9ICJpZGVudGl0eSIsIGZpbGw9InNreWJsdWUiKSArCiAgZ2VvbV9iYXIoZGF0YT1NRV9jbHVzdGVyc190YWJsZV9zdGF0c19GX3N1YltjaGFuZ2VfZGlyPT0tMV0sIGFlcyhmYWN0b3IoZGlmZl9hZ2UpLCAtUGVyY2VudGFnZSksIHN0YXQgPSAiaWRlbnRpdHkiLCBmaWxsPSJmaXJlYnJpY2siKSArCiAgZmFjZXRfZ3JpZCggTUVfY2x1c3Rlci5uYW1lIH4gLikgKwogIHlsaW0oYygtMzAsIDc1KSkgKwogIHRoZW1lKGF4aXMudGV4dC54ID0gZWxlbWVudF90ZXh0KGFuZ2xlID0gOTAsIHZqdXN0ID0gMCksCiAgICBheGlzLnRpdGxlID0gZWxlbWVudF90ZXh0KGNvbG91ciA9IE5BKSkKCkZpZzUuQwoKYGBgCgoKCgoKCmBgYHtyfQoKRGVsdGFfU0tNX3doaXBwZXQgPC0gZnJlYWQoIi9Vc2Vycy9ncDcvR29vZ2xlX0RyaXZlL1Jlc3VsdHMvTUUvUGFwZXIvRmluYWxfUmVwb3J0L1JlcHMvUmVwMS9XaGlwcGV0L2NvbnRyb2xfdnNfc2tlbGV0YWxfbXVzY2xlX3Rpc3N1ZS5kaWZmLm1pY3JvZXhvbnMiKQpEZWx0YV9TS01fTUUgPC0gZnJlYWQoIi9Vc2Vycy9ncDcvR29vZ2xlX0RyaXZlL1Jlc3VsdHMvTUUvUGFwZXIvRmluYWxfUmVwb3J0L1JlcHMvUmVwMS9XaGlwcGV0L2NvbnRyb2xfdnNfc2tlbGV0YWxfbXVzY2xlX3Rpc3N1ZS5kaWZmLk1FLm1pY3JvZXhvbnMiKQpEZWx0YV9TS01fd2hpcHBldF9NRSA8LSBtZXJnZShEZWx0YV9TS01fd2hpcHBldCwgRGVsdGFfU0tNX01FLCBieT1jKCJleG9uX0lEIiwgIkdlbmUiLCAiTm9kZSIsICAiQ29vcmQiLCAiU3RyYW5kIiwgIlR5cGUiKSAgKQpEZWx0YV9TS01fd2hpcHBldF9NRVssIGRpZmZfaGlnaDo9RkFMU0VdCkRlbHRhX1NLTV93aGlwcGV0X01FWyAoYWJzKERlbHRhUHNpLngpPj0wLjEgJiAgUHJvYmFiaWxpdHkueCA+PSAwLjkpICYgIChhYnMoRGVsdGFQc2kueSk+PTAuMSAmICBQcm9iYWJpbGl0eS55ID49IDAuOSkgLCBkaWZmX2hpZ2g6PVRSVUVdCkRlbHRhX1NLTV93aGlwcGV0X01FW2RpZmZfaGlnaD09VFJVRSAsIGNoYW5nZV9kaXI6PXNpZ24oRGVsdGFQc2kueCkgXQpEZWx0YV9TS01fd2hpcHBldF9NRVssIFRpc3N1ZTo9IlNLTSJdCgpEZWx0YV9IZWFydF93aGlwcGV0IDwtIGZyZWFkKCIvVXNlcnMvZ3A3L0dvb2dsZV9Ecml2ZS9SZXN1bHRzL01FL1BhcGVyL0ZpbmFsX1JlcG9ydC9SZXBzL1JlcDEvV2hpcHBldC9jb250cm9sX3ZzX2hlYXJ0LmRpZmYubWljcm9leG9ucyIpCkRlbHRhX0hlYXJ0X01FIDwtIGZyZWFkKCIvVXNlcnMvZ3A3L0dvb2dsZV9Ecml2ZS9SZXN1bHRzL01FL1BhcGVyL0ZpbmFsX1JlcG9ydC9SZXBzL1JlcDEvV2hpcHBldC9jb250cm9sX3ZzX2hlYXJ0LmRpZmYuTUUubWljcm9leG9ucyIpCkRlbHRhX0hlYXJ0X3doaXBwZXRfTUUgPC0gbWVyZ2UoRGVsdGFfSGVhcnRfd2hpcHBldCwgRGVsdGFfSGVhcnRfTUUsIGJ5PWMoImV4b25fSUQiLCAiR2VuZSIsICJOb2RlIiwgICJDb29yZCIsICJTdHJhbmQiLCAiVHlwZSIpICApCkRlbHRhX0hlYXJ0X3doaXBwZXRfTUVbLCBkaWZmX2hpZ2g6PUZBTFNFXQpEZWx0YV9IZWFydF93aGlwcGV0X01FWyAoYWJzKERlbHRhUHNpLngpPj0wLjEgJiAgUHJvYmFiaWxpdHkueCA+PSAwLjkpICYgIChhYnMoRGVsdGFQc2kueSk+PTAuMSAmICBQcm9iYWJpbGl0eS55ID49IDAuOSkgLCBkaWZmX2hpZ2g6PVRSVUVdCkRlbHRhX0hlYXJ0X3doaXBwZXRfTUVbZGlmZl9oaWdoPT1UUlVFICwgY2hhbmdlX2Rpcjo9c2lnbihEZWx0YVBzaS54KSBdCkRlbHRhX0hlYXJ0X3doaXBwZXRfTUVbLCBUaXNzdWU6PSJIZWFydCJdCgoKCkRlbHRhX0FHX3doaXBwZXQgPC0gZnJlYWQoIi9Vc2Vycy9ncDcvR29vZ2xlX0RyaXZlL1Jlc3VsdHMvTUUvUGFwZXIvRmluYWxfUmVwb3J0L1JlcHMvUmVwMS9XaGlwcGV0L2NvbnRyb2xfdnNfYWRyZW5hbF9nbGFuZC5kaWZmLm1pY3JvZXhvbnMiKQpEZWx0YV9BR19NRSA8LSBmcmVhZCgiL1VzZXJzL2dwNy9Hb29nbGVfRHJpdmUvUmVzdWx0cy9NRS9QYXBlci9GaW5hbF9SZXBvcnQvUmVwcy9SZXAxL1doaXBwZXQvY29udHJvbF92c19hZHJlbmFsX2dsYW5kLmRpZmYuTUUubWljcm9leG9ucyIpCkRlbHRhX0FHX3doaXBwZXRfTUUgPC0gbWVyZ2UoRGVsdGFfQUdfd2hpcHBldCwgRGVsdGFfQUdfTUUsIGJ5PWMoImV4b25fSUQiLCAiR2VuZSIsICJOb2RlIiwgICJDb29yZCIsICJTdHJhbmQiLCAiVHlwZSIpICApCkRlbHRhX0FHX3doaXBwZXRfTUVbLCBkaWZmX2hpZ2g6PUZBTFNFXQpEZWx0YV9BR193aGlwcGV0X01FWyAoYWJzKERlbHRhUHNpLngpPj0wLjEgJiAgUHJvYmFiaWxpdHkueCA+PSAwLjkpICYgIChhYnMoRGVsdGFQc2kueSk+PTAuMSAmICBQcm9iYWJpbGl0eS55ID49IDAuOSkgLCBkaWZmX2hpZ2g6PVRSVUVdCkRlbHRhX0FHX3doaXBwZXRfTUVbZGlmZl9oaWdoPT1UUlVFICwgY2hhbmdlX2Rpcjo9c2lnbihEZWx0YVBzaS54KV0KRGVsdGFfQUdfd2hpcHBldF9NRVssIFRpc3N1ZTo9IkhlYXJ0Il0KCmBgYAoKCmBgYHtyLCBmaWcuaGVpZ2h0PTcsIGZpZy53aWR0aD01fQpuZXVyYWxfQUdfUFNJIDwtIG1lcmdlKG1lcmdlKApEZWx0YV9BR193aGlwcGV0X01FW2FicyhEZWx0YVBzaS54KT49MC4xICYgUHJvYmFiaWxpdHkueD4wLjkgJiBhYnMoRGVsdGFQc2kueSk+PTAuMSAmIFByb2JhYmlsaXR5Lnk+MC45LCAuKGV4b25fSUQsIFBTSV9BRz1Qc2lfQi54KSAgXSwKCkRlbHRhX0hOTV9tZXJnZVthYnMoRGVsdGFQc2kueCk+PTAuMSAmIFByb2JhYmlsaXR5Lng+MC45ICYgYWJzKERlbHRhUHNpLnkpPj0wLjEgJiBQcm9iYWJpbGl0eS55PjAuOSwgIC4oZXhvbl9JRCwgYWdlLCBQU0lfSE5NPVBzaV9CLngpXSwKYnk9ImV4b25fSUQiKSwKCkRlbHRhX0ZfbWVyZ2VbYWJzKERlbHRhUHNpLngpPj0wLjEgJiBQcm9iYWJpbGl0eS54PjAuOSAmIGFicyhEZWx0YVBzaS55KT49MC4xICYgUHJvYmFiaWxpdHkueT4wLjksICAuKGV4b25fSUQsIGFnZSwgUFNJX0Y9UHNpX0IueCldLApieT1jKCJleG9uX0lEIiwgImFnZSIpICkKCnRhYmxlKG5ldXJhbF9BR19QU0kkYWdlKSAjIE92ZXIgMTQuNSB0aGVyZSBpcyB0aGUgaGlnaGVzdCBudW1iZXIgb2YgaW50ZXJzZWN0aW9ucwoKCm5ldXJhbF9BR19QU0lfbWVsdGVkIDwtIG1lbHQobmV1cmFsX0FHX1BTSVthZ2U9PTE0LjUsIGMoImV4b25fSUQiLCAiUFNJX0FHIiwgIlBTSV9ITk0iLCAiUFNJX0YiKV0pCgoKClN1cC5BR19NSE5GLkEgPC0gZ2dwbG90KG5ldXJhbF9BR19QU0lfbWVsdGVkLCBhZXModmFyaWFibGUsIHZhbHVlKSkgKwogIGdlb21fYm94cGxvdCgpICsKICBnZW9tX2ppdHRlcigpICsKICAjZ2VvbV9saW5lKGFlcyhncm91cD1leG9uX0lEKSkgKwogIHhsYWIoIlRpc3N1ZSIpICsKICB5bGFiKCJQU0kiKSArCiAgZ2dzaWduaWY6Omdlb21fc2lnbmlmKHRlc3QgPSAid2lsY294LnRlc3QiLCBjb21wYXJpc29ucyA9IGxpc3QoYygiUFNJX0FHIiwgIlBTSV9ITk0iKSwgYygiUFNJX0FHIiwgIlBTSV9GIikpCiwgc3RlcF9pbmNyZWFzZT0wLjE1ICwgdGVzdC5hcmdzID0gbGlzdChhbHRlcm5hdGl2ZSA9ICJ0d28uc2lkZWQiLCBwYWlyZWQgPSBUUlVFKSwgbWFwX3NpZ25pZl9sZXZlbCA9IFRSVUUpICsKICBzY2FsZV94X2Rpc2NyZXRlKGxhYmVscyA9IGMoJ0FkcmVuYWwgR2xhbmQnLCdCcmFpbiBNSE4nLCdGb3JlYnJhaW4nKSkKCgoKU3VwLkFHX01ITkYuQiA8LSBnZ3Bsb3QobmV1cmFsX0FHX1BTSV9tZWx0ZWQsIGFlcyh2YXJpYWJsZSwgdmFsdWUpKSArCiAgI2dlb21fYm94cGxvdCgpICsKICAjZ2VvbV9qaXR0ZXIoKSArCiAgZ2VvbV9saW5lKGFlcyhncm91cD1leG9uX0lEKSkgKwogIHhsYWIoIlRpc3N1ZSIpICsKICB5bGFiKCJQU0kiKSArCiAgZ2dzaWduaWY6Omdlb21fc2lnbmlmKHRlc3QgPSAid2lsY294LnRlc3QiLCBjb21wYXJpc29ucyA9IGxpc3QoYygiUFNJX0FHIiwgIlBTSV9ITk0iKSwgYygiUFNJX0FHIiwgIlBTSV9GIikpCiwgc3RlcF9pbmNyZWFzZT0wLjE1ICwgdGVzdC5hcmdzID0gbGlzdChhbHRlcm5hdGl2ZSA9ICJ0d28uc2lkZWQiLCBwYWlyZWQgPSBUUlVFKSwgbWFwX3NpZ25pZl9sZXZlbCA9IFRSVUUpICsKICBzY2FsZV94X2Rpc2NyZXRlKGxhYmVscyA9IGMoJ0FkcmVuYWwgR2xhbmQnLCdCcmFpbiBNSE4nLCdGb3JlYnJhaW4nKSkKCgpwbG90X2dyaWQoU3VwLkFHX01ITkYuQSwgU3VwLkFHX01ITkYuQiwgbmNvbD0xLCBsYWJlbHM9IkFVVE8iKQoKYGBgCgoKCgpgYGB7ciwgIGZpZy5oZWlnaHQ9MTAsIGZpZy53aWR0aD0zfQoKCgpNRV9jbHVzdGVyc190YWJsZVsgLCBEaWZmX1NLTTo9IkZBTFNFIl0KTUVfY2x1c3RlcnNfdGFibGVbTUUgJWluJSBEZWx0YV9TS01fd2hpcHBldF9NRVtkaWZmX2hpZ2g9PVRSVUUgJiBjaGFuZ2VfZGlyPT0xLCBleG9uX0lEXSwgRGlmZl9TS006PSJFeGNsdWRlZCIgXQpNRV9jbHVzdGVyc190YWJsZVtNRSAlaW4lIERlbHRhX1NLTV93aGlwcGV0X01FW2RpZmZfaGlnaD09VFJVRSAmIGNoYW5nZV9kaXI9PS0xLCBleG9uX0lEXSwgRGlmZl9TS006PSJJbmNsdWRlZCJdCgpNRV9jbHVzdGVyc190YWJsZVsgLCBEaWZmX0hlYXJ0Oj0iRkFMU0UiXQpNRV9jbHVzdGVyc190YWJsZVtNRSAlaW4lIERlbHRhX0hlYXJ0X3doaXBwZXRfTUVbZGlmZl9oaWdoPT1UUlVFICYgY2hhbmdlX2Rpcj09MSwgZXhvbl9JRF0sIERpZmZfSGVhcnQ6PSJFeGNsdWRlZCIgXQpNRV9jbHVzdGVyc190YWJsZVtNRSAlaW4lIERlbHRhX0hlYXJ0X3doaXBwZXRfTUVbZGlmZl9oaWdoPT1UUlVFICYgY2hhbmdlX2Rpcj09LTEsIGV4b25fSURdLCBEaWZmX0hlYXJ0Oj0iSW5jbHVkZWQiXQoKTUVfY2x1c3RlcnNfdGFibGVbICwgRGlmZl9BRzo9IkZBTFNFIl0KTUVfY2x1c3RlcnNfdGFibGVbTUUgJWluJSBEZWx0YV9BR193aGlwcGV0X01FW2RpZmZfaGlnaD09VFJVRSAmIGNoYW5nZV9kaXI9PTEsIGV4b25fSURdLCBEaWZmX0FHOj0iRXhjbHVkZWQiIF0KTUVfY2x1c3RlcnNfdGFibGVbTUUgJWluJSBEZWx0YV9BR193aGlwcGV0X01FW2RpZmZfaGlnaD09VFJVRSAmIGNoYW5nZV9kaXI9PS0xLCBleG9uX0lEXSwgRGlmZl9BRzo9IkluY2x1ZGVkIl0KCgoKCgpNRV9jbHVzdGVyc190YWJsZV9zdGF0c19TS00gPC0gTUVfY2x1c3RlcnNfdGFibGVbIERpZmZfU0tNIT0iRkFMU0UiLCAuTiwgYnk9YygiTUVfY2x1c3RlcnMiLCAiVG90YWwiLCAiRGlmZl9TS00iKSBdCk1FX2NsdXN0ZXJzX3RhYmxlX3N0YXRzX1NLTVssIFBlcmNlbnRhZ2U6PShOKjEwMC9Ub3RhbCldCk1FX2NsdXN0ZXJzX3RhYmxlX3N0YXRzX1NLTVssIFRpc3N1ZTo9IlNLTSJdCmNvbG5hbWVzKE1FX2NsdXN0ZXJzX3RhYmxlX3N0YXRzX1NLTSkgPC0gYygiTUVfY2x1c3RlcnMiLCAiVG90YWwiLCAiRGlmZiIsICJOIiwgIlBlcmNlbnRhZ2UiLCAiVGlzc3VlIiApCgpNRV9jbHVzdGVyc190YWJsZV9zdGF0c19IZWFydCA8LSBNRV9jbHVzdGVyc190YWJsZVsgRGlmZl9IZWFydCE9IkZBTFNFIiwgLk4sIGJ5PWMoIk1FX2NsdXN0ZXJzIiwgIlRvdGFsIiwgIkRpZmZfSGVhcnQiKSBdCk1FX2NsdXN0ZXJzX3RhYmxlX3N0YXRzX0hlYXJ0WywgUGVyY2VudGFnZTo9KE4qMTAwL1RvdGFsKV0KTUVfY2x1c3RlcnNfdGFibGVfc3RhdHNfSGVhcnRbLCBUaXNzdWU6PSJIUlQiXQpjb2xuYW1lcyhNRV9jbHVzdGVyc190YWJsZV9zdGF0c19IZWFydCkgPC0gYygiTUVfY2x1c3RlcnMiLCAiVG90YWwiLCAiRGlmZiIsICJOIiwgIlBlcmNlbnRhZ2UiLCAiVGlzc3VlIiApCgpNRV9jbHVzdGVyc190YWJsZV9zdGF0c19BRyA8LSBNRV9jbHVzdGVyc190YWJsZVsgRGlmZl9BRyE9IkZBTFNFIiwgLk4sIGJ5PWMoIk1FX2NsdXN0ZXJzIiwgIlRvdGFsIiwgIkRpZmZfQUciKSBdCk1FX2NsdXN0ZXJzX3RhYmxlX3N0YXRzX0FHWywgUGVyY2VudGFnZTo9KE4qMTAwL1RvdGFsKV0KTUVfY2x1c3RlcnNfdGFibGVfc3RhdHNfQUdbLCBUaXNzdWU6PSJBRCJdCmNvbG5hbWVzKE1FX2NsdXN0ZXJzX3RhYmxlX3N0YXRzX0FHKSA8LSBjKCJNRV9jbHVzdGVycyIsICJUb3RhbCIsICJEaWZmIiwgIk4iLCAiUGVyY2VudGFnZSIsICJUaXNzdWUiICkKCgpNRV9jbHVzdGVyc190YWJsZV9zdGF0c19TSEEgPC0gcmJpbmQoTUVfY2x1c3RlcnNfdGFibGVfc3RhdHNfU0tNLCBNRV9jbHVzdGVyc190YWJsZV9zdGF0c19IZWFydCwgTUVfY2x1c3RlcnNfdGFibGVfc3RhdHNfQUcpCgoKCk1FX2NsdXN0ZXJzX3RhYmxlX3N0YXRzX1NIQSA8LSBtZXJnZShNRV9jbHVzdGVyc190YWJsZV9zdGF0c19TSEEsIE1FX2NsdXN0ZXJfbmFtZXNbLCBjKCJNRV9jbHVzdGVyIiwgIk1FX2NsdXN0ZXIubmFtZSIsICJNRV9jbHVzdGVyLnR5cGUiKV0sIGJ5Lng9Ik1FX2NsdXN0ZXJzIiwgYnkueT0iTUVfY2x1c3RlciIpCgpNRV9jbHVzdGVyc190YWJsZV9zdGF0c19TSEFfc3ViIDwtIE1FX2NsdXN0ZXJzX3RhYmxlX3N0YXRzX1NIQVtNRV9jbHVzdGVyLm5hbWUgJWluJSAgTUVfY2x1c3Rlcl9uYW1lc1tNRV9jbHVzdGVyLnR5cGUgJWluJSBjKCJOZXVyb25hbCIsICJOZXVyby1NdXNjdWxhciIsICJNdXNjdWxhciIsICJOb24tTmV1cm9uYWwiKSwgTUVfY2x1c3Rlci5uYW1lXSwgXQoKCk1FX2NsdXN0ZXJzX3RhYmxlX3N0YXRzX1NIQV9zdWIkTUVfY2x1c3Rlci5uYW1lIDwtIGZhY3RvcihNRV9jbHVzdGVyc190YWJsZV9zdGF0c19TSEFfc3ViJE1FX2NsdXN0ZXIubmFtZSwgbGV2ZWxzID0gc29ydCh1bmlxdWUoYXMuY2hhcmFjdGVyKE1FX2NsdXN0ZXJzX3RhYmxlX3N0YXRzX1NIQV9zdWIkTUVfY2x1c3Rlci5uYW1lKSkpKQoKCgoKRmlnNS5EIDwtIGdncGxvdCgpICsKICBnZW9tX2JhcihkYXRhPU1FX2NsdXN0ZXJzX3RhYmxlX3N0YXRzX1NIQV9zdWJbRGlmZj09IkluY2x1ZGVkIl0sIGFlcyhmYWN0b3IoVGlzc3VlKSwgUGVyY2VudGFnZSksIHN0YXQgPSAiaWRlbnRpdHkiLCBmaWxsPSJza3libHVlIikgKwogIGdlb21fYmFyKGRhdGE9TUVfY2x1c3RlcnNfdGFibGVfc3RhdHNfU0hBX3N1YltEaWZmPT0iRXhjbHVkZWQiXSwgYWVzKGZhY3RvcihUaXNzdWUpLCAtUGVyY2VudGFnZSksIHN0YXQgPSAiaWRlbnRpdHkiLCBmaWxsPSJmaXJlYnJpY2siKSArCiAgZmFjZXRfZ3JpZCggTUVfY2x1c3Rlci5uYW1lIH4gLikgKwogIHlsaW0oYygtMzAsIDc1KSkgKwogIHRoZW1lKGF4aXMudGV4dC54ID0gZWxlbWVudF90ZXh0KGFuZ2xlID0gOTAsIHZqdXN0ID0gMCksCiAgICBheGlzLnRpdGxlID0gZWxlbWVudF90ZXh0KGNvbG91ciA9IE5BKSkKRmlnNS5ECgpgYGAKCgpgYGB7ciwgZmlnLmhlaWdodD0gMTAsIGZpZy53aWR0aD0xNX0KbGlicmFyeShVcFNldFIpCgpyZXF1aXJlKFVwU2V0UikKCmxpc3RJbnB1dCA8LSBsaXN0KG9uZSA9IGMoMSwgMiwgMywgNSwgNywgOCwgMTEsIDEyLCAxMyksIHR3byA9IGMoMSwgMiwgNCwgNSwgCiAgICAxMCksIHRocmVlID0gYygxLCA1LCA2LCA3LCA4LCA5LCAxMCwgMTIsIDEzKSkKCgoKRGVsdGFfSE5NX3doaXBwZXRfTUVfYWdlX2RpZmZfdG90YWxbLCAgZXhvbl9JRF0KCkRlbHRhX0Zfd2hpcHBldF9NRV9hZ2VfZGlmZl90b3RhbFssIGV4b25fSUQgXQoKCnRpc3N1ZV9pbnRlcnNlY3Rpb24gPC0gbGlzdChCcmFpbiA9IE1FX2NsdXN0ZXJzX3RhYmxlW2RpZmZfYWdlIT0iTkEiLCBNRV0sCiAgICAgICAgICAgICAgICAgICAgICAgICAgICBTS00gPSBNRV9jbHVzdGVyc190YWJsZVtEaWZmX1NLTSE9RkFMU0UsIE1FXSwKICAgICAgICAgICAgICAgICAgICAgICAgICAgIEhlYXJ0ID0gTUVfY2x1c3RlcnNfdGFibGVbRGlmZl9IZWFydCE9RkFMU0UsIE1FXSwKICAgICAgICAgICAgICAgICAgICAgICAgICAgIEFHID0gTUVfY2x1c3RlcnNfdGFibGVbRGlmZl9BRyE9RkFMU0UsIE1FXQogICAgICAgICAgICAgICAgICAgICAgICAgICAgKQoKCnRpc3N1ZV9pbnRlcnNlY3Rpb24gPC0gbGlzdChCcmFpbl9ITk0gPSBEZWx0YV9ITk1fd2hpcHBldF9NRV9hZ2VfZGlmZl90b3RhbFssICBleG9uX0lEXSwKICAgICAgICAgICAgICAgICAgICAgICAgICAgIEJyYWluX0YgPSBEZWx0YV9GX3doaXBwZXRfTUVfYWdlX2RpZmZfdG90YWxbLCAgZXhvbl9JRF0sCiAgICAgICAgICAgICAgICAgICAgICAgICAgICBTS00gPSBNRV9jbHVzdGVyc190YWJsZVtEaWZmX1NLTSE9RkFMU0UsIE1FXSwKICAgICAgICAgICAgICAgICAgICAgICAgICAgIEhlYXJ0ID0gTUVfY2x1c3RlcnNfdGFibGVbRGlmZl9IZWFydCE9RkFMU0UsIE1FXSwKICAgICAgICAgICAgICAgICAgICAgICAgICAgIEFHID0gTUVfY2x1c3RlcnNfdGFibGVbRGlmZl9BRyE9RkFMU0UsIE1FXQogICAgICAgICAgICAgICAgICAgICAgICAgICAgKQoKCgpGaWc1LnkgPC0gdXBzZXQoZnJvbUxpc3QodGlzc3VlX2ludGVyc2VjdGlvbiksIG9yZGVyLmJ5ID0gImZyZXEiLCBuc2V0cyA9IDUsIGtlZXAub3JkZXIgPSBUKQpGaWc1LnkKYGBgCgoKCgoKYGBge3J9CmdnX2NvbG9yX2h1ZSA8LSBmdW5jdGlvbihuKSB7CiAgaHVlcyA9IHNlcSgxNSwgMzc1LCBsZW5ndGggPSBuICsgMSkKICBoY2woaCA9IGh1ZXMsIGwgPSA2NSwgYyA9IDEwMClbMTpuXQp9CgoKYGBgCgoKYGBge3J9CgoKbGlicmFyeShldWxlcnIpCgoKCnRpc3N1ZV9pbnRlcnNlY3Rpb24gPC0gbGlzdChCcmFpbiA9IE1FX2NsdXN0ZXJzX3RhYmxlW2RpZmZfYWdlIT0iTkEiLCBNRV0sCiAgICAgICAgICAgICAgICAgICAgICAgICAgICBTS00gPSBNRV9jbHVzdGVyc190YWJsZVtEaWZmX1NLTSE9RkFMU0UsIE1FXSwKICAgICAgICAgICAgICAgICAgICAgICAgICAgIEhlYXJ0ID0gTUVfY2x1c3RlcnNfdGFibGVbRGlmZl9IZWFydCE9RkFMU0UsIE1FXSwKICAgICAgICAgICAgICAgICAgICAgICAgICAgIEFHID0gTUVfY2x1c3RlcnNfdGFibGVbRGlmZl9BRyE9RkFMU0UsIE1FXSkKCgp0aXNzdWVfaW50ZXJzZWN0aW9uLnZlbm4gPC0gZXVsZXIoYygiQnJhaW4iID0gMjEyLCAgCiAgICAgICAgICAgICAgICAgICAgIlNLTSIgPSAzNSwgIAogICAgICAgICAgICAgICAgICAgICJIZWFydCIgPSAxMSwgCiAgICAgICAgICAgICAgICAgICAgIkFHIiA9IDExLAogICAgICAgICAgICAgICAgICAgICJCcmFpbiZBRyIgPSAyOSwKICAgICAgICAgICAgICAgICAgICAiU0tNJkhlYXJ0IiA9IDIxLAogICAgICAgICAgICAgICAgICAgICJCcmFpbiZIZWFydCIgPSAxOCwKICAgICAgICAgICAgICAgICAgICAiQnJhaW4mU0tNJkhlYXJ0IiA9IDE4LAogICAgICAgICAgICAgICAgICAgICJCcmFpbiZTS00iID0gMTQsCiAgICAgICAgICAgICAgICAgICAgIkJyYWluJlNLTSZIZWFydCZBRyIgPSAzLAogICAgICAgICAgICAgICAgICAgICJCcmFpbiZIZWFydCZBRyIgPSAyLAogICAgICAgICAgICAgICAgICAgICJIZWFydCZBRyIgPSAxLAogICAgICAgICAgICAgICAgICAgICJTS00mQUciID0gMSwKICAgICAgICAgICAgICAgICAgICAiQnJhaW4mU0tNJkFHIiA9IDEpKQoKCgoKdGlzc3VlX2ludGVyc2VjdGlvbi52ZW5uIDwtIGV1bGVyKGMoIkJyYWluX0hOTSZCcmFpbl9GIiA9IDE1NiwKICAgICAgICAgICAgICAgICAgICAgICAgICAgICAgICAgICAgIkJyYWluX0hOTSIgPSA3MSwKICAgICAgICAgICAgICAgICAgICAgICAgICAgICAgICAgICAgIlNLTSIgPSAzNCwKICAgICAgICAgICAgICAgICAgICAgICAgICAgICAgICAgICAgIkJyYWluX0hOTSZCcmFpbl9GJkFHIiA9IDI5LAogICAgICAgICAgICAgICAgICAgICAgICAgICAgICAgICAgICAiU0tNJkhlYXJ0IiA9IDIwLAogICAgICAgICAgICAgICAgICAgICAgICAgICAgICAgICAgICAiQnJhaW5fSE5NJkJyYWluX0YmSGVhcnQiID0gMTksCiAgICAgICAgICAgICAgICAgICAgICAgICAgICAgICAgICAgICJCcmFpbl9GIiA9IDE1LAogICAgICAgICAgICAgICAgICAgICAgICAgICAgICAgICAgICAiQnJhaW5fSE5NJkJyYWluX0YmU0tNJkhlYXJ0IiA9IDE0LAogICAgICAgICAgICAgICAgICAgICAgICAgICAgICAgICAgICAiQnJhaW5fSE5NJkJyYWluX0YmU0tNIiA9IDEzLAogICAgICAgICAgICAgICAgICAgICAgICAgICAgICAgICAgICAiSGVhcnQiID0gMTEsCiAgICAgICAgICAgICAgICAgICAgICAgICAgICAgICAgICAgICJBRyIgPSAxMSwKICAgICAgICAgICAgICAgICAgICAgICAgICAgICAgICAgICAgIkJyYWluX0hOTSZTS00iID0gNSwKICAgICAgICAgICAgICAgICAgICAgICAgICAgICAgICAgICAgIkJyYWluX0hOTSZCcmFpbl9GJlNLTSZIZWFydCZBRyIgPSA1LCAgICAgICAgICAgICAgICAgICAgICAgICAgICAgICAgICAgCiAgICAgICAgICAgICAgICAgICAgICAgICAgICAgICAgICAgICJCcmFpbl9ITk0mU0tNJkhlYXJ0IiA9IDQsCiAgICAgICAgICAgICAgICAgICAgICAgICAgICAgICAgICAgICJCcmFpbl9ITk0mSGVhcnQiID0gMywKICAgICAgICAgICAgICAgICAgICAgICAgICAgICAgICAgICAgIkJyYWluX0YmQUciID0gMiwKICAgICAgICAgICAgICAgICAgICAgICAgICAgICAgICAgICAgIkJyYWluX0hOTSZIZWFydCZBRyIgPSAyLAogICAgICAgICAgICAgICAgICAgICAgICAgICAgICAgICAgICAiSGVhcnQmQUciID0gMSwKICAgICAgICAgICAgICAgICAgICAgICAgICAgICAgICAgICAgIlNLTSZBRyIgPSAxICwKICAgICAgICAgICAgICAgICAgICAgICAgICAgICAgICAgICAgIkJyYWluX0YmU0tNIiA9IDEsCiAgICAgICAgICAgICAgICAgICAgICAgICAgICAgICAgICAgICJCcmFpbl9GJlNLTSZIZWFydCIgPSAxLAogICAgICAgICAgICAgICAgICAgICAgICAgICAgICAgICAgICAiQnJhaW5fSE5NJlNLTSZBRyIgPSAxLAogICAgICAgICAgICAgICAgICAgICAgICAgICAgICAgICAgICAiQnJhaW5fSE5NJkJyYWluX0YmSGVhcnQmQUciID0gMSwKICAgICAgICAgICAgICAgICAgICAgICAgICAgICAgICAgICAgIkJyYWluX0hOTSZCcmFpbl9GJlNLTSZBRyIgPSAxKSkKCiAgICAgICAgICAgICAgICAgICAgCiAgICAKdmVuIDwtIHBsb3QodGlzc3VlX2ludGVyc2VjdGlvbi52ZW5uLCBjb3VudHMgPSBGQUxTRSwgZm9udD00LCBjZXg9MSwgYWxwaGE9MC41LAogICAgIGZpbGw9Z2dfY29sb3JfaHVlKDUpLCBxdWFudGl0aWVzID0gbGlzdChmb250c2l6ZSA9IDApKQoKdmVuCgpgYGAKCgpgYGB7ciwgZmlnLmhlaWdodD0yMCwgZmlnLndpZHRoPTE2fQoKCmxpYnJhcnkoZ2dwbG90aWZ5KQoKCgpGaWc1LmNvbDIucm93MSA8LSBwbG90X2dyaWQoRmlnNS5CLCBGaWc1LkMsIEZpZzUuRCwgbnJvdz0xLCAgbGFiZWxzID0gYygiRiIsICJHIiwgIkgiKSwgcmVsX3dpZHRocyA9IGMoMiwgMS41LCAxLjUpICkKCkZpZzUuY29sMiA8LSBwbG90X2dyaWQoRmlnNS5jb2wyLnJvdzEsIGFzLmdyb2IoRmlnNS55KSwgbmNvbCA9IDEsICAgbGFiZWxzID0gYygiIiwgIkkiKSwgcmVsX2hlaWdodHMgPSBjKDMsIDEpICkKCkZpZzUuYm90dG9tIDwtIHBsb3RfZ3JpZChGaWc1LkEsIEZpZzUuY29sMiwgbnJvdz0xLCAgcmVsX3dpZHRocyA9IGMoMSwgMSksIGxhYmVscyA9IGMoIkUiLCAiIikpCgoKI0ZpZzUgPC0gcGxvdF9ncmlkKEZpZzUuY29sMSwgRmlnNS5CLCBGaWc1LkMsIEZpZzUuRCwgbnJvdz0xLCByZWxfd2lkdGhzID0gYygxMCwgMiwgMS41LCAxLjUgKSwgbGFiZWxzID0gYygiIiwgIkYiLCAiRyIsICJIIikgKQpGaWc1IDwtIHBsb3RfZ3JpZChGaWc1LlgsIEZpZzUuYm90dG9tLCBuY29sPTEsIHJlbF9oZWlnaHRzID0gYygxLCA0KSApCkZpZzUgCmBgYAoKCgoKI0Vycm9yCgoKCmBgYHtyfQoKRGVsdGFfSE5NX3doaXBwZXRfTUVfYWdlX2RpZmZbZGlmZl9hZ2UhPSJJbmYiLCBdJGV4b25fSUQKCkRlbHRhX0hOTV93aGlwcGV0X01FJGRlbHRhX3NpbmcgPC0gc2lnbihEZWx0YV9ITk1fd2hpcHBldF9NRSREZWx0YVBzaS54KQoKRGVsdGFfSE5NX3doaXBwZXRfTUVfZGlmZl9jb3VudCA8LSBEZWx0YV9ITk1fd2hpcHBldF9NRVtkaWZmX2hpZ2g9PVRSVUUgLCAuTiAsIGJ5PWMoImV4b25fSUQiLCAiZGVsdGFfc2luZyIpXQoKCgpEZWx0YV9ITk1fd2hpcHBldF9NRV9kaWZmX2NvdW50WyBOPT0yICYgIWV4b25fSUQgJWluJSBEZWx0YV9ITk1fd2hpcHBldF9NRV9hZ2VfZGlmZltkaWZmX2FnZSE9IkluZiIsIF0kZXhvbl9JRCwgXQoKRGVsdGFfSE5NX3doaXBwZXRfTUVfYWdlX2RpZmZfbG93W2RpZmZfYWdlIT1JbmZdCmBgYAoKCgpgYGB7cn0KCmxpYnJhcnkoYmlvbWFSdCkKCgoKCgpNaWNyb0V4b25fZ2VuZXMgPC0gZ3N1YigiXFwuLioiLCIiLCBEZWx0YV9ITk1fd2hpcHBldF9NRSRHZW5lKSAKbmFtZXMoTWljcm9FeG9uX2dlbmVzKSA8LSBEZWx0YV9ITk1fd2hpcHBldF9NRSRleG9uX0lECiAKRGVsdGFfSE5NX3doaXBwZXRfTUVfYWdlX2RpZmZfdG90YWxbLCBlbnNlbWJsX2dlbmVfaWQ6PU1pY3JvRXhvbl9nZW5lc1tleG9uX0lEXV0KCmVuc2VtYmwgPSB1c2VFbnNlbWJsKGJpb21hcnQ9ImVuc2VtYmwiLCBkYXRhc2V0PSJtbXVzY3VsdXNfZ2VuZV9lbnNlbWJsIikKCgoKCgpnZW5lX3RhYmxlIDwtIGRhdGEudGFibGUoZ2V0Qk0oYXR0cmlidXRlcz1jKCdlbnNlbWJsX2dlbmVfaWQnLCAibWdpX3N5bWJvbCIpLGZpbHRlcnMgPSAnZW5zZW1ibF9nZW5lX2lkJywgdmFsdWVzID0gRGVsdGFfSE5NX3doaXBwZXRfTUVfYWdlX2RpZmZfdG90YWwkZW5zZW1ibF9nZW5lX2lkICwgbWFydCA9IGVuc2VtYmwpKQoKCmdlbmVfdGFibGUgPC0gZGF0YS50YWJsZShnZXRCTShhdHRyaWJ1dGVzPWMoJ2Vuc2VtYmxfZ2VuZV9pZCcsICJtZ2lfc3ltYm9sIiksZmlsdGVycyA9ICdlbnNlbWJsX2dlbmVfaWQnLCB2YWx1ZXMgPSBEZWx0YV9ITk1fd2hpcHBldF9NRV9hZ2VfZGlmZl90b3RhbCRlbnNlbWJsX2dlbmVfaWQgLCBtYXJ0ID0gZW5zZW1ibCkpCgoKRGVsdGFfSE5NX3doaXBwZXRfTUVfYWdlX2RpZmZfdG90YWwgPC0gbWVyZ2UoIERlbHRhX0hOTV93aGlwcGV0X01FX2FnZV9kaWZmX3RvdGFsLCBnZW5lX3RhYmxlLCBieT0nZW5zZW1ibF9nZW5lX2lkJykKCgoKY2F0KGFzLmNoYXJhY3Rlcih1bmlxdWUoRGVsdGFfSE5NX3doaXBwZXRfTUVfYWdlX2RpZmZfdG90YWwkbWdpX3N5bWJvbCkpLCBzZXA9J1xuJykgCgoKRGVsdGFfSE5NX3doaXBwZXRfTUVfYWdlX2RpZmZfdG90YWxbbWdpX3N5bWJvbD09IkRjdG4yIl0KYGBgCgoKIyBJbnB1dCBmb3IgUFBJIG5ldHdvcmsKCgoKYGBge3J9CmdlbmVzX2JhY2tncm91bmQgPC0gdW5pcXVlKGMoRGVsdGFfSE5NX21lcmdlJEdlbmUueCwgRGVsdGFfRl9tZXJnZSRHZW5lLngpKQoKZ2VuZXNfYmFja2dyb3VuZCA8LSAgZ3N1YigiXFwuLioiLCIiLCBnZW5lc19iYWNrZ3JvdW5kKQoKCm5ldXJvbmFsX01FX2dlbmVzIDwtIHVuaXF1ZShjKERlbHRhX0hOTV93aGlwcGV0X01FX2FnZV9kaWZmX3RvdGFsJEdlbmUsIERlbHRhX0Zfd2hpcHBldF9NRV9hZ2VfZGlmZl90b3RhbCRHZW5lKSkKbmV1cm9uYWxfTUVfZ2VuZXMgPC0gZ3N1YigiXFwuLioiLCIiLCBuZXVyb25hbF9NRV9nZW5lcykKCm5ldXJvbmFsX01FX2dlbmVfbmFtZXMgPC0gIGdlbmVfdGFibGVbZW5zZW1ibF9nZW5lX2lkICVpbiUgbmV1cm9uYWxfTUVfZ2VuZXMsIF0KCmNhdChhcy5jaGFyYWN0ZXIodW5pcXVlKG5ldXJvbmFsX01FX2dlbmVfbmFtZXMkbWdpX3N5bWJvbCkpLCBzZXA9J1xuJykgCgoKZndyaXRlKGRhdGEudGFibGUoZ2VuZXNfYmFja2dyb3VuZCksICIuLi9QUEkvZ2VuZXNfYmFja2dyb3VuZC50eHQiLCBxdW90ZSA9IEZBTFNFLCBjb2wubmFtZXMgPSBGQUxTRSkKYGBgCgoKCgoKCmBgYHtyfQoKRGVsdGFfSE5NX3doaXBwZXRfTUVfYWdlX2RpZmZfdG90YWxbICwgZW5zZW1ibF9nZW5lX2lkOj1nc3ViKCJcXC4uKiIsIiIsIEdlbmUpIF0KRGVsdGFfSE5NX3doaXBwZXRfTUVfYWdlX2RpZmZfdG90YWxbICwgZW5zZW1ibF9nZW5lX2lkOj1nc3ViKCJcXC4uKiIsIiIsIEdlbmUpIF0KCgpEZWx0YV9ITk1GX3doaXBwZXRfTUVfYWdlX2RpZmZfdG90YWwgPC0gbWVyZ2UoRGVsdGFfSE5NX3doaXBwZXRfTUVfYWdlX2RpZmZfdG90YWwsIERlbHRhX0Zfd2hpcHBldF9NRV9hZ2VfZGlmZl90b3RhbCwgYnk9YygiZXhvbl9JRCIsICJHZW5lIiwgImNoYW5nZV9kaXIiKSwgYWxsPVRSVUUpCkRlbHRhX0hOTUZfd2hpcHBldF9NRV9hZ2VfZGlmZl90b3RhbFssIGRpZmZfYWdlOj1taW4oZGlmZl9hZ2UueSwgZGlmZl9hZ2UueCwgbmEucm09VFJVRSksIGJ5PWV4b25fSURdCgpEZWx0YV9ITk1GX3doaXBwZXRfTUVfYWdlX2RpZmZfdG90YWxbICwgZW5zZW1ibF9nZW5lX2lkOj1nc3ViKCJcXC4uKiIsIiIsIEdlbmUpIF0KCgoKCgpEZWx0YV9ITk1GX3doaXBwZXRfTUVfYWdlX2RpZmZfdG90YWwgPC0gbWVyZ2UoRGVsdGFfSE5NRl93aGlwcGV0X01FX2FnZV9kaWZmX3RvdGFsLCBuZXVyb25hbF9NRV9nZW5lX25hbWVzLCBieT0iZW5zZW1ibF9nZW5lX2lkIikKCgoKCiNtZXJnZSggRGVsdGFfSE5NRl93aGlwcGV0X01FX2FnZV9kaWZmX3RvdGFsLCBQUElfY2VudHJhbGl0eSwgYnk9Im1naV9zeW1ib2wiKQoKCgojRGVsdGFfSE5NRl93aGlwcGV0X01FX2FnZV9kaWZmX3RvdGFsWyFtZ2lfc3ltYm9sICVpbiUgUFBJX2NlbnRyYWxpdHkkbWdpX3N5bWJvbF0KCgpmd3JpdGUoZGF0YS50YWJsZShkYXRhLnRhYmxlKHVuaXF1ZShEZWx0YV9ITk1GX3doaXBwZXRfTUVfYWdlX2RpZmZfdG90YWwkZW5zZW1ibF9nZW5lX2lkKSkpLCAiLi4vRmluYWxfRmlndXJlcy9GaWd1cmU1L21pY3JvZXhvbnNfSE1ORl9nZW5lcy50eHQiLCBxdW90ZSA9IEZBTFNFLCBjb2wubmFtZXMgPSBGQUxTRSkKCmBgYAoKCmBgYHtyfQoKRGVsdGFfSGVhcnRfd2hpcHBldF9NRVsgLCBlbnNlbWJsX2dlbmVfaWQ6PWdzdWIoIlxcLi4qIiwiIiwgR2VuZSkgXQpEZWx0YV9TS01fd2hpcHBldF9NRVsgLCBlbnNlbWJsX2dlbmVfaWQ6PWdzdWIoIlxcLi4qIiwiIiwgR2VuZSkgXQpEZWx0YV9BR193aGlwcGV0X01FWyAsIGVuc2VtYmxfZ2VuZV9pZDo9Z3N1YigiXFwuLioiLCIiLCBHZW5lKSBdCgoKRGVsdGFfSGVhcnRfd2hpcHBldF9NRSA8LSBtZXJnZShEZWx0YV9IZWFydF93aGlwcGV0X01FLCBuZXVyb25hbF9NRV9nZW5lX25hbWVzLCBieT0iZW5zZW1ibF9nZW5lX2lkIikKRGVsdGFfU0tNX3doaXBwZXRfTUUgPC0gbWVyZ2UoRGVsdGFfU0tNX3doaXBwZXRfTUUsIG5ldXJvbmFsX01FX2dlbmVfbmFtZXMsIGJ5PSJlbnNlbWJsX2dlbmVfaWQiKQpEZWx0YV9BR193aGlwcGV0X01FIDwtIG1lcmdlKERlbHRhX0FHX3doaXBwZXRfTUUsIG5ldXJvbmFsX01FX2dlbmVfbmFtZXMsIGJ5PSJlbnNlbWJsX2dlbmVfaWQiKQoKZndyaXRlKGRhdGEudGFibGUoZGF0YS50YWJsZSh1bmlxdWUoRGVsdGFfSGVhcnRfd2hpcHBldF9NRVtkaWZmX2hpZ2g9PVRSVUUsIGVuc2VtYmxfZ2VuZV9pZF0pKSksICIuLi9GaW5hbF9GaWd1cmVzL0ZpZ3VyZTUvaGVhcnRfZ2VuZXMudHh0IiwgcXVvdGUgPSBGQUxTRSwgY29sLm5hbWVzID0gRkFMU0UpCmZ3cml0ZShkYXRhLnRhYmxlKGRhdGEudGFibGUodW5pcXVlKERlbHRhX1NLTV93aGlwcGV0X01FW2RpZmZfaGlnaD09VFJVRSwgZW5zZW1ibF9nZW5lX2lkXSkpKSwgIi4uL0ZpbmFsX0ZpZ3VyZXMvRmlndXJlNS9TS01fZ2VuZXMudHh0IiwgcXVvdGUgPSBGQUxTRSwgY29sLm5hbWVzID0gRkFMU0UpCmZ3cml0ZShkYXRhLnRhYmxlKGRhdGEudGFibGUodW5pcXVlKERlbHRhX0FHX3doaXBwZXRfTUVbZGlmZl9oaWdoPT1UUlVFLCBlbnNlbWJsX2dlbmVfaWRdKSkpLCAiLi4vRmluYWxfRmlndXJlcy9GaWd1cmU1L0FHX2dlbmVzLnR4dCIsIHF1b3RlID0gRkFMU0UsIGNvbC5uYW1lcyA9IEZBTFNFKQoKCkRlbHRhX0hlYXJ0X3doaXBwZXRfTUVbZGlmZl9oaWdoPT1UUlVFLCBlbnNlbWJsX2dlbmVfaWRdCkRlbHRhX1NLTV93aGlwcGV0X01FW2RpZmZfaGlnaD09VFJVRSwgZW5zZW1ibF9nZW5lX2lkXQpEZWx0YV9BR193aGlwcGV0X01FW2RpZmZfaGlnaD09VFJVRSwgZW5zZW1ibF9nZW5lX2lkXQpgYGAKCgoKYGBge3J9CgptaWNyb2V4b25zX1Zhc3RkYiA8LSByZWFkX2RlbGltKCJ+L0dvb2dsZV9Ecml2ZS9SZXN1bHRzL01FL21tMTAvVmFzdERCL1Zhc3REYi5taWNyb2V4b25zLnR4dCIsIAogICAgIiAiLCBlc2NhcGVfZG91YmxlID0gRkFMU0UsIGNvbF9uYW1lcyA9IEZBTFNFLCAKICAgIHRyaW1fd3MgPSBUUlVFKQoKCm1pY3JvZXhvbnNfVmFzdGRiIDwtIG1pY3JvZXhvbnNfVmFzdGRiJFgxCgoKCm1pY3JvZXhvbnNfR0VOQ09ERSA8LSByZWFkX2RlbGltKCJ+L0dvb2dsZV9Ecml2ZS9SZXN1bHRzL01FL21tMTAvZ2VuY29kZS52TTE2LmFubm90YXRpb24ubWljcm9leG9ucy50eHQiLCAKICAgICIgIiwgZXNjYXBlX2RvdWJsZSA9IEZBTFNFLCBjb2xfbmFtZXMgPSBGQUxTRSwgCiAgICB0cmltX3dzID0gVFJVRSkKCgptaWNyb2V4b25zX0dFTkNPREUgPC0gbWljcm9leG9uc19HRU5DT0RFJFgxCmBgYAoKCgpgYGB7cn0KCgoKTUVfZmluYWxbICwgdHlwZTo9IkFubm90YXRlZCIgIF0KTUVfZmluYWxbIU1FICVpbiUgbWljcm9leG9uc19WYXN0ZGIsIHR5cGU6PSJNaXNzaW5nIGluIFZhc3REQiIgICBdCk1FX2ZpbmFsWyFNRSAlaW4lIG1pY3JvZXhvbnNfR0VOQ09ERSwgdHlwZTo9Ik1pc3NpbmcgaW4gR0VOQ09ERSIgICBdCk1FX2ZpbmFsWyFNRSAlaW4lIG1pY3JvZXhvbnNfR0VOQ09ERSAmICFNRSAlaW4lIG1pY3JvZXhvbnNfVmFzdGRiICwgdHlwZTo9Ik5vdmVsIiAgIF0KCgpNRV9maW5hbFssIEFsdDVfMzo9Ik5BIl0KCk1FX2ZpbmFsW01FICVpbiUgTUVfY292W0FsdDUhPSJOb25lIiwgTUVfY29vcmRzXSwgQWx0NV8zOj0iQWx0NSIgXQpNRV9maW5hbFtNRSAlaW4lIE1FX2NvdltBbHQzIT0iTm9uZSIsIE1FX2Nvb3Jkc10sIEFsdDVfMzo9IkFsdDMiIF0KCk1FX2ZpbmFsW01FICVpbiUgTUVfY292W0FsdDUhPSJOb25lIiwgTUVfY29vcmRzXSAmIE1FICVpbiUgTUVfY292W0FsdDMhPSJOb25lIiwgTUVfY29vcmRzXSwgQWx0NV8zOj0iQWx0NV8zIiBdCgpsZW5ndGgodW5pcXVlKE1FX2ZpbmFsJE1FKSkKCk1FX2ZpbmFsWyAsIC5OICwgYnk9YygiQWx0NV8zIiwgInR5cGUiKSBdCgpucm93KHVuaXF1ZShNRV9maW5hbC5vdXQpKQoKTUVfZmluYWwub3V0IDwtICBtZXJnZSgKdW5pcXVlKE1FX2ZpbmFsKSwKIE1FX2NsdXN0ZXJzLmluZm9bLCAuKE1FLCBNRV9jbHVzdGVyLnR5cGUsIE1FX2NsdXN0ZXIubmFtZSwgTUVfY2x1c3Rlci5udW1iZXI9TUVfY2x1c3RlciwgUEMxLCBQQzIsIFBDMyApXSwgYWxsLng9VFJVRSkKCgogIE1FX2NsdXN0ZXJzLmluZm9bLCAuKCJNRSIsICJNRV9jbHVzdGVyLnR5cGUiLCAiTUVfY2x1c3Rlci5uYW1lIildCgpNRV9maW5hbC5vdXRbICwgSW4uMTBfcGVyY2VudF9vZl9idWxrOj1NRSAlaW4lIG1lX2FmdGVyXzEwX3NhbXBsZXNfZmlsdGVyIF0gIAogICAKZndyaXRlKCBNRV9maW5hbC5vdXQsICJ+L0Rlc2t0b3AvTUVfZmluYWwudGFibGVfbWFydGluLnR4dCIsIHF1b3RlID0gRkFMU0UsIGNvbC5uYW1lcyA9IFRSVUUsIHNlcD0iXHQiLCApCgoKYGBgCgoKYGBge3J9Ck1FX2ZpbmFsLm91dFtJbi4xMF9wZXJjZW50X29mX2J1bGs9PVRSVUUsIC5OLCBieT0idHlwZSJdCmBgYAoKCgpgYGB7cn0KCk1FX2NsdXN0ZXJzLnRhYmxlCgpgYGAKCgoKCmBgYHtyfQoKI25ldXJvbmFsX01FX0hOTUYgIDwtIHVuaXF1ZShyYmluZChEZWx0YV9ITk1fd2hpcHBldF9NRV9hZ2VfZGlmZl90b3RhbFssIGMoImV4b25fSUQiLCAiR2VuZSIpXSwgRGVsdGFfRl93aGlwcGV0X01FX2FnZV9kaWZmX3RvdGFsWywgYygiZXhvbl9JRCIsICJHZW5lIildICkpCm5ldXJvbmFsX01FX0hOTUYgIDwtIHVuaXF1ZShtZXJnZShEZWx0YV9ITk1fd2hpcHBldF9NRV9hZ2VfZGlmZl90b3RhbCwgRGVsdGFfRl93aGlwcGV0X01FX2FnZV9kaWZmX3RvdGFsLCBieT1jKCJleG9uX0lEIiwgIkdlbmUiKSwgYWxsPVQgKSkKCgoKbmV1cm9uYWxfTUVfSE5NRlsgLCB0eXBlOj0iQW5ub3RhdGVkIiAgXQoKbmV1cm9uYWxfTUVfSE5NRlshZXhvbl9JRCAlaW4lIG1pY3JvZXhvbnNfVmFzdGRiLCB0eXBlOj0iTWlzc2luZyBpbiBWYXN0REIiICAgXQoKbmV1cm9uYWxfTUVfSE5NRlshZXhvbl9JRCAlaW4lIG1pY3JvZXhvbnNfR0VOQ09ERSwgdHlwZTo9Ik1pc3NpbmcgaW4gR0VOQ09ERSIgICBdCgpuZXVyb25hbF9NRV9ITk1GWyFleG9uX0lEICVpbiUgbWljcm9leG9uc19HRU5DT0RFICYgIWV4b25fSUQgJWluJSBtaWNyb2V4b25zX1Zhc3RkYiAsIHR5cGU6PSJOb3ZlbCIgICBdCgpuZXVyb25hbF9NRV9ITk1GLnRhYmxlIDwtIHVuaXF1ZShtZXJnZSggZ2VuZV90YWJsZS5UT1RBTFssIGMoIk1FIiwgIm1naV9zeW1ib2wiKV0sIG5ldXJvbmFsX01FX0hOTUYgLCBieS54PSJNRSIsIGJ5Lnk9ImV4b25fSUQiKSkKCgpuZXVyb25hbF9NRV9ITk1GLnRhYmxlIDwtIG5ldXJvbmFsX01FX0hOTUYudGFibGVbb3JkZXIobWdpX3N5bWJvbCwgTUUpXQoKZndyaXRlKG5ldXJvbmFsX01FX0hOTUYudGFibGUsICIuLi8uLi8uLi8uLi9UaGVzaXMvbmV1cm9uYWxfTUVfSE5NRi50c3YiLCBzZXA9Ilx0IiApCgoKbmV1cm9uYWxfTUVfSE5NRl9zdGF0cyA8LSBuZXVyb25hbF9NRV9ITk1GWyAsIC5OICwgYnk9InR5cGUiXQoKbmV1cm9uYWxfTUVfSE5NRl9zdGF0c1ssIFRvdGFsOj1zdW0oTildCgpuZXVyb25hbF9NRV9ITk1GX3N0YXRzWywgUGVyY2VudGFnZTo9TioxMDAvVG90YWxdCgpgYGAKCgoKYGBge3J9CgojbGlicmFyeSgiU1RSSU5HZGIiKQoKI3N0cmluZ19kYiA8LSBTVFJJTkdkYiRuZXcoIHZlcnNpb249IjEwIiwgc3BlY2llcz05NjA2LCBzY29yZV90aHJlc2hvbGQ9MCwgaW5wdXRfZGlyZWN0b3J5PSIiICkKCgojbmV1cm9uYWxfTUVfZ2VuZV9uYW1lc19tYXBwZWQgPC0gc3RyaW5nX2RiJG1hcCggbmV1cm9uYWxfTUVfZ2VuZV9uYW1lcywgIm1naV9zeW1ib2wiLCByZW1vdmVVbm1hcHBlZFJvd3MgPSBUUlVFICkKCiMgc3RyaW5nX2RiJHBsb3RfbmV0d29yayggbmV1cm9uYWxfTUVfZ2VuZV9uYW1lc19tYXBwZWQgKQoKCgojaGl0cyA8LSBhcy5jaGFyYWN0ZXIodW5pcXVlKG5ldXJvbmFsX01FX2dlbmVfbmFtZXMkbWdpX3N5bWJvbCkpCgojZXhhbXBsZTFfbWFwcGVkIDwtIHN0cmluZ19kYiRtYXAoIGhpdHMsICJnZW5lIiwgcmVtb3ZlVW5tYXBwZWRSb3dzID0gVFJVRSApCgojcGxvdF9uZXR3b3JrKCBoaXRzICkKCiNkYXRhKGRpZmZfZXhwX2V4YW1wbGUxKQoKCiNleGFtcGxlMV9tYXBwZWQgPC0gc3RyaW5nX2RiJG1hcCggZGlmZl9leHBfZXhhbXBsZTEsICJnZW5lIiwgcmVtb3ZlVW5tYXBwZWRSb3dzID0gVFJVRSApCgojbWFwCgojP1NUUklOR2RiCmBgYAoKCgoKCgoKYGBge3J9CgoKbmV1cm9uYWxfY2x1c3RlcnNfZ2VuZXMgPC0gbWVyZ2UobmV1cm9uYWxfY2x1c3RlcnMsIGdlbmVfaW5mbywgYnkueD0idHJhbnNjcmlwdCIsIGJ5Lnk9ImVuc2VtYmxfdHJhbnNjcmlwdF9pZCIpCgpuZXVyb25hbF9jbHVzdGVyc19nZW5lcyRNRV9jbHVzdGVycyA8LSBtYXB2YWx1ZXMobmV1cm9uYWxfY2x1c3RlcnNfZ2VuZXMkTUVfY2x1c3RlcnMsIAogICAgICAgICAgZnJvbSA9MToxOCwKICAgICAgICAgIHRvID0gYygiSTEiLCAiRTEiLCAiRTMiLCAiSTIiLCAiTjEiLCAiTTEiLCAiTjIiLCAiTk0zIiwgIk5NMiIsICJONSIsICJOTTEiLCAiTjMiLCAiTjQiLCAiTk4yIiwgIkUyIiwgIkk0IiwgIkkzIiwgIk5OMSIpKQoKI0dlbmVyYXJpbmcgdmFsdWVzIGZvciBwaWUgdmVydGljcwoKY29sb3VyX3BhbGxldGUgPC0gbGlzdCggYyhoZWF0LmNvbG9ycyg4KSwgIiMxRTkwRkZGRiIpKQp2YWx1ZXNfY2x1c3RlcnMgPSBsaXN0KCkKCk1FX3Blcl9nZW5lID0gbGlzdCgpCgp2ZXJ0ZXhfc2hhcGUgPSBsaXN0KCkKY2lyY2xlX2NvbG9yID0gbGlzdCgpCgpsYWJlbF9jb2xvciA9IGxpc3QoKQoKZm9yIChpIGluIDE6MTQ0KXsKICAKCiAgZ2VuZV9uYW1lID0gbm9kZXNbaSwgXSRpZAoKCiAgaWYgKGk9PTE2KXsKICAgIAogICAgZ2VuZV9uYW1lID0gIlNwdGFuMSIgICAjIFNUUklORyBEQiBjaGFuZ2VkIHRoZSBuYW1lIGZyb20gU3BuYTIgdG8gU3BhdGFuMQogIH0KICAKICBpZiAoaT09NDkpewogICAgCiAgICBnZW5lX25hbWUgPSAiRGlhcGgxIiAgICMjIFNUUklORyBEQiBjaGFuZ2VkIHRoZSBuYW1lIGZyb20gRGlhcDEgdG8gRGlhcGgxCiAgfQogIAogIAogIE1FX3Blcl9nZW5lW1tpXV0gPC0gbnJvdyhuZXVyb25hbF9jbHVzdGVyc19nZW5lc1ttZ2lfc3ltYm9sPT1nZW5lX25hbWUsIF0pCgoKICBOMSA9ICBucm93KG5ldXJvbmFsX2NsdXN0ZXJzX2dlbmVzW21naV9zeW1ib2w9PWdlbmVfbmFtZSAgJiBNRV9jbHVzdGVycz09Ik4xIiwgXSkKICBOMiA9ICBucm93KG5ldXJvbmFsX2NsdXN0ZXJzX2dlbmVzW21naV9zeW1ib2w9PWdlbmVfbmFtZSAgJiBNRV9jbHVzdGVycz09Ik4yIiwgXSkKICBOMyA9ICBucm93KG5ldXJvbmFsX2NsdXN0ZXJzX2dlbmVzW21naV9zeW1ib2w9PWdlbmVfbmFtZSAgJiBNRV9jbHVzdGVycz09Ik4zIiwgXSkKICBONCA9ICBucm93KG5ldXJvbmFsX2NsdXN0ZXJzX2dlbmVzW21naV9zeW1ib2w9PWdlbmVfbmFtZSAgJiBNRV9jbHVzdGVycz09Ik40IiwgXSkKICBONSA9ICBucm93KG5ldXJvbmFsX2NsdXN0ZXJzX2dlbmVzW21naV9zeW1ib2w9PWdlbmVfbmFtZSAgJiBNRV9jbHVzdGVycz09Ik41IiwgXSkKICBOTTEgPSAgbnJvdyhuZXVyb25hbF9jbHVzdGVyc19nZW5lc1ttZ2lfc3ltYm9sPT1nZW5lX25hbWUgICYgTUVfY2x1c3RlcnM9PSJOTTEiLCBdKQogIE5NMiA9ICBucm93KG5ldXJvbmFsX2NsdXN0ZXJzX2dlbmVzW21naV9zeW1ib2w9PWdlbmVfbmFtZSAgJiBNRV9jbHVzdGVycz09Ik5NMiIsIF0pCiAgTk0zID0gIG5yb3cobmV1cm9uYWxfY2x1c3RlcnNfZ2VuZXNbbWdpX3N5bWJvbD09Z2VuZV9uYW1lICAmIE1FX2NsdXN0ZXJzPT0iTk0zIiwgXSkKICBOTjEgPSAgbnJvdyhuZXVyb25hbF9jbHVzdGVyc19nZW5lc1ttZ2lfc3ltYm9sPT1nZW5lX25hbWUgICYgTUVfY2x1c3RlcnM9PSJOTjEiLCBdKQogIAogIAogICN2YWx1ZXNfY2x1c3RlcnNbW2ldXSA8LSBjKE4xLCBOMiwgTjMsIE40LCBONSwgTk0xLCBOTTIsIE5NMywgTk4xKQogIAogIGNsdXN0ZXJfY291bnRzIDwtIGMoTk0xLCBOMSwgTk0yLCAgTjIsIE5NMywgTjMsIE40LCBONSwgIE5OMSkKICAKICB2YWx1ZXNfY2x1c3RlcnNbW2ldXSA8LSBjbHVzdGVyX2NvdW50cwogIAogIGlmIChsZW5ndGgod2hpY2goY2x1c3Rlcl9jb3VudHMhPTApICkgPT0gMSAgKSB7CiAgICAKICAgIHZlcnRleF9zaGFwZVtbaV1dIDwtICJjaXJjbGUiICAgI05vZGVzIHdpdGggb25seSBvbmUga2luZCBvZiBjbHVzdGVyIG5lZWQgdG8gYmUgY2lyY2xlcyBpbnN0ZWFkIG9mIHBpZSB0byBhdm9pZCBwaWUgYm9yZGVyCiAgICBjaXJjbGVfY29sb3JbW2ldXSA8LSAgY29sb3VyX3BhbGxldGVbWzFdXVt3aGljaChjbHVzdGVyX2NvdW50cyE9MCldIAogICAgCiAgICAKICB9IGVsc2UgewogICAgCiAgICAKICAgIHZlcnRleF9zaGFwZVtbaV1dIDwtICJwaWUiCiAgICBjaXJjbGVfY29sb3JbW2ldXSA8LSAgIiNGRkZGMDBGRiIgICAjVGhpcyBjb2xvdXIgaXMgbm90IHRha2VuIGluIGNvbnNpZGVyYXRpb24gZm9yIHBpZXMKICAgIAogICAgCiAgfQogIAogIAogICNpZiAoZ2VuZV9uYW1lICVpbiUgIFNGQVJJX0dlbmVfYW5pbWFsX2dlbmVzW21vZGVsLnNwZWNpZXM9PSJNdXMgbXVzY3VsdXMiICwgZ2VuZS5zeW1ib2xdKSB7CiAgICAKICBpZiAoZ2VuZV9uYW1lICVpbiUgIFNGQVJJX0dlbmVfaHVtYW5fZ2VuZXNbIGdlbmUuc2NvcmU8PTMgLCBnZW5lLnN5bWJvbF0pIHsgIAogICAgCiAgICAKICAgIAogICAgbGFiZWxfY29sb3JbW2ldXSA8LSAiZGFya2dyZWVuIgogICAgCiAgICAKICB9IGVsc2V7CiAgICAKICAgICBsYWJlbF9jb2xvcltbaV1dIDwtICJibGFjayIKICAgIAogIH0KICAKICAgIAogICAgCiAgaWYgKCBzdW0oIGMoIE4xLCBOMiwgTjMsIE40LCBONSwgTk0xLCBOTTIsIE5NMywgTk4xKSkgPT0gMCApIHsKICAgIAogICAgcHJpbnQoIGMoaSwgbm9kZXNbaSwgXSRpZCApICkKICB9CiAgCgp9CgoKCmBgYAoKCgoKCgoKCmBgYHtyLCBmaWcuaGVpZ2h0PTIwLCBmaWcud2lkdGg9MjB9CgpsaWJyYXJ5KGlncmFwaCkKCnZlcnRleC5waWUuY29sb3I9bGlzdChoZWF0LmNvbG9ycyg5KSkKCgoKCgpNRV9QUEkgPC0gZnJlYWQoIi4uL1BQSS9NRS5QUEkudHN2IikKCgoKCmxpbmtzIDwtIE1FX1BQSVssIGMoIiNub2RlMSIsICJub2RlMiIsICJjb21iaW5lZF9zY29yZSIpXQoKCgpjb2xuYW1lcyhsaW5rcykgPC0gYygiZnJvbSIsICJ0byIsICJzY29yZSIpCgpub2Rlcy4xIDwtIE1FX1BQSVssIGMoIiNub2RlMSIpXQpjb2xuYW1lcyhub2Rlcy4xKSA8LSBjKCJpZCIpCgpub2Rlcy4yIDwtIE1FX1BQSVssIGMoIm5vZGUyIildCmNvbG5hbWVzKG5vZGVzLjIpIDwtIGMoImlkIikKCgpub2RlcyA8LSByYmluZChub2Rlcy4xLCBub2Rlcy4yKQoKCm5vZGVzIDwtIHVuaXF1ZSggIG5vZGVzWyAsICwgYnk9ImlkIl0gKQoKCgpuZXQgPC0gZ3JhcGhfZnJvbV9kYXRhX2ZyYW1lKGQ9bGlua3MsIHZlcnRpY2VzPW5vZGVzLCBkaXJlY3RlZD1GKQoKRShuZXQpJHNpemUgPC0gbG9nKDItICBFKG5ldCkkc2NvcmUpCgpFKG5ldCkkd2lkdGggPC0gKDEtICBFKG5ldCkkc2NvcmUpICAqIDE1CgoKCgojVihuZXQpJHNpemUgPC0gbG9nKHVubGlzdChNRV9wZXJfZ2VuZSkgKyAyKSAqIDQKCiNFKG5ldCkkc2NvcmUKCiNwbG90KG5ldCwgdmVydGV4LnNoYXBlPSB1bmxpc3QodmVydGV4X3NoYXBlKSwgdmVydGV4LmNvbG9yID0gdW5saXN0KGNpcmNsZV9jb2xvcikgICwgdmVydGV4LnBpZT12YWx1ZXNfY2x1c3RlcnMsIHJlc2NhbGU9VCwgcGllLmx0eT0yLCB2ZXJ0ZXgucGllLmNvbG9yPWNvbG91cl9wYWxsZXRlLCB2ZXJ0ZXgubGFiZWwuZm9udD0yLCB2ZXJ0ZXgubGFiZWwuZGlzdD0wLjQsIHZlcnRleC5sYWJlbC5jb2xvcj11bmxpc3QobGFiZWxfY29sb3IpLCB2ZXJ0ZXgubGFiZWwuY2V4PTEuMikKCiNjb2xvdXJfcGFsbGV0ZQoKCiNwbG90KG5ldCxyZXNjYWxlPVQsIHZlcnRleC5sYWJlbC5jZXg9MS4yLCB2ZXJ0ZXguc2l6ZT0xICkKCgoKCgojaGFybW9uaWNfY2VudHJhbGl0eSA8LSBQUElfZGlmZl9hZ2VfSE5NRiRoYXJtb25pY19jZW50cmFsaXR5CiNuYW1lcyhoYXJtb25pY19jZW50cmFsaXR5KSA8LSBQUElfZGlmZl9hZ2VfSE5NRiRtYXBwZWRfZ2VuZQoKI1YobmV0KSRzaXplIDwtIGxvZyhoYXJtb25pY19jZW50cmFsaXR5W25hbWVzKFYobmV0KSldICsxICkKCgojVihuZXQpJHNpemUgPC0gaGFybW9uaWNfY2VudHJhbGl0eVtuYW1lcyhWKG5ldCkpXS82CgojcGxvdChuZXQpCgoKCgojUFBJX2RpZmZfYWdlX0hOTV9jZW50cmFsIFsgICVpbiUgbmFtZXMoVihuZXQpKQoKI2hhcm1vbmljX2NlbnRyYWxpdHlbbmFtZXMoVihuZXQpKV0KCgojaGFybW9uaWNfY2VudHJhbGl0eVtuYW1lcyhWKG5ldCkpXQoKCgoKI2hhcm1vbmljX2NlbnRyYWxpdHlbbmFtZXMoVihuZXQpKV0KCgojbmFtZXMoVihuZXQpKSAKCiNQUElfZGlmZl9hZ2VfSE5NX2NlbnRyYWxbICAobWdpX3N5bWJvbCAlaW4lICBuYW1lcyhWKG5ldCkpKT09RkFMU0UgLCAgXQoKI1BQSV9kaWZmX2FnZV9ITk1fY2VudHJhbFttZ2lfc3ltYm9sPT0iSXRzbjEiXQoKI1BQSV9kaWZmX2FnZV9ITk1GCmBgYAoKCgoKYGBge3J9CmxpYnJhcnkoQ0lOTkEpCgpQUElfYmV0d2Vlbm5lc3MgPC0gZXN0aW1hdGVfYmV0d2Vlbm5lc3MobmV0LCB2aWRzID0gVihuZXQpLCBkaXJlY3RlZCA9IEYsIGN1dG9mZj0tMSwKICB3ZWlnaHRzID0gTlVMTCwgbm9iaWdpbnQgPSBUUlVFKQoKUFBJX2VpZ2VuX2NlbnRyYWxpdHkgPC0gZWlnZW5fY2VudHJhbGl0eShuZXQsIGRpcmVjdGVkID0gRiwgc2NhbGUgPSBULCB3ZWlnaHRzID0gTlVMTCkkdmVjdG9yCgoKI2NiaW5kKGFzLm51bWVyaWMoUFBJX2JldHdlZW5uZXNzW1YobmV0KV0pLCBhcy5udW1lcmljKCBQUElfZWlnZW5fY2VudHJhbGl0eVtWKG5ldCldICApICkKClBQSV9oYXJtb25pY19jZW50cmFsaXR5IDwtIGhhcm1vbmljX2NlbnRyYWxpdHkobmV0KQoKClBQSV9kZWdyZWVfY2VudHJhbGl0eSA8LSBjZW50cl9kZWdyZWUobmV0KSRyZXMKbmFtZXMoUFBJX2RlZ3JlZV9jZW50cmFsaXR5KSA8LSBWKG5ldCkkbmFtZQoKUFBJX2NlbnRyYWxpdHkgPC0gZGF0YS5mcmFtZShjYmluZCggIFBQSV9iZXR3ZWVubmVzc1tWKG5ldCldICwgIFBQSV9laWdlbl9jZW50cmFsaXR5W1YobmV0KV0sIFBQSV9oYXJtb25pY19jZW50cmFsaXR5LCBQUElfZGVncmVlX2NlbnRyYWxpdHkgICApKQoKY29sbmFtZXMoUFBJX2NlbnRyYWxpdHkpIDwtIGMoImJldHdlZW5uZXNzIiwgImVpZ2VuX2NlbnRyYWxpdHkiLCAiaGFybW9uaWNfY2VudHJhbGl0eSIsICJkZWdyZWVfY2VudHJhbGl0eSIpCgpQUElfY2VudHJhbGl0eSRtYXBwZWRfZ2VuZSA8LSAgcm93Lm5hbWVzKFBQSV9jZW50cmFsaXR5KQoKUFBJX2NlbnRyYWxpdHkgPC0gZGF0YS50YWJsZShQUElfY2VudHJhbGl0eSkKCgpgYGAKCgoKCgoKYGBge3IsIGZpZy5oZWlnaHQ9NiwgZmlnLndpZHRoPTh9CgpsaWJyYXJ5KGdncmVwZWwpCmxpYnJhcnkoY293cGxvdCkKbGlicmFyeShyZWFkcikKClBQSV9nZW5lcyA8LSByZWFkX2RlbGltKCJ+L0dvb2dsZV9Ecml2ZS9SZXN1bHRzL01FL1BhcGVyL1BQSS9NRS5QUEkuYW5ub3RhdGlvbnMudHN2IiwgCiAgICAiXHQiLCBlc2NhcGVfZG91YmxlID0gRkFMU0UsIHRyaW1fd3MgPSBUUlVFKQoKUFBJX2dlbmVzIDwtIGZyZWFkKCIuLi9GaWd1cmVzL05ld19GaWd1cmUgNS9TVFJJTkcvSE1ORl9NRV9nZW5lLlNUUklOR19tYXAudHh0Iiwgc2VwPSJcdCIpCgpEZWx0YV9ITk1GX3doaXBwZXRfTUVfYWdlX2RpZmZfdG90YWwKCgpEZWx0YV9ITk1GX3doaXBwZXRfTUVfYWdlX2RpZmZfdG90YWxfUFBJIDwtIG1lcmdlKERlbHRhX0hOTUZfd2hpcHBldF9NRV9hZ2VfZGlmZl90b3RhbCwgUFBJX2dlbmVzLCAgYnkueD0iZW5zZW1ibF9nZW5lX2lkIiwgYnkueT0iaW5wdXRfdGVybSIpCgpQUElfZGlmZl9hZ2VfSE5NRiA8LSBtZXJnZShEZWx0YV9ITk1GX3doaXBwZXRfTUVfYWdlX2RpZmZfdG90YWxfUFBJLCBQUElfY2VudHJhbGl0eSwgYnk9Im1hcHBlZF9nZW5lIikKICAKICAKICAKCiNuZXVyb25hbF9NRV9nZW5lX25hbWVzX2RpZmZfYWdlIDwtIG1lcmdlKG5ldXJvbmFsX01FX2dlbmVfbmFtZXMsIFBQSV9jZW50cmFsaXR5LCBieT0ibWdpX3N5bWJvbCIpCgojRGVsdGFfSE5NX3doaXBwZXRfTUVfYWdlX2RpZmZfdG90YWxbICwgZW5zZW1ibF9nZW5lX2lkOj1nc3ViKCJcXC4uKiIsIiIsIEdlbmUpIF0KCgojUFBJX2RpZmZfYWdlX0hOTUYgPC0gbWVyZ2UoRGVsdGFfSE5NRl93aGlwcGV0X01FX2FnZV9kaWZmX3RvdGFsLCBuZXVyb25hbF9NRV9nZW5lX25hbWVzX2RpZmZfYWdlLCBieT0iZW5zZW1ibF9nZW5lX2lkIikKCiNQUElfZGlmZl9hZ2VfSE5NRiA8LSB1bmlxdWUoUFBJX2RpZmZfYWdlX0hOTSkKCgoKUFBJX2RpZmZfYWdlX0hOTUZbICwgdHlwZTo9IkFubm90YXRlZCIgIF0KClBQSV9kaWZmX2FnZV9ITk1GWyFleG9uX0lEICVpbiUgbWljcm9leG9uc19WYXN0ZGIsIHR5cGU6PSJNaXNzaW5nIGluIFZhc3REQiIgICBdCgpQUElfZGlmZl9hZ2VfSE5NRlshZXhvbl9JRCAlaW4lIG1pY3JvZXhvbnNfR0VOQ09ERSwgdHlwZTo9Ik1pc3NpbmcgaW4gR0VOQ09ERSIgICBdCgpQUElfZGlmZl9hZ2VfSE5NRlshZXhvbl9JRCAlaW4lIG1pY3JvZXhvbnNfR0VOQ09ERSAmICFleG9uX0lEICVpbiUgbWljcm9leG9uc19WYXN0ZGIgLCB0eXBlOj0iTm92ZWwiICAgXQoKCgoKTDFpbnQgPC0gYygiQW5rMSIsICJBcDJtMSIsICJLY25xMiIsICJOZmFzYyIsICJTcmMiLCAiQW5rMiIsICJEbm0xIiwgIkwxY2FtIiwgIk5yY2FtIiwgIlZhdjIiLCAiQW5rMyIsICJEbm0zIiwgIk5jYW0xIiwgIlNwdGFuMSIpCiNQUElfZGlmZl9hZ2VfSE5NWywgTDFjYW1fYW5kX2ludGVyYWN0b3JzIDo9IG1naV9zeW1ib2wgJWluJSBMMWludF0KCgpQUElfZGlmZl9hZ2VfSE5NRl9zdGF0c19IQyA8LSBQUElfZGlmZl9hZ2VfSE5NRlssICAuKG1pbl9kaWZfYWdlPW1pbihkaWZmX2FnZSksIE51bWJlcl9vZl9NRT0uTiksIGJ5PWMoImVuc2VtYmxfZ2VuZV9pZCIsICJoYXJtb25pY19jZW50cmFsaXR5IikgXQpQUElfZGlmZl9hZ2VfSE5NRl9zdGF0c19IQ1ssIHJhbms6PWZyYW5rKC1oYXJtb25pY19jZW50cmFsaXR5KV0KUFBJX2RpZmZfYWdlX0hOTUZfc3RhdHNfSENbICwgbWF4X3Jhbms6PW1heChyYW5rKV0KUFBJX2RpZmZfYWdlX0hOTUZfc3RhdHNfSENbICwgcGVyY2VudGlsOj1yYW5rL21heF9yYW5rKjEwMF0KUFBJX2RpZmZfYWdlX0hOTUZfc3RhdHNfSENbLCBjZW50cmFsOj1GQUxTRV0KUFBJX2RpZmZfYWdlX0hOTUZfc3RhdHNfSENbcGVyY2VudGlsPDE1LCBjZW50cmFsOj1UUlVFXQoKClBQSV9kaWZmX2FnZV9ITk1GX3N0YXRzX0IgPC0gUFBJX2RpZmZfYWdlX0hOTUZbLCAgLihtaW5fZGlmX2FnZT1taW4oZGlmZl9hZ2UpLCBOdW1iZXJfb2ZfTUU9Lk4pLCBieT1jKCJlbnNlbWJsX2dlbmVfaWQiLCAiYmV0d2Vlbm5lc3MiKSBdClBQSV9kaWZmX2FnZV9ITk1GX3N0YXRzX0JbLCByYW5rOj1mcmFuaygtYmV0d2Vlbm5lc3MpXQpQUElfZGlmZl9hZ2VfSE5NRl9zdGF0c19CWyAsIG1heF9yYW5rOj1tYXgocmFuayldClBQSV9kaWZmX2FnZV9ITk1GX3N0YXRzX0JbICwgcGVyY2VudGlsOj1yYW5rL21heF9yYW5rKjEwMF0KUFBJX2RpZmZfYWdlX0hOTUZfc3RhdHNfQlssIGNlbnRyYWw6PUZBTFNFXQpQUElfZGlmZl9hZ2VfSE5NRl9zdGF0c19CW3BlcmNlbnRpbDwxNSwgY2VudHJhbDo9VFJVRV0KCgpQUElfZGlmZl9hZ2VfSE5NRl9zdGF0c19FQyA8LSBQUElfZGlmZl9hZ2VfSE5NRlssICAuKG1pbl9kaWZfYWdlPW1pbihkaWZmX2FnZSksIE51bWJlcl9vZl9NRT0uTiksIGJ5PWMoImVuc2VtYmxfZ2VuZV9pZCIsICJlaWdlbl9jZW50cmFsaXR5IiwgInR5cGUiLCAibWFwcGVkX2dlbmUiKSBdClBQSV9kaWZmX2FnZV9ITk1GX3N0YXRzX0VDWywgcmFuazo9ZnJhbmsoLWVpZ2VuX2NlbnRyYWxpdHkpXQpQUElfZGlmZl9hZ2VfSE5NRl9zdGF0c19FQ1sgLCBtYXhfcmFuazo9bWF4KHJhbmspXQpQUElfZGlmZl9hZ2VfSE5NRl9zdGF0c19FQ1sgLCBwZXJjZW50aWw6PXJhbmsvbWF4X3JhbmsqMTAwXQpQUElfZGlmZl9hZ2VfSE5NRl9zdGF0c19FQ1ssIGNlbnRyYWw6PUZBTFNFXQpQUElfZGlmZl9hZ2VfSE5NRl9zdGF0c19FQ1twZXJjZW50aWw8MjUsIGNlbnRyYWw6PVRSVUVdCgoKUFBJX2RpZmZfYWdlX0hOTUZfY2VudHJhbCA8LSBtZXJnZShQUElfZGlmZl9hZ2VfSE5NRiwgUFBJX2RpZmZfYWdlX0hOTUZfc3RhdHNfRUNbLCBjKCJlbnNlbWJsX2dlbmVfaWQiLCAiY2VudHJhbCIpXSwgYnk9ImVuc2VtYmxfZ2VuZV9pZCIgKQoKCkY2RiA8LSBnZ3Bsb3QoUFBJX2RpZmZfYWdlX0hOTUZfY2VudHJhbCkgKwogIGdlb21faml0dGVyKGFlcyhlaWdlbl9jZW50cmFsaXR5LCBkaWZmX2FnZSwgIGNvbG91cj0gdHlwZSAgKSwgd2lkdGg9MCwgaGVpZ2h0PTAuMSkgKyAKICB4bGFiKCJFaWdlbmNlbnRyYWxpdHkiKSArCiAgeWxhYigiRWFybGllc3QgbWljcm9leG9uIGRpZmZlcmVudGlhbCBpbmNsdXNpb24gZGV0ZWN0aW9uIChEUEMpIikgKwogICBzY2FsZV95X2NvbnRpbnVvdXMoYnJlYWtzPXNlcSgxMC41LDE2LjUsMSkpICsKICAgIGdlb21fdGV4dF9yZXBlbChkYXRhPVBQSV9kaWZmX2FnZV9ITk1GX2NlbnRyYWxbICBjZW50cmFsPT1UUlVFICBdLCAKICAgICAgICAgICAgICAgICAgYWVzKHg9ZWlnZW5fY2VudHJhbGl0eSwgeT1kaWZmX2FnZSwgY29sb3VyPSB0eXBlICksIAogICAgICAgICAgICAgICAgICBudWRnZV95ICAgICAgPSAwLjUsCiAgICAgICAgICAgICAgICAgIGRpcmVjdGlvbiAgICA9ICJ4IiwKICAgICAgICAgICAgICAgICAgYW5nbGUgICAgICAgID0gOTAsCiAgICAgICAgICAgICAgICAgIHNlZ21lbnQuc2l6ZSA9IDAuMiwKICAgICAgICAgICAgICAgICAgbGFiZWw9UFBJX2RpZmZfYWdlX0hOTUZfY2VudHJhbFtjZW50cmFsPT1UUlVFICAsIG1hcHBlZF9nZW5lXSwKICAgICAgICAgICAgICAgICAgc2hvdy5sZWdlbmQgPSBGQUxTRSkgKwogICAgICAgICAgdGhlbWUoYXhpcy50ZXh0LnggPSBlbGVtZW50X3RleHQoYW5nbGUgPSA5MCksIGxlZ2VuZC5wb3NpdGlvbiA9ICJ0b3AiLCBsZWdlbmQuZGlyZWN0aW9uID0gImhvcml6b250YWwiKSArCiAgICAgIHRoZW1lKGxlZ2VuZC5wb3NpdGlvbj0ibm9uZSIpCgoKCgoKUFBJX2RpZmZfYWdlX0hOTUZfY2VudHJhbCA8LSBtZXJnZShQUElfZGlmZl9hZ2VfSE5NRiwgUFBJX2RpZmZfYWdlX0hOTUZfc3RhdHNfSENbLCBjKCJlbnNlbWJsX2dlbmVfaWQiLCAiY2VudHJhbCIpXSwgYnk9ImVuc2VtYmxfZ2VuZV9pZCIgKQoKCkY2LnN1cC4xIDwtIGdncGxvdChQUElfZGlmZl9hZ2VfSE5NRl9jZW50cmFsKSArCiAgZ2VvbV9qaXR0ZXIoYWVzKGhhcm1vbmljX2NlbnRyYWxpdHksIGRpZmZfYWdlLCAgY29sb3VyPSB0eXBlICApLCB3aWR0aD0wLCBoZWlnaHQ9MC4xKSArIAogIHhsYWIoIkhhcm1vbmljIENlbnRyYWxpdHkiKSArCiAgeWxhYigiRWFybGllc3QgbWljcm9leG9uIGRpZmZlcmVudGlhbCBpbmNsdXNpb24gZGV0ZWN0aW9uIChEUEMpIikgKwogICBzY2FsZV95X2NvbnRpbnVvdXMoYnJlYWtzPXNlcSgxMC41LDE2LjUsMSkpICsKICAgIGdlb21fdGV4dF9yZXBlbChkYXRhPVBQSV9kaWZmX2FnZV9ITk1GX2NlbnRyYWxbICBjZW50cmFsPT1UUlVFICBdLCAKICAgICAgICAgICAgICAgICAgYWVzKHg9aGFybW9uaWNfY2VudHJhbGl0eSwgeT1kaWZmX2FnZSwgY29sb3VyPSB0eXBlICksIAogICAgICAgICAgICAgICAgICBudWRnZV95ICAgICAgPSAwLjUsCiAgICAgICAgICAgICAgICAgIGRpcmVjdGlvbiAgICA9ICJ4IiwKICAgICAgICAgICAgICAgICAgYW5nbGUgICAgICAgID0gOTAsCiAgICAgICAgICAgICAgICAgIHNlZ21lbnQuc2l6ZSA9IDAuMiwKICAgICAgICAgICAgICAgICAgbGFiZWw9UFBJX2RpZmZfYWdlX0hOTUZfY2VudHJhbFtjZW50cmFsPT1UUlVFICAsIG1hcHBlZF9nZW5lXSwKICAgICAgICAgICAgICAgICAgc2hvdy5sZWdlbmQgPSBUUlVFKSArCiAgICAgICAgICB0aGVtZShheGlzLnRleHQueCA9IGVsZW1lbnRfdGV4dChhbmdsZSA9IDkwKSwgbGVnZW5kLnBvc2l0aW9uID0gInRvcCIsIGxlZ2VuZC5kaXJlY3Rpb24gPSAiaG9yaXpvbnRhbCIpCgpGNkYKYGBgCgoKYGBge3J9CgpQUElfZGlmZl9hZ2VfSE5NX3N0YXRzX0VDIDwtIFBQSV9kaWZmX2FnZV9ITk1GW2lzLm5hKGRpZmZfYWdlLngpIT1UUlVFXVssICAuKG1pbl9kaWZfYWdlPW1pbihkaWZmX2FnZS54KSwgTnVtYmVyX29mX01FPS5OKSwgYnk9YygiZW5zZW1ibF9nZW5lX2lkIiwgImVpZ2VuX2NlbnRyYWxpdHkiKSBdClBQSV9kaWZmX2FnZV9ITk1fc3RhdHNfRUNbLCByYW5rOj1mcmFuaygtZWlnZW5fY2VudHJhbGl0eSldClBQSV9kaWZmX2FnZV9ITk1fc3RhdHNfRUNbICwgbWF4X3Jhbms6PW1heChyYW5rKV0KUFBJX2RpZmZfYWdlX0hOTV9zdGF0c19FQ1sgLCBwZXJjZW50aWw6PXJhbmsvbWF4X3JhbmsqMTAwXQpQUElfZGlmZl9hZ2VfSE5NX3N0YXRzX0VDWywgY2VudHJhbDo9RkFMU0VdClBQSV9kaWZmX2FnZV9ITk1fc3RhdHNfRUNbcGVyY2VudGlsPDE1LCBjZW50cmFsOj1UUlVFXQoKCgpnZ3Bsb3QoUFBJX2RpZmZfYWdlX0hOTV9zdGF0c19FQykgKwogIGdlb21fYmFyKGFlcyhtaW5fZGlmX2FnZSwgZmlsbD1jZW50cmFsKSwgc3RhdCA9ICJjb3VudCIsIHBvc2l0aW9uPSJmaWxsIiApICsKICB4bGFiKCJFYXJsaWVzdCBtaWNyb2V4b24gZGlmZmVyZW50aWFsIGluY2x1c2lvbiBkZXRlY3Rpb24gcGVyIGdlbmUgKERQQykiKSsKICB5bGFiKCJQcm90ZWluIHR5cGUgcHJvcG9ydGlvbiIpICsKICBzY2FsZV94X2NvbnRpbnVvdXMoYnJlYWtzPXNlcSgxMC41LDE2LjUsMSkpICsKICBzY2FsZV9maWxsX2Rpc2NyZXRlKG5hbWUgPSAiUHJvdGVpbiB0eXBlIiwgbGFiZWxzID0gYygiTm9uLWNlbnRyYWwiLCAiQ2VudHJhbCIpKSAgKwogICAgICAgICAgICB0aGVtZShheGlzLnRleHQueCA9IGVsZW1lbnRfdGV4dChhbmdsZSA9IDkwKSwgbGVnZW5kLnBvc2l0aW9uID0gInRvcCIsIGxlZ2VuZC5kaXJlY3Rpb24gPSAiaG9yaXpvbnRhbCIpCgoKCgpQUElfZGlmZl9hZ2VfSE5NX3N0YXRzX0hDIDwtIFBQSV9kaWZmX2FnZV9ITk1GW2lzLm5hKGRpZmZfYWdlLngpIT1UUlVFXVssICAuKG1pbl9kaWZfYWdlPW1pbihkaWZmX2FnZS54KSwgTnVtYmVyX29mX01FPS5OKSwgYnk9YygiZW5zZW1ibF9nZW5lX2lkIiwgImhhcm1vbmljX2NlbnRyYWxpdHkiKSBdClBQSV9kaWZmX2FnZV9ITk1fc3RhdHNfSENbLCByYW5rOj1mcmFuaygtaGFybW9uaWNfY2VudHJhbGl0eSldClBQSV9kaWZmX2FnZV9ITk1fc3RhdHNfSENbICwgbWF4X3Jhbms6PW1heChyYW5rKV0KUFBJX2RpZmZfYWdlX0hOTV9zdGF0c19IQ1sgLCBwZXJjZW50aWw6PXJhbmsvbWF4X3JhbmsqMTAwXQpQUElfZGlmZl9hZ2VfSE5NX3N0YXRzX0hDWywgY2VudHJhbDo9RkFMU0VdClBQSV9kaWZmX2FnZV9ITk1fc3RhdHNfSENbcGVyY2VudGlsPDE1LCBjZW50cmFsOj1UUlVFXQoKCgpnZ3Bsb3QoUFBJX2RpZmZfYWdlX0hOTV9zdGF0c19IQykgKwogIGdlb21fYmFyKGFlcyhtaW5fZGlmX2FnZSwgZmlsbD1jZW50cmFsKSwgc3RhdCA9ICJjb3VudCIsIHBvc2l0aW9uPSJmaWxsIiApICsKICB4bGFiKCJFYXJsaWVzdCBtaWNyb2V4b24gZGlmZmVyZW50aWFsIGluY2x1c2lvbiBkZXRlY3Rpb24gcGVyIGdlbmUgKERQQykiKSsKICB5bGFiKCJQcm90ZWluIHR5cGUgcHJvcG9ydGlvbiIpICsKICBzY2FsZV94X2NvbnRpbnVvdXMoYnJlYWtzPXNlcSgxMC41LDE2LjUsMSkpICsKICBzY2FsZV9maWxsX2Rpc2NyZXRlKG5hbWUgPSAiUHJvdGVpbiB0eXBlIiwgbGFiZWxzID0gYygiTm9uLWNlbnRyYWwiLCAiQ2VudHJhbCIpKSAgKwogICAgICAgICAgICB0aGVtZShheGlzLnRleHQueCA9IGVsZW1lbnRfdGV4dChhbmdsZSA9IDkwKSwgbGVnZW5kLnBvc2l0aW9uID0gInRvcCIsIGxlZ2VuZC5kaXJlY3Rpb24gPSAiaG9yaXpvbnRhbCIpCgoKCgpjZW50cmFsaXR5X2xvZ2lzdGljX3JlZ3Jlc3Npb24gPC0gZ2xtKGZvcm11bGEgPSBjZW50cmFsIH4gbWluX2RpZl9hZ2UsIGZhbWlseSA9ICJiaW5vbWlhbCIsIGRhdGEgPSBQUElfZGlmZl9hZ2VfSE5NX3N0YXRzX0VDKQpzdW1tYXJ5KGNlbnRyYWxpdHlfbG9naXN0aWNfcmVncmVzc2lvbikKCgpjZW50cmFsaXR5X2xvZ2lzdGljX3JlZ3Jlc3Npb24gPC0gZ2xtKGZvcm11bGEgPSBjZW50cmFsIH4gbWluX2RpZl9hZ2UsIGZhbWlseSA9ICJiaW5vbWlhbCIsIGRhdGEgPSBQUElfZGlmZl9hZ2VfSE5NX3N0YXRzX0hDKQpzdW1tYXJ5KGNlbnRyYWxpdHlfbG9naXN0aWNfcmVncmVzc2lvbikKYGBgCgoKCgoKCgpgYGB7cn0KdmFyMnN0ciA8LSBmdW5jdGlvbih2MSkgewogIGRlcGFyc2Uoc3Vic3RpdHV0ZSh2MSkpCn0KCmBgYAoKCgoKCmBgYHtyfQojR09fUmVhY3RvbWUgPC0gZnJlYWQoIi4uL0ZpZ3VyZXMvTmV3X0ZpZ3VyZSA1L1NUUklORy9HTy9XaG9sZV9nZW5vbWVfYmFja2dyb3VuZC9SZWFjdG9tZSBQYXRod2F5cy50c3YiKQoKCgoKQXhvbl9ndWlkYW5jZSA8LSB1bmxpc3Qoc3Ryc3BsaXQoR09fUmVhY3RvbWVbYHRlcm0gZGVzY3JpcHRpb25gPT0iQXhvbiBndWlkYW5jZSJdJGBtYXRjaGluZyBwcm90ZWlucyBpbiB5b3VyIG5ldHdvcmsgKGxhYmVscylgLCAiLCIpKQpMMUNBTV9pbnRlcmFjdGlvbnMgPC0gdW5saXN0KHN0cnNwbGl0KEdPX1JlYWN0b21lW2B0ZXJtIGRlc2NyaXB0aW9uYD09IkwxQ0FNIGludGVyYWN0aW9ucyJdJGBtYXRjaGluZyBwcm90ZWlucyBpbiB5b3VyIG5ldHdvcmsgKGxhYmVscylgLCAiLCIpKQpQcm90ZWluX3Byb3RlaW5faW50ZXJhY3Rpb25zX2F0X3N5bmFwc2VzIDwtIHVubGlzdChzdHJzcGxpdChHT19SZWFjdG9tZVtgdGVybSBkZXNjcmlwdGlvbmA9PSJQcm90ZWluLXByb3RlaW4gaW50ZXJhY3Rpb25zIGF0IHN5bmFwc2VzIl0kYG1hdGNoaW5nIHByb3RlaW5zIGluIHlvdXIgbmV0d29yayAobGFiZWxzKWAsICIsIikpCkVSX3RvX0dvbGdpX0FudGVyb2dyYWRlX1RyYW5zcG9ydCA8LSB1bmxpc3Qoc3Ryc3BsaXQoR09fUmVhY3RvbWVbYHRlcm0gZGVzY3JpcHRpb25gPT0iRVIgdG8gR29sZ2kgQW50ZXJvZ3JhZGUgVHJhbnNwb3J0Il0kYG1hdGNoaW5nIHByb3RlaW5zIGluIHlvdXIgbmV0d29yayAobGFiZWxzKWAsICIsIikpCkNsYXRocmluX21lZGlhdGVkX2VuZG9jeXRvc2lzIDwtIHVubGlzdChzdHJzcGxpdChHT19SZWFjdG9tZVtgdGVybSBkZXNjcmlwdGlvbmA9PSJDbGF0aHJpbi1tZWRpYXRlZCBlbmRvY3l0b3NpcyJdJGBtYXRjaGluZyBwcm90ZWlucyBpbiB5b3VyIG5ldHdvcmsgKGxhYmVscylgLCAiLCIpKQpHb2xnaV9Bc3NvY2lhdGVkX1Zlc2ljbGVfQmlvZ2VuZXNpcyA8LSB1bmxpc3Qoc3Ryc3BsaXQoR09fUmVhY3RvbWVbYHRlcm0gZGVzY3JpcHRpb25gPT0iR29sZ2kgQXNzb2NpYXRlZCBWZXNpY2xlIEJpb2dlbmVzaXMiXSRgbWF0Y2hpbmcgcHJvdGVpbnMgaW4geW91ciBuZXR3b3JrIChsYWJlbHMpYCwgIiwiKSkKTWVtYnJhbmVfVHJhZmZpY2tpbmcgPC0gdW5saXN0KHN0cnNwbGl0KEdPX1JlYWN0b21lW2B0ZXJtIGRlc2NyaXB0aW9uYD09Ik1lbWJyYW5lIFRyYWZmaWNraW5nIl0kYG1hdGNoaW5nIHByb3RlaW5zIGluIHlvdXIgbmV0d29yayAobGFiZWxzKWAsICIsIikpCkludHJhX0dvbGdpX2FuZF9yZXRyb2dyYWRlX0dvbGdpX3RvX0VSX3RyYWZmaWMgPC0gdW5saXN0KHN0cnNwbGl0KEdPX1JlYWN0b21lW2B0ZXJtIGRlc2NyaXB0aW9uYD09IkludHJhLUdvbGdpIGFuZCByZXRyb2dyYWRlIEdvbGdpLXRvLUVSIHRyYWZmaWMiXSRgbWF0Y2hpbmcgcHJvdGVpbnMgaW4geW91ciBuZXR3b3JrIChsYWJlbHMpYCwgIiwiKSkKCkdPcyA8LSBsaXN0KEF4b25fZ3VpZGFuY2UsIEwxQ0FNX2ludGVyYWN0aW9ucykKClBQSV9jZW50cmFsaXR5X0dPIDwtIFBQSV9kaWZmX2FnZV9ITk1GWywgYygibWFwcGVkX2dlbmUiLCAiZXhvbl9JRCIsICJkaWZmX2FnZSIsICAiYmV0d2Vlbm5lc3MiLCAiZWlnZW5fY2VudHJhbGl0eSIsICJoYXJtb25pY19jZW50cmFsaXR5IiwgImRlZ3JlZV9jZW50cmFsaXR5IiwgInR5cGUiKSBdCgpQUElfY2VudHJhbGl0eV9HTyA8LQogIHJiaW5kKFBQSV9kaWZmX2FnZV9ITk1GWyBtYXBwZWRfZ2VuZSAlaW4lIEF4b25fZ3VpZGFuY2UsIC4obWFwcGVkX2dlbmUsIGV4b25fSUQsIGRpZmZfYWdlLCAgYmV0d2Vlbm5lc3MsIGVpZ2VuX2NlbnRyYWxpdHksIGhhcm1vbmljX2NlbnRyYWxpdHksIGRlZ3JlZV9jZW50cmFsaXR5LCB0eXBlLCBHTz12YXIyc3RyKEF4b25fZ3VpZGFuY2UpKSBdLApQUElfZGlmZl9hZ2VfSE5NRlsgbWFwcGVkX2dlbmUgJWluJSBMMUNBTV9pbnRlcmFjdGlvbnMsIC4obWFwcGVkX2dlbmUsIGV4b25fSUQsIGRpZmZfYWdlLCAgYmV0d2Vlbm5lc3MsIGVpZ2VuX2NlbnRyYWxpdHksIGhhcm1vbmljX2NlbnRyYWxpdHksIGRlZ3JlZV9jZW50cmFsaXR5LCB0eXBlLCBHTz12YXIyc3RyKEwxQ0FNX2ludGVyYWN0aW9ucykpIF0sClBQSV9kaWZmX2FnZV9ITk1GWyBtYXBwZWRfZ2VuZSAlaW4lIFByb3RlaW5fcHJvdGVpbl9pbnRlcmFjdGlvbnNfYXRfc3luYXBzZXMsIC4obWFwcGVkX2dlbmUsIGV4b25fSUQsIGRpZmZfYWdlLCAgYmV0d2Vlbm5lc3MsIGVpZ2VuX2NlbnRyYWxpdHksIGhhcm1vbmljX2NlbnRyYWxpdHksIGRlZ3JlZV9jZW50cmFsaXR5LCB0eXBlLCBHTz12YXIyc3RyKFByb3RlaW5fcHJvdGVpbl9pbnRlcmFjdGlvbnNfYXRfc3luYXBzZXMpKSBdLApQUElfZGlmZl9hZ2VfSE5NRlsgbWFwcGVkX2dlbmUgJWluJSBFUl90b19Hb2xnaV9BbnRlcm9ncmFkZV9UcmFuc3BvcnQsIC4obWFwcGVkX2dlbmUsIGV4b25fSUQsIGRpZmZfYWdlLCAgYmV0d2Vlbm5lc3MsIGVpZ2VuX2NlbnRyYWxpdHksIGhhcm1vbmljX2NlbnRyYWxpdHksIGRlZ3JlZV9jZW50cmFsaXR5LCB0eXBlLCBHTz12YXIyc3RyKEVSX3RvX0dvbGdpX0FudGVyb2dyYWRlX1RyYW5zcG9ydCkpIF0sClBQSV9kaWZmX2FnZV9ITk1GWyBtYXBwZWRfZ2VuZSAlaW4lIENsYXRocmluX21lZGlhdGVkX2VuZG9jeXRvc2lzLCAuKG1hcHBlZF9nZW5lLCBleG9uX0lELCBkaWZmX2FnZSwgIGJldHdlZW5uZXNzLCBlaWdlbl9jZW50cmFsaXR5LCBoYXJtb25pY19jZW50cmFsaXR5LCBkZWdyZWVfY2VudHJhbGl0eSwgdHlwZSwgR089dmFyMnN0cihDbGF0aHJpbl9tZWRpYXRlZF9lbmRvY3l0b3NpcykpIF0sClBQSV9kaWZmX2FnZV9ITk1GWyBtYXBwZWRfZ2VuZSAlaW4lIEdvbGdpX0Fzc29jaWF0ZWRfVmVzaWNsZV9CaW9nZW5lc2lzLCAuKG1hcHBlZF9nZW5lLCBleG9uX0lELCBkaWZmX2FnZSwgIGJldHdlZW5uZXNzLCBlaWdlbl9jZW50cmFsaXR5LCBoYXJtb25pY19jZW50cmFsaXR5LCBkZWdyZWVfY2VudHJhbGl0eSwgdHlwZSwgR089dmFyMnN0cihHb2xnaV9Bc3NvY2lhdGVkX1Zlc2ljbGVfQmlvZ2VuZXNpcykpIF0sClBQSV9kaWZmX2FnZV9ITk1GWyBtYXBwZWRfZ2VuZSAlaW4lIE1lbWJyYW5lX1RyYWZmaWNraW5nLCAuKG1hcHBlZF9nZW5lLCBleG9uX0lELCBkaWZmX2FnZSwgIGJldHdlZW5uZXNzLCBlaWdlbl9jZW50cmFsaXR5LCBoYXJtb25pY19jZW50cmFsaXR5LCBkZWdyZWVfY2VudHJhbGl0eSwgdHlwZSwgR089dmFyMnN0cihNZW1icmFuZV9UcmFmZmlja2luZykpIF0sClBQSV9kaWZmX2FnZV9ITk1GWyBtYXBwZWRfZ2VuZSAlaW4lIEludHJhX0dvbGdpX2FuZF9yZXRyb2dyYWRlX0dvbGdpX3RvX0VSX3RyYWZmaWMsIC4obWFwcGVkX2dlbmUsIGV4b25fSUQsIGRpZmZfYWdlLCAgYmV0d2Vlbm5lc3MsIGVpZ2VuX2NlbnRyYWxpdHksIGhhcm1vbmljX2NlbnRyYWxpdHksIGRlZ3JlZV9jZW50cmFsaXR5LCB0eXBlLCBHTz12YXIyc3RyKEludHJhX0dvbGdpX2FuZF9yZXRyb2dyYWRlX0dvbGdpX3RvX0VSX3RyYWZmaWMpKSBdKQoKCgoKCgoKZ2dwbG90KFBQSV9jZW50cmFsaXR5X0dPLCBhZXMoR08sIGxvZyhiZXR3ZWVubmVzcykpKSArCiAgZ2VvbV9qaXR0ZXIoKSArCiAgZ2VvbV9ib3hwbG90KCkgKwogIHRoZW1lKGF4aXMudGV4dC54ID0gZWxlbWVudF90ZXh0KGFuZ2xlID0gOTAsIHZqdXN0ID0gMCkpCgoKUFBJX2NlbnRyYWxpdHlfR09fZ2VuZV9jZW50ZXJlZCA8LSBQUElfY2VudHJhbGl0eV9HT1sgLCAuKGRpZmZfYWdlPW1pbihkaWZmX2FnZSkpICwgYnk9YygibWFwcGVkX2dlbmUiLCAiYmV0d2Vlbm5lc3MiLCAiZWlnZW5fY2VudHJhbGl0eSIsICJoYXJtb25pY19jZW50cmFsaXR5IiwgImRlZ3JlZV9jZW50cmFsaXR5IiwgIkdPIildCgoKZ2dwbG90KFBQSV9jZW50cmFsaXR5X0dPX2dlbmVfY2VudGVyZWQgKSArCiAgZ2VvbV9qaXR0ZXIoYWVzKGhhcm1vbmljX2NlbnRyYWxpdHksIGRpZmZfYWdlLCBjb2xvcj1HTyksIHdpZHRoPTAuNSwgaGVpZ2h0PTAuMSApICsKICAgdGhlbWUoYXhpcy50ZXh0LnggPSBlbGVtZW50X3RleHQoYW5nbGUgPSA5MCksIGxlZ2VuZC5wb3NpdGlvbiA9ICJ0b3AiLCBsZWdlbmQuZGlyZWN0aW9uID0gImhvcml6b250YWwiKQoKYGBgCgpgYGB7cn0KZ2dwbG90KFBQSV9jZW50cmFsaXR5X0dPX2dlbmVfY2VudGVyZWQsIGFlcyhHTywgZGlmZl9hZ2UpKSArCisgICAgIGdlb21faml0dGVyKGhlaWdodCA9IDAuMSwgd2lkdGggPSAwLjEpICsKKyAgICAgdGhlbWUoYXhpcy50ZXh0LnggPSBlbGVtZW50X3RleHQoYW5nbGUgPSA5MCwgdmp1c3QgPSAwKSkKCgpgYGAKCgpgYGB7cn0KClBQSV9jZW50cmFsaXR5X0dPX2dlbmVfY2VudGVyZWRfc3RhdHMgPC0gIFBQSV9jZW50cmFsaXR5X0dPX2dlbmVfY2VudGVyZWRbICwgLihOPS5OKSwgIGJ5PWMoIkdPIiwgImRpZmZfYWdlIildCgpQUElfY2VudHJhbGl0eV9HT19nZW5lX2NlbnRlcmVkX3N0YXRzWyAsIFRvdGFsOj1zdW0oTiksIGJ5ID0iR08iXQoKUFBJX2NlbnRyYWxpdHlfR09fZ2VuZV9jZW50ZXJlZF9zdGF0c1ssIGZyYWN0aW9uOj1OL1RvdGFsIF0KCmRpZmZfYWdlX2xldmVscyA8LSBQUElfY2VudHJhbGl0eV9HT19nZW5lX2NlbnRlcmVkX3N0YXRzWyAsIC4obWVhbj1tZWFuKGRpZmZfYWdlKSksIGJ5PSJHTyJdW29yZGVyKC1tZWFuKV0kR08KCgpQUElfY2VudHJhbGl0eV9HT19nZW5lX2NlbnRlcmVkX3N0YXRzJEdPIDwtIGZhY3RvcihQUElfY2VudHJhbGl0eV9HT19nZW5lX2NlbnRlcmVkX3N0YXRzJEdPLCBsZXZlbHM9IGRpZmZfYWdlX2xldmVscykKCmdncGxvdChQUElfY2VudHJhbGl0eV9HT19nZW5lX2NlbnRlcmVkX3N0YXRzKSArCiAgZ2VvbV90aWxlKGFlcyggZGlmZl9hZ2UsIEdPLCBmaWxsID0gZnJhY3Rpb24pKSArCiAgc2NhbGVfZmlsbF9ncmFkaWVudChsb3c9ImdyZXkiLCBoaWdoPSJyZWQiKSsKICB0aGVtZV9idygpKwogIGxhYnMoZmlsbCA9ICJHZW5lIGZyYWN0aW9uIikgKwogIHRoZW1lKGF4aXMudGV4dC54ID0gZWxlbWVudF90ZXh0KGFuZ2xlID0gOTAsIHZqdXN0ID0gMCksCiAgICBheGlzLnRpdGxlID0gZWxlbWVudF90ZXh0KGNvbG91ciA9IE5BKSwKICAgIHN0cmlwLnRleHQgPSBlbGVtZW50X3RleHQoaGp1c3QgPSAwLjUpKQpgYGAKCgoKYGBge3J9CgpHT19SZWFjdG9tZV9hbGwgPC0gcmJpbmQoCkRlbHRhX0hOTUZfd2hpcHBldF9NRV9hZ2VfZGlmZl90b3RhbF9QUElbIG1hcHBlZF9nZW5lICVpbiUgQXhvbl9ndWlkYW5jZSwgLihtYXBwZWRfZ2VuZSwgZXhvbl9JRCwgZGlmZl9hZ2UsIEdPPXZhcjJzdHIoQXhvbl9ndWlkYW5jZSkpIF0gLApEZWx0YV9ITk1GX3doaXBwZXRfTUVfYWdlX2RpZmZfdG90YWxfUFBJWyBtYXBwZWRfZ2VuZSAlaW4lIEwxQ0FNX2ludGVyYWN0aW9ucywgLihtYXBwZWRfZ2VuZSwgZXhvbl9JRCwgZGlmZl9hZ2UsIEdPPXZhcjJzdHIoTDFDQU1faW50ZXJhY3Rpb25zKSkgXSwKRGVsdGFfSE5NRl93aGlwcGV0X01FX2FnZV9kaWZmX3RvdGFsX1BQSVsgbWFwcGVkX2dlbmUgJWluJSBQcm90ZWluX3Byb3RlaW5faW50ZXJhY3Rpb25zX2F0X3N5bmFwc2VzLCAuKG1hcHBlZF9nZW5lLCBleG9uX0lELCBkaWZmX2FnZSwgR089dmFyMnN0cihQcm90ZWluX3Byb3RlaW5faW50ZXJhY3Rpb25zX2F0X3N5bmFwc2VzKSkgXSwKRGVsdGFfSE5NRl93aGlwcGV0X01FX2FnZV9kaWZmX3RvdGFsX1BQSVsgbWFwcGVkX2dlbmUgJWluJSBFUl90b19Hb2xnaV9BbnRlcm9ncmFkZV9UcmFuc3BvcnQsIC4obWFwcGVkX2dlbmUsIGV4b25fSUQsIGRpZmZfYWdlLCBHTz12YXIyc3RyKEVSX3RvX0dvbGdpX0FudGVyb2dyYWRlX1RyYW5zcG9ydCkpIF0sCkRlbHRhX0hOTUZfd2hpcHBldF9NRV9hZ2VfZGlmZl90b3RhbF9QUElbIG1hcHBlZF9nZW5lICVpbiUgQ2xhdGhyaW5fbWVkaWF0ZWRfZW5kb2N5dG9zaXMsIC4obWFwcGVkX2dlbmUsIGV4b25fSUQsIGRpZmZfYWdlLCBHTz12YXIyc3RyKENsYXRocmluX21lZGlhdGVkX2VuZG9jeXRvc2lzKSkgXSwKRGVsdGFfSE5NRl93aGlwcGV0X01FX2FnZV9kaWZmX3RvdGFsX1BQSVsgbWFwcGVkX2dlbmUgJWluJSBHb2xnaV9Bc3NvY2lhdGVkX1Zlc2ljbGVfQmlvZ2VuZXNpcywgLihtYXBwZWRfZ2VuZSwgZXhvbl9JRCwgZGlmZl9hZ2UsIEdPPXZhcjJzdHIoR29sZ2lfQXNzb2NpYXRlZF9WZXNpY2xlX0Jpb2dlbmVzaXMpKSBdLApEZWx0YV9ITk1GX3doaXBwZXRfTUVfYWdlX2RpZmZfdG90YWxfUFBJWyBtYXBwZWRfZ2VuZSAlaW4lIE1lbWJyYW5lX1RyYWZmaWNraW5nLCAuKG1hcHBlZF9nZW5lLCBleG9uX0lELCBkaWZmX2FnZSwgR089dmFyMnN0cihNZW1icmFuZV9UcmFmZmlja2luZykpIF0sCkRlbHRhX0hOTUZfd2hpcHBldF9NRV9hZ2VfZGlmZl90b3RhbF9QUElbIG1hcHBlZF9nZW5lICVpbiUgSW50cmFfR29sZ2lfYW5kX3JldHJvZ3JhZGVfR29sZ2lfdG9fRVJfdHJhZmZpYywgLihtYXBwZWRfZ2VuZSwgZXhvbl9JRCwgZGlmZl9hZ2UsIEdPPXZhcjJzdHIoSW50cmFfR29sZ2lfYW5kX3JldHJvZ3JhZGVfR29sZ2lfdG9fRVJfdHJhZmZpYykpXSApCgoKR09fUmVhY3RvbWVfYWxsX3N0YXRzIDwtICBHT19SZWFjdG9tZV9hbGxbICwgLihOPS5OKSwgIGJ5PWMoIkdPIiwgImRpZmZfYWdlIildCkdPX1JlYWN0b21lX2FsbF9zdGF0c1sgLCBUb3RhbDo9c3VtKE4pLCBieSA9IkdPIl0KR09fUmVhY3RvbWVfYWxsX3N0YXRzWywgZnJhY3Rpb246PU4vVG90YWwgXQpkaWZmX2FnZV9sZXZlbHMgPC0gR09fUmVhY3RvbWVfYWxsX3N0YXRzWyAsIC4obWVhbj1tZWFuKGRpZmZfYWdlKSksIGJ5PSJHTyJdW29yZGVyKC1tZWFuKV0kR08KCmRpZmZfYWdlX2xldmVscyA8LSBjKCJNZW1icmFuZV9UcmFmZmlja2luZyIsCiAgICAgICAgICAgICAgICAgICAgICJJbnRyYV9Hb2xnaV9hbmRfcmV0cm9ncmFkZV9Hb2xnaV90b19FUl90cmFmZmljIiwKICAgICAgICAgICAgICAgICAgICAgIkdvbGdpX0Fzc29jaWF0ZWRfVmVzaWNsZV9CaW9nZW5lc2lzIiwKICAgICAgICAgICAgICAgICAgICAgIkNsYXRocmluX21lZGlhdGVkX2VuZG9jeXRvc2lzIiwKICAgICAgICAgICAgICAgICAgICAgIkVSX3RvX0dvbGdpX0FudGVyb2dyYWRlX1RyYW5zcG9ydCIsCiAgICAgICAgICAgICAgICAgICAgICAiQXhvbl9ndWlkYW5jZSIsCiAgICAgICAgICAgICAgICAgICAgICAiTDFDQU1faW50ZXJhY3Rpb25zIiwKICAgICAgICAgICAgICAgICAgICAgICJQcm90ZWluX3Byb3RlaW5faW50ZXJhY3Rpb25zX2F0X3N5bmFwc2VzIikKCgpHT19SZWFjdG9tZV9hbGxfc3RhdHMkR08gPC0gZmFjdG9yKEdPX1JlYWN0b21lX2FsbF9zdGF0cyRHTywgbGV2ZWxzPSByZXYoZGlmZl9hZ2VfbGV2ZWxzKSkKCgpGNkcgPC0gZ2dwbG90KEdPX1JlYWN0b21lX2FsbF9zdGF0cykgKwogIGdlb21fdGlsZShhZXMoIGRpZmZfYWdlLCBHTywgZmlsbCA9IGZyYWN0aW9uKSkgKwogIGdlb21fdGV4dChhZXMoZGlmZl9hZ2UsIEdPLCBsYWJlbCA9IE4pLCBjb2xvcj0id2hpdGUiKSArCiAgc2NhbGVfZmlsbF9ncmFkaWVudChsb3c9ImdyZXkiLCBoaWdoPSJyZWQiLCBsaW1pdHM9YygwLCAwLjcpICkrCiAgICBzY2FsZV94X2NvbnRpbnVvdXMoYnJlYWtzPXNlcSgxMC41LDE2LjUsMSkpICsKICB0aGVtZV9idygpKwogIHhsYWIoIkRQQyIpICsKICB5bGFiKCIiKSArCiAgbGFicyhmaWxsID0gIkdlbmUgZnJhY3Rpb24iKSArCiAgdGhlbWUoYXhpcy50ZXh0LnggPSBlbGVtZW50X3RleHQoYW5nbGUgPSA5MCksIGxlZ2VuZC5wb3NpdGlvbiA9ICJ0b3AiLCBsZWdlbmQuZGlyZWN0aW9uID0gImhvcml6b250YWwiKQoKRjZHCgpgYGAKCgpgYGB7ciwgZmlnLmhlaWdodD04LCBmaWcud2lkdGg9NX0KRjZGRyA8LSBwbG90X2dyaWQoRjZGLCBGNkcsIG5jb2w9MSwgbGFiZWxzID0gYygiRiIsICJHIiksIHJlbF9oZWlnaHRzID0gYygxLjMsIDEpKQpgYGAKCgoKYGBge3J9CgoKQmlvbG9naWNhbF9Qcm9jZXNzIDwtIGZyZWFkKCIuLi9QUEkvZW5yaWNobWVudC5Qcm9jZXNzLnRzdiIpCkNlbGx1bGFyX0NvbXBvbmVudCA8LSBmcmVhZCgiLi4vUFBJL2VucmljaG1lbnQuQ29tcG9uZW50LnRzdiIpCkdPX1JlYWN0b21lIDwtIGZyZWFkKCIuLi9QUEkvZW5yaWNobWVudC5SQ1RNLnRzdiIpCgoKbmVydm91c19zeXN0ZW1fZGV2ZWxvcG1lbnQgPC0gdW5saXN0KHN0cnNwbGl0KEJpb2xvZ2ljYWxfUHJvY2Vzc1tgdGVybSBkZXNjcmlwdGlvbmA9PSJuZXJ2b3VzIHN5c3RlbSBkZXZlbG9wbWVudCJdJGBtYXRjaGluZyBwcm90ZWlucyBpbiB5b3VyIG5ldHdvcmsgKGxhYmVscylgLCAiLCIpKQp2ZXNpY2xlX21lZGlhdGVkX3RyYW5zcG9ydCA8LSB1bmxpc3Qoc3Ryc3BsaXQoQmlvbG9naWNhbF9Qcm9jZXNzW2B0ZXJtIGRlc2NyaXB0aW9uYD09InZlc2ljbGUtbWVkaWF0ZWQgdHJhbnNwb3J0Il0kYG1hdGNoaW5nIHByb3RlaW5zIGluIHlvdXIgbmV0d29yayAobGFiZWxzKWAsICIsIikpCmN5dG9za2VsZXRvbl9vcmdhbml6YXRpb24gPC0gdW5saXN0KHN0cnNwbGl0KEJpb2xvZ2ljYWxfUHJvY2Vzc1tgdGVybSBkZXNjcmlwdGlvbmA9PSJjeXRvc2tlbGV0b24gb3JnYW5pemF0aW9uIl0kYG1hdGNoaW5nIHByb3RlaW5zIGluIHlvdXIgbmV0d29yayAobGFiZWxzKWAsICIsIikpCnN5bmFwc2VfYXNzZW1ibHkgPC0gdW5saXN0KHN0cnNwbGl0KEJpb2xvZ2ljYWxfUHJvY2Vzc1tgdGVybSBkZXNjcmlwdGlvbmA9PSJzeW5hcHNlIGFzc2VtYmx5Il0kYG1hdGNoaW5nIHByb3RlaW5zIGluIHlvdXIgbmV0d29yayAobGFiZWxzKWAsICIsIikpCnNpZ25hbF90cmFuc2R1Y3Rpb24gPC0gdW5saXN0KHN0cnNwbGl0KEJpb2xvZ2ljYWxfUHJvY2Vzc1tgdGVybSBkZXNjcmlwdGlvbmA9PSJzaWduYWwgdHJhbnNkdWN0aW9uIl0kYG1hdGNoaW5nIHByb3RlaW5zIGluIHlvdXIgbmV0d29yayAobGFiZWxzKWAsICIsIikpCgpzeW5hcHNlIDwtIHVubGlzdChzdHJzcGxpdChDZWxsdWxhcl9Db21wb25lbnRbYHRlcm0gZGVzY3JpcHRpb25gPT0ic3luYXBzZSJdJGBtYXRjaGluZyBwcm90ZWlucyBpbiB5b3VyIG5ldHdvcmsgKGxhYmVscylgLCAiLCIpKQpzb21hdG9kZW5kcml0aWNfY29tcGFydG1lbnQgPC0gdW5saXN0KHN0cnNwbGl0KENlbGx1bGFyX0NvbXBvbmVudFtgdGVybSBkZXNjcmlwdGlvbmA9PSJzb21hdG9kZW5kcml0aWMgY29tcGFydG1lbnQiXSRgbWF0Y2hpbmcgcHJvdGVpbnMgaW4geW91ciBuZXR3b3JrIChsYWJlbHMpYCwgIiwiKSkKcG9zdHN5bmFwc2UgPC0gdW5saXN0KHN0cnNwbGl0KENlbGx1bGFyX0NvbXBvbmVudFtgdGVybSBkZXNjcmlwdGlvbmA9PSJwb3N0c3luYXBzZSJdJGBtYXRjaGluZyBwcm90ZWlucyBpbiB5b3VyIG5ldHdvcmsgKGxhYmVscylgLCAiLCIpKQpwcmVzeW5hcHNlIDwtIHVubGlzdChzdHJzcGxpdChDZWxsdWxhcl9Db21wb25lbnRbYHRlcm0gZGVzY3JpcHRpb25gPT0icHJlc3luYXBzZSJdJGBtYXRjaGluZyBwcm90ZWlucyBpbiB5b3VyIG5ldHdvcmsgKGxhYmVscylgLCAiLCIpKQpncm93dGhfY29uZSA8LSB1bmxpc3Qoc3Ryc3BsaXQoQ2VsbHVsYXJfQ29tcG9uZW50W2B0ZXJtIGRlc2NyaXB0aW9uYD09Imdyb3d0aCBjb25lIl0kYG1hdGNoaW5nIHByb3RlaW5zIGluIHlvdXIgbmV0d29yayAobGFiZWxzKWAsICIsIikpCgpHT19CaW9sb2dpY2FsX1Byb2Nlc3MgPC0gcmJpbmQoCkRlbHRhX0hOTUZfd2hpcHBldF9NRV9hZ2VfZGlmZl90b3RhbF9QUElbIG1hcHBlZF9nZW5lICVpbiUgbmVydm91c19zeXN0ZW1fZGV2ZWxvcG1lbnQsIC4obWFwcGVkX2dlbmUsIGV4b25fSUQsIGRpZmZfYWdlLCBHTz12YXIyc3RyKG5lcnZvdXNfc3lzdGVtX2RldmVsb3BtZW50KSkgXSwKRGVsdGFfSE5NRl93aGlwcGV0X01FX2FnZV9kaWZmX3RvdGFsX1BQSVsgbWFwcGVkX2dlbmUgJWluJSB2ZXNpY2xlX21lZGlhdGVkX3RyYW5zcG9ydCwgLihtYXBwZWRfZ2VuZSwgZXhvbl9JRCwgZGlmZl9hZ2UsIEdPPXZhcjJzdHIodmVzaWNsZV9tZWRpYXRlZF90cmFuc3BvcnQpKSBdLApEZWx0YV9ITk1GX3doaXBwZXRfTUVfYWdlX2RpZmZfdG90YWxfUFBJWyBtYXBwZWRfZ2VuZSAlaW4lIGN5dG9za2VsZXRvbl9vcmdhbml6YXRpb24sIC4obWFwcGVkX2dlbmUsIGV4b25fSUQsIGRpZmZfYWdlLCBHTz12YXIyc3RyKGN5dG9za2VsZXRvbl9vcmdhbml6YXRpb24pKSBdLApEZWx0YV9ITk1GX3doaXBwZXRfTUVfYWdlX2RpZmZfdG90YWxfUFBJWyBtYXBwZWRfZ2VuZSAlaW4lIHN5bmFwc2VfYXNzZW1ibHksIC4obWFwcGVkX2dlbmUsIGV4b25fSUQsIGRpZmZfYWdlLCBHTz12YXIyc3RyKHN5bmFwc2VfYXNzZW1ibHkpKSBdLApEZWx0YV9ITk1GX3doaXBwZXRfTUVfYWdlX2RpZmZfdG90YWxfUFBJWyBtYXBwZWRfZ2VuZSAlaW4lIHNpZ25hbF90cmFuc2R1Y3Rpb24sIC4obWFwcGVkX2dlbmUsIGV4b25fSUQsIGRpZmZfYWdlLCBHTz12YXIyc3RyKHNpZ25hbF90cmFuc2R1Y3Rpb24pKSBdICkKCkdPX0NlbGx1bGFyX0NvbXBvbmVudCA8LSByYmluZCgKRGVsdGFfSE5NRl93aGlwcGV0X01FX2FnZV9kaWZmX3RvdGFsX1BQSVsgbWFwcGVkX2dlbmUgJWluJSBzeW5hcHNlLCAuKG1hcHBlZF9nZW5lLCBleG9uX0lELCBkaWZmX2FnZSwgR089dmFyMnN0cihzeW5hcHNlKSkgXSwKRGVsdGFfSE5NRl93aGlwcGV0X01FX2FnZV9kaWZmX3RvdGFsX1BQSVsgbWFwcGVkX2dlbmUgJWluJSBzb21hdG9kZW5kcml0aWNfY29tcGFydG1lbnQsIC4obWFwcGVkX2dlbmUsIGV4b25fSUQsIGRpZmZfYWdlLCBHTz12YXIyc3RyKHNvbWF0b2RlbmRyaXRpY19jb21wYXJ0bWVudCkpIF0sCkRlbHRhX0hOTUZfd2hpcHBldF9NRV9hZ2VfZGlmZl90b3RhbF9QUElbIG1hcHBlZF9nZW5lICVpbiUgcG9zdHN5bmFwc2UsIC4obWFwcGVkX2dlbmUsIGV4b25fSUQsIGRpZmZfYWdlLCBHTz12YXIyc3RyKHBvc3RzeW5hcHNlKSkgXSwKRGVsdGFfSE5NRl93aGlwcGV0X01FX2FnZV9kaWZmX3RvdGFsX1BQSVsgbWFwcGVkX2dlbmUgJWluJSBwcmVzeW5hcHNlLCAuKG1hcHBlZF9nZW5lLCBleG9uX0lELCBkaWZmX2FnZSwgR089dmFyMnN0cihwcmVzeW5hcHNlKSkgXSwKRGVsdGFfSE5NRl93aGlwcGV0X01FX2FnZV9kaWZmX3RvdGFsX1BQSVsgbWFwcGVkX2dlbmUgJWluJSBncm93dGhfY29uZSwgLihtYXBwZWRfZ2VuZSwgZXhvbl9JRCwgZGlmZl9hZ2UsIEdPPXZhcjJzdHIoZ3Jvd3RoX2NvbmUpKSBdICkKCgoKCkdPX0Jpb2xvZ2ljYWxfUHJvY2Vzc19zdGF0cyA8LSAgR09fQmlvbG9naWNhbF9Qcm9jZXNzWyAsIC4oTj0uTiksICBieT1jKCJHTyIsICJkaWZmX2FnZSIpXQpHT19CaW9sb2dpY2FsX1Byb2Nlc3Nfc3RhdHNbICwgVG90YWw6PXN1bShOKSwgYnkgPSJHTyJdCkdPX0Jpb2xvZ2ljYWxfUHJvY2Vzc19zdGF0c1ssIGZyYWN0aW9uOj1OL1RvdGFsIF0KZGlmZl9hZ2VfbGV2ZWxzIDwtIEdPX0Jpb2xvZ2ljYWxfUHJvY2Vzc19zdGF0c1sgLCAuKG1lYW49bWVkaWFuKGRpZmZfYWdlKSksIGJ5PSJHTyJdW29yZGVyKC1tZWFuKV0kR08KR09fQmlvbG9naWNhbF9Qcm9jZXNzX3N0YXRzJEdPIDwtIGZhY3RvcihHT19CaW9sb2dpY2FsX1Byb2Nlc3Nfc3RhdHMkR08sIGxldmVscz0gZGlmZl9hZ2VfbGV2ZWxzKQoKCgpTdXBfR09fdGltZS5BIDwtIGdncGxvdChHT19CaW9sb2dpY2FsX1Byb2Nlc3Nfc3RhdHMpICsKICBnZW9tX3RpbGUoYWVzKCBkaWZmX2FnZSwgR08sIGZpbGwgPSBmcmFjdGlvbikpICsKICAgIGdlb21fdGV4dChhZXMoZGlmZl9hZ2UsIEdPLCBsYWJlbCA9IE4pLCBjb2xvcj0id2hpdGUiKSArCiAgc2NhbGVfZmlsbF9ncmFkaWVudChsb3c9ImdyZXkiLCBoaWdoPSJyZWQiICkrCiAgdGhlbWVfYncoKSsKICBsYWJzKGZpbGwgPSAiR2VuZSBmcmFjdGlvbiIpICsKICB0aGVtZShheGlzLnRleHQueCA9IGVsZW1lbnRfdGV4dChhbmdsZSA9IDkwLCB2anVzdCA9IDApLAogICAgYXhpcy50aXRsZSA9IGVsZW1lbnRfdGV4dChjb2xvdXIgPSBOQSksCiAgICBzdHJpcC50ZXh0ID0gZWxlbWVudF90ZXh0KGhqdXN0ID0gMC41KSkKCgoKR09fQ2VsbHVsYXJfQ29tcG9uZW50X3N0YXRzIDwtICBHT19DZWxsdWxhcl9Db21wb25lbnRbICwgLihOPS5OKSwgIGJ5PWMoIkdPIiwgImRpZmZfYWdlIildCkdPX0NlbGx1bGFyX0NvbXBvbmVudF9zdGF0c1sgLCBUb3RhbDo9c3VtKE4pLCBieSA9IkdPIl0KR09fQ2VsbHVsYXJfQ29tcG9uZW50X3N0YXRzWywgZnJhY3Rpb246PU4vVG90YWwgXQpkaWZmX2FnZV9sZXZlbHMgPC0gR09fQ2VsbHVsYXJfQ29tcG9uZW50X3N0YXRzWyAsIC4obWVhbj1tZWRpYW4oZGlmZl9hZ2UpKSwgYnk9IkdPIl1bb3JkZXIoLW1lYW4pXSRHTwpHT19DZWxsdWxhcl9Db21wb25lbnRfc3RhdHMkR08gPC0gZmFjdG9yKEdPX0NlbGx1bGFyX0NvbXBvbmVudF9zdGF0cyRHTywgbGV2ZWxzPSBkaWZmX2FnZV9sZXZlbHMpCgoKClN1cF9HT190aW1lLkIgPC0gZ2dwbG90KEdPX0NlbGx1bGFyX0NvbXBvbmVudF9zdGF0cykgKwogIGdlb21fdGlsZShhZXMoIGRpZmZfYWdlLCBHTywgZmlsbCA9IGZyYWN0aW9uKSkgKwogIGdlb21fdGV4dChhZXMoZGlmZl9hZ2UsIEdPLCBsYWJlbCA9IE4pLCBjb2xvcj0id2hpdGUiKSArCiAgc2NhbGVfZmlsbF9ncmFkaWVudChsb3c9ImdyZXkiLCBoaWdoPSJyZWQiICkrCiAgdGhlbWVfYncoKSsKICBsYWJzKGZpbGwgPSAiR2VuZSBmcmFjdGlvbiIpICsKICB0aGVtZShheGlzLnRleHQueCA9IGVsZW1lbnRfdGV4dChhbmdsZSA9IDkwLCB2anVzdCA9IDApLAogICAgYXhpcy50aXRsZSA9IGVsZW1lbnRfdGV4dChjb2xvdXIgPSBOQSksCiAgICBzdHJpcC50ZXh0ID0gZWxlbWVudF90ZXh0KGhqdXN0ID0gMC41KSkKCgoKYGBgCgoKCmBgYHtyfQoKbGlicmFyeShnZ3B1YnIpCgoKR09fQmlvbG9naWNhbF9Qcm9jZXNzX2NlbnRyYWxpdHkgPC0gIG1lcmdlKEdPX0Jpb2xvZ2ljYWxfUHJvY2VzcywgIFBQSV9kaWZmX2FnZV9ITk1GWywgYygiZXhvbl9JRCIsICJtYXBwZWRfZ2VuZSIsICJiZXR3ZWVubmVzcyIsICJlaWdlbl9jZW50cmFsaXR5IiwgImhhcm1vbmljX2NlbnRyYWxpdHkiLCAiZGVncmVlX2NlbnRyYWxpdHkiLCAidHlwZSIpIF0sIGJ5PWMoImV4b25fSUQiLCAibWFwcGVkX2dlbmUiKSkKCgpHT19CaW9sb2dpY2FsX1Byb2Nlc3NfY2VudHJhbGl0eSA8LSB1bmlxdWUoR09fQmlvbG9naWNhbF9Qcm9jZXNzX2NlbnRyYWxpdHlbLCBjKCJtYXBwZWRfZ2VuZSIsICJHTyIsICJiZXR3ZWVubmVzcyIsICJlaWdlbl9jZW50cmFsaXR5IiwgImhhcm1vbmljX2NlbnRyYWxpdHkiLCAiZGVncmVlX2NlbnRyYWxpdHkiKV0pW2hhcm1vbmljX2NlbnRyYWxpdHk+MTBdICAjRmlsdGVybmluZyBub2RlcyB0aGF0IGFyZSBub3QgY29uZWN0ZWQgdG8gdGhlIG1haW4gbmV0d29yawoKCkdPX0Jpb2xvZ2ljYWxfUHJvY2Vzc19jZW50cmFsaXR5JEdPIDwtIGZhY3RvcihHT19CaW9sb2dpY2FsX1Byb2Nlc3NfY2VudHJhbGl0eSRHTywgIGxldmVscyA9IEdPX0Jpb2xvZ2ljYWxfUHJvY2Vzc19jZW50cmFsaXR5WyAsIC4obWVkaWFuPW1lZGlhbihoYXJtb25pY19jZW50cmFsaXR5KSkgLCBieT0iR08iIF1bb3JkZXIoLW1lZGlhbildJEdPKQoKCmdncGxvdCh1bmlxdWUoR09fQmlvbG9naWNhbF9Qcm9jZXNzX2NlbnRyYWxpdHlbLCBjKCJtYXBwZWRfZ2VuZSIsICJHTyIsICJiZXR3ZWVubmVzcyIsICJlaWdlbl9jZW50cmFsaXR5IiwgImhhcm1vbmljX2NlbnRyYWxpdHkiLCAiZGVncmVlX2NlbnRyYWxpdHkiKV0pW2hhcm1vbmljX2NlbnRyYWxpdHk+MTBdICkgICsKICBnZW9tX2JveHBsb3QoYWVzKEdPLCBoYXJtb25pY19jZW50cmFsaXR5ICApICkgKwogICAgeWxhYigiZWlnZW5jZW50cmFsaXR5IikgKwogICAgdGhlbWUoYXhpcy50ZXh0LnggPSBlbGVtZW50X3RleHQoYW5nbGUgPSA0NSwgdmp1c3QgPSAwLjYpLAogICAgYXhpcy50aXRsZS55ID0gZWxlbWVudF90ZXh0KGNvbG91ciA9IE5BKSwKICAgIHN0cmlwLnRleHQgPSBlbGVtZW50X3RleHQoaGp1c3QgPSAwLjUpKQoKCgpHT19CaW9sb2dpY2FsX1Byb2Nlc3NfY2VudHJhbGl0eSRHTyA8LSBmYWN0b3IoR09fQmlvbG9naWNhbF9Qcm9jZXNzX2NlbnRyYWxpdHkkR08sICBsZXZlbHMgPSBHT19CaW9sb2dpY2FsX1Byb2Nlc3NfY2VudHJhbGl0eVsgLCAuKG1lZGlhbj1tZWRpYW4oZWlnZW5fY2VudHJhbGl0eSkpICwgYnk9IkdPIiBdW29yZGVyKC1tZWRpYW4pXSRHTykKCgpnZ3Bsb3QoIEdPX0Jpb2xvZ2ljYWxfUHJvY2Vzc19jZW50cmFsaXR5ICkgICsKICBnZW9tX2JveHBsb3QoYWVzKEdPLCBlaWdlbl9jZW50cmFsaXR5ICApICkgKwogICAgeWxhYigiZWlnZW5jZW50cmFsaXR5IikgKwogICAgdGhlbWUoYXhpcy50ZXh0LnggPSBlbGVtZW50X3RleHQoYW5nbGUgPSA5MCwgdmp1c3QgPSAwLjYpLAogICAgYXhpcy50aXRsZS54ID0gZWxlbWVudF90ZXh0KGNvbG91ciA9IE5BKSwKICAgIHN0cmlwLnRleHQgPSBlbGVtZW50X3RleHQoaGp1c3QgPSAwLjUpKQoKR09fQmlvbG9naWNhbF9Qcm9jZXNzX2NlbnRyYWxpdHkkR08gPC0gZmFjdG9yKEdPX0Jpb2xvZ2ljYWxfUHJvY2Vzc19jZW50cmFsaXR5JEdPLCAgbGV2ZWxzID0gR09fQmlvbG9naWNhbF9Qcm9jZXNzX2NlbnRyYWxpdHlbICwgLihtZWRpYW49bWVkaWFuKGJldHdlZW5uZXNzKSkgLCBieT0iR08iIF1bb3JkZXIoLW1lZGlhbildJEdPKQoKCmdncGxvdCh1bmlxdWUoR09fQmlvbG9naWNhbF9Qcm9jZXNzX2NlbnRyYWxpdHlbLCBjKCJtYXBwZWRfZ2VuZSIsICJHTyIsICJiZXR3ZWVubmVzcyIsICJlaWdlbl9jZW50cmFsaXR5IiwgImhhcm1vbmljX2NlbnRyYWxpdHkiLCAiZGVncmVlX2NlbnRyYWxpdHkiKV0pW2hhcm1vbmljX2NlbnRyYWxpdHk+MTBdICkgICsKICBnZW9tX2JveHBsb3QoYWVzKEdPLCBsb2cxMChiZXR3ZWVubmVzcykpICAgKSArCiAgICB0aGVtZShheGlzLnRleHQueCA9IGVsZW1lbnRfdGV4dChhbmdsZSA9IDQ1LCB2anVzdCA9IDAuNiksCiAgICBheGlzLnRpdGxlID0gZWxlbWVudF90ZXh0KGNvbG91ciA9IE5BKSwKICAgIHN0cmlwLnRleHQgPSBlbGVtZW50X3RleHQoaGp1c3QgPSAwLjUpKQpgYGAKCgpgYGB7ciwgZmlnLmhlaWdodD0gNywgZmlnLndpZHRoPTV9CkdPX0Jpb2xvZ2ljYWxfUHJvY2Vzc19jZW50cmFsaXR5JEdPIDwtIGZhY3RvcihHT19CaW9sb2dpY2FsX1Byb2Nlc3NfY2VudHJhbGl0eSRHTywgIGxldmVscyA9IEdPX0Jpb2xvZ2ljYWxfUHJvY2Vzc19jZW50cmFsaXR5WyAsIC4obWVkaWFuPW1lZGlhbihlaWdlbl9jZW50cmFsaXR5KSkgLCBieT0iR08iIF1bb3JkZXIoLW1lZGlhbildJEdPKQoKCgoKCm15X2NvbXBhcmlzb25zIDwtIGxpc3QoYygidmVzaWNsZV9tZWRpYXRlZF90cmFuc3BvcnQiLCAiY3l0b3NrZWxldG9uX29yZ2FuaXphdGlvbiIgKSwKICAgICAgICAgICAgICAgICAgICAgICBjKCJ2ZXNpY2xlX21lZGlhdGVkX3RyYW5zcG9ydCIsICJzaWduYWxfdHJhbnNkdWN0aW9uIiApLAogICAgICAgICAgICAgICAgICAgICAgIGMoInZlc2ljbGVfbWVkaWF0ZWRfdHJhbnNwb3J0IiwgIm5lcnZvdXNfc3lzdGVtX2RldmVsb3BtZW50IiksCiAgICAgICAgICAgICAgICAgICAgICAgYygidmVzaWNsZV9tZWRpYXRlZF90cmFuc3BvcnQiLCAic3luYXBzZV9hc3NlbWJseSIgKSwKICAgICAgICAgICAgICAgICAgICAgICBjKCJjeXRvc2tlbGV0b25fb3JnYW5pemF0aW9uIiwgInNpZ25hbF90cmFuc2R1Y3Rpb24iICksCiAgICAgICAgICAgICAgICAgICAgICAgYygiY3l0b3NrZWxldG9uX29yZ2FuaXphdGlvbiIsICJuZXJ2b3VzX3N5c3RlbV9kZXZlbG9wbWVudCIgKSwKICAgICAgICAgICAgICAgICAgICAgICBjKCJjeXRvc2tlbGV0b25fb3JnYW5pemF0aW9uIiwgInN5bmFwc2VfYXNzZW1ibHkiKSwKICAgICAgICAgICAgICAgICAgICAgICBjKCJzaWduYWxfdHJhbnNkdWN0aW9uIiwgIm5lcnZvdXNfc3lzdGVtX2RldmVsb3BtZW50IiApLAogICAgICAgICAgICAgICAgICAgICAgIGMoInNpZ25hbF90cmFuc2R1Y3Rpb24iLCAibmVydm91c19zeXN0ZW1fZGV2ZWxvcG1lbnQiICksCiAgICAgICAgICAgICAgICAgICAgICAgYygibmVydm91c19zeXN0ZW1fZGV2ZWxvcG1lbnQiLCAic3luYXBzZV9hc3NlbWJseSIgKQogICAgICAgICAgICAgICAgICAgICAgICkKCgpnZ3Bsb3QoIEdPX0Jpb2xvZ2ljYWxfUHJvY2Vzc19jZW50cmFsaXR5LCBhZXMoR08sIGVpZ2VuX2NlbnRyYWxpdHkgICkgICkgICsKICBnZW9tX2JveHBsb3QoKSArCiAgICB5bGFiKCJlaWdlbmNlbnRyYWxpdHkiKSArCiAgICB0aGVtZShheGlzLnRleHQueCA9IGVsZW1lbnRfdGV4dChhbmdsZSA9IDkwLCB2anVzdCA9IDAuNiksCiAgICBheGlzLnRpdGxlLnggPSBlbGVtZW50X3RleHQoY29sb3VyID0gTkEpLAogICAgc3RyaXAudGV4dCA9IGVsZW1lbnRfdGV4dChoanVzdCA9IDAuNSkpICsKICBnZW9tX3NpZ25pZihjb21wYXJpc29ucyA9IG15X2NvbXBhcmlzb25zLCAKICAgICAgICAgICAgICBtYXBfc2lnbmlmX2xldmVsPVRSVUUgLCB5X3Bvc2l0aW9uID0gIHNlcSgwLjksIDEuOSwgMC4xKSkKCmBgYAoKCgoKYGBge3IsIGZpZy5oZWlnaHQ9IDcsIGZpZy53aWR0aD01fQpHT19CaW9sb2dpY2FsX1Byb2Nlc3NfY2VudHJhbGl0eSRHTyA8LSBmYWN0b3IoR09fQmlvbG9naWNhbF9Qcm9jZXNzX2NlbnRyYWxpdHkkR08sICBsZXZlbHMgPSBHT19CaW9sb2dpY2FsX1Byb2Nlc3NfY2VudHJhbGl0eVsgLCAuKG1lZGlhbj1tZWRpYW4oZWlnZW5fY2VudHJhbGl0eSkpICwgYnk9IkdPIiBdW29yZGVyKC1tZWRpYW4pXSRHTykKCgoKCgpteV9jb21wYXJpc29ucyA8LSBsaXN0KCNjKCJ2ZXNpY2xlX21lZGlhdGVkX3RyYW5zcG9ydCIsICJjeXRvc2tlbGV0b25fb3JnYW5pemF0aW9uIiApLAogICAgICAgICAgICAgICAgICAgICAgIGMoInZlc2ljbGVfbWVkaWF0ZWRfdHJhbnNwb3J0IiwgInNpZ25hbF90cmFuc2R1Y3Rpb24iICksCiAgICAgICAgICAgICAgICAgICAgICAgYygidmVzaWNsZV9tZWRpYXRlZF90cmFuc3BvcnQiLCAibmVydm91c19zeXN0ZW1fZGV2ZWxvcG1lbnQiKSwKICAgICAgICAgICAgICAgICAgICAgICBjKCJ2ZXNpY2xlX21lZGlhdGVkX3RyYW5zcG9ydCIsICJzeW5hcHNlX2Fzc2VtYmx5IiApLAogICAgICAgICAgICAgICAgICAgICAgIGMoImN5dG9za2VsZXRvbl9vcmdhbml6YXRpb24iLCAic2lnbmFsX3RyYW5zZHVjdGlvbiIgKSwKICAgICAgICAgICAgICAgICAgICAgICBjKCJjeXRvc2tlbGV0b25fb3JnYW5pemF0aW9uIiwgIm5lcnZvdXNfc3lzdGVtX2RldmVsb3BtZW50IiApLAogICAgICAgICAgICAgICAgICAgICAgIGMoImN5dG9za2VsZXRvbl9vcmdhbml6YXRpb24iLCAic3luYXBzZV9hc3NlbWJseSIpCiAgICAgICAgICAgICAgICAgICAgICAgI2MoInNpZ25hbF90cmFuc2R1Y3Rpb24iLCAibmVydm91c19zeXN0ZW1fZGV2ZWxvcG1lbnQiICksCiAgICAgICAgICAgICAgICAgICAgICAgI2MoInNpZ25hbF90cmFuc2R1Y3Rpb24iLCAibmVydm91c19zeXN0ZW1fZGV2ZWxvcG1lbnQiICksCiAgICAgICAgICAgICAgICAgICAgICAgI2MoIm5lcnZvdXNfc3lzdGVtX2RldmVsb3BtZW50IiwgInN5bmFwc2VfYXNzZW1ibHkiICkKICAgICAgICAgICAgICAgICAgICAgICApCgoKRjZIIDwtICBnZ3Bsb3QoIEdPX0Jpb2xvZ2ljYWxfUHJvY2Vzc19jZW50cmFsaXR5LCBhZXMoR08sIGVpZ2VuX2NlbnRyYWxpdHkgICkgICkgICsKICBnZW9tX2JveHBsb3QoKSArCiAgICB5bGFiKCJFaWdlbmNlbnRyYWxpdHkiKSArCiAgIHRoZW1lX2J3KCkgKwogICAgdGhlbWUoYXhpcy50ZXh0LnggPSBlbGVtZW50X3RleHQoYW5nbGUgPSA5MCwgdmp1c3QgPSAwLjYpLAogICAgYXhpcy50aXRsZS54ID0gZWxlbWVudF90ZXh0KGNvbG91ciA9IE5BKSwKICAgIHN0cmlwLnRleHQgPSBlbGVtZW50X3RleHQoaGp1c3QgPSAwLjUpKSArCiAgZ2dzaWduaWY6Omdlb21fc2lnbmlmKHRlc3QgPSAid2lsY294LnRlc3QiLCBjb21wYXJpc29ucyA9IG15X2NvbXBhcmlzb25zLCBzdGVwX2luY3JlYXNlPTAuMTUgLCB0ZXN0LmFyZ3MgPSBsaXN0KGFsdGVybmF0aXZlID0gImdyZWF0ZXIiLCBwYWlyZWQgPSBGQUxTRSksIG1hcF9zaWduaWZfbGV2ZWwgPSBUUlVFKSArCiAgc2NhbGVfeF9kaXNjcmV0ZShsYWJlbHM9YygiVmVzaWNsZSBtZWRpYXRlZCB0cmFuc3BvcnR0IiwgIkN5dG9za2VsZXRvbiBvcmdhbml6YXRpb24iLCAiU2lnbmFsIHRyYW5zZHVjdGlvbiIsICJOZXJ2b3VzIHN5c3RlbSBkZXZlbG9wbWVudCIsICJTeW5hcHNlIGFzc2VtYmx5IikpCgpGNkgKCmBgYAoKCmBgYHtyLCBmaWcuaGVpZ2h0PSA3LCBmaWcud2lkdGg9NX0KCkdPX0Jpb2xvZ2ljYWxfUHJvY2Vzc19jZW50cmFsaXR5X0hOTV9hZ2UgPC0gbWVyZ2UoR09fQmlvbG9naWNhbF9Qcm9jZXNzX2NlbnRyYWxpdHksIERlbHRhX0hOTUZfd2hpcHBldF9NRV9hZ2VfZGlmZl90b3RhbF9QUElbaXMubmEoZGlmZl9hZ2UueCkhPVRSVUVdWyAsIC4obWluX2FnZV9ITk09bWluKGRpZmZfYWdlLngpKSAgLCBieT0ibWFwcGVkX2dlbmUiIF0sIGJ5PSJtYXBwZWRfZ2VuZSIpCgprcnVza2FsLnRlc3QoZGF0YSA9IEdPX0Jpb2xvZ2ljYWxfUHJvY2Vzc19jZW50cmFsaXR5X0hOTV9hZ2UsICBtaW5fYWdlX0hOTSB+IEdPKQoKCm15X2NvbXBhcmlzb25zIDwtIGxpc3QoI2MoInZlc2ljbGVfbWVkaWF0ZWRfdHJhbnNwb3J0IiwgImN5dG9za2VsZXRvbl9vcmdhbml6YXRpb24iICksCiAgICAgICAgICAgICAgICAgICAgICAgI2MoInZlc2ljbGVfbWVkaWF0ZWRfdHJhbnNwb3J0IiwgInNpZ25hbF90cmFuc2R1Y3Rpb24iICksCiAgICAgICAgICAgICAgICAgICAgICAgI2MoInZlc2ljbGVfbWVkaWF0ZWRfdHJhbnNwb3J0IiwgIm5lcnZvdXNfc3lzdGVtX2RldmVsb3BtZW50IiksCiAgICAgICAgICAgICAgICAgICAgICAgI2MoInZlc2ljbGVfbWVkaWF0ZWRfdHJhbnNwb3J0IiwgInN5bmFwc2VfYXNzZW1ibHkiICksCiAgICAgICAgICAgICAgICAgICAgICAgYygiY3l0b3NrZWxldG9uX29yZ2FuaXphdGlvbiIsICJzaWduYWxfdHJhbnNkdWN0aW9uIiApLAogICAgICAgICAgICAgICAgICAgICAgIGMoImN5dG9za2VsZXRvbl9vcmdhbml6YXRpb24iLCAibmVydm91c19zeXN0ZW1fZGV2ZWxvcG1lbnQiICksCiAgICAgICAgICAgICAgICAgICAgICAgYygiY3l0b3NrZWxldG9uX29yZ2FuaXphdGlvbiIsICJzeW5hcHNlX2Fzc2VtYmx5IikKICAgICAgICAgICAgICAgICAgICAgICAjYygic2lnbmFsX3RyYW5zZHVjdGlvbiIsICJuZXJ2b3VzX3N5c3RlbV9kZXZlbG9wbWVudCIgKSwKICAgICAgICAgICAgICAgICAgICAgICAjYygic2lnbmFsX3RyYW5zZHVjdGlvbiIsICJuZXJ2b3VzX3N5c3RlbV9kZXZlbG9wbWVudCIgKSwKICAgICAgICAgICAgICAgICAgICAgICAjYygibmVydm91c19zeXN0ZW1fZGV2ZWxvcG1lbnQiLCAic3luYXBzZV9hc3NlbWJseSIgKQogICAgICAgICAgICAgICAgICAgICAgICkKCgpGNkkgPC0gZ2dwbG90KCBHT19CaW9sb2dpY2FsX1Byb2Nlc3NfY2VudHJhbGl0eV9ITk1fYWdlLCBhZXMoR08sIG1pbl9hZ2VfSE5NICApICApICArCiAgZ2VvbV9ib3hwbG90KCkgKwogICAgeWxhYigiRWFybGllc3QgbWljcm9leG9uIGFsdGVybmF0aXZlIGluY2x1c2lvbiBwZXIgZ2VuZSIpICsKICAgdGhlbWVfYncoKSArCiAgICB0aGVtZShheGlzLnRleHQueCA9IGVsZW1lbnRfdGV4dChhbmdsZSA9IDkwLCB2anVzdCA9IDAuNiksCiAgICBheGlzLnRpdGxlLnggPSBlbGVtZW50X3RleHQoY29sb3VyID0gTkEpLAogICAgc3RyaXAudGV4dCA9IGVsZW1lbnRfdGV4dChoanVzdCA9IDAuNSkpICsKICAgICAgc2NhbGVfeV9jb250aW51b3VzKGJyZWFrcz1zZXEoMTAuNSwxNi41LDEpKSArCiAgZ2dzaWduaWY6Omdlb21fc2lnbmlmKHRlc3QgPSAid2lsY294LnRlc3QiLCBjb21wYXJpc29ucyA9IG15X2NvbXBhcmlzb25zLCBzdGVwX2luY3JlYXNlPTAuMTUgLCB0ZXN0LmFyZ3MgPSBsaXN0KGFsdGVybmF0aXZlID0gImxlc3MiLCBwYWlyZWQgPSBGQUxTRSksIG1hcF9zaWduaWZfbGV2ZWwgPSBUUlVFKSArCiAgc2NhbGVfeF9kaXNjcmV0ZShsYWJlbHM9YygiVmVzaWNsZSBtZWRpYXRlZCB0cmFuc3BvcnR0IiwgIkN5dG9za2VsZXRvbiBvcmdhbml6YXRpb24iLCAiU2lnbmFsIHRyYW5zZHVjdGlvbiIsICJOZXJ2b3VzIHN5c3RlbSBkZXZlbG9wbWVudCIsICJTeW5hcHNlIGFzc2VtYmx5IikpCgoKCmtydXNrYWwudGVzdChHT19CaW9sb2dpY2FsX1Byb2Nlc3NfY2VudHJhbGl0eV9ITk1fYWdlJG1pbl9hZ2VfSE5NLCBHT19CaW9sb2dpY2FsX1Byb2Nlc3NfY2VudHJhbGl0eV9ITk1fYWdlJEdPKQpwYWlyd2lzZS53aWxjb3gudGVzdChHT19CaW9sb2dpY2FsX1Byb2Nlc3NfY2VudHJhbGl0eV9ITk1fYWdlJG1pbl9hZ2VfSE5NLCBHT19CaW9sb2dpY2FsX1Byb2Nlc3NfY2VudHJhbGl0eV9ITk1fYWdlJEdPKQoKYGBgCgoKCmBgYHtyLCBmaWcuaGVpZ2h0PTE1LCBmaWcud2lkdGg9Ny41fQoKRjZISSA8LSBwbG90X2dyaWQoRjZIICwgRjZJICwgbGFiZWxzID0gYyggJ0gnLCAnSScpLCBucm93PTEpCgpGNi5ib3R0b20gPC0gcGxvdF9ncmlkKEY2RkcsIEY2SEksIG5jb2w9MSwgcmVsX2hlaWdodHMgICA9IGMoMiwxKSkKRjYuYm90dG9tCmBgYAoKCgpgYGB7cn0KbGlicmFyeShnZ3N0YXRzcGxvdCkKZGZfcGFpciA8LSBnZ3N0YXRzcGxvdDo6cGFpcndpc2VfcChHT19CaW9sb2dpY2FsX1Byb2Nlc3NfY2VudHJhbGl0eSwgZWlnZW5fY2VudHJhbGl0eSwgIHR5cGUgPSAibm9ucGFyYW1ldHJpYyIsIEdPKQoKYGBgCgoKCmBgYHtyfQprcnVza2FsLnRlc3QoZGF0YSA9IEdPX0Jpb2xvZ2ljYWxfUHJvY2Vzc19jZW50cmFsaXR5LCAgZWlnZW5fY2VudHJhbGl0eSB+IEdPKQprcnVza2FsLnRlc3QoZGF0YSA9IEdPX0Jpb2xvZ2ljYWxfUHJvY2Vzc19jZW50cmFsaXR5LCAgaGFybW9uaWNfY2VudHJhbGl0eSB+IEdPKQoKCgpwYWlyd2lzZS53aWxjb3gudGVzdChHT19CaW9sb2dpY2FsX1Byb2Nlc3NfY2VudHJhbGl0eSRlaWdlbl9jZW50cmFsaXR5LCBHT19CaW9sb2dpY2FsX1Byb2Nlc3NfY2VudHJhbGl0eSRHTykKcGFpcndpc2Uud2lsY294LnRlc3QoR09fQmlvbG9naWNhbF9Qcm9jZXNzX2NlbnRyYWxpdHkkaGFybW9uaWNfY2VudHJhbGl0eSwgR09fQmlvbG9naWNhbF9Qcm9jZXNzX2NlbnRyYWxpdHkkR08pCmBgYAoKYGBge3J9CnBhaXJ3aXNlLndpbGNveC50ZXN0KEdPX0Jpb2xvZ2ljYWxfUHJvY2Vzc19jZW50cmFsaXR5JGVpZ2VuX2NlbnRyYWxpdHksIEdPX0Jpb2xvZ2ljYWxfUHJvY2Vzc19jZW50cmFsaXR5JEdPKQpwYWlyd2lzZS53aWxjb3gudGVzdChHT19CaW9sb2dpY2FsX1Byb2Nlc3NfY2VudHJhbGl0eSRoYXJtb25pY19jZW50cmFsaXR5LCBHT19CaW9sb2dpY2FsX1Byb2Nlc3NfY2VudHJhbGl0eSRHTykKYGBgCgoKCgoKYGBge3J9CkJpb2xvZ2ljYWxfUHJvY2VzcyA8LSBmcmVhZCgiLi4vRmlndXJlcy9OZXdfRmlndXJlIDUvU1RSSU5HL0dPL01FX2dlbmVzX2JhY2tncm91bmQvQmlvbG9naWNhbF9Qcm9jZXNzLnRzdiIpCkNlbGx1bGFyX0NvbXBvbmVudCA8LSBmcmVhZCgiLi4vRmlndXJlcy9OZXdfRmlndXJlIDUvU1RSSU5HL0dPL01FX2dlbmVzX2JhY2tncm91bmQvQ2VsbHVsYXJfQ29tcG9uZW50LnRzdiIpClByb3RlaW5fRG9tYWlucyA8LSBmcmVhZCgiLi4vRmlndXJlcy9OZXdfRmlndXJlIDUvU1RSSU5HL0dPL01FX2dlbmVzX2JhY2tncm91bmQvSU5URVJQUk8gUHJvdGVpbiBEb21haW5zIGFuZCBGZWF0dXJlcy50c3YiKQoKCgpCaW9sb2dpY2FsX1Byb2Nlc3NbICwgYDo9YCggbWludXNfbG9nMTBfRkRSPS1sb2cxMChgZmFsc2UgZGlzY292ZXJ5IHJhdGVgKSwKICAgICAgICAgICAgICAgICAgICAgICAgICAgIEdPX3NpemU9KGBvYnNlcnZlZCBnZW5lIGNvdW50YCArIGBiYWNrZ3JvdW5kIGdlbmUgY291bnRgKSAsCiAgICAgICAgICAgICAgICAgICAgICAgICAgICBHT19mcmFjdGlvbj0oYG9ic2VydmVkIGdlbmUgY291bnRgL2BiYWNrZ3JvdW5kIGdlbmUgY291bnRgKSAsCiAgICAgICAgICAgICAgICAgICAgICAgICAgICBHT19jbGFzcz0iQmlvbG9naWNhbCBQcm9jZXNzIikgXQoKCgpDZWxsdWxhcl9Db21wb25lbnRbICwgYDo9YCggbWludXNfbG9nMTBfRkRSPS1sb2cxMChgZmFsc2UgZGlzY292ZXJ5IHJhdGVgKSwKICAgICAgICAgICAgICAgICAgICAgICAgICAgIEdPX3NpemU9KGBvYnNlcnZlZCBnZW5lIGNvdW50YCArIGBiYWNrZ3JvdW5kIGdlbmUgY291bnRgKSAsCiAgICAgICAgICAgICAgICAgICAgICAgICAgICBHT19mcmFjdGlvbj0oYG9ic2VydmVkIGdlbmUgY291bnRgL2BiYWNrZ3JvdW5kIGdlbmUgY291bnRgKSAsCiAgICAgICAgICAgICAgICAgICAgICAgICAgICBHT19jbGFzcz0iQ2VsbHVsYXIgQ29tcG9uZW50IikgXQoKClByb3RlaW5fRG9tYWluc1sgLCBgOj1gKCBtaW51c19sb2cxMF9GRFI9LWxvZzEwKGBmYWxzZSBkaXNjb3ZlcnkgcmF0ZWApLAogICAgICAgICAgICAgICAgICAgICAgICAgICAgR09fc2l6ZT0oYG9ic2VydmVkIGdlbmUgY291bnRgICsgYGJhY2tncm91bmQgZ2VuZSBjb3VudGApICwKICAgICAgICAgICAgICAgICAgICAgICAgICAgIEdPX2ZyYWN0aW9uPShgb2JzZXJ2ZWQgZ2VuZSBjb3VudGAvYGJhY2tncm91bmQgZ2VuZSBjb3VudGApICwKICAgICAgICAgICAgICAgICAgICAgICAgICAgIEdPX2NsYXNzPSJJTlRFUlBSTyBQcm90ZWluIERvbWFpbnMiKSBdCgoKVG90YWxfR08gPC0gcmJpbmQoQmlvbG9naWNhbF9Qcm9jZXNzLCBDZWxsdWxhcl9Db21wb25lbnQsIFByb3RlaW5fRG9tYWlucykKCgoKZ2dwbG90KFRvdGFsX0dPW0dPX3NpemU+MjBdKSArIAogIGdlb21fcG9pbnQoIGFlcyhHT19mcmFjdGlvbiwgbWludXNfbG9nMTBfRkRSLCBzaXplPWxvZzIoR09fc2l6ZSksIGNvbG91cj1HT19jbGFzcyksICBhbHBoYT0wLjIpICsKICBmYWNldF9ncmlkKCAuIH4gIEdPX2NsYXNzICkgKwogIHRoZW1lKGF4aXMudGV4dC54ID0gZWxlbWVudF90ZXh0KGFuZ2xlID0gOTApLCBsZWdlbmQucG9zaXRpb24gPSAidG9wIiwgbGVnZW5kLmRpcmVjdGlvbiA9ICJob3Jpem9udGFsIikKYGBgCgoKCgoKCmBgYHtyfQpnZ3Bsb3QoUFBJX2RpZmZfYWdlX0hOTV9zdGF0c19FQykgKwogIGdlb21fYmFyKGFlcyhtaW5fZGlmX2FnZSwgZmlsbD1jZW50cmFsKSwgc3RhdCA9ICJjb3VudCIsIHBvc2l0aW9uPSJmaWxsIiApICsKICB4bGFiKCJFYXJsaWVzdCBtaWNyb2V4b24gZGlmZmVyZW50aWFsIGluY2x1c2lvbiBkZXRlY3Rpb24gcGVyIGdlbmUgKERQQykiKSsKICB5bGFiKCJQcm90ZWluIHR5cGUgcHJvcG9ydGlvbiIpICsKICBzY2FsZV94X2NvbnRpbnVvdXMoYnJlYWtzPXNlcSgxMC41LDE2LjUsMSkpICsKICBzY2FsZV9maWxsX2Rpc2NyZXRlKG5hbWUgPSAiUHJvdGVpbiB0eXBlIiwgbGFiZWxzID0gYygiTm9uLWNlbnRyYWwiLCAiQ2VudHJhbCIpKSAgKwogICAgICAgICAgICB0aGVtZShheGlzLnRleHQueCA9IGVsZW1lbnRfdGV4dChhbmdsZSA9IDkwKSwgbGVnZW5kLnBvc2l0aW9uID0gInRvcCIsIGxlZ2VuZC5kaXJlY3Rpb24gPSAiaG9yaXpvbnRhbCIpCgoKZ2dwbG90KFBQSV9kaWZmX2FnZV9ITk1fc3RhdHNfQikgKwogIGdlb21fYmFyKGFlcyhtaW5fZGlmX2FnZSwgZmlsbD1jZW50cmFsKSwgc3RhdCA9ICJjb3VudCIsIHBvc2l0aW9uPSJmaWxsIiApICsKICB4bGFiKCJFYXJsaWVzdCBtaWNyb2V4b24gZGlmZmVyZW50aWFsIGluY2x1c2lvbiBkZXRlY3Rpb24gcGVyIGdlbmUgKERQQykiKSsKICB5bGFiKCJQcm90ZWluIHR5cGUgcHJvcG9ydGlvbiIpICsKICBzY2FsZV94X2NvbnRpbnVvdXMoYnJlYWtzPXNlcSgxMC41LDE2LjUsMSkpICsKICBzY2FsZV9maWxsX2Rpc2NyZXRlKG5hbWUgPSAiUHJvdGVpbiB0eXBlIiwgbGFiZWxzID0gYygiTm9uLWNlbnRyYWwiLCAiQ2VudHJhbCIpKSAgKwogICAgICAgICAgICB0aGVtZShheGlzLnRleHQueCA9IGVsZW1lbnRfdGV4dChhbmdsZSA9IDkwKSwgbGVnZW5kLnBvc2l0aW9uID0gInRvcCIsIGxlZ2VuZC5kaXJlY3Rpb24gPSAiaG9yaXpvbnRhbCIpCgpgYGAKCiNaZWJyYWZpc2gKCmBgYHtyLCBmaWd1cmV9Cgp0b3RhbF96ZWJyYSA8LSAgZnJlYWQoIi4uLy4uL1plYnJhZmlzaC9GaW5hbF9SZXBvcnQvb3V0X2ZpbHRlcmVkX01FLnR4dCIpCnRvdGFsX3plYnJhX2NvdiA8LSBmcmVhZCgiLi4vLi4vWmVicmFmaXNoL0ZpbmFsX1JlcG9ydC9vdXRfZmlsdGVyZWRfTUUuY292LnR4dCIpCgoKZXhvbnMuemVicmEgPC0gZnJlYWQoIi4uLy4uL1plYnJhZmlzaC9kYW5SZXIxMS5lbnNlbWJsLmJlZDEyLmV4b25zIikKCgptb3VzZV90b196ZWJyYSA8LSBmcmVhZCgiLi4vLi4vWmVicmFmaXNoL0NvbnNlcnZlZC9vdXQuaGlnaF9xdWFsaXR5LnR4dC5tbTEwLmJlZC5saWZ0T3Zlcl9taW5NYXRjaF8wLjAxLmRhblJlcjExLmNsb3Nlc3RfZC5vdXQuaGlnaF9xdWFsaXR5LnR4dC5kYW5SZXIxMS5iZWQuZm9ybWF0dGVkIikKemVicmFfdG9fbW91c2UgPC0gZnJlYWQoIi4uLy4uL1plYnJhZmlzaC9Db25zZXJ2ZWQvb3V0LmhpZ2hfcXVhbGl0eS50eHQuZGFuUmVyMTEuYmVkLmxpZnRPdmVyX21pbk1hdGNoXzAuMDEubW0xMC5jbG9zZXN0X2Qub3V0LmhpZ2hfcXVhbGl0eS50eHQubW0xMC5mb3JtYXR0ZWQiKQoKCgptb3VzZV90b196ZWJyYSA8LSBmcmVhZCgiLi4vLi4vWmVicmFmaXNoL0NvbnNlcnZlZC9PbGQvb3V0LmhpZ2hfcXVhbGl0eS5tbTEwLmJlZF9saWZ0b3Zlcl9tbTEwdG9kYW5SZXIxMS5vdmVybGFwX3dvLm91dC5oaWdoX3F1YWxpdHkuYmVkIikKemVicmFfdG9fbW91c2UgPC0gZnJlYWQoIi4uLy4uL1plYnJhZmlzaC9Db25zZXJ2ZWQvT2xkL291dC5oaWdoX3F1YWxpdHkuYmVkLmxpZnRvdmVyXzAuMDAxX21tMTAub3ZlcmxhcF93by5vdXQuaGlnaF9xdWFsaXR5LmJlZC5saWZ0b3Zlcl8wLjAwMV9tbTEwIikKCk1vdXNlX1plYnJhLklEcyA8LSB1bmlxdWUocmJpbmQobW91c2VfdG9femVicmFbICAsIC4oTUUubW91c2U9VjQsIE1FLnplYnJhPVYxMCApIF0sIHplYnJhX3RvX21vdXNlWyAsIC4oTUUubW91c2U9VjEwLCBNRS56ZWJyYT1WNCkgXSApKSAgI0luIGhlcmUgd2UgYXJlIGFscmVhZHkgYXBwbHlpbmcgYSBmaWx0ZXIgb2YgMC44IGxlbmdoCgoKI1ZpZXcoTW91c2VfWmVicmEuSURzWywgLihtb3VzZS5sZW4sIHplYnJhLmxlbiwgYWJzKG1vdXNlLmxlbi1hYnMobW91c2UubGVuLXplYnJhLmxlbikpL21heChtb3VzZS5sZW4semVicmEubGVuKSApXSApCgoKbW91c2VfbGVuIDwtIGMobW91c2VfdG9femVicmFbLCBWMy1WMl0sIHplYnJhX3RvX21vdXNlWywgVjktVjhdKQpuYW1lcyhtb3VzZV9sZW4pIDwtIGMobW91c2VfdG9femVicmEkVjQsIHplYnJhX3RvX21vdXNlJFYxMCkKCgp6ZWJyYV9sZW4gPC0gIGMoemVicmFfdG9fbW91c2VbLCBWMy1WMl0sIG1vdXNlX3RvX3plYnJhWywgVjktVjhdKQpuYW1lcyh6ZWJyYV9sZW4pIDwtIGMoemVicmFfdG9fbW91c2UkVjQsIG1vdXNlX3RvX3plYnJhJFYxMCkKCgoKCk1vdXNlX1plYnJhLklEc1ssIG1vdXNlLmxlbjo9bW91c2VfbGVuW01FLm1vdXNlXV0KTW91c2VfWmVicmEuSURzWywgemVicmEubGVuOj16ZWJyYV9sZW5bTUUuemVicmFdXQoKCmdncGxvdChNb3VzZV9aZWJyYS5JRHMpICsKICBnZW9tX2JpbjJkKGFlcyhtb3VzZS5sZW4sIHplYnJhLmxlbiksIGJpbnMgPSAzMCwgY29sb3IgPSJ3aGl0ZSIpICsKICB0aGVtZV9idygpICsKICBzY2FsZV9maWxsX2dyYWRpZW50KGxvdyA9ICAiIzAwQUZCQiIsIGhpZ2ggPSAiI0ZDNEUwNyIpCgoKTW91c2VfWmVicmEuSURzIDwtIE1vdXNlX1plYnJhLklEc1ttb3VzZS5sZW49PXplYnJhLmxlbiwgXQoKTW91c2VfWmVicmEuSURzLnRhYmxlIDwtIG1lcmdlKE1vdXNlX1plYnJhLklEcywgTUVfY2x1c3Rlcl9uYW1lc1sgLCBjKCJNRV9jbHVzdGVyLm5hbWUiICwgIk1FX2NsdXN0ZXIudHlwZSIpXSwgYnk9Ik1FX2NsdXN0ZXIubmFtZSIsIGFsbCA9IFRSVUUpCgoKTW91c2VfWmVicmEuSURzLnRhYmxlIDwtIE1vdXNlX1plYnJhLklEcy50YWJsZVsgLCAuKG1vdXNlLmNvb3JkPU1FLm1vdXNlLCBtb3VzZS5sZW49bW91c2UubGVuLCBtb3VzZS5jbHVzdGVyX25hbWU9TUVfY2x1c3Rlci5uYW1lLCBtb3VzZS5jbHVzdGVyX3R5cGU9TUVfY2x1c3Rlci50eXBlLCAKICAgICAgICAgICAgICAgICAgICAgICAgICAgbW91c2UuYW5ubz10eXBlLCBtb3VzZS5lbnNlbWJsPWVuc2VtYmxlX21vdXNlLCAKICAgICAgICAgICAgICAgICAgICAgICAgICAgemVicmEuY29vcmQ9TUUuemVicmEsIHplYnJhLmxlbj16ZWJyYS5sZW4sIHplYnJhLmVuc2VtYmw9ZW5zZW1ibGVfemVicmEpXSAKCgppcy5kYXRhLnRhYmxlKE1vdXNlX1plYnJhLklEcy50YWJsZSkKCk1vdXNlX1plYnJhLklEcy50YWJsZSA8LSB1bmlxdWUobWVyZ2UoTW91c2VfWmVicmEuSURzLnRhYmxlLCBnZW5lX3RhYmxlLlRPVEFMWyAsIGMoIk1FIiwgIm1naV9zeW1ib2wiKV0sIGJ5Lng9Im1vdXNlLmNvb3JkIiAsIGJ5Lnk9Ik1FIiApKQoKZGl2XzMgPC0gZnVuY3Rpb24oeCkgeCAlJSAzID09IDBdCgpNb3VzZV9aZWJyYS5JRHMudGFibGVbICwgLihtb3VzZS5jb29yZCwgbWdpX3N5bWJvbCwgbW91c2UuY2x1c3Rlcl9uYW1lLCBtb3VzZS5jbHVzdGVyX3R5cGUsIG1vdXNlLmxlbiwgbW91c2Uuc3ltPShtb3VzZS5sZW4lJTMgPT0gMCksIG1vdXNlLmVuc2VtYmwsIHplYnJhLmNvb3JkLCB6ZWJyYS5sZW4sIHplYnJhLnN5bT0oemVicmEubGVuJSUzID09IDApLCB6ZWJyYS5lbnNlbWJsKSAgXQoKCk1vdXNlX1plYnJhLklEcy50aGVzaXMudGFibGUgPC0gdW5pcXVlKE1vdXNlX1plYnJhLklEcy50YWJsZVsgLCAuKG1vdXNlLmNvb3JkLCBtZ2lfc3ltYm9sLCBtb3VzZS5jbHVzdGVyX25hbWUsIG1vdXNlLmNsdXN0ZXJfdHlwZSwKICAgICAgICAgICAgICAgICAgICAgICAgICAgICAgICAgICAgICAgICAgICAgICAgICAgICAgICAgICAgICAgICAgbW91c2UubGVuLCBtb3VzZS5zeW09KG1vdXNlLmxlbiUlMyA9PSAwKSwgbW91c2UuZW5zZW1ibCwKICAgICAgICAgICAgICAgICAgICAgICAgICAgICAgICAgICAgICAgICAgICAgICAgICAgICAgICAgICAgICAgICAgemVicmEubnVtPS5OLAogICAgICAgICAgICAgICAgICAgICAgICAgICAgICAgICAgICAgICAgICAgICAgICAgICAgICAgICAgICAgICAgICB6ZWJyYS5jb29yZHM9YXMuY2hhcmFjdGVyKGxpc3QoemVicmEuY29vcmQpKSwKICAgICAgICAgICAgICAgICAgICAgICAgICAgICAgICAgICAgICAgICAgICAgICAgICAgICAgICAgICAgICAgICAgemVicmEubGVucz1hcy5jaGFyYWN0ZXIobGlzdCh6ZWJyYS5sZW4pKSwKICAgICAgICAgICAgICAgICAgICAgICAgICAgICAgICAgICAgICAgICAgICAgICAgICAgICAgICAgICAgICAgICAgemVicmEuc3ltcz1hcy5jaGFyYWN0ZXIobGlzdCgoemVicmEubGVuJSUzID09IDApKSksCiAgICAgICAgICAgICAgICAgICAgICAgICAgICAgICAgICAgICAgICAgICAgICAgICAgICAgICAgICAgICAgICAgIHplYnJhLmVuc2VtYmxzPWFzLmNoYXJhY3RlcihsaXN0KHplYnJhLmVuc2VtYmwpICkpLAogICAgICAgICAgICAgICAgICAgICAgICAgICAgICAgICAgICAgICAgICAgICAgICAgICAgICAgICAgICAgIGJ5PSBtb3VzZS5jb29yZCAgXVtvcmRlcihtZ2lfc3ltYm9sLCBtb3VzZS5jb29yZCldKQoKCgpNb3VzZV9aZWJyYS5JRHMudGhlc2lzLnRhYmxlWyAsIGA6PWAoIHplYnJhLmNvb3Jkcz1nc3ViKCdeY1xcKHxcXCkkfFwgfFwiJyAsICIiLCB6ZWJyYS5jb29yZHMpLAogICAgICAgICAgICAgICAgICAgICAgICAgICAgICAgICAgICAgIHplYnJhLmxlbnM9Z3N1YignXmNcXCh8XFwpJHxcIHxcIicgLCAiIiwgemVicmEubGVucyksCiAgICAgICAgICAgICAgICAgICAgICAgICAgICAgICAgICAgICAgemVicmEuc3ltcz1nc3ViKCdeY1xcKHxcXCkkfFwgfFwiJyAsICIiLCB6ZWJyYS5zeW1zKSwKICAgICAgICAgICAgICAgICAgICAgICAgICAgICAgICAgICAgICB6ZWJyYS5lbnNlbWJscz1nc3ViKCdeY1xcKHxcXCkkfFwgfFwiJyAsICIiLCB6ZWJyYS5lbnNlbWJscykKICAgICAgICAgICAgICAgICAgICAgICAgICAgICAgICAgICAgICAgICAgICAgICAgICAgICAgICApXQoKCgoKZndyaXRlKE1vdXNlX1plYnJhLklEcy50YWJsZSwgIi4uL0ZpbmFsX0ZpZ3VyZXMvU3VwcGxlbWVudGFyeS9Nb3VzZV9aZWJyYV9taWNyb2V4b25zLnRzdiIsIGFwcGVuZCA9IEZBTFNFLCBxdW90ZSA9ICJhdXRvIiwgc2VwID0gIiwiLCAgcm93Lm5hbWVzID0gRkFMU0UsIGNvbC5uYW1lcyA9IFRSVUUgKQpmd3JpdGUoTW91c2VfWmVicmEuSURzLnRoZXNpcy50YWJsZSwgIn4vR29vZ2xlX0RyaXZlL1RoZXNpcy9Nb3VzZV96ZWJyYWZpc2gudHN2IiwgYXBwZW5kID0gRkFMU0UsIHF1b3RlID0gImF1dG8iLCBzZXAgPSAiXHQiLCAgcm93Lm5hbWVzID0gRkFMU0UsIGNvbC5uYW1lcyA9IFRSVUUgKQpgYGAKICAKYGBge3J9CgpNb3VzZV9aZWJyYS5JRHMudGFibGUuc3VtbWVyIDwtIG1lcmdlKE1vdXNlX1plYnJhLklEcy50YWJsZSwgRGVsdGFfSE5NX3doaXBwZXRfTUVfYWdlX2RpZmZfdG90YWxbICwgLihleG9uX0lELCBNSE4uZGlmZl9hZ2U9ZGlmZl9hZ2UsICBNSE4uY2hhbmdlX2Rpcj1jaGFuZ2VfZGlyKV0sIGJ5Lng9Im1vdXNlLmNvb3JkIiwgYnkueT0iZXhvbl9JRCIsIGFsbC54ID0gVCkKCgpNb3VzZV9aZWJyYS5JRHMudGFibGUuc3VtbWVyIDwtIG1lcmdlKE1vdXNlX1plYnJhLklEcy50YWJsZS5zdW1tZXIsIERlbHRhX0Zfd2hpcHBldF9NRV9hZ2VfZGlmZl90b3RhbFsgLCAuKGV4b25fSUQsIEYuZGlmZl9hZ2U9ZGlmZl9hZ2UsICBGLmNoYW5nZV9kaXI9Y2hhbmdlX2RpcildLCBieS54PSJtb3VzZS5jb29yZCIsIGJ5Lnk9ImV4b25fSUQiLCBhbGwueCA9IFQpCgoKZndyaXRlKE1vdXNlX1plYnJhLklEcy50YWJsZS5zdW1tZXIsICIuLi9GaW5hbF9GaWd1cmVzL1N1cHBsZW1lbnRhcnkvTW91c2VfWmVicmFfbWljcm9leG9ucy5zdW1tZXIudHN2IiwgYXBwZW5kID0gRkFMU0UsIHF1b3RlID0gImF1dG8iLCBzZXAgPSAiLCIsICByb3cubmFtZXMgPSBGQUxTRSwgY29sLm5hbWVzID0gVFJVRSApCgpNb3VzZV9aZWJyYS5JRHMudGFibGUuc3VtbWVyWyFpcy5uYShtb3VzZS5jb29yZCksIF0KYGBgCgogIAoKYGBge3J9CgoKCk1vdXNlX1plYnJhLklEcwoKTUVfY2x1c3RlcnMuaW5mbwpgYGAKCgoKICAKICAKYGBge3J9ICAKTW91c2VfWmVicmEuSURzW01FLm1vdXNlICVpbiUgIG5hbWVzKE1FX2NsdXN0ZXJzKSwgTUVfY2x1c3Rlcjo9TUVfY2x1c3RlcnNbTUUubW91c2VdIF0KCgoKTW91c2VfWmVicmEuSURzPC0gbWVyZ2UoTW91c2VfWmVicmEuSURzICwgCk1FX2NsdXN0ZXJfbmFtZXNbLCBjKCJNRV9jbHVzdGVyIiwgIk1FX2NsdXN0ZXIubmFtZSIpXSAsIGJ5PSJNRV9jbHVzdGVyIiwgYWxsLng9VFJVRSApCgoKZ2dwbG90KE1vdXNlX1plYnJhLklEcykgKwogIGdlb21fYmFyKGFlcyh4PU1FX2NsdXN0ZXIubmFtZSksIHN0YXQ9ImNvdW50IikKCgoKCk1vdXNlX1plYnJhLklEc1sgLCB0eXBlOj0iQW5ub3RhdGVkIiAgXQoKTW91c2VfWmVicmEuSURzWyFNRS5tb3VzZSAlaW4lIG1pY3JvZXhvbnNfVmFzdGRiLCB0eXBlOj0iTWlzc2luZyBpbiBWYXN0REIiICAgXQpNb3VzZV9aZWJyYS5JRHNbIU1FLm1vdXNlICVpbiUgbWljcm9leG9uc19HRU5DT0RFLCB0eXBlOj0iTWlzc2luZyBpbiBHRU5DT0RFIiAgIF0KTW91c2VfWmVicmEuSURzWyFNRS5tb3VzZSAlaW4lIG1pY3JvZXhvbnNfR0VOQ09ERSAmICFNRS5tb3VzZSAlaW4lIG1pY3JvZXhvbnNfVmFzdGRiICwgdHlwZTo9Ik5vdmVsIiAgIF0KCgpNb3VzZV9aZWJyYS5JRHNbICwgZW5zZW1ibGVfemVicmE6PUZBTFNFICBdCk1vdXNlX1plYnJhLklEc1tNRS56ZWJyYSAlaW4lICBleG9ucy56ZWJyYSRWNCwgZW5zZW1ibGVfemVicmE6PVRSVUUgXQoKTW91c2VfWmVicmEuSURzWyAsIGVuc2VtYmxlX21vdXNlOj1GQUxTRSAgXQpNb3VzZV9aZWJyYS5JRHNbTUUubW91c2UgJWluJSAgbWljcm9leG9uc19HRU5DT0RFLCBlbnNlbWJsZV9tb3VzZTo9VFJVRSBdCgoKCgoKCgpEZWx0YV9ITk1GX3doaXBwZXRfTUVfYWdlX2RpZmZfdG90YWxbZXhvbl9JRCAlaW4lIE1vdXNlX1plYnJhLklEcyRNRS5tb3VzZV0KCgpEZWx0YV9ITk1GX3doaXBwZXRfTUVfYWdlX2RpZmZfdG90YWxbXQoKCk1vdXNlX1plYnJhLklEc1tNRS5tb3VzZSAlaW4lIERlbHRhX0hOTUZfd2hpcHBldF9NRV9hZ2VfZGlmZl90b3RhbCRleG9uX0lEXVsgTUUuemVicmEgJWluJSBleG9ucy56ZWJyYSRWNF0KCiAKCmdncGxvdChNb3VzZV9aZWJyYS5JRHMpICsKICBnZW9tX2JhcihhZXMoeD1NRV9jbHVzdGVyLm5hbWUsIGZpbGw9dHlwZSksIHN0YXQ9ImNvdW50IiwgcG9zaXRpb249ImZpbGwiKQoKCmxlbmd0aCh1bmlxdWUoTW91c2VfWmVicmEuSURzJE1FLm1vdXNlKSkKCmxlbmd0aCh1bmlxdWUoTW91c2VfWmVicmEuSURzJE1FLnplYnJhKSkKClBQSV9kaWZmX2FnZV9ITk1GX2NlbnRyYWxbICwgY29uc2VydmVkOj1GQUxTRSBdClBQSV9kaWZmX2FnZV9ITk1GX2NlbnRyYWxbZXhvbl9JRCAlaW4lIE1vdXNlX1plYnJhLklEcyRNRS5tb3VzZSwgY29uc2VydmVkOj1UUlVFIF0KCmdncGxvdChQUElfZGlmZl9hZ2VfSE5NRl9jZW50cmFsKSArCiAgZ2VvbV9qaXR0ZXIoYWVzKGhhcm1vbmljX2NlbnRyYWxpdHksIGRpZmZfYWdlLCAgc2hhcGU9IHR5cGUsIGNvbG91cj1jb25zZXJ2ZWQgICksIHdpZHRoPTAsIGhlaWdodD0wLjEpICsgCiAgeGxhYigiSGFybW9uaWMgQ2VudHJhbGl0eSIpICsKICB5bGFiKCJFYXJsaWVzdCBtaWNyb2V4b24gZGlmZmVyZW50aWFsIGluY2x1c2lvbiBkZXRlY3Rpb24gKERQQykiKSArCiAgIHNjYWxlX3lfY29udGludW91cyhicmVha3M9c2VxKDEwLjUsMTYuNSwxKSkgKwogICAgZ2VvbV90ZXh0X3JlcGVsKGRhdGE9UFBJX2RpZmZfYWdlX0hOTUZfY2VudHJhbFsgIGNlbnRyYWw9PVRSVUUgIF0sIAogICAgICAgICAgICAgICAgICBhZXMoeD1oYXJtb25pY19jZW50cmFsaXR5LCB5PWRpZmZfYWdlLCAgY29sb3VyPWNvbnNlcnZlZCAgKSwgCiAgICAgICAgICAgICAgICAgIG51ZGdlX3kgICAgICA9IDAuNSwKICAgICAgICAgICAgICAgICAgZGlyZWN0aW9uICAgID0gIngiLAogICAgICAgICAgICAgICAgICBhbmdsZSAgICAgICAgPSA5MCwKICAgICAgICAgICAgICAgICAgc2VnbWVudC5zaXplID0gMC4yLAogICAgICAgICAgICAgICAgICBsYWJlbD1QUElfZGlmZl9hZ2VfSE5NRl9jZW50cmFsW2NlbnRyYWw9PVRSVUUgICwgbWFwcGVkX2dlbmVdLAogICAgICAgICAgICAgICAgICBzaG93LmxlZ2VuZCA9IEZBTFNFKSArCiAgICAgICAgICB0aGVtZShheGlzLnRleHQueCA9IGVsZW1lbnRfdGV4dChhbmdsZSA9IDkwKSwgbGVnZW5kLnBvc2l0aW9uID0gInRvcCIsIGxlZ2VuZC5kaXJlY3Rpb24gPSAiaG9yaXpvbnRhbCIpCgoKZ2dwbG90KFBQSV9kaWZmX2FnZV9ITk1GX2NlbnRyYWwpICsKICBnZW9tX2JveHBsb3QoYWVzKGNvbnNlcnZlZCwgbG9nMihiZXR3ZWVubmVzcykgKSkKCmBgYAoKCmBgYHtyfQpNb3VzZV9aZWJyYS5JRHNbZW5zZW1ibGVfemVicmE9PUZBTFNFXQoKCnplYnJhLnplYnJhIDwtICBnZ3Bsb3QoTW91c2VfWmVicmEuSURzKSArIAogIGdlb21fYmFyKGFlcyhNRV9jbHVzdGVyLm5hbWUsIGZpbGw9ZW5zZW1ibGVfemVicmEpLCBzdGF0PSJjb3VudCIgKSArCiAgICB0aGVtZShheGlzLnRleHQueCA9IGVsZW1lbnRfdGV4dChhbmdsZSA9IDQ1KSwgbGVnZW5kLnBvc2l0aW9uID0gInRvcCIsIGxlZ2VuZC5kaXJlY3Rpb24gPSAiaG9yaXpvbnRhbCIpCgoKemVicmEubW91c2UgPC0gZ2dwbG90KE1vdXNlX1plYnJhLklEcykgKyAKICBnZW9tX2JhcihhZXMoTUVfY2x1c3Rlci5uYW1lLCBmaWxsPWVuc2VtYmxlX21vdXNlKSwgc3RhdD0iY291bnQiKSArCiAgICB0aGVtZShheGlzLnRleHQueCA9IGVsZW1lbnRfdGV4dChhbmdsZSA9IDQ1KSwgbGVnZW5kLnBvc2l0aW9uID0gInRvcCIsIGxlZ2VuZC5kaXJlY3Rpb24gPSAiaG9yaXpvbnRhbCIpCiAgCmBgYAoKYGBge3IsIH0KCgoKTW91c2VfWmVicmEuSURzLmNsdXN0ZXJfbmFtZXMgPC0gbWVyZ2UoTW91c2VfWmVicmEuSURzLCBNRV9jbHVzdGVyX25hbWVzWywgYygiTUVfY2x1c3Rlci5uYW1lIiwgIk1FX2NsdXN0ZXIudHlwZSIpXSwgYnk9Ik1FX2NsdXN0ZXIubmFtZSIpCk1vdXNlX1plYnJhX3N0YXRzICA8LSBNb3VzZV9aZWJyYS5JRHMuY2x1c3Rlcl9uYW1lc1sgLCAuTiAsIGJ5PWMoIk1FX2NsdXN0ZXIudHlwZSIsICJlbnNlbWJsZV96ZWJyYSIsICJlbnNlbWJsZV9tb3VzZSIpXQoKTW91c2VfWmVicmFfc3RhdHNbICwgVHlwZTo9cGFzdGUoZW5zZW1ibGVfemVicmEsIGVuc2VtYmxlX21vdXNlLCBzZXAgPSAiXyIpIF0KCk1vdXNlX1plYnJhX3N0YXRzJE1FX2NsdXN0ZXIudHlwZSA8LSBmYWN0b3IoTW91c2VfWmVicmFfc3RhdHMkTUVfY2x1c3Rlci50eXBlLCAgbGV2ZWxzPU1vdXNlX1plYnJhX3N0YXRzWyFlbnNlbWJsZV96ZWJyYSAgfCAhZW5zZW1ibGVfbW91c2UgLCBzdW0oTiksIGJ5PWMoIk1FX2NsdXN0ZXIudHlwZSIpXVtvcmRlcihWMSksIE1FX2NsdXN0ZXIudHlwZV0pCgogIAogIAoKRmlnNy5FIDwtIGdncGxvdChNb3VzZV9aZWJyYV9zdGF0c1shZW5zZW1ibGVfemVicmEgIHwgIWVuc2VtYmxlX21vdXNlXSkgKwogIGdlb21fYmFyKGFlcyh4PU1FX2NsdXN0ZXIudHlwZSwgeT1OLCBmaWxsPVR5cGUpLCBzdGF0ID0gImlkZW50aXR5IiwgcG9zaXRpb249InN0YWNrIiAgKSArIAogIHlsYWIoIk51bWJlciBvZiBtaWNyb2V4b25zIikgKwogIHhsYWIoIk1pY3JvZXhvbiBjbHVzdGVycyIpICsgCiAgIHNjYWxlX2ZpbGxfZGlzY3JldGUobmFtZSA9ICJUeXBlIiwgbGFiZWxzID0gYygiTWlzc2luZyBpbiBib3RoIiwgIk1pc3NpbmcgaW4gemVicmFmaXNoIiwgIk1pc3NpbmcgaW4gbW91c2UiKSkgKwogICAgICAgICAgICB0aGVtZShheGlzLnRleHQueCA9IGVsZW1lbnRfdGV4dChhbmdsZSA9IDkwKSwgbGVnZW5kLnBvc2l0aW9uID0gInRvcCIsIGxlZ2VuZC5kaXJlY3Rpb24gPSAiaG9yaXpvbnRhbCIpICsKICAgIHRoZW1lX2J3KCkgKyAKICB0aGVtZShheGlzLnRleHQueCA9IGVsZW1lbnRfdGV4dChhbmdsZSA9IDkwKSkKCgpGaWc3LkUKCmBgYAoKCmBgYHtyfQpsZW5ndGgodW5pcXVlKE1vdXNlX1plYnJhLklEcyRNRS5tb3VzZSkpCmxlbmd0aCh1bmlxdWUoTW91c2VfWmVicmEuSURzJE1FLnplYnJhKSkKCnJvdW5kKChNb3VzZV9aZWJyYV9zdGF0c1tlbnNlbWJsZV9tb3VzZT09RkFMU0UsIHN1bShOKSBdICogMTAwKSAvIGxlbmd0aCh1bmlxdWUoTW91c2VfWmVicmEuSURzJE1FLm1vdXNlKSksIDEpCnJvdW5kKChNb3VzZV9aZWJyYV9zdGF0c1tlbnNlbWJsZV96ZWJyYT09RkFMU0UsIHN1bShOKSBdICogMTAwICkgLyBsZW5ndGgodW5pcXVlKE1vdXNlX1plYnJhLklEcyRNRS56ZWJyYSkpLCAxKQpgYGAKCgoKYGBge3J9CmxpYnJhcnkoZ2dzaWduaWYpCgojTW91c2VfWmVicmEuSURzWywgTUVfY2x1c3Rlci5uYW1lOj1NRV9jbHVzdGVyLm5hbWUueF0KCk1vdXNlX1plYnJhLmNvbnNlcnZlZF9mcmFjIDwtIHVuaXF1ZShNb3VzZV9aZWJyYS5JRHNbLCBjKCJNRS5tb3VzZSIsICJNRV9jbHVzdGVyLm5hbWUiKV0pWyAsLk4gLCBieT1NRV9jbHVzdGVyLm5hbWUgXQoKTW91c2VfWmVicmEuY29uc2VydmVkX2ZyYWMgPC0gIG1lcmdlKCBNb3VzZV9aZWJyYS5jb25zZXJ2ZWRfZnJhYyAsIE1FX2NsdXN0ZXJzLm5hbWVzWyAsIC4oVG90YWw9Lk4pICwgYnk9TUVfY2x1c3Rlci5uYW1lXSwgYnk9Ik1FX2NsdXN0ZXIubmFtZSIpCgpNb3VzZV9aZWJyYS5jb25zZXJ2ZWRfZnJhY1ssIHBlcmNldGFnZTo9KE4qMTAwL1RvdGFsKV0KCk1vdXNlX1plYnJhLmNvbnNlcnZlZF9mcmFjW29yZGVyKHBlcmNldGFnZSldCgpNb3VzZV9aZWJyYS5jb25zZXJ2ZWRfZnJhYyA8LSBtZXJnZShNb3VzZV9aZWJyYS5jb25zZXJ2ZWRfZnJhYywgTUVfY2x1c3Rlcl9uYW1lc1ssIGMoIk1FX2NsdXN0ZXIubmFtZSIsICJNRV9jbHVzdGVyLnR5cGUiKV0sIGJ5PSJNRV9jbHVzdGVyLm5hbWUiICkKCk1vdXNlX1plYnJhLmNvbnNlcnZlZF9mcmFjWywgYnJvYWRfY2xhc3M6PSJPdGhlciJdCk1vdXNlX1plYnJhLmNvbnNlcnZlZF9mcmFjW01FX2NsdXN0ZXIudHlwZSAlaW4lIGMoIk5ldXJvbmFsIiwgIk5ldXJvLU11c2N1bGFyIiwgIk5vbi1OZXVyb25hbCIsICJXZWFrLU5ldXJvbmFsIiksIGJyb2FkX2NsYXNzOj0iTmV1cm9uYWwiXQpNb3VzZV9aZWJyYS5jb25zZXJ2ZWRfZnJhY1tNRV9jbHVzdGVyLnR5cGUgJWluJSBjKCJOZXVyby1NdXNjdWxhciIpLCBicm9hZF9jbGFzczo9Ik5ldXJvLU11c2N1bGFyIl0KCkZpZzcuRCA8LSBnZ3Bsb3QoTW91c2VfWmVicmEuY29uc2VydmVkX2ZyYWMsIGFlcyh4ID0gYnJvYWRfY2xhc3MsICB5ID0gcGVyY2V0YWdlICkpICsKICBnZW9tX2ppdHRlciggYWVzKCBjb2xvdXIgPSBNRV9jbHVzdGVyLnR5cGUpLCB3aWR0aD0wLjEpICsKICBnZW9tX3NpZ25pZihjb21wYXJpc29ucyA9IGxpc3QoYygiTmV1cm9uYWwiLCAiT3RoZXIiKSksIG1hcF9zaWduaWZfbGV2ZWw9VFJVRSwgdGVzdD0id2lsY294LnRlc3QiLCBtYXJnaW5fdG9wPTUsICB5X3Bvc2l0aW9uPTM1LCB0ZXN0LmFyZ3MgPSBsaXN0KGFsdGVybmF0aXZlID0gInR3by5zaWRlZCIsIHBhaXJlZCA9IEZBTFNFKSkgKwogIHhsYWIoIk1pY3JvZXhvbiBjbHVzdGVyIHdpdGggbmV1cm9uYWwgcGF0dGVybiIpICsKICB5bGFiKCJQZXJjZW50YWdlIG9mIGNvbnNlcnZlZCBtaWNyb2V4b25zIikgKwogIHRoZW1lX2J3KCkgKyAKICB0aGVtZShheGlzLnRleHQueCA9IGVsZW1lbnRfdGV4dChhbmdsZSA9IDkwKSkKCkZpZzcuRAoKYGBgCgoKRXhwbG9yaW5nIGxpZnQgemVicmEgdGhhdCBkbyBub3Qgb3ZlcmxhcCB3aXRoIG1vdXNlIG1pY3JvZXhvbnMKCmBgYHtyfQp6ZWJyYS50b3RhbF9saWZ0IDwtIGZyZWFkKCIuLi8uLi9aZWJyYWZpc2gvQ29uc2VydmVkL291dC5oaWdoX3F1YWxpdHkuYmVkLmxpZnRvdmVyXzAuMDAxX21tMTAiKQoKemVicmEudG90YWxfbGlmdFshVjQgJWluJSAgemVicmFfdG9fbW91c2UkVjQsIF0KYGBgCgoKCmBgYHtyLCBmaWcud2lkdGg9NSwgZmlnLmhlaWdodD01fQoKcGxvdF9ncmlkKEZpZzcuRCwgRmlnNy5FLCBuY29sPTEpCgpgYGAKCgoKIyMjIyBaZWJyYSBkaWZmCgoKYGBge3J9CgoKZGlmZi5XLmZpbGUgPC0gICIuLi8uLi9aZWJyYWZpc2gvRmluYWxfUmVwb3J0L1doaXBwZXQvRGVsdGEvMTZNb192c180TW8tVE9UQUwuZGlmZi5taWNyb2V4b25zIgpkaWZmLk1FLmZpbGUgPC0gIi4uLy4uL1plYnJhZmlzaC9GaW5hbF9SZXBvcnQvV2hpcHBldC9EZWx0YS8xNk1vX3ZzXzRNby1UT1RBTC5kaWZmLk1FLm1pY3JvZXhvbnMiCmNvbXBfbmFtZSA9ICIxNk1vX3ZzXzRNby1UT1RBTCIKCgpkaWZmX3plYnJhIDwtIGZ1bmN0aW9uKGRpZmYuVy5maWxlLCBkaWZmLk1FLmZpbGUsIGNvbXBfbmFtZSkgewoKCmRpZmYuVyA8LSBmcmVhZChkaWZmLlcuZmlsZSkKZGlmZi5NRSA8LSBmcmVhZChkaWZmLk1FLmZpbGUpCgpkaWZmLldNRSA8LSBtZXJnZShkaWZmLlcsIGRpZmYuTUUsIGJ5PWMoImV4b25fSUQiLCAiR2VuZSIsICJOb2RlIiwgICJDb29yZCIsICJTdHJhbmQiLCAiVHlwZSIpKQoKZGlmZi5XTUVbLCBOYW1lOj1jb21wX25hbWVdCgpkaWZmLldNRVsgLCBkaWZmOj0iTkEiXQpkaWZmLldNRVsgKERlbHRhUHNpLng+PTAuMSAmICBQcm9iYWJpbGl0eS54ID49IDAuOSkgJiAgKERlbHRhUHNpLnk+PTAuMSAmICBQcm9iYWJpbGl0eS55ID49IDAuOSksIGRpZmY6PSJJbmNsdWRlZCJdCmRpZmYuV01FWyAoRGVsdGFQc2kueDw9LTAuMSAmICBQcm9iYWJpbGl0eS54ID49IDAuOSkgJiAgKERlbHRhUHNpLnk8PS0wLjEgJiAgUHJvYmFiaWxpdHkueSA+PSAwLjkpICAsIGRpZmY6PSJFeGNsdWRlZCIgXQoKcmV0dXJuKGRpZmYuV01FKQp9CmBgYAoKCgpgYGB7cn0KWmVicmEuMTZNb192c180TW8uVE9UQUwgPC0gZGlmZl96ZWJyYSgiLi4vLi4vWmVicmFmaXNoL0ZpbmFsX1JlcG9ydC9XaGlwcGV0L0RlbHRhLzE2TW9fdnNfNE1vLVRPVEFMLmRpZmYubWljcm9leG9ucyIsCiAgICAgICAgICAgICAgICAgICAgICAgICAgICAgICAgIi4uLy4uL1plYnJhZmlzaC9GaW5hbF9SZXBvcnQvV2hpcHBldC9EZWx0YS8xNk1vX3ZzXzRNby1UT1RBTC5kaWZmLk1FLm1pY3JvZXhvbnMiLCAKICAgICAgICAgICAgICAgICAgICAgICAgICAgICAgICAiMTZNb192c180TW8tVE9UQUwiKQoKCgpaZWJyYS4yME1vX3ZzXzE2TW8uWlQxNiA8LSBkaWZmX3plYnJhKCIuLi8uLi9aZWJyYWZpc2gvRmluYWxfUmVwb3J0L1doaXBwZXQvRGVsdGEvMjBNb192c18xNk1vLVpUMTYuZGlmZi5taWNyb2V4b25zIiwKICAgICAgICAgICAgICAgICAgICAgICAgICAgICAgICAiLi4vLi4vWmVicmFmaXNoL0ZpbmFsX1JlcG9ydC9XaGlwcGV0L0RlbHRhLzIwTW9fdnNfMTZNby1aVDE2LmRpZmYuTUUubWljcm9leG9ucyIsIAogICAgICAgICAgICAgICAgICAgICAgICAgICAgICAgICIyME1vX3ZzXzE2TW8tWlQxNiIpCgoKWmVicmEuMjBNb192c180TS5aVDE2IDwtIGRpZmZfemVicmEoIi4uLy4uL1plYnJhZmlzaC9GaW5hbF9SZXBvcnQvV2hpcHBldC9EZWx0YS8yME1vX3ZzXzRNLVpUMTYuZGlmZi5taWNyb2V4b25zIiwKICAgICAgICAgICAgICAgICAgICAgICAgICAgICAgICAiLi4vLi4vWmVicmFmaXNoL0ZpbmFsX1JlcG9ydC9XaGlwcGV0L0RlbHRhLzIwTW9fdnNfNE0tWlQxNi5kaWZmLk1FLm1pY3JvZXhvbnMiLCAKICAgICAgICAgICAgICAgICAgICAgICAgICAgICAgICAiMjBNb192c180TS1aVDE2IikKCgoKWmVicmEuMTZNb192c180TW8uWlQxNiA8LSBkaWZmX3plYnJhKCIuLi8uLi9aZWJyYWZpc2gvRmluYWxfUmVwb3J0L1doaXBwZXQvRGVsdGEvMTZNb192c180TW8tWlQxNi5kaWZmLm1pY3JvZXhvbnMiLAogICAgICAgICAgICAgICAgICAgICAgICAgICAgICAgICIuLi8uLi9aZWJyYWZpc2gvRmluYWxfUmVwb3J0L1doaXBwZXQvRGVsdGEvMTZNb192c180TW8tWlQxNi5kaWZmLk1FLm1pY3JvZXhvbnMiLCAKICAgICAgICAgICAgICAgICAgICAgICAgICAgICAgICAiMTZNb192c180TW8tWlQxNiIpCgoKClplYnJhLjIwTW9fdnNfMTZNby5aVDQgPC0gZGlmZl96ZWJyYSgiLi4vLi4vWmVicmFmaXNoL0ZpbmFsX1JlcG9ydC9XaGlwcGV0L0RlbHRhLzIwTW9fdnNfMTZNby1aVDQuZGlmZi5taWNyb2V4b25zIiwKICAgICAgICAgICAgICAgICAgICAgICAgICAgICAgICAiLi4vLi4vWmVicmFmaXNoL0ZpbmFsX1JlcG9ydC9XaGlwcGV0L0RlbHRhLzIwTW9fdnNfMTZNby1aVDQuZGlmZi5NRS5taWNyb2V4b25zIiwgCiAgICAgICAgICAgICAgICAgICAgICAgICAgICAgICAgIjIwTW9fdnNfMTZNby1aVDQiKQoKClplYnJhLjIwTW9fdnNfNE0uWlQ0IDwtIGRpZmZfemVicmEoIi4uLy4uL1plYnJhZmlzaC9GaW5hbF9SZXBvcnQvV2hpcHBldC9EZWx0YS8yME1vX3ZzXzRNLVpUNC5kaWZmLm1pY3JvZXhvbnMiLAogICAgICAgICAgICAgICAgICAgICAgICAgICAgICAgICIuLi8uLi9aZWJyYWZpc2gvRmluYWxfUmVwb3J0L1doaXBwZXQvRGVsdGEvMjBNb192c180TS1aVDQuZGlmZi5NRS5taWNyb2V4b25zIiwgCiAgICAgICAgICAgICAgICAgICAgICAgICAgICAgICAgIjIwTW9fdnNfNE0tWlQ0IikKCgoKWmVicmEuMTZNb192c180TW8uWlQ0IDwtIGRpZmZfemVicmEoIi4uLy4uL1plYnJhZmlzaC9GaW5hbF9SZXBvcnQvV2hpcHBldC9EZWx0YS8xNk1vX3ZzXzRNby1aVDQuZGlmZi5taWNyb2V4b25zIiwKICAgICAgICAgICAgICAgICAgICAgICAgICAgICAgICAiLi4vLi4vWmVicmFmaXNoL0ZpbmFsX1JlcG9ydC9XaGlwcGV0L0RlbHRhLzE2TW9fdnNfNE1vLVpUNC5kaWZmLk1FLm1pY3JvZXhvbnMiLCAKICAgICAgICAgICAgICAgICAgICAgICAgICAgICAgICAiMTZNb192c180TW8tWlQ0IikKCgoKClplYnJhLlpUMTZfdnNfWlQ0LjIwTSA8LSBkaWZmX3plYnJhKCIuLi8uLi9aZWJyYWZpc2gvRmluYWxfUmVwb3J0L1doaXBwZXQvRGVsdGEvWlQxNl92c19aVDQtMjBNby5kaWZmLm1pY3JvZXhvbnMiLAogICAgICAgICAgICAgICAgICAgICAgICAgICAgICAgICIuLi8uLi9aZWJyYWZpc2gvRmluYWxfUmVwb3J0L1doaXBwZXQvRGVsdGEvWlQxNl92c19aVDQtMjBNby5kaWZmLk1FLm1pY3JvZXhvbnMiLCAKICAgICAgICAgICAgICAgICAgICAgICAgICAgICAgICAiWlQxNl92c19aVDQtMjBNbyIpCgpaZWJyYS5aVDE2X3ZzX1pUNC4xNk0gPC0gZGlmZl96ZWJyYSgiLi4vLi4vWmVicmFmaXNoL0ZpbmFsX1JlcG9ydC9XaGlwcGV0L0RlbHRhL1pUMTZfdnNfWlQ0LTE2TW8uZGlmZi5taWNyb2V4b25zIiwKICAgICAgICAgICAgICAgICAgICAgICAgICAgICAgICAiLi4vLi4vWmVicmFmaXNoL0ZpbmFsX1JlcG9ydC9XaGlwcGV0L0RlbHRhL1pUMTZfdnNfWlQ0LTE2TW8uZGlmZi5NRS5taWNyb2V4b25zIiwgCiAgICAgICAgICAgICAgICAgICAgICAgICAgICAgICAgIlpUMTZfdnNfWlQ0LTE2TW8iKQoKWmVicmEuWlQxNl92c19aVDQuNE0gPC0gZGlmZl96ZWJyYSgiLi4vLi4vWmVicmFmaXNoL0ZpbmFsX1JlcG9ydC9XaGlwcGV0L0RlbHRhL1pUMTZfdnNfWlQ0LTRNby5kaWZmLm1pY3JvZXhvbnMiLAogICAgICAgICAgICAgICAgICAgICAgICAgICAgICAgICIuLi8uLi9aZWJyYWZpc2gvRmluYWxfUmVwb3J0L1doaXBwZXQvRGVsdGEvWlQxNl92c19aVDQtNE1vLmRpZmYuTUUubWljcm9leG9ucyIsIAogICAgICAgICAgICAgICAgICAgICAgICAgICAgICAgICJaVDE2X3ZzX1pUNC00TW8iKQoKCgoKWmVicmEuVE9UQUxfZGlmZiA8LSByYmluZCgKWmVicmEuMjBNb192c18xNk1vLlpUMTZbZGlmZiE9Ik5BIl0sClplYnJhLjIwTW9fdnNfNE0uWlQxNltkaWZmIT0iTkEiXSwKWmVicmEuMTZNb192c180TW8uWlQxNltkaWZmIT0iTkEiXSwKWmVicmEuMjBNb192c18xNk1vLlpUNFtkaWZmIT0iTkEiXSwKWmVicmEuMjBNb192c180TS5aVDRbZGlmZiE9Ik5BIl0sClplYnJhLjE2TW9fdnNfNE1vLlpUNFtkaWZmIT0iTkEiXSwKWmVicmEuWlQxNl92c19aVDQuMjBNW2RpZmYhPSJOQSJdLApaZWJyYS5aVDE2X3ZzX1pUNC4xNk1bZGlmZiE9Ik5BIl0sClplYnJhLlpUMTZfdnNfWlQ0LjRNW2RpZmYhPSJOQSJdICkKCgpaZWJyYS5UT1RBTF9kaWZmIDwtIFplYnJhLlRPVEFMX2RpZmZbICwgbGFwcGx5KC5TRCwgcGFzdGUwLCBjb2xsYXBzZT0iLCIpICwgYnk9ImV4b25fSUQiXQoKCk1vdXNlX1plYnJhLklEc1tNRS56ZWJyYSAlaW4lIFplYnJhLlRPVEFMX2RpZmYkZXhvbl9JRF0KCmBgYAoKCgpgYGB7cn0KRGVsdGFfSE5NX3doaXBwZXRfTUUgPC0gbWVyZ2UoRGVsdGFfSE5NX3doaXBwZXQsIERlbHRhX0hOTV9NRSwgYnk9YygiZXhvbl9JRCIsICJHZW5lIiwgIk5vZGUiLCAgIkNvb3JkIiwgIlN0cmFuZCIsICJUeXBlIiwgImFnZSIpICkKCkRlbHRhX0hOTV93aGlwcGV0X01FWyAoYWJzKERlbHRhUHNpLngpPj0wLjEgJiAgUHJvYmFiaWxpdHkueCA+PSAwLjkpICYgIChhYnMoRGVsdGFQc2kueSk+PTAuMSAmICBQcm9iYWJpbGl0eS55ID49IDAuOSkgLCAuTiAsIGJ5PShhZ2UpXQoKRGVsdGFfSE5NX3doaXBwZXRfTUVfaW5jbHVkZWQgPC0gRGVsdGFfSE5NX3doaXBwZXRfTUVbIChEZWx0YVBzaS54PD0tMC4xICYgIFByb2JhYmlsaXR5LnggPj0gMC45KSAmICAoRGVsdGFQc2kueTw9LTAuMSAmICBQcm9iYWJpbGl0eS55ID49IDAuOSkgICwgLk4gLCBieT0oYWdlKV0KRGVsdGFfSE5NX3doaXBwZXRfTUVfZXhjbHVkZWQgPC0gRGVsdGFfSE5NX3doaXBwZXRfTUVbIChEZWx0YVBzaS54Pj0wLjEgJiAgUHJvYmFiaWxpdHkueCA+PSAwLjkpICYgIChEZWx0YVBzaS55Pj0wLjEgJiAgUHJvYmFiaWxpdHkueSA+PSAwLjkpICAsIC5OICwgYnk9KGFnZSldCgoKRGVsdGFfSE5NX2luY2x1ZGVkX3N0YXRzIDwtIGNiaW5kKERlbHRhX0hOTV93aGlwcGV0X2luY2x1ZGVkW29yZGVyKGFnZSldLCBEZWx0YV9ITk1fTUVfaW5jbHVkZWRbb3JkZXIoYWdlKSwgMl0sIERlbHRhX0hOTV93aGlwcGV0X01FX2luY2x1ZGVkW29yZGVyKGFnZSksIDJdKQpjb2xuYW1lcyhEZWx0YV9ITk1faW5jbHVkZWRfc3RhdHMpIDwtIGMoImFnZSIsICJXaGlwcGV0IiwgIk1pY3JvRXhvbmF0b3IiLCAiQm90aCIpCgpgYGAKCgoKCiMjIyMjCgoKYGBge3J9CgpjZW50cmFsaXR5X2xvZ2lzdGljX3JlZ3Jlc3Npb25fSEMgPC0gZ2xtKGZvcm11bGEgPSBjZW50cmFsIH4gbWluX2RpZl9hZ2UsIGZhbWlseSA9ICJiaW5vbWlhbCIsIGRhdGEgPSBQUElfZGlmZl9hZ2VfSE5NX3N0YXRzX0hDKQpzdW1tYXJ5KGNlbnRyYWxpdHlfbG9naXN0aWNfcmVncmVzc2lvbl9IQykKCgpjZW50cmFsaXR5X2xvZ2lzdGljX3JlZ3Jlc3Npb25fRUMgPC0gZ2xtKGZvcm11bGEgPSBjZW50cmFsIH4gbWluX2RpZl9hZ2UsIGZhbWlseSA9ICJiaW5vbWlhbCIsIGRhdGEgPSBQUElfZGlmZl9hZ2VfSE5NX3N0YXRzX0VDKQpzdW1tYXJ5KGNlbnRyYWxpdHlfbG9naXN0aWNfcmVncmVzc2lvbl9FQykKCgpjZW50cmFsaXR5X2xvZ2lzdGljX3JlZ3Jlc3Npb25fQiA8LSBnbG0oZm9ybXVsYSA9IGNlbnRyYWwgfiBtaW5fZGlmX2FnZSwgZmFtaWx5ID0gImJpbm9taWFsIiwgZGF0YSA9IFBQSV9kaWZmX2FnZV9ITk1fc3RhdHNfQikKc3VtbWFyeShjZW50cmFsaXR5X2xvZ2lzdGljX3JlZ3Jlc3Npb25fQikKYGBgCgoKYGBge3J9CgpQUElfZGlmZl9hZ2VfSE5NJEwxY2FtX2FuZF9pbnRlcmFjdG9ycyA8LSBmYWN0b3IoUFBJX2RpZmZfYWdlX0hOTSRMMWNhbV9hbmRfaW50ZXJhY3RvcnMsIGxldmVscz1jKFRSVUUsIEZBTFNFKSkKCgpGNkcgPC0gZ2dwbG90KFBQSV9kaWZmX2FnZV9ITk0pICsKICBnZW9tX3BvaW50KGFlcyhlaWdlbl9jZW50cmFsaXR5LCBiZXR3ZWVubmVzcywgY29sb3VyPUwxY2FtX2FuZF9pbnRlcmFjdG9ycykpICsKICBmYWNldF93cmFwKCAuIH4gZGlmZl9hZ2UsIG5jb2wgPSAyKSAgKwogICAgICBnZW9tX3RleHRfcmVwZWwoZGF0YT1QUElfZGlmZl9hZ2VfSE5NWyBMMWNhbV9hbmRfaW50ZXJhY3RvcnM9PVRSVUUsICBdLCAKICAgICAgICAgICAgICAgICAgYWVzKHg9ZWlnZW5fY2VudHJhbGl0eSwgeT1iZXR3ZWVubmVzcywgY29sb3VyPUwxY2FtX2FuZF9pbnRlcmFjdG9ycyApLCAKICAgICAgICAgICAgICAgICAgbnVkZ2VfeSAgICAgID0gMzAwMCwKICAgICAgICAgICAgICAgICAgZGlyZWN0aW9uICAgID0gIngiLAogICAgICAgICAgICAgICAgICBhbmdsZSAgICAgICAgPSA5MCwKICAgICAgICAgICAgICAgICAgc2VnbWVudC5zaXplID0gMC4yLAogICAgICAgICAgICAgICAgICBsYWJlbD1QUElfZGlmZl9hZ2VfSE5NW0wxY2FtX2FuZF9pbnRlcmFjdG9ycz09VFJVRSAgLCBtZ2lfc3ltYm9sXSwKICAgICAgICAgICAgICAgICAgc2hvdy5sZWdlbmQgPSBGQUxTRSkgKwogICAgICAgICAgdGhlbWUoYXhpcy50ZXh0LnggPSBlbGVtZW50X3RleHQoYW5nbGUgPSA5MCksIGxlZ2VuZC5wb3NpdGlvbiA9ICJ0b3AiLCBsZWdlbmQuZGlyZWN0aW9uID0gImhvcml6b250YWwiKSArCiAgICBzY2FsZV9maWxsX2Rpc2NyZXRlKG5hbWUgPSAiTDFDQU0gcGF0aHdheSIsIGxhYmVscyA9IGMoIk1lbWJlciIsICJOb24tbWVtYmVyIikpICAKCgoKCmBgYAoKCgoKCgoKCgpgYGB7cn0KY2VudHJhbGl0eV9sb2dpc3RpY19yZWdyZXNzaW9uIDwtIGdsbShmb3JtdWxhID0gY2VudHJhbCB+IG1pbl9kaWZfYWdlLCBmYW1pbHkgPSAiYmlub21pYWwiLCBkYXRhID0gUFBJX2RpZmZfYWdlX0hOTV9zdGF0cykKc3VtbWFyeShjZW50cmFsaXR5X2xvZ2lzdGljX3JlZ3Jlc3Npb24pCmBgYAoKCgoKYGBge3IsIGZpZy5oZWlnaHQ9NiwgZmlnLndpZHRoPTh9CiBnZ3Bsb3QoUFBJX2RpZmZfYWdlX0hOTSkgKwogIGdlb21faml0dGVyKGFlcyhsb2coYmV0d2Vlbm5lc3MpLCBkaWZmX2FnZSwgY29sb3I9dHlwZSApLCB3aWR0aD0wLCBoZWlnaHQ9MC4xKSArCiAgeGxhYigiQmV0d2Vlbm5lc3MiKSArCiAgeWxhYigiRWFybGllc3QgbWljcm9leG9uIGRpZmZlcmVudGlhbCBpbmNsdXNpb24gZGV0ZWN0aW9uIChEUEMpIikgKwogICBzY2FsZV95X2NvbnRpbnVvdXMoYnJlYWtzPXNlcSgxMC41LDE2LjUsMSkpICsKICAgIGdlb21fdGV4dF9yZXBlbChkYXRhPVBQSV9kaWZmX2FnZV9ITk1bICBsb2coYmV0d2Vlbm5lc3MpPjYsICBdLAogICAgICAgICAgICAgICAgICBhZXMoeD1sb2coYmV0d2Vlbm5lc3MpLCB5PWRpZmZfYWdlLCBjb2xvdXI9dHlwZSksIAogICAgICAgICAgICAgICAgICBudWRnZV95ICAgICAgPSAwLjUsCiAgICAgICAgICAgICAgICAgIGRpcmVjdGlvbiAgICA9ICJ4IiwKICAgICAgICAgICAgICAgICAgYW5nbGUgICAgICAgID0gOTAsCiAgICAgICAgICAgICAgICAgIHNlZ21lbnQuc2l6ZSA9IDAuMiwKICAgICAgICAgICAgICAgICAgc2hvdy5sZWdlbmQgPSBGQUxTRSwKICAgICAgICAgICAgICAgICAgbGFiZWw9UFBJX2RpZmZfYWdlX0hOTVtsb2coYmV0d2Vlbm5lc3MpPjYgICwgbWdpX3N5bWJvbF0gKSAKCgpgYGAKCgoKCiMjIFRhc2ljCgoKYGBge3J9CmVuc2VtYmwgPSB1c2VFbnNlbWJsKGJpb21hcnQ9ImVuc2VtYmwiLCBkYXRhc2V0PSJtbXVzY3VsdXNfZ2VuZV9lbnNlbWJsIiwgaG9zdCA9ICJ3d3cuZW5zZW1ibC5vcmciKQoKCk1FX2ZpbmFsLnRyYW5zY3JpcHQgPC0gZ3N1YigiXFwuLioiLCIiLCBNRV9maW5hbCR0cmFuc2NyaXB0KQoKTUVfZmluYWwkZW5zZW1ibF90cmFuc2NyaXB0X2lkIDwtIGdzdWIoIlxcLi4qIiwiIiwgTUVfZmluYWwkdHJhbnNjcmlwdCkgCgpnZW5lX3RhYmxlLlRPVEFMIDwtIGRhdGEudGFibGUoZ2V0Qk0oYXR0cmlidXRlcz1jKCdlbnNlbWJsX3RyYW5zY3JpcHRfaWQnLCAnZW5zZW1ibF9nZW5lX2lkJywgIm1naV9zeW1ib2wiKSxmaWx0ZXJzID0gJ2Vuc2VtYmxfdHJhbnNjcmlwdF9pZCcsIHZhbHVlcyA9IE1FX2ZpbmFsLnRyYW5zY3JpcHRbbmNoYXIoTUVfZmluYWwudHJhbnNjcmlwdCkhPTBdLCBtYXJ0ID0gZW5zZW1ibCwgICkpCgoKZ2VuZV90YWJsZS5UT1RBTCA8LSBtZXJnZShnZW5lX3RhYmxlLlRPVEFMLCBNRV9maW5hbFssIGMoImVuc2VtYmxfdHJhbnNjcmlwdF9pZCIsICJNRSIpXSwgYnk9ImVuc2VtYmxfdHJhbnNjcmlwdF9pZCIpCgoKCmBgYAoKCgoKYGBge3J9CgpQQ0FfbG9hZGluZ3NbLCBNRV9jbHVzdGVyOj1hcy5udW1lcmljKChhcy5jaGFyYWN0ZXIoTUVfY2x1c3RlcikpKV0KTUVfY2x1c3Rlcl9uYW1lc1ssIE1FX2NsdXN0ZXI6PWFzLm51bWVyaWMoKGFzLmNoYXJhY3RlcihNRV9jbHVzdGVyKSkpXQpNRV9jbHVzdGVycy5pbmZvIDwtIG1lcmdlKFBDQV9sb2FkaW5ncywgTUVfY2x1c3Rlcl9uYW1lcywgYnk9Ik1FX2NsdXN0ZXIiKQoKTUVfY2x1c3RlcnMuaW5mb1tNRV9jbHVzdGVyLnR5cGU9PSJOZXVyb25hbCIgfCBNRV9jbHVzdGVyLnR5cGU9PSJOZXVyb211c2N1bGFyIl0KCgpsaWJyYXJ5KHJlYWRyKQoKbGlicmFyeShIbWlzYykKCgpUYXNpY19zYW1wbGVfaW5mbyA8LSBkYXRhLnRhYmxlKHJlYWRfZGVsaW0oIn4vR29vZ2xlX0RyaXZlL1Jlc3VsdHMvTUUvU2luZ2xlX2NlbGwvVGFzaWMvR1NFNzE1ODUudHh0IiwgCiAgICAiXHQiLCBlc2NhcGVfZG91YmxlID0gRkFMU0UsIHRyaW1fd3MgPSBUUlVFKSkKCgpUYXNpY19jbHVzdGVyaW5nIDwtIGRhdGEudGFibGUocmVhZF9kZWxpbSgifi9Hb29nbGVfRHJpdmUvUmVzdWx0cy9NRS9TaW5nbGVfY2VsbC9UYXNpYy9HU0U3MTU4NV9DbHVzdGVyaW5nX1Jlc3VsdHMuY3N2IiwgCiAgICAiLCIsIGVzY2FwZV9kb3VibGUgPSBGQUxTRSwgdHJpbV93cyA9IFRSVUUpKQoKdGFzaWNfZXRfYWxfU1QzIDwtIGRhdGEudGFibGUocmVhZF9jc3YoIn4vR29vZ2xlX0RyaXZlL1Jlc3VsdHMvTUUvU2luZ2xlX2NlbGwvVGFzaWMvdGFzaWNfZXRfYWwuU1QzLmNzdiIpKQoKCgp0YXNpY19tZXRhZGF0YSA8LSBtZXJnZShUYXNpY19zYW1wbGVfaW5mbywgdGFzaWNfZXRfYWxfU1QzLCBieS54PSJTYW1wbGVfTmFtZV9zIiwgYnkueT0iR0VPIFNhbXBsZSBBY2Nlc3Npb24iKQoKI3Rhc2ljX21ldGFkYXRhIDwtIHRhc2ljX21ldGFkYXRhWywgYygiUnVuX3MiLCAiR0VPIFNhbXBsZSBUaXRsZSIpIF0KCgp0YXNpY19tZXRhZGF0YSA8LSBtZXJnZSggVGFzaWNfY2x1c3RlcmluZywgdGFzaWNfbWV0YWRhdGEsIGJ5Lng9InNhbXBsZV90aXRsZSIsIGJ5Lnk9IkdFTyBTYW1wbGUgVGl0bGUiKQoKCgoKCk1FX2Nvdl9maWx0ZXJlZF90YXNpYyA8LSBtZXJnZSggTUVfY292LCAKICAgICAgIHRhc2ljX21ldGFkYXRhWywgYygic2FtcGxlX3RpdGxlIiwgImJyb2FkX3R5cGUiLCAiY29yZV9pbnRlcm1lZGlhdGUiLCAicHJpbWFyeV90eXBlIiwgIlJ1bl9zIildLAogICAgICAgYnkueD0iRklMRV9OQU1FIiwgYnkueSA9ICJSdW5fcyIpCgoKTUVfY292X2ZpbHRlcmVkX3Rhc2ljIDwtICBNRV9jb3ZfZmlsdGVyZWRfdGFzaWNbTUUgJWluJSBNRV9maW5hbFssIE1FXSAgLCBdCgoKTUVfY292X2ZpbHRlcmVkX3Rhc2ljJHN1bV9NRV9jb3ZlcmFnZSA8LSAgc2FwcGx5KHN0cnNwbGl0KGFzLmNoYXJhY3RlcihNRV9jb3ZfZmlsdGVyZWRfdGFzaWMkTUVfY292ZXJhZ2VzKSwgIiwiLCBmaXhlZD1UKSwgZnVuY3Rpb24oeCkgc3VtKGFzLm51bWVyaWMoeCkpKQpNRV9jb3ZfZmlsdGVyZWRfdGFzaWMkc3VtX1NKX2NvdmVyYWdlIDwtICBzYXBwbHkoc3Ryc3BsaXQoYXMuY2hhcmFjdGVyKE1FX2Nvdl9maWx0ZXJlZF90YXNpYyRTSl9jb3ZlcmFnZXMpLCAiLCIsIGZpeGVkPVQpLCBmdW5jdGlvbih4KSBzdW0oYXMubnVtZXJpYyh4KSkpCgoKI01FX2Nvdl9maWx0ZXJlZF90YXNpY1ssIGA6PWAoIHRvdGFsX2Nvdl9hbHRlcm5hdGl2ZXNfNT1BbHQ1X2NvdmVyYWdlcywgdG90YWxfY292X2FsdGVybmF0aXZlc18zPUFsdDNfY292ZXJhZ2VzKV0KTUVfY292X2ZpbHRlcmVkX3Rhc2ljWyBBbHQ1X2NvdmVyYWdlcz09Ik5vbmUiICwgYDo9YCggQWx0NV9jb3ZlcmFnZXM9MCldCk1FX2Nvdl9maWx0ZXJlZF90YXNpY1sgQWx0M19jb3ZlcmFnZXM9PSJOb25lIiAsIGA6PWAoIEFsdDNfY292ZXJhZ2VzPTApXQpNRV9jb3ZfZmlsdGVyZWRfdGFzaWMkdG90YWxfY292X2FsdGVybmF0aXZlc181IDwtICBzYXBwbHkoc3Ryc3BsaXQoYXMuY2hhcmFjdGVyKE1FX2Nvdl9maWx0ZXJlZF90YXNpYyRBbHQ1X2NvdmVyYWdlcyksICIsIiwgZml4ZWQ9VCksIGZ1bmN0aW9uKHgpIHN1bShhcy5udW1lcmljKHgpKSkKTUVfY292X2ZpbHRlcmVkX3Rhc2ljJHRvdGFsX2Nvdl9hbHRlcm5hdGl2ZXNfMyA8LSAgc2FwcGx5KHN0cnNwbGl0KGFzLmNoYXJhY3RlcihNRV9jb3ZfZmlsdGVyZWRfdGFzaWMkQWx0M19jb3ZlcmFnZXMpLCAiLCIsIGZpeGVkPVQpLCBmdW5jdGlvbih4KSBzdW0oYXMubnVtZXJpYyh4KSkpCgpNRV9jb3ZfZmlsdGVyZWRfdGFzaWNfcHJpbWFyeV90eXBlIDwtIE1FX2Nvdl9maWx0ZXJlZF90YXNpY1ssIC4oc3VtX01FX2NvdmVyYWdlPXN1bShzdW1fTUVfY292ZXJhZ2UpLAogICAgICAgICAgICAgICAgICAgICAgICAgIHN1bV9TSl9jb3ZlcmFnZSA9c3VtKHN1bV9TSl9jb3ZlcmFnZSksCiAgICAgICAgICAgICAgICAgICAgICAgICAgdG90YWxfY292X2FsdGVybmF0aXZlc18zID0gc3VtKHRvdGFsX2Nvdl9hbHRlcm5hdGl2ZXNfMyksCiAgICAgICAgICAgICAgICAgICAgICAgICAgdG90YWxfY292X2FsdGVybmF0aXZlc181ID0gc3VtKHRvdGFsX2Nvdl9hbHRlcm5hdGl2ZXNfNSkKICAgICAgICAgICAgICAgICAgICAgICAgICApLCBieT1jKCJwcmltYXJ5X3R5cGUiLCAiYnJvYWRfdHlwZSIsICJNRSIpIF0KCgoKCgoKTUVfY292X2ZpbHRlcmVkX3Rhc2ljX3ByaW1hcnlfdHlwZVssUFNJOj0oc3VtX01FX2NvdmVyYWdlLyhzdW1fTUVfY292ZXJhZ2Urc3VtX1NKX2NvdmVyYWdlK3RvdGFsX2Nvdl9hbHRlcm5hdGl2ZXNfMyt0b3RhbF9jb3ZfYWx0ZXJuYXRpdmVzXzUpKV0KCm1pbl9QU0lfc3VtIDwtIDUKICAKTUVfY292X2ZpbHRlcmVkX3Rhc2ljX3ByaW1hcnlfdHlwZVtzdW1fTUVfY292ZXJhZ2Urc3VtX1NKX2NvdmVyYWdlK3RvdGFsX2Nvdl9hbHRlcm5hdGl2ZXNfMyt0b3RhbF9jb3ZfYWx0ZXJuYXRpdmVzXzU8bWluX1BTSV9zdW0sIFBTSTo9TmFOXQoKbGlicmFyeShIbWlzYyApCgpNRV9jb3ZfZmlsdGVyZWRfdGFzaWNfcHJpbWFyeV90eXBlWyAsIAogICAgICAgICAgICBgOj1gKAogICAgICAgICAgICAgIGxvd2VyPWJpbmNvbmYoeD1zdW1fTUVfY292ZXJhZ2UsIG49KHN1bV9NRV9jb3ZlcmFnZStzdW1fU0pfY292ZXJhZ2UrdG90YWxfY292X2FsdGVybmF0aXZlc18zK3RvdGFsX2Nvdl9hbHRlcm5hdGl2ZXNfNSkgKVssICJMb3dlciJdLAogICAgICAgICAgICAgIHVwcGVyPWJpbmNvbmYoeD1zdW1fTUVfY292ZXJhZ2UsIG49KHN1bV9NRV9jb3ZlcmFnZStzdW1fU0pfY292ZXJhZ2UrdG90YWxfY292X2FsdGVybmF0aXZlc18zK3RvdGFsX2Nvdl9hbHRlcm5hdGl2ZXNfNSkgKVssICJVcHBlciJdCiAgICAgICAgICAgICAgKSBdCgoKCk1FX2Nvdl9maWx0ZXJlZF90YXNpY19wcmltYXJ5X3R5cGUgPC0gbWVyZ2UoTUVfY292X2ZpbHRlcmVkX3Rhc2ljX3ByaW1hcnlfdHlwZSwgdW5pcXVlKGdlbmVfdGFibGUuVE9UQUwpLCBieT0iTUUiKQoKCk1FX2Nvdl9maWx0ZXJlZF90YXNpY19wcmltYXJ5X3R5cGUuTUVfY2x1c3RlcnMgPC0gbWVyZ2UoTUVfY292X2ZpbHRlcmVkX3Rhc2ljX3ByaW1hcnlfdHlwZSwgTUVfY2x1c3RlcnMuaW5mb1ssIGMoIk1FIiwgIk1FX2NsdXN0ZXIudHlwZSIsICJNRV9jbHVzdGVyLm5hbWUiKV0sIGJ5PSJNRSIpCgoKCgoKYGBgCgpgYGB7cn0KbGlicmFyeShzdHJpbmdyKQoKdGFibGUoZ3JlcGwoIkVOQyIsICB1bmlxdWUoTUVfY292X2ZpbHRlcmVkWywgRklMRV9OQU1FXSkpKQoKYGBgCgoKCgoKCmBgYHtyLCBmaWcud2lkdGg9MTUsICBmaWcuaGVpZ2h0PTIwfQoKCgoKCiNDN19pbnRlcmVzdGluZyA8LSBjKCJjaHIxXy1fOTMwMjY4MzFfOTMwMjY4NTUiLCAiY2hyMV8rXzExODUxMjcwNF8xMTg1MTI3MjgiLCAiY2hyMTFfLV8xMDYxODA5NTRfMTA2MTgwOTgzIiwgImNocjExXy1fMzM1Mjg5OV8zMzUyOTE3IiwgImNocjExXytfNTQ2NzE2NTRfNTQ2NzE2NzgiLCAiY2hyM18tXzE0ODgyNzIxMV8xNDg4MjcyMzgiLCIgY2hyNF8tXzc2MTM4NTU1Xzc2MTM4NTczIiwgImNocjVfK18xNDM3MDMzNzJfMTQzNzAzMzc1IiwgImNocjVfK180ODI2MzY0MF80ODI2MzY2NyIsICJjaHI4Xy1fNTQ2NDUzMzdfNTQ2NDUzNTgiKQoKCiNnZ3Bsb3QoTUVfY292X2ZpbHRlcmVkX3Rhc2ljX3ByaW1hcnlfdHlwZVsgTUUgJWluJSBDN19pbnRlcmVzdGluZywgXSAgKSArCiMgIGdlb21fcG9pbnRyYW5nZShhZXMoeD1wcmltYXJ5X3R5cGUsIHk9UFNJLCBjb2xvdXI9IGJyb2FkX3R5cGUsIGdyb3VwPWJyb2FkX3R5cGUsIHNoYXBlPWJyb2FkX3R5cGUsIHltaW49bG93ZXIsIHltYXg9dXBwZXIpKSsKIyAgdGhlbWUoYXhpcy50ZXh0LnggPSBlbGVtZW50X3RleHQoYW5nbGUgPSA5MCwgaGp1c3QgPSAwLCB2anVzdCA9IDAuNSkpICsKIyAgZmFjZXRfZ3JpZCggZ2VuZV9uYW1lc1tNRV0gfiAuICkKCgp0YXJnZXRfZ2VuZXMgPC0gYygiS2lmMWEiLCAiRGN0bjEiLCAiS2RtMWEiLCAiS2RtMmEiLCAgIkl0c24xIiwgIkRubTEiLCAiRG5tMyIsICJUcnJhcCIsICJEY2xrMiIpCgp0YXJnZXRfZ2VuZXMgPC0gYygiS2lmMWEiLCAiRGN0bjEiLCAiS2RtMWEiLCAiS2RtMmEiLCAgIkl0c24xIiwgIkRubTEiLCAiRG5tMyIsICJUcnJhcCIsICJEY2xrMiIpCgoKCmdncGxvdChNRV9jb3ZfZmlsdGVyZWRfdGFzaWNfcHJpbWFyeV90eXBlWyBtZ2lfc3ltYm9sICVpbiUgdGFyZ2V0X2dlbmVzICYgIE1FICVpbiUgRGVsdGFfSE5NRl93aGlwcGV0X01FX2FnZV9kaWZmX3RvdGFsJGV4b25fSUQgLCBdICApICsKICBnZW9tX3BvaW50cmFuZ2UoYWVzKHg9cHJpbWFyeV90eXBlLCB5PVBTSSwgY29sb3VyPSBicm9hZF90eXBlLCBncm91cD1icm9hZF90eXBlLCBzaGFwZT1icm9hZF90eXBlLCB5bWluPWxvd2VyLCB5bWF4PXVwcGVyKSkrCiAgZmFjZXRfZ3JpZCggcGFzdGUwKCBtZ2lfc3ltYm9sLCBNRSkgfiAuICkgKwogIHRoZW1lX2J3KCkgKwogIHRoZW1lKGF4aXMudGV4dC54ID0gZWxlbWVudF90ZXh0KGFuZ2xlID0gOTAsIGhqdXN0ID0gMCwgdmp1c3QgPSAwLjUpKQoKCgpnZ3Bsb3QoTUVfY292X2ZpbHRlcmVkX3Rhc2ljX3ByaW1hcnlfdHlwZVsgbWdpX3N5bWJvbCAlaW4lIHRhcmdldF9nZW5lcywgXSAgKSArCiAgZ2VvbV9wb2ludHJhbmdlKGFlcyh4PXByaW1hcnlfdHlwZSwgeT1QU0ksIGNvbG91cj0gYnJvYWRfdHlwZSwgZ3JvdXA9YnJvYWRfdHlwZSwgc2hhcGU9YnJvYWRfdHlwZSwgeW1pbj1sb3dlciwgeW1heD11cHBlcikpKwogIGZhY2V0X2dyaWQoIHBhc3RlMCggbWdpX3N5bWJvbCwgTUUpIH4gLiApICsKICB0aGVtZV9idygpICsKICB0aGVtZShheGlzLnRleHQueCA9IGVsZW1lbnRfdGV4dChhbmdsZSA9IDkwLCBoanVzdCA9IDAsIHZqdXN0ID0gMC41KSkKCmBgYAoKCgojIyMjIyMKCiMjIE5ldyBUYXNpYyAjIyMKCgpgYGB7cn0KClRhc2ljX3RvdGFsX2RpZmZfbm9kZXMgPC0gZnJlYWQoIi9Vc2Vycy9ncDcvR29vZ2xlX0RyaXZlL1Jlc3VsdHMvTUUvUGFwZXIvU2luZ2xlX2NlbGwvR0FCQS1lcmdpY19OZXVyb25fdnNfR2x1dGFtYXRlcmdpY19OZXVyb24uYWxsX25vZGVzLm1pY3JvZXhvbnMudHh0IikKCgoKVGFzaWNfdG90YWxfZGlmZl9ub2Rlcy5taWNyb2V4b25zIDwtIG1lcmdlKCBUYXNpY190b3RhbF9kaWZmX25vZGVzW2lzLmRpZmY9PSJUUlVFIiAmICFpcy5uYShtaWNyb2V4b25fSUQpLCBdLCAKICAgICAgICAgICAgICAgICAgICAgICAgICAgICAgICAgICAgICAgICAgICB1bmlxdWUoZ2VuZV90YWJsZS5UT1RBTFssIGMoIk1FIiwgIm1naV9zeW1ib2wiKV0pLAogICAgICAgICAgICAgICAgICAgICAgICAgICAgICAgICAgICAgICAgICAgIGJ5Lng9Im1pY3JvZXhvbl9JRCIsIGJ5Lnk9Ik1FIikKClRhc2ljX3RvdGFsX2RpZmZfbm9kZXMubWljcm9leG9uc1shbWljcm9leG9uX0lEICVpbiUgU2lnX0dBQkFlcmdpY19OZXVyb25fdnNfR2x1dGFtYXRlcmdpY19OZXVyb24kZXhvbl9JRCwgXQoKYGBgCgoKCmBgYHtyfQoKI3JlZiBTbGM0YTEwIGh0dHBzOi8vd3d3LmZyb250aWVyc2luLm9yZy9hcnRpY2xlcy8xMC4zMzg5L2ZuY2VsLjIwMTUuMDAyMjMvZnVsbAojcmVmIEFuazMgaHR0cHM6Ly93d3cuc2NpZW5jZWRpcmVjdC5jb20vc2NpZW5jZS9hcnRpY2xlL3BpaS9TMDg5NjYyNzMxNDAwOTA4OAojcmVmIEtDTk1BMSBodHRwczovL3d3dy5zY2llbmNlZGlyZWN0LmNvbS9zY2llbmNlL2FydGljbGUvcGlpL1MwODk2NjI3MzEzMDAxODUyCiNyZWYgS2lmM2EgaHR0cHM6Ly93d3cuc2NpZW5jZWRpcmVjdC5jb20vc2NpZW5jZS9hcnRpY2xlL3BpaS9TMDg5NjYyNzMwMzAwMDYyWAojIHJlZiBETEdBUCBodHRwczovL21vbGVjdWxhcmJyYWluLmJpb21lZGNlbnRyYWwuY29tL2FydGljbGVzLzEwLjExODYvczEzMDQxLTAxNy0wMzI0LTkKCgpQcmVfc3luYXB0aWMgPC0gYyggIlB0cHJkIiAsICJQcGZpYTIiLCAiRGxnYXAxIiwgIkdhYnJnMiIsICJLY25tYTEiLCAiS2lmM2EiLCAiQ2FkcHMiKSAKUG9zdF9zeW5hcHRpYyA8LSBjKCAiTnJ4bjMiICwgIk5yeG4xIikKUHJlUG9zdF9zeW5hcHRpYyA8LSBjKCJBbmszIiwiU2xjNGExMCIpCmBgYAoKCgoKCmBgYHtyfQpUYXNpY190b3RhbF9kaWZmX25vZGVzLm1pY3JvZXhvbnNbTG9jYXRpb25dCmBgYAoKCgpgYGB7ciwgZmlnLmhlaWdodD00LCBmaWcud2lkdGg9Nn0KCgoKZ2dwbG90KCkrCiAgZ2VvbV9wb2ludChkYXRhPVRhc2ljX3RvdGFsX2RpZmZfbm9kZXNbIWlzLm5hKG1pY3JvZXhvbl9JRCkgLCAgXSwgYWVzKERlbHRhUHNpLm1lYW4sIFByb2JhYmlsaXR5Lm1lYW4pLCBjb2xvcj0iZ3JleSIpICsKICBnZW9tX3BvaW50KGRhdGE9VGFzaWNfdG90YWxfZGlmZl9ub2Rlcy5taWNyb2V4b25zLCBhZXMoRGVsdGFQc2kubWVhbiwgUHJvYmFiaWxpdHkubWVhbiksIGNvbG9yPSJibGFjayIpICsKICBnZW9tX3RleHRfcmVwZWwoZGF0YSA9IFRhc2ljX3RvdGFsX2RpZmZfbm9kZXMubWljcm9leG9uc1shaXMubmEoTG9jYXRpb24pLF0sCiAgICAgICAgICAgICAgICAgIGFlcyh4PURlbHRhUHNpLm1lYW4sIHk9UHJvYmFiaWxpdHkubWVhbiwgY29sb3VyPUxvY2F0aW9uKSwgCiAgICAgICAgICAgICAgICAgIG51ZGdlX3kgICAgICA9IDMsCiAgICAgICAgICAgICAgICAgIGRpcmVjdGlvbiAgICA9ICJ4IiwKICAgICAgICAgICAgICAgICAgYW5nbGUgICAgICAgID0gOTAsCiAgICAgICAgICAgICAgICAgIHZqdXN0ICAgICAgICA9IDEsCiAgICAgICAgICAgICAgICAgIHNlZ21lbnQuc2l6ZSA9IDAuMiwKICAgICAgICAgICAgICAgICAgZm9udGZhY2UgPSAiYm9sZCIsCiAgICAgICAgICAgICAgICAgIGZvcmNlID0gMSwKICAgICAgICAgICAgICAgICAgbGFiZWw9VGFzaWNfdG90YWxfZGlmZl9ub2Rlcy5taWNyb2V4b25zWyFpcy5uYShMb2NhdGlvbikgLCBtZ2lfc3ltYm9sXSApICsKICAKCiAgCiAgeWxpbSgwLjUsIDEuMTUpICsKICB0aGVtZV9idygpICsKICB4bGFiKCIiKSArCiAgeGxhYigiTWVhbiBkZWx0YSBQU0kiKSArCiAgeWxhYigiTWVhbiBwcm9iYWJpbGl0eSIpICsKICBzY2FsZV9jb2xvcl9tYW51YWwodmFsdWVzPWMoImRhcmtnb2xkZW5yb2QiICwgImZpcmVicmljazQiLCAiZm9yZXN0Z3JlZW4iKSkgKwoKCiAgdGhlbWUoIGxlZ2VuZC5wb3NpdGlvbiA9ICJub25lIikgCgpgYGAKCgoKYGBge3J9CgpQQ0FfbG9hZGluZ3NbLCBNRV9jbHVzdGVyOj1hcy5udW1lcmljKChhcy5jaGFyYWN0ZXIoTUVfY2x1c3RlcikpKV0KTUVfY2x1c3Rlcl9uYW1lc1ssIE1FX2NsdXN0ZXI6PWFzLm51bWVyaWMoKGFzLmNoYXJhY3RlcihNRV9jbHVzdGVyKSkpXQpNRV9jbHVzdGVycy5pbmZvIDwtIG1lcmdlKFBDQV9sb2FkaW5ncywgTUVfY2x1c3Rlcl9uYW1lcywgYnk9Ik1FX2NsdXN0ZXIiKQoKbGlicmFyeShyZWFkcikKCmxpYnJhcnkoSG1pc2MpCgoKVGFzaWNfc2FtcGxlX2luZm8gPC0gZGF0YS50YWJsZShyZWFkX2RlbGltKCJ+L0dvb2dsZV9Ecml2ZS9SZXN1bHRzL01FL1NpbmdsZV9jZWxsL1Rhc2ljL0dTRTcxNTg1LnR4dCIsIAogICAgIlx0IiwgZXNjYXBlX2RvdWJsZSA9IEZBTFNFLCB0cmltX3dzID0gVFJVRSkpCgoKVGFzaWNfY2x1c3RlcmluZyA8LSBkYXRhLnRhYmxlKHJlYWRfZGVsaW0oIn4vR29vZ2xlX0RyaXZlL1Jlc3VsdHMvTUUvU2luZ2xlX2NlbGwvVGFzaWMvR1NFNzE1ODVfQ2x1c3RlcmluZ19SZXN1bHRzLmNzdiIsIAogICAgIiwiLCBlc2NhcGVfZG91YmxlID0gRkFMU0UsIHRyaW1fd3MgPSBUUlVFKSkKCnRhc2ljX2V0X2FsX1NUMyA8LSBkYXRhLnRhYmxlKHJlYWRfY3N2KCJ+L0dvb2dsZV9Ecml2ZS9SZXN1bHRzL01FL1NpbmdsZV9jZWxsL1Rhc2ljL3Rhc2ljX2V0X2FsLlNUMy5jc3YiKSkKCgoKdGFzaWNfbWV0YWRhdGEgPC0gbWVyZ2UoVGFzaWNfc2FtcGxlX2luZm8sIHRhc2ljX2V0X2FsX1NUMywgYnkueD0iU2FtcGxlX05hbWVfcyIsIGJ5Lnk9IkdFTyBTYW1wbGUgQWNjZXNzaW9uIikKCiN0YXNpY19tZXRhZGF0YSA8LSB0YXNpY19tZXRhZGF0YVssIGMoIlJ1bl9zIiwgIkdFTyBTYW1wbGUgVGl0bGUiKSBdCgoKdGFzaWNfbWV0YWRhdGEgPC0gbWVyZ2UoIFRhc2ljX2NsdXN0ZXJpbmcsIHRhc2ljX21ldGFkYXRhLCBieS54PSJzYW1wbGVfdGl0bGUiLCBieS55PSJHRU8gU2FtcGxlIFRpdGxlIikKCgoKCgpNRV9jb3ZfZmlsdGVyZWRfdGFzaWMgPC0gbWVyZ2UoIE1FX2NvdiwgCiAgICAgICB0YXNpY19tZXRhZGF0YVssIGMoInNhbXBsZV90aXRsZSIsICJicm9hZF90eXBlIiwgImNvcmVfaW50ZXJtZWRpYXRlIiwgInByaW1hcnlfdHlwZSIsICJSdW5fcyIpXSwKICAgICAgIGJ5Lng9IkZJTEVfTkFNRSIsIGJ5LnkgPSAiUnVuX3MiKQoKCk1FX2Nvdl9maWx0ZXJlZF90YXNpYyA8LSAgTUVfY292X2ZpbHRlcmVkX3Rhc2ljW01FICVpbiUgTUVfZmluYWxbLCBNRV0gICwgXQoKCk1FX2Nvdl9maWx0ZXJlZF90YXNpYyRzdW1fTUVfY292ZXJhZ2UgPC0gIHNhcHBseShzdHJzcGxpdChhcy5jaGFyYWN0ZXIoTUVfY292X2ZpbHRlcmVkX3Rhc2ljJE1FX2NvdmVyYWdlcyksICIsIiwgZml4ZWQ9VCksIGZ1bmN0aW9uKHgpIHN1bShhcy5udW1lcmljKHgpKSkKTUVfY292X2ZpbHRlcmVkX3Rhc2ljJHN1bV9TSl9jb3ZlcmFnZSA8LSAgc2FwcGx5KHN0cnNwbGl0KGFzLmNoYXJhY3RlcihNRV9jb3ZfZmlsdGVyZWRfdGFzaWMkU0pfY292ZXJhZ2VzKSwgIiwiLCBmaXhlZD1UKSwgZnVuY3Rpb24oeCkgc3VtKGFzLm51bWVyaWMoeCkpKQoKCiNNRV9jb3ZfZmlsdGVyZWRfdGFzaWNbLCBgOj1gKCB0b3RhbF9jb3ZfYWx0ZXJuYXRpdmVzXzU9QWx0NV9jb3ZlcmFnZXMsIHRvdGFsX2Nvdl9hbHRlcm5hdGl2ZXNfMz1BbHQzX2NvdmVyYWdlcyldCk1FX2Nvdl9maWx0ZXJlZF90YXNpY1sgQWx0NV9jb3ZlcmFnZXM9PSJOb25lIiAsIGA6PWAoIEFsdDVfY292ZXJhZ2VzPTApXQpNRV9jb3ZfZmlsdGVyZWRfdGFzaWNbIEFsdDNfY292ZXJhZ2VzPT0iTm9uZSIgLCBgOj1gKCBBbHQzX2NvdmVyYWdlcz0wKV0KTUVfY292X2ZpbHRlcmVkX3Rhc2ljJHRvdGFsX2Nvdl9hbHRlcm5hdGl2ZXNfNSA8LSAgc2FwcGx5KHN0cnNwbGl0KGFzLmNoYXJhY3RlcihNRV9jb3ZfZmlsdGVyZWRfdGFzaWMkQWx0NV9jb3ZlcmFnZXMpLCAiLCIsIGZpeGVkPVQpLCBmdW5jdGlvbih4KSBzdW0oYXMubnVtZXJpYyh4KSkpCk1FX2Nvdl9maWx0ZXJlZF90YXNpYyR0b3RhbF9jb3ZfYWx0ZXJuYXRpdmVzXzMgPC0gIHNhcHBseShzdHJzcGxpdChhcy5jaGFyYWN0ZXIoTUVfY292X2ZpbHRlcmVkX3Rhc2ljJEFsdDNfY292ZXJhZ2VzKSwgIiwiLCBmaXhlZD1UKSwgZnVuY3Rpb24oeCkgc3VtKGFzLm51bWVyaWMoeCkpKQoKTUVfY292X2ZpbHRlcmVkX3Rhc2ljX3ByaW1hcnlfdHlwZSA8LSBNRV9jb3ZfZmlsdGVyZWRfdGFzaWNbLCAuKHN1bV9NRV9jb3ZlcmFnZT1zdW0oc3VtX01FX2NvdmVyYWdlKSwKICAgICAgICAgICAgICAgICAgICAgICAgICBzdW1fU0pfY292ZXJhZ2UgPXN1bShzdW1fU0pfY292ZXJhZ2UpLAogICAgICAgICAgICAgICAgICAgICAgICAgIHRvdGFsX2Nvdl9hbHRlcm5hdGl2ZXNfMyA9IHN1bSh0b3RhbF9jb3ZfYWx0ZXJuYXRpdmVzXzMpLAogICAgICAgICAgICAgICAgICAgICAgICAgIHRvdGFsX2Nvdl9hbHRlcm5hdGl2ZXNfNSA9IHN1bSh0b3RhbF9jb3ZfYWx0ZXJuYXRpdmVzXzUpCiAgICAgICAgICAgICAgICAgICAgICAgICAgKSwgYnk9YygicHJpbWFyeV90eXBlIiwgImJyb2FkX3R5cGUiLCAiTUUiKSBdCgoKCgoKCk1FX2Nvdl9maWx0ZXJlZF90YXNpY19wcmltYXJ5X3R5cGVbLFBTSTo9KHN1bV9NRV9jb3ZlcmFnZS8oc3VtX01FX2NvdmVyYWdlK3N1bV9TSl9jb3ZlcmFnZSt0b3RhbF9jb3ZfYWx0ZXJuYXRpdmVzXzMrdG90YWxfY292X2FsdGVybmF0aXZlc181KSldCgptaW5fUFNJX3N1bSA8LSA1CiAgCk1FX2Nvdl9maWx0ZXJlZF90YXNpY19wcmltYXJ5X3R5cGVbc3VtX01FX2NvdmVyYWdlK3N1bV9TSl9jb3ZlcmFnZSt0b3RhbF9jb3ZfYWx0ZXJuYXRpdmVzXzMrdG90YWxfY292X2FsdGVybmF0aXZlc181PG1pbl9QU0lfc3VtLCBQU0k6PU5hTl0KCmxpYnJhcnkoSG1pc2MgKQoKTUVfY292X2ZpbHRlcmVkX3Rhc2ljX3ByaW1hcnlfdHlwZVsgLCAKICAgICAgICAgICAgYDo9YCgKICAgICAgICAgICAgICBsb3dlcj1iaW5jb25mKHg9c3VtX01FX2NvdmVyYWdlLCBuPShzdW1fTUVfY292ZXJhZ2Urc3VtX1NKX2NvdmVyYWdlK3RvdGFsX2Nvdl9hbHRlcm5hdGl2ZXNfMyt0b3RhbF9jb3ZfYWx0ZXJuYXRpdmVzXzUpIClbLCAiTG93ZXIiXSwKICAgICAgICAgICAgICB1cHBlcj1iaW5jb25mKHg9c3VtX01FX2NvdmVyYWdlLCBuPShzdW1fTUVfY292ZXJhZ2Urc3VtX1NKX2NvdmVyYWdlK3RvdGFsX2Nvdl9hbHRlcm5hdGl2ZXNfMyt0b3RhbF9jb3ZfYWx0ZXJuYXRpdmVzXzUpIClbLCAiVXBwZXIiXQogICAgICAgICAgICAgICkgXQoKCgpNRV9jb3ZfZmlsdGVyZWRfdGFzaWNfcHJpbWFyeV90eXBlIDwtIG1lcmdlKE1FX2Nvdl9maWx0ZXJlZF90YXNpY19wcmltYXJ5X3R5cGUsIHVuaXF1ZShnZW5lX3RhYmxlLlRPVEFMKSwgYnk9Ik1FIikKCgpNRV9jb3ZfZmlsdGVyZWRfdGFzaWNfcHJpbWFyeV90eXBlLk1FX2NsdXN0ZXJzIDwtIG1lcmdlKE1FX2Nvdl9maWx0ZXJlZF90YXNpY19wcmltYXJ5X3R5cGUsIE1FX2NsdXN0ZXJzLmluZm9bLCBjKCJNRSIsICJNRV9jbHVzdGVyLnR5cGUiLCAiTUVfY2x1c3Rlci5uYW1lIildLCBieT0iTUUiKQpgYGAKCmBgYHtyfQpwcmltYXJ5X3R5cGVfbGV2ZWxzIDwtIGModW5pcXVlKE1FX2Nvdl9maWx0ZXJlZF90YXNpY19wcmltYXJ5X3R5cGVbYnJvYWRfdHlwZT09IkVuZG90aGVsaWFsIENlbGwiLCBwcmltYXJ5X3R5cGVdKSwKdW5pcXVlKE1FX2Nvdl9maWx0ZXJlZF90YXNpY19wcmltYXJ5X3R5cGVbYnJvYWRfdHlwZT09Ik9saWdvZGVuZHJvY3l0ZSIsIHByaW1hcnlfdHlwZV0pLAp1bmlxdWUoTUVfY292X2ZpbHRlcmVkX3Rhc2ljX3ByaW1hcnlfdHlwZVticm9hZF90eXBlPT0iTWljcm9nbGlhIiwgcHJpbWFyeV90eXBlXSksCnVuaXF1ZShNRV9jb3ZfZmlsdGVyZWRfdGFzaWNfcHJpbWFyeV90eXBlW2Jyb2FkX3R5cGU9PSJPbGlnb2RlbmRyb2N5dGUgUHJlY3Vyc29yIENlbGwiLCBwcmltYXJ5X3R5cGVdKSwKc29ydCh1bmlxdWUoTUVfY292X2ZpbHRlcmVkX3Rhc2ljX3ByaW1hcnlfdHlwZVticm9hZF90eXBlPT0iQXN0cm9jeXRlIiwgcHJpbWFyeV90eXBlXSkpLApzb3J0KHVuaXF1ZShNRV9jb3ZfZmlsdGVyZWRfdGFzaWNfcHJpbWFyeV90eXBlW2Jyb2FkX3R5cGU9PSJHbHV0YW1hdGVyZ2ljIE5ldXJvbiIsIHByaW1hcnlfdHlwZV0pKSwKdW5pcXVlKE1FX2Nvdl9maWx0ZXJlZF90YXNpY19wcmltYXJ5X3R5cGVbYnJvYWRfdHlwZT09IkdBQkEtZXJnaWMgTmV1cm9uIiwgcHJpbWFyeV90eXBlXSkgKQoKTUVfY292X2ZpbHRlcmVkX3Rhc2ljX3ByaW1hcnlfdHlwZSRwcmltYXJ5X3R5cGUgPC0gZmFjdG9yKE1FX2Nvdl9maWx0ZXJlZF90YXNpY19wcmltYXJ5X3R5cGUkcHJpbWFyeV90eXBlICwgbGV2ZWxzID0gcHJpbWFyeV90eXBlX2xldmVscykKYGBgCgoKYGBge3IsIGZpZy53aWR0aD0xMiwgIGZpZy5oZWlnaHQ9OX0KCnRhcmdldF9nZW5lcyA8LSBjKCJHYWJyZzIiLCAiTnJ4bjEiLCAiTnJ4bjMiLCAiUHRwcmQiKQoKTUVfY292X2ZpbHRlcmVkX3Rhc2ljX3ByaW1hcnlfdHlwZVsobWdpX3N5bWJvbCAlaW4lIHRhcmdldF9nZW5lcykgJiAoTUUgJWluJSBTaWdfR0FCQWVyZ2ljX05ldXJvbl92c19HbHV0YW1hdGVyZ2ljX05ldXJvbiRleG9uX0lEKV0KTUVfY292X2ZpbHRlcmVkX3Rhc2ljX3ByaW1hcnlfdHlwZVtpcy5uYShQU0kpLCBgOj1gKGxvd2VyPU5BLCB1cHBlcj1OQSkgXQoKCkZpZzguYy50b3AgPC0gZ2dwbG90KE1FX2Nvdl9maWx0ZXJlZF90YXNpY19wcmltYXJ5X3R5cGVbIG1naV9zeW1ib2wgJWluJSB0YXJnZXRfZ2VuZXMgJiBNRSAlaW4lIFNpZ19HQUJBZXJnaWNfTmV1cm9uX3ZzX0dsdXRhbWF0ZXJnaWNfTmV1cm9uJGV4b25fSUQsIF0gICkgKwogIGdlb21fcG9pbnRyYW5nZShhZXMoeD1wcmltYXJ5X3R5cGUsIHk9UFNJLCBjb2xvdXI9IGJyb2FkX3R5cGUsIGdyb3VwPWJyb2FkX3R5cGUsIHltaW49bG93ZXIsIHltYXg9dXBwZXIpKSsKICBmYWNldF9ncmlkKCBwYXN0ZTAoIG1naV9zeW1ib2wsIE1FKSB+IC4gKSArCiAgdGhlbWVfYncoKSArCiAgdGhlbWUoYXhpcy50ZXh0LnggPSBlbGVtZW50X3RleHQoYW5nbGUgPSA5MCwgaGp1c3QgPSAwLCB2anVzdCA9IDAuNSkpICsKICB0aGVtZShheGlzLnRleHQueCA9IGVsZW1lbnRfdGV4dChhbmdsZSA9IDkwKSwgbGVnZW5kLnBvc2l0aW9uID0gInRvcCIsIGxlZ2VuZC5kaXJlY3Rpb24gPSAiaG9yaXpvbnRhbCIpICsKICB0aGVtZShheGlzLnRpdGxlLng9ZWxlbWVudF9ibGFuaygpLAogICAgICAgIGF4aXMudGV4dC54PWVsZW1lbnRfYmxhbmsoKSwKICAgICAgICBheGlzLnRpY2tzLng9ZWxlbWVudF9ibGFuaygpKQoKCgoKRmlnOC5jLnRvcApgYGAKCgpgYGB7cn0KTUVfY292X2ZpbHRlcmVkX3Rhc2ljX3ByaW1hcnlfdHlwZQpgYGAKCgoKYGBge3IsIGZpZy53aWR0aD0xMiwgIGZpZy5oZWlnaHQ9Mn0KClRhc2ljX2NsdXN0ZXJpbmckcHJpbWFyeV90eXBlIDwtIGZhY3RvcihUYXNpY19jbHVzdGVyaW5nJHByaW1hcnlfdHlwZSAsIGxldmVscyA9IHByaW1hcnlfdHlwZV9sZXZlbHMpCgpGaWc4LmMuYm90dG9tIDwtIGdncGxvdChUYXNpY19jbHVzdGVyaW5nWyBicm9hZF90eXBlIT0iVW5jbGFzc2lmaWVkIiwgLk4gLCBieT1jKCJwcmltYXJ5X3R5cGUiLCAiYnJvYWRfdHlwZSIpIF0pICsKICBnZW9tX2JhciggYWVzKHg9cHJpbWFyeV90eXBlLCB5PU4sICBmaWxsPWJyb2FkX3R5cGUpICwgc3RhdD0iaWRlbnRpdHkiKSArCiAgICB0aGVtZV9idygpICsKICB0aGVtZShheGlzLnRleHQueCA9IGVsZW1lbnRfdGV4dChhbmdsZSA9IDkwLCBoanVzdCA9IDAsIHZqdXN0ID0gMC41KSkgKwogIHRoZW1lKGF4aXMudGV4dC54ID0gZWxlbWVudF90ZXh0KGFuZ2xlID0gOTApLCBsZWdlbmQucG9zaXRpb24gPSAibm9uZSIsIGxlZ2VuZC5kaXJlY3Rpb24gPSAiaG9yaXpvbnRhbCIsIHBhbmVsLmdyaWQubWFqb3IgPSBlbGVtZW50X2JsYW5rKCksIHBhbmVsLmdyaWQubWlub3IgPSBlbGVtZW50X2JsYW5rKCkpCgpGaWc4LmMuYm90dG9tCmBgYAoKYGBge3IsIGZpZy53aWR0aD0xMiwgIGZpZy5oZWlnaHQ9OX0KCnBsb3RfZ3JpZChGaWc4LmMudG9wLCBGaWc4LmMuYm90dG9tLCBuY29sPTEsIHJlbF9oZWlnaHRzID0gYyg3LDIpICkKCgpgYGAKCgoKCiMjIyMjCgpTaW5nbGUgY2VsbCBzdGFzdAoKYGBge3J9Cm5yb3coVGFzaWNfdG90YWxfZGlmZl9ub2RlcykKYGBgCgoKYGBge3J9CnRhYmxlKFRhc2ljX3RvdGFsX2RpZmZfbm9kZXMkaXMubWljcm9leG9uKQpgYGAKCgpgYGB7cn0KVGFzaWNfdG90YWxfZGlmZl9ub2Rlc1sgICwgaXMubWljcm9leG9uOj0haXMubmEobWljcm9leG9uX0lEKSBdCgpUYXNpY190b3RhbF9kaWZmX25vZGVzWyBpcy5uYShpcy5kaWZmKSAsIGlzLmRpZmY6PSJGQUxTRSIgIF0KClRhc2ljX3RvdGFsX2RpZmZfbm9kZXMuc3RhdHMgPC0gVGFzaWNfdG90YWxfZGlmZl9ub2Rlc1sgLCAuTiAsIGJ5PWMoIlR5cGUiLCAiaXMuZGlmZiIsICJpcy5taWNyb2V4b24iKV0KClRhc2ljX3RvdGFsX2RpZmZfbm9kZXMuc3RhdHNbICwgVG90YWw6PXN1bShOKSwgYnk9YygiVHlwZSIsICJpcy5taWNyb2V4b24iKV0KClRhc2ljX3RvdGFsX2RpZmZfbm9kZXMuc3RhdHNbaXMuZGlmZj09IlRSVUUiLCBdCgoKVGFzaWNfdG90YWxfZGlmZl9ub2Rlcy5zdGF0c1ssIHJhdGlvOj1OL1RvdGFsXQoKClRhc2ljX3RvdGFsX2RpZmZfbm9kZXMuc3RhdHMgPC0gVGFzaWNfdG90YWxfZGlmZl9ub2Rlcy5zdGF0c1tpcy5kaWZmPT0iVFJVRSIsIF0KClRhc2ljX3RvdGFsX2RpZmZfbm9kZXMuc3RhdHNbaXMuZGlmZj09IlRSVUUiLCBdCgpgYGAKCgpgYGB7cn0KCkNFLmNvbnQgPC0gbWF0cml4KG5yb3c9MiwgYyhUYXNpY190b3RhbF9kaWZmX25vZGVzLnN0YXRzW1R5cGU9PSJDRSIgJiBpcy5taWNyb2V4b249PSJUUlVFIiwgTiBdLApUYXNpY190b3RhbF9kaWZmX25vZGVzLnN0YXRzW1R5cGU9PSJDRSIgJiBpcy5taWNyb2V4b249PSJUUlVFIiwgVG90YWwtTiBdLApUYXNpY190b3RhbF9kaWZmX25vZGVzLnN0YXRzW1R5cGU9PSJDRSIgJiBpcy5taWNyb2V4b249PSJGQUxTRSIsIE4gXSwKVGFzaWNfdG90YWxfZGlmZl9ub2Rlcy5zdGF0c1tUeXBlPT0iQ0UiICYgaXMubWljcm9leG9uPT0iRkFMU0UiLCBUb3RhbC1OIF0pKQoKY2hpc3EudGVzdChDRS5jb250KQpgYGAKCgpgYGB7cn0KQ0UuY29udC50b3RhbCA8LSBtYXRyaXgobnJvdz0yLCBjKHN1bShUYXNpY190b3RhbF9kaWZmX25vZGVzLnN0YXRzWyBpcy5taWNyb2V4b249PSJUUlVFIiwgTiBdKSwKc3VtKFRhc2ljX3RvdGFsX2RpZmZfbm9kZXMuc3RhdHNbIGlzLm1pY3JvZXhvbj09IlRSVUUiLCBUb3RhbC1OIF0pLApzdW0oVGFzaWNfdG90YWxfZGlmZl9ub2Rlcy5zdGF0c1tpcy5taWNyb2V4b249PSJGQUxTRSIsIE4gXSksCnN1bShUYXNpY190b3RhbF9kaWZmX25vZGVzLnN0YXRzWyBpcy5taWNyb2V4b249PSJGQUxTRSIsIFRvdGFsLU4gXSkpKQoKY2hpc3EudGVzdChDRS5jb250LnRvdGFsKQpgYGAKCgoKCmBgYHtyfQpuPW5yb3coVGFzaWNfdG90YWxfZGlmZl9ub2RlcykKYT1ucm93KFRhc2ljX3RvdGFsX2RpZmZfbm9kZXNbIGlzLmRpZmY9PSJUUlVFIiwgXSkKYj1ucm93KFRhc2ljX3RvdGFsX2RpZmZfbm9kZXNbIGlzLm1pY3JvZXhvbj09IlRSVUUiLCBdKQp0PW5yb3coVGFzaWNfdG90YWxfZGlmZl9ub2Rlc1sgaXMubWljcm9leG9uPT0iVFJVRSIgJiAgaXMuZGlmZj09IlRSVUUiLCBdKQoKZGh5cGVyKHQsIGEsIG4gLSBhLCBiKQpzdW0oZGh5cGVyKHQ6YiwgYSwgbiAtIGEsIGIpKQpgYGAKCgoKCgpgYGB7cn0Kbj1ucm93KFRhc2ljX3RvdGFsX2RpZmZfbm9kZXNbVHlwZT09IkNFIiwgXSkKYT1ucm93KFRhc2ljX3RvdGFsX2RpZmZfbm9kZXNbVHlwZT09IkNFIiAmIGlzLmRpZmY9PSJUUlVFIiwgXSkKYj1ucm93KFRhc2ljX3RvdGFsX2RpZmZfbm9kZXNbVHlwZT09IkNFIiAmIGlzLm1pY3JvZXhvbj09IlRSVUUiLCBdKQp0PW5yb3coVGFzaWNfdG90YWxfZGlmZl9ub2Rlc1tUeXBlPT0iQ0UiICYgaXMubWljcm9leG9uPT0iVFJVRSIgJiAgaXMuZGlmZj09IlRSVUUiLCBdKQoKZGh5cGVyKHQsIGEsIG4gLSBhLCBiKQpzdW0oZGh5cGVyKHQ6YiwgYSwgbiAtIGEsIGIpKQpgYGAKCgoKYGBge3J9Cgpucm93KFRhc2ljX3RvdGFsX2RpZmZfbm9kZXNbIGlzLm1pY3JvZXhvbj09IlRSVUUiICYgIGlzLmRpZmY9PSJU
[truncated: 41,468 more chars]
