# Supplementary material for: MicroExonator enables systematic discovery and quantification of microexons across mouse embryonic development
Source: Genome Biol. 2021 Jan 22;22:43. doi: 10.1186/s13059-020-02246-2 (PMC7821500; doi:10.1186/s13059-020-02246-2)
Supplement: Supplementary file 9 — Additional file 9. Review history. [file 13059_2020_2246_MOESM9_ESM.docx]

Review History

**First round of review**

**Reviewer 1**

**Are you able to assess all statistics in the manuscript, including the appropriateness of statistical tests used?**

Yes, and I have assessed the statistics in my report.

**Comments to author:**

In this manuscript by Parada et al., a computational workflow is introduced for the discovery of microexons (<30nt) that have traditionally been difficult to detect with standard RNA-seq mapping algorithms. Notably, the workflow can discover new microexons from novel splice junctions in the RNA-seq data being processed, and this can thus extend alternative workflows that rely on a pre-compiled database of splice junctions. The authors benchmark the workflow against related methods through simulated incorporation of microexons into annotated transcripts. Having proved its specificity and sensitivity, the workflow is used to detect and quantify microexon usage across mouse embryonic development. This revealed nearly 3000 microexons detected across 271 RNA-seq datasets, and many of these could be stratified into microexons that where neuronal, muscle or adrenal in origin. Programs of microexon usage were found to be tightly coordinated across development and enriched in specific gene ontologies. Impressively, after demonstrating discovery of new microexons in less well-annotated species, the authors show how microexon discovery can be extended to single-cell SMART-seq2 datasets. This revealed cell sub-type specific splicing patterns. Whilst this workflow not be possible with the increasingly used 3' DGE single cell profiling strategies, it nevertheless provides opportunity to gain new understanding of microexons using the many existing SMART-seq2 datasets.

The work is of high quality and would be suited to publication in this journal subject to minor revisions. Indeed, I believe it would be a well-used approach and could have similar impact to the increasingly used IRFinder workflow for detection of intron retention events.

In the absence of page numbers, page 1 is considered the first page of the manuscript containing the title, authors and start of abstract.

1. Figure 1A: It's unclear why there is need to show two isoforms in both the first and last part of panel A. This could add confusion to the reader.

2. Figure 1D: The green and red lines should be clearly explained in the legend.

3. Page 6, line 16/17: VastTools is perhaps conspicuous by its absence without further clarity to the comparisons being made in this section. It is suggested that the start of the section adds that the comparison is being made to other methods that are capable of detecting new microexons, thus excluding VastTools.

4. Page 6, line 33-35: It would be helpful to detail how removing 3/4nt microexons improves the FDR of the other mappers being compared to.

5. Page 8, line 15/16: As written, it appears that VastDB (and by association VastTools) was used for the analysis of mouse embryonic data. Indeed, microExonater is not mentioned in this section. It would help if the use of microExonater is reinforced here to avoid any potential confusion.

6. Page 8, line 57/58. The N, M, NM, NN and WN terms are used in figure 3D in addition to 4A-B. This reference should be added accordingly.

7. There appears to be no reference to Figure 3F or 3G in line with the text.

8. Figure 3A-C: It would be useful to have the percentage contributions of the PCs added to the axis.

9. Page 12, line 10/11: More detail is required on how forebrain samples were pooled given that three categories are shown in 4G. The text implies all time points were pooled together.

10. Figure 4 has no panel J marked despite the reference in text and figure legend. I presume that the letter J is missing from the euler diagram.

11. Page 14, line 40/41: Should this not reference 5G rather than 5A-C. Similar mis-labelling of panels occurs in other places in this section and it is suggested that this (and other figure referencing) is thoroughly checked during a revision.

12. Figure 5: More detail is required in the figure legend to describe parts A-E. At present it is too vague and not fully explained in the main text. Adding titles to these individual panels should also be considered to aid interpretation.

13. Page 15, lines 18-22: Rather than simply being described, can these results be interpreted in the context of what centrality represents?

14. Page 17, line 30-31: I'm not clear how figure 6A demonstrates that the 31 microexons are true positives. Whilst the statement might be true, an appropriate summary of figure 6A should be added to the main text to cover panel A appropriately.

15. Page 17, line 41/42: There appears more figure mis-labelling which should have been checked before submission. Panel 6C relates to ES cells and not a a developmental time-point. It appears 6C/D are in wrong orientation to one another in the text (see line 49).

16. Page 18, line 28: As above - figure reference should be updated to Fig 6E

17. Page 19, line 30/34: I'm unclear how 20 microexons were identified from the single cell PSI values (line 34), when previously only 19 were reported to be identified in this analysis (line 30).

18. Figure 7C: It would be useful to know how many cells are in each cell sub-type.

19. Page 21, line 37/38: The 941 previously uncharacterised microexons appears out of the blue as it was not used in the results section. It be useful to cover how this figure was derived in the appropriate section of the results.

20. Page 23, line 16/17: The text states 39 microexons were differentially included between the cell types, but only 38 were covered in the results. This should be corrected accordingly.

**Reviewer 2**

**Are you able to assess all statistics in the manuscript, including the appropriateness of statistical tests used?**

Yes, and I have assessed the statistics in my report.

**Comments to author:**

Parada and colleagues present MicroExonator, a workflow to identify and quantify microexons in RNA-Seq data. Microexons have recently emerged as the most highly conserved component of regulated alternative splicing in vertebrates, and an increasing number have been reported with important roles in nervous system development and function. However, efficient tools to directly identify them from RNA-Seq data have not been previously described.

MicroExonator incorporates existing annotations and augments them with de novo detection, and subsequently incorporates any newly found microexons in a second quantification step. Based on simulated data, the authors demonstrate that MicroExonator possesses greater specificity than current de-novo spliced aligners on their own, and currently is the only effective tool to identify exons shorter than ~ 6 nt. Analyzing a compendium of 289 mouse RNA-seq datasets, the authors classify microexons by regulatory patterns and conclude that developmental stage, rather than tissue type, is the axis along which most microexon regulation occurs. They further confirm previous findings about microexon characteristics, identify some new neural microexons as well as some that are expressed in the adrenal gland. The authors also present an interesting analysis of single-cell RNA-seq data from the murine cortex and identify microexons that display neural sub-type-specific co-regulation. They also apply their tool to identify microexons in zebrafish RNA-Seq data.

MicroExonator will be a welcome method for the important emerging field of microexons. The presentation of the tool and results from mouse and zebrafish are generally solid. This reviewer was not able to access Supplemental Table 1. This reviewer recommends publication after the authors address or comment on the following points:

1. Why do the authors require inserts, corresponding to putative microexons, to map to intronic sequences with canonical splice site dinucleotides? Is there a high-false positive rate when non-canonical sites are permitted?

2. Fig. S1: How much of the difference in assigned reads is due to complementing the junction library with novel putative microexons vs. use of a different aligner (BWA-MEM vs. bowtie)?

3. During library construction, how were cases dealt with where the upstream or downstream transcript region encompassed alternative exons or 3'/5' alternative splice sites?

4. What purpose does hisat2 re-mapping of the reads with insertions to the reference genome during the discovery step serve?

5. In the quantification module, reads are aligned to modified junction libraries allowing two mismatches. If both mismatches overlap with the microexon portion of the junction, that could conceivably lead to false positive assignment. Have the authors tested this in their simulations?

6. Quantification of microexon inclusion from RNA-seq data was done both with MicroExonator's own scripts and using Whippet. How did they compare, and which do the authors recommend? Moreover, which was used for benchmarking (Fig. 2D)?

7. Fig. 3: Labeling the sample clusters in panel D in the same way as in E would facilitate comparison.

8. The claim that most microexons, once included during development, remain included (Fig. 4E) should be supported by concrete numbers.

9. It is not obvious from the referenced Fig. H-I that microexons in genes with the highest eigencentrality are first to be included. In fact, Fig. 5F seems to suggest there is no correlation.

10. A list of detected microexons in mouse and zebrafish should be included.

11. The author validate a single one of the 31 newly identified neuronal microexons. They should use the RNA they generated to validate a larger panel.

12. Installation of MicroExonator via conda failed. The authors should do some robustness testing of the software framework.

Recommended text changes

13. Abstract: "…they play a key role during neuronal differentiation". This could be updated to reflect key roles in nervous system development and function, especially in light of recent results demonstrating functions of microexons in behaviour and cognition.

Introduction:

14. "Consequently, alternative splicing impinges directly onto numerous biological processes such as cell cycle, cell differentiation, development, sex, circadian rhythm, response to environmental change, pathogen exposure and disease (Bell et al., 1988; Irimia and Blencowe, 2012; Kalsotra and Cooper, 2011; Venables et al., 2012)." There are several more recent reviews on these topics, including some from the same authors.

15. "Importantly, microexon inclusion has been proposed to have a key regulatory role during brain development, having an influence over neurite outgrowth, cortical layering and axon guidance (Quesnel-Vallières et al., 2015)". There are additional papers on this topic including the following:

Johnson V, Junge HJ, Chen Z: Temporal regulation of axonal repulsion by alternative splicing of a conserved microexon in mammalian Robo1 and Robo2. Elife 2019, 8.

Saito Y, Miranda-Rottmann S, Ruggiu M, Park CY, Fak JJ, Zhong R, Duncan JS, Fabella BA, Junge HJ, Chen Z, et al.: NOVA2-mediated RNA regulation is required for axonal pathfinding during development. Elife 2016, 5.

Laurent B, Ruitu L, Murn J, Hempel K, Ferrao R, Xiang Y, Liu S, Garcia BA, Wu H, Wu F, et al.: A Specific LSD1/KDM1A Isoform Regulates Neuronal Differentiation through H3K9 Demethylation. Mol Cell 2015, 57:957-970.

Ohnishi T, Shirane M, Hashimoto Y, Saita S, Nakayama KI: Identification and characterization of a neuron-specific isoform of protrudin. Genes to Cells 2014, 19:97-111.

Jacob J, Haspel J, Kane-Goldsmith N, Grumet M: L1 mediated homophilic binding and neurite outgrowth are modulated by alternative splicing of exon 2. J Neurobiol 2002, 51:177-189.

Discussion

16. "…it has been proposed that microexon inclusion relies on an upstream intronic splicing enhancer which is recognized by specific neuronal splicing factors (Gonatopoulos-Pournatzis et al., 2018)". Note that this element is recognized by both neural-specific and widely expressed factors and confirmed experimentally to promote neural microexon inclusion.

17. "In fact, alternative splicing changes associated with ASD are enriched in microexons and they are recapitulated in mutant mice haploinsufficient for SRRM4 (Irimia et al., 2014; Quesnel-Vallières et al., 2015)". I suggest revising to: "In fact, alternative splicing changes associated with ASD are enriched in microexons and they are recapitulated in mutant mice haploinsufficient for SRRM4, which also display multiple autistic-like features (Irimia et al., 2014; replace reference with Quesnel-Vallières et al., 2016: PMID: 27984743). Please also note the activity-dependent results in this study in relation to your bioRxv manuscript on G-quadruplexes.

**Authors Response**

**Point-by-point responses to the reviewers’ comments:**

Reviewer reports:

Reviewer #1: In this manuscript by Parada et al., a computational workflow is introduced for the discovery of microexons (<30nt) that have traditionally been difficult to detect with standard RNA-seq mapping algorithms. Notably, the workflow can discover new microexons from novel splice junctions in the RNA-seq data being processed, and this can thus extend alternative workflows that rely on a pre-compiled database of splice junctions. The authors benchmark the workflow against related methods through simulated incorporation of microexons into annotated transcripts. Having proved its specificity and sensitivity, the workflow is used to detect and quantify microexon usage across mouse embryonic development. This revealed nearly 3000 microexons detected across 271 RNA-seq datasets, and many of these could be stratified into microexons that where neuronal, muscle or adrenal in origin. Programs of microexon usage were found to be tightly coordinated across development and enriched in specific gene ontologies. Impressively, after demonstrating discovery of new microexons in less well-annotated species, the authors show how microexon discovery can be extended to single-cell SMART-seq2 datasets. This revealed cell sub-type specific splicing patterns. Whilst this workflow not be possible with the increasingly used 3' DGE single cell profiling strategies, it nevertheless provides opportunity to gain new understanding of microexons using the many existing SMART-seq2 datasets.

The work is of high quality and would be suited to publication in this journal subject to minor revisions. Indeed, I believe it would be a well-used approach and could have similar impact to the increasingly used IRFinder workflow for detection of intron retention events.

*We thank the reviewer for the positive assessment of our manuscript and for bringing IRFinder to our attention.*

In the absence of page numbers, page 1 is considered the first page of the manuscript containing the title, authors and start of abstract.

1. Figure 1A: It's unclear why there is need to show two isoforms in both the first and last part of panel A. This could add confusion to the reader.

*We thank the reviewer for making this point. We have removed the second isoform, while keeping the splice junctions to make sure that the main point is the presence of unannotated microexons.*

2. Figure 1D: The green and red lines should be clearly explained in the legend.

*We thank the reviewer for pointing out this omission and we have added a description in the caption*

3. Page 6, line 16/17: VastTools is perhaps conspicuous by its absence without further clarity to the comparisons being made in this section. It is suggested that the start of the section adds that the comparison is being made to other methods that are capable of detecting new microexons, thus excluding VastTools.

*We did not include vast-tools for microexon discovery since the software is currently not publicly available. Following the reviewer’s request, we contacted the vast-tools developers and they gave us access to this module, making it possible to include vast-tools as part of our benchmark. The vast-tools microexon discovery module performed better than the other RNA-seq mappers, but MicroExonator was superior in terms of sensitivity, specificity and precision. These results are now included in Figure 2 in the manuscript.*

4. Page 6, line 33-35: It would be helpful to detail how removing 3/4nt microexons improves the FDR of the other mappers being compared to.

*We agree with the reviewer and we have now included a supplementary figure (Fig S3) to show the FDR results after different microexon length threshold for all the different softwares (including vast-tools)*

5. Page 8, line 15/16: As written, it appears that VastDB (and by association VastTools) was used for the analysis of mouse embryonic data. Indeed, microExonater is not mentioned in this section. It would help if the use of microExonater is reinforced here to avoid any potential confusion.

*We thank the reviewer for this suggestion and we have updated the text to clarify the usage of MicroExonator on this section.*

6. Page 8, line 57/58. The N, M, NM, NN and WN terms are used in figure 3D in addition to 4A-B. This reference should be added accordingly.

*We apologize for this omission and we have updated the caption.*

7. There appears to be no reference to Figure 3F or 3G in line with the text.

*We have added this reference in the second paragraph, 10th line of the section called “Microexon inclusion changes dramatically throughout mouse embryonic development“*

8. Figure 3A-C: It would be useful to have the percentage contributions of the PCs added to the axis.

*We have added this information as suggested by the reviewer.*

9. Page 12, line 10/11: More detail is required on how forebrain samples were pooled given that three categories are shown in 4G. The text implies all time points were pooled together.

*We have added a new supplementary table S2 which provides details on how samples were pooled.*

10. Figure 4 has no panel J marked despite the reference in text and figure legend. I presume that the letter J is missing from the euler diagram.

*The reviewer is correct and we have updated the figure.*

11. Page 14, line 40/41: Should this not reference 5G rather than 5A-C. Similar mis-labelling of panels occurs in other places in this section and it is suggested that this (and other figure referencing) is thoroughly checked during a revision.

*We apologize for the mislabeled panels and we have triple-checked them in the revised manuscript.*

12. Figure 5: More detail is required in the figure legend to describe parts A-E. At present it is too vague and not fully explained in the main text. Adding titles to these individual panels should also be considered to aid interpretation.

*We have re-plotted the PPI network figure as a single panel (Fig 5A), indicating each Reactome Pathway in the figure as the reviewer suggested by the reviewer. We have also added missing details to the figure legend, and included further explanation of the results related to Fig 5B in the main text.*

13. Page 15, lines 18-22: Rather than simply being described, can these results be interpreted in the context of what centrality represents?

*Indeed, we agree that an interpretation of these findings were missing. We now include an additional statement to clarify what these results suggest.*

14. Page 17, line 30-31: I'm not clear how figure 6A demonstrates that the 31 microexons are true positives. Whilst the statement might be true, an appropriate summary of figure 6A should be added to the main text to cover panel A appropriately.

*We have updated the text to “The high sensitivity and specificity demonstrated in simulations implies that the false discover rate is <1% for microexons ≥6 nts (Fig 2, S3). Thus, we expect that all 31 microexons ≥6 nts are true positives, and our conclusion is further supported by the fact that the lengths follow a similar periodicity patterns to annotated microexons (Fig 6A).”*

15. Page 17, line 41/42: There appears more figure mis-labelling which should have been checked before submission. Panel 6C relates to ES cells and not a a developmental time-point. It appears 6C/D are in wrong orientation to one another in the text (see line 49).

*We apologise for this mislabeling issue. In this case, our mistake was in the figure. We swapped Fig 6C and 6D to match the text.*

16. Page 18, line 28: As above - figure reference should be updated to Fig 6E

*We have fixed this error.*

17. Page 19, line 30/34: I'm unclear how 20 microexons were identified from the single cell PSI values (line 34), when previously only 19 were reported to be identified in this analysis (line 30).

*We thank the reviewer for pointing this out. We have now updated results related to scRNA-seq analyses and this time we carefully checked the numbers.*

18. Figure 7C: It would be useful to know how many cells are in each cell sub-type.

*We have added a bottom panel indicating the number of cells belonging to each sub-type. We also added relevant annotations related to PTPRD microexons.*

19. Page 21, line 37/38: The 941 previously uncharacterised microexons appears out of the blue as it was not used in the results section. It be useful to cover how this figure was derived in the appropriate section of the results.

*We agree with the reviewer's concern. We have updated this number to the results we got in our latest analysis and we now include this number as part of the first paragraph of the section “Microexon inclusion changes dramatically throughout mouse embryonic development”. Moreover, we have included a Table S1 which contains relevant information for every microexon included in our analysis.*

20. Page 23, line 16/17: The text states 39 microexons were differentially included between the cell types, but only 38 were covered in the results. This should be corrected accordingly.

*We apologize for the inconsistency. Since we have improved our statistical method for detecting microexons that are differentially included between single cell clusters, we have updated these numbers and we have made sure that they are consistent throughout the manuscript.*

Reviewer #2: Parada and colleagues present MicroExonator, a workflow to identify and quantify microexons in RNA-Seq data. Microexons have recently emerged as the most highly conserved component of regulated alternative splicing in vertebrates, and an increasing number have been reported with important roles in nervous system development and function. However, efficient tools to directly identify them from RNA-Seq data have not been previously described.

MicroExonator incorporates existing annotations and augments them with de novo detection, and subsequently incorporates any newly found microexons in a second quantification step. Based on simulated data, the authors demonstrate that MicroExonator possesses greater specificity than current de-novo spliced aligners on their own, and currently is the only effective tool to identify exons shorter than ~ 6 nt. Analyzing a compendium of 289 mouse RNA-seq datasets, the authors classify microexons by regulatory patterns and conclude that developmental stage, rather than tissue type, is the axis along which most microexon regulation occurs. They further confirm previous findings about microexon characteristics, identify some new neural microexons as well as some that are expressed in the adrenal gland. The authors also present an interesting analysis of single-cell RNA-seq data from the murine cortex and identify microexons that display neural sub-type-specific co-regulation. They also apply their tool to identify microexons in zebrafish RNA-Seq data.

MicroExonator will be a welcome method for the important emerging field of microexons. The presentation of the tool and results from mouse and zebrafish are generally solid. This reviewer was not able to access Supplemental Table 1. This reviewer recommends publication after the authors address or comment on the following points:

1. Why do the authors require inserts, corresponding to putative microexons, to map to intronic sequences with canonical splice site dinucleotides? Is there a high-false positive rate when non-canonical sites are permitted?

*The reviewer is correct, high false positive rates would be expected if non-canonical sites are permitted, and requiring canonical dinucleotides reduces the number of matches in an intron. We think that the false positives are particularly troublesome for the very short microexons (as indicated by Fig 1C). MicroExonator currently only allows for discovery of microexons that are flanked by non-canonical dinucleotides, but we hope to enable the detection of microexons flanked by non-canonical dinucleotides in future versions of MicroExonator.*

2. Fig. S1: How much of the difference in assigned reads is due to complementing the junction library with novel putative microexons vs. use of a different aligner (BWA-MEM vs. bowtie)?

*Figure S1 only shows microexons that were detected during both the discovery phase (Round1) and quantification phase (Round2), excluding those that were directly extracted from GENCODE and VastDB. Thus, any microexon that was found during the discovery phase would have been assigned zero reads. The difference due to the use of bowtie instead of BWA-MEM is expected since the former performs ungapped alignments to splice junction tags that already have the microexon sequences. Consequently, the read assignment is more sensitive and as evidenced by the improved microexon inclusion quantification.*

3. During library construction, how were cases dealt with where the upstream or downstream transcript region encompassed alternative exons or 3'/5' alternative splice sites?

*During library construction we extract all annotated splice junctions and we use them to build splice junction tags. When we detect insertions that are located at the splice junctions, they are re-aligned to the intronic sequence. As the reviewer points out, if a putative microexon is detected next to alternative exons or 3’/5’ alternative splice sites, then more than one splice junction may lead to the detection of the same microexon. During the second library construction we place the found microexon sequence into all the splice junctions that span across the microexon coordinate and then for PSI quantification we quantify the reads that support microexon inclusion and all splice junctions that span over the exon, which are evidence of microexon skipping. MicroExonator output reports all the splice junctions that were considered for PSI quantification. If a splice junction is related to two microexons, it will influence the PSI values of both, but the list is filtered to ensure that the list of microexons is non-repetitive.*

4. What purpose does hisat2 re-mapping of the reads with insertions to the reference genome during the discovery step serve?

*The purpose of re-mapping with hisat2 is to discard putative microexons that might be detected due to alignment artefacts. Since hisat2 is a splice aware mapper, we use it to make sure that the reads could not be better mapped to annotated splice junctions to reduce the number of false positives.*

5. In the quantification module, reads are aligned to modified junction libraries allowing two mismatches. If both mismatches overlap with the microexon portion of the junction, that could conceivably lead to false positive assignment. Have the authors tested this in their simulations?

*The reviewer raises an important question regarding the possibility of false positives. To investigate if allowing for two mismatches can cause these issues, we ran additional simulations where we only allowed for zero or one mismatch. As shown in Figure R1, the estimated errors from the PSI quantification are indistinguishable for the three scenarios. Thus, we conclude that allowing for two mismatches does not result in a large number of false positives.*

*Figure R1. Estimation of quantification errors allowing for 0, 1 or 2 mismatches. Using the same simulation strategy as for Figure 2 in the main text, we allowed for fewer mismatches during quantification.*

6. Quantification of microexon inclusion from RNA-seq data was done both with MicroExonator's own scripts and using Whippet. How did they compare, and which do the authors recommend? Moreover, which was used for benchmarking (Fig. 2D)?

*We initially found a high correlation between the MicroExonator and Whippet quantifications. However, upon closer inspection we observed differences for microexons associated with splice sites that result in the inclusion of a long exon (> 30 nt), i.e. when an existing microexon gets extended by a novel microexon so that it is longer than 30 nt. We included the results for this special case in Fig S9E, where we show that Whippet fails to provide independent quantification for these microexon and their corresponding longer exons. Therefore we believe that MicroExonator quantification is more accurate for this small class of microexons. We found 426 microexons that were found differentially included with both quantifications across neuronal samples, and we used this group of all the downstream analyses of bulk RNA-seq results. For benchmarking we used MicroExonator quantification.*

7. Fig. 3: Labeling the sample clusters in panel D in the same way as in E would facilitate comparison.

*We thank the reviewer for suggesting these improvements. We have implemented these changes to Fig 3.*

8. The claim that most microexons, once included during development, remain included (Fig. 4E) should be supported by concrete numbers.

*We did include these numbers and here there is an extraction of the paragraph where we mention this:*

*“Nevertheless, we found 426 microexons that were consistently detected as differentially included (delta PSI > 0.1 and probability > 0.9 using MicroExonator and Whippet PSI values) in at least one of the comparisons between MHN and control groups (Fig S8). Interestingly, 326 of these microexon changes are maintained for all subsequent stages once they have been observed.”*

*Thus from a total of 426 differentially included microexons, 326 are maintained. Relevant information about these alternative splicing events can be found in Table S1.*

9. It is not obvious from the referenced Fig. H-I that microexons in genes with the highest eigencentrality are first to be included. In fact, Fig. 5F seems to suggest there is no correlation.

*We thank the reviewer for pointing out this issue. We observed that groups of genes related with certain GO terms, such as vesicle mediated transport and cytoskeleton organization have significantly higher eigencentrallity than microexons associated with signal transduction, nervous system development and synapse assembly. At the same time, microexons from genes related with cytoskeleton organization are included earlier in development than microexons associated with signal transduction, nervous system development and synapse assembly. As the direct evidence suggesting that microexons that are included early are more central in the PPI network remains weak, we have changed these statements in the text.*

10. A list of detected microexons in mouse and zebrafish should be included.

*We have now included the full list of microexon found in zebrafish in Table S4 and list of conserved microexons between mouse and zebrafish in Table S5.*

11. The author validate a single one of the 31 newly identified neuronal microexons. They should use the RNA they generated to validate a larger panel.

*We agree with the reviewer that a higher number of validation experiments would be desirable. However, the COVID-19 pandemic resulted in major complications and the delays meant that other experiments had to be prioritized. Thus, we have been unable to carry out this work.*

12. Installation of MicroExonator via conda failed. The authors should do some robustness testing of the software framework.

*We apologize for these problems. With the help of several volunteers, we have tested installation across multiple platforms and we believe that the process should be more robust now. Moreover we have extended MicroExonator documentation at https://microexonator.readthedocs.io/.*

13. Abstract: "…they play a key role during neuronal differentiation". This could be updated to reflect key roles in nervous system development and function, especially in light of recent results demonstrating functions of microexons in behaviour and cognition.

*We thank the reviewer for this suggestion, we applied these chances to the abstract.*

Introduction:

14. "Consequently, alternative splicing impinges directly onto numerous biological processes such as cell cycle, cell differentiation, development, sex, circadian rhythm, response to environmental change, pathogen exposure and disease (Bell et al., 1988; Irimia and Blencowe, 2012; Kalsotra and Cooper, 2011; Venables et al., 2012)." There are several more recent reviews on these topics, including some from the same authors.

*We have included more recent references as suggested by the reviewer : (Salz 2011; Kalsotra and Cooper 2011; Baralle and Giudice 2017; Ule and Blencowe 2019). We have also changed the reference format to match the journal style.*

15. "Importantly, microexon inclusion has been proposed to have a key regulatory role during brain development, having an influence over neurite outgrowth, cortical layering and axon guidance (Quesnel-Vallières et al., 2015)". There are additional papers on this topic including the following:

Johnson V, Junge HJ, Chen Z: Temporal regulation of axonal repulsion by alternative splicing of a conserved microexon in mammalian Robo1 and Robo2. Elife 2019, 8.

Saito Y, Miranda-Rottmann S, Ruggiu M, Park CY, Fak JJ, Zhong R, Duncan JS, Fabella BA, Junge HJ, Chen Z, et al.: NOVA2-mediated RNA regulation is required for axonal pathfinding during development. Elife 2016, 5.

Laurent B, Ruitu L, Murn J, Hempel K, Ferrao R, Xiang Y, Liu S, Garcia BA, Wu H, Wu F, et al.: A Specific LSD1/KDM1A Isoform Regulates Neuronal Differentiation through H3K9 Demethylation. Mol Cell 2015, 57:957-970.

Ohnishi T, Shirane M, Hashimoto Y, Saita S, Nakayama KI: Identification and characterization of a neuron-specific isoform of protrudin. Genes to Cells 2014, 19:97-111.

Jacob J, Haspel J, Kane-Goldsmith N, Grumet M: L1 mediated homophilic binding and neurite outgrowth are modulated by alternative splicing of exon 2. J Neurobiol 2002, 51:177-189.

*We thank the reviewer for drawing our attention to these papers. We have now incorporated these references.*

Discussion

16. "…it has been proposed that microexon inclusion relies on an upstream intronic splicing enhancer which is recognized by specific neuronal splicing factors (Gonatopoulos-Pournatzis et al., 2018)". Note that this element is recognized by both neural-specific and widely expressed factors and confirmed experimentally to promote neural microexon inclusion.

*We have changed the text to “but it has been shown that microexon inclusion relies on ….”*

17. "In fact, alternative splicing changes associated with ASD are enriched in microexons and they are recapitulated in mutant mice haploinsufficient for SRRM4 (Irimia et al., 2014; Quesnel-Vallières et al., 2015)". I suggest revising to: "In fact, alternative splicing changes associated with ASD are enriched in microexons and they are recapitulated in mutant mice haploinsufficient for SRRM4, which also display multiple autistic-like features (Irimia et al., 2014; replace reference with Quesnel-Vallières et al., 2016: PMID: 27984743). Please also note the activity-dependent results in this study in relation to your bioRxv manuscript on G-quadruplexes.

*We thank the reviewer for the suggestions and we incorporate these changes in the manuscript. In particular, we are grateful for the suggestions relating to a different manuscript and we will also include Quesnel-Vallières et al. in our G-quadruplex manuscript.*

**Second round of review**

**Reviewer 1**

The revised manuscript has addressed all my comments and more. It has been considerably strengthened and I am happy to recommend it for acceptance in its current form.
